# Design and Synthesis of 56 Shape‐Diverse 3D Fragments

**DOI:** 10.1002/chem.202001123

**Published:** 2020-07-08

**Authors:** Thomas D. Downes, S. Paul Jones, Hanna F. Klein, Mary C. Wheldon, Masakazu Atobe, Paul S. Bond, James D. Firth, Ngai S. Chan, Laura Waddelove, Roderick E. Hubbard, David C. Blakemore, Claudia De Fusco, Stephen D. Roughley, Lewis R. Vidler, Maria Ann Whatton, Alison J.‐A. Woolford, Gail L. Wrigley, Peter O'Brien

**Affiliations:** ^1^ Department of Chemistry University of York Heslington York YO10 5DD UK; ^2^ Asahi Kasei Pharma Corporation 632-1 Mifuku, Izunokuni Shizuoka 410-2321 Japan; ^3^ Vernalis (R&D) Ltd Granta Park, Abington Cambridge CB21 6GB UK; ^4^ Medicine Design, Pfizer Inc 445 Eastern Point Road Groton CT 06340 USA; ^5^ Discovery Sciences, R&D AstraZeneca Cambridge CB4 0WG UK; ^6^ Eli Lilly and Company Limited Erl Wood Manor, Sunninghill Road Windlesham Surrey GU20 6PH UK; ^7^ Astex Pharmaceuticals 436 Cambridge Science Park, Milton Road Cambridge CB4 0QA UK; ^8^ Medicinal Chemistry, Oncology R&D AstraZeneca CB4 0WG Cambridge UK

**Keywords:** 3D fragments, conformational diversity, fragment-based drug discovery, medicinal chemistry, synthesis design

## Abstract

Fragment‐based drug discovery is now widely adopted for lead generation in the pharmaceutical industry. However, fragment screening collections are often predominantly populated with flat, 2D molecules. Herein, we describe a workflow for the design and synthesis of 56 3D disubstituted pyrrolidine and piperidine fragments that occupy under‐represented areas of fragment space (as demonstrated by a principal moments of inertia (PMI) analysis). A key, and unique, underpinning design feature of this fragment collection is that assessment of fragment shape and conformational diversity (by considering conformations up to 1.5 kcal mol^−1^ above the energy of the global minimum energy conformer) is carried out prior to synthesis and is also used to select targets for synthesis. The 3D fragments were designed to contain suitable synthetic handles for future fragment elaboration. Finally, by comparing our 3D fragments with six commercial libraries, it is clear that our collection has high three‐dimensionality and shape diversity.

## Graphical abstract

**Thinking in 3D**: 56 3D (rather than sp^3^‐rich) disubstituted pyrrolidine and piperidine fragments were designed and synthesised. Computationally generating global minimum energy and higher‐energy (but accessible) conformers of potential 3D fragments and assessing their shape by principal moments of inertia analysis prior to synthesis ensured that the collection was significantly three‐dimensional and shape diverse, as demonstrated by comparison with six commercial fragment libraries.

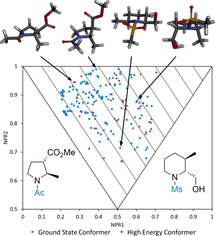

## Introduction

Over the past 20 years, fragment‐based drug discovery (FBDD) has developed into a well‐established method for hit and lead generation.^1^ To date, three approved anti‐cancer drugs, Vemurafenib,^2^ Venetoclax^3^ and Erdafitinib^4^ have originated from FBDD campaigns, with 30 additional compounds having entered clinical trials.^5^ Due to the low molecular weight (MW) of fragments (MW typically <300 Da),^6^ establishing and employing a fragment library that can effectively sample chemical space (typically a few thousand compounds) is far cheaper and more straightforward than establishing a high‐throughput screening library.1d, 5, 7 However, due to their small size, care must be taken with the design of fragment libraries to make them suitable for the generation of high quality starting points for drug.

Although the physicochemical properties of fragment libraries often follow the widely accepted ‘rule‐of‐three’,^6^ little attention is generally paid to shape diversity within fragment collections—indeed, sp^2^ rich compounds with planar, aromatic ring systems predominate.^8^, ^9^, ^10^ 3D fragments are increasingly being considered as complementary to their 2D counterparts and as crucial components of well‐rounded screening libraries^8^, ^11^, ^12^ since they improve the coverage of chemical space and the overall diversity of the library. Of course, it is possible that, being more complex than their planar counterparts, 3D fragments would lead to reduced hit rates.^7^, ^13^ However, the use of 3D fragments may offer advantages in terms of pharmacophore coverage and solubility, leading to better starting points for lead generation.1d, 14, 15 It has also been suggested that a highly shape diverse library could display a broader range of biological activities and be useful in generating hits for challenging targets.^8^, ^9^

To meet this developing need for representation of 3D compounds in fragment libraries, there have been several reports on the synthesis of 3D fragments,^16^, ^17^ including the use of diversity oriented synthesis,^9^, ^12^, ^18^ and natural product‐based approaches ^[10, 19]^ as well as a set of fluorinated fragments.^20^ Furthermore, several 3D fragment libraries are commercially available (e.g. Life Chemicals 3D Fragment Library, ChemDiv 3D FL Fragment Library, Enamine 3D Shape Diverse Fragment Library). In most cases, the assessment of the three‐dimensionality of commercial 3D libraries is performed by analyzing the fraction of sp^3^ carbons (Fsp^3^) and, whilst it has been shown that increasing Fsp^3[14]^ and controlling the number of aromatic rings^21^ in a potential drug candidate can aid drug development, these descriptors are poor surrogates for measuring the *three‐dimensionality* of a molecule.^7^ Two commonly used methods for assessing 3D shape are plane‐of‐best‐fit^22^ and principal moments of inertia (PMI)^23^ analysis. In both cases, the 3D shape of molecular mechanics‐computed global minimum energy conformers of molecules can be easily compared and there is a good correlation between plane‐of‐best‐fit and PMI analyses.^22^ In contrast, and perhaps unsurprisingly, it has been shown that plane‐of‐best‐fit does not correlate with Fsp^3^ for a wide range of medicinally‐relevant compounds.^22^ To further validate the argument that use of Fsp^3^ as a surrogate for three‐dimensionality is flawed, we assessed the correlation between Fsp^3^ and PMI for sets of fragments. Analysis of six commercially available 2D and 3D fragment libraries was performed by calculating PMI values for a random 1000 compounds (for each library) and comparing with Fsp3. No correlation was found (see Supporting Information for details). Furthermore, PMI analysis of these six commercially available fragment libraries showed that the 3D libraries (typically designed using Fsp^3^ as a guide) have only a marginally better 3D profile compared to the standard 2D rich commercial fragment libraries (see Supporting Information for a detailed analysis).

Given that most commercial fragment libraries appear to contain a limited number of 3D shaped fragments, we set out to synthesise a library of ≈50 3D fragments that would specifically occupy the under‐represented areas of fragment space (as determined by PMI analyses of the conformations of fragments). Our 3D collection would be available to supplement commercially available screening collections and thereby provides alternative starting points in FBDD programs. At the outset, the following key design criteria for our workflow were devised: (i) 3D fragments would be based on disubstituted pyrrolidines and piperidines since these heterocycles are ubiquitous in bioactive molecules, being the most common five‐ and six‐membered ring nitrogen heterocycles found in FDA‐approved drugs;^24^ (ii) 3D fragments would be designed to possess properties broadly within ‘rule‐of‐three’ fragment space (MW<300 Da, ClogP<3, number of hydrogen bond acceptors (HBA) and donors (HBD)≤3);^6^ (iii) 3D shape analysis using PMI plots would be an integral part of the 3D fragment design protocol and used to select compounds for synthesis to ensure that we were targeting novel fragment space; (iv) uniquely, conformational diversity of 3D fragments would be achieved by assessing the 3D shape of all conformations up to 1.5 kcal mol^−1^ above the energy of the global minimum energy conformer for each fragment; (v) all of the 3D fragments would be synthesis‐enabled via a readily functionalisable secondary amino group. Of note, design criteria (iii) and (iv) are distinct to previous approaches^8^, ^9^, 17b, 17c, 17f, 17g where PMI analysis of *global minimum energy conformers* is used, mostly retroactively, to assess 3D shape. Herein, using design criteria (i)–(iv), we report the design, synthesis and analysis of a unique collection of 56 shape‐diverse pyrrolidine and piperidine 3D fragments.

## Results and Discussion

**Figure 1:**
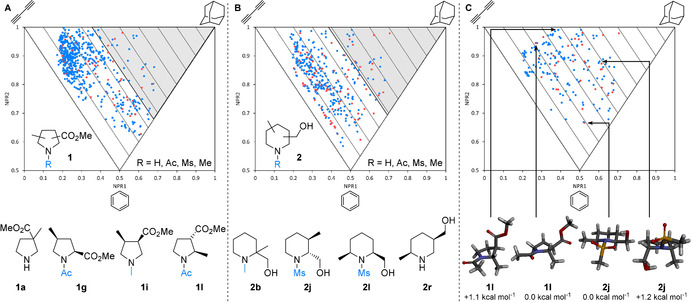
PMI analysis of potential fragments. A: Conformers of pyrrolidine scaffold **1** (top) and exemplar fragments (bottom). B: Conformers of piperidine scaffold **2** (top) and exemplar fragments (bottom). Compounds with conformations within the grey areas were selected for synthesis. C: Conformers of 33 selected fragments (top) and global minimum energy and selected higher energy 3D conformers of **1 l** and **2 j**. Red dots indicate global minimum energy conformers and blue dots indicate higher energy conformers.

Our overall approach was to design a set pyrrolidine and piperidine 3D fragments and to select compounds for synthesis by considering the computational PMI analysis of the 3D shape of their conformations up to 1.5 kcal mol^−1^ above the energy of the global minimum energy conformer. Although the choice of 1.5 kcal mol^−1^ had an arbitrary element, we were keen to consider accessible conformations—for example, at 37 °C, a conformer that was 1.5 kcal mol^−1^ above the energy of the global minimum energy conformer would be present in ≈8 %. Thus, to start, we virtually enumerated and analysed all possible regio‐ and diastereomers arising from pyrrolidine scaffold **1** (Figure 1 A), substituted with an ester and a methyl group, and from piperidine scaffold **2** (Figure 1 B), substituted with a hydroxymethyl and a methyl group. Both scaffolds were decorated with either an acetyl, mesyl, methyl or proton at the nitrogen, giving 56 and 92 possible racemic or achiral isomers for **1** and **2** respectively.^25^ Despite such apparently simple design criteria, the majority of these 148 compounds were in fact novel. Representative 3D fragments include pyrrolidines **1 a**, **1 g**, **1 i** and **1 l** and piperidines **2 b**, **2 j**, **2 l** and **2 r** (Figure 1 A and 1B). It was envisaged that this approach would lead to a wide range of shape‐diverse fragments with two potential protein binding groups in addition to a hydrophobic methyl group. For these scaffolds, using a Pipeline Pilot protocol described in the Supporting Information, we calculated and constructed the PMI plot for all 955 conformers (582 for **1** and 373 for **2**) up to 1.5 kcal mol^−1^ above the energy of the global minimum energy conformer for each of the 148 compounds (Figure 1 A and B, red dots are global minimum energy conformers and blue dots are higher energy conformers^26^). With triangular PMI plots of the normalized PMIs (NPR1 versus NPR2), the three apexes correspond to disc (bottom), rod (top‐left) and spherical (top‐right) shapes; lines parallel to the rod‐disc axis correspond to ΣNPR values (where ΣNPR=NPR1+NPR2, ranging from 1.00–2.00). Conformations that lie furthest from this rod‐disc axis (in which ΣNPR=1.00), will be of interest as they deviate the most from planarity. It is striking how the enumeration of a representative set of simple disubstituted pyrrolidines and piperidines leads to such a high degree of shape diversity of both the global minimum energy and higher energy conformations (Figure 1 A and 1B)—clearly, elaborate and structurally complex molecules are not a requirement for shape diversity. Using the PMI plots in Figure 1 A and 1B, 3D fragments furthest from the rod‐disc axis were selected for synthesis. For the pyrrolidines **1**, 14 fragments with one or more conformer with ΣNPR≥1.36 (Figure 1 A, grey area) were selected, corresponding to the 25 % most 3D fragments. A similar selection criterion (ΣNPR≥1.39) resulted in 19 piperidine fragments being chosen for synthesis and inclusion in the 3D fragment collection.

The PMI plot of these initially selected 33 pyrrolidines **1** and piperidines **2** (Figure 1 C) shows that the selected 3D fragments have highly 3D conformations and provide excellent coverage of 3D chemical space on the PMI plot. Unlike many fragment collections, there are no conformers occupying the rod‐disc axis and very few within the first 10 % of the PMI plot (ΣNPR<1.10); there are no global minimum energy conformers in the ΣNPR 1.00–1.10 region. Consideration of higher energy conformers provides greater conformer diversity (and therefore shape diversity) than if only the global minimum energy conformers are considered. For example, the lowest energy conformer of pyrrolidine **1 l** has pseudo‐diequatorial substituents and is less three‐dimensional (ΣNPR=1.21) than a higher energy (but readily accessible) conformer with pseudo‐diaxial substituents (ΣNPR=1.38) (Figure 1 C). Similarly, piperidine **2 j** exhibits diequatorial and diaxial conformers with significantly different degrees of three‐dimensionality (ΣNPR=1.19 and 1.48 respectively).

**Scheme 1:**
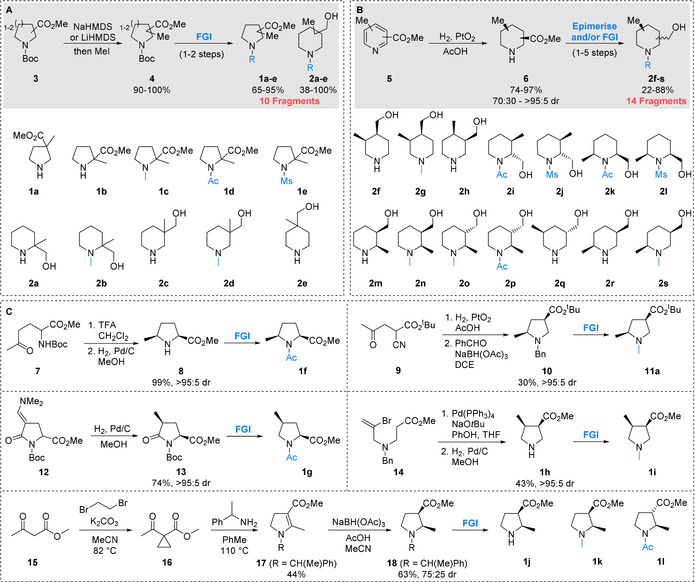
Synthesis of selected 3D fragments.

The structures of the initially selected 3D fragments **1 a**–**l** and **2 a**–**s** are shown in Scheme 1, together with their associated synthetic routes (see Supporting Information for structures of all 33 selected fragments). The PMI‐based compound selection protocol resulted in the identification of geminal disubstituted pyrrolidines **1 a**–**e** and piperidines **2 a**–**e**. Since this geminal disubstitution was present in all of these fragments, they were conveniently accessed through methylation of the enolates^27^ of the requisite Boc protected esters **3**, giving **4** in high yields, followed by simple functional group manipulations (Scheme 1 A). For the 14 selected diastereomeric piperidines **2 f**–**s**, we envisaged that these fragments could be accessed through a unified approach employing an initial stereoselective hydrogenation of disubstituted pyridines **5** (Scheme 1 B).^28^ Treatment of pyridines **5** with hydrogen and 10–30 mol % PtO_2_ gave *cis*‐piperidine esters **6** in good yields and 70:30 to >95:5 dr. The only exception was with a 3,5‐disubstituted piperidine which in fact gave the *trans*‐piperidine ester **6** (and ultimately fragment **2 q**) as the major product.^29^ Subsequent functional group interconversions converted the esters into hydroxymethyl groups and installed the requisite functionality on the secondary amine giving 14 fragments **2 f**–**s**; in the case of **2 i**, **2 j**, **2 o** and **2 p**, epimerisation of *cis*‐esters to *trans*‐esters^30^ using alkoxide bases was used to access the desired *trans*‐isomers (see Supporting Information for full synthetic details).

The remaining pyrrolidine fragments were accessed through different diastereoselective reduction processes, as detailed in Scheme 1 C. First, intermediate 2,5‐*cis*‐pyrrolidine **8** was synthesised in 99 % yield as a single diastereomer through Boc removal from **7** and diastereoselective reduction of the resulting cyclic iminium ion.^31^ Subsequent acetylation gave fragment **1 f**. Similarly, reduction of keto‐nitrile **9** proceeded via an iminium ion and (after *N*‐benzylation) gave 2,4‐*cis* pyrrolidine **10**. *N*‐Benzyl to *N*‐methyl transposition gave fragment **11 a**, the *tert*‐butyl ester analogue of an initially selected target compound.^32^ 2,4‐*cis* Pyrrolidine fragment **1 g** was accessed through stereoselective reduction of enamine **12** to pyrrolidinone **13** followed by functional group interconversions. Intermediates **7** and **12** are available in a single step from a common commercially available building block.^33^ An intramolecular Pd‐catalysed coupling of **14**,^34^ followed by hydrogenation of the resulting α,β‐unsaturated ester with concomitant debenzylation gave 3,4‐disubstituted pyrrolidine fragment **1 h**. Subsequent *N*‐methylation gave fragment **1 i**. Finally, addition of α‐methyl benzylamine to activated cyclopropane **16** (synthesised from β‐ketoester **15**) gave dihydropyrrole **17**.^35^ Reduction^36^ gave *cis*‐pyrrolidine **18** in 72:25 dr, which was subsequently transformed into the desired 2,3‐disubstituted fragments **1 j**–**l**. This synthetic campaign resulted in the synthesis of 31 targeted 3D fragments **1 a**–**l** and **2 a**–**s**, along with a *tert*‐butyl ester analogue of a further fragment **11 a**.

**Figure 2:**
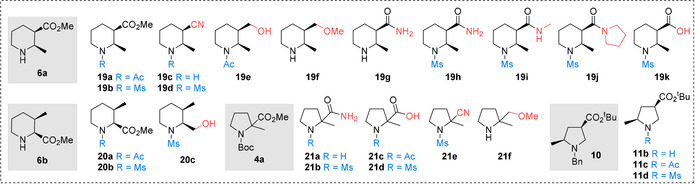
Additional structurally diverse 3D fragments.

To further increase the library diversity and coverage of chemical space, we explored altering the potential protein binding groups. To this end, a further 24 3D fragments that could be accessed from readily available building blocks in an expedient manner were synthesised (Figure 2). Prior to synthesis, a PMI analysis was carried out on all targeted 3D fragments to ensure that they had at least one conformation with ΣNPR value >1.10. 2,3‐Disubstituted piperidine **6 a**, itself a 3D fragment, was first manipulated to give simple *N*‐functionalised fragments **19 a** and **19 b**. Alternatively, the ester group was modified to introduce other hydrogen bonding motifs to give nitriles **19 c** and **19 d**, alcohol **19 e**, ether **19 f**, amides **19 g**–**j** and acid **19 k**. Likewise, building block **6 b** was modified to give piperidines **20 a**–**c**. Further structural diversity was introduced into the collection through the modification of pyrrolidine building blocks **4 a** and **10**, resulting in nine fragments **21 a**–**f** and **11 b**–**d**.

In total, a collection of 56 designed 3D fragments encompassing medicinally‐relevant disubstituted piperidines and pyrrolidines that targeted under‐represented areas of fragment space was synthesised. Despite the simplicity of these fragments, it is notable that 42 are in fact novel molecules. Calculation of the physicochemical properties showed that almost all fragments conformed to the ‘rule‐of‐three’ (Table 1). Of particular note, the mean lipophilicity of the collection (ClogP 0.54) is low in comparison with commercially available fragment libraries (see Supporting Information for full details), making these compounds excellent starting points for lead discovery programs.^37^, ^38^ The stability and solubility of the fragments was assessed to ensure that they were suitable for incorporation into a screening collection. Of the 56 fragments, 52 fragments were stable to prolonged storage on the bench and in DMSO stock solutions (>6 weeks). Of these, 48 fragments were stable in aqueous buffer for >24 h. Crucially, 40 fragments were soluble at a concentration of >0.5 mm in aqueous buffer (see the Supporting Information) and are therefore suitable for biophysical screening.1e

**Table 1:** Mean physicochemical properties of the synthesised 3D fragment collection.

Property^[a]^	Ideal range^[b]^	Calculated values
MW	≤300	173±38
ClogP	≤3	0.54±0.55
HBA	≤3	2.68±0.73
HBD	≤3	0.89±0.70
RBC	≤3	1.64±0.77
TPSA/ Å^2^	≤60	46.7±19.1

[a] MW=molecular Weight, HBA=number of hydrogen bond acceptors, HBD=number of hydrogen bond donors, RBC=rotatable bond count, TPSA=topological polar surface area. [b] ‘Rule‐of‐three’ guidelines.^5^

**Figure 3:**
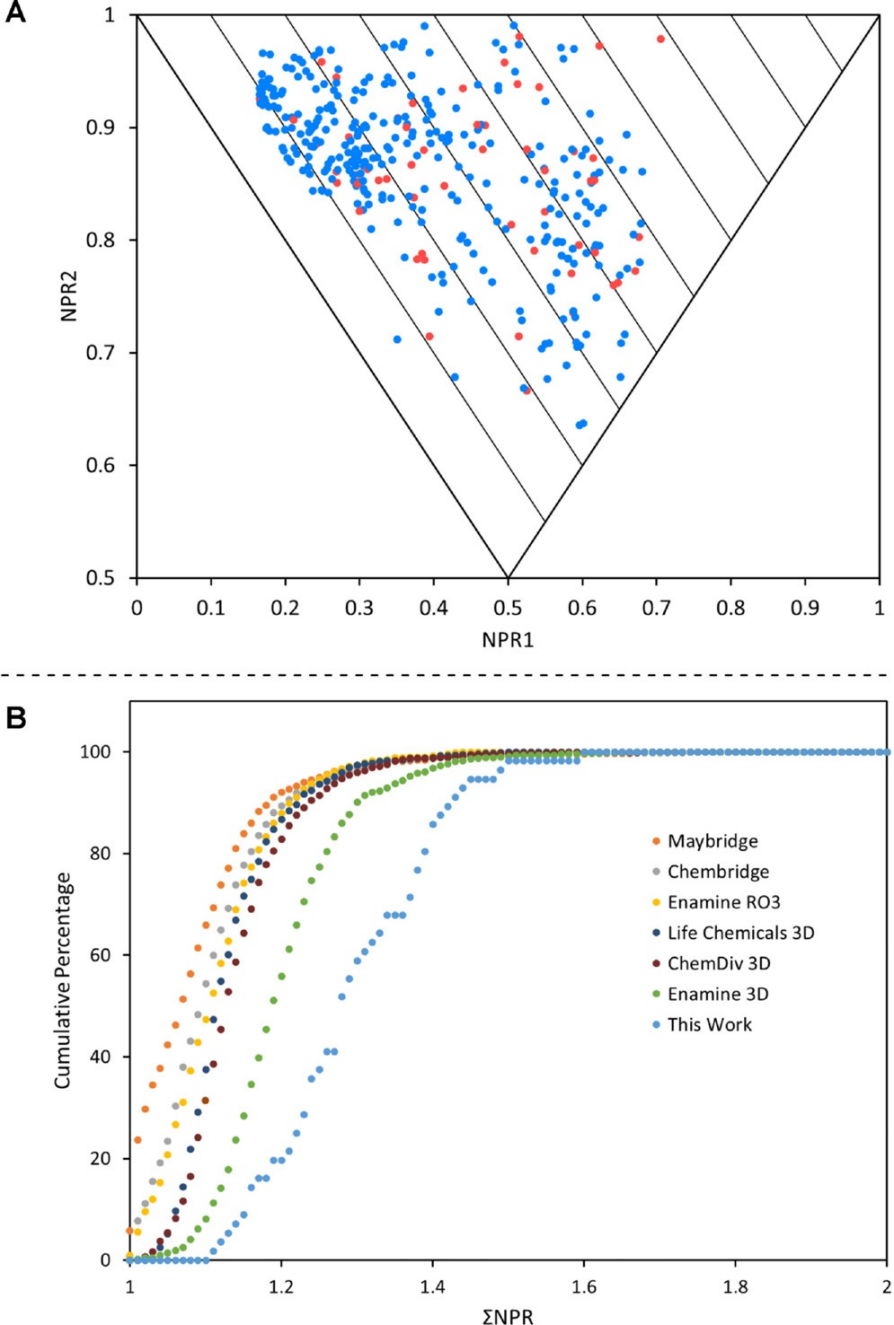
A: PMI plot of the final fragment collection. Red dots indicate global minimum energy conformers and blue dots indicate higher energy conformers. B: Cumulative PMI analysis of the fragment collection (light blue) along with six commercially available libraries.

The PMI plot of the 56 3D fragments is shown in Figure 3 A, clearly demonstrating that our fragments target conformations far from the rod‐disc axis and with a wide‐ranging spread throughout the plot. Finally, to show that our fragments targeted under‐represented areas of fragment space, we compared this collection of 3D fragments with six commercial fragment libraries, including three that were designed to be 3D in nature (Life Chemicals 3D Fragment Library, ChemDiv 3D FL Fragment Library, Enamine 3D Shape Diverse Fragment Library). Using a random selection of 1000 compounds from each of the six commercial fragment libraries, all conformers (up to 1.5 kcal mol^−1^ above the energy of the global minimum energy conformer) were generated (see the Supporting Information for full details). Then, the mean distance from the rod‐disc axis (ΣNPR) was determined for each fragment, based on its conformations. Figure 3 B shows the cumulative percentage of fragments within a defined mean distance from the rod–disc axis (ΣNPR). The fact that our 3D fragments are the furthest to the right on this plot highlight that they are more three‐dimensional than even commercially available 3D fragment libraries. Interestingly, visual inspection of some of the conformers showed the presence of internal hydrogen bonds. Since such conformers are unlikely to exist under physiological conditions, care must be taken to fully interrogate the conformations generated from such molecular mechanics‐generated PMI analyses. It is clear that this is an inherent issue with all molecular shape analyses that depend upon simple conformer generation within computational software packages such as Pipeline Pilot.

## Conclusions

In conclusion, we have developed a workflow to design and select 3D, rather than sp^3^‐rich, fragments by generating global minimum energy conformers and low‐energy conformers of potential fragments and assessing shape by PMI analysis. This approach leads to conformational diversity in addition to 3D shape diversity. We have used this approach to generate a collection of 56 3D fragments based on disubstituted pyrrolidine and piperidine cores that are suitable for inclusion into existing screening libraries and possess synthetic handles for fragment elaboration. The majority of fragments adhere to recommended ‘rule‐of‐three’ guidelines for physicochemical properties, as well as solubility and stability guidelines whilst covering under‐represented areas of fragment space. Furthermore, this library covers diverse and typically unrepresented pharmacophores. The majority of these 3D fragments are available for protein screening at the Diamond‐XChem facility.^39^ It is envisaged that the workflow demonstrated herein could be applied to many analogous potential 3D fragments and new synthetic methodologies, thus enabling the generation of other fit‐for‐purpose 3D fragments.

## Conflict of interest

The authors declare no conflict of interest.

## Supporting information

As a service to our authors and readers, this journal provides supporting information supplied by the authors. Such materials are peer reviewed and may be re‐organized for online delivery, but are not copy‐edited or typeset. Technical support issues arising from supporting information (other than missing files) should be addressed to the authors.

Supplementary
