# Supplementary material for: Design and Synthesis of 56 Shape‐Diverse 3D Fragments
Source: Chemistry. 2020 Jul 8;26(41):8969–75. doi: 10.1002/chem.202001123 (PMC7496344; doi:10.1002/chem.202001123)
Supplement: Supplementary file 1 — Supplementary [file CHEM-26-8969-s001.pdf]

Chemistry–A European Journal

Supporting Information

Design and Synthesis of 56 Shape-Diverse 3D Fragments

Thomas D. Downes+,[a] S. Paul Jones+,[a] Hanna F. Klein+,[a] Mary C. Wheldon,[a] Masakazu Atobe,[a, b] Paul S. Bond,[a] James D. Firth,[a] Ngai S. Chan,[a] Laura Waddelove,[a] Roderick E. Hubbard,[a, c] David C. Blakemore,[d] Claudia De Fusco,[e] Stephen D. Roughley,[c] Lewis R. Vidler,[f] Maria Ann Whatton,[f] Alison J.-A. Woolford,[g] Gail L. Wrigley,[h] and Peter O'Brien*[a]

Table of Contents

1. Experimental Details S3

1.1 General S3

1.2 General Procedures S4

1.3 Experimental Procedures and Characterisation S8

1.4 Proof of Stereochemistry S88

2. Library Analysis S89

2.1 General S89

2.2 Commercial Fragment Library Details S91

2.3 Relationship between Fsp^3 and ΣNPR for Commecial Libaries S92

2.4 Cumulative PMI analysis of commercially available libraries S96

2.5 Selected Fragments S97

2.6 Synthesised Fragments S98

2.7 Molecular Properties of Synthesised Fragments and Commercial Libraries S99

2.8 Calculated Boltzmann Populations of Molecular Mechanics-Computed Conformers S104

3. ^{1}H and ^{13}C NMR Spectra S105

4. References S231

1. Experimental Details

1.1. General

All non-aqueous reactions were carried out under oxygen free Ar or N_2 using flame-dried glassware. Et_2O and THF were freshly distilled from sodium and benzophenone. Alkyllithiums were titrated against *N*-benzylbenzamide before use. Brine refers to a saturated solution. Water is distilled water.

Flash column chromatography was carried out using Fluka Chemie GmbH silica (220-440 mesh). Thin layer chromatography was carried out using commercially available Merck F_{254} aluminium backed silica plates. Proton (400 MHz) and carbon (100.6 MHz) NMR spectra were recorded on a Jeol ECX-400 instrument using an internal deuterium lock. For samples recorded in $CDCl_3$, chemical shifts are quoted in parts per million relative to $CHCl_3$ (δ_H 7.26) and $CDCl_3$ (δ_C 77.0, central line of triplet). For samples recorded in DMSO-d_6, chemical shifts are quoted in parts per million relative to DMSO-d_6 (δ_H 2.50, central line of quintet) and DMSO-d_6 (δ_C 39.52, central line of septet). For samples recorded in MeOH-d_4, chemical shifts are quoted in parts per million relative to MeOH-d_4 (δ_H 3.31, central line of quintet) and MeOH-d_4 (δ_C 50-41, central line of septet). Carbon NMR spectra were recorded with broad band proton decoupling and assigned using DEPT experiments. Coupling constants (J) are quoted in Hertz. Melting points were carried out on a Gallenkamp melting point apparatus. Infrared spectra were recorded on a Perkin Elmer UATR Two FT-IR spectrometer. Electrospray high and low resonance mass spectra were recorded at room temperature on a Bruker Daltronics microOTOF spectrometer.

1.2 General Procedures

General procedure A: Methyl Ester formation and Boc protection

Thionyl chloride (1.85-3.50 mL, 25.5-47.8 mmol, 1.1 eq.) was added dropwise over 5 min to a stirred solution of the carboxylic acid (23.2-43.4 mmol, 1.0 eq.) in MeOH (40-100 mL) at 0 °C under Ar. The resulting solution was stirred and heated at reflux for 1 h. The mixture was then allowed to cool to rt and the solvent was evaporated under reduced pressure to give the crude methyl ester. Et_3N (3.24-6.10 mL, 23.2-43.4 mmol, 1.0 eq.) was added to a stirred solution of crude methyl ester in CH_2Cl_2 (20-50 mL) at rt under Ar. Then, a solution of Boc_2O (5.07-10.0 g, 23.2-43.4 mmol, 1.0 eq.) in CH_2Cl_2 (20-50 mL) was added. The resulting solution was stirred at rt for 18 h. The solids were removed by filtration and the filtrate was evaporated under reduced pressure to give a wet solid. The wet solid was dissolved in Et_2O (50 mL) and washed with 1 M $HCl_{(aq)}$ (2 × 25 mL) and saturated $NaHCO_{3(aq)}$ (10 mL). The organic layer was dried ($MgSO_4$) and evaporated under reduced pressure to give the crude product.

General procedure B: *N*-Methylation using $NaBH(OAc)_3$

$NaBH(OAc)_3$ (0.32-1.3 g, 1.50-6.12 mmol, 1.5-3.0 eq.) was added portionwise to a stirred solution of secondary amine (1.0-2.04 mmol, 1.0 eq.), 37% $HCHO_{(aq)}$ (0.74-4.52 mL, 10-61 mmol, 10.0 eq.) and $MgSO_4$ (0.7-1.5 g) in 4:1 CH_2Cl_2-AcOH (5-17 mL) at 0 °C under air. The resulting solution was allowed to warm to rt and stirred at rt for 2 h. Then, sat. $NH_4OH_{(aq)}$ (20 mL) was added and the mixture was extracted with CH_2Cl_2 (3 × 20 mL). The combined organic extracts were dried ($MgSO_4$) and evaporated under reduced pressure to give the crude product.

General procedure C: Amine Acetylation

Ac_2O (0.22-7.2 mL, 2.30-76.0 mmol, 6.0 eq.) was added dropwise to a stirred solution of the secondary amine (0.39-12.7 mmol, 1.0 eq.) in pyridine (2-12 mL) at rt under Ar and the resulting solution was stirred at rt for 1 h. Then, the mixture was evaporated under reduced pressure to give the crude product.

General procedure D: Amine Mesylation

MsCl (0.07-1.8 mL, 0.96-23.0 mmol, 2.2-3.3 eq.) was added dropwise to a stirred solution of Et_3N (0.13-1.0 mL, 0.96-7.50 mmol, 1.0-4.8 eq.) and amine (0.29-7.5 mmol, 1.0 eq.) in CH_2Cl_2 (5-20 mL) at rt under Ar. The resulting solution was stirred at rt for 18 h. The mixture was poured into water (10 mL) and extracted with CH_2Cl_2 (3 × 10 mL). The combined organics were dried ($MgSO_4$) and evaporated under reduced pressure to give the crude product.

General procedure E: Enolate alkylation with KHMDS

Potassium bis(trimethylsilyl)amide (15 mL of a 0.5 M solution in toluene, 7.50 mmol, 1.5 eq.) was added to a stirred solution of methyl ester (1.22 g, 5.00 mmol, 1.0 eq.) in THF (10 mL) at –78 °C under Ar. The resulting mixture was stirred at or –78 °C for 1 h. Then, methyl iodide (0.47 mL, 7.50 mmol, 1.5 eq.) was added. The resulting solution was allowed to warm to rt over 4 h and then stirred at rt for 16 h. Saturated $NH_4Cl_{(aq)}$ was added and the mixture was extracted with Et_2O. The combined organic extracts were dried ($MgSO_4$) and evaporated under reduced pressure to give the crude product.

General procedure F: Reduction of methyl esters

A solution of the methyl ester (0.39-5.10 mmol, 1.0 eq.) in THF (3-5 mL) was added dropwise to a stirred suspension of $LiAlH_4$ (30-380 mg, 0.78-10.0 mmol, 1.0-3.0 eq.) in THF (6-65 mL) at 0 °C under Ar. The resulting mixture was stirred at 0 °C for 1 h and then 2 M $NaOH_{(aq)}$ (1 µL per 1 mg of $LiAlH_4$), Et_2O (20 mL) and $MgSO_4$ were carefully added. The mixture was allowed to warm to rt and the solids were removed by filtration through Celite and evaporated under reduced pressure to give the crude product.

General Procedure G: Reduction of *N*-Boc to *N*-Me

A solution of the *N*-Boc piperidine (0.88-1.56 mmol, 1.0 eq.) in THF (20 mL) was added dropwise to a stirred suspension of $LiAlH_4$ (165-295 mg, 4.36-7.78 mmol, 5.0 eq.) in THF (30 mL) at 0 °C under Ar. The resulting mixture was allowed to warm to rt for 30 min and then stirred and heated at reflux for 24 h. After being allowed to cool to rt, the mixture was cooled to 0 °C then H_2O (1 µL per mg of $LiAlH_4$), 20% $NaOH_{(aq)}$ (2 µL per mg of $LiAlH_4$) and H_2O (1 µL per mg of $LiAlH_4$) were carefully added. The mixture was allowed to warm to rt and stirred for 30 min. $MgSO_4$ was added and the mixture stirred for 30 min. Then, the solids were removed by filtration through Celite and the filtrate was evaporated under reduced pressure to give the crude product.

General procedure H: Pyridine methyl ester formation

Thionyl chloride (58-291 µL, 0.8-4.0 mmol, 1.1 eq.) was added dropwise over 5 min to a stirred solution of the carboxylic acid (0.73-3.65 mmol, 1.0 eq.) in MeOH (5 mL) at 0 °C under Ar. The resulting solution was stirred and heated at reflux for 1 h. The mixture was then allowed to cool to rt and the solvent was evaporated under reduced pressure. Then, the mixture was dissolved in CH_2Cl_2 (50 mL) and washed with saturated $NaHCO_{3(aq)}$ (10 mL). The organic layer was dried ($MgSO_4$) and evaporated under reduced pressure to give the crude product.

General procedure I: Pyridine hydrogenation and neutralisation

PtO_2 (22-250 mg, 10-30 mol%) or 10% Pd/C (70 mg, 10 mol%) was added to a stirred solution of pyridine ester (0.1-1.0 g, 0.66-6.62 mmol) in AcOH (3-10 mL) at rt under Ar. The reaction flask was evacuated under reduced pressure and back-filled with Ar three times. After the final evacuation, H_2 was charged and the reaction mixture was stirred vigorously under a balloon of H_2 for 24 h. The solids were removed by filtration through Celite and washed with MeOH (30-100 mL). The filtrate was evaporated under reduced pressure to give the crude product. The crude product was dissolved in CH_2Cl_2 (10 mL) and $NH_4OH_{(aq)}$ (10 mL) was added. The two layers were separated and the aqueous layer was extracted with CH_2Cl_2 (3 × 50 mL). The combined organic layers were dried (Na_2SO_4) and evaporated under reduced pressure to give the crude product.

General Procedure J: Amide formation with T3P

DIPEA (132-917 µL, 0.76-5.26 mmol, 3.0 eq.) and T3P (0.11-1.57 mL of a 50% wt solution in EtOAc, 1.14 mmol, 1.5-3.0 eq.) were added sequentially to a stirred solution of the acid (0.25-1.75 mmol, 1.0 eq.) in CH_2Cl_2 (3-40 mL) at rt under Ar. Then, the amine (0.28-8.77 mmol, 1.1-5.0 eq.) was added and the resulting solution was stirred and heated at 55 °C for 3 h. The solution was poured into water (20 mL) and 3 M $HCl_{(aq)}$ (5 mL) was added. The two layers were separated and the aqueous layer was extracted with EtOAc (3 × 20 mL). The combined organics were washed with 2 M $NaOH_{(aq)}$ (20 mL) and brine (20 mL), dried ($MgSO_4$) and evaporated under reduced pressure to give the crude product as a yellow oil.

General Procedure K: Amide dehydration

Et_3N (88-656 µL, 0.63-4.70 mmol, 2.4 eq.) and trifluoroacetic anhydride (45-332 µL, 0.32-2.35 mmol, 1.2 eq.) were sequentially added dropwise to a stirred solution of the amide (0.26-1.96 mmol, 1.0 eq.) in THF (7-35 mL) at 0 °C under Ar. The resulting solution was stirred at 0 °C for 1 h. After being allowed

to warm to rt, CH_2Cl_2 (20 mL) was added and the solution was washed with saturated $NaHCO_{3(aq)}$ (20 mL). The aqueous layer was extracted with CH_2Cl_2 (2 × 20 mL). The combined organics were dried (Na_2SO_4) and evaporated under reduced pressure to give the crude product.

1.3 Experimental Procedures and Characterisation

1-*tert*-Butyl 3-methyl 3-methylpyrrolidine-1,3-dicarboxylate 4b

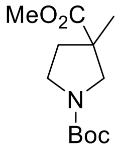

4b

Lithium bis(trimethylsilyl)amide (1.0 M solution in THF, 6.0 mL 6.0 mmol, 1.2 eq.) was added to a stirred solution of methyl 1-Boc-3-pyrrolidinecarboxylate (1.15 g, 5.0 mmol, 1.0 eq.) in THF (15 mL) at –78 °C under Ar. The resulting mixture was stirred at –78 °C for 1 h. Then, methyl iodide (436 µL, 7.0 mmol, 1.4 eq.) was added. The resulting solution was stirred at –78 °C for 1 h then allowed to warm to rt and stirred at rt for 2 h. Saturated $NH_4Cl_{(aq)}$ (20 mL) was added and the mixture was extracted with EtOAc (3 × 30 mL). The combined organic extracts were dried ($MgSO_4$) and evaporated under reduced pressure to give the crude product. Purification by flash column chromatography on silica with 80:20 hexane-EtOAc as eluent gave **4b** (1.09 g, 90%) as a colourless oil, R_F (75:25 hexane-EtOAc) 0.24; IR (ATR) 2880, 1733 (C=O, CO_2Me), 1694 (C=O, Boc), 1398, 1366, 1279, 1161, 1138, 1099 cm^{-1}; ^1H NMR (400 MHz, $CDCl_3$) (55:45 mixture of rotamers) δ 3.81–3.68 (m, 1H, NCH), 3.70 (s, 3H, OMe), 3.47-3.35 (m, 2H, NCH), 3.22 (d, J = 11.0 Hz, 0.45H, NCH), 3.16 (d, J = 11.0 Hz, 0.55H, NCH), 2.37–2.24 (m, 1H, CH), 1.80–1.70 (m, 1H, CH), 1.45 (s, 9H, CMe_3), 1.32 (s, 3H, CMe); ^{13}C NMR (100.6 MHz, $CDCl_3$) (rotamers) δ 176.0 (C=O, CO_2Me), 154.5 (C=O, Boc), 79.4 (O*C*Me_3), 79.3 (O*C*Me_3), 55.0 (NCH_2), 54.7 (NCH_2), 52.3 (OMe), 48.7 (*C*Me), 47.9 (*C*Me), 44.9 (NCH_2), 44.5 (NCH_2), 35.8 (CH_2), 35.0 (CH_2), 28.5 (C*Me*$_3$), 22.4 (C*Me*), 22.3 (C*Me*); HRMS (ESI) *m/z* calcd for $C_{12}H_{21}NO_4$ $(M + Na)^+$ 266.1363, found 266.1364 (–0.5 ppm error).

Lab book reference JDF_B_399

Methyl 3-methylpyrrolidine-3-carboxylate hydrochloride 1a

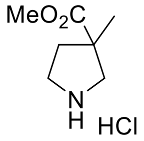

1a

HCl (2.0 mL of a 2 M solution in Et_2O, 4.0 mmol, 4.0 eq.) was added to pyrrolidine **4b** (243 mg, 1.0 mmol, 1.0 eq.) and the resulting solution was stirred at rt for 16 h. Then, the solvent was evaporated under reduced pressure to give **1a**·HCl (170 mg, 95%), as a clear oil, IR (ATR) 2953, 2743, 1729 (C=O), 1632, 1453, 1290, 1215, 1135, 985 cm^{-1}; ^{1}H NMR (400 MHz, $CDCl_3$) δ 9.95 (br s, 1H, NH), 9.72 (br s, 1H, NH), 3.79–3.72 (m, 1H, NCH), 3.74 (s, 3H, OMe), 3.58–3.31 (m, 2H, NCH), 3.15 (ddd, J = 12.0, 7.0, 5.0 Hz, 1H, NCH), 2.45 (ddd, J = 13.5, 7.5, 5.5 Hz, 1H, CH), 1.94 (ddd, J = 13.5, 8.0, 8.0 Hz, 1H, CH), 1.44 (s, 3H, CMe); ^{13}C NMR (100.6 MHz, $CDCl_3$) δ 174.3 (C=O), 52.9 (OMe), 52.9 (NCH_2), 48.9 (*C*Me), 44.5 (NCH_2), 35.4 (CH_2), 22.2 (C*Me*); HRMS (ESI) *m/z* calcd for $C_7H_{14}NO_2$ M^+ 144.1019, found 144.1018 (0.5 ppm error).

Lab book reference JDF_B_400

1-*tert*-Butyl 2-methyl pyrrolidine-1,2-dicarboxylate 3a

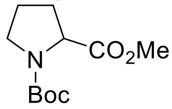

3a

Using general procedure A, thionyl chloride (3.5 mL, 47.8 mmol, 1.1 eq.) and proline (5.0 g, 43.4 mmol, 1.0 eq.) in MeOH (100 mL) then Et_3N (6.1 mL, 43.4 mmol, 1.0 eq.) in CH_2Cl_2 (50 mL) and Boc_2O (10.0 g, 43.4 mmol, 1.0 eq.) in CH_2Cl_2 (50 mL) gave the crude product. Purification by flash column chromatography on silica with 4:1 hexane-EtOAc as eluent gave methyl ester **3a** (766 mg, 77% over 2 steps) as a pale yellow oil, R_F (2:1 hexane-EtOAc) 0.45; IR (ATR) 2976, 2880, 1746 (C=O, CO_2Me), 1695 (C=O, Boc), 1391, 1157, 771 cm^{-1}; ^{1}H NMR (400 MHz, $CDCl_3$) (60:40 mixture of rotamers) δ 4.30 (dd, J = 8.5, 3.5 Hz, 0.4H, NCHCO), 4.20 (dd, J = 8.5, 4.0 Hz, 0.6H, NCHCO), 3.70 (m, 3H, OMe), 3.60-3.28 (m, 2H, NCH), 2.28-2.08 (m, 1H, CH), 2.01-1.75 (m, 3H, CH), 1.44 (s, 3.6H, CMe_3), 1.39 (s, 5.4H, CMe_3); ^{13}C NMR (100.6 MHz, $CDCl_3$) (rotamers) δ 173.9 (C=O, CO_2Me), 173.6 (C=O, CO_2Me), 154.5 (C=O, Boc), 153.9 (C=O, Boc), 80.0 (O*C*Me_3), 79.9 (O*C*Me_3), 59.2 (NCH), 58.8 (NCH), 52.2

(OMe), 52.0 (OMe), 46.7 (NCH_2), 46.4 (NCH_2), 31.0 (CH_2), 30.0 (CH_2), 28.5 (CMe_3), 28.4 (CMe_3), 24.4 (CH_2), 23.8 (CH_2). Spectroscopic data consistent with those reported in the literature.[1]
Lab Book Reference: HFK 1-001

1-*tert*-Butyl 2-methyl 2-methylpyrrolidine-1,2-dicarboxylate 4a

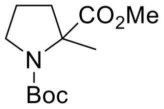

4a

LHMDS (24.4 mL of a 1 M solution in THF, 24.4 mmol, 1.4 eq.) was added dropwise to a stirred solution of **3a** (4.02 g, 17. 5 mmol, 1.0 eq.) in THF (45 mL) at –20 °C under Ar. The resulting solution was stirred at –20 °C for 1.5 h. Then, methyl iodide (1.5 mL, 24.4 mmol, 1.4 eq.) was added dropwise. After being allowed to warm to rt, the resulting solution was stirred at rt for 18 h. Saturated $NH_4Cl_{(aq)}$ (20 mL) and 35% $NH_{3(aq)}$ (20 mL) were added sequentially and the mixture was extracted with EtOAc (3 × 40 mL). The combined organic extracts were washed with brine (3 × 40 mL), dried ($MgSO_4$) and evaporated under reduced pressure to give the crude product as an orange oil. Purification by flash column chromatography on silica with 3:1 hexane-EtOAc as eluent gave methylated product **4a** (3.97 g, 93%) as a pale yellow oil, R_F (3:2 hexane-EtOAc) 0.79; IR (ATR) 2976, 2877, 1741 (C=O, CO_2Me), 1694 (C=O, Boc), 1387, 1161, 773 cm^{-1}; ^1H NMR (400 MHz, $CDCl_3$) (70:30 mixture of rotamers) δ 3.67 (s, 3H, OMe), 3.57-3.42 (m, 2H, NCH), 2.18-2.08 (m, 1H, CH), 1.94-1.78 (m, 3H, CH), 1.52 (s, 0.9H, CMe), 1.47 (s, 2.1H, CMe), 1.40 (s, 2.7H, CMe_3), 1.37 (s, 6.3H, CMe_3); ^{13}C NMR (100.6 MHz, $CDCl_3$) (rotamers) δ 175.4 (C=O, CO_2Me), 175.3 (C=O, CO_2Me), 154.0 (C=O, Boc), 153.6 (C=O, Boc), 79.9 ($OCMe_3$), 79.5 ($OCMe_3$), 65.2 (NCMe), 64.8 (NCMe), 52.2 (OMe), 47.9 (OMe), 40.2 (NCH_2), 39.2 (NCH_2), 28.5 (CH_2), 28.4 (CH_2), 23.4 (CH_2), 23.2 (CH_2), 22.9 (C*Me*), 22.3 (C*Me*); HRMS (ESI) *m/z* calcd for $C_{12}H_{21}NO_4$ $(M + Na)^+$ 266.1363, found 266.1370 (–2.2 ppm error). Spectroscopic data consistent with those reported in the literature.[2]
Lab Book Reference: HFK 1-040

Methyl 2-methylpyrrolidine-2-carboxylate hydrochloride 1b

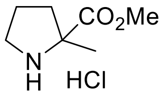

1b

HCl (22.6 mL of a 2 M solution in Et_2O, 45.2 mmol, 4.4 eq.) was added dropwise to a stirred solution of methyl ester **4a** (2.44 g, 10.3 mmol, 1.0 eq.) in Et_2O (20 mL) at rt under Ar. The resulting mixture was stirred and heated at reflux for 23 h. Then, the solvent was evaporated under reduced pressure to give pyrrolidine **1b**·HCl (1.75 g, 97%) as a brown solid, mp 86-94 ºC (lit.,[3] 106-108 ºC); R_F (100:9:1 CH_2Cl_2-MeOH-$NH_4OH_{(aq)}$) 0.67; IR (ATR) 3387 (NH), 2919, 2744, 2508, 1741 (C=O), 1585, 1440, 1290, 1214, 1129, 985, 886, 763 cm^{-1}; ^1H NMR (400 MHz, $CDCl_3$) δ 10.62 (s, 1H, NH), 9.43 (s, 1H, NH), 3.85 (s, 3H, OMe), 3.60-3.57 (m, 2H, NCH), 2.42-2.38 (m, 1H, CH), 2.17-1.97 (m, 3H, CH), 1.86 (s, 3H, CMe); ^{13}C NMR (100.6 MHz, $CDCl_3$) δ 171.3 (C=O), 69.0 (N*C*Me), 53.9 (OMe), 45.4 (NCH_2), 36.0 (CH_2), 22.7 (CH_2), 21.4 (C*Me*); HRMS (ESI) *m/z* calcd for $C_7H_{14}NO_2$ M^+ 144.1019, found 144.1020 (0.3 ppm error). Spectroscopic data consistent with those reported in the literature.[4]

Lab Book Reference: IC 1-46

Methyl 1,2-dimethylpyrrolidine-2-carboxylate hydrochloride 1c

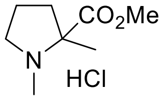

1c

Using general procedure B, $NaBH(OAc)_3$ (882 mg, 4.16 mmol, 3.0 eq.), pyrrolidine **1b** (200 mg, 1.39 mmol, 1.0 eq.), 37% $HCHO_{(aq)}$ (1.13 mL, 13.9 mmol, 10 eq.), $MgSO_4$ (0.7 g) in 4:1 CH_2Cl_2-AcOH (10 mL) gave the crude product. HCl (3.47 mL of a 2 M solution in Et_2O, 6.94 mmol, 5.0 eq.) was added and the resulting solution was stirred at rt for 30 min. The solvent was evaporated under reduced pressure to give pyrrolidine **1c**·HCl (205 mg, 86%) as a yellow oil, R_F (100:9:1 CH_2Cl_2–MeOH–$NH_4OH_{(aq)}$) 0.44; IR (ATR) 3405 (NH), 2957, 2465, 1738 (C=O), 1448, 1286, 1215, 1118, 976, 729, 484 cm^{-1}; ^1H NMR (400 MHz, MeOH-d_4) δ 3.87 (s, 3H, OMe), 3.88-3.83 (m, 1H, NCH), 3.71-3.76 (m, 1H, NCH), 2.90 (s, 3H, NMe), 2.38-2.21 (m, 3H, CH), 2.10-2.00 (m, 1H, CH) 1.61 (s, 3H, CMe); ^{13}C NMR (100.6 MHz, MeOH-d_4) δ 172.1 (C=O), 73.7 (N*C*Me), 55.5 (NCH_2), 54.5 (OMe), 49.8 (CH_2), 36.6 (NMe), 21.3 (C*Me*), 17.1 (CH_2); HRMS (ESI) *m/z* calcd for $C_8H_{16}NO_2$ M^+ 158.1176, found 158.1172 (+2.6 ppm error).

Lab Book Reference: IC 1-52

Methyl 1-acetyl-2-methylpyrrolidine-2-carboxylate 1d

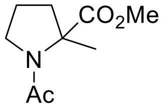

1d

Using general procedure C, Ac_2O (630 µL, 6.68 mmol, 6.0 eq.) and hydrochloride salt **1b**•HCl (223 mg, 1.23 mmol, 1.0 eq.) in pyridine (2 mL) gave the crude product. Purification by flash column chromatography on silica with 19:1-9:1 CH_2Cl_2-MeOH as eluent gave *N*-acyl pyrrolidine **1d** (220 mg, 96%) as a yellow oil, R_F (9:1 CH_2Cl_2-MeOH) 0.73; IR (ATR) 2953, 1737 (C=O, CO_2Me), 1635 (C=O, amide), 1611, 1415, 728 cm^{-1}; ^1H NMR (400 MHz, $CDCl_3$) δ 3.61 (s, 3H, OMe), 3.59-3.47 (m, 2H, NCH), 2.14-2.02 (m, 1H, CH), 1.96 (s, 3H, C(O)Me), 1.98-1.89 (m, 2H, CH), 1.87-1.75 (m, 1H, CH), 1.46 (s, 3H, NCMe); ^{13}C NMR (100.6 MHz, $CDCl_3$) δ 174.5 (C=O), 169.3 (C=O), 65.7 (N*C*Me), 52.4 (NCH_2), 49.0 (OMe), 38.7 (C(O)*Me*), 24.0 (NC*Me*), 22.9 (CH_2), 21.5 (CH_2); HRMS (ESI) *m/z* calcd for $C_9H_{15}NO_3$ $(M + Na)^+$ 208.0944, found 208.0945 (–1.1 ppm error).
Lab book reference HFK1-068

Methyl-1-methanesulfonyl-2-methylpyrrolidine-2-carboxylate 1e

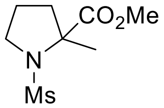

1e

Trifluoroacetic acid (1.0 mL, 13.0 mmol, 16.0 eq.) was added dropwise to a stirred solution of Boc protected ester **4a** (200 mg, 0.82 mmol, 1.0 eq.) in CH_2Cl_2 (5 mL) at rt under Ar. The resulting solution was stirred at rt for 1.5 h. Then, the solvent was evaporated under reduced pressure to give the crude TFA salt (385 mg). Using general procedure D, Et_3N (560 µL, 3.96 mmol, 4.8 eq.) and MsCl (140 µL, 1.80 mmol, 2.2 eq.) in CH_2Cl_2 (4 mL) gave the crude product as a yellow oil. Purification by flash column chromatography on silica with 7:3-1:1 hexane-EtOAc as eluent gave *N*-sulfonamide pyrrolidine **1e** (127 mg, 70%) as a white solid, mp 79-81 °C; R_F (1:1 hexane-EtOAc) 0.30; IR (ATR) 2955, 2882, 1735 (C=O), 1320, 1132, 518 cm^{-1}; ^1H NMR (400 MHz, $CDCl_3$) δ 3.73 (s, 3H, OMe), 3.59-3.44 (m, 2H, NCH), 2.95 (s, 3H, SO_2Me), 2.29-2.12 (m, 1H, CH), 2.05-1.87 (m, 3H, CH), 1.65 (s, 3H, NCMe); ^{13}C NMR (100.6 MHz, $CDCl_3$) δ 174.8 (C=O), 68.9 (N*C*Me), 52.8 (OMe), 49.0 (NCH_2), 41.1 (CH_2), 39.7 (SO_2Me),

24.8 (CH_2), 23.5 (NC*Me*); HRMS (ESI) *m/z* calcd for $C_8H_{15}NO_4S$ $(M + Na)^+$ 244.0614, found 244.0614 (–0.2 ppm error).
Lab book reference HFK2-090

1-*tert*-Butyl 2-methyl piperidine-1,2-dicarboxylate 3c

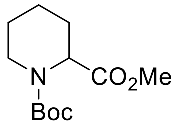

3c

Using general procedure A, thionyl chloride (3.09 mL, 42.6 mmol, 1.1 eq.), pipecolinic acid (5.00 g, 38.7 mmol, 1.0 eq.) in MeOH (50 mL) then Et_3N (5.40 mL, 38.7 mmol, 1.0 eq.) in CH_2Cl_2 (25 mL) and Boc_2O (8.45 g, 38.7 mmol, 1.0 eq.) in CH_2Cl_2 (25 mL) gave the crude product. Purification by flash chromatography on silica with 80:20 hexane-Et_2O as eluent gave methyl ester **3c** (8.88 g, 94%) as a colourless oil, R_F (80:20 hexane-Et_2O) 0.2; ^{1}H NMR (400 MHz, $CDCl_3$) (50:50 mixture of rotamers) δ 4.89 (br s, 0.5H, NCHCO), 4.72 (br s, 0.5H, NCHCO), 4.05-3.87 (m, 1H, NCH), 3.723 (s, 1.5H, OMe), 3.721 (s, 1.5H, OMe), 2.96 (br dd, J = 13.0, 13.0 Hz, 0.5H, NCH), 2.86 (br dd, J = 13.0, 13.0 Hz, 0.5H. NCH), 2.19 (br d, J = 12.0 Hz, 1H, CH), 1.73-1.57 (m, 4H, CH), 1.45 (s, 9H, CMe_3), 1.29-1.15 (m, 1H, CH); ^{13}C NMR (100.6 MHz, $CDCl_3$) (rotamers) δ 172.4 (C=O, CO_2Me), 155.5 (C=O, Boc), 79.9 (O*C*Me_3), 55.0 (NCH), 53.7 (NCH), 52.0 (OMe), 42.1 (NCH_2), 41.0 (NCH_2), 28.3 (C*Me_3*), 26.8 (CH_2), 24.8 (CH_2), 20.8 (CH_2). Spectroscopic data consistent with those reported in the literature.[5]
Lab book reference: mcw/3/44/1

1-*tert*-Butyl 2-methyl 2-methylpiperidine-1,2-dicarboxylate 4c

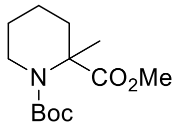

4c

Using general procedure E, potassium bis(trimethylsilyl)amide (15 mL of a 0.5 M solution in toluene, 7.50 mmol, 1.5 eq.) and methyl ester **3c** (1.22 g, 5.00 mmol, 1.0 eq.) in THF (10 mL) and then methyl iodide (0.47 mL, 7.50 mmol, 1.5 eq.) gave the crude product as a 95:5 mixture (by ^{1}H NMR spectroscopy) of methylated methyl ester **4c** and starting methyl ester **3c** (1.26 g, 94% of methylated ester **4c** and 5% of starting ester **3c**) as a colourless oil, R_F (80:20 hexane-Et_2O) 0.2; ^{1}H NMR (400 MHz, $CDCl_3$) for **4c**: δ 3.84 (ddd, J = 13.5, 4.0, 4.0 Hz, 1H, NCH), 3.70 (s, 3H, OMe), 2.95 (br dd, J = 13.5, 13.5 Hz, 1H, NCH), 1.92-1.77 (m, 1H, CH), 1.75-1.53 (m, 5H, CH), 1.40 (s, 9H, CMe_3); ^{13}C NMR (100.6 MHz, $CDCl_3$) for **4c**: δ 175.6 (C=O, CO_2Me), 155.5 (C=O, Boc), 79.9 (O*C*Me_3), 60.3 (NCH_2), 52.0 (OMe), 41.0 (CH_2), 34.8 (CH_2), 28.2 (C*Me_3*), 23.7 (Me), 18.4 (CH_2). Spectroscopic data for **4c** consistent with those reported in the literature.[5]
Lab Book Reference: mcw/3/49/1

***tert*-Butyl 2-(hydroxymethyl)-2-methylpiperidine-1-carboxylate S1**

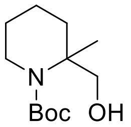

S1

Using general procedure F, a 95:5 mixture of methyl esters **4c** and **3c** (200 mg, 190 mg of **4c** (0.74 mmol) and 10 mg of **3c** (0.041 mmol), 1.0 eq.) in THF (5 mL) and $LiAlH_4$ (30 mg, 0.78 mmol, 1.0 eq.) in THF (30 mL) gave the crude product. Purification by flash chromatography on silica with 70:30 and then 50:50 hexane-Et_2O as eluent gave alcohol **S1** (111 mg, 65% from **4c**) as a colourless oil, R_F (50:50 hexane-Et_2O) 0.4; ^{1}H NMR (400 MHz, $CDCl_3$) δ 4.70 (br s, 1H, OH), 3.85 (br ddd, J = 12.0, 4.0, 4.0 Hz, 1H, NCH), 3.67-3.59 (m, 2H, HOC*H*), 2.96 (ddd, J = 13.5, 12.0, 3.0 Hz, 1H, NCH), 1.68-1.56 (m, 5H, CH), 1.44 (s, 9H, CMe_3), 1.36-1.31 (m, 1H, CH), 1.23 (s, 3H, CMe); ^{13}C NMR (100.6 MHz, $CDCl_3$) δ 156.4 (C=O), 80.0 (O*C*Me_3), 71.4 (OCH_2), 59.5 (*C*Me), 42.5 (NCH_2), 35.4 (CH_2), 28.4 (C*Me_3*), 24.9 (CH_2), 19.6 (CH_2), 18.2 (C*Me*). Spectroscopic data consistent with those reported in the literature.[5]
Lab Book Reference: mcw/3/56/1

2-(Hydroxymethyl)-2-methylpiperidine hydrochloride 2a

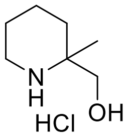

2a

HCl (1.0 M solution in Et_2O, 3.49 mL, 3.49 mmol, 8.0 eq.) was added to a stirred solutiuon of *N*-Boc amino alcohol **S1** (100 mg, 0.44 mmol, 1.0 eq.) in Et_2O (1.75 mL) at rt under Ar. The resulting solution was stirred at rt for 18 h. The solvent was evaporated under reduced pressure to give piperidine **2a**·HCl (68 mg, 94%) as a white solid, mp 120-122 °C; IR (ATR) 3345 (OH or NH), 2943, 2870, 2747, 1671, 1394, 1274, 1253, 1156, 1049 cm^{-1}; ^1H NMR (400 MHz, DMSO-*d*$_6$) δ 8.81 (br s, 1H, NH), 8.36 (br s, 1H, NH), 3.53 (d, *J* = 11.5 Hz, 1H, HOC*H*), 3.38 (d, *J* = 11.5 Hz, 1H, HOC*H*), 2.94 (br s, 2H, NCH_2), 1.70–1.52 (m, 5H, CH), 1.47–1.40 (m, 1H, CH), 1.21 (s, 3H, CMe); ^{13}C NMR (100.6 MHz, DMSO-*d*$_6$) δ 65.0 (OCH_2), 57.3 (N*C*Me), 39.0 (NCH_2), 29.45 (CH_2), 21.5 (CH_2), 18.1 (C*Me*), 17.6 (CH_2); HMRS (ESI) *m/z* calcd for $C_7H_{16}NO$ M^+ 130.1226, found 130.1231 (–3.9 ppm error).
Lab Book Reference: mcw/5/15/c

(1,2-Dimethylpiperidin-2-yl)methanol 2b

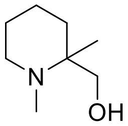

2b

Using general procedure G, *N*-Boc piperidine **S1** (200 mg, 0.88 mmol, 1.0 eq.) in THF (20 mL) and $LiAlH_4$ (165 mg, 4.36 mmol, 5.0 eq.) in THF (30 mL) gave *N*-methyl piperidine **2b** (96 mg, 76%) as a colourless oil, IR (ATR) 3350 (OH), 2930, 2862, 1450, 1371, 1106, 1051, 669 cm^{-1}; ^1H NMR (400 MHz, $CDCl_3$) δ 3.55 (d, *J* = 10.5 Hz, 1H, HOC*H*), 3.08 (d, *J* = 10.5 Hz, 1H, HOC*H*), 2.65 (br d, *J* = 12.0, 1H, NCH), 2.44 (ddd, *J* = 12.0, 12.0, 3.0 Hz, 1H, NCH), 2.17 (s, 3H, NMe), 1.88-1.78 (m, 1H, CH), 1.62-1.57 (m, 2H, CH), 1.49-1.40 (m, 2H), 1.32-1.24 (m, 1H, CH), 0.86 (s, 3H, Me); ^{13}C NMR (100.6 MHz, $CDCl_3$) δ 62.8 (OCH_2), 50.8 (NCH_2), 37.3 (NMe), 34.1 (CH_2), 29.8 (CH_2), 20.5 (CH_2), 11.7 (Me) (*C*Me resonance not resolved); HMRS (ESI) *m/z* calcd for $C_8H_{17}NO$ $(M + H)^+$ 144.1383, found 144.1384 (–0.6 ppm error).
Lab book reference: mcw/3/98

1-*tert*-Butyl 3-methyl piperidine-1,3-dicarboxylate 3d

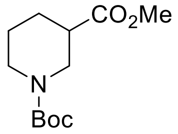

3d

Using general procedure A, thionyl chloride (1.85 mL, 25.5 mmol, 1.1 eq.) and nipecotic acid (3.00 g, 23.2 mmol, 1.0 eq.) in MeOH (40 mL) and then Et_3N (3.24 mL, 23.2 mmol, 1.0 eq.) in CH_2Cl_2 (20 mL) and Boc_2O (5.07 g, 23.2 mmol, 1.0 eq.) in CH_2Cl_2 (20 mL) gave the crude product. Purification by flash chromatography on silica with 80:20 hexane-Et_2O as eluent gave methyl ester **3d** (4.56 g, 81%) as a yellow solid, mp 43-45 °C; R_F (80:20 hexane-Et_2O) 0.2; ^{1}H NMR (400 MHz, $CDCl_3$) δ 4.07 (br s, 1H, NCH), 3.89 (br d, J = 13.0 Hz, 1H, NCH), 3.66 (s, 3H, OMe), 2.96 (br s, 1H, NCH), 2.79 (ddd, J = 13.0, 11.0, 3.0 Hz, 1H, NCH), 2.43 (dddd, J = 10.5, 10.5, 3.5, 3.5 Hz, 1H, C*H*CO_2Me), 2.06-1.98 (m, 1H, CH), 1.72-1.62 (m, 1H, CH), 1.61-1.51 (m, 1H, CH), 1.49-1.35 (m, 10H, CH and CMe_3); ^{13}C NMR (100.6 MHz, $CDCl_3$) δ 173.8 (C=O, CO_2Me), 154.6 (C=O, Boc), 79.6 (O*C*Me_3), 51.7 (OMe), 45.4 (NCH_2), 43.6 (NCH_2), 41.3 (CH), 28.4 (C*Me_3*), 27.3 (CH_2), 24.2 (CH_2). Spectroscopic data consistent with those reported in the literature.[6]

Lab Book reference: mcw/3/15/2

1-*tert*-Butyl 3-methyl 3-methylpiperidine-1,3-dicarboxylate 4d

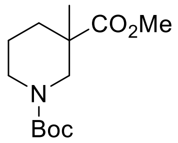

4d

Using general procedure E, potassium bis(trimethylsilyl)amide (15 mL of a 0.5 M solution in toluene, 7.50 mmol, 1.5 eq.) and methyl ester **3d** (1.22 g, 5.00 mmol, 1.0 eq.) in THF (10 mL) and then methyl iodide (0.47 mL, 7.50 mmol, 1.5 eq.) gave the crude product. Purification by flash chromatography on silica with 80:20 hexane-Et_2O as eluent gave methylated piperidine **4d** (1.25 g, 97%) as a colourless oil, IR (ATR) 2974, 2863, 1731 (C=O, CO_2Me), 1689 (C=O, Boc), 1422, 1364, 1277, 1152, 1001, 866 cm^{-1}; ^{1}H NMR (400 MHz, $CDCl_3$) δ 3.84 (d, J = 13.0 Hz, 1H, NCH), 3.67 (s, 3H, OMe), 3.49-3.38 (m, 1H, NCH), 3.28-3.18 (m, 1H, NCH), 3.11 (d, J = 13.0 Hz, 1H, NCH), 2.06-1.97 (m, 1H, CH), 1.62-1.52 (m, 2H, CH), 1.46-1.40 (m, 1H, CH), 1.44 (s, 9H, CMe_3), 1.15 (s, 3H, Me); ^{13}C NMR (100.6 MHz, $CDCl_3$) δ 176.3 (C=O, CO_2Me), 154.7 (C=O, Boc), 79.3 (O*C*Me_3), 51.8 (OMe), 42.6 (NCH_2), 33.6 (CH_2), 28.4

(C*Me*$_3$), 22.1 (Me), 21.8 (CH_2) (NCH_2 resonance not resolved); HMRS (ESI) *m/z* calcd for $C_{13}H_{23}NO_4$ $(M + Na)^+$ 280.1519, found 280.1512 (+2.7 ppm error).
Lab Book Reference: mcw/3/72/1

***tert*-Butyl 3-(hydroxymethyl)-3-methylpiperidine-1-carboxylate S2**

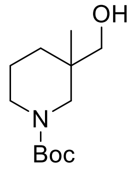

S2

Using general procedure F, methyl ester **4d** (400 mg, 1.55 mmol, 1.0 eq.) in THF (5 mL) and $LiAlH_4$ (59 mg, 1.55 mmol, 1.0 eq.) in THF (25 mL) gave the crude product. Purification by flash chromatography on silica with 50:50 hexane-Et_2O as eluent gave alcohol **S2** (323 mg, 91%) as a colourless oil, R_F (50:50 Et_2O-hexane) 0.3; IR (ATR) 3446 (OH), 2933, 2862, 1663 (C=O), 1426, 1364, 1245, 1156, 766 cm^{-1}; ^{1}H NMR (400 MHz, $CDCl_3$) δ 3.83 (d, J = 11.5 Hz, 1H, HOC*H*), 3.53 (dd, J = 11.5, 3.0 Hz, 1H, HOC*H*), 3.46-2.80 (m, 3H, NCH), 2.51 (d, J = 12.5 Hz, 1H, NCH), 1.69 (br s, 1H, CH), 1.57-1.39 (m, 2H, CH), 1.46 (s, 9H, CMe_3), 1.38-1.28 (m, 1H, CH), 0.91 (s, 3H, Me); ^{13}C NMR (100.6 MHz, $CDCl_3$) δ 156.2 (C=O), 79.8 (O*C*Me_3), 66.0 (OCH_2), 50.2 (NCH_2), 45.4 (NCH_2), 35.9 (*C*Me), 33.5 (CH_2), 28.4 (C*Me*$_3$), 23.2 (Me), 21.6 (CH_2); HMRS (ESI) *m/z* calcd for $C_{12}H_{23}NO_3$ $(M + Na)^+$ 252.1570, found 252.1561 (+3.8 ppm error).
Lab Book Reference: mcw/4/35

3-(Hydroxymethyl)-3-methylpiperidine hydrochloride 2c

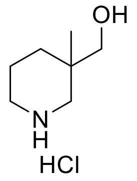

2c

HCl (2.0 M solution in Et_2O, 1.74 mL, 3.49 mmol, 8.0 eq.) was added to a stirred solutiuon of *N*-Boc amino alcohol **S2** (100 mg, 0.44 mmol, 1.0 eq.) in Et_2O (1 mL) at rt under Ar. The resulting solution was stirred at rt for 18 h. The solvent was then evaporated under reduced pressure to give piperidine **2c**·HCl (72 mg, 100%) as a thick colourless oil, IR (ATR) 3362 (OH or NH), 2943, 2805, 2729, 1595, 1451, 1027, 519 cm^{-1}; ^{1}H NMR (400 MHz, DMSO-d_6) δ 8.51 (br s, 2H, NH_2), 4.86 (br s, 1H, OH), 3.25 (d, J = 10.5 Hz, 1H, HOC*H*), 3.20 (d, J = 10.5 Hz, 1H, HOC*H*), 2.98 (ddd, J = 12.5, 5.0, 5.0 Hz, 1H, NCH),

2.88-2.80 (m, 2H, NCH), 2.75 (d, J = 12.5 Hz, 1H, NCH), 1.69-1.62 (m, 2H, CH), 1.55-1.47 (m, 1H, CH), 1.51 (ddd, J = 12.5, 5.0, 5.0, 1H, CH), 0.92 (s, 3H, Me); ^{13}C NMR (100.6 MHz, DMSO-d_6) δ 67.2 (OCH_2), 49.2 (NCH_2), 43.3 (NCH_2), 33.9 (*C*Me), 29.8 (CH_2), 20.8 (Me), 18.2 (CH_2); HMRS (ESI) *m/z* calcd for $C_7H_{16}NO$ M^+ 130.1226, found 130.1231 (–3.5 ppm error).
Lab Book Reference: mcw/5/11

(1,3-Dimethylpiperidin-3-yl)methanol 2d

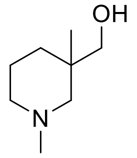

2d

Using general procedure G, *N*-Boc piperidine **S2** (257 mg, 1.00 mmol, 1.0 eq.) in THF (20 mL) and $LiAlH_4$ (190 mg, 5.00 mmol, 5.0 eq.) in THF (30 mL) gave *N*-methyl piperidine **2d** (138 mg, 96%) as a colourless oil, IR (ATR) 3337 (OH), 2934, 2777, 1447, 1211, 1054, 811 cm^{-1}; ^{1}H NMR (400 MHz, $CDCl_3$) δ 3.62 (dd, J = 10.5, 1.5 Hz, 1H, HOC*H*), 3.56 (dd, J = 10.5, 1.5 Hz, 1H, HOC*H*), 2.67 (br s, 2H, NCH), 2.20 (s, 3H, NMe), 2.11-2.01 (m, 1H, CH), 1.96 (br d, J = 10.5 Hz, 2H, NCH), 1.65-1.54 (m, 2H, CH), 1.26-1.14 (m, 1H, CH), 0.77 (s, 3H, Me); ^{13}C NMR (100.6 MHz, $CDCl_3$) δ 74.3 (OCH_2), 62.8 (NCH_2), 55.9 (NCH_2), 46.4 (Me), 34.9 (CH_2), 34.4 (*C*Me), 29.9 (CH_2), 23.1 (Me); HMRS (ESI) *m/z* calcd for $C_8H_{17}NO$ $(M + H)^+$ 144.1383, found 144.1379 (+2.7 ppm error).
Lab Book Reference: mcw/3/80

1-*tert*-Butyl 4-methyl piperidine-1,4-dicarboxylate 3e

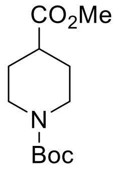

3e

Using general procedure A, thionyl chloride (3.09 mL, 42.6 mmol, 1.1 eq.) and isonipecotic acid (5.00 g, 38.7 mmol, 1.0 eq.) in MeOH (50 mL) and then Et_3N (5.40 mL, 38.7 mmol, 1.0 eq.) in CH_2Cl_2 (25 mL) and Boc_2O (8.45g, 38.7 mmol, 1.0 eq.) in CH_2Cl_2 (25 mL) gave the crude product. Purification by flash chromatography on silica with 80:20 hexane-Et_2O as eluent gave methyl ester **3e** (7.98 g, 85%) as a colourless oil, R_F (80:20 hexaneEt_2O) 0.1; IR (ATR) 2953, 2860, 1734 (C=O, CO_2Me), 1689 (C=O, Boc), 1448, 1419, 1365, 1158, 1038, 768 cm^{-1}; ^{1}H NMR (400 MHz, $CDCl_3$) δ 4.01 (br d, J = 13.5 Hz,

2H, NCH), 3.68 (s, 3H, OMe), 2.81 (ddd, *J* = 13.5, 11.5, 3.0 Hz, 2H, NCH), 2.44 (tt, *J* = 11.0, 4.0 Hz, 1H, C*H*CO_2Me), 1.91-1.81 (m, 2H, CH), 1.67-1.55 (m, 2H, CH), 1.45 (s, 9H, CMe_3); ^{13}C NMR (100.6 MHz, $CDCl_3$) δ 175.0 (C=O, CO_2Me), 154.7 (C=O, Boc), 79.6 (O*C*Me_3), 51.8 (OMe), 43.1 (NCH_2), 41.0 (CH), 28.4 (C*Me₃*), 27.9 (CH_2); HMRS (ESI) *m/z* calcd for $C_{12}H_{21}NO_4$ (M + Na)$^+$ 266.1363, found 266.1355 (+2.6 ppm error).

Lab Book Reference: mcw/3/3/2

1-*tert*-Butyl 4-methyl 4-methylpiperidine-1,4-dicarboxylate 4e

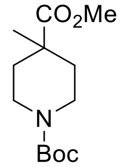

4e

Using general procedure E, potassium bis(trimethylsilyl)amide (15 mL of a 0.5 M solution in toluene, 7.50 mmol, 1.5 eq.) and methyl ester **3e** (1.22 g, 5.00 mmol, 1.0 eq.) in THF (10 mL) and then methyl iodide (0.47 mL, 7.50 mmol, 1.5 eq.) gave the crude product. Purification by flash chromatography on silica with 80:20 hexane-Et_2O as eluent gave methylated piperidine ester **4e** (1.29 g, 100%) as a colourless oil, R_F (80:20 hexane-Et_2O) 0.3; IR (ATR) 2971, 2872, 1729 (C=O, CO_2Me), 1691 (C=O, Boc), 1459, 1419, 1169, 1147, 1110, 872 cm^{-1}; ^{1}H NMR (400 MHz, $CDCl_3$) δ 3.79-3.71 (m, 2H, NCH), 3.70 (s, 3H, OMe), 2.98 (br dd, *J* = 11.0, 11.0 Hz, 2H, NCH), 2.09-2.02 (m, 2H, CH), 1.45 (s, 9H, CMe_3), 1.35 (ddd, *J* = 14.5, 11.0, 4.5 Hz, 2H, CH), 1.20 (s, 3H, Me); ^{13}C NMR (100.6 MHz, $CDCl_3$) δ 177.0 (C=O, CO_2Me), 154.9 (C=O, Boc), 79.4 (O*C*Me_3), 51.9 (OMe), 41.7 (*C*Me), 41.2 (NCH_2), 34.6 (CH_2), 28.4 (C*Me₃*), 25.8 (Me); HMRS (ESI) *m/z* calcd for $C_{13}H_{23}NO_4$ (M + Na)$^+$ 280.1519, found 280.1510 (+3.1 ppm error).

Lab Book Reference: mcw/3/5/1

***tert*-Butyl 4-(hydroxymethyl)-4-methylpiperidine-1-carboxylate S3**

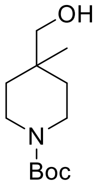

S3

Using general procedure F, methyl ester **4e** (200 mg, 0.78 mmol, 1.0 eq.) in THF (5 mL) and $LiAlH_4$ (29.5 mg, 0.78 mmol, 1.0 eq.) in THF (30 mL) gave the crude product. Purification by flash chromatography on silica with 50:50 hexane-Et_2O as eluent gave amino alcohol **S3** (168 mg, 94%) as a colourless oil, R_F (50:50 Et_2O-hexane) 0.3; IR (ATR) 3435 (OH), 2972, 2817, 1666 (C=O), 1423, 1247, 1158, 1047, 860, 735 cm^{-1}; ^1H NMR (400 MHz, $CDCl_3$) δ 3.67 (br d, *J* = 10.5 Hz, 2H, NCH), 3.37 (s, 2H, HOC*H₂*), 3.12 (ddd, *J* = 13.5, 10.5, 3.0 Hz, 2H, NCH), 1.53-1.40 (m, 11H, CH and CMe_3), 1.32-1.23 (m, 2H, CH), 0.98 (s, 3H, Me); ^{13}C NMR (100.6 MHz, $CDCl_3$) δ 154.9 (C=O), 79.3 (O*C*Me_3), 71.7 (OCH_2), 39.9 (NCH_2), 33.8 (*C*Me), 33.1 (CH_2), 28.4 (C$\mathit{Me_3}$), 20.5 (Me); HMRS (ESI) *m/z* calcd for $C_{12}H_{23}NO_3$ (M + Na)$^+$ 252.1570, found 252.1567 (+1.1 ppm error).
Lab Book Reference: mcw/3/73/1

4-(Hydroxymethyl)-4-methylpiperidine hydrochloride 2e

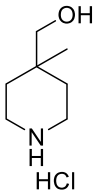

2e

HCl (2.0 M solution in Et_2O, 1.74 mL, 3.49 mmol, 8.0 eq.) was added to a stirred solutiuon of *N*-Boc amino alcohol **S3** (100 mg, 0.44 mmol, 1.0 eq.) in Et_2O (1 mL) at rt under Ar. The resulting solution was stirred at rt for 18 h. The solvent was then evaporated under reduced pressure to give piperidine **2e**·HCl (72 mg, 100%) as a white solid, mp 120-122 °C; IR (ATR) 3193 (OH or NH), 2984, 2744, 2665, 1610, 1468, 1027, 701, 611 cm^{-1}; ^1H NMR (400 MHz, DMSO-*d₆*) δ 8.50 (s, 2H, NH_2), 3.17 (d, *J* = 5.0 Hz, 2H, HOC$\mathit{H_2}$), 3.06 (ddd, *J* = 12.5, 4.0, 4.0 Hz, 2H, NCH), 2.96 (ddd, *J* = 12.5, 10.0, 4.0 Hz, 2H, NCH), 1.62 (ddd, *J* = 14.5, 10.0, 4.0 Hz, 2H, CH), 1.38 (br s, 1H, OH), 1.34 (ddd, *J* = 14.5, 4.0, 4.0 Hz, 2H, CH); ^{13}C NMR (100.6 MHz, DMSO-d_6) δ 68.9 (OCH_2), 32.6 (NCH_2), 29.8 (CH_2), 28.1 (*C*Me), 21.2 (Me); HMRS (ESI) *m/z* calcd for $C_7H_{16}NO$ M^+ 130.1226, found 130.1228 (–1.5 ppm error).
Lab Book Reference: mcw/5/12

Methyl 3-methylpyridine-4-carboxylate 5c

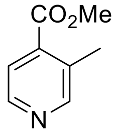

5c

Using general procedure H, thionyl chloride (175 µL, 2.41 mmol, 1.1 eq.) and 3-methyl-isonicotinic acid (300 mg, 2.19 mmol, 1.0 eq.) in MeOH (5 mL) gave **5c** (326 mg, 99%) as a colourless oil, R_F (100:9:1 CH_2Cl_2-MeOH-$NH_4OH_{(aq)}$) 0.7; ^{1}H NMR (400 MHz, $CDCl_3$) δ 8.56 (s, 1H, Ar), 8.54 (d, J = 6.0 Hz, 1H, Ar), 7.67 (d, J = 6.0 Hz, 1H, Ar), 3.92 (s, 3H, OMe), 2.56 (s, 3H, Me); ^{13}C NMR (100.6 MHz, $CDCl_3$) δ 166.7 (C=O), 153.0 (Ar), 147.0 (Ar), 136.5 (*ipso*-Ar), 133.6 (*ipso*-Ar), 123.2 (Ar), 52.5 (OMe), 18.3 (Me). Spectroscopic data consistent with those reported in the literature.[7]

Lab book reference MA 5-19

Methyl (3*R,4*R**)-3-methylpiperidine-4-carboxylate 6c**

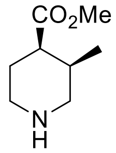

6c

Using general procedure I, PtO_2 (100 mg, 0.53 mmol, 25 mol%) and pyridine ester **5c** (326 mg, 1.99 mmol, 1.0 eq.) in glacial acetic acid (3 mL) gave the crude product which contained an 85:15 mixture (by ^{1}H NMR spectroscopy) of **6c** and its *trans* diastereomer. Purification by flash column chromatography on silica with 200:9:1 CH_2Cl_2-MeOH-$NH_4OH_{(aq)}$ as eluent gave **6c** (297 mg, 88%) as a colourless oil, R_F (100:9:1 CH_2Cl_2-MeOH-$NH_4OH_{(aq)}$) 0.3; ^{1}H NMR (400 MHz, $CDCl_3$) δ 3.68 (s, 3H, OMe), 3.09 (ddd, J = 12.0, 4.5, 4.5 Hz, 1H, CH), 2.88 (dd, J = 12.0, 4.0 H, 1H, NCH), 2.82 (dd, J = 12.0, 3.0 Hz, 1H, NCH), 2.66-2.59 (m, 2H, NCH), 2.17-2.08 (m, 1H, CH), 1.86-1.75 (m, 1H, CH), 1.66-1.60 (m, 1H, CH), 0.95 (d, J = 7.0 H, 3H, CH*Me*); ^{13}C NMR (100.6 MHz, $CDCl_3$) δ 175.0 (C=O), 51.7 (NCH_2), 51.5 (OMe), 45.2 (NCH_2), 44.5 (CH), 31.2 (CH), 24.4 (CH_2), 13.8 (Me). Spectroscopic data consistent with those reported in the literature.[8]

Lab book reference MA 5-21

[(3*R,4*R**)-3-Methylpiperidin-4-yl]methanol hydrochloride 2f**

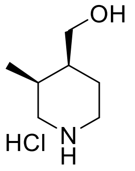

2f

Using general procedure F, $LiAlH_4$ (138 mg, 3.63 mmol, 3.0 eq.) and piperidine ester **6c** (190 mg, 1.21 mmol, 1.0 eq.) in THF (12 mL) gave the crude alcohol as a colourless oil, R_F (100:9:1 CH_2Cl_2-MeOH-$NH_4OH_{(aq)}$) 0.1. Then, HCl (1.0 mL of a 2 M solution in Et_2O, 2.0 mmol) was added and the solvent was evaporated under reduced pressure to give **2f**·HCl (155 mg, 77%) as a white solid, IR (ATR) 3358 (NH_2 or OH), 2961, 1466 cm^{-1}; ^1H NMR (400 MHz, MeOH-*d*$_4$) δ 3.45 (d, J = 6.5 Hz, 2H, OCH_2), 3.09 (dd, J = 12.5, 5.0 Hz, 1H, NCH), 3.03 (dd, J = 12.5, 4.0 Hz, 1H,NCH), 2.96 (dd, J = 9.5, 4.5 Hz, 1H, NCH), 2.93 (dd, J = 9.5, 4.5 Hz, 1H, NCH), 2.23-2.21 (m, 1H, CH), 1.91-1.81 (m, 1H, CH), 1.73-1.58 (m, 2H, CH), 0.96 (d, J = 7.5 Hz, 3H, CH*Me*); ^{13}C NMR (100.6 MHz, MeOH-*d*$_4$) δ 63.3 (OCH_2), 50.5 (NCH_2), 44.3 (NCH_2), 39.6 (CH), 29.4 (CH), 22.7 (CH_2), 12.5 (Me); MS (ESI) *m/z* 130 [$(M + H)^+$]; HRMS (ESI) *m/z* calcd for $C_7H_{16}NO$ M^+ 130.1226, found 130.1230 (−2.1 ppm error).
Lab book reference MA 5-27

[(3*R,4*R**)-1,3-Dimethylpiperidin-4-yl]methanol 2g**

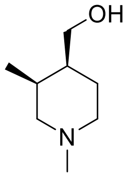

2g

Using general procedure B, $NaBH(OAc)_3$ (318 mg, 1.5 mmol, 1.5 eq.), free base of piperidine **2f** (129 mg, 1.0 mmol, 1.0 eq.), 37% $HCHO_{(aq)}$ (743 μL, 10 mmol, 10 eq.) and $MgSO_4$ (1.0 g) in 4:1 CH_2Cl_2-AcOH (10 mL) gave the crude product. Purification by flash column chromatography on silica with 400:9:1 CH_2Cl_2-MeOH-$NH_4OH_{(aq)}$ as eluent gave **2g** (127 mg, 89%) as a colourless oil, R_F (100:9:1 CH_2Cl_2-MeOH-$NH_4OH_{(aq)}$) 0.2; IR (ATR) 3335 (OH), 2925, 1462, 1445 cm^{-1}; ^1H NMR (400 MHz, $CDCl_3$) δ 3.57 (dd, J = 10.5, 7.0 Hz, 1H, OCH), 3.52 (dd, J = 10.5, 7.0 Hz, 1H, OCH), 2.75 (br d, J = 9.0 Hz, 1H, NCH), 2.54 (br d, J = 9.0 Hz, 1H, NCH), 2.22 (s, 3H, NMe), 2.13 (br d, J = 11.0 Hz, 1H, NCH), 1.71-1.61 (m, 1H, NCH), 1.57-1.49 (m, 2H, CH), 0.97 (d, J = 7.0 Hz, 3H, CH*Me*); ^{13}C NMR (100.6

MHz, $CDCl_3$) δ 74.9 (CH_2), 64.7 (CH_2), 62.6 (CH_2), 46.9 (OMe), 40.4 (CH), 30.1 (CH), 24.4 (CH_2), 13.4 (Me); HRMS (ESI) *m/z* calcd for $C_8H_{17}NO$ $(M + H)^+$ 144.1383, found 144.1389 (−3.6 ppm error).
Lab book reference MA 6-20

Methyl (3*R,4*S**)-4-methylpiperidine-3-carboxylate S4 and methyl (3*S**,4*R**)-4-methylpiperidine-3-carboxylate 6d**

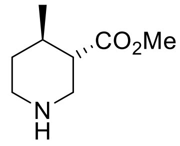

S4

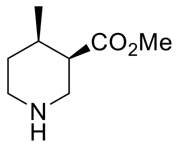

6d

Using general procedure I, PtO_2 (120 mg, 0.636 mmol, 30 mol%) and methyl 4-methylnicotinate (300 mg, 1.99 mmol, 1.0 eq.) in glacial acetic acid (3 mL) gave the crude product which contained a 70:30 mixture (by ^{1}H NMR spectroscopy) of **6d** and **S4**. Purification by flash column chromatography on silica with 200:9:1 CH_2Cl_2-MeOH-$NH_4OH_{(aq)}$ as eluent gave piperidine **S4** (96 mg, 31%) as a yellow oil, R_F (100:9:1 CH_2Cl_2-MeOH-$NH_4OH_{(aq)}$) 0.40; IR (ATR) 2951, 1726 (C=O), 1434 cm^{-1}; ^{1}H NMR (400 MHz, $CDCl_3$) δ 3.66 (s, 3H, OMe), 3.17 (ddd, 1H, J = 12.5, 3.5, 1.0 Hz, NCH), 3.07-3.00 (m, 1H, NCH), 2.67 (dd, 1H, J = 12.0, 10.5 Hz, NCH), 2.61 (ddd, 1H, J = 12.0, 12.0, 3.0, 3.0 Hz, NCH), 2.09 (ddd, 1H, J = 11.0, 11.0, 3.5 Hz, CH), 1.92 (s, 1H, NH), 1.83-1.71 (m, 1H, CH), 1.70-1.64 (m, 1H, CH), 1.10 (ddd, 1H, J = 25.0, 12.0, 4.5 Hz, CH), 0.91 (d, 3H, J = 6.5 Hz, Me); ^{13}C NMR (100.6 MHz, $CDCl_3$) □ 174.8 (C=O), 51.5 (OMe), 49.1 (CH_2), 46.3 (CH_2), 34.3 (CH_2), 33.3 (CH), 20.6 (Me); HRMS (ESI) *m/z* calcd for $C_8H_{16}NO_2$ $(M + H)^+$ 158.1176, found 158.1173 (+1.5 ppm error) and piperidine **6d** (142 mg, 45%) as a colourless oil, R_F (100:9:1 CH_2Cl_2-MeOH-$NH_4OH_{(aq)}$) 0.4; IR (ATR) 2923, 1722 (C=O), 1435 cm^{-1}; ^{1}H NMR (400 MHz, $CDCl_3$) δ 3.65 (s, 3H, OMe), 3.12 (dd, J = 13.0, 5.0 Hz, 1H, NCH), 3.00 (ddd, J = 13.0, 5.0, 5.0 Hz, 1H, NCH), 2.82 (dd, J = 13.0, 3.5 Hz, 1H, NCH), 2.62 (ddd, J = 13.0, 7.5, 4.0 Hz, 1H, NCH), 2.52 (dd, J = 9.0, 5.0 Hz, 1H, CH), 2.23 (s, 1H, NH), 2.03-1.94 (m, 1H, CH), 1.59-1.45 (m, 2H, CH), 0.94 (d, J = 7.0 Hz, 3H, CH*Me*); ^{13}C NMR (100.6 MHz, $CDCl_3$) δ 174.6 (C=O), 51.2 (OMe), 46.6 (NCH_2), 45.0 (CH), 44.6 (NCH_2), 31.5 (CH), 31.2 (CH_2), 18.1 (Me); HRMS (ESI) *m/z* calcd for $C_8H_{15}NO_2$ $(M + H)^+$ 158.1176, found 158.1176 (−0.5 ppm error).
Lab book reference MA 5-38

[(3*R,4*R**)-4-Methylpiperidin-3-yl]methanol hydrochloride 2h**

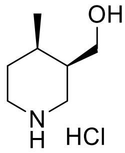

2h

Using general procedure F, $LiAlH_4$ (103 mg, 2.71 mmol, 3.0 eq.) and piperidine ester **6d** (142 mg, 0.90 mmol, 1.0 eq.) in THF (9 mL) gave the crude alcohol. Then, HCl (450 µL of a 2 M solution in Et_2O, 0.94 mmol) was added and the solvent was evaporated under reduced pressure to give **2h**·HCl (132 mg, 88%) as a colourless oil, R_F (100:9:1 CH_2Cl_2-MeOH-$NH_4OH_{(aq)}$) 0.4; IR (ATR) 3352 (NH_2 or OH), 2962, 1453 cm^{-1}; ^1H NMR (400 MHz, MeOH-*d₄*) δ 3.56-3.53 (m, 2H, OCH), 3.15-2.97 (m, 4H, NCH), 2.05-1.98 (m, 1H, CH), 1.95-1.88 (m, 1H, CH), 1.80-1.63 (m, 2H, CH), 0.93 (d, J = 6.5 Hz, 3H, CH*Me*); ^{13}C NMR (100.6 MHz, MeOH-d_4) δ 62.0 (OCH_2), 45.1 (NCH_2), 42.9 (NCH_2), 39.7 (CH), 29.7 (CH_2), 29.5 (CH), 14.9 (Me); HRMS (ESI) *m/z* calcd for $C_7H_{16}NO$ M^+ 130.1226, found 130.1231 (−3.0 ppm error).
Lab book reference MA 5-45

Methyl (2*R,3*S**)-3-methylpiperidine-2-carboxylate 6b and methyl (2*R**,3*R**)-3-methylpiperidine-2-carboxylate S5**

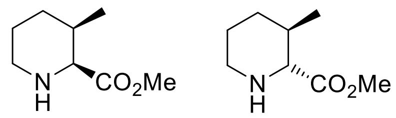

6b **S5**

Using general procedure I, PtO_2 (22 mg, 0.10 mmol, 10 mol%) and methyl-3-methylpicolinate (0.13 mL, 0.99 mmol, 1.0 eq.) in AcOH (1.5 mL) gave the crude product which contained an 85:15 mixture (by ^1H NMR spectroscopy) of piperidine ester **6b** and **S5** (1.85 g, 78%) as a colourless oil, IR (ATR) 2929, 1741 (C=O), 1435, 1202 1005, 758 cm^{-1}; ^1H NMR (400 MHz, $CDCl_3$) δ 3.72 (s, 0.45H, OMe), 3.71 (s, 2.55H, OMe), 3.52 (d, J = 3.5 Hz, 0.85H, $NCHCO_2$), 3.16-3.08 (m, 1H, NCH), 2.96 (d, J = 10.0 Hz, 0.15H, $NCHCO_2$), 2.65-2.57 (m, 1H, NCH), 2.24-2.13 (m, 1H, C*H*Me), 1.68-1.57 (m, 3H, CH), 1.39-1.32 (m, 1H, CH), 0.93 (d, J = 7.0 Hz, 2.55H, CH*Me*), 0.87 (d, J = 7.0 Hz, 0.45H, CH*Me*); ^{13}C NMR (100.6 MHz, $CDCl_3$) for **S5**: δ 173.6 (C=O), 62.2 (N*C*HCO₂), 51.8 (OMe), 46.1 (NCH_2), 30.9 (CH_2), 30.6 (*C*HMe), 21.2 (CH_2), 13.6 (CH*Me*); for **6b**: 173.6 (C=O), 66.5 (NCH*C*O₂), 45.9 (CH_2), 34.9 (CH), 33.1 (CH_2), 26.7 (CH_2), 19.0 (CH*Me*); MS (ESI) *m/z* 158 $(M + H)^+$; HRMS (ESI) *m/z* calcd for $C_8H_{15}NO_2$ $(M + H)^+$ 158.1176, found 158.1177 (–1.3 ppm error).
Lab book reference PJ-04-37.

Methyl (2*R,3*S**)-1-benzyl-3-methylpiperidine-2-carboxylate S6 and methyl (2*R**,3*R**)-1-benzyl-3-methylpiperidine-2-carboxylate S7**

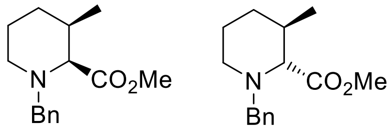

S6 **S7**

PhCHO (0.36 mL, 3.5 mmol, 1.1 eq.) was added dropwise to a stirred solution of an 85:15 mixture of piperidines **S6** and **S7** (500 mg, 3.18 mmol, 1.0 eq.), $NaBH(OAc)_3$ (1.35 g, 6.37 mmol, 2.0 eq.) and AcOH (0.03 mL, 0.64 mmol, 0.2 eq.) in DCE (30 mL) at rt under Ar. The resulting mixture was stirred at rt for 18 h. The reaction mixture was poured into saturated $NaHCO_{3(aq)}$ (100 mL) and extracted with CH_2Cl_2 (3 × 50 mL). The combined organics were washed with brine (100 mL), dried ($MgSO_4$) and evaporated under reduced pressure to give the crude product as a green oil which contained a 90:10 mixture (by ^{1}H NMR spectroscopy) of *N*-benzyl piperidine ester **S6** and **S7**. Purification by flash column chromatography on silica with 98:2 hexane-EtOAc as eluent gave *N*-benzyl piperidine ester **S6** (626 mg, 80%) as a yellow oil, R_F (95:5 hexane-EtOAc) 0.23; IR (ATR) 2928, 1728 (C=O), 1453, 1148, 697 cm^{-1}; ^{1}H NMR (400 MHz, $CDCl_3$) δ 7.32-7.29 (m, 4H, Ph), 7.25-7.22 (m, 1H, Ph), 3.69 (s, 3H, OMe), 3.61 (d, J = 13.5 Hz, 1H, NCHPh), 3.56 (d, J = 13.5 Hz, 1H, NCHPh), 3.45 (d, J = 5.0 Hz, 1H, $NCHCO_2$), 3.00-2.92 (m, 1H, NCH), 2.54-2.45 (m, 1H, NCH), 2.03-1.93 (m, 1H, C*H*Me), 1.71-1.64 (m, 1H, CH), 1.60-1.46 (m, 3H, CH), 0.90 (d, J = 7.0 Hz, 3H, CH*Me*); ^{13}C NMR (100.6 MHz, $CDCl_3$) δ 173.0 (C=O), 139.1 (*ipso*-Ph), 128.9 (Ph), 128.3 (Ph), 127.1 (Ph), 66.2 (N*C*HCO$_2$), 60.2 (NCH_2Ph), 50.5 (OMe), 47.0 (NCH_2), 33.2 (*C*HMe), 27.8 (CH_2), 25.2 (CH_2), 18.2 (CH*Me*); HRMS (ESI) *m/z* calcd for $C_{15}H_{21}NO_2$ (M + H)$^+$ 248.1645, found 248.1643 (–0.7 ppm error) and *N*-benzyl piperidine ester **S7** (82 mg, 10%) as a yellow oil, R_F (95:5 hexane-EtOAc) 0.1; IR (ATR) 2928, 1727 (C=O), 1453, 1147, 734, 697 cm^{-1}; ^{1}H NMR (400 MHz, $CDCl_3$) δ 7.32-7.29 (m, 4H, Ph), 7.26-7.22 (m, 1H, Ph), 3.76 (s, 3H, OMe), 3.71 (d, J = 13.5 Hz, 1H, NCHPh), 3.25 (d, J = 13.5 Hz, 1H, NCHPh), 2.87 (ddd, J = 11.0, 5.5, 5.5 Hz, 1H, NCH), 2.64 (d, J = 9.0 Hz, 1H, $NCHCO_2$), 1.95-1.86 (m, 2H, CH), 1.76-1.68 (m, 1H, CH), 1.61-1.53 (m, 2H, CH), 1.05-0.94 (m, 1H, CH), 0.90 (d, J = 7.0 Hz, 3H, CH*Me*); ^{13}C NMR (100.6 MHz, $CDCl_3$) δ 174.3 (C=O), 137.7 (*ipso*-Ph), 129.6 (Ph), 128.3 (Ph), 127.2 (Ph), 73.7 (N*C*HCO$_2$), 61.2 (NCH_2Ph), 51.8 (OMe), 51.3 (NCH_2), 34.4 (*C*HMe), 32.0 (CH_2), 24.6 (CH_2), 18.9 (CH*Me*); HRMS (ESI) *m/z* calcd for $C_{15}H_{21}NO_2$ (M + H)$^+$ 248.1645, found 248.1641 (+1.3 ppm error). Spectroscopic data consistent with those reported in the literature.[9]

Lab Book – PJ-02-45.

N-Benzyl piperidine **S6** (173 mg, 0.70 mmol, 1.0 eq.) was added to a flask containing dry THF (5 mL) at rt under Ar. The reaction flask was evacuated under reduced pressure and back-filled with Ar three times and the solution was cooled to –78 °C. KO*t*Bu (0.84 mL of a 1 M solution in THF, 0.84 mmol, 1.2 eq.) was added dropwise. The resulting solution was stirred at –78 °C for 2 h. Then, water (1 mL) was added at –78 °C and the reaction mixture was allowed to warm to rt. The reaction mixture was then evaporated under reduced pressure to give an orange oil. The solution was taken up into water (2 mL) and extracted with EtOAc (4 × 10 mL). The combined organics were dried ($MgSO_4$) and evaporated under reduced pressure to give a 70:30 mixture (by ^{1}H NMR spectroscopy) of *N*-benzyl piperidine ester **S7** and **S6**. Purification by flash column chromatography on silica with 95:5 hexane-EtOAc as eluent gave *N*-benzyl piperidine ester **S6** (40 mg, 23%) as a colourless oil and *N*-benzyl piperidine ester **S7** (104 mg, 60%) as a colourless oil.

Lab Book – PJ-06-08.

[(2*R,3*R**)-1-Benzyl-3-methylpiperidin-2-yl]methanol S8**

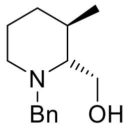

S8

Using general procedure F, $LiAlH_4$ (114 mg, 1.5 mmol, 2.0 eq.) and piperidine ester **S7** (370 mg, 1.5 mmol, 1.0 eq.) in THF (25 mL) gave piperidine alcohol **S8** (330 mg, 100%) as a colourless oil, IR (ATR) 3401 (OH), 2926, 1452, 1061, 697 cm^{-1}; ^{1}H NMR (400 MHz, $CDCl_3$) δ 7.33-7.21 (m, 5H, Ph), 4.09 (d, *J* = 13.5 Hz, 1H, NCHPh), 3.94 (dd, *J* = 11.5, 3.5 Hz, 1H, OCH), 3.69 (dd, *J* = 11.5, 2.0 Hz, 1H, OCH), 3.23 (d, *J* = 13.5 Hz, 1H, NCHPh), 2.91 (br s, 1H, OH), 2.86 (dddd, *J* = 12.0, 4.0, 4.0, 2.0 Hz, 1H, NCH), 2.11 (ddd, *J* = 11.5, 11.5, 4.0 Hz, 1H, NCH), 2.05 (ddd, *J* = 9.0, 3.0, 3.0 Hz, 1H, NCH), 1.85-1.70 (m, 2H, CH), 1.52-1.45 (m, 2H, CH), 1.11-1.00 (m, 1H, CH), 0.97 (d, *J* = 6.5 Hz, 3H, CH*Me*); ^{13}C NMR (100.6 MHz, $CDCl_3$) δ 139.1 (*ipso*-Ph), 128.9 (Ph), 128.5 (Ph), 127.1 (Ph), 68.2 (NCH), 58.8 (OCH_2), 57.1 (NC*H*$_2$Ph), 52.0 (NCH_2), 33.2 (CH_2), 30.5 (*C*HMe), 24.1 (CH_2), 20.0 (CH*Me*); HRMS (ESI) *m/z* calcd for $C_{14}H_{21}NO$ (M + H)$^+$ 220.1696, found 220.1691 (+2.5 ppm error).

Lab Book – PJ-03-79.

[(2*R,3*R**)-3-Methylpiperidin-2-yl]methanol S9**

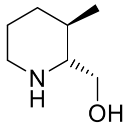

S9

20% $Pd(OH)_2/C$ (124 mg, 0.18 mmol, 10 mol%) was added to a stirred solution of piperidine **S8** (389 mg, 1.78 mmol, 1.0 eq.) and $NH_4^+HCO_2^-$ (2.24 g, 35.6 mmol, 20.0 eq.) in EtOH (35 mL) at rt under Ar. The resulting suspension was stirred and heated at reflux for 16 h. After being allowed to cool to rt, the solids were removed by filtration through Celite. The filtrate was evaporated under reduced pressure to give piperidine alcohol **S9** (172 mg, 73%) as an orange oil, IR (ATR) 3314 (OH or NH), 2924, 1455, 1055, 829, 578 cm^{-1}; ^{1}H NMR (400 MHz, $CDCl_3$) δ 3.75 (dd, J = 11.0, 3.0 Hz, 1H, OCH), 3.44 (dd, J = 11.0, 8.0 Hz, 1H, OCH), 3.07 (dddd, J = 12.0, 4.0, 2.0, 2.0 Hz, 1H, NCH), 2.87 (br s, 2H, OH and NH), 2.56 (ddd, J = 12.0, 12.0, 3.0 Hz, 1H, NCH), 2.25 (ddd, J = 10.0, 8.0, 3.0 Hz, 1H, NHC*H*), 1.77-1.68 (m, 1H, CH), 1.66-1.58 (m, 1H, CH), 1.52-1.37 (m, 1H, CH), 1.37-1.25 (m, 1H, C*H*Me), 1.12-1.00 (m, 1H, CH), 0.85 (d, J = 7.0 Hz, 3H, CH*Me*); ^{13}C NMR (100.6 MHz, $CDCl_3$) δ 64.1 (NCH), 63.9 (OCH_2), 46.5 (NCH_2), 33.9 (CH_2), 33.2 (*C*HMe), 26.7 (CH_2), 18.6 (CH*Me*); HRMS (ESI) *m/z* calcd for $C_7H_{15}NO$ (M + H)$^+$ 130.1226, found 130.1233 (–4.9 ppm error).
Lab Book – PJ-03-06.

1-[(2*R,3*R**)-2-(Hydroxymethyl)-3-methylpiperidin-1-yl]ethan-1-one 2i**

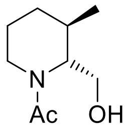

2i

Ac_2O (0.48 mL, 5.12 mmol, 6.0 eq.) was added dropwise to a stirred solution of piperidine alcohol **S9** (100 mg, 0.85 mmol, 1.0 eq.) and DMAP (95 mg, 0.85 mmol, 1.0 eq.) in pyridine (8.5 mL) at rt under Ar. The resulting solution was stirred at rt for 16 h. The reaction mixture was evaporated under reduced pressure to give an orange oil. The crude product was dissolved in CH_2Cl_2 (50 mL) and washed with 15% $CuSO_{4(aq)}$ (3 × 40 mL). The combined organics were washed with saturated $EDTA_{(aq)}$ (2 × 50 mL), dried ($MgSO_4$) and evaporated under reduced pressure to give the crude product as a yellow oil. K_2CO_3 (352 mg, 2.60 mmol, 3.0 eq.) was added to the crude product in MeOH (8.5 mL). The resulting solution was stirred at rt for 16 h. The solution was poured into water (100 mL) and the two layers were separated. The aqueous layer was extracted with CH_2Cl_2 (7 × 30 mL) and the combined organics were dried ($MgSO_4$) and evaporated under reduced pressure to give piperidine acetamide **2i** (106 mg, 71%) as a

yellow oil, IR (ATR) 3256 (OH), 2918, 1624 (C=O), 1440, 1076, 522 cm^{-1}; ^{1}H NMR (400 MHz, $CDCl_3$) (55:45 mixture of rotamers) δ 4.57-4.48 (m, 0.45H, CH), 4.33 (ddd, J = 9.0, 4.5, 4.5 Hz, 0.55H, CH), 4.02 (dd, J = 11.5, 10.0 Hz, 0.45H, CH), 3.80-3.66 (m, 1.55H, CH), 3.61-3.55 (m, 0.45H, CH), 3.56-3.52 (m, 0.45H, CH), 3.27-3.17 (m, 0.55H, CH), 2.66-2.56 (m, 0.55H, CH), 2.13 (s, 1.55H, NC(O)Me), 2.13 (s, 1.45H, NC(O)Me), 1.91-1.81 (m, 1H, C*H*Me), 1.76-1.61 (m, 2H, CH), 1.54-1.46 (m, 0.55H, CH), 1.45-1.33 (m, 1.45H, CH, OH), 1.07 (d, J = 7.0 Hz, 1.35H, CH*Me*), 1.02 (d, J = 7.0 Hz, 1.65H, CH*Me*); ^{13}C NMR (100.6 MHz, $CDCl_3$) δ 172.3 (C=O, C(O)Me), 171.7 (C=O, C(O)Me), 62.6 (OCH_2), 61.9 (NCH), 61.5 (OCH), 58.3 (NCH), 42.8 (NCH_2), 36.5 (NCH_2), 28.5 (*C*HMe), 28.2 (*C*HMe), 26.7 (CH_2), 25.9 (CH_2), 22.2 (NC(O)*Me*), 22.0 (NC(O)*Me*), 21.1 (CH_2), 19.6 (CH_2), 18.8 (CH*Me*), 18.3 (CH*Me*); HRMS (ESI) *m/z* calcd for $C_9H_{17}NO_2$ $(M + Na)^+$ 194.1151, found 194.1146 (+2.7 ppm error).
Lab Book PJ-03-81/86.

Ethyl (2*R,3*R**)-1-benzyl-3-methylpiperidine-2-carboxylate S11**

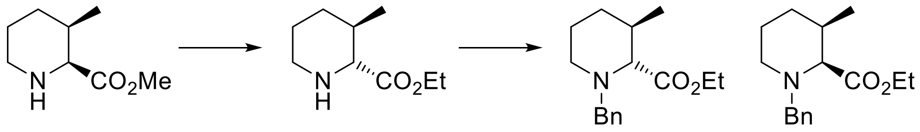

6b 85:15 dr **S10** 80:20 dr **S11** **S12**

NaOEt (2.5 mL of a 13 M solution in EtOH, 32.5 mmol, 3.0 eq.) was added dropwise to an 85:15 mixture of piperidine ester **6b** and **S5** (1.7 g, 10.8 mmol, 1.0 eq.) in EtOH (108 mL) at rt under Ar. The resulting solution was stirred and heated at reflux for 24 h. After being allowed to cool to rt, the solvent was evaporated under reduced pressure to give an orange oil. CH_2Cl_2 (100 mL) and water (100 mL) were added and the two layers were separated. The aqueous layer was extracted with CH_2Cl_2 (3 × 50 mL). The combined organic layers were dried ($MgSO_4$) and evaporated under reduced pressure to give the crude piperidines (1.04 g, 6.62 mmol assumed) as an orange oil which contained an 80:20 mixture (by ^{1}H NMR spectroscopy) of piperidine esters **S10**. PhCHO (0.74 mL, 7.28 mmol, 1.1 eq.) was added dropwise to a stirred solution of the crude piperidines, $NaBH(OAc)_3$ (2.8 g, 13.2 mmol, 2.0 eq.) and AcOH (0.08 mL, 1.32 mmol, 0.2 eq.) in DCE (66 mL) at rt under Ar. The resulting mixture was stirred at rt for 18 h. The reaction mixture was poured into saturated $NaHCO_{3(aq)}$ (100 mL) and extracted with CH_2Cl_2 (3 × 50 mL). The combined organics were washed with brine (100 mL), dried ($MgSO_4$) and evaporated under reduced pressure to give the crude product as an orange oil which contained a 75:25 mixture (by ^{1}H NMR spectroscopy) of *N*-benzyl piperidine ester **S11** and **S12**. Purification by flash column chromatography on silica with 9:1 hexane-EtOAc as eluent gave *N*-benzyl piperidine ester **S11** (975 mg, 36%) as a yellow oil, R_F (9:1 hexane-EtOAc) 0.18; IR (ATR) 2927, 1730 (C=O), 1453, 1176, 697 cm^{-1}; ^{1}H NMR (400

MHz, $CDCl_3$) δ 7.34-7.27 (m, 4H, Ph), 7.26-7.21 (m, 1H, Ph), 4.25 (q, J = 7.0 Hz, 2H, OCH_2), 3.73 (d, J = 13.5 Hz, 1H, NCHPh), 3.24 (d, J = 13.5 Hz, 1H, NCHPh), 2.89-2.83 (m, 1H, NCH), 2.61 (d, J = 9.0 Hz, 1H, $NCHCO_2$), 1.96-1.85 (m, 2H, CH), 1.76-1.68 (m, 1H, CH), 1.60-1.53 (m, 2H, CH), 1.31 (t, J = 7.0 Hz, 3H, OCH_2*Me*), 1.05-0.94 (m, 1H, CH), 0.91 (d, J = 6.5 Hz, 3H, CH*Me*); ^{13}C NMR (100.6 MHz, $CDCl_3$) δ 173.8 (C=O), 137.9 (*ipso*-Ph), 129.6 (Ph), 128.2 (Ph), 127.2 (Ph), 73.8 ($NCHCO_2$), 61.0 (O*C*H_2Me), 60.6 (N*C*H_2Ph), 51.3 (NCH_2), 34.3 (*C*HMe), 32.1 (CH_2), 24.6 (CH_2), 18.8 (CH*Me*), 14.5 (OCH_2*Me*); HRMS (ESI) *m/z* calcd for $C_{16}H_{23}NO_2$ $(M + H)^+$ 262.1802, found 262.1804 (–0.5 ppm error).
Lab Book – PJ-02-83/85.

Ethyl (2*R,3*R**)-3-methylpiperidine-2-carboxylate S13**

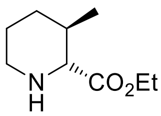

S13

A suspension of 20% $Pd(OH)_2/C$ (106 mg, 0.15 mmol, 10 mol%) in EtOH (10 mL) was added to a stirred solution of *N*-benzyl piperidine ester **S11** (395 mg, 1.51 mmol, 1.0 eq.) and $NH_4^+HCO_2^-$ (1.91 g, 30.3 mmol, 20.0 eq.) in EtOH (13 mL) at rt under Ar. The resulting suspension was stirred and heated at reflux for 16 h. After being allowed to cool to rt, the solids were removed by filtration through Celite and washed with EtOH (40 mL). The filtrate was evaporated under reduced pressure to give piperidine ester **S13** (159 mg, 62%) as a colourless oil, R_F (9:1 CH_2Cl_2-MeOH) 0.23; IR (ATR) 2926, 1731 (C=O), 1187, 1031, 733 cm^{-1}; ^{1}H NMR (400 MHz, $CDCl_3$) δ 4.17 (q, J = 7.0 Hz, 2H, O*CH_2*Me), 3.11-3.04 (m, 1H, NCH), 2.93 (d, J = 10.0 Hz, 1H, $NCHCO_2$), 2.55 (ddd, J = 12.0, 12.0, 4.0 Hz, 1H, NCH), 1.84-1.77 (m, 1H, CH), 1.66-1.54 (m, 2H, CH), 1.45 (ddddd, J = 12.0, 12.0, 12.0, 4.0, 4.0 Hz 1H, CH), 1.26 (t, J = 7.0 Hz, 3H, OCH_2*Me*), 1.14 (dddd, J = 12.0, 12.0, 12.0, 4.0 Hz, 1H, CH), 0.87 (d, J = 6.5 Hz, 3H, CH*Me*); ^{13}C NMR (100.6 MHz, $CDCl_3$) δ 173.5 (C=O), 66.6 (N*C*HCO_2), 60.7 (O*C*H_2Me), 45.9 (NCH_2), 34.9 (*C*HMe), 33.2 (CH_2), 26.8 (CH_2), 19.0 (CH*Me*), 14.4 (OCH_2*Me*); HRMS (ESI) *m/z* calcd for $C_9H_{17}NO_2$ $(M + H)^+$ 172.1332, found 172.1336 (+2.3 ppm error).
Lab Book – PJ-03-09.

Ethyl (2*R,3*R**)-1-methanesulfonyl-3-methylpiperidine-2-carboxylate S14**

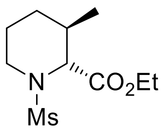

S14

Using general procedure D, Et_3N (0.13 mL, 0.96 mmol, 3.3 eq.), MsCl (70 µL, 0.96 mmol, 3.3 eq.) and piperidine ester **S13** (50 mg, 0.29 mmol, 1.0 eq.) in CH_2Cl_2 (5 mL) gave the crude product. Purification by flash column chromatography on silica with 9:1 hexane-EtOAc as eluent gave piperidine sulfonamide **S14** (62 mg, 86%) as a yellow oil, R_F (9:1 hexane-EtOAc) 0.2; IR (ATR) 2938, 1731 (C=O), 1320, 1139, 768 cm^{-1}; ^1H NMR (400 MHz, $CDCl_3$) δ 4.38 (br s, 1H, $NCHCO_2$), 4.25-4.17 (m, 2H, OCH_2Me), 3.72-3.68 (m, 1H, NCH), 3.19 (ddd, J = 12.0, 12.0, 3.0 Hz, 1H, NCH), 2.93 (s, 3H, SO_2Me), 2.58-2.50 (m, 1H, C*H*Me), 1.89-1.77 (m, 1H, CH), 1.53-1.50 (m, 1H, CH), 1.49-1.46 (m, 1H, CH), 1.46-1.41 (m, 1H, CH), 1.30 (t, J = 7.0 Hz, 3H, OCH_2Me), 1.20 (d, J = 7.0 Hz, 3H, CH*Me*); ^{13}C NMR (100.6 MHz, $CDCl_3$) δ 171.5 (C=O), 61.6 (OCH_2Me), 61.1 ($NCHCO_2$), 42.5 (NCH_2), 38.9 (SO_2Me), 29.7 (*C*HMe), 26.3 (CH_2), 19.2 (CH_2), 17.2 (OCH_2Me), 14.4 (CH*Me*); HRMS (ESI) *m/z* calcd for $C_{10}H_{19}NO_4S$ $(M + H)^+$ 250.1108, found 250.1114 (+2.7 ppm error).

Lab Book – PJ-03-14.

[(2*R,3*R**)-1-Methanesulfonyl-3-methylpiperidin-2-yl]methanol 2j**

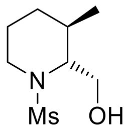

2j

Using general procedure F, $LiAlH_4$ (45 mg, 1.18 mmol, 2.0 eq.) and piperidine sulfonamide **S14** (148 mg, 0.59 mmol, 1.0 eq.) in THF (10 mL) gave the crude product as a yellow oil. Purification by flash column chromatography on silica with 95:5 CH_2Cl_2-MeOH as eluent gave piperidine alcohol **2j** (100 mg, 82%) as a white solid, mp 40-42 °C; R_F (95:5 CH_2Cl_2-MeOH) 0.2 ; IR (ATR) 3500 (OH), 2933, 1307, 1133, 766 cm^{-1}; ^1H NMR (400 MHz, $CDCl_3$) δ 3.94 (ddd, J = 11.0, 9.5, 6.0 Hz, 1H, NCH), 3.78-3.72 (m, 1H, NCH), 3.67-3.59 (m, 2H, OCH_2), 3.08-2.99 (ddd, J = 12.0, 12.0, 4.0 Hz, 1H, NCH), 2.97 (s, 3H, SO_2Me), 2.00-1.94 (m, 1H, OH), 1.90-1.82 (m, 1H, C*H*Me), 1.82-1.72 (m, 1H, CH), 1.69-1.66 (m, 2H, CH), 1.49-1.38 (m, 2H, CH), 1.13 (d, J = 7.0 Hz, 3H, CH*Me*); ^{13}C NMR (100.6 MHz, $CDCl_3$) δ 61.6 (OCH_2), 61.1 (NCH), 40.7 (NCH_2), 40.0 (SO_2Me), 28.1 (*C*HMe), 25.7 (CH_2), 20.1 (CH_2), 18.5 (CH*Me*); HRMS (ESI) *m/z* calcd for $C_8H_{17}NO_3S$ $(M + Na)^+$ 230.0821, found 230.0822 (0.0 ppm error).

Lab Book – PJ-03-34.

Methyl (2*R,6*R**)-6-methylpiperidine-2-carboxylate 6e**

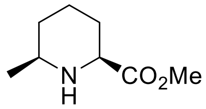

6e

Using general procedure I, PtO_2 (30 mg, 0.13 mmol, 10 mol%) and methyl 6-methylpyridine-2-carboxylate (0.18 mL, 1.32 mmol, 1.0 eq.) in glacial acetic acid (4.9 mL) gave piperidine ester **6e** (200 mg, 97%) as a colourless oil, IR (ATR) 2929, 1737 (C=O), 1436, 1212, 1056, 735 cm^{-1}; ^1H NMR (400 MHz, $CDCl_3$) δ 3.68 (s, 3H, OMe), 3.34 (dd, J = 11.0, 3.0 Hz, 1H, $NCHCO_2$), 2.62 (dqd, J = 12.5, 6.5, 2.5 Hz, 1H, NC*H*Me), 1.99-1.92 (m, 1H, CH), 1.88-1.80 (m, 1H, CH), 1.62-1.54 (m, 1H, CH), 1.45-1.26 (m, 2H, CH), 1.08 (d, J = 6.5 Hz, 3H, CH*Me*), 1.06-0.96 (m, 1H, CH); ^{13}C NMR (100.6 MHz, $CDCl_3$) δ 173.8 (C=O), 59.4 (NCH), 52.0 (OMe), 51.9 (N*C*HMe), 33.8 (CH_2), 29.0 (CH_2), 24.6 (CH_2), 22.9 (CH*Me*); HRMS (ESI) *m/z* calcd for $C_8H_{15}NO_2$ $(M + H)^+$ 158.1176, found 158.1174 (+1.3 ppm error).
Lab Book – PJ-05-88.

[(2*R,6*R**)-6-Methylpiperidin-2-yl]methanol S15**

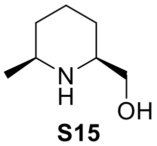

S15

Using general procedure F, $LiAlH_4$ (380 mg, 10.0 mmol, 2.0 eq.) and piperidine ester **6e** (800 mg, 5.1 mmol, 1.0 eq.) in THF (70 mL) gave the crude product as green oil. Purification by flash column chromatography on silica with 90:9:1 CH_2Cl_2-MeOH-$NH_4OH_{(aq)}$ as eluent gave piperidine alcohol **S15** (490 mg, 76%) as a white solid, mp 62-64 °C (lit.,[10] 93-94 ºC); R_F (90:9:1 CH_2Cl_2-MeOH-$NH_4OH_{(aq)}$) 0.03; IR (ATR) 3117 (OH or NH), 2927, 1488, 1061, 868 cm^{-1}; ^1H NMR (400 MHz, $CDCl_3$) δ 3.62 (dd, J = 10.5, 4.0 Hz, 1H, OCH), 3.43 (dd, J = 10.5, 8.0 Hz, 1H, OCH), 2.77-2.62 (m, 2H, NCH), 2.30 (br s, 2H, OH and NH) 1.84-1.76 (m, 2H, CH), 1.67-1.59 (m, 1H, CH), 1.56-1.49 (m, 1H, CH), 1.38 (ddddd, J = 13.0, 13.0, 13.0, 4.0, 4.0 Hz, 1H, CH), 1.09 (d, J = 6.5 Hz, 3H, CH*Me*), 1.14-0.98 (m, 1H, CH); ^{13}C NMR (100.6 MHz, $CDCl_3$) δ 67.1 (OCH_2), 58.3 (NCH), 52.1 (N*C*HMe), 34.4 (CH_2), 28.1 (CH_2), 24.5 (CH_2), 23.1 (CH*Me*); HRMS (ESI) *m/z* calcd for $C_7H_{15}NO$ $(M + H)^+$ 130.1226, found 130.1224 (+2.8 ppm error). Spectroscopic data consistent with those reported in the literature.[10]
Lab Book – PJ-03-72.

1-[(2*R,6*R**)-2-(Hydroxymethyl)-6-methylpiperidin-1-yl]ethan-1-one 2k**

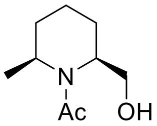

2k

Ac_2O (0.44 mL, 4.65 mmol, 6.0 eq.) was added dropwise to a stirred solution of piperidine alcohol **S15** (100 mg, 0.78 mmol, 1.0 eq.) in pyridine (7.8 mL) at rt under Ar. The resulting solution was stirred at rt for 16 h. The reaction mixture was evaporated under reduced pressure to give an orange oil. CH_2Cl_2 (20 mL) was added and the solution was washed with 15% $CuSO_{4(aq)}$ (3 × 80 mL) and saturated $EDTA_{(aq)}$ (3 × 20 mL). The combined organics were dried ($MgSO_4$) and evaporated under reduced pressure to give a colourless oil. MeOH (4 mL) was added followed by K_2CO_3 (165 mg, 1.2 mmol, 3.0 eq.). The resulting solution was stirred at rt for 30 min. The solution was poured into water (20 mL) and extracted with CH_2Cl_2 (3 × 20 mL). The combined organics were dried ($MgSO_4$) and evaporated under reduced pressure to give the crude product as an orange oil. Purification by flash column chromatography on silica with 95:5 CH_2Cl_2-MeOH as eluent gave piperidine acetamide **2k** (39 mg, 60%) as a colourless oil, R_F (95:5, CH_2Cl_2-MeOH) 0.21; IR (ATR) 3368 (OH), 2936, 1606 (C=O), 1416, 1372, 1049, 1009, 616 cm^{-1}; ^1H NMR (400 MHz, $CDCl_3$) (75:25 mixture of rotamers) δ 4.79-4.66 (m, 1H, NCH), 4.11-3.99 (m, 0.75H, NC*H*Me), 3.99-3.88 (m, 0.25H, NC*H*Me), 3.72-3.59 (m, 2H, OCH), 3.06-2.92 (m, 0.75H, OH), 2.92-2.74 (m, 0.25H, OH), 2.13 (s, 3H, NC(O)Me), 1.84-1.77 (m, 1H, CH), 1.70-1.44 (m, 5H, CH), 1.24 (d, J = 7.0 Hz, 2.25H, CH*Me*), 1.17–1.08 (m, 0.75H, CH*Me*); ^{13}C NMR (100.6 MHz, $CDCl_3$) δ 172.8 (C=O), 66.1 (OCH_2), 50.1 (NCH), 48.6 (N*C*HMe), 30.5 (CH_2), 25.0 (CH_2), 22.0 (C(O)*Me*), 21.2 (CH*Me*), 14.4 (CH_2); HRMS (ESI) *m/z* calcd for $C_9H_{17}NO_2$ $(M + Na)^+$ 194.1151, found 194.1146 (+2.5 ppm error).
Lab Book – PJ-03-50/68.

Methyl (2*R,6*R**)-1-methanesulfonyl-6-methylpiperidine-2-carboxylate S16**

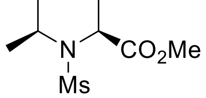

S16

MsCl (0.03 mL, 0.38 mmol, 1.2 eq.) was added dropwise to a stirred solution of piperidine ester **6e** (50 mg, 0.32 mmol, 1.0 eq.) in pyridine (3.2 mL) at 0 °C under Ar. The resulting solution was stirred at rt for 72 h. The reaction mixture was evaporated under reduced pressure to give the crude product. Purification

by flash column chromatography on silica with 80:20 hexane-EtOAc as eluent gave piperidine sulfonamide **S16** (45 mg, 60%) as a yellow oil, R_F (60:40 hexane-EtOAc) 0.33; IR (ATR) 2944, 1734 (C=O), 1315, 1160, 766 cm^{-1}; ^{1}H NMR (400 MHz, $CDCl_3$) δ 4.77-4.71 (m, 1H, $NCHCO_2$), 4.31-4.23 (m, 1H, NC*H*Me), 3.74 (s, 3H, OMe), 3.01 (s, 3H, SO_2Me), 2.35-2.28 (m, 1H, CH), 1.74-1.50 (m, 5H, CH), 1.15 (d, *J* = 7.0 Hz, 3H, CH*Me*); ^{13}C NMR (100.6 MHz, $CDCl_3$) δ 173.2 (C=O), 52.8 (NCH), 52.5 (OMe), 48.4 (N*C*HMe), 41.2 (SO_2Me), 30.5 (CH_2), 26.4 (CH_2), 18.2 (CH*Me*), 15.3 (CH_2); HRMS (ESI) *m/z* calcd for $C_9H_{17}NO_4S$ $(M + Na)^+$ 258.0770, found 258.0769 (+0.5 ppm error).
Lab Book – PJ-04-62.

[(2*R,6*R**)-1-Methanesulfonyl-6-methylpiperidin-2-yl]methanol 2l**

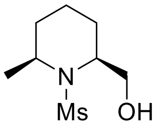

2l

Using general procedure F, $LiAlH_4$ (30 mg, 0.79 mmol, 2.0 eq.) and piperidine ester **S16** (92 mg, 0.39 mmol, 1.0 eq.) in THF (9 mL) gave the crude product as green oil. Purification by flash column chromatography on silica with 95:5 CH_2Cl_2-acetone as eluent gave piperidine sulfonamide **2l** (63 mg, 78%) as a white solid, mp 58-60 °C; R_F (95:5 CH_2Cl_2-acetone) 0.06; IR (ATR) 3505 (OH), 2941, 1305, 1138, 966, 766 cm^{-1}; ^{1}H NMR (400 MHz, $CDCl_3$) δ 4.21-4.13 (m, 1H, NC*H*Me), 4.03-3.98 (m, 1H, NCH), 3.79 (ddd, *J* = 11.0, 8.0, 5.5 Hz, 1H, HOC*H*), 3.66-3.59 (m, 1H, HOC*H*), 2.89 (s, 3H, SO_2Me), 2.03 (dd, *J* = 8.0, 8.0 Hz, 1H, OH), 1.82-1.76 (m, 1H, CH), 1.73-1.51 (m, 5H, CH), 1.34 (d, *J* = 7.0 Hz, 3H, CH*Me*); ^{13}C NMR (100.6 MHz, $CDCl_3$) δ 64.6 (OCH_2), 53.7 (NCH), 48.1 (N*C*HMe), 40.0 (SO_2Me), 30.3 (CH_2), 25.4 (CH_2), 22.2 (CH*Me*), 14.3 (CH_2); HRMS (ESI) *m/z* calcd for $C_8H_{17}NO_3S$ $(M + Na)^+$ 230.0821, found 230.0814 (+3.7 ppm error).
Lab Book – PJ-03-60.

Structure confirmed by small molecule X-Ray crystallography (CCDC 1995341):

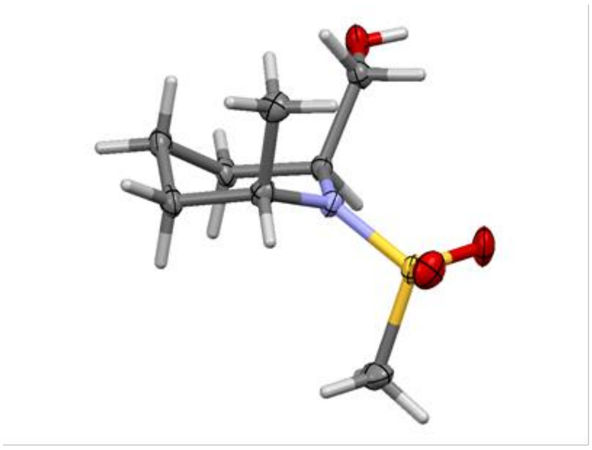

Methyl 2-methylpyridine-3-carboxylate 5a

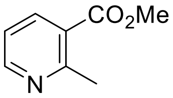

5a

Using general procedure H, thionyl chloride (291 μL, 4.01 mmol, 1.1 eq.) and 2-methylnicotinic acid (500 mg, 3.65 mmol, 1.0 eq.) and MeOH (5 mL) gave ester **5a** (507 mg, 92%) as a colourless oil, R_F (100:9:1 CH_2Cl_2-MeOH-$NH_4OH_{(aq)}$) 0.8; ^{1}H NMR (400 MHz, $CDCl_3$) δ 8.61 (dd, J = 5.0, 1.5 Hz, 1H, Ar), 8.18 (dd, J = 8.0, 1.5 Hz, 1H, Ar), 7.20 (dd, J = 8.0, 5.0 Hz, 1H, Ar), 3.91 (s, 3H, OMe), 2.83 (s, 3H, Me); ^{13}C NMR (100.6 MHz, $CDCl_3$) δ 167.1 (C=O), 160.0 (*ipso*-Ar), 151.9 (*ipso*-Ar), 138.5 (Ar), 125.4 (Ar), 120.9 (Ar), 52.3 (OMe), 24.9 (Me). Spectroscopic data consistent with those reported in the literature.[11]

Lab book reference MA 5-15

Methyl (2*R,3*R**)-2-methylpiperidine-3-carboxylate 6a**

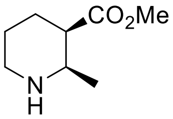

6a

Using general procedure I, PtO_2 (250 mg, 1.33 mmol, 20 mol%) and pyridine **5a** (1.0 g, 6.62 mmol, 1.0 eq.) in glacial acetic acid (10 mL) gave the crude product. Purification by flash column chromatography on silica with 200:9:1 CH_2Cl_2-MeOH-$NH_4OH_{(aq)}$ as eluent gave piperidine **6a** (971 mg, 93%) as a colourless oil, R_F (100:9:1 CH_2Cl_2-MeOH-$NH_4OH_{(aq)}$) 0.5; ^{1}H NMR (400 MHz, $CDCl_3$) δ 3.67 (s, 3H,

OMe), 3.07 (ddd, J = 13.5, 4.0, 4.0 Hz, 1H, CH), 3.01 (dddd, J = 7.0, 7.0, 7.0, 4.0 Hz, 1H, CH), 2.68 (ddd, J = 13.5, 10.0, 3.5 Hz, 1H, CH), 2.57 (dd, J = 8.5, 4.0 Hz, 1H, CH), 2.53-2.31 (m, 2H, CH and NH), 2.15-1.93 (m, 1H, CH), 1.81-1.62 (m, 2H, CH), 1.45-1.36 (m, 1H, CH), 1.12 (d, J = 7.0 Hz, 3H, CH*Me*); ^{13}C NMR (100.6 MHz, $CDCl_3$) δ 174.5 (C=O), 52.3 (CH), 51.3 (OMe), 44.9 (NCH_2), 44.0 (CH), 26.1 (CH_2), 22.5 (CH_2), 18.8 (Me). Spectroscopic data consistent with those reported in the literature.[12]
Lab book reference MA 5-88

[(2*R,3*R**)-2-Methylpiperidin-3-yl]methanol hydrochloride 2m**

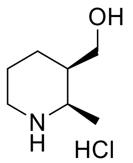

2m

Using general procedure F, $LiAlH_4$ (217 mg, 5.73 mmol, 3.0 eq.) and piperidine ester **6a** (300 mg, 1.91 mmol, 1.0 eq.) in THF (19 mL) gave the crude product. Purification by flash column chromatography on silica with 100:9:1 CH_2Cl_2-MeOH-$NH_4OH_{(aq)}$ as eluent gave crude alcohol (121 mg) as a colourless oil. Then, HCl (1.0 mL of a 2 M solution in Et_2O, 2.0 mmol) was added and the solvent was evaporated under reduced pressure to give **2m**·HCl (155 mg, 49%), IR (ATR) 3343 (NH_2 or OH), 2942, 1449 cm^{-1}; ^{1}H NMR (400 MHz, MeOH-d_4) δ 3.59-3.51 (m, 2H, OCH), 3.47-3.39 (m, 1H, NCH), 3.12-3.03 (m, 1H, NCH), 3.00-2.93 (m, 1H, NCH), 1.94-1.80 (m, 2H, CH), 1.66-1.43 (m, 3H, CH and OH), 1.23 (d, J = 7.0 Hz, 3H, CH*Me*); ^{13}C NMR (100.6 MHz, MeOH-d_4) δ 63.5 (OCH_2), 52.9 (NCH_2), 41.9 (NCH), 39.5 (CH_2), 23.6 (CH), 22.3 (Me), 12.3 (CH_2); HRMS (ESI) *m/z* calcd for $C_7H_{16}NO$ M^+ 130.1226, found 130.1228 (–0.6 ppm error).
Lab book reference MA 5-90

[(2*R,3*R**)-1,2-Dimethylpiperidin-3-yl]methanol 2n**

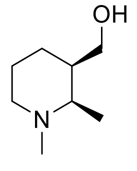

2n

Using general procedure B, NaBH(OAc)$_3$ (318 mg, 1.5 mmol, 1.5 eq.), piperidine **2m** (171 mg, 1.0 mmol, 1.0 eq.), 37% HCHO$_{(aq)}$ (743 µL, 10.0 mmol, 10 eq.) and MgSO$_4$ (1.0 g) in 4:1 CH$_2$Cl$_2$-AcOH (10 mL) gave the crude product. Purification by flash column chromatography on silica with 100:9:1 CH$_2$Cl$_2$-MeOH-NH$_4$OH$_{(aq)}$ as eluent gave piperidine **2n** (127 mg, 88%) as a colourless oil, R_F (100:9:1 CH$_2$Cl$_2$-MeOH-NH$_4$OH$_{(aq)}$) 0.3; IR (ATR) 3368 (OH), 2932, 1446 cm^{-1}; ^{1}H NMR (400 MHz, CDCl$_3$) δ 4.20 (dd, J = 10.0, 3.0 Hz, 1H, OCH), 3.62 (dd, J = 11.0, 3.0 Hz, 1H, OCH), 2.79-2.74 (m, 1H, NCH), 2.46 (br s, 1H, NCH), 219 (s, 3H, NMe), 2.15 (dd, J = 11.0, 2.5 Hz, 1H, NCH), 2.11-2.01 (m, 1H, CH), 1.75-1.67 (br m, 1H, CH), 1.65-1.51 (m, 3H, CH), 1.16 (d, J = 7.0 Hz, 3H, CH*Me*); ^{13}C NMR (100.6 MHz, CDCl$_3$) δ 74.8 (NMe), 65.2 (CH), 60.7 (OCH$_2$), 40.7 (CH$_2$), 42.9 (CH$_2$), 40.2 (CH$_2$), 24.0 (Me) (one CH resonance not resolved); HRMS (ESI) *m/z* calcd for C$_8$H$_{17}$NO (M + H)$^+$ 144.1383, found 144.1383 (0.1 ppm error).

Lab book reference MA 6-28

Ethyl (2*R,3*S**)-2-methylpiperidine-3-carboxylate S17**

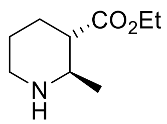

S17

A solution of NaOEt (21 wt% in EtOH, 2.78 mL, 8.6 mmol, 3.0 eq.) was added to a stirred solution of amine **6a** (450 mg, 2.87 mmol, 1.0 eq.) in EtOH (28 mL) at rt under Ar. The resulting solution was stirred and heated at reflux for 24 h. After being allowed to cool to rt, the solvent was evaporated under reduced pressure to give the crude product which contained an 85:15 mixture (by ^{1}H NMR spectroscopy) of **S17** and its *cis* diastereomer. Purification by flash column chromatography on silica with 100:9:1 CH$_2$Cl$_2$-MeOH-NH$_4$OH$_{(aq)}$ as eluent gave amine **S17** (137 mg, 31%) as a yellow oil, R_F (100:9:1 CH$_2$Cl$_2$-MeOH-NH$_4$OH$_{(aq)}$) 0.3; IR (ATR) 2935, 1722 (C=O), 1171, 1128 cm^{-1}; ^{1}H NMR (400 MHz, CDCl$_3$) δ 4.12 (q, J = 7.0 Hz, 2H, OCH$_2$), 3.07-3.01 (dddd, J = 12.5, 4.0, 2.0, 2.0 Hz, 1H, NCH), 2.80 (dq, J = 10.0, 6.5 Hz, 1H, NCH), 2.67 (dddd, J = 12.5, 12.5, 3.0 Hz, 1H, NCH), 2.07-2.00 (m, 1H, CH), 1.99-1.93 (m, 1H,

CH), 1.70-1.61 (m, 1H, CH), 1.60-1.52 (m, 2H, CH), 1.46-1.34 (m, 1H, CH), 1.25 (t, J = 7.0 Hz, 3H, CH_2*Me*), 1.06 (d, J = 6.5 Hz, 3H, CH*Me*); ^{13}C NMR (100.6 MHz, $CDCl_3$) δ 175.0 (C=O), 60.2 (OCH_2), 53.6 (CH), 50.8 (CH), 46.5 (CH_2), 28.5 (CH_2), 25.6 (CH_2), 21.0 (Me), 14.3 (Me); HRMS (ESI) *m/z* calcd for $C_9H_{17}NO_2$ $(M + H)^+$ 172.1332, found 172.1338 (–3.4 ppm error).
Lab Book Reference: MA 5-94

[(2*R,3*S**)-2-Methylpiperidin-3-yl]methanol S18**

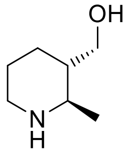

S18

Using general procedure F, $LiAlH_4$ (234 mg, 6.18 mmol, 3.0 eq.) and piperidine ester **S17** (353 mg, 2.06 mmol, 1.0 eq.) in THF (21 mL) gave **S18** (223 mg, 84%) as a colourless oil, R_F (100:9:1 CH_2Cl_2-MeOH-$NH_4OH_{(aq)}$) 0.2; IR (ATR) 3270, 2922, 1440 cm^{-1}; ^{1}H NMR (400 MHz, $CDCl_3$) δ 3.65 (dd, J = 11.0, 3.0 Hz, 1H, OCH), 3.51 (dd, J = 11.0, 5.5 Hz, 1H, OCH), 3.02 (dddd, J = 12.0, 4.0, 2.0, 2.0 Hz, 1H, NCH), 2.61 (ddd, J = 12.0, 12.0, 3.0 Hz, 1H, NCH), 2.48 (dddd, J = 9.0, 6.5, 6.5, 6.5 Hz, 1H, NCH) 1.91-1.82 (m, 1H, CH), 1.73-1.66 (m, 1H, CH), 1.48 (dddd, J = 24.0, 12.0, 4.0, 4.0 Hz, 1H, CH), 1.27-1.16 (m, 1H, CH), 1.11 (d, J = 6.5 Hz, 3H, CH*Me*); ^{13}C NMR (100.6 MHz, $CDCl_3$) δ 65.2 (OCH_2), 54.0 (CH), 46.8 (CH_2), 45.9 (CH), 28.2 (CH_2), 26.5 (CH_2), 20.8 (Me); HRMS (ESI) *m/z* calcd for $C_7H_{16}NO$ $(M + H)^+$ 130.1226, found 130.1224 (+1.6 ppm error).
Lab book reference MA 6-8

[(2*R,3*S**)-1,2-Dimethylpiperidin-3-yl]methanol 2o**

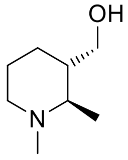

2o

Using general procedure B, $NaBH(OAc)_3$ (550 mg, 2.59 mmol, 1.5 eq.), piperidine **S18** (223 mg, 1.73 mmol, 1.0 eq.), 37% $HCHO_{(aq)}$ (743 µL, 10.0 mmol, 10 eq.) and $MgSO_4$ (1.4 g) in 4:1 CH_2Cl_2-AcOH (17 mL) gave the crude product. Purification by flash column chromatography on silica with 100:9:1 CH_2Cl_2-MeOH-$NH_4OH_{(aq)}$ as eluent gave **2o** (149 mg, 60%) as a colourless oil, R_F (100:9:1 CH_2Cl_2-MeOH-$NH_4OH_{(aq)}$) 0.3; IR (ATR) 3325 (OH), 2926, 1458, 1444 cm^{-1}; ^{1}H NMR (400 MHz, $CDCl_3$) δ 3.74-3.72

(m, 2H, OCH), 2.76-2.70 (m, 1H, NCH), 2.34-2.25 (m, 1H, NCH), 2,28 (s, 3H, NMe), 2.21 (ddd, *J* = 12.0, 9.0, 3.0 Hz, 1H, NCH), 1.85-1.75 (m, 2H, CH), 1.67-1.56 (m, 1H, CH), 1.47-1.39 (m, 1H, CH), 1.38-1.30 (m, 1H, CH), 1.11 (d, *J* = 6.5 Hz, 3H, CH*Me*); ^{13}C NMR (100.6 MHz, $CDCl_3$) δ 67.1 (OCH_2), 59.9 (NCH), 53.7 (NCH_2), 43.1 (NMe), 42.6 (CH), 26.4 (CH_2), 24.5 (CH_2), 14.0 (Me); HRMS (ESI) *m/z* calcd for $C_8H_{17}NO_2$ $(M + H)^+$ 144.1383, found 144.1384 (−0.9 ppm error).
Lab book reference MA 6-9

1-[(2*R,3*S**)-3-(Hydroxymethyl)-2-methylpiperidin-1-yl]ethan-1-one 2p**

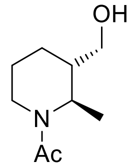

2p

Ac_2O (570 μL, 6.0 mmol, 6.0 eq.) was added to a stirred solution of **2o** (130 mg, 1.0 mmol, 1.0 eq.) in pyridine (10 mL) and the resulting solution was stirred at rt for 16 h. Then, the solvent was evaporated under reduced pressure. The residue was dissolved in MeOH (10 mL) and K_2CO_3 (415 mg, 3.0 mmol, 3.0 eq.) was added. The resulting mixture was stirred at rt for 1 h. H_2O (10 mL) was added and the mixture was extracted with CH_2Cl_2 (3 × 10 mL). The combined organic extracts were dried ($MgSO_4$) and evaporated under reduced pressure to give the crude product. Purification by flash column chromatography on silica with 10:1 CH_2Cl_2-MeOH as eluent gave **2p** (130 mg, 76%) as a colourless oil, R_F (10:1 CH_2Cl_2-MeOH) 0.7; IR (ATR) 3369 (OH), 2923, 1728 (C=O), 1435, 1213 cm^{-1}; ^{1}H NMR (400 MHz, $CDCl_3$) (60:40 mixture of rotamers) δ 4.95 (dd, *J* = 14.5, 8.0 Hz, 0.6H, OCH), 4.48 (br d, *J* = 13.5 Hz, 0.4H, OCH), 4.19 (dd, *J* = 14.5, 8.0 Hz, 0.4H, OCH), 3.72-3.65 (m, 0.4H, NCH), 3.63-3.40 (m, 2.6H, NCH, OH), 3.30-3.22 (m, 0.6H, NCH), 2.80-2.74 (m, 0.6H, NCH), 2.70-2.64 (m, 0.4H, NCH), 2.10 (s, 1.2H, C(O)Me), 2.09 (s, 1.8H, C(O)Me), 1.91-1.68 (m, 2H, CH), 1.52-1.42 (m, 2H, CH), 1.30 (d, *J* = 6.5 Hz, 1.2H, CH*Me*), 1.25 (d, *J* = 6.5 Hz, 1.8H, CH*Me*); ^{13}C NMR (100.6 MHz, $CDCl_3$) (rotamers) δ 170.5 (C=O), 170.0 (C=O), 63.0 (OCH_2), 62.4 (OCH_2), 49.0 (CH), 43.6 (CH), 41.3 (CH_2), 40.9 (CH), 40.1 (CH), 35.6 (CH_2), 21.75 (Me), 21.70 (CH_2), 21.6 (Me), 20.8 (CH_2), 20.7 (CH_2), 20.2 (CH_2), 17.3 (Me), 16.4 (Me); HRMS (ESI) *m/z* calcd for $C_9H_{17}NO_2$ $(M + Na)^+$ 194.1151, found 194.1151 (+0.5 ppm error).
Lab book reference MA 6-12 and MA 6-13

Methyl 5-methylpyridine-3-carboxylate 5f

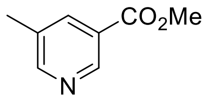

5f

Using general procedure H, $SOCl_2$ (58 µL, 0.80 mmol, 1.1 eq.) and 5-methylpyridine-3-carboxylic acid (100 mg, 0.73 mmol, 1.0 eq.) in MeOH (5 mL) gave pyridine **5f** (106 mg, 96%) as a white solid, mp 37-39 °C; IR (ATR) 2960, 1712 (C=O), 1574, 1293, 1108, 766 cm^{-1}; ^1H NMR (400 MHz, $CDCl_3$) δ 9.03 (d, J = 2.0 Hz, 1H, Ar), 8.61 (d, J = 2.0 Hz, 1H, Ar), 8.13-8.09 (m, 1H, Ar), 3.95 (s, 3H, OMe), 2.40 (s, 3H, Me); ^{13}C NMR (100.6 MHz, MeOH-d_4) δ 166.9 (C=O), 154.3 (Ar), 148.2 (Ar), 139.1 (Ar), 135.7 (*ipso*-Ar), 127.5 (*ipso*-Ar), 52.9 (OMe), 18.2 (Me); HRMS (ESI) *m/z* calcd for $C_8H_9NO_2$ $(M + H)^+$ 152.0706, found 152.0702 (+2.7 ppm error). Spectroscopic data consistent with those reported in the literature.[13]

Lab Book – PJ-04-81.

Methyl (3*R,5*R**)-5-methylpiperidine-3-carboxylate S19 and methyl (3*R**,5S*)-5-methylpiperidine-3-carboxylate 6f**

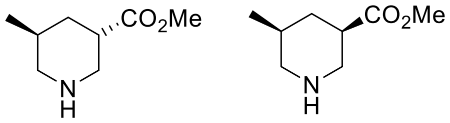

S19 **6f**

Using general procedure I, 10% Pd/C (70 mg, 0.07 mmol, 10 mol%) and pyridine **5f** (100 mg, 0.66 mmol, 1.0 eq.) in AcOH (5 mL) gave the crude product which contained a 70:30 mixture (by ^1H NMR spectroscopy) of piperidines esters **S19** and **6f** (94 mg, 91%) as a colourless oil, IR (ATR) 2951, 1725 (C=O), 1435, 1198, 1177, 860 cm^{-1}; ^1H NMR (400 MHz, $CDCl_3$) δ 3.69 (s, 2.1H, OMe), 3.65 (s, 0.9H, OMe), 3.30-3.23 (m, 1H, NCH), 2.97-2.90 (m, 1H, NCH), 2.73 (dd, J = 13.0, 3.5 Hz, 0.70H, NCH), 2.59-2.54 (m, 1H, $CHCO_2$), 2.51-2.43 (m, 0.3H, NCH), 2.24 (dd, J = 13.0, 10.0 Hz, 0.7H, NCH), 2.15-2.04 (m, 1.3H, NCH, CH), 1.69-1.57 (m, 0.7H, C*H*Me), 1.57-1.47 (m, 0.3H, C*H*Me), 1.39-1.30 (m, 0.7H, NCH), 1.17 (m, 0.3H, CH), 0.86 (d, J = 6.6 Hz, 0.9H, CH*Me*), 0.84 (d, J = 6.7 Hz, 2.1H, CH*Me*); ^{13}C NMR (100.6 MHz, $CDCl_3$) δ 175.4 (C=O), 174.7 (C=O), 53.8 (NCH_2), 53.7 (NCH_2), 51.8 (OMe), 51.7 (OMe), 48.3 (NCH_2), 47.4 (NCH_2), 43.4 (*C*HCO_2), 39.7 (*C*HCO_2), 36.2 (CH_2), 34.5 (CH_2), 31.8 (*C*HMe), 28.8 (*C*HMe), 19.5 (CH*Me*), 19.1 (CH*Me*); HRMS (ESI) *m/z* calcd for $C_8H_{16}NO_2$ $(M + H)^+$ 158.1176, found 158.1171 (–1.5 ppm error).

Lab Book – PJ-05-25.

Methyl (3*R,5*R**)-1-benzyl-5-methylpiperidine-3-carboxylate S20 and methyl (3*R**,5*S**)-1-benzyl-5-methylpiperidine-3-carboxylate S21**

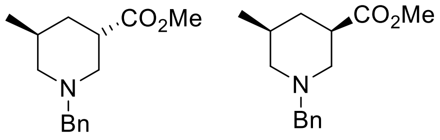

S20 **S21**

BnBr (0.09 mL, 0.76 mmol, 1.2 eq.) was added dropwise to a 70:30 mixture of piperidine esters **S19** and **6f** (100 mg, 0.63 mmol, 1.0 eq.) and Et_3N (0.11 mL, 0.79 mmol, 1.2 eq.) in CH_2Cl_2 (4 mL) at rt under Ar. The resulting solution was stirred at rt for 16 h. The two layers were separated, and the aqueous layer was extracted with CH_2Cl_2 (4 × 10 mL). The combined organics were dried ($MgSO_4$) and evaporated under reduced pressure to give the crude product as a colourless oil. Purification by flash column chromatography on silica with 90:10 hexane-EtOAc as eluent gave *N*-benzyl piperidine ester **S20** (77 mg, 51%) as a colourless oil, R_F (90:10 hexane-EtOAc) 0.14; IR (ATR) 2949, 1732 (C=O), 1453, 1199, 1150, 697 cm^{-1}; ^1H NMR (400 MHz,$CDCl_3$) δ 7.31-7.28 (m, 4H, Ph), 7.25-7.22 (m, 1H, Ph), 3.67 (s, 3H, OMe), 3.56 (d, J = 13.5 Hz, 1H, NC*H*Ph), 3.39 (d, J = 13.5 Hz, 1H, NC*H*Ph), 2.96-2.88 (m, 1H, NCH), 2.69-2.63 (m, 1H, $CHCO_2$), 2.63-2.57 (m, 1H, NCH), 2.31-2.23 (m, 1H, NCH), 2.06-1.94 (m, 2H, C*H*Me and CH), 1.91-1.83 (m, 1H, NCH), 1.24-1.14 (m, 1H, CH), 0.92 (d, J = 6.5 Hz, 3H, CH*Me*); ^{13}C NMR (100.6 MHz, $CDCl_3$) δ 174.9 (C=O), 138.8 (*ipso*-Ph), 128.9 (Ph), 128.2 (Ph), 127.0 (Ph), 63.1 (N*C*H_2Ph), 61.3 (NCH_2), 54.9 (NCH_2), 51.6 (OMe), 39.7 (*C*HCO_2), 33.4 (CH_2), 28.1 (*C*HMe), 19.2 (CH*Me*); HRMS (ESI) *m/z* calcd for $C_{15}H_{22}NO_2$ $(M + H)^+$ 248.1645, found 248.1646 (–0.9 ppm error) and *N*-benzyl piperidine ester **S21** (27 mg, 17%) as a colourless oil, R_F (90:10 hexane-EtOAc) 0.05; IR (ATR) 2951, 1732 (C=O), 1434, 1136, 1154, 697 cm^{-1}; ^1H NMR (400 MHz, $CDCl_3$) δ 7.34-7.29 (m, 4H, Ph), 7.280-7.24 (m, 1H, Ph), 3.64 (s, 3H, OMe), 3.52 (s, 2H, NC*H*$_2$Ph), 3.12-3.06 (m, 1H, NCH), 2.84-2.79 (m, 1H, NCH), 2.64 (dddd, J = 12.0, 12.0, 4.0, 4.0 Hz, 1H, $CHCO_2$), 2.04-1.98 (m, 1H, CH), 1.95 (dd, J = 12.0, 12.0 Hz, 1H, NCH), 1.78-1.66 (m, 1H, C*H*Me), 1.55 (d, J = 12.0 Hz, 1H, NCH), 1.02 (ddd, J = 12.0, 12.0, 12.0 Hz, 1H, CH), 0.87 (d, J = 6.5 Hz, 3H, CH*Me*); ^{13}C NMR (100.6 MHz, $CDCl_3$) δ 174.9 (C=O), 140.1 (*ipso*-Ph), 129.2 (Ph), 128.4 (Ph), 127.1 (Ph), 63.2 (N*C*H_2Ph), 61.2 (NCH_2), 55.1 (NCH_2), 51.7 (OMe), 42.3 ($CHCO_2$), 35.9 (CH_2), 30.6 (*C*HMe), 19.5 (CH*Me*); HRMS (ESI) *m/z* calcd for $C_{15}H_{21}NO_2$ $(M + H)^+$ 248.1645, found 248.1647 (–1.1 ppm error).

Lab Book – PJ-06-36.

[(3*R,5*R**)-1-Benzyl-5-methylpiperidin-3-yl]methanol S22**

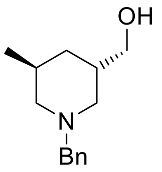

S22

Using general procedure F, $LiAlH_4$ (71 mg, 1.86 mmol, 2.0 eq.) and piperidine ester **S20** (230 mg, 0.93 mmol, 1.0 eq.) in THF (5 mL) gave **S22** (202 mg, 99%) as a colourless oil, IR (ATR) 3027, 2907, 2795, 1454, 1066, 1035, 736, 697, 605, 464 cm^{-1}; ^1H NMR (400 MHz, $CDCl_3$) δ 7.37–7.21 (m, 5H, Ph), 4.40 (br s, 1H, OH), 3.87 (dd, J = 10.5, 4.0 Hz, 1H, OCH), 3.78–3.71 (m, 1H, OCH), 3.43 (s, 2H, NCH_2Ph), 2.86 (br d, J = 11.0 Hz, 1H, NCH), 2.75 (br d, J = 11.0 Hz, 1H, NCH), 2.25 (br d, J = 11.0 Hz, 1H, NCH), 2.13 (ddd, J = 15.0, 10.0, 5.0 Hz, 1H, CH), 1.84–1.57 (m, 4H, NCH, CH), 1.18 (ddd, J = 12.5, 12.5, 5.0 Hz, 1H, CH), 0.82 (d, J = 6.5 Hz, 3H, CH*Me*); ^{13}C NMR (100.6 MHz, $CDCl_3$) δ 138.0 (*ipso*-Ph), 128.9 (Ph), 128.3 (Ph), 127.1 (Ph), 69.0 (OCH_2), 63.4 (NCH_2Ph), 61.5 (NCH_2), 57.4 (NCH_2), 36.8 (CH_2), 34.6 (CH), 28.4 (CH), 20.0 (Me); HRMS (ESI) *m/z* calcd for $C_{14}H_{21}NO_2$ $(M + H)^+$ 220.1696, found 220.1694 (+0.9 ppm error).

Lab book reference JDF_B_358

(3*R,5*R**)-3-(Hydroxymethyl)-5-methylpiperidin-1-ium acetate 2q**

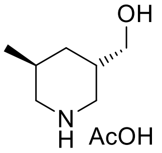

2q

$Pd(OH)_2/C$ (18 mg, 10 wt%) was added to a stirred solution of amine **S22** (178 mg, 0.81 mmol, 1.0 eq.) and glacial acetic acid (232 µL, 4.1 mmol, 5.0 eq.) in EtOH (10 mL). The reaction flask evacuated under reduced pressure and back-filled with N_2 three times. After a final evacuation, a balloon of hydrogen was added and the reaction mixture was stirred vigorously at rt for 16 h. The solids were removed by filtration through Celite and washed with EtOH (30 mL). The filtrate was evaporated under reduced pressure to give **2q**·AcOH (139 mg, 91%), as a clear oil, IR (ATR) 2927, 1556, 1403, 1259, 1040, 651, 616, 536, 491cm^{-1}; ^1H NMR (400 MHz, $CDCl_3$) δ 3.74 (dd, J = 11.0, 9.5 Hz, 1H, OCH), 3.60 (dd, J = 11.0, 5.0

Hz, 1H, OCH), 3.35 (br d, J = 12.5 Hz, 1H, NCH), 3.16 (dd, J = 12.5, 3.5 Hz, 1H, NCH), 2.88 (dd, J = 12.5, 4.0 Hz, 1H, NCH), 2.49–2.41 (m, 1H, NCH), 2.10–1.99 (m, 2H, CH), 1.94 (s, 3H, Me), 1.67–1.59 (m, 1H, CH), 1.41–1.28 (m, 1H, CH), 0.93 (d, J = 6.5 Hz, 3H, CH*Me*); ^{13}C NMR (100.6 MHz, $CDCl_3$) δ 178.1 (C=O, $MeCO_2^-$), 62.3 (OCH_2), 49.6 (NCH_2), 44.0 (NCH_2), 32.9 (CH_2), 32.8 (CH), 24.9 (CH), 23.3 (*Me*CO_2^-), 18.8 (Me); HRMS (ESI) *m/z* calcd for $C_7H_{16}NO$ M^+ 130.1226, found 130.1225 (+1.2 ppm error).

Lab book reference JDF_B_363

Methyl (3*R,6*S**)-6-methylpiperidine-3-carboxylate 6g and methyl (3*R**,6*R**)-6-methylpiperidine-3-carboxylate S23**

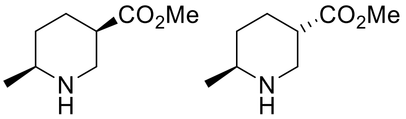

6g **S23**

Using general procedure I, PtO_2 (60 mg, 0.26 mmol, 10 mol%) and methyl-6-methylnicotinate (400 mg, 2.64 mmol, 1.0 eq.) in AcOH (4 mL) gave the crude product which contained an 85:15 mixture (by ^{1}H NMR spectroscopy) of piperidine esters **6g** and **S23** (386 mg, 93%) as a colourless oil which was used without further purification, ^{1}H NMR (400 MHz, $CDCl_3$) δ 3.72 (s, 2.55H, OMe), 3.66 (s, 0.45H, OMe), 3.43 (ddd, J = 13.0, 2.5, 2.5 Hz, 0.85H, NCH), 3.35–3.25 (m, 0.15H, NCH), 2.82 (dd, J = 13.0, 3.5 Hz, 0.85H, NCH), 2.73 (dd, J = 11.5 Hz, 0.15H, NCH), 2.68–2.56 (m, 1H, NCH), 2.50-2.47 (m, 0.85H, CH), 2.45–2.36 (m, 0.15H, CH), 2.23–2.14 (m, 0.85H, CH), 2.10–2.02 (m, 0.15H, CH), 1.72–1.61 (m, 1H, CH), 1.60–1.39 (m, 2H, CH), 1.25–1.12 (m, 1H, CH), 1.08–1.02 (m, 3H, CH*Me*).

Lab Book – PJ-04-56.

1-*tert*-Butyl 3-methyl (3*R,6*S**)-6-methylpiperidine-1,3-dicarboxylate S24 and 1-*tert*-butyl 3-methyl (3*R**,6*R**)-6-methylpiperidine-1,3-dicarboxylate S25**

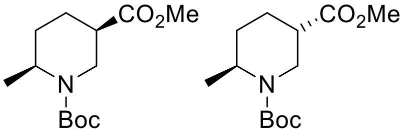

S24 **S25**

Et_3N (4.15 mL, 29.8 mmol, 3.0 eq.) was added dropwise to a stirred solution of an 85:15 mixture of piperidine esters **6g** and **S23** (1.5 g, 9.93 mmol, 1.0 eq.), Boc_2O (2.16 g, 9.93 mmol, 1.0 eq.) and DMAP (60 mg, 4.97 mmol, 0.05 eq.) in CH_2Cl_2 (100 mL) at rt under Ar. The resulting solution was stirred at rt for 16 h. Then, the reaction mixture was evaporated under reduced pressure to give the crude product. Purification by flash column chromatography on silica with 9:1 hexane-EtOAc as eluent gave Boc piperidine **S24** (1.73 g, 68%) as a colourless oil, R_F (9:1 hexane-EtOAc) 0.22; IR (ATR) 2973, 1734 (C=O, CO_2Me), 1688 (C=O, Boc), 1408, 1161, 871 cm^{-1}; ^1H NMR (400 MHz, $CDCl_3$) δ 4.52-4.29 (m, 1H, NC*H*Me), 4.29-4.04 (m, 1H, NCH), 3.69 (s, 3H, OMe), 2.97-2.82 (m, 1H, NCH), 2.45-2.33 (m, 1H, $CHCO_2$), 1.98-1.85 (m, 1H, CH), 1.79-1.65 (m, 2H, CH), 1.59-1.50 (m, 1H, CH), 1.45 (s, 9H, CMe_3), 1.12 (d, J = 7.0 Hz, 3H, CH*Me*); ^{13}C NMR (100.6 MHz, $CDCl_3$) δ 174.1 (C=O, CO_2Me), 154.7 (C=O, Boc), 79.6 (O*C*Me_3), 51.7 (OMe), 45.0 (N*C*HMe), 41.9 (*C*HCO_2), 39.7 (NCH_2), 29.1 (CH_2), 28.4 (C*Me_3*), 22.1 (CH_2), 15.5 (CH*Me*); HRMS (ESI) *m/z* calcd for $C_{13}H_{23}NO_4$ $(M + Na)^+$ 280.1519, found 280.1507 (+4.4 ppm error), a 65:35 mixture of *N*-Boc piperidine esters **S25** and **S24** (144 mg, 6%) as a colourless oil and and *N*-Boc piperidine ester **S25** (150 mg, 6%) as a colourless oil, R_F (9:1 hexane-EtOAc) 0.17; IR (ATR) 2972, 1735 (C=O, CO_2Me), 1686 (C=O, Boc), 1414, 1152, 865 cm^{-1}; ^1H NMR (400 MHz, $CDCl_3$) δ 4.45-4.29 (m, 2H, NC*H*Me, NCH), 3.67 (s, 3H, OMe), 3.04 (dd, J = 14.0, 4.0 Hz, 1H, NCH), 2.58-2.53 (m, 1H, $CHCO_2$), 2.04-1.96 (m, 1H, CH), 1.92-1.72 (m, 2H, CH), 1.43 (s, 9H, CMe_3), 1.37-1.30 (m, 1H, CH), 1.11 (d, J = 7.0 Hz, 1H, CH*Me*); ^{13}C NMR (100.6 MHz, $CDCl_3$) δ 173.9 (C=O, CO_2Me), 154.9 (C=O, Boc), 77.5 (O*C*Me_3), 51.9 (OMe), 45.9 (N*C*HMe), 39.4 (NCH_2), 39.2 (*C*HCO_2), 28.6 (C*Me_3*), 26.6 (CH_2), 20.2 (CH_2), 16.0 (CH*Me*); HRMS (ESI) *m/z* calcd for $C_{13}H_{23}NO_4$ $(M + Na)^+$ 280.1519, found 280.1519 (–0.3 ppm error).

Lab Book Reference – PJ-02-32.

***tert*-Butyl (2*R**,5*S**)-5-(hydroxymethyl)-2-methylpiperidine-1-carboxylate S26**

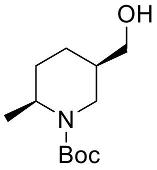

S26

Using general procedure F, $LiAlH_4$ (37 mg, 0.97 mmol, 1.0 eq.) and piperidine ester **S24** (250 mg, 0.97 mmol, 1.0 eq.) in THF (15 mL) gave *N*-Boc piperidine **S26** (218 mg, 98%) as a colourless oil, IR (ATR) 3415 (OH), 2929, 1687 (C=O), 1411, 1142, 867 cm^{-1}; ^{1}H NMR (400 MHz, $CDCl_3$) δ 4.46-4.30 (m, 1H, NC*H*Me), 4.07-3.96 (m, 1H, NCH), 3.58-3.41 (m, 2H, HOC*H*$_2$), 2.56-2.48 (m, 1H, NCH), 1.72-1.48 (m, 4H, CH), 1.46-1.40 (m, 9H, CMe_3), 1.36-1.20 (m, 1H, CH), 1.08 (d, J = 7.0 Hz, 3H, CH*Me*); ^{13}C NMR (100.6 MHz, $CDCl_3$) δ 155.2 (C=O), 79.5 (O*C*Me$_3$), 66.0 (OCH_2), 46.2 (N*C*HMe), 41.5 (NCH_2), 39.3 (CH), 29.7 (CH_2), 28.6 (C*Me*$_3$), 22.0 (CH_2), 15.8 (Me); HRMS (ESI) *m/z* calcd for $C_{12}H_{23}NO_3$ $(M + Na)^+$ 252.1570, found 252.1573 (–1.0 ppm error).

Lab Book Reference – PJ-02-33.

(3*R,6*S**)-6-Methylpiperidin-3-yl]methanol hydrochloride 2r**

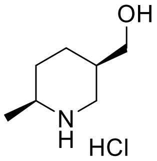

2r

HCl (2.1 mL of a 2 M solution in Et_2O, 4.37 mmol, 5.0 eq.) was added dropwise to piperidine ester **S26** (200 mg, 0.87 mmol, 1.0 eq.) at rt under Ar. Then, the solvent was evaporated under reduced pressure to give piperidine **2r**·HCl (143 mg, 99%) as a colourless oil, IR (ATR) 3360 (NH_2 or OH), 2940, 1450, 1053 cm^{-1}; ^{1}H NMR (400 MHz, MeOH-*d*$_4$) δ 3.68-3.54 (m, 2H, OCH), 3.46-3.37 (m, 1H, NC*H*Me), 3.23-3.12 (m, 2H, NCH), 2.00-1.93 (m, 1H, CH), 1.89-1.80 (m, 1H, CH), 1.77-1.65 (m, 3H, CH), 1.33 (d, J = 7.0 Hz, 3H, CH*Me*); ^{13}C NMR (100.6 MHz, MeOH-*d*$_4$) δ 63.9 (OCH_2), 51.9 (N*C*HMe), 44.2 (NCH_2), 35.5 (CH), 28.0 (CH_2), 23.1 (CH_2), 17.1 (Me); HRMS (ESI) *m/z* calcd for $C_7H_{16}NO$ M^+ 130.1226, found 130.1229 (–1.7 ppm error).

Lab Book Reference – PJ-02-37.

[(3*R,6*S**)-1,6-Dimethylpiperidin-3-yl]methanol 2s**

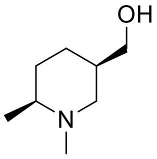

2s

Using general procedure G, *N*-Boc piperidine **S26** (400 mg, 1.56 mmol, 1.0 eq.) in THF (20 mL) and $LiAlH_4$ (295 mg, 7.78 mmol, 5.0 eq.) in THF (30 mL) gave the crude product. Purification by flash column chromatography on silica with 85:14:1 CH_2Cl_2-MeOH-$NH_4OH_{(aq)}$ as eluent gave piperidine **2s** (93 mg, 42%) as a red oil, R_F (85:14:1 CH_2Cl_2-MeOH-$NH_4OH_{(aq)}$) 0.15; IR (ATR) 3333 (OH), 2925, 1446, 1046, 597 cm^{-1}; ^1H NMR (400 MHz, $CDCl_3$) δ 3.84 (dd, *J* = 11.0, 5.0 Hz, 1H, OCH), 3.74 (dd, *J* = 11.0, 5.0 Hz, 1H, OCH), 2.85 (dd, *J* = 11.0, 3.0 Hz, 1H, NCH), 2.37 (dd, *J* = 11.0, 3.0 Hz, 1H, NCH), 2.22 (s, 3H, NMe), 2.13-2.04 (m, 1H, C*H*Me), 1.81-1.74 (m, 1H, CH), 1.69-1.52 (m, 4H, CH), 1.05 (d, *J* = 6.5 Hz, CH*Me*); ^{13}C NMR (100.6 MHz, $CDCl_3$) δ 67.8 (OCH_2), 58.9 (NCH_2), 58.8 (N*C*HMe), 43.3 (NMe), 35.4 (CH), 31.8 (CH_2), 27.4 (CH_2), 18.9 (Me); HRMS (ESI) *m/z* calcd for $C_8H_{17}NO$ $(M + H)^+$ 144.1383, found 144.1379 (+3.1 ppm error).
Lab Book Reference – PJ-02-50.

1-*tert*-Butyl 2-methyl 5-oxopyrrolidine-1,2-dicarboxylate S27

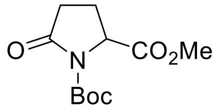

S27

p-Toluenesulfonic acid monohydrate (884 mg, 4.64 mmol, 0.03 eq.) was added a stirred solution of pyroglutamic acid (20.0 g, 155 mmol, 1.0 eq.) in MeOH (300 mL) at rt under Ar. The resulting solution was stirred and heated at reflux for 24 h. After being allowed to cool to rt, the solvent was evaporated under reduced pressure to give the crude methyl ester. Et_3N (23.8 mL, 170 mmol, 1.1 eq.) was added to a stirred solution of the crude methyl ester in CH_2Cl_2 (5 mL) at rt under Ar. Then, a solution of Boc_2O (37.2 g, 170.4 mmol, 1.1 eq.) and DMAP (1.892 g, 170.4 mmol, 1.1 eq.) in CH_2Cl_2 (142 mL) were added dropwise. The resulting mixture was stirred at rt for 23 h. Then, the solvent was evaporated under reduced pressure and the resulting residue was partitioned between saturated $NH_4Cl_{(aq)}$ (80 mL) and EtOAc (40 mL). The two layers were separated and the aqueous layer was extracted with EtOAc (2 × 40 mL). The combined organics were dried ($MgSO_4$) and evaporated under reduced pressure to give the crude product. Purification by flash column chromatography on silica with 80:20 hexane–EtOAc as eluent gave methyl

ester **S27** (28.5 g, 76%) as a pale yellow oil, R_F (1:1 hexane–EtOAc) 0.26; IR (ATR) 2980, 2878, 1789 (C=O, CO_2Me), 1746 (C=O), 1714 (C=O), 1369, 1256, 1146, 843, 729 cm^{-1}; ^1H NMR (400 MHz, $CDCl_3$) δ 4.60 (dd, J = 9.5, 3.0 Hz, 1H, NCH), 3.77 (s, 3H, OMe), 2.61 (ddd, J = 17.5, 10.0, 10.0 Hz, 1H, CH), 2.47 (ddd, J = 13.0, 10.0, 10.0 Hz, 1H, CH), 2.06-1.99 (m, 2H, CH), 1.47 (s, 9H, CMe_3); ^{13}C NMR (100.6 MHz, $CDCl_3$) δ 173.2 (C=O), 171.8 (C=O), 149.2 (C=O, Boc), 83.6 (O*C*Me_3), 58.8 (NCH), 52.5 (OMe), 31.1 (CH_2), 27.8 (C*Me_3*), 21.4 (CH_2); HRMS (ESI) *m/z* calcd for $C_{11}H_{17}NO_5$ $(M + Na)^+$ 266.0999, found 266.0986 (+4.7 ppm error). Spectroscopic data consistent with those reported in the literature.[14]
Lab Book Reference: IC 2-22

Methyl 2-{[(*tert*-butoxy)carbonyl]amino}-5-oxohexanoate 7

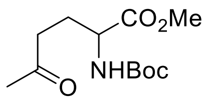

7

MeMgBr (3 M solution in diethyl ether, 4.1 mL, 12.3 mmol, 1.2 eq.) was added dropwise to a stirred solution of **S27** (2.5 g, 10.3 mmol, 1.0 eq.) at −40 °C under Ar. The resulting mixture was stirred at −40 °C for 4 h and then at rt for 16 h. After cooling to 0 °C, saturated $NH_4Cl_{(aq)}$ (10 mL) was added and the mixture was extracted with EtOAc (3 × 20 mL). The combined organics were dried ($MgSO_4$) and evaporated under reduced pressure to give the crude product. Purification by flash column chromatography on silica with 70:30 hexane-EtOAc as eluent gave **7** (1.17 g, 44%) as a colourless oil, R_F (1:1 hexane-EtOAc) 0.7; IR (ATR) 3367 (NH), 2977, 1742 (C=O, CO_2Me), 1707 (C=O, ketone), 1513, 1365, 1160 cm^{-1}; ^1H NMR (400 MHz, $CDCl_3$) δ 5.09 (br d, J = 6.5 Hz, 1H, NH), 4.30-4.25 (br m, 1H, CH), 3.74 (s, 3H, OMe), 2.59 (ddd, J = 18.0, 7.0, 7.0 Hz, 1H, C(O)CH), 2.50 (ddd, J = 18.0, 7.0, 7.0 Hz, 1H, C(O)CH), 2.17-2.08 (m, 1H, CH), 2.15 (s, 3H, C(O)Me), 1.92-1.82 (m, 1H, CH), 1.44 (s, 9H, CMe_3); ^{13}C NMR (100.6 MHz, $CDCl_3$) δ 207.5 (C=O, CO_2Me), 172.9 (C=O, ketone), 155.5 (C=O, Boc), 80.0 (O*C*Me_3), 52.9 (NCH), 52.5 (OMe), 39.4 (*C*H_2CO), 30.1 (C(O)*Me*), 28.3 (C*Me_3*), 26.6 (CH_2); HRMS (ESI) *m/z* calcd for $C_{12}H_{21}NO_5$ $(M + Na)^+$ 282.1312, found 282.1318 (–2.3 ppm error).
Lab book reference MA 2-85

Methyl (2*R,5*R**)-5-methylpyrrolidine-2-carboxylate 8**

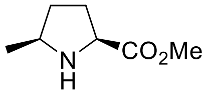

8

TFA (2.95 mL, 38.6 mmol, 10 eq.) was added to a stirred solution of **7** (1.0 g, 3.86 mmol, 1.0 eq.) in CH_2Cl_2 (39 mL). The resulting mixture was stirred at rt for 1 h. Then, the solvent was evaporated under reduced pressure. The residue was dissolved in MeOH (39 mL) and 10% Pd/C (250 mg, 0.14 mmol, 0.04 eq.) was added. Then, the reaction flask evacuated under reduced pressure and back-filled with Ar three times. After a final evacuation, a balloon of H_2 was attached and the reaction mixture was stirred vigorously at rt under H_2 for 16 h. Then, the solids were removed by filtration through Celite® and washed with MeOH (30 mL). The filtrate was evaporated under reduced pressure to give the crude product. Purification by flash column chromatography on silica with 400:9:1 CH_2Cl_2-MeOH-$NH_4OH_{(aq)}$ as eluent gave **8** (552 mg, 99%) as a colourless oil, R_F (100:9:1 CH_2Cl_2-MeOH-$NH_4OH_{(aq)}$) 0.4; IR (ATR) 3345 (NH), 2958, 1732 (C=O), 1435, 1210 cm^{-1}; ^1H NMR (400 MHz, $CDCl_3$) δ 3.76-3.71 (m, 1H, NCH), 3.72 (s, 3H, OMe), 3.18-3.11 (m, 1H, NCH), 2.15-2.05 (m, 2H, CH), 2.02 (br s, 1H, NH), 1.95-1.83 (m, 2H, CH), 1.21 (d, J = 6.0 Hz, 3H, CH*Me*); ^{13}C NMR (100.6 MHz, $CDCl_3$) δ 175.8 (C=O), 60.2 (NCH), 55.5 (NCH), 52.2 (OMe), 33.6 (CH_2), 30.8 (CH_2), 20.8 (Me); HRMS (ESI) *m/z* calcd for $C_7H_{13}NO_2$ $(M + H)^+$ 144.1019, found 144.1016 (+2.0 ppm error).
Lab book reference MA 2-87

Methyl (2*R,5*R**)-1-acetyl-5-methylpyrrolidine-2-carboxylate 1f**

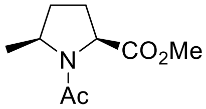

1f

Acetyl chloride (0.14 mL, 2.01 mmol, 3.0 eq.) and Et_3N (0.28 mL, 2.01 mmol, 3.0 eq.) were added to a stirred solution of **8** (150 mg, 0.67 mmol, 1 eq.) in CH_2Cl_2 (3 mL) at rt under Ar. The resulting solution was stirred at rt for 30 min. Saturated $NaHCO_{3(aq)}$ (10 mL) was added and the mixture was extracted with CH_2Cl_2 (3 × 10 mL). The combined organics were dried ($MgSO_4$) and evaporated under reduced pressure to give the crude product. Purification by flash column chromatography on silica with 50:1 CH_2Cl_2-MeOH as eluent gave acetamide **1f** (131 mg, 67%), R_F (100:9:1 CH_2Cl_2-MeOH-$NH_4OH_{(aq)}$) 0.5; IR (ATR) 2954, 1740 (C=O, CO_2Me), 1625 (C=O, C(O)Me), 1411, 1171 cm^{-1}; ^1H NMR (400 MHz, $CDCl_3$) (75:25 mixture of rotamers) δ 4.41 (dd, J = 8.0, 8.0 Hz, 0.75H, NC*H*CO_2Me), 4.36 (dd, J = 8.0, 5.0 Hz,

0.25H, NC*H*CO_2Me), 3.77 (s, 0.75H, OMe), 3.73 (s, 2.25H, OMe), 2.30-2.22 (m, 1H, CH), 2.11-1.99 (m, 4H, CH, NC(O)Me) 1.78-1.73 (m, 1H, CH), 1.66-1.60 (m, 1H, CH), 1.33 (d, J = 6.5 Hz, 2.25H, CH*Me*), 1.30 (d, J = 6.5 Hz, 0.75H, CH*Me*); ^{13}C NMR (100.6 MHz, $CDCl_3$) (rotamers) δ 173.2 (C=O), 173.0 (C=O), 169.6 (C=O), 169.2 (C=O), 60.9 (NCH), 59.4 (NCH), 54.9 (NCH), 54.3 (NCH), 52.6 (OMe), 52.2 (OMe), 32.7 (CH_2), 31.5 (CH_2), 29.7 (CH_2), 27.4 (CH_2), 22.6 (C(O)*Me*), 21.7 (C(O)*Me*), 20.7 (Me), 19.6 (Me); HRMS (ESI) *m/z* calcd for $C_9H_{15}NO_3$ $(M + Na)^+$ 208.0944, found 208.0942 (+1.1 ppm error).

Lab book reference MA 2-96

tert-Butyl-2-cyano-5-oxopentanoate 9

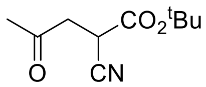

9

t-BuOK (500 mg, 3.54 mmol, 1.0 eq.) was added to a stirred solution of *tert*-butyl cyanoacetate (506 µL, 3.54 mmol, 1.0 eq.) in *t*-BuOH (30 mL) under Ar. The resulting mixture was stirred and heated at 40 °C for 10 min. Then, chloroacetone (2.41 mL, 30.3 mmol, 8.5 eq.) was added dropwise. The resulting solution was stirred and heated at 40 °C for 5 h. Then, saturated $NH_4Cl_{(aq)}$ (20 mL) and EtOAc (20 mL) were added and the two layers were separated. The aqueous layer was extracted with EtOAc (2 × 20 mL). The combined organic layers were dried (Na_2SO_4) and evaporated under reduced pressure to give the crude product. Purification by flash column chromatography on silica with 80:20 hexane-EtOAc as eluent gave keto nitrile **9** (530 mg, 76%) as a brown oil, R_F (50:50 hexane-EtOAc) 0.51; IR (ATR) 2982, 2253 (C≡N), 1740 (C=O, CO_2*t*-Bu), 1722 (C=O, ketone), 1370, 1151 cm^{-1}; ^{1}H NMR (400 MHz, $CDCl_3$) δ 3.87-3.83 (m, 1H, CHCN), 3.14 (dd, J = 18.0, 7.5 Hz, 1H, C*H*C(O)Me), 2.92 (dd, J = 18.0, 5.0 Hz, 1H, C*H*C(O)Me), 2.22 (s, 3H, CHC(O)*Me*), 1.48 (s, 9H, CMe_3); ^{13}C NMR (100.6 MHz, $CDCl_3$) δ 202.7 (C=O, ketone), 164.1 (C=O, CO_2*t*-Bu), 116.6 (C≡N), 84.6 (O*C*Me_3), 42.1 (CH_2), 32.5 (CH), 29.6 (C(O)*Me*), 27.8 (C*Me*$_3$); HRMS (ESI) *m/z* calcd for $C_{10}H_{15}NO_3$ $(M + Na)^+$ 220.0944, found 220.0947 (–1.5 ppm error).

Lab Book Reference: TD 2/91

***tert*-Butyl (3*R**,5*S**)-1-benzyl-5-methylpyrrolidine-3-carboxylate 10**

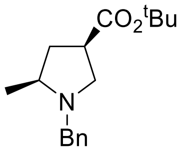

10

PtO_2 (103 mg, 0.45 mmol, 0.05 eq.) was added to a stirred solution of nitrile **9** (1.79 g, 9.05 mmol, 1.0 eq.) in glacial AcOH (40 mL) at rt. The reaction flask was evacuated under reduced pressure and back-filled with Ar three times. Then, the reaction flask was evacuated under reduced pressure and back-filled with H_2 three times. After the final evacuation, H_2 was charged and the reaction mixture was stirred vigorously under a H_2 balloon at rt for 16 h. Then, the solids were removed by filtration through Celite. The solvent was evaporated under reduced pressure to give the crude pyrrolidine. $NaBH(OAc)_3$ (3.84 g, 18.1 mmol, 2.0 eq.) was added to a stirred solution of the crude pyrrolidine, PhCHO (1.02 mL, 9.96 mmol, 1.1 eq.) and AcOH (0.10 mL, 1.81 mmol, 0.2 eq.) in dichloroethane (100 mL) at rt under Ar. The resulting solution was stirred at rt for 16 h. Then, saturated $NaHCO_{3(aq)}$ (100 mL) and CH_2Cl_2 (100 mL) were added and the two layers were separated. The aqueous layer was extracted with CH_2Cl_2 (2 × 100 mL). The combined organics were dried (Na_2SO_4) and evaporated under reduced pressure to give the crude product. Purification by flash column chromatography on silica with 90:10 hexane-EtOAc as eluent gave *N*-benzyl pyrrolidine **10** (756 mg, 30%) as a yellow oil, R_F (90:10 hexane-EtOAc) 0.21; IR (ATR) 2971, 1728 (C=O), 1367, 1150 cm^{-1}; ^1H NMR (400 MHz, $CDCl_3$) δ 7.34-7.18 (m, 5H, Ph), 4.00 (d, J = 13.5 Hz, 1H, NC*H*Ph), 3.16 (d, J = 13.5 Hz, 1H, NC*H*Ph), 3.14 (dd, J = 10.0, 3.5 Hz, 1H, NCH), 2.74 (dddd, J = 9.0, 7.0, 6.5, 3.5 Hz, 1H, $CHCO_2$), 2.48 (ddq, J = 9.0, 9.0, 6.0 Hz, 1H, NC*H*Me), 2.30 (dd, J = 10.0, 9.0 Hz, 1H, NCH), 2.14 (ddd, J = 13.0, 9.0, 6.5 Hz, 1H, CH), 1.77 (ddd, J = 13.0, 9.0, 7.0 Hz, 1H, CH), 1.40 (s, 9H, CMe_3) 1.18 (d, J = 6.0 Hz, 3H, CH*Me*); ^{13}C NMR (100.6 MHz, $CDCl_3$) δ 174.5 (C=O), 139.4 (*ipso*-Ph), 128.7 (Ph), 128.2 (Ph), 126.8 (Ph), 80.2 (O*C*Me_3) 59.4 (N*C*HMe), 57.2 (N*C*H_2Ph), 56.1 (NCH_2), 41.3 (*C*HCO_2), 36.6 (CH_2), 28.1 (C*Me*$_3$), 18.6 (CH*Me*); HRMS (ESI) *m/z* calcd for $C_{17}H_{25}NO_2$ $(M + H)^+$ 276.1958, found 276.1957 (0.2 ppm error).

Lab Book Reference: TD 3/59

(2*R,4*S**)-4-[(*tert*-Butoxy)carbonyl]-2-methylpyrrolidin-1-ium acetate 11b**

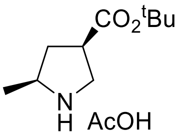

11b

10% $Pd(OH)_2/C$ (10 mg) was added to a stirred solution of pyrrolidine **10** (200 mg, 0.73 mmol, 1.0 eq.) and $NH_4^+HCO_2^-$ (229 mg, 3.63 mmol, 5.0 eq.) in MeOH (10 mL) at rt under Ar. The resulting solution was stirred and heated at 60 °C for 2 h. After being allowed to cool to rt, the solids were removed by filtration through Celite. Glacial AcOH (87 µL, 1.46 mmol, 2.0 eq.) was added dropwise to the filtrate and the resulting mixture was stirred at rt for 30 min. The solvent was evaporated under reduced pressure. Then, the residue was dissolved in CH_2Cl_2 and the solids were removed by filtration. The filtrate was evaporated under reduced pressure to give a 65:35 mixture (by ^1H NMR spectroscopy) of AcOH and pyrrolidine **11b**·AcOH (198 mg,141 mg (79%) of pyrrolidine **11b**·AcOH) as a brown oil, IR (ATR) 2979, 1724 (C=O), 1367, 1153 cm^{-1}; ^1H NMR (400 MHz, $CDCl_3$) for **11b**·AcOH: δ 3.72-3.62 (m, 1H, NC*H*Me), 3.49-3.37 (m, 2H, NCH), 3.13 (dddd, J = 8.5, 8.5, 8.5, 8.5 Hz, 1H, C*H*CO_2), 2.40 (ddd, J = 13.5, 8.5, 6.5 Hz, 1H, CH), 2.03 (s, 3H, $MeCO_2^-$), 1.81 (ddd, J = 13.5, 8.5, 8.5 Hz, 1H, CH), 1.43 (s, 9H, CMe_3), 1.38 (d, J = 6.5 Hz, CH*Me*); ^{13}C NMR (100.6 MHz, $CDCl_3$) **11b**·AcOH: δ 177.0 (C=O, Me*C*O_2^-), 171.3 (C=O, CO_2CMe_3), 82.1 (O*C*Me_3), 55.5 (N*C*HMe), 46.2 (NCH), 43.3 (*C*HCO_2), 36.2 (*C*H_2), 28.0 (C*Me*$_3$), 21.7 (*Me*CO_2^-), 17.5 (CH*Me*); HRMS (ESI) *m/z* calcd for $C_{10}H_{20}NO_2$ M^+ 186.1489, found 186.1491 (–1.6 ppm error).

Lab Book Reference: TD 3/67

(2*R,4*S**)-4-[(*tert*-Butoxy)carbonyl]-1,2-dimethylpyrrolidin-1-ium acetate 11a**

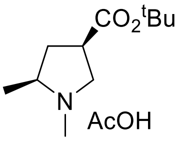

11a

Using general procedure B, $NaBH(OAc)_3$ (1.30 g, 6.12 mmol, 3.0 eq.), pyrrolidine **11b**•AcOH (500 mg, 2.04 mmol, 1.0 eq.), 37% $HCHO_{(aq)}$ (4.52 mL, 61 mmol, 10.0 eq.) and $MgSO_4$ (1.5 g) in 3:1 CH_2Cl_2-AcOH (20 mL) gave the crude product. Glacial AcOH (0.12 mL, 2.04 mmol, 1.0 eq.) was added dropwise to the crude product which was stirred at rt for 30 min. The solvent was evaporated under reduced pressure to give a 65:35 mixture (by ^1H NMR spectroscopy) of AcOH and pyrrolidine **11a**·AcOH (124 mg, 85 mg (16%) of pyrrolidine **11a**·AcOH) as a yellow oil, IR (ATR) 2977, 1726 (C=O), 1368, 1252,

1155 cm^{-1}; ^{1}H NMR (400 MHz, $CDCl_3$) for **11a**·AcOH: δ 3.53 (dd, J = 10.5, 5.0 Hz, 1H, NCH), 3.20-3.01 (m, 3H, NCH, NC*H*Me, $CHCO_2C$), 2.56 (s, 3H, NMe), 2.38 (ddd, J = 13.5, 9.5, 6.5 Hz, 1H, CH), 2.02 (br s, 3H, $MeCO_2^-$), 1.95 (ddd, J = 13.5, 9.0, 7.5 Hz, 1H, CH), 1.43 (s, 9H, CMe_3), 1.27 (d, J = 6.5 Hz, 3H, CH*Me*); ^{13}C NMR (100.6 MHz, $CDCl_3$) **11a**·AcOH: δ 176.4 (C=O, $MeCO_2^-$) 172.0 (C=O, *C*O_2CMe_3), 81.8 (O*C*Me_3), 61.8 (N*C*HMe), 56.0 (NCH), 41.0 (*C*HCO_2), 37.7 (NMe), 35.5 (CH_2), 28.0 (C*Me*$_3$) 21.6 (*Me*CO_2^-), 15.2 (CH*Me*); HRMS (ESI) *m/z* calcd for $C_{11}H_{22}NO_2$ M^+ 200.1646, found 200.1645 (–0.6 ppm error).

Lab Book Reference: TD 3/52

1-*tert*-Butyl 2-methyl (4*E*)-4-[(dimethylamino)methylidene]-5-oxopyrrolidine-1,2-dicarboxylate 12

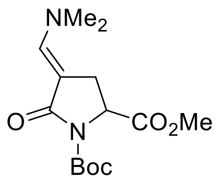

12

tert-Butoxy bis(dimethylamino)methane (575 µL, 2.79 mmol, 1.5 eq.) was added to a stirred solution of methyl ester **S27** (452 mg, 1.86 mmol, 1.0 eq.) in DME (2 mL) at rt under Ar. The resulting solution was heated to reflux and stirred for 20 h. The solvent was then evaporated under reduced pressure. Hexane (200 mL) was added and the solids were removed by filtration. The filtrate was evaporated under reduced pressure to give the enaminone **12** (453 mg, 82%) as yellow solid, mp 127-129 ºC (lit.,[15] mp 124–127 ºC); IR (ATR) 2990, 2956, 2815, 1960, 1757 (C=O), 1740 (C=O), 1677 (C=O), 1607 (C=C), 1440, 1374, 1366, 1308, 1250, 1152, 1110, 1015, 843, 771, 737, 457 cm^{-1}; ^{1}H NMR (400 MHz, $CDCl_3$) δ 7.11 (s, 1H, C=*C*H), 4.60 (dd, J = 11.0, 4.0 Hz, 1H, NCH), 3.73 (s, 3H, OMe), 3.23 (dd, J = 14.0, 11.0 Hz, 1H, CH) 3.00 (s, 6H, NMe_2), 2.87 (dd, J = 14.0, 4.0 Hz, 1H, CH), 1.47 (s, 9H, CMe_3); ^{13}C NMR (100.6 MHz, $CDCl_3$) δ 172.9 (C=O), 169.6 (C=O), 150.6 (C=O, Boc), 146.6 (N*C*H=C), 91.0 (NCH=*C*), 82.4 (O*C*Me_3), 56.1 (NCH), 52.5 (OMe), 42.1 (CH_2), 28.2 (C*Me*$_3$), 26.4 (CH_2); HRMS (ESI) *m/z* calcd for $C_{14}H_{22}N_2O_5$ $(M + Na)^+$ 321.1421, found 321.1413 (+2.0 ppm error). Spectroscopic data consistent with those reported in the literature.[14]

Lab Book Reference: IC/1/31

1-*tert*-Butyl 2-methyl (2*R,4*R**)-4-methyl-5-oxopyrrolidine-1,2-dicarboxylate 13**

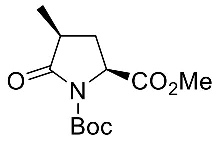

13

10% Pd/C (135 mg, 20 wt%) was added to a stirred solution of enaminone **12** (700 mg, 2.35 mmol, 1.0 eq.) in *i*-PrOH (4 mL) at rt under Ar. Then, the reaction flask was evacuated under reduced pressure and back-filled with Ar three times. After a final evacuation, a balloon of H_2 was attached and the reaction mixture was stirred vigorously at rt under H_2 for 71 h. Then, the solids were removed by filtration through Celite® and washed with *i*-PrOH (20 mL). The filtrate was evaporated under reduced pressure to give the crude product. Purification by flash column chromatography on silica with 80:20 hexane–Et_2O as eluent gave methyl pyroglutamate **13** (423 mg, 74%) as a pale yellow oil, R_F (80:20 hexane–EtOAc) 0.1; IR (ATR) 1759 (C=O, CO_2Me), 1678 (C=O, Boc), 1609 cm^{-1}; ^1H NMR (400 MHz, $CDCl_3$) δ 4.49 (dd, J = 4.5, 4.5 Hz, 1H, NCH), 3.77 (s, 3H, OMe), 2.67-2.49 (m, 2H, CH), 1.64-1.61 (m, 1H, CH), 1.49 (s, 9H, CMe_3), 1.25 (d, J = 7.0 Hz, 1H, CH*Me*); ^{13}C NMR (100.6 MHz, $CDCl_3$) δ 175.8 (C=O), 172.2 (C=O), 149.6 (C=O, Boc), 83.8 (O*C*Me_3), 57.5 (NCH), 52.7 (OMe), 37.7 (N*C*HMe), 29.9 (CH_2), 28.0 (C*Me*$_3$), 16.3 (CH*Me*); HRMS (ESI) *m/z* calcd for $C_{12}H_{19}NO_5$ $(M + Na)^+$ 280.1155, found 280.1145 (+3.2 ppm error). Spectroscopic data consistent with those reported in the literature.[14]

Lab Book Reference: IC 1-56

1-*tert*-Butyl 2-methyl (2*R,4 *R**)-4-methylpyrrolidine-1,2-dicarboxylate S28**

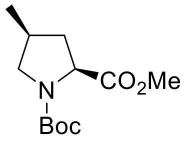

S28

BH_3·DMS (0.49 mL of a 2 M solution in THF, 0.972 mmol, 1.0 eq.) was added dropwise to a stirred solution of methyl pyroglutamate **13** (125 mg, 0.49 mmol, 1.0 eq.) in THF (4 mL) at rt under Ar. The resulting solution was stirred and heated at reflux for 9 h. After being allowed to cool to rt, the solvent was evaporated under reduced pressure. The residue was partitioned between EtOAc (20 mL) and H_2O (20 mL) and the two layers were seperated. The aqueous layer was extracted with EtOAc (3 × 10 mL) and the combined organic layers were dried ($MgSO_4$) and evaporated under reduced pressure to give the crude product. Purification by flash column chromatography on silica with 60:40 hexane–Et_2O as eluent gave methyl ester **S28** (78 mg, 66%) as a pale yellow oil, R_F (1:1 hexane–EtOAc) 0.6; IR (ATR) 2977,

2931, 2851, 1742 (C=O, CO_2Me), 1694 (C=O, Boc), 1393, 1367, 1256, 1169, 994, 906 cm^{-1}; ^1H NMR (400 MHz, $CDCl_3$) (60:40 mixture of rotamers) δ 4.25 (dd, J = 8.0, 8.0 Hz, 0.4H, NCH), 4.19 (dd, J = 9.0, 8.0 Hz, 0.6H, NCH), 3.75-3.64 (m, 1H, NCH), 3.73 (s, 1.2H, OMe), 3.72 (s, 1.8H, OMe), 2.98 (dd, J = 10.0, 10.0 Hz, 1H, NCH), 2.41-2.34 (m, 1H, CH), 2.29-2.15 (m, 1H, CH), 1.61-1.50 (m, 2H, CH), 1.45 (s, 3.6H, CMe_3), 1.40, (s, 5.4H, CMe_3), 1.06 (d, J = 6.5 Hz, 1.8H, CH*Me*), 1.04 (d, J = 6.5 Hz, 1.2H, CH*Me*); ^{13}C NMR (100.6 MHz, $CDCl_3$) (rotamers) δ 173.9 (C=O), 173.7 (C=O), 154.3 (C=O, Boc), 153.6 (C=O, Boc), 79.9 (O*C*Me_3), 79.9 (O*C*Me_3), 59.8 (NCH), 59.3 (NCH), 53.8 (NCH_2), 53.3 (NCH_2), 52.2 (CH), 52.0 (CH), 39.0 (CH_2), 38.1 (CH_2), 33.3 (CH), 32.7 (CH), 28.5 (C*Me*$_3$), 28.3 (C*Me*$_3$), 17.07 (CH*Me*), 16.95 (CH*Me*); HRMS (ESI) *m/z* calcd for $C_{12}H_{21}NO_5$ $(M + Na)^+$ 266.1363, found 266.1370 (–2.6 ppm error).

Lab Book Reference: IC 1-64

Methyl (2*R,4 *R**)-4-methylpyrrolidine-2-carboxylate hydrochloride S29**

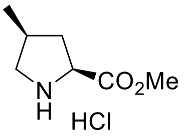

S29

HCl (2.0 M solution in Et_2O, 22.7 mL, 45.3 mmol, 4.4 eq.) was added to a stirred solutiuon of ester **S28** (2.44 g, 10.3 mmol, 1.0 eq.) in Et_2O (20 mL) at rt under Ar. The resulting mixture was stirred and heated at reflux for 17 h. After being allowed to cool to rt, the solvent was evaporated under reduced pressure to give pyrrolidine **S29**·HCl (1.40 g, 99%) as a brown oil which was used without purification, ^1H NMR (400 MHz, MeOH-d_4) δ 4.43 (dd, J = 10.0, 8.0 Hz, 1H, NCH), 3.84 (s, 3H, OMe), 3.48 (dd, J = 11.0, 8.0 Hz, 1H, NCH), 2.88 (dd, J = 11.0, 8.0 Hz, 1H, NCH), 2.61-2.54 (m, 1H, CH) 2.52-2.42 (m, 1H, CH), 1.72-1.65 (m, 1H, CH), 1.12 (d, J = 6.5 Hz, 3H, CH*Me*); ^{13}C NMR (100.6 MHz, MeOH-d_4) δ 170.6 (C=O), 60.8 (NCH), 53.9 (NCH_2), 53.1 (OMe), 37.3 (*C*HMe), 34.4 (CH_2), 16.7 (CH*Me*).

Lab Book Reference: IC 1-75

Methyl (2*R,4*R**)-1-acetyl-4-methylpyrrolidine-2-carboxylate 1g**

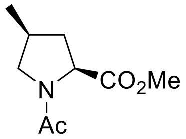

1g

Acetyl chloride (0.24 mL, 3.34 mmol, 3.0 eq.) was added dropwise to a stirred solution of pyrrolidine **S29**·HCl (200 mg, 1.11 mmol, 1.0 eq.) and Et_3N (0.47 mL, 3.34 mmol, 3.0 eq.) in CH_2Cl_2 (1 mL) at rt under Ar. The resulting solution was stirred at rt for 4 h. Water (20 mL) was added and the mixture was extracted with CH_2Cl_2 (3 × 20 mL). The combined organic extracts were dried ($MgSO_4$) and evaporated under reduced pressure to give the crude product. Purification by flash column chromatography with 10:1 CH_2Cl_2–MeOH as eluent gave acetamide **1g** (175 mg, 85%) as a pale yellow oil, R_F (100:9:1 CH_2Cl_2–MeOH–$NH_4OH_{(aq)}$) 0.59; IR (ATR) 2957, 2875, 1740 (C=O, CO_2Me), 1644 (C=O, C(O)Me), 1417, 1197, 1174, 1026, 877, 800, 626, 597, 504 cm^{-1}; ^1H NMR (400 MHz, $CDCl_3$) (80:20 mixture of rotamers) δ 4.35 (dd, J = 8.0, 8.0 Hz, 0.8H, NCH), 4.05 (dd, J = 8.0, 8.0 Hz, 0.2H, NCH), 3.77 (s, 2.4H, OMe), 3.76 (s, 0.6H, OMe), 3.72-3.66 (m, 1H, NCH), 3.18 (dd, J = 8.0, 8.0 Hz, 0.8H, NCH), 2.96 (dd, J = 8.0, 8.0 Hz, 0.2H, NCH), 2.44-2.30 (m, 2H, CH), 2.07 (s, 2.4H, C(O)Me), 1.93 (s, 0.6H, C(O)Me), 1.57-1.51 (m, 0.8H, CH), 1.29-1.25 (m, 0.2H, CH), 1.10 (d, J = 8.0 Hz, 2.4H, CH*Me*), 1.05 (d, J = 8.0 Hz, 0.6H, CH*Me*); ^{13}C NMR (100.6 MHz, $CDCl_3$) (rotamers) δ 173.2 (C=O, CO_2Me), 173.1 (C=O, CO_2Me), 169.7 (C=O, C(O)Me), 169.2 (C=O, C(O)Me), 60.3 (NCH), 59.3 (NCH), 55.0 (OMe), 53.4 (NCH_2), 52.7 (NCH_2), 52.3 (NCH_2), 39.6 (C*H*Me), 37.6 (C*H*Me), 33.9 (CH_2), 31.9 (CH_2), 22.4 (C(O)*Me*), 21.4 (C(O)*Me*), 17.0 (CH*Me*); HRMS (ESI) *m/z* calcd for $C_9H_{15}NO_3$ $(M + Na)^+$ 208.0944, found 208.0942 (–1.1 ppm error). Spectroscopic data consistent with those reported in the literature.[16]

Lab Book Reference: IC 1-86

Methyl 3-[benzyl(2-bromoprop-2-en-1-yl)amino]propanoate 14

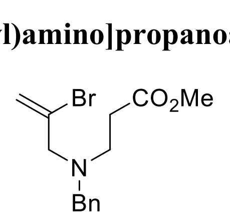

14

Benzylamine (1.09 mL, 10.0 mmol, 1.0 eq.) was added dropwise to a stirred solution of methyl acrylate (900 µL, 10.0 mmol, 1.0 eq.) in MeOH (30 mL) at 0 °C under N_2. The resulting solution was stirred at rt for 16 h. Then, the solvent was evaporated under reduced pressure to give the crude amine. The crude amine was dissolved in MeCN (30 mL) and K_2CO_3 (3.32 g, 24.0 mmol, 2.4 eq.), NaI (1.80 g, 12.0 mmol,

1.2 eq.) and 2,3-dibromopropene (1.47 mL, 12.0 mmol, 1.2 eq.) were added under N_2. The resulting mixture was stirred and heated at 80 °C for 2 h. After being allowed to cool to rt, the solvent was evaporated under reduced pressure. The residue was partitioned between CH_2Cl_2 (50 mL) and saturated $NaHCO_{3(aq)}$ (50 mL) and the two layers were separated. The aqueous layer was extracted with CH_2Cl_2 (3 × 20 mL) and the combined organics were washed with saturated $Na_2S_2O_{3(aq)}$ (50 mL), dried ($MgSO_4$) and evaporated under reduced pressure to give the crude product. Purification by flash column chromatography on silica with 90:10 hexane–Et_2O as eluent gave amine **14** (2.23 g, 71%) as a clear oil, R_F (90:10 hexane–Et_2O) 0.16; IR (ATR) 1737 (C=O), 1629, 1436, 1251, 1196, 1127, 898, 740, 699 cm^{-1}; ^{1}H NMR (400 MHz, $CDCl_3$) δ 7.38–7.17 (m, 5H, Ph), 5.89 (d, J = 1.0 Hz, 1H, C=CH), 5.58 (d, J = 1.0 Hz, 1H, C=CH), 3.65 (s, 5H, OMe and NCH_2), 3.29 (s, 2H, NCH_2), 2.87 (t, J = 7.0 Hz, 2H, NCH_2), 2.50 (t, J = 7.0 Hz, 2H, CH_2CO_2Me); ^{13}C NMR (100.6 MHz, $CDCl_3$) δ 172.8 (C=O), 138.6 (*ipso*-Ph), 131.7 (*C*=CH_2), 128.7 (Ph), 128.3 (Ph), 127.1 (Ph), 118.4 (C=CH_2), 61.9 (NCH_2), 57.7 (NCH_2), 51.6 (OMe), 48.9 (NCH_2), 32.7 (CH_2CO_2Me); HRMS (ESI) *m/z* calcd for $C_{14}H_{18}{}^{79}BrNO_2$ $(M + H)^+$ 312.0594, found 312.0595 (–0.4 ppm error). Spectroscopic data consistent with those reported in the literature.[17]

Lab Book Reference: JDF_B_403

Methyl 1-benzyl-4-methyl-2,5-dihydro-1H-pyrrole-3-carboxylate S30

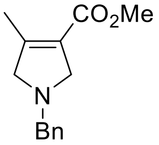

S30

t-BuOK (14.9 mL of a 1 M solution in *t*-BuOH, 14.9 mmol, 2.5 eq.) and $Pd(PPh_3)_4$ (344 mg, 0.30 mmol, 5 mol%) were added sequentially to a stirred solution of vinyl bromide **14** (1.86 g, 5.96 mmol, 1.0 eq.) and phenol (1.68 g, 17.9 mmol, 3.0 eq.) in THF at rt under N_2. The resulting solution was and stirred and heated at reflux for 3 h. After being allowed to cool to rt, EtOAc (50 mL) and saturated $NaHCO_{3(aq)}$ (50 mL) were added and the two layers were separated. The aqueous layer was extracted with EtOAc (3 × 20 mL). The combined organics were dried ($MgSO_4$) and evaporated under reduced pressure to give the crude product. Purification by flash column chromatography on silica with 75:25 hexane-EtOAc as eluent gave dihydropyrrole **S30** (1.04 g, 75%) as an orange oil, R_F (70:30 hexane-EtOAc) 0.19; IR (ATR) 1714 (C=O), 1661, 1436, 1269, 1243, 1158, 1124, 1060, 698 cm^{-1}; ^{1}H NMR (400 MHz, $CDCl_3$) δ 7.36–7.30 (m, 4H, Ph), 7.29–7.23 (m, 1H, Ph), 3.77 (s, 2H, NCH_2Ph), 3.70 (s, 3H, OMe), 3.70–3.67 (m, 2H, NCH_2), 3.57–3.54 (m, 2H, NCH_2), 2.08 (s, 3H, Me); ^{13}C NMR (100.6 MHz, $CDCl_3$) δ 164.8 (C=O),

152.3 (*C*=CCO$_2$Me), 138.9 (*ipso*-Ph), 128.6 (Ph), 128.3 (Ph), 127.1 (Ph), 124.7 (C=*C*CO$_2$Me), 65.7 (NCH$_2$), 60.00 (NCH$_2$), 59.97 (NCH$_2$), 51.0 (OMe), 14.0 (Me); HRMS (ESI) *m/z* calcd for $C_{14}H_{17}NO_2$ $(M + H)^+$ 232.1332, found 232.1331 (+0.7 ppm error). Spectroscopic data consistent with those reported in the literature.[17]

Lab Book Reference: JDF_B_409

Methyl (3*R,4*S**)-4-methylpyrrolidine-3-carboxylate 1h**

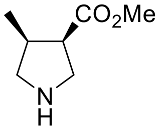

1h

10% Pd/C (74 mg, 10 wt. %) was added to a stirred solution of *N*-benzyl amine **S30** (740 mg, 3.20 mmol, 1.0 eq.) in MeOH (25 mL). The reaction flask evacuated under reduced pressure and back-filled with N_2 three times. After a final evacuation, a balloon of hydrogen was attached, and the reaction mixture was stirred vigorously at rt for 40 h. The solids were removed by filtration through Celite and washed with MeOH (30 mL). The filtrate was evaporated under reduced pressure to give the crude product. Purification with column chromatography on silica with 89:10:1 CH_2Cl_2-MeOH-$NH_4OH_{(aq)}$ as eluent gave pyrrolidine **1h** (260 mg, 57%) as a yellow oil, R_F (89:10:1CH_2Cl_2-MeOH-$NH_4OH_{(aq)}$) 0.1; IR (ATR) 1732 (C=O), 1543, 1414, 1309, 1208, 1173, 811 cm^{-1}; ^{1}H NMR (400 MHz, $CDCl_3$) δ 3.69 (s, 3H, OMe), 3.26 (dd, *J* = 11.5, 5.5 Hz, 1H, NCH), 3.13–3.04 (m, 2H, NCH), 2.99–2.91 (m, 1H, NCH), 2.63 (dd, *J* = 11.0, 7.0 Hz, 1H, C*H*CO$_2$Me), 2.53–2.41 (m, 1H, C*H*Me), 0.97 (d, *J* = 6.5 Hz, 3H, CH*Me*); ^{13}C NMR (100.6 MHz, $CDCl_3$) δ 174.5 (C=O), 54.4 (NCH$_2$), 51.4 (OMe), 49.9 (NCH$_2$), 48.4 (*C*HCO$_2$Me), 37.6 (*C*HMe), 14.7 (CH*Me*); HRMS (ESI) *m/z* calcd for $C_7H_{13}NO_2$ $(M + H)^+$ 144.1019, found 144.1019 (0.0 ppm error).

Lab Book Reference: JDF_B_411

Methyl (3*R,4*S**)-1,4-dimethylpyrrolidine-3-carboxylate 1i**

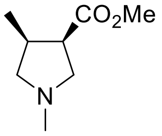

1i

Using general procedure B, NaBH(OAc)$_3$ (318 mg, 1.5 mmol, 1.5 eq.), pyrrolidine **1h** (143 mg, 1.0 mmol, 1.0 eq.), 37% $HCHO_{(aq)}$ (743 µL, 10 mmol, 10 eq.) and $MgSO_4$ (1.0 g) in 4:1 CH_2Cl_2-AcOH (5

mL) gave the crude product. Purification by flash column chromatography on silica with 90:10 CH_2Cl_2-MeOH as eluent gave **1i** (86 mg, 55%) as a colourless oil, R_F (90:10 CH_2Cl_2-MeOH) 0.08; IR (ATR) 2959, 2494, 1999, 1731 (C=O), 1459, 1439, 1381, 1268, 1216 cm^{-1}; ^{1}H NMR (400 MHz, $CDCl_3$) δ 3.68 (s, 3H, OMe), 3.19–3.10 (m, 2H, NCH and C*H*CO_2Me), 3.07 (dd, *J* = 9.0, 7.0 Hz, 1H, NCH), 2.82–2.72 (m, 1H, NCH), 2.71–2.60 (m, 1H, C*H*Me), 2.44 (s, 3H, NMe), 2.13 (t, *J* = 9.0 Hz, 1H, NCH); 0.95 (d, *J* = 7.0 Hz, 3H, CH*Me*); ^{13}C NMR (100.6 MHz, $CDCl_3$) δ 173.8 (C=O), 63.1 (NCH_2), 57.8 (NCH_2), 51.5 (OMe), 46.9 (*C*HCO_2Me), 41.9 (NMe), 35.7 (*C*HMe), 14.6 (CH*Me*); HRMS (ESI) *m/z* calcd for $C_8H_{15}NO_2$ $(M + H)^+$ 158.1176, found 158.1176 (−0.4 ppm error).
Lab book reference JDF_B_412

Methyl 2-methyl-1-(1-phenylethyl)-4,5-dihydro-1H-pyrrole-3-carboxylate 17

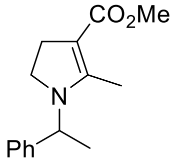

17

Methyl acetoacetate (3.0 mL, 27.8 mmol, 1.0 eq.) was added dropwise to a stirred suspension of 1,2-dibromoethane (4.7 mL, 55.6 mmol, 2.0 eq.) and K_2CO_3 (9.5 g, 69.5 mmol, 2.5 eq.) in MeCN (50 mL) at rt under Ar. The resulting suspension was stirred and heated at reflux for 23 h. After being allowed to cool to rt, the solids were removed by filtration and the filtrate was evaporated under reduced pressure to give the crude product as an orange oil. α-Methylbenzylamine (4.45 mL, 39.0 mmol, 1.5 eq.) was added to a stirred solution of the crude product in toluene (130 mL) at rt under Ar. The resulting solution was stirred and heated at reflux for 48 h. After being allowed to cool to rt, the solvent was evaporated under reduced pressure to give the crude product as an orange oil. Purification by flash column chromatography on silica with 19:1 hexane-EtOAc as eluent gave α-methyl dihydropyrrole **17** (3.04 g, 44%) as an orange oil, R_F (1:1 hexane-EtOAc) 0.29; IR (ATR) 2976, 2942, 1667 (C=O), 1417, 1130, 1025, 699 cm^{-1}; ^{1}H NMR (400 MHz, $CDCl_3$) δ 7.37-7.31 (m, 2H, Ph), 7.28-7.20 (m, 3H, Ph), 4.88 (q, *J* = 7.0 Hz, 1H, NC*H*Me), 3.66 (s, 3H, OMe), 3.46-3.37 (m, 1H, NCH), 3.18-3.11 (m, 1H, NCH), 2.71-2.64 (m, 2H, CH), 2.32 (s, 3H, Me), 1.54 (d, *J* = 7.0 Hz, 3H, CH*Me*); ^{13}C NMR (100.6 MHz, $CDCl_3$) δ 167.9 (C=O), 160.9 (C=*C*Me), 141.2 (*ipso*-Ph), 128.7 (Ph), 127.4 (Ph), 126.4 (Ph), 95.7 (C=*C*CO_2Me), 52.6 (N*C*HMe), 50.2 (OMe), 45.2 (NCH_2), 26.6 (CH_2), 17.6 (Me), 12.2 (CH*Me*); HRMS (ESI) *m/z* calcd for $C_{15}H_{20}NO_2$ $(M + H)^+$ 246.1489, found 246.1497 (–3.2 ppm error).
Lab Book Reference: PJ-01-80.

Methyl (2*R,3*R**)-2-methyl-1-[(1*R**)-1-phenylethyl]pyrrolidine-3-carboxylate 18a, methyl (2*R**,3*R**)-2-methyl-1-[(1*S**)-1-phenylethyl]pyrrolidine-3-carboxylate methyl 18b and (2*R**,3*S**)-2-methyl-1-[(1*R**)-1-phenylethyl]pyrrolidine-3-carboxylate S31**

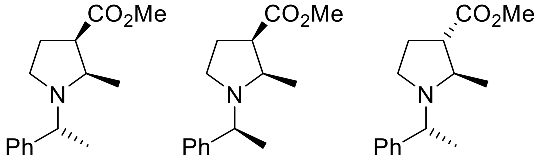

18a **18b** **S31**

NaBH(OAc)$_3$ (1.3 g, 6.12 mmol, 3.0 eq.) was added to a stirred solution of dihydropyrrole **17** (500 mg, 2.04 mmol, 1.0 eq.) in 1:1 AcOH-MeCN (8 mL) at rt under Ar. The resulting solution was cooled and stirred at 0 °C for 3 h. After being allowed to warm to rt, the solvent was evaporated under reduced pressure to give an orange oil. The residue was dissolved in CH_2Cl_2 (50 mL) and saturated $NaHCO_{3(aq)}$ (50 mL) was added. The two layers were separated and the aqueous layer was extracted with CH_2Cl_2 (3 × 50 mL). The combined organics were dried ($MgSO_4$) and evaporated under reduced pressure to give the crude product as a yellow/orange oil which contained a 70:20:10 mixture of **18a**, **18b** and **S31** (by ^{1}H NMR spectroscopy). Purification by flash column chromatography on silica with 7:3 hexane-EtOAc as eluent gave a 90:10 mixture of α-methyl benzyl pyrrolidines **18a** and **18b** (230 mg, 45%) as a yellow oil, R_F (7:3 hexane-EtOAc) 0.22; IR (ATR) 2971, 1736 (C=O), 1452, 1163, 700 cm^{-1}; ^{1}H NMR (400 MHz, $CDCl_3$) δ 7.37-7.19 (m, 5H, Ph), 3.67 (s, 2.7H, OMe), 3.63 (s, 0.3H, OMe), 3.58 (q, J = 6.5 Hz, 1H, PhC*H*Me), 3.53-3.44 (m, 1H, NC*H*Me), 3.15-3.06 (m, 1H, $CHCO_2$), 2.70 (ddd, J = 9.0, 9.0, 4.0 Hz, 1H, NCH), 2.53 (ddd, J = 9.0, 8.0, 8.0 Hz, 1H, NCH), 2.24-2.12 (m, 1H, CH), 1.93-1.82 (m, 1H, CH), 1.36 (d, J = 6.5, 0.3H, PhCH*Me*), 1.35 (d, J = 6.5, 2.7H, PhCH*Me*), 0.81 (d, J = 7.0 Hz, 0.3H, NCH*Me*), 0.75 (d, J = 7.0 Hz, 2.7H, NCH*Me*); ^{13}C NMR (100.6 MHz, $CDCl_3$) for **18a** δ 173.8 (C=O), 145.6 (*ipso*-Ph), 128.3 (Ph), 127.5 (Ph), 127.0 (Ph), 60.9 (Ph*C*HMe), 57.1 (N*C*HMe), 51.6 (OMe), 48.9 (NCH_2), 47.6 (*C*HCO_2), 25.0 (CH_2), 21.1 (PhCH*Me*), 12.7 (CH*Me*); HRMS (ESI) *m/z* calcd for $C_{15}H_{21}NO_2$ $(M + H)^+$ 248.1645, found 248.1647 (–0.3 ppm error) and a 60:25:15 mixture of pyrrolidines **18a**, **18b** and **S31** (162 mg, 18%) as a yellow oil. Diagnostic signals for pyrrolidine **18b**: ^{13}C NMR (100.6 MHz, $CDCl_3$) δ 60.2 (Ph*C*HMe), 56.4 (N*C*HMe), 48.1 (NCH_2), 24.8 (CH_2), 23.3 (PhCH*Me*), 11.9 (CH*Me*). Diagnostic signal for **S31**: ^{1}H NMR (400 MHz, $CDCl_3$) δ 1.00 (d, J = 6.0 Hz, 3H, NCH*Me*). Spectroscopic data consistent with those reported in the literature.[18]

Lab book reference PJ-02-02.

(2*R,3*S**)-2-Methyl-1-[(1*R**)-1-phenylethyl]pyrrolidine-3-carboxylate S32**

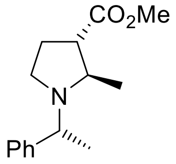

S32

A solution of a 90:10 mixture of pyrrolidines **18a** and **18b** (1.8 g, 7.3 mmol, 1.0 eq.) in DBU (4.3 mL, 29.1 mmol, 4.0 eq.) was stirred and heated at 100 °C for 16 h under Ar. After being allowed to cool to rt, water (50 mL) was added and the aqueous layer was extracted with Et_2O (3 × 50 mL). The combined organics were dried ($MgSO_4$) and evaporated under reduced pressure to give the crude product as an orange oil. Purification by flash column chromatography on silica with 9:1 hexane-EtOAc as eluent gave a 60:30:10 mixture (by ^{1}H NMR spectroscopy) of α-methylbenzyl pyrrolidines **S32**, **18a** and **18b** (530 mg, 30%) and a 95:3:2 mixture (by ^{1}H NMR spectroscopy) of α-methylbenzyl pyrrolidines **S32**, **18a** and **18b** (380 mg, 24%) as an orange oil, R_F (9:1 hexane-EtOAc) 0.1; IR (ATR) 2971, 1731 (C=O), 1452, 1168, 908, 729 cm^{-1}; ^{1}H NMR (400 MHz, $CDCl_3$) for **S32** δ 7.39-7.35 (m, 2H, Ph), 7.33-7.27 (m, 2H, Ph), 7.24-7.20 (m, 1H, Ph), 3.81 (q, J = 6.5 Hz, 1H, PhC*H*Me), 3.70 (s, 3H, OMe), 3.06-2.99 (m, 1H, NC*H*Me), 2.79-2.74 (m, 1H, NCH), 2.61 (ddd, J = 8.0, 8.0, 8.0 Hz, 1H, NCH), 2.56 (ddd, J = 6.0, 6.0, 6.0 Hz, 1H, $CHCO_2$), 2.01-1.94 (m, 2H, CH), 1.34 (d, J = 6.5 Hz, 3H, PhCH*Me*), 1.00 (d, J = 6.0 Hz, 3H, NCH*Me*); ^{13}C NMR (100.6 MHz, $CDCl_3$) for **S32** δ 175.9 (C=O), 145.2 (*ipso*-Ph), 128.2 (Ph), 127.7 (Ph), 126.8 (Ph), 60.4 (N*C*HMe), 58.8 (Ph*C*HMe), 51.9 (OMe), 50.8 (*C*HCO_2), 48.0 (NCH_2), 26.4 (CH_2), 19.7 (PhCH*Me*), 16.3 (CH*Me*); HRMS (ESI) *m/z* calcd for $C_{15}H_{21}NO_2$ (M + H)$^+$ 248.1645, found 248.1640 (+2.0 ppm error).
Lab book reference PJ-02-08.

(2*R,3*R**)-3-(Methoxycarbonyl)-2-methylpyrrolidin-1-ium acetate 1j**

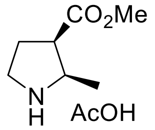

1j

10% Pd/C (149 mg, 0.14 mmol, 0.07 eq.) was added to a stirred solution of a 90:10 mixture of pyrrolidines **18a** and **18b** (500 mg, 2.0 mmol, 1.0 eq.) in MeOH (20 mL) at rt under Ar. The reaction flask was evacuated under reduced pressure and back-filled with Ar three times. After the final evacuation, H_2 was charged and the reaction mixture was stirred vigorously at rt under a H_2 balloon for

3 h. The solids were removed by filtration through Celite and washed with the Et_2O (10 mL). AcOH (0.11 mL, 2.0 mmol, 1.0 eq.) was added dropwise to the filtrate and stirred at rt for 30 min. The solvent was evaporated under reduced pressure to give pyrrolidine **1j**·AcOH (392 mg, 96%) as a yellow oil, IR (ATR) 2955, 1733 (C=O), 1553, 1390, 1009, 654 cm^{-1}; ^1H NMR (400 MHz, MeOH-d_4) δ 3.82 (dq, J = 7.0, 7.0 Hz, 1H, NC*H*Me), 3.70 (s, 3H, OMe), 3.46 (ddd, J = 8.0, 6.0, 6.0 Hz, 1H, $CHCO_2$), 3.35-3.20 (m, 2H, NCH), 2.31-2.18 (m, 2H, CH), 1.91 (s, 3H, $MeCO_2^-$), 1.29 (d, J = 7.0 Hz, 3H, NCH*Me*); ^{13}C NMR (100.6 MHz, MeOH-d_4) δ 178.0 (C=O, $MeCO_2^-$), 173.6 (C=O, CO_2Me), 58.4 (N*C*HMe), 52.6 (OMe), 47.7 (*C*HCO_2), 45.0 (NCH_2), 28.2 (CH_2), 22.5 (*Me*CO_2^-), 14.0 (CH*Me*); HRMS (ESI) *m/z* calcd for $C_7H_{14}NO_2$ M^+ 144.1019, found 144.1023 (–2.7 ppm error).

Lab Book Reference: PJ-02-91.

(2*R,3*R**)-3-(Methoxycarbonyl)-1,2-dimethylpyrrolidin-1-ium acetate 1k**

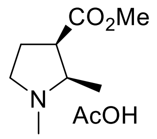

1k

37% aqueous formaldehyde (0.55 mL, 7.4 mmol, 10.0 eq.), was added dropwise to a stirred suspension of pyrrolidine **1j**·AcOH (150 mg, 0.74 mmol, 1.0 eq.), $NaBH(OAc)_3$ (466 mg, 2.2 mmol, 3.0 eq.) and $MgSO_4$ (550 mg, 4.5 mmol) in 4:1 CH_2Cl_2-AcOH (7.4 mL) at rt under Ar. The resulting mixture was stirred at rt for 1 h. Then, the solids were removed by filtration and $NaHCO_{3(aq)}$ was added to the filtrate until pH 9 was reached. The mixture was extracted with CH_2Cl_2 (3 × 20 mL) and the combined organic layers were washed with brine (20 mL) and dried (Na_2SO_4). Glacial AcOH (42 µL, 0.74 mmol, 1.0 eq.) was added dropwise to the filtrate which was stirred at rt for 30 min. The solvent was evaporated under reduced pressure to give *N*-methyl pyrrolidine **1k**·AcOH (91mg, 0.42 mmol, 57%) as an orange oil, IR (ATR) 2917, 1734 (C=O), 1562, 1366, 1256, 2008, 659 cm^{-1}; ^1H NMR (400 MHz, MeOH-d_4) δ 3.65-3.53 (m, 1H, NCH), 3.63 (s, 3H, OMe), 3.47 (dq, J = 7.0, 7.0 Hz, 1H, NC*H*Me), 3.28 (ddd, J = 7.5, 6.0, 6.0 Hz, 1H, $CHCO_2$), 3.05 (ddd, J =8.0, 6.0, 6.0 Hz, 1H, NCH), 2.72 (s, 3H, NMe), 2.23-2.15 (m, 2H, CH), 1.84 (s, 3H, $MeCO_2^-$), 1.21 (d, J = 7.0 Hz, 3H, CH*Me*); ^{13}C NMR (100.6 MHz, MeOH-d_4) δ 176.8 (C=O, $MeCO_2^-$), 173.8 (C=O, CO_2Me), 66.4 (N*C*HMe), 55.9 (NCH_2), 52.7 (OMe), 47.8 (*C*HCO_2), 40.0 (NMe), 26.6 (CH_2), 21.9 (*Me*CO_2^-), 12.35 (CH*Me*); HRMS (ESI) *m/z* calcd for $C_8H_{17}NO_2$ M^+ 158.1176, found 158.1179 (–0.9 ppm error).

Lab Book Reference PJ-02-100.

Methyl (2*R,3*S**)-1-acetyl-2-methylpyrrolidine-3-carboxylate 1l**

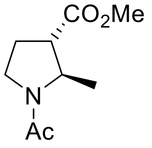

1l

10% Pd/C (106 mg, 1.0 mmol, 0.7 eq.) was added to a stirred solution of a 95:3:2 mixture of pyrrolidines **S32**, **18a** and **18b** (350 mg, 1.3 mmol, 1.0 eq.) in MeOH (7 mL) at rt under Ar. The reaction flask was evacuated under reduced pressure and back-filled with Ar three times. After the final evacuation, H_2 was charged and the reaction mixture was stirred vigorously at rt under a H_2 balloon for 3 h. The solids were removed by filtration through Celite and washed with the MeOH (20 mL). Acetyl chloride (0.49 mL, 5.0 eq.) was added to the filtrate under Ar and the mixture was stirred at rt for 18 h. Then, the solvent was evaporated under reduced pressure to give crude pyrrolidine **1j**·HCl (250 mg). Et_3N (0.8 mL, 5.8 mmol, 3.0 eq.) was added dropwise to a stirred solution of crude pyrrolidine **1j**·HCl (250 mg) in CH_2Cl_2 (30 mL) and the mixture was stirred at rt for 10 min under Ar. Then, acetyl chloride (0.4 mL, 5.8 mmol, 3.0 eq.) was added dropwise and the resulting mixture was stirred at rt for 18 h. The solution was poured into water (50 mL) and the two layers were separated. The organic layer was dried ($MgSO_4$) and evaporated under reduced pressure to give the crude product as an orange oil. Purification by flash column chromatography on silica with 8:2 hexane-EtOAc as eluent gave pyrrolidine acetamide **1l** (140 mg, 42%) as a yellow oil, R_F (8:2 hexane-EtOAc) 0.1; IR (ATR) 2955, 1731 (C=O, CO_2Me), 1634 (C=O, C(O)Me), 1412, 1171, 669 cm^{-1}; ^{1}H NMR (400 MHz, $CDCl_3$) (65:35 mixture of rotamers) δ 4.40 (qd, J = 6.5, 2.5 Hz, 0.65H, NC*H*Me), 4.25 (dq, J = 6.5, 2.5 Hz, 0.35H, NC*H*Me), 3.70 (s, 1.05H, OMe), 3.68 (s, 1.95H, OMe), 3.63-3.54 (m, 1H, NCH), 3.53-3.46 (m, 0.65H, NCH), 3.45-3.38 (m, 0.35H, NCH), 2.77 (ddd, J = 6.0, 6.0, 3.0 Hz, 0.65H, $CHCO_2$), 2.72-2.65 (m, 0.35H, $CHCO_2$), 2.26-2.14 (m, 2H, CH), 2.09 (s, 1.05H, MeCO), 2.01 (s, 1.95H, MeCO), 1.31-1.25 (s, 3H, NCH*Me*); ^{13}C NMR (100.6 MHz, $CDCl_3$) (rotamers) δ 173.9 (C=O), 169.1 (C=O), 56.1 (N*C*HMe), 52.2 (OMe), 49.4 (*C*HCO_2), 46.7 (NCH_2), 26.9 (CH_2), 23.0 (C(O)*Me*), 20.2 (CH*Me*); HRMS (ESI) *m/z* calcd for $C_9H_{15}NO_3$ $(M + Na)^+$ 208.0944, found 208.0951 (–3.5 ppm error).

Lab book reference PJ-02-11/13.

Methyl (2*R,3*R**)-1-acetyl-2-methylpiperidine-3-carboxylate 19a**

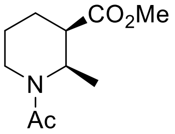

19a

Using general procedure C, Ac_2O (7.2 mL, 76 mmol, 6.0 eq.) and piperidine **6a** (2.00 g, 12.7 mmol, 1.0 eq.) in pyridine (12 mL) gave the crude product. Purification by flash column chromatography on silica with EtOAc as eluent gave *N*-acyl piperidine **19a** (2.11 g, 83%) as a pale yellow oil, R_F (EtOAc) 0.24; IR (ATR) 2950, 1731 (C=O, CO_2Me), 1634 (C=O, C(O)Me), 1421, 1161, 1018 cm^{-1}; ^1H NMR (400 MHz, $CDCl_3$) (60:40 mixture of rotamers) δ 5.20 (dq, J = 7.0, 7.0 Hz, 0.6H, NC*H*Me), 4.42 (br dd, J = 13.5, 4.0 Hz, 0.4H, NCH), 4.33 (dq, J = 7.0, 7.0 Hz, 0.4H, NC*H*Me), 3.64 (s, 1.2H, OMe), 3.60 (s, 1.8, OMe), 3.52 (br dd, J = 13.5, 4.5 Hz, 0.6H, NCH), 3.04 (ddd, J = 13.5, 13.5, 3.0 Hz, 0.6H, NCH), 2.632.42 (m, 1.4H, CH), 2.07 (s, 1.2H, C(O)Me), 2.00 (s, 1.8H, C(O)Me), 1.91-1.60 (m, 3H, CH), 1.451.20 (m, 1H, CH), 1.06 (d, J = 7.0 Hz, 1.2H, NCH*Me*), 0.95 (d, J = 7.0 Hz, 1.8H, NCH*Me*); ^{13}C NMR (100.6 MHz, $CDCl_3$) (rotamers) δ 173.1 (C=O), 172.7 (C=O), 168.9 (C=O), 51.9 (OMe), 51.7 (OMe), 50.4 (CH), 45.7 (CH), 44.5 (CH), 44.5 (CH), 40.9 (NCH_2), 35.3 (NCH_2), 25.2 (CH_2), 24.3 (CH_2), 22.1 (C(O)*Me*), 21.5 (C(O)*Me*), 20.5 (CH_2), 20.4 (CH_2), 13.0 (CH*Me*), 12.0 (CH*Me*); HRMS (ESI) *m/z* calcd for $C_{10}H_{17}NO_3$ $(M + Na)^+$ 222.1101, found 222.1096 (+2.0 ppm error).
Lab book reference HFK2-034

Methyl (2*R,3*R**)-1-methanesulfonyl-2-methylpiperidine-3-carboxylate 19b**

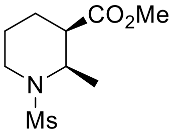

19b

Using general procedure D, Et_3N (1 mL, 7.5 mmol, 1.0 eq.), piperidine **6a** (1.18 g, 7.5 mmol, 1.0 eq.) and MsCl (1.8 mL, 23 mmol, 3.0 eq.) in CH_2Cl_2 (20 mL) gave the crude product as a white solid. Purification by flash column chromatography on silica with 6:1-1:1 hexane-EtOAc as eluent gave sulfonamide **19b** (1.60 g, 88%) as a white solid, mp 114-117 °C; R_F (1:1 hexane-EtOAc) 0.43; IR (ATR) 3005, 2961, 1731 (C=O), 1315, 1134, 784 cm^{-1}; ^1H NMR (400 MHz, $CDCl_3$) δ 4.55-4.49 (m, 1H, NC*H*Me), 3.66 (s, 3H, OMe), 3.63 (br dd, J = 13.0, 5.0 Hz, 1H, NCH), 2.93 (ddd, J = 13.0, 13.0, 2.5 Hz, 1H, NCH), 2.84 (s, 3H, SO_2Me), 2.71 (ddd, J = 12.5, 4.5, 4.5 Hz, 1H, C*H*CO_2Me), 1.95-1.83 (m, 1H, CH), 1.81-1.63 (m, 2H, CH), 1.60-1.41 (m, 1H, CH), 1.11 (d, J = 7.0 Hz, 3H, NCH*Me*); ^{13}C NMR (100.6

MHz, $CDCl_3$) δ 172.6 (C=O), 51.9 (OMe), 49.4 (N*C*HMe), 45.5 (*C*HCO$_2$Me), 40.2 (SO$_2$Me), 39.4 (NCH$_2$), 24.9 (CH$_2$), 20.1 (CH$_2$), 12.3 (CH*Me*); HRMS (ESI) *m/z* calcd for $C_9H_{17}NO_4S$ (M + Na)$^+$ 258.0770, found 258.0766 (+1.3 ppm error).
Lab book reference HFK 2-032

1-*tert*-Butyl 3-methyl (2*R,3*R**)-2-methylpiperidine-1,3-dicarboxylate S33 and 1-*tert*-butyl 3-methyl (2*R**,3*S**)-2-methylpiperidine-1,3-dicarboxylate S34**

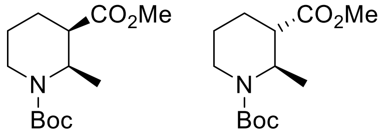

S33 **S34**

Et_3N (432 µL, 7.00 mmol, 1.0 eq) and a solution of Boc$_2$O (1.58 g, 7.24 mmol, 1.1 eq) in CH_2Cl_2 (6 mL) were added sequentially to a stirred solution of a 90:10 mixture of piperidine **6a** and its *trans* diastereomer (1.10 g, 7.00 mmol, 1.0 eq) in CH_2Cl_2 (18 mL) at 0 °C under Ar. The resulting solution was stirred at rt for 18 h. The solvent was evaporated under reduced pressure. The residue was dissolved in Et_2O (25 mL) and the solution was washed with 1 M $HCl_{(aq)}$ (2 × 20 mL) and saturated $NaHCO_{3(aq)}$ (20 mL), dried ($MgSO_4$) and evaporated under reduced pressure to give the crude product as a clear oil. Purification by flash column chromatography on silica with 95:5-90:10 hexane-EtOAc as eluent gave methyl ester **S33** (1.41 g, 78%) as a white solid, mp 49-51 °C (lit.,[19] 37 °C); R_F (4:1 hexane-EtOAc) 0.42; IR (ATR) 2974, 2950, 1736 (C=O, CO$_2$Me), 1687 (C=O, Boc), 1408, 1132, 856 cm^{-1}; ^{1}H NMR (400 MHz, $CDCl_3$) (50:50 mixture of rotamers) δ 4.78 (br s, 0.5H, N*C*HMe), 4.60 (br s, 0.5H, N*C*HMe), 3.95 (br d, *J* = 12.0 Hz, 0.5H, NCH), 3.82 (br d, *J* = 12.0 Hz, 0.5H, NCH), 3.65 (s, 1.5H, OMe), 3.63 (s, 1.5H, OMe), 2.84-2.64 (m, 1H, CH), 2.58 (ddd, *J* = 13.0, 4.0, 4.0 Hz, 1H, C*H*CO$_2$Me), 1.86-1.57 (m, 3H, CH), 1.47-1.36 (m, 10H, CMe$_3$ and CH), 0.98 (d, *J* = 7.0 Hz, 3H, NCH*Me*); ^{13}C NMR (100.6 MHz, $CDCl_3$) (rotamers) δ 173.4 (C=O, CO$_2$Me), 154.6 (C=O, Boc), 79.7 (O*C*Me$_3$), 51.8 (OMe), 51.7 (OMe), 48.0 (N*C*HMe), 46.8 (N*C*HMe), 45.2 (*C*HCO$_2$Me), 44.8 (*C*HCO$_2$Me), 38.6 (NCH$_2$), 37.5 (NCH$_2$), 28.5 (C*Me*$_3$), 24.9 (CH$_2$), 24.5 (CH$_2$), 20.5 (CH$_2$), 12.3 (NCH*Me*), 12.0 (NCH*Me*); HRMS *m/z* calcd for $C_{13}H_{23}NO_4$ (M + Na)$^+$ 280.1519, found 280.1525 (+1.9 ppm error) and **S34** (83 mg, 4%) as a yellow oil, R_F (4:1 hexane-EtOAc) 0.35; IR (ATR) 2973, 1735 (C=O, CO$_2$Me), 1686 (C=O, Boc), 1416, 1174, 862 cm^{-1}; ^{1}H NMR (400 MHz, $CDCl_3$) 4.81 (q, *J* = 7.0 Hz, 1H, N*C*HMe), 3.88 (br dd, *J* = 13.0, 4.0 Hz, 1H, NCH), 3.63 (s, 3H, OMe), 2.75 (ddd, *J* = 13.0, 13.0, 3.0 Hz, 1H, NCH), 2.39-2.29 (m, 1H, C*H*CO$_2$Me), 1.98 (br dd, *J* = 13.0, 3.0 Hz, 1H, CH), 1.77-1.65 (m, 1H, CH), 1.65-1.51 (m, 1H, CH), 1.45-1.37 (m, 10H, CMe$_3$ and

CH), 1.16 (d, J = 7.0 Hz, 3H, NCH*Me*); ^{13}C NMR (100.6 MHz, $CDCl_3$) δ 173.9 (C=O, CO_2Me), 154.9 (C=O, Boc), 79.2 (O*C*Me_3), 51.8 (OMe), 47.3 (N*C*HMe), 44.0 (*C*HCO_2Me), 37.8 (NCH_2), 28.4 (C*Me_3*), 21.8 (CH_2), 20.5 (CH_2), 16.5 (Me); HRMS m/z calcd for $C_{13}H_{23}NO_4$ $(M + Na)^+$ 280.1519, found 280.1522 (–1.0 ppm error). Spectroscopic data consistent with those reported in the literature for **S33**.[19]
Lab book reference HFK1-096

(2*R,3*R**)-1-[(*tert*-Butoxy)carbonyl]-2-methylpiperidine-3-carboxylic acid S35**

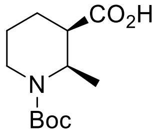

S35

A solution of LiOH (303 mg, 12.7 mmol, 5.0 eq.) in H_2O (12 mL) was added dropwise to a stirred solution of ester **S33** (652 mg, 2.53 mmol, 1.0 eq.) in MeOH (12 mL) at rt under Ar. The resulting solution was stirred at rt for 2 h. Then, the solvent was evaporated under reduced pressure. H_2O (15 mL) was added and the mixture acidified to pH 2 with 12 M $HCl_{(aq)}$ (1 mL). EtOAc (20 mL) was added and the two layers were separated. The aqueous layer was extracted with EtOAc (3 × 15 mL) and the combined organics were dried ($MgSO_4$) and evaporated under reduced pressure to give acid **S35** (462 mg, 75%) as a white solid, mp 167-169 °C (lit.,[20] 178 °C); R_F (1:1 hexane-EtOAC) 0.26; IR (ATR) 2975, 1733 (C=O, CO_2H), 1691 (C=O, Boc), 1413, 1134, 732 cm^{-1}; ^{1}H NMR (400 MHz, $CDCl_3$) δ 4.96-4.56 (m, 1H, NC*H*Me), 4.06-3.81 (m, 1H, NCH), 2.86-2.70 (m, 1H NCH), 2.65 (ddd, J = 13.0, 4.0, 4.0 Hz, 1H, C*H*CO_2H), 1.91-1.78 (m, 1H, CH), 1.78-1.56 (m, 2H, CH), 1.44-1.37 (m, 10H, CMe_3 and CH), 1.08 (d, J = 7.0 Hz, 3H, CH*Me*); ^{13}C NMR (100.6 MHz, $CDCl_3$) (rotamers) δ 178.4 (C=O, CO_2H), 154.8 (C=O, Boc), 80.0 (O*C*Me_3), 47.9 (N*C*HMe), 46.6 (N*C*HMe), 45.1 (*C*HCO_2H), 38.5 (NCH_2), 37.6 (NCH_2), 28.6 (C*Me_3*), 24.7 (CH_2), 20.4 (CH_2), 12.2 (Me); HRMS (ESI) *m/z* calcd for $C_{12}H_{21}NO_4$ $(M + Na)^+$ 266.1363, found 266.1365 (–1.0 ppm error). Spectroscopic data consistent with those reported in the literature.[20]
Lab book reference HFK3-032

***tert*-Butyl (2*R**,3*R**)-3-carbamoyl-2-methylpiperidine-1-carboxylate S36**

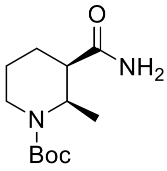

S36

Using general procedure J, DIPEA (917 µL, 5.26 mmol, 3.0 eq.), T3P (1.57 mL of a 50% wt solution in EtOAc, 5.27 mmol, 3.0 eq.), acid **S35** (427 mg, 1.75 mmol, 1.0 eq.) in CH_2Cl_2 (40 mL) and 35% $NH_4OH_{(aq)}$ (2.64 mL, 8.77 mmol, 5.0 eq.) gave the crude product. Purification by flash column chromatography on silica with 13:1 hexane-CH_2Cl_2 as eluent gave amide **S36** (332 mg, 78%) as a white solid, mp 127-129 °C; R_F (9:1 CH_2Cl_2-MeOH) 0.36; IR (ATR) 3334 (NH), 2976, 1660 (C=O), 1404, 1157, 730 cm^{-1}; ^1H NMR (400 MHz, $CDCl_3$) δ 5.86 (br s, 2H, NH_2), 4.69-4.52 (m, 1H, NC*H*Me), 3.93-3.83 (m, 1H, NCH), 2.77 (ddd, J = 13.0, 3.0 Hz, 1H, NCH), 2.48 (ddd, J = 12.0, 4.0, 4.0 Hz, 1H, C*H*$CONH_2$), 1.88-1.61 (m, 3H, CH), 1.43 (s, 9H, CMe_3), 1.43-1.33 (m, 1H, CH), 1.07 (d, J = 7.0 Hz, 3H, NCH*Me*); ^{13}C NMR (100.6 MHz, $CDCl_3$) δ 175.1 (C=O, $CONH_2$), 154.9 (C=O, Boc), 79.9 (O*C*Me_3), 48.3 (N*C*HMe), 45.9 (*C*H$CONH_2$), 38.3 (NCH_2), 28.6 (C*Me*$_3$), 24.8 (CH_2), 20.6 (CH_2), 11.9 (Me); HRMS (ESI) *m/z* calcd for $C_{12}H_{22}N_2O_3$ $(M + Na)^+$ 265.1523, found 265.1526 (–1.2 ppm error).
Lab book reference HFK3-034

***tert*-Butyl (2*R**,3*R**)-3-cyano-2-methylpiperidine-1-carboxylate S37**

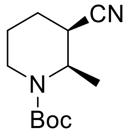

S37

Using general procedure K, Et_3N (249 µL, 1.78 mmol, 2.4 eq.), trifluoroacetic anhydride (126 µL, 0.89 mmol, 1.2 eq.) and piperidine amide **S36** (180 mg, 0.74 mmol, 1.0 eq.) in THF (18 mL) gave the crude product. Purification by flash column chromatography on silica with 5:1 hexane-EtOAc as eluent gave piperidine nitrile **S37** (134 mg, 80%) as a clear oil, R_F (4:1 hexane-EtOAc) 0.37; IR (ATR) 2977, 1690 (C=O), 1409, 1160, 1139 cm^{-1}; ^1H NMR (400 MHz, $CDCl_3$) δ 4.72-4.51 (m, 1H, NC*H*Me), 3.94-3.90 (m, 1H, NCH), 2.84-2.69 (m, 2H, NCH and CHCN), 1.99-1.93 (m, 1H, CH), 1.82 (dddd, J = 13.0, 13.0, 13.0, 4.0 Hz, 1H, CH), 1.70-1.65 (m, 1H, CH), 1.43 (s, 9H, CMe_3), 1.41-1.31 (m, 1H, CH), 1.28 (d, J = 7.0 Hz, 3H, CH*Me*); ^{13}C NMR (100.6 MHz, $CDCl_3$) δ 154.3 (C=O, Boc), 120.5 (CN), 80.4 (O*C*Me_3), 46.7 (N*C*HMe), 37.4 (NCH_2), 31.6 (*C*HCN), 28.5 (C*Me*$_3$), 24.4 (CH_2), 22.9 (CH_2), 12.3 (Me); HRMS (ESI) *m/z* calcd for $C_{12}H_{20}N_2O_2$ $(M + Na)^+$ 247.1417, found 247.1421 (–1.4 ppm error).

Lab book reference HFK3-036

(2*R,3*R**)-2-Methylpiperidine-3-carbonitrile hydrochloride 19c**

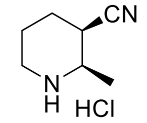

19c

Boc protected nitrile **S37** (78 mg, 0.35 mmol, 1.0 eq.) was dissolved in HCl (5.0 mL of a 2 M solution in Et_2O, 10 mmol, 29 eq.) and the resulting solution was stirred at rt for 36 h. Then, the solvent was evaporated under reduced pressure to give piperidine hydrochoride **19c**·HCl (48 mg, quant.) as a white solid, mp 188-190 °C; R_F (1:1 hexane-EtOAc) 0.75; IR (ATR) 2921, 2703, 2243 (CN), 1588, 1446, 1021 cm^{-1}; ^{1}H NMR (400 MHz, MeOH-*d*$_4$) 3.52 (m, 1H, NC*H*Me), 3.46 (m, 1H, NCH), 3.40-3.31 (m, 1H, NCH), 3.12-3.00 (m, 1H, CHCN), 2.12 (m, 1H, CH), 2.01-1.80 (m, 3H, CH), 1.47 (d, *J* = 7.0 Hz, 3H, CH*Me*); ^{13}C NMR (100.6 MHz, MeOH-*d*$_4$) 118.7 (CN), 52.4 (N*C*HMe), 44.8 (NCH_2), 32.7 (*C*HCN), 26.0 (CH_2), 20.0 (CH_2), 17.5 (Me); HRMS (ESI) *m/z* calcd for $C_7H_{13}N_2$ M^+ 125.1073, found 125.1073 (+0.4 ppm error).

Lab book reference HFK3-042

(2*R,3*R**)-1-Methanesulfonyl-2-methylpiperidine-3-carboxylic acid 19k**

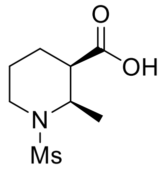

19k

A solution of LiOH (458 mg, 19.1 mmol, 5.0 eq.) in H_2O (15 mL) was added dropwise to a stirred solution of ester **19b** (900 mg, 3.82 mmol, 1.0 eq.) in MeOH (15 mL) at rt under Ar. The resulting solution was stirred and heated at 40 °C for 2 h. Then, the mixture was allowed to cool to rt and the solvent was evaporated under reduced pressure. H_2O (10 mL) was added and the mixture acidified to pH 2 with 12 M $HCl_{(aq)}$ (1 mL). Then, EtOAc (15 mL) was added and the two layers were separated. The aqueous layer was extracted with EtOAc (3 × 15 mL) and the combined organics were dried ($MgSO_4$) and evaporated under reduced pressure to give acid **19k** (789 mg, 93%) as a white solid, mp 116-119 °C; R_F (9:1 CH_2Cl_2-MeOH) 0.35; IR (ATR) 3212 (OH), 2978, 2956, 1732 (C=O), 1306, 1120 cm^{-1}; ^{1}H NMR (400 MHz, $CDCl_3$) δ 4.55-4.49 (m, 1H, NC*H*Me), 3.65 (br dd, *J* = 13.0, 4.0 Hz, 1H, NCH), 2.95 (ddd, *J* = 13.0, 13.0, 2.5 Hz, 1H, NCH), 2.86 (s, 3H, SO_2Me), 2.77 (ddd, *J* = 13.0, 4.0, 4.0 Hz, 1H, C*H*CO_2H),

1.99-1.87 (m, 1H, CH), 1.84-1.64 (m, 2H, CH), 1.62-1.43 (m, 1H, CH), 1.19 (d, 7.0 Hz, 3H, CH*Me*); ^{13}C NMR (100.6 MHz, $CDCl_3$) δ 178.0 (C=O), 49.2 (N*C*HMe), 45.5 (SO_2Me), 40.3 (*C*HCO_2H), 39.3 (NCH_2), 24.9 (CH_2), 19.9 (CH_2), 12.3 (NCH*Me*); HRMS (ESI) *m/z* calcd for $C_8H_{15}NO_4S$ $(M + Na)^+$ 244.0614, found 244.0614 (–0.7 ppm error).

Lab book reference HFK 2-037

Structure confirmed by small molecule X-Ray crystallography (CCDC 1995344):

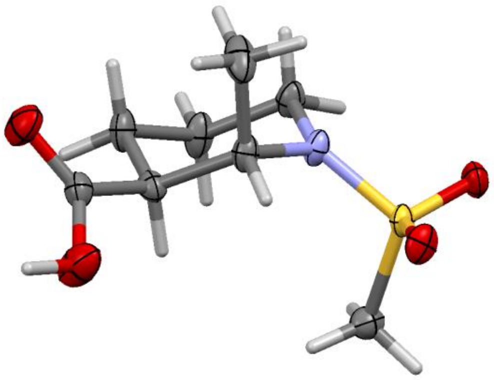

(2*R,3*R**)-1-Methanesulfonyl-2-methylpiperidine-3-carboxamide 19h**

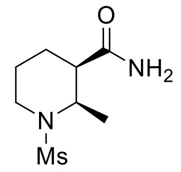

19h

Using general procedure J, DIPEA (132 μL, 0.76 mmol, 3.0 eq.), T3P (112 μL of a 50% wt solution in EtOAc, 0.38 mmol, 1.5 eq.), acid **19k** (56 mg, 0.25 mmol, 1.0 eq.) in CH_2Cl_2 (3 mL) and 35% $NH_{3(aq)}$ (706 μL, 0.28 mmol, 1.1 eq.) gave amide **19h** (32 mg, 57%) as a white solid, mp 164-168 °C; R_F (9:1 CH_2Cl_2-MeOH) 0.39; IR (ATR) 3436 (NH), 3401 (NH), 2967, 2871, 1617 (C=O), 1134, 775 cm^{-1}; ^{1}H NMR (400 MHz, MeOH-*d*$_4$) δ 4.41-4.34 (m, 1H, NC*H*Me), 3.60 (br dd, *J* = 13.0, 4.0 Hz, 1H, NCH), 3.01 (ddd, *J* = 13.0, 13.0, 2.5 Hz, 1H, NCH), 2.92 (s, 3H, SO_2Me), 2.62 (ddd, *J* = 12.5, 4.5, 4.5 Hz, 1H, C*H*$CONH_2$), 1.89-1.67 (m, 3H, CH), 1.60-1.41 (m, 1H, CH), 1.16 (d, *J* = 7.0 Hz, 3H, CH*Me*); ^{13}C NMR (100.6 MHz, MeOH-*d*$_4$) δ 177.6 (C=O), 51.6 (N*C*HMe), 47.4 (*C*$HCONH_2$), 40.4 (NCH_2), 40.3 (SO_2Me), 25.8 (CH_2), 21.2 (CH_2), 12.2 (CH*Me*); HRMS (ESI) *m/z* calcd for $C_8H_{16}N_2O_3S$ $(M + Na)^+$ 243.0774, found 243.0771 (+3.5 ppm error).

Lab book reference HFK 2-038

(2*R,3*R**)-1-Methanesulfonyl-2-methylpiperidine-3-carbonitrile 19d**

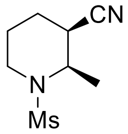

19d

Using general procedure K, Et_3N (88 µL, 0.63 mmol, 2.4 eq.), trifluoroacetic anhydride (45 µL, 0.32 mmol, 1.2 eq.) and amide **19h** (58 mg, 0.26 mmol, 1.0 eq.) in THF (7 mL) gave the crude product. Purification by flash column chromatography on silica with hexane and then 1:1 hexane-EtOAc as eluent gave piperidine nitrile **19d** (45 mg, 85%) as a cream solid, mp 108-111 °C; R_F (7:3 hexane-EtOAc) 0.48; IR (ATR) 2950, 2240 (C≡N), 1321, 1138, 769, 1134, 775 cm^{-1}; ^1H NMR (400 MHz, $CDCl_3$) δ 4.44-4.38 (m, 1H, NC*H*Me), 3.63 (br dd, *J* = 13.0, 4.0 Hz, 1H, NCH), 2.97 (ddd, *J* = 13.0, 13.0, 3.0 Hz, 1H, NCH), 2.91 (ddd, *J* = 13.0, 4.5, 4.5 Hz, 1H, CHCN), 2.84 (s, 3H, SO_2Me), 2.11-1.97 (m, 1H, CH), 1.85 (dddd, *J* = 13.0, 13.0, 13.0, 4.0 Hz, 1H, CH), 1.79-1.71 (m, 1H, CH), 1.54 (ddddd, *J* = 13.0, 13.0, 13.0, 4.0, 4.0 Hz, 1H, CH), 1.38 (d, *J* = 7.0 Hz, 3H, CH*Me*); ^{13}C NMR (100.6 MHz, $CDCl_3$) δ 119.8 (CN), 48.7 (N*C*HMe), 40.3 (SO_2Me), 38.8 (NCH_2), 32.2 (*C*HCN), 24.5 (CH_2), 22.5 (CH_2), 12.4 (CH*Me*); HRMS (ESI) *m/z* calcd for $C_8H_{14}N_2O_2S$ $(M + Na)^+$ 225.0668, found 225.0657 (+4.5 ppm error).

Lab book reference HFK 2-047

Structure confirmed by small molecule X-Ray crystallography (CCDC 1995343):

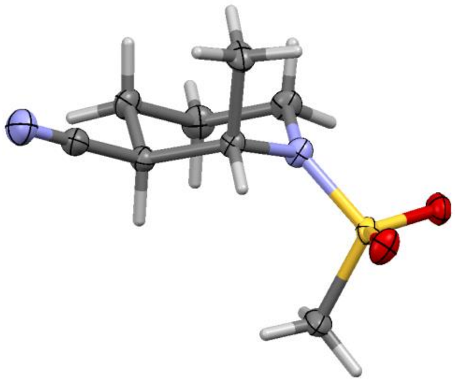

1-[(2*R,3*R**)-3-(Hydroxymethyl)-2-methylpiperidin-1-yl]ethan-1-one 19e**

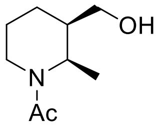

19e

$LiBH_4$ (0.58 mL of a 4 M solution in THF, 2.3 mmol, 2.3 eq.) was added dropwise to a stirred solution of ester **19a** (200 mg, 1.00 mmol, 1.0 eq.) in THF (6 mL) at 0 °C under Ar. The resulting solution was stirred at rt for 18 h. Then, the reaction was cooled to 0 °C and $LiBH_4$ (0.50 mL of a 4 M solution in THF, 2.0 mmol, 2.0 eq.) was added. The resulting solution was stirred at rt for 6 h. Saturated NH_4Cl_{aq} (10 mL) and EtOAc (10 mL) were added and the two layers were sperated. The aqueous layer was extracted with EtOAc (3 × 10 mL) and the combined organic layers were dried (Mg_2SO_4) and evaporated under reduced pressure to give alcohol **19e** (110 mg, 64%) as a pale yellow oil, R_F (9:1 CH_2Cl_2-MeOH) 0.42; IR (ATR) 3377 (OH), 2931, 2863, 1611 (C=O), 1426, 727 cm^{-1}; ^1H NMR (400 MHz, $CDCl_3$) (50:50 mixture of rotamers) δ 4.83 (m, 0.5H, NC*H*Me), 4.39-4.29 (m, 0.5H, NCH), 4.16-4.02 (m, 1H, NC*H*Me and OH), 3.72 (s, 0.5H, OH), 3.52-3.44 (m, 0.5H, NCH), 3.43-3.36 (m, 0.5H, HOC*H*), 3.34-3.21 (m, 1.5H, C*H*CH_2OH and HOC*H*), 3.01 (ddd, J = 13.0, 13.0, 3.0 Hz, 0.5H, NCH), 2.49 (ddd, J = 13.0, 13.0, 3.0 Hz, 0.5H, NCH), 1.99 (s, 1.5H, C(O)Me), 1.95 (s, 1.5H, C(O)Me), 1.85-1.75 (m, 0.5H, CH), 1.73-1.50 (m, 1H, CH), 1.46-1.39 (m, 0.5H, CH), 1.34-1.09 (m, 2H, CH), 1.01 (d, J = 7.0 Hz, 1.5H, CH*Me*), 0.89 (d, J = 7.0 Hz, 1.5H, CH*Me*); ^{13}C NMR (100.6 MHz, $CDCl_3$) (rotamers) δ 169.3 (C=O), 169.0 (C=O), 64.3 (OCH_2), 63.9 (OCH_2), 50.2 (N*C*HMe), 44.8 (N*C*HMe), 42.1 (*C*HCH_2OH), 41.49 (NCH_2), 41.46 (*C*HCH_2OH), 36.1 (NCH_2), 25.7 (CH_2), 24.8 (CH_2), 21.9 (C(O)*Me*), 21.51 (CH_2), 21.47 (CH_2), 21.4 (C(O)*Me*), 11.2 (Me), 10.6 (Me); HRMS (ESI) *m/z* calcd for $C_9H_{17}NO_2$ $(M + Na)^+$ 194.1151, found 194.1142 (–5.0 ppm error).

Lab book reference HFK2-062

***tert*-Butyl (2*R**,3*R**)-3-(hydroxymethyl)-2-methylpiperidine-1-carboxylate S38**

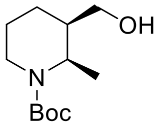

S38

Using general procedure F, $LiAlH_4$ (37 mg, 0.97 mmol, 1.0 eq.) and piperidine ester **S33** (250 mg, 0.97 mmol, 1.0 eq.) in THF (15 mL) gave *N*-Boc piperidine **S38** (218 mg, 98%) as a clear oil, R_F (7:3 hexane-EtOAc) 0.20; IR (ATR) 3434 (OH), 2974, 2930, 2862, 1687, 1661 (C=O), 1412, 1364, 1158 cm^{-1}; 1H NMR (400 MHz, $CDCl_3$) δ 4.39 (br s, 1H, NC*H*Me), 3.82 (s, 1H, NCH), 3.39 (br d, J = 6.0 Hz, 2H,

HOC*H*), 2.76-2.70 (m, 1H, NCH), 2.49-2.30 (m, 1H, OH), 1.92-1.71 (m, 1H, C*H*CH_2), 1.70-1.49 (m, 2H, CH), 1.36-1.43 (m, 10H, CMe_3 and CH), 1.28-1.08 (m, 1H, CH), 0.96 (d, J = 7.0 Hz, 3H, CH*Me*); ^{13}C NMR (100.6 MHz, $CDCl_3$) δ 155.1 (C=O), 79.4 (O*C*Me_3), 65.0 (OCH_2), 47.4 (N*C*HMe), 41.9 (*C*HCH_2OH), 38.6 (NCH_2), 28.6 (CMe_3), 25.4 (CH_2), 21.8 (CH_2), 10.9 (CH*Me*); HRMS (ESI) *m/z* calcd for $C_{12}H_{23}NO_3$ $(M + Na)^+$ 252.1570, found 252.1574 (–1.6 ppm error).
Lab book reference HFK2-002

***tert*-Butyl (2*R**,3*R**)-3-(methoxymethyl)-2-methylpiperidine-1-carboxylate S39**

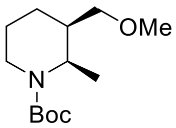

S39

NaH (60% dispersion in mineral oil, 79 mg 1.96 mmol, 1.6 eq.) was added portionwise to a stirred solution of **S38** (282 mg, 1.23 mmol, 1.0 eq.) in THF (6 mL) at –78 °C under Ar. Then, methyl iodide (139 µL, 2.22 mmol, 1.8 eq.) was added. After being allowed to warm to rt, the resulting mixture was stirred at rt for 18 h. 35% $NH_4OH_{(aq)}$ (20 mL) and EtOAc (20 mL) were added and the two layers were separated. The aqueous layer was extracted with EtOAc (3 × 15 mL). The combined organics were dried (Na_2SO_4) and evaporated under reduced pressure to give the crude product as a yellow oil. Purification by flash column chromatography on silica with 4:1 hexane-EtOAc as eluent gave ether **S39** (286 mg, 96%) as a pale yellow oil, R_F (3:2 hexane-EtOAc) 0.67; IR (ATR) 2975, 2929, 2860, 1686 (C=O), 1409, 1364, 1140 cm^{-1}; ^{1}H NMR (400 MHz, $CDCl_3$) δ 4.51-4.20 (m, 1H, NC*H*Me), 3.98-3.73 (m, 1H, NCH), 3.27 (s, 3H, OMe), 3.16-3.07 (m, 2H, OCH), 2.72 (br s, 1H, NCH), 1.95-1.83 (m, 1H, C*H*CH_2OMe), 1.64-1.45 (m, 2H, CH), 1.42-1.31 (m, 10H, CMe_3 and CH), 1.29-1.09 (m, 1H, CH), 0.92 (d, J = 7.0 Hz, 3H, CH*Me*); ^{13}C NMR (100.6 MHz, $CDCl_3$) (rotamers) δ 155.0 (C=O), 79.0 (O*C*Me_3), 75.1 (CH_2O*Me*), 58.8 (*C*H_2OMe), 48.0 (N*C*HMe), 47.1 (N*C*HMe), 39.1 (*C*HCH_2OMe), 38.2 (NCH_2), 28.5 (C*Me*$_3$), 25.3 (CH_2), 22.0 (CH_2), 10.9 (CH*Me*); HRMS (ESI) *m/z* calcd for $C_{13}H_{25}NO_3$ $(M + Na)^+$ 266.1727, found 266.1729 (+0.8 ppm error).
Lab book reference HFK2-007

(2*R,3*R**)-3-(Methoxymethyl)-2-methylpiperidine hydrochloride 19f**

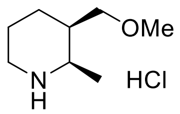

19f

Piperidine **S39** (90 mg, 0.37 mmol, 1.0 eq.) was dissolved in HCl (2.0 mL of a 2 M solution in Et_2O, 4.0 mmol, 11.0 eq.) and the resulting solution was stirred at rt for 18 h. Then, the solution was stirred and heated at 30 °for 2 h. After being allowed to cool to rt, the solvent was evaporated under reduced pressure to give piperidine hydrochloride **19f**·HCl (69 mg, quant.) as a white solid, mp 71-73 °C; R_F (9:1 CH_2Cl_2-MeOH) 0.36; IR (ATR) 2952, 2810, 1456, 1135, 1097, 474 cm^{-1}; ^1H NMR (400 MHz, $CDCl_3$) δ 9.64 (br s, 1H, NH), 8.93 (br s, 1H, NH), 3.65 (br s, 1H, NC*H*Me), 3.37 (dd, *J* = 10.0, 6.0 Hz, 1H, C*H*OMe), 3.31-3.27 (m, 1H, C*H*OMe), 3.29 (s, 3H, OMe), 3.15-3.01 (m, 2H, NCH), 2.36-2.27 (m, 1H, CH), 1.90-1.80 (m, 2H, CH), 1.66-1.44 (m, 2H, CH), 1.36 (d, *J* = 7.0 Hz, 3H, CH*Me*); ^{13}C NMR (100.6 MHz, $CDCl_3$) δ 73.0 (CH_2OMe), 59.1 (OMe), 51.0 (N*C*HMe), 40.2 (NCH_2), 36.2 (*C*HCH_2OMe), 22.4 (CH_2), 21.0 (CH_2), 12.0 (CH*Me*); HRMS (ESI) *m/z* calcd for $C_8H_{18}NO$ M^+ 144.1383, found 144.1385 (–1.6 ppm error).
Lab book reference HFK2-011

(2*R,3*R**)-2-Methylpiperidine-3-carboxamide hydrochloride 19g**

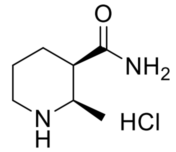

19g

Boc protected amide **S36** (56 mg, 0.23 mmol, 1.0 eq.) was dissolved in HCl (10 mL of a 2 M solution in Et_2O, 20 mmol, 87 eq.) and the resulting solution was stirred at rt for 18 h. Then, the solvent was evaporated under reduced pressure to give piperidine **19g**·HCl (46 mg, quant.) as a yellow oil, IR (ATR) 3162 (NH), 2952, 1634 (C=O), 748 cm^{-1}; 1H NMR (400 MHz, D_2O) δ 4.43 (br s, 2H, NC*H*Me and NH) 2.92 (br s, 2H, NCH), 2.55 (br s, 1H, C*H*$CONH_2$), 2.35 (br s, 1H, NH), 1.50-1.29 (m, 4H, CH), 0.85 (s, 3H, CH*Me*); ^{13}C NMR (100.6 MHz, D_2O) δ 177.7 (C=O), 52.6 (N*C*HMe), 43.5 (NCH_2), 40.5 (*C*$HCONH_2$), 24.8 (CH_2), 17.8 (CH_2), 15.2 (CH*Me*); HRMS (ESI) *m/z* calcd for $C_7H_{15}N_2O$ M^+ 143.1179, found 143.1179 (+0.2 ppm error).
Lab book reference HFK3-038

(2*R,3*R**)-1-Methanesulfonyl-*N*,2-dimethylpiperidine-3-carboxamide 19i**

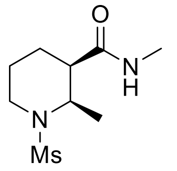

19i

Using general procedure J, DIPEA (347 µL, 2.0 mmol, 3.0 eq.), T3P (50 wt% in EtOAc, 297 µL, 1.00 mmol, 1.5 eq.) and acid **19k** (147 mg, 0.66 mmol, 1.0 eq.) in CH_2Cl_2 (5 mL) and methylamine (30 µL of a 33% wt solution in EtOH, 0.73 mmol, 1.1 eq.) gave the crude product. Purification by flash column chromatography on silica with EtOAc and then 9:1 CH_2Cl_2-MeOH as eluent gave amide **19i** (79 mg, 51%) as a colourless oil, R_F (9:1 CH_2Cl_2-MeOH) 0.52; IR (ATR) 3314 (NH), 2944, 1645 (C=O), 1317, 1133, 727 cm^{-1}; ^1H NMR (400 MHz, $CDCl_3$) δ 6.24 (br s, 1H, NH), 4.37-4.30 (m, 1H, NC*H*Me), 3.58 (br dd, *J* = 13.0, 4.5 Hz, 1H, NCH), 2.92 (ddd, *J* = 13.0, 13.0, 2.5 Hz, 1H, NCH), 2.82 (s, 3H, SO_2Me), 2.73 (d, *J* = 5.0 Hz, 3H, NH*Me*), 2.54 (ddd, *J* = 13.0, 4.0, 4.0 Hz, 1H, C*H*CONHMe), 1.81 (dddd, *J* = 13.0, 13.0, 13.0, 3.0 Hz, 1H, CH), 1.77-1.64 (m, 2H, CH), 1.49 (dddd, *J* = 13.0, 13.0, 13.0, 4.5, 4.5 Hz, 1H, CH), 1.11 (d, *J* = 7.0 Hz, 3H, CH*Me*); ^{13}C NMR (100.6 MHz, $CDCl_3$) δ 172.6 (C=O), 50.6 (N*C*HMe), 46.8 (*C*HCONHMe), 40.1 (SO_2Me), 39.4 (NCH_2), 26.3 (NHMe), 24.9 (CH_2), 20.2 (CH_2), 11.9 (CH*Me*). HRMS (ESI) *m/z* calcd for $C_9H_{18}N_2O_3S$ $(M + Na)^+$ 257.0930, found 257.0941 (–4.2 ppm error).
Lab book reference HFK 2-048

(2*R,3*R**)-1-Methanesulfonyl-2-methyl-3-(pyrrolidine-1-carbonyl)piperidine 19j**

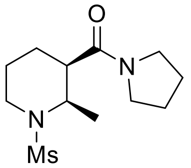

19j

Using general procedure J, DIPEA (246 µL, 1.41 mmol, 3.0 eq.), T3P (50 wt% in EtOAc, 210 µL 0.71 mmol, 1.5 eq.), acid **19k** (104 mg, 0.47 mmol, 1.0 eq.) in CH_2Cl_2 (5 mL) and pyrrolidine (43 µL, 0.52 mmol, 1.1 eq.) gave the crude product. Purification by flash column chromatography on silica with EtOAc and then 19:1 CH_2Cl_2-MeOH as eluent gave amide **19j** (63 mg, 49%) as a colourless oil, R_F (19:1 CH_2Cl_2-MeOH) 0.42; IR (ATR) 2953, 1625 (C=O), 1320, 1139, 725 cm^{-1}; ^1H NMR (400 MHz, $CDCl_3$) δ 4.37-4.24 (m, 1H, N*C*HMe), 3.58 (br dd, *J* = 13.0, 4.5 Hz, 1H, NCH), 3.56-3.48 (m, 2H, NCH), 3.47-3.41 (m, 1H, NCH), 3.33-3.24 (m, 1H, NCH), 2.92 (ddd, *J* = 13.0, 13.0, 3.0 Hz, 1H, NCH), 2.79 (s, 3H, SO_2Me), 2.76 (ddd, *J* = 13.0, 4.0, 4.0 Hz, 1H, *C*HC(O)N), 1.97 (dddd, *J* = 13.0, 13.0, 13.0, 4.0 Hz, 1H,

CH), 1.94-1.86 (m, 2H, CH), 1.81-1.74 (m, 2H, CH), 1.73-1.69 (m, 1H, CH), 1.66-1.56 (m, 1H, CH), 1.51 (ddddd, J = 13.0, 13.0, 13.0, 4.0, 4.0 Hz, 1H, CH), 1.13 (d, J = 7.0 Hz, 3H, CH*Me*); ^{13}C NMR (100.6 MHz, $CDCl_3$) δ 170.6 (C=O), 48.6 (N*C*HMe), 46.4 (NCH_2), 46.0 (NCH_2), 45.4 (*C*HC(O)N), 40.2 (SO_2Me), 39.3 (NCH_2), 26.3 (CH_2), 25.0 (CH_2), 24.1 (CH_2), 20.4 (CH_2), 12.1 (CH*Me*); HRMS (ESI) *m/z* calcd for $C_{12}H_{22}N_2O_3S$ $(M + Na)^+$ 297.1243, found 297.1250 (–2.5 ppm error).

Lab book reference HFK 2-050

1-*tert*-Butyl 2-methyl (2*R,3*S**)-3-methylpiperidine-1,2-dicarboxylate S40**

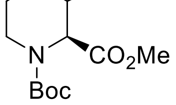

S40

A solution of Boc_2O (1.78 g, 8.15 mmol, 1.5 eq.) in CH_2Cl_2 (10 mL) was added to a stirred solution of an 85:15 mixture of **6b** and **S5** (854 mg, 5.44 mmol, 1.0 eq.) and Et_3N (2.27 mL, 16.3 mmol, 3.0 eq.) in CH_2Cl_2 (8 mL) at 0 °C under Ar. The resulting solution was stirred at rt for 60 h. The solvent was evaporated under reduced pressure and the residue was dissolved in Et_2O (20 mL). The organic layer was washed with 1 M $HCl_{(aq)}$ (2 × 20 mL) and saturated $NaHCO_{3(aq)}$ (20 mL), dried ($MgSO_4$) and evaporated under reduced pressure to give the crude product which contained an 85:15 mixture (by ^{1}H NMR spectroscopy) of **S40** and its *trans* diastereomer. Purification by flash column chromatography on silica with 95:5-80:20 hexane-EtOAc as eluent gave methyl ester **S40** (997 mg, 70%) as a yellow oil, R_F (95:5 hexane-EtOAc) 0.24; IR (ATR) 2934, 1738 (C=O, CO_2Me), 1692 (C=O, Boc), 1364, 1152, 871 cm^{-1}; ^{1}H NMR (400 MHz, $CDCl_3$) (50:50 mixture of rotamers) δ 4.72 (br s, 0.5H, NC*H*CO_2Me), 4.52 (br s, 0.5H, NC*H*CO_2Me), 3.93-3.82 (m, 1H, NCH), 3.64 (s, 3H, CO_2Me), 3.26-3.09 (m, 1H, NCH), 1.83-1.76 (m, 1H, C*H*Me), 1.67-1.63 (m, 1H, CH), 1.56-1.46 (m, 1H, CH), 1.39 (s, 9H, CMe_3), 1.36-1.18 (m, 2H, CH), 0.96 (d, J = 7.1 Hz, 3H, CH*Me*); ^{13}C NMR (100.6 MHz, $CDCl_3$) (rotamers) δ 172.2 (C=O, CO_2Me), 171.98 (C=O, CO_2Me), 155.8 (C=O, Boc), 155.4 (C=O, Boc), 80.1 (OC*Me*$_3$), 59.3 (*C*HCO_2Me), 57.8 (*C*HCO_2Me), 51.3 (OMe), 41.2 (NCH_2), 40.4 (NCH_2), 33.2 (*C*HMe), 33.0 (*C*HMe), 28.4 (C*Me*$_3$), 28.0 (CH_2), 25.3 (CH_2), 24.9 (CH_2), 18.6 (CH*Me*); HRMS (ESI) *m/z* calcd for $C_{13}H_{23}NO_4$ $(M + Na)^+$ 280.1519, found 280.1528 (–3.0 ppm error).

Lab book reference HFK3-019

Methyl (2*R,3*S**)-1-acetyl-3-methylpiperidine-2-carboxylate 20a**

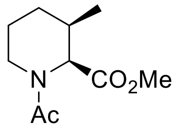

20a

TFA (1.0 mL, 13.0 mmol, 33 eq.) was added dropwise to a stirred solution of ester **S40** (100 mg, 0.39 mmol, 1.0 eq.) in CH_2Cl_2 (5 mL) at rt under Ar. The resulting solution was stirred at rt for 1 h. Then, the solvent was evaporated under reduced pressure to give the crude piperidine TFA salt as a clear oil. Using general procedure C, Ac_2O (220 µL, 2.30 mmol, 6.0 eq.) and crude piperidine TFA salt (max 0.39 mmol, 1.0 eq.) in pyridine (5 mL) gave the crude product as a yellow oil. Purification by flash column chromatography on silica with 4:1 hexane-EtOAc as eluent gave *N*-acyl piperidine **20a** (72 mg, 94%) as a clear oil, R_F (3:2 hexane-EtOAc) 0.13; IR (ATR) 2934, 1734 (C=O, CO_2Me), 1642 (C=O, C(O)Me), 1418, 1160, 1006 cm^{-1}; ^{1}H NMR (400 MHz, $CDCl_3$) (80:20 mixture of rotamers) δ 5.16 (d, J = 6.0 Hz, 0.8H, NC*H*CO_2Me), 4.50-4.40 (m, 0.2H, NCH), 4.32 (d, J = 6.0 Hz, 0.2H, NC*H*CO_2Me), 3.66-3.65 (m, 0.6H, OMe), 3.63-3.62 (m, 2.4HOMe), 3.60-3.59 (m, 0.8H, NCH), 3.51 (ddd, J = 13.0, 13.0, 3.0 Hz, 0.8H, NCH), 2.84 (ddd, J = 13.0, 13.0, 3.0 Hz, 0.2H, NCH), 2.07-2.06 (m, 0.6H, C(O)Me), 2.06-2.05 (m, 2.4H, C(O)Me), 1.84-1.64 (m, 1H, C*H*Me), 1.61-1.50 (m, 1H, CH), 1.50-1.30 (m, 2.8H, CH), 1.30-1.15 (m, 0.2H, CH), 0.98 (d, J = 7.0 Hz, 2.4H, CH*Me*), 0.97 (d, J = 7.0 Hz, 0.6H, CH*Me*); ^{13}C NMR (100.6 MHz, $CDCl_3$) (rotamers) δ 171.5 (C=O), 170.5 (C=O), 170.1 (C=O), 170.1 (C=O), 61.1 (*C*HCO_2Me), 55.7 (*C*HCO_2Me), 51.8 (OMe), 51.4 (OMe), 43.3 (NCH_2), 38.0 (NCH_2), 33.7 (*C*HMe), 33.1 (*C*HMe), 27.9 (CH_2), 27.6 (CH_2), 25.6 (CH2), 24.8 (CH_2), 22.1 (C(O)*Me*), 21.8 (C(O)*Me*), 18.6 (CH*Me*), 18.3 (CH*Me*); HRMS (ESI) *m/z* calcd for $C_{10}H_{17}NO_3$ $(M + Na)^+$ 222.1101, found 222.1108 (–3.9 ppm error).

Lab book reference HFK3-020

Methyl (2*R,3*S**)-1-methanesulfonyl-3-methylpiperidine-2-carboxylate 20b**

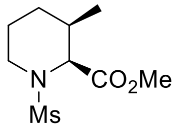

20b

Using general procedure D, Et_3N (533 µL, 3.82 mmol, 3.0 eq.), amine **6b** (200 mg, 1.27 mmol, 1.0 eq.) and MsCl (217 µL, 2.8 mmol, 2.2 eq.) in CH_2Cl_2 (5 mL) gave the crude product. Purification by flash column chromatography on silica with 95:5 hexane-EtOAc as eluent gave *N*-sulfonamide piperidine **20b** (217 mg, 73%) as a white solid, mp 50-52 °C; R_F (4:1 hexane-EtOAc) 0.32; IR (ATR) 2936, 1733 (C=O),

1326, 1146, 1000 cm^{-1}; ^{1}H NMR (400 MHz, $CDCl_3$) δ 4.50 (d, J = 6.0 Hz, 1H, NC*H*CO_2Me), 3.70 (s, 3H, OMe), 3.68-3.61 (m, 1H, NCH), 3.44 (ddd, J = 12.0, 12.0, 3.0 Hz, 1H, NCH), 2.76 (s, 3H, SO_2Me), 2.04-1.89 (m, 1H, C*H*Me), 1.82-1.71 (m, 1H, CH), 1.70-1.52 (m, 2H, CH), 1.35 (dddd, J = 13.0, 13.0, 13.0, 4.0 Hz, 1H, CH), 0.97 (d, J = 7.0 Hz, 3H, CH*Me*); ^{13}C NMR (100.6 MHz, $CDCl_3$) δ 171.4 (C=O), 59.2 (*C*HCO_2Me), 51.7 (OMe), 41.7 (NCH_2), 38.0 (SO_2Me), 34.1 (*C*HMe), 27.5 (CH_2), 25.3 (CH_2), 18.6 (CH*Me*); HRMS (ESI) *m/z* calcd for $C_9H_{17}NO_4S$ $(M + Na)^+$ 258.0770, found 258.0773 (–0.6 ppm error). Lab book reference HFK3-015

[(2*R,3*S**)-1-Methanesulfonyl-3-methylpiperidin-2-yl]methanol 20c**

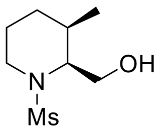

20c

Using general procedure F, methyl ester **20b** (233 mg, 1.00 mmol, 1.0 eq.) in THF (10 mL) and $LiAlH_4$ (83 mg, 2.19 mmol, 2.2 eq.) in THF (20 mL) gave the crude product. Purification by flash column chromatography on silica with 70:30-60:40 hexane-EtOAc as eluent gave alcohol **20c** (39 mg, 19%) as a clear oil, R_F (1:1 hexane-EtOAc) 0.21; IR (ATR) 3502 (OH), 2930, 2876, 1313, 1140, 1059, 769 cm^{-1}; ^{1}H NMR (400 MHz, $CDCl_3$) δ 3.99-3.94 (m, 1H, NC*H*CH_2OH), 3.78 (dd, J = 12.0, 12.0 Hz, 1H, HOC*H*), 3.76-3.68 (m, 1H, NCH), 3.67 (dd, J = 12.0, 4.0 Hz, 1H, HOC*H*), 3.02-2.97 (m, 1H, NCH), 2.93 (s, 3H, SO_2Me), 2.07 (br s, 1H, OH), 1.94-1.86 (m, 1H, C*H*Me), 1.73-1.64 (m, 1H, CH), 1.64-1.47 (m, 2H, CH), 1.35-1.12 (m, 1H, CH), 0.92 (d, J = 7.0 Hz, 3H, CH*Me*); ^{13}C NMR (100.6 MHz, $CDCl_3$) δ 59.6 (N*C*HCH_2OH), 56.5 (OCH_2), 40.4 (SO_2Me), 39.9 (NCH_2), 33.5 (*C*HMe), 27.9 (CH_2), 25.8 (CH_2), 18.8 (CH*Me*); HRMS (ESI) *m/z* calcd for $C_8H_{17}NO_3S$ $(M + Na)^+$ 230.0821, found 230.0820 (0.5 ppm error). Lab book reference HFK3-023

1-[(*tert*-Butoxy)carbonyl]-2-methylpyrrolidine-2-carboxylic acid S41

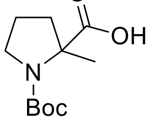

S41

A solution of NaOH (1.88 g, 46.9 mmol, 3.0 eq.) in H_2O (20 mL) was added dropwise to a stirred solution of ester **4a** (3.80 g, 15.6 mmol, 1.0 eq.) in MeOH (20 mL) at rt under Ar. The resulting solution was stirred and heated at 80 °C for 1 h. After being allowed to cool to rt, NaOH (1.27 g, 32.8 mmol, 2.1 eq.)

was added and the resulting solution was stirred and heated at 80 °C for 1 h. After being allowed to cool to rt, the solvent was evaporated under reduced pressure. H_2O (25 mL) was added and the mixture was acidified to pH 2 with 12 M $HCl_{(aq)}$ (3 mL). Et_2O (25 mL) was added and the two layers were separated. The aqueous layer was extracted with Et_2O (2 × 25 mL) and the combined organics were dried ($MgSO_4$) and evaporated under reduced pressure to give acid **S41** (3.32 g, 93%) as a white solid, mp 90-93 °C (lit.,[21] 91-94 °C); R_F (3:2 hexane-EtOAc) 0.35; ^{1}H NMR (400 MHz, $CDCl_3$) (55:45 mixture of rotamers) δ 3.68-3.29 (m, 2H, NCH), 2.56-2.39 (m, 0.45H, CH), 2.28-2.21 (m, 0.55H, CH), 2.02-1.71 (m, 3H, CH), 1.58 (s, 1.35H, NCMe), 1.49 (s, 1.65H, NCMe), 1.44 (s, 4.05H, CMe_3), 1.40 (s, 4.95H, CMe_3); ^{13}C NMR (100.6 MHz, $CDCl_3$) (rotamers) δ 181.1 (C=O, CO_2H), 177.9 (C=O, CO_2H), 155.8 (C=O, Boc), 153.7 (C=O, Boc), 81.2 (O*C*Me$_3$), 80.5 (O*C*Me$_3$), 66.2 (N*C*Me), 64.9 (N*C*Me), 48.6 (NCH_2), 47.9 (NCH_2), 40.5 (CH_2), 38.8 (CH_2), 28.6 (C*Me*$_3$), 28.4 (C*Me*$_3$), 23.1 (CH_2), 23.0 (NC*Me*), 22.9 (CH_2), 22.4 (NC*Me*). Spectroscopic data consistent with those reported in the literature.[21]

Lab book reference HFK1-034

***tert*-Butyl 2-carbamoyl-2-methylpyrrolidine-1-carboxylate S42**

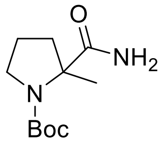

S42

Isobutyl chloroformate (1.2 mL, 9.00 mmol, 1.2 eq.) was added dropwise to a stirred solution of carboxylic acid **S41** (1.72 g, 7.51 mmol, 1.0 eq.) and Et_3N (1.1 mL, 7.51 mmol, 1.0 eq.) in THF (80 mL) at –20 °C under Ar. The resulting solution was stirred at –20 °C for 40 min. Then 35% $NH_4OH_{(aq)}$ (16 mL) was added and the mixture was allowed to warm to rt over 30 min. The solvent was evaporated under reduced pressure and the residue was dissolved in EtOAc (50 mL). The organic layer was washed with $NaHCO_{3(aq)}$ (40 mL) and brine (40 mL), dried ($MgSO_4$) and evaporated under reduced pressure to give the crude product. Purification by flash column chromatography on silica with 60:40 hexane-EtOAc and then 90:10 CH_2Cl_2-MeOH as eluent gave amide **S42** (608 mg, 36%) as a white solid, mp 106-108 °C; R_F (9:1 CH_2Cl_2-MeOH) 0.49; ^{1}H NMR (400 MHz, $CDCl_3$) (55:45 mixture of rotamers) δ 6.96 (br s, 0.45H, NH), 6.31 (br s, 0.55H, NH), 6.10 (br s, 1H, NH), 3.67-3.21 (m, 2H, NCH), 2.45 (br s, 0.55H, CH), 2.19 (br s, 0.55H, CH), 1.93-1.60 (m, 2.9H, CH), 1.53 (s, 1.35H, NCMe), 1.44 (s, 1.65H, NCMe), 1.34 (s, 9H, CMe_3); ^{13}C NMR (100.6 MHz, $CDCl_3$) (rotamers) δ 178.0 (C=O, $CONH_2$), 177.8 (C=O, $CONH_2$), 154.7 (C=O, Boc), 153.9 (C=O, Boc), 80.5 (O*C*Me$_3$), 80.3 (O*C*Me$_3$), 66.7 (N*C*Me), 65.9 (N*C*Me), 48.7 (NCH_2), 47.9 (NCH_2), 41.3 (CH_2), 39.0 (CH_2), 28.4 (C*Me*$_3$), 22.5 (NC*Me*).

Lab book reference HFK1-070

2-Methylpyrrolidine-2-carboxamide hydrochloride 21a

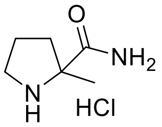

21a

Boc protected amide **S42** (200 mg, 0.93 mmol, 1.0 eq.) was dissolved in HCl (2.8 mL of a 2 M solution in Et_2O, 5.6 mmol, 6.0 eq.) and the resulting solution was stirred at rt for 18 h. Then, HCl (5.6 mL of a 2 M solution in Et_2O, 11.2 mmol, 12.0 eq.) was added and the resulting solution was stirred at rt for 18 h. Then, the solvent was evaporated under reduced pressure to give pyrrolidine **21a**·HCl (67 mg, 47%) as a cream powder, mp 100-103 °C; IR (ATR) 3336 (NH), 3272 (NH), 3180 (N-H), 1697 (C=O), 1629 (C=O), 609 cm^{-1}; ^{1}H NMR (400 MHz, MeOH-*d*$_4$) δ 3.40-3.30 (m, 2H, NCH), 2.39-2.28 (m, 1H, CH), 2.17-2.03 (m, 2H, CH), 2.00-1.88 (m, 1H, CH), 1.65 (s, 3H, NCMe); ^{13}C NMR (100.6 MHz, MeOH-*d*$_4$) δ 174.7 (C=O), 70.8 (N*C*Me), 46.6 (NCH_2), 37.3 (CH_2), 24.6 (CH_2), 22.4 (NC*Me*); HRMS (ESI) *m/z* calcd for $C_6H_{13}N_2O$ M^+ 129.1022, found 129.1021 (–1.9 ppm error).
Lab book reference HFK1-060

1-Methanesulfonyl-2-methylpyrrolidine-2-carboxylic acid 21d

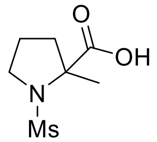

21d

A solution of LiOH (110 mg, 4.59 mmol, 8.1 eq.) in H_2O (4 mL) was added dropwise to a stirred solution of ester **1e** (125 mg, 0.57 mmol, 1.0 eq.) in MeOH (4 mL) at rt under Ar. The resulting solution was stirred at rt for 2.5 h. Then, the solvent was evaorated under reduced pressure. H_2O (10 mL) was added and the mixture acidified to pH 2 with 12 M $HCl_{(aq)}$ (1 mL). EtOAc (10 mL) was added and the two layers were separated. The aqueous layer was extracted with EtOAc (3 × 10 mL) and the combined organics were dried ($MgSO_4$) and evaporated under reduced pressure to give acid **21d** (100 mg, 85%) as a white solid, mp 127-129 °C; R_F (9:1 CH_2Cl_2-MeOH) 0.21; IR (ATR) 2893, 2676, 1701 (C=O), 1312, 1141, 767 cm^{-1}; ^{1}H NMR (400 MHz, MeOH-*d*$_4$) 3.68-3.42 (m, 2H, NCH), 2.99 (s, 3H, SO_2Me), 2.35-2.16 (m, 1H, CH), 2.12-1.87 (m, 3H, CH), 1.62 (s, 3H, NCMe); ^{13}C NMR (100.6 MHz, MeOH-*d*$_4$) 177.5

(C=O), 69.8 (N*C*Me), 50.0 (NCH_2), 42.1 (CH_2), 39.7 (SO_2Me), 24.9 (NC*Me*), 24.4 (CH_2); HRMS (ESI) *m/z* calcd for $C_7H_{13}NO_4S$ $(M + Na)^+$ 230.0457, found 230.0452 (+2.4 ppm error).
Lab book reference HFK2-089

1-Methanesulfonyl-2-methylpyrrolidine-2-carboxamide 21b

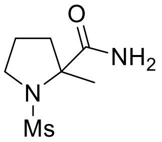

21b

Using general procedure J, DIPEA (399 µL, 2.29 mmol, 3.0 eq.), T3P (50 wt% in EtOAc, 339 µL, 1.14 mmol, 1.5 eq.), acid **21d** (158 mg, 0.763 mmol, 1.0 eq.) in CH_2Cl_2 (4 mL) and 35% $NH_4OH_{(aq)}$ (1.12 mL, 3.81 mmol, 5 eq.) gave the crude product. Purification by flash column chromatography on silica with 95:5 CH_2Cl_2-MeOH as eluent gave sulfonamide **21b** (39 mg, 25%) as a cream solid, mp 117-119 °C; R_F (9:1 CH_2Cl_2-MeOH) 0.32; IR (ATR) 3457 (NH), 3356 (NH), 2983, 2879, 1668 (C=O), 1314, 1138, 728 cm^{-1}; ^1H NMR (400 MHz, $CDCl_3$) δ 6.58 (br s, 1H, NH), 5.87 (br s, 1H, NH), 3.70-3.55 (m, 1H, NCH), 3.49-3.29 (m, 1H, NCH), 2.95 (s, 3H, SO_2Me), 2.59-2.42 (m, 1H, CH), 2.07-1.77 (m, 3H, CH), 1.68 (s, 3H, NCMe); ^{13}C NMR (100.6 MHz, $CDCl_3$) δ 176.8 (C=O), 69.5 (N*C*Me), 50.1 (NCH_2), 41.3 (CH_2), 39.1 (SO_2Me), 23.2 (CH_2), 22.9 (NC*Me*); HRMS (ESI) *m/z* calcd for $C_7H_{14}N_2O_3S$ $(M + Na)^+$ 229.0617, found 229.0618 (–0.4 ppm error).
Lab book reference HFK2-092

1-Acetyl-2-methylpyrrolidine-2-carboxylic acid 21c

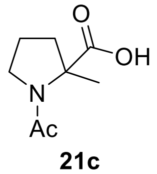

21c

A solution of LiOH (90 mg, 0.49 mmol, 3.0 eq.) in H_2O (3 mL) was added dropwise to a stirred solution of ester **1d** (100 mg, 0.54 mmol, 1.0 eq.) in MeOH (3 mL) at rt under Ar. The resulting solution was stirred and heated at 40 °C for 18 h. After being allowed to cool to rt, the solvent was evaporated under reduced pressure. H_2O (10 mL) was added and the mixture was acidified to pH 2 with 12 M $HCl_{(aq)}$ (0.5 mL). EtOAc (10 mL) was added and the two layers were separated. The aqueous layer was extracted with EtOAc (3 × 10 mL) and the combined organics were dried ($MgSO_4$) and evaporated under reduced pressure to give acid **21c** (74 mg, 80%) as a white solid, mp 119-121 °C; R_F (9:1 CH_2Cl_2-MeOH) 0.58;

IR (ATR) 2979, 1727 (C=O, CO_2H), 1598 (C=O, C(O)Me), 1419, 1180, 725 cm^{-1}; ^1H NMR (400 MHz, $CDCl_3$) δ 10.37 (s, 1H, OH), 3.72-3.47 (m, 2H, NCH), 2.39 (ddd, J = 12.0, 7.0, 7.0 Hz, 1H, CH), 2.09 (s, 3H, C(O)Me), 2.06-1.90 (m, 2H, CH), 1.82 (br dd, J = 12.0, 7.0 Hz, 1H, CH), 1.56 (s, 3H, CMe); ^{13}C NMR (100.6 MHz, $CDCl_3$) δ 176.0 (C=O), 171.5 (C=O), 66.9 (N*C*Me), 49.7 (NCH_2), 38.3 (CH_2), 23.8 (CH_2), 23.2 (C(O)Me), 21.7 (C*Me*); HRMS (ESI) *m/z* calcd for $C_8H_{13}NO_3$ $(M + Na)^+$ 194.0788, found 194.0788 (+0.1 ppm error).
Lab book reference HFK1-079

1-Methanesulfonyl-2-methylpyrrolidine-2-carbonitrile 21e

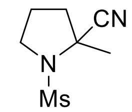

21e

Using general procedure L, Et_3N (656 µL, 4.70 mmol, 2.4 eq.), trifluoroacetic anhydride (332 µL, 2.35 mmol, 1.2 eq.) and pyrrolidine amide **21b** (404 mg, 1.96 mmol, 1.0 eq.) in THF (35 mL) gave the crude product. Purification by flash column chromatography on silica with 50:50 hexane-EtOAc as eluent gave pyrrolidine nitrile **21e** (312 mg, 85%) as a white solid, mp 99-101 °C; R_F (50:50 hexane-EtOAc) 0.30; IR (ATR) 2987, 2938, 1330, 1146, 757, 517 cm^{-1}; ^1H NMR (400 MHz, $CDCl_3$) δ 3.65-3.54 (m, 1H, NCH), 3.48-3.43 (m, 1H, NCH), 3.03 (s, 3H, SO_2Me), 2.55-2.48 (m, 1H, CH), 2.21-1.94 (m, 3H, CH), 1.82 (s, 3H, NCMe); ^{13}C NMR (100.6 MHz, $CDCl_3$) δ 120.3 (CN), 58.2 (N*C*Me), 49.5 (NCH_2), 42.2 (CH_2), 37.0 (SO_2Me), 27.1 (NC*Me*), 22.9 (CH_2); HRMS (ESI) *m/z* calcd for $C_7H_{12}N_2O_2S$ $(M + Na)^+$ 211.0512, found 211.0514 (–1.3 ppm error).
Lab book reference HFK2-099

***tert*-Butyl 2-(hydroxymethyl)-2-methylpyrrolidine-1-carboxylate S43**

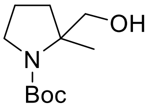

S43

$LiBH_4$ (0.31 mL of a 4 M solution in THF, 1.23 mmol, 1.5 eq.) was added dropwise to a stirred solution of Boc pyrrolidine **4a** (200 mg, 0.82 mmol, 1.0 eq.) in THF (5 mL) at 0 °C under Ar. The resulting solution was stirred at rt for 48 h. After cooling to –20 °C, water (5 mL) and 3 M $HCl_{(aq)}$ (5 mL) were added sequentially. The two layers were separated and the aqueous layer was extracted with EtOAc (3 × 20 mL). The combined organics were washed with brine (20 mL), dried ($MgSO_4$) and evaporated under

reduced pressure to give alcohol **S43** (157 mg, 89%) as a colourless oil, R_F (60:40 hexane-EtOAc) 0.50; IR (ATR) 3398 (OH), 2972, 2876, 1662 (C=O), 1391, 1170, 771 cm^{-1}; ^{1}H NMR (400 MHz, $CDCl_3$) δ 5.32 (d, J = 10.0 Hz, 1H, OH), 3.64 (dd, J = 10.0, 10.0 Hz, 1H, HOC*H*), 3.56 (br d, J = 10.0 Hz, 1H, HOC*H*), 3.52-3.45 (m, 1H, NCH), 3.31-3.24 (m, 1H, NCH), 1.88-1.69 (m, 2H, CH) 1.69-1.59 (m, 2H, CH), 1.42 (s, 9H, CMe_3), 1.33 (s, 3H, CMe); ^{13}C NMR (100.6 MHz, $CDCl_3$) δ 156.1 (C=O), 80.1 (O*C*Me_3), 70.8 (OCH_2), 64.9 (N*C*Me), 48.6 (NCH_2), 37.7 (CH_2), 28.6 (C*Me*$_3$), 21.9 (CH_2), 20.4 (C*Me*); HRMS (ESI) *m/z* calcd for $C_{11}H_{21}NO_3$ $(M + Na)^+$ 238.1414, found 238.1410 (+1.8 ppm error).
Lab Book Reference: HFK 1-015

***tert*-Butyl 2-(methoxymethyl)-2-methylpyrrolidine-1-carboxylate S44**

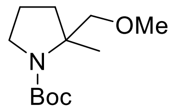

S44

NaH (60% dispersion in mineral oil, 397 mg, 9.90 mmol, 1.5 eq.) was added portion-wise to a stirred solution of **S43** (1.42 g, 6.60 mmol, 1.0 eq.) in THF (24 mL) at –78 °C under Ar. Then, methyl iodide (698 µL, 11.2 mmol, 1.7 eq.) was added. After being allowed to warm to rt, the resulting mixture was stirred at rt for 18 h. Saturated $NH_4Cl_{(aq)}$ (20 mL) and 35% $NH_4OH_{(aq)}$ (20 mL) and EtOAc (20 mL) were added sequentially and the two layers were separated. The aqueous layer was extracted with EtOAc (2 × 40 mL). The combined organic extracts were dried (Na_2SO_4) and evaporated under reduced pressure to give the crude product. Purification by flash column chromatography on silica with 80:20 hexane-EtOAc as eluent gave ester **S44** (561 mg, 37%) as a pale yellow oil, R_F (67:33 hexane-EtOAc) 0.38; IR (ATR) 2973, 2875, 1689 (C=O), 1364, 1174, 771 cm^{-1}; ^{1}H NMR (400 MHz, $CDCl_3$) (50:50 mixture of rotamers) δ 3.67 (d, J = 9.0 Hz, 0.5H, C*H*OMe), 3.49-3.45 (m, 1.5H, C*H*OMe), 3.38-3.35 (m, 1.5H, NCH), 3.31 (s, 3H, OMe), 3.30-3.24 (m, 0.5H, NCH), 2.16-2.05 (m, 1H, CH), 1.78-1.56 (m, 3H, CH), 1.45 (s, 4.5H, CMe_3), 1.41 (s, 4.5H, CMe_3), 1.29 (s, 1.5H, CMe), 1.24 (s, 1.5H, CMe); ^{13}C NMR (100.6 MHz, $CDCl_3$) (rotamers) δ 154.4 (C=O), 153.8 (C=O), 79.4 (O*C*Me_3), 78.7 (O*C*Me_3), 77.3 (OCH_2), 76.3 (OCH_2), 62.8 (N*C*Me), 62.2 (N*C*Me), 59.3 (OMe), 48.7 (NCH_2), 38.3 (CH_2), 37.1 (CH_2), 28.7 (C*Me*$_3$), 23.2 (C*Me*), 22.2 (CH_2), 22.1 (C*Me*), 21.7 (CH_2); HRMS (ESI) *m/z* calcd for $C_{12}H_{23}NO_3$ $(M + Na)^+$ 252.1570, found 252.1566 (+1.7 ppm error).
Lab Book Reference: HFK 1-042

2-(Methoxymethyl)-2-methylpyrrolidine hydrochloride 21f

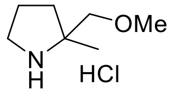

21f

Pyrrolidine **S44** (200 mg, 0.87 mmol, 1.0 eq.) was dissolved in HCl (5.0 mL of a 2 M solution in Et_2O, 10 mmol, 11.0 eq.) and the resulting solution was stirred at rt for 18h. Then, HCl (3.0 mL of a 2 M solution in Et_2O, 6.0 mmol, 7.0 eq.) was added and the resulting solution was stirred at rt for 6 h. The solvent was evaporated under reduced pressure to give pyrrolidine **21f**·HCl (123 mg, 85%) as a white solid, mp 100-104 °C; R_F (9:1 CH_2Cl_2-MeOH) 0.29; IR (ATR) 2895, 2653, 2504, 1394, 1107, 572 cm^{-1}; ^{1}H NMR (400 MHz, MeOH-d_4) δ 3.45 (s, 2H, CH_2OMe), 3.41 (s, 3H, OMe), 3.31-3.27 (m, 2H, NCH), 2.14-1.95 (m, 3H, CH), 1.83-1.78 (m, 1H, CH), 1.39 (s, 3H, CMe); ^{13}C NMR (100.6 MHz, MeOH-d_4) δ 76.6 (CH$_2$OMe), 68.2 (NCMe), 59.6 (OMe), 46.3 (NCH_2), 34.7 (CH_2), 24.4 (CH_2), 21.7 (CMe); HRMS (ESI) *m/z* calcd for $C_7H_{16}NO$ M^+ 130.1226, found 130.1228 (–1.5 ppm error).
Lab Book Reference: HFK 1-062

***tert*-Butyl 1-acetyl-2-methylpyrrolidine-4-carboxylate 11c**

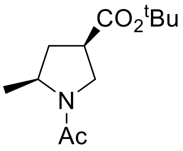

11c

MsCl (474 µL, 6.12 mmol, 3.0 eq.) was added dropwise to a stirred solution of pyrrolidine salt **11b**·AcOH (500 mg, 2.04 mmol, 1.0 eq.) and Et_3N (1.14 mL, 8.16 mmol, 4.0 eq.) in CH_2Cl_2 (20 mL) at rt. The resulting solution was stirred at rt for 3 h. Saturated $NaHCO_{3(aq)}$ (20 mL) was added and the aqueous layer was extracted with CH_2Cl_2 (3 × 20 mL). The combined organic layers were dried (Na_2SO_4) and evaporated under reduced pressure to give the crude product. Purification by flash column chromatography on silica with 50:50 hexane-EtOAc, then EtOAc and then 80:20 EtOAc-MeOH as eluent gave pyrrolidine **11c** (242 mg, 52%) as a red oil, R_F (50:50 hexane-EtOAc) 0.16; IR (ATR) 1724 (C=O, CO_2CMe_3), 1631 (C=O, C(O)Me), 1418, 1368, 1154 cm^{-1}; ^{1}H NMR (400 MHz, $CDCl_3$) (65:35 mixture of rotamers) δ 4.10 (ddq, J = 6.5, 6.5, 6.5 Hz, 0.65H, NCHMe), 4.00 (dd, J = 12.5, 8.5 Hz, 0.35H, NCH), 3.97-3.90 (m, 0.35H, NCHMe), 3.70 (dd, J = 10.5, 7.5 Hz, 0.65H, NCH), 3.60 (dd, J = 10.5, 8.0 Hz, 0.65H, NCH), 3.52 (dd, J = 12.5, 7.0 Hz, 0.35H, NCH), 2.96-2.84 (m, 1H, CHCO$_2$Me$_3$), 2.44-2.29 (m, 1H, CH), 2.05 (s, 1.05H, C(O)Me), 2.03 (s, 1.95H, C(O)Me), 2.00-1.94 (m, 0.35H, CH), 1.82 (ddd, J = 13.5, 7.5, 6.0 Hz, 0.65H, CH), 1.44 (s, 5.85H, CMe_3), 1.43 (s, 3.15H, CMe_3), 1.22 (d, J = 6.5 Hz, 3H,

CHMe); ^{13}C NMR (100.6 MHz, $CDCl_3$) (rotamers) 172.5 (C=O, *C*(O)Me), 172.2 (C=O, *C*(O)Me), 169.08 (C=O, *C*O_2CMe$_3$), 169.05 (C=O, *C*O_2CMe$_3$), 81.5 (O*C*Me$_3$), 81.3 (O*C*Me$_3$), 53.8 (N*C*HMe), 52.9 (N*C*HMe), 49.3 (NCH_2), 47.2 (NCH_2), 43.5 (*C*HCO_2), 42.2 (*C*HCO_2), 36.7 (CH_2), 36.2 (CH_2), 28.1 (C*Me*$_3$), 23.2 (C(O)*Me*), 21.8 (Me), 21.7 (Me), 20.0 (C*Me*); HRMS (ESI) *m/z* calcd for $C_{12}H_{21}NO_3$ (M + Na)$^+$ 250.1414, found 250.1425 (–4.5 ppm error).
Lab Book Reference: TD 3/53

***tert*-Butyl 1-methanesulfonyl-2-methylpyrrolidine-4-carboxylate 11d**

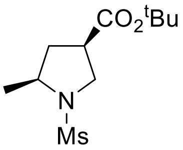

11d

MsCl (0.032 mL, 1.0 eq, 0.40 mmol) was added dropwise to a stirred solution of pyrrolidine salt **11b**·AcOH (100 mg, 1.0 eq, 0.40 mmol) in pyridine (10 mL) at 0 °C. The resulting solution was stirred at 0 °C for 1 h then allowed to warm to rt and stirred at rt for 16 h. The solvent was evaporated under reduced pressure to give the crude product. Purification by flash column chromatography on silica with 50:50 hexane-EtOAc as eluent gave pyrrolidine **11d** (89 mg, 83%) as a red oil, R_F (50:50 hexane-EtOAc) 0.32; IR (ATR) 1725 (C=O), 1332, 1150 cm^{-1}; ^{1}H NMR (400 MHz, $CDCl_3$) δ 3.90 (ddq, J = 6.5, 6.5, 6.5 Hz, 1H, NC*H*Me), 3.70 (dd, J = 11.0, 8.0 Hz, 1H, NCH), 3.54 (dd, J = 11.0, 8.5 Hz, 1H, NCH), 2.97-2.88 (m, 1H, $CHCO_2$), 2.87 (s, 3H, SO_2Me), 2.41 (ddd, J = 13.0, 7.5, 6.5 Hz, 1H, CH), 1.88 (ddd, J = 13.0, 9.0, 6.5 Hz, 1H, CH), 1.46 (s, 9H, CMe_3), 1.36 (d, J = 6.5 Hz, 3H, CH*Me*); ^{13}C NMR (100.6 MHz, $CDCl_3$) δ 171.4 (C=O), 81.7 (O*C*Me$_3$), 56.2 (N*C*HMe), 50.2 (NCH_2), 43.7 (*C*HCO_2), 37.8 (SO_2Me), 37.6 (CH_2), 28.1 (C*Me*$_3$), 22.0 (CH*Me*); HRMS (ESI) *m/z* calcd for $C_{11}H_{21}NO_4S$ (M + Na)$^+$ 286.1083, found 286.1085 (–0.5 ppm error).

Structure confirmed by small molecule X-Ray crystallography (CCDC 1919206):

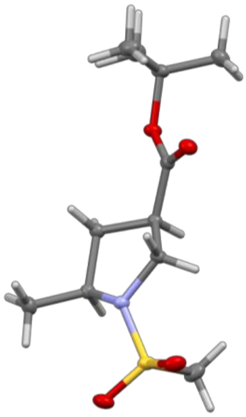

Lab Book Reference: TD 3/55

Methyl 1-benzyl-2-methyl-4,5-dihydro-1H-pyrrole-3-carboxylate S45

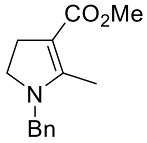

S45

Methyl acetoacetate (3.0 mL, 26.0 mmol, 1.0 eq) was added dropwise to a stirred suspension of 1,2-dibromoethane (4.7 mL, 53.0 mmol, 2.0 eq) and K_2CO_3 (9.5 g, 66.0 mmol, 2.5 eq) in MeCN (50 mL, 0.55 M) at rt under Ar. The resulting suspension was stirred and heated at reflux for 23 h. After being allowed to cool to rt, the solids were removed by filtration and the filtrate was evaporated under reduced pressure to give the crude product as an orange oil. Benzylamine (4.26 mL, 39.0 mmol, 1.5 eq) was added to a stirred solution of the crude product in toluene (30 mL) at rt under Ar. The resulting solution was stirred and heated at reflux for 48 h. After being allowed to cool to rt, the solvent was evaporated under reduced pressure to give the crude product as an orange oil. Purification by flash column chromatography on silica with 19:1 hexane-EtOAc as eluent gave dihydropyrrole **S45** (2.88 g, 44% over 2 steps) as a colourless oil, R_F (5:1 hexane-EtOAc) 0.24; IR (ATR) 2946, 1668 (C=O), 1421, 1128, 727 cm^{-1}; ^1H NMR (400 MHz, $CDCl_3$) δ 7.36-7.30 (m, 2H, Ph), 7.29-7.25 (m, 1H, Ph), 7.19-7.14 (m, 2H, Ph), 4.28 (s, 2H, NCH_2Ph), 3.65 (s, 3H, OMe), 3.29 (t, J = 9.5, 2H, NCH_2), 2.70 (t, J = 9.5, 2H, NCH_2CH_2), 2.29 (s, 3H, Me); ^{13}C NMR (100.6 MHz, $CDCl_3$) δ 167.8 (C=O), 161.7 (C=*C*Me), 137.3 (*ipso*-Ph), 128.8 (Ph), 127.5 (Ph), 127.2 (Ph), 96.4 (C=*C*CO_2Me), 50.7 (NCH_2Ph), 50.5 (NCH_2), 50.2 (OMe), 27.0 (CH_2), 21.2 (Me); MS (ESI) *m/z* 232 $(M + H)^+$; HRMS *m/z* calcd for $C_{14}H_{17}NO_2$ $(M + H)^+$ 232.1332, found 232.1337 (–1.5 ppm error). Spectroscopic data consistent with those reported in the literature.[22]

Lab Book Reference: PJ-01-71

Methyl 1-benzyl-2-methylpyrrolidine-3-carboxylate S46

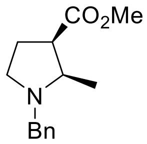

S46

$NaBH(OAc)_3$ (2.47 g, 1.17 mmol, 3.0 eq) was added to a stirred solution of dihydropyrrole **S45** (900 mg, 3.9 mmol, 1.0 eq) in 1:1 AcOH-MeCN (12 mL) at rt under Ar. The resulting solution was cooled to 0 °C and stirred at 0 °C for 3 h. Then, the solvent was evaporated under reduced pressure to give an orange oil. The residue was dissolved in CH_2Cl_2 (30 mL) and saturated $NaHCO_{3(aq)}$ (70 mL) was added. The two layers were separated and the aqueous layer was extracted with CH_2Cl_2 (3 x 30 mL). The combined organics were dried ($MgSO_4$) and evaporated under reduced pressure to give the crude product as a yellow/orange oil which contained an 85:15 mixture of pyrrolidines **S46** and its *trans* diastereomer (by ^{1}H NMR spectroscopy). Purification by flash column chromatography on silica with 9:1 hexane-EtOAc as eluent gave pyrrolidine **S46** (590 mg, 65%) as a colourless oil, R_F (9:1 hexane-EtOAc) 0.1; IR (ATR) 2970, 1736 (C=O), 1434, 1164, 731 cm^{-1}; ^{1}H NMR (400 MHz, $CDCl_3$) δ 7.31-7.14 (m, 5H, Ph), 3.85 (d, J = 13.5 Hz, 1H, NCHPh), 3.65 (s, 3H, OMe), 3.33 (d, J = 13.5 Hz, 1H, NCHPh), 3.02 (ddd, J = 8.0, 8.0, 8.0 Hz, 1H, $CHCO_2$) 2.94-2.80 (m, 2H, C*H*Me and NCH) 2.26 (ddd, J = 9.0, 9.0, 7.0 Hz, 1H, NCH), 2.23-2.14 (m, 1H, CH), 1.92-1.83 (m, 1H, CH), 0.99 (d, J = 6.5 Hz, 3H, CH*Me*); ^{13}C NMR (100.6 MHz, $CDCl_3$) δ 174.4 (C=O), 139.2 (*ipso*-Ph), 128.9 (Ph), 128.3 (Ph), 127.0 (Ph), 60.1 (NCH), 57.4 (NCH_2Ph), 52.3 (NCH_2), 51.6 (OMe), 47.4 (*C*HCO_2Me), 25.9 (CH_2), 14.7 (Me); MS (ESI) *m/z* 234 (M + H)$^+$; HRMS *m/z* calcd for $C_{14}H_{19}NO_2$ (M + H)$^+$ 234.1489, found 234.1498 (–3.9 ppm error) and a 65:35 mixture (by ^{1}H NMR spectroscopy) of the *trans*-pyrrolidine and **S46** (162 mg, 17%) as a yellow oil. Diagnostic signal for the *trans* diastereomer: ^{1}H NMR (400 MHz, $CDCl_3$) δ 1.29 (d, J = 6.0 Hz, 3H, CH*Me*).

Lab Book Reference: PJ-01-23

3-(Methoxycarbonyl)-2-methylpyrrolidin-1-ium chloride S47·HCl

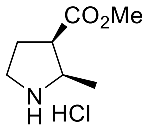

S47

10% Pd/C (160 mg, 0.15 mmol, 0.7 eq) was added to a stirred solution of benzyl pyrrolidine **S46** (500 mg, 2.15 mmol, 1.0 eq) in MeOH (10 mL) at rt under Ar. The reaction flask was evacuated under reduced pressure and back-filled with Ar three times. After the final evacuation, H_2 was charged and the reaction mixture was stirred vigorously under a H_2 balloon at rt for 3 h. The solids were removed by filtration through Celite and washed with Et_2O (30 mL). HCl (5.3 mL of a 2 M solution in Et_2O, 5.3 mmol, 5.0 eq) was added dropwise to the filtrate. The resulting solution was stirred at rt for 30 min. Then, the solvent was evaporated under reduced pressure to give pyrrolidine **47**·HCl (300 mg, 78%) as a red oil, IR (ATR) 2912, 1730 (C=O), 1437, 1177, 731 cm^{-1}; ^{1}H NMR (400 MHz, MeOH-*d*$_4$) δ 3.96-3.82 (m, 1H, NC*H*Me), 3.72 (s, 3H, OMe), 3.58-3.46 (m, 1H, NCH), 3.42-3.32 (m, 1H, NCH), 3.32-3.24 (m, 1H, CHCO$_2$), 2.38-2.23 (m, 2H, CH$_2$), 1.33 (d, J = 6.0 Hz, 3H, CH*Me*); ^{13}C NMR (100.6 MHz, MeOH-*d*$_4$) δ 173.5 (C=O), 58.7 (N*C*HMe), 52.8 (OMe), 47.6 (*C*HCO$_2$), 45.2 (NCH$_2$), 28.2 (CH$_2$), 13.9 (CH*Me*); MS (ESI) *m/z* 144 (M$^+$); HRMS *m/z* calcd for $C_7H_{14}NO_2$ M$^+$ 144.1019, found 144.1023 (–2.2 ppm error). Spectroscopic data for consistent with those reported in the literature.[23]

Lab Book Reference: PJ-02-04

Methyl 2-methyl-1-(methylsulfonyl)pyrrolidine-3-carboxylate S48

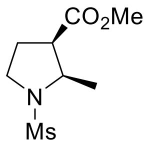

S48

Et_3N (0.23 mL, 1.68 mmol, 3.0 eq) was added dropwise to a stirred solution of **47**·HCl (100 mg, 0.56 mmol, 1.0 eq) in CH_2Cl_2 (10 mL) at rt under Ar. The resulting mixture was stirred at rt for 10 min and MsCl (0.13 mL, 1.68 mmol, 3.0 eq) was added dropwise. The resulting mixture was stirred at rt for 18 h. The reaction mixture was poured into water (50 mL) and extracted with CH_2Cl_2 (3 x 30 mL). The combined organics were dried ($MgSO_4$) and evaporated under reduced pressure to give the crude product as an orange solid. Purification by flash column chromatography on silica with 1:1 hexane-EtOAc as eluent gave *N*-sulfonamide pyrrolidine **S48** (90 mg, 73%) as a white solid, mp 83-86 °C; R_F (1:1 hexane-EtOAc) 0.23; IR (ATR) 2973, 1745 (C=O), 1316, 1147, 1130, 783 cm^{-1}; ^{1}H NMR (400 MHz, $CDCl_3$) δ

4.17 (dq, J = 13.0, 6.5 Hz, 1H, NC*H*Me), 3.72 (s, 3H, OMe), 3.60-3.50 (m, 1H, NCH), 3.30 (ddd, J = 10.0, 10.0, 7.0 1H, NCH), 3.15-3.06 (m, 1H, C*H*CO_2Me), 2.85 (s, 3H, SO_2Me), 2.41-2.30 (m, 1H, CH), 2.18-2.10 (m, 1H, CH), 1.17 (d, J = 6.5 Hz, 3H, CH*Me*); ^{13}C NMR (100.6 MHz, $CDCl_3$) δ 171.2 (C=O), 56.5 (N*C*HMe), 52.2 (OMe), 47.9 (*C*HCO_2Me), 46.9 (NCH_2), 36.7 (SO_2Me), 26.0 (CH_2), 18.2 (CH*Me*); MS (ESI) *m/z* 244 $(M + H)^+$; HRMS *m/z* calcd for $C_8H_{15}NO_4S$ $(M + Na)^+$ 244.0614, found 244.0617 (–3.8 ppm error).

Lab Book Reference: PJ-02-57

Structure confirmed by small molecule X-Ray crystallography (CCDC 1995342):

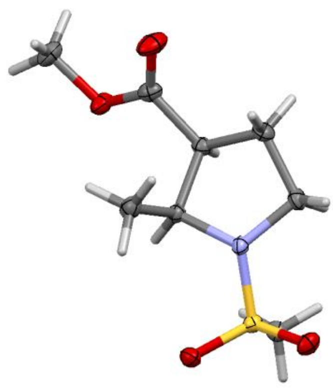

Methyl-4-methyl-1-tosyl piperidine-3-carboxylate S49

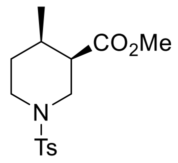

S49

HCl (0.78 mL of a 2 M solution in Et_2O, 1.56 mmol, 10.0 eq) was added dropwise to *N*-Boc piperidine **S50** (40 mg, 0.15 mmol, 1.0 eq, synthesised by Boc protection of piperidine **6d**) at rt under Ar. The resulting solution was stirred at rt for 16 h. Then, the solvent was evaporated under reduced pressure to give the hydrochloride salt (33 mg). Et_3N (0.065 mL, 0.47 mmol, 3.0 eq) was added dropwise to a solution of the hydrochloride salt (33 mg, 0.95 mmol, 1.0 eq) and TsCl (6 mg, 0.052 mmol, 0.1 eq) in CH_2Cl_2 (5 mL) under Ar. The reaction mixture was stirred at rt for 16 h. The reaction mixture was poured into water (10 mL) and extracted with CH_2Cl_2 (3 x 10 mL). The combined organics were dried (Na_2SO_4) and evaporated under reduced pressure to give the crude product as a yellow oil. Purification by flash column chromatography on silica with 8:2 hexane-EtOAc as eluent gave *N*-tosyl piperidine **S49** (41 mg,

84%) as a white solid, mp 65-67 °C; R_F (0.12, 8:2 hexane-EtOAc); IR (ATR) 2929, 1726 (C=O),1340, 1161, 920 cm^{-1}; ^1H NMR (400 MHz, $CDCl_3$) δ 7.65 (d, *J* = 8.0 Hz, 2H, Ar), 7.32 (d, *J* = 8.0 Hz, 1H, Ar), 3.66 (s, 3H, OMe), 3.50 (ddd, *J* = 11.5, 3.0, 1.0 Hz, 1H, NCH), 3.31 (dddd, *J* = 11.5, 4.5, 4.5, 1.5 Hz, 1H, NCH), 2.86-2.73 (m, 2H, NCH and $CHCO_2$), 2.66 (ddd, *J* = 11.5, 11.5, 3.0 Hz, 1H, NCH), 2.43 (s, 3H, Ar*Me*), 2.26-2.17 (m, 1H, C*H*Me), 1.88-1.78 (m, 1H, CH), 1.70-1.62 (m, 1H, CH), 0.78 (d, *J* = 7.0 Hz, 3H, CH*Me*); ^{13}C NMR (100.6 MHz, $CDCl_3$) δ 172.6 (C=O), 143.7 (*ispo*-Ar), 133.4 (*ipso*-Ar), 129.9 (Ar), 127.8 (Ar), 51.8 (OMe), 44.6 ($CHCO_2$), 43.3 (NCH_2), 42.0 (NCH_2), 30.7 (NCH_2), 28.7 (*C*HMe), 21.7 (Ar*Me*), 13.9 (CH*Me*); MS (ESI) *m/z* 334 $(M + Na)^+$; HRMS *m/z* calcd for $C_{15}H_{21}NO_4S$ $(M + Na)^+$ 334.1083, found 334.1089 (–1.4 ppm error).

Lab Book Reference: PJ-05-5

Structure confirmed by small molecule X-Ray crystallography (CCDC 1830138):

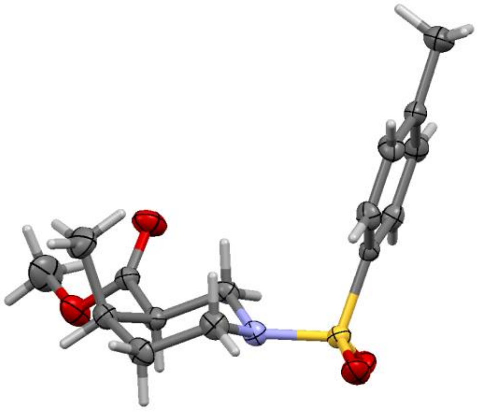

1.4. Proof of Stereochemistry

Fragments 2f and 2g: The NH methyl ester **6c** which is the precursor to these two fragments is a known compound.[8]

Fragment 2h: Stereochemistry proven by X-ray crystallography of *N*-Ts methyl ester **S49**.

Fragments 2i and 2j: Stereochemistry proven by ^{3}J values in compounds **S6** and **S7**. Also establishes the stereochemistry of **20a-20c**.

Fragments 2k and 2l: Stereochemistry proven by X-ray crystallography of **2l**.

Fragments 2m, 2n, 2o and 2p: Stereochemistry proven by X-ray crystallography of **19d** and **19k**; the NH methyl ester **6a** which is the precursor to **2m** and **2n** is a known compound;[12] stereochemistry proven by ^{3}J values in **S17**. Also establishes stereochemistry of fragments **19a-19k**.

Fragment 2q: Stereochemistry proven by synthesis of the corresponding *N*-Ts methyl ester which is a known compound.[24]

Fragments 2r and 2s: Stereochemistry proven by synthesis of the corresponding *N*-Cbz derivatives of **S24** and **S25** which are both known compounds.[25]

Fragment 1f: Sterechemistry assumed from well-precedented similar reductions.[26]

Fragments 11a, 11b, 11c and 11d: Stereochemistry proven by X-ray crystallography of **11d**.

Fragment 1g: Compound **13**[14] and fragment **1g**[16] are both known compounds.

Fragments 1h and 1i: Stereochemistry assumed from *cis*-hydrogenation.

Fragments 1j, 1k and 1l: Stereochemistry proven by X-ray crystallography of *N*-Ms derivative **S48**.

2. Library Analysis

2.1. General

Shape analysis

3-Dimensional structures were generated Pipeline Pilot 16.5.0.143, 2016, Accelrys Software Inc.

Prior to conformer generation a wash step was performed, which involved stripping salts and ionising the molecule at pH 7.4. SMILES strings were converted to their canonical representation and the original stereochemistry at each chiral centre was recorded. Any stereocentre created during the ionisation would have undefined stereochemistry. A SMILES files was written that contained all possible stereoisomers of the molecule. Conformers were generated using Catalyst with the BEST conformational analysis method and relative stereochemistry. Catalyst was run directly on the server and not through the built-in Conformation Generator component. The maximum relative energy threshold was left at the default 20 kcal mol^{-1} and a maximum of 255 conformers were generated for each compound. The aim of this was to give the best possible coverage of conformational space. The resulting conformations from Catalyst were read and only those where the stereochemistry matched the original molecule or its enantiomer were kept. These were then all standardised to the original stereochemistry by mirroring the coordinates of the enantiomers. Duplicate conformations were filtered with a RMSD threshold of 0.1. Each conformation was minimised using 200 steps of Conjugate Gradient minimisation with an RMS gradient tolerance of 0.1. This was performed using the CHARMm forcefield with Momany-Rone partial charge estimation and a Generalised Born implicit solvent model. After minimisation, duplicates were filtered again with a RMSD threshold of 0.1.

Generated conformations were used to generate the three Principal Moments of Inertia (I1, I2 and I3) which were then normalised by dividing the two lower values by the largest (I1/I3 and I2/I3) using Pipeline Pilot built-in components.

Principal moments of inertia (PMI) about the principal axes of a molecule were calculated according to the following rules:

1. The moments of inertia are computed for a series of straight lines through the centre of mass.

2. Distances are established along each line proportional to the reciprocal of the square root of I on either side of the centre of mass. The locus of these distances forms an ellipsoidal surface. The principal moments are associated with the principal axes of the ellipsoid.

Cumulative PMI analysis was performed in the following way. The ΣNPR (ΣNPR = NPR1 + NPR2) was calculated for each conformer and then the mean ΣNPR for each fragment was obtained. This value was used as a measure of the three-dimentionality of each fragment. The cumulative percentage of fragments within a defined distance from the rod-disc axis (ΣNPR) was calculated and plotted.

Molecular properties

Molecular Weight (MW), heavy atom count (HAC), lipophilicity (SlogP), number of hydrogen bond donors (HBD), number of hydrogen bond acceptors (HBA), rotatable bond count (RBC), fraction of sp^3 carbons (Fsp^3) and topological polar surface area (TPSA) were calculated using RDKit v3.4 in KNIME v3.5.2. Prior to calculation, salts were stripped and canonical SMILES were generated. ClogP values were caluculated using Daylight/BioByte ClogP v4.3.

Solubility

Fragment solubility was assessed in a 20 mM sodium phosphate buffer (pH 7.48) (1 mM concentration of fragment). Fragment solubility was determined by ^{1}H NMR spectroscopy with the aid of a reference compound, 4,4-dimethyl-4-silapentane-1-sulfonic acid (DSS), of known concentration. Integrals of the fragment were compared with those of DSS and the fragment concentration was calculated using the following formula: $C_x = I_x/I_{cal} \times N_{cal}/N_x \times C_{cal}$ where I, N, and C are the integral area, number of nuclei, and concentration of the fragment (x) and the calibrant (cal), respectively.

Stability

Fragment stability in solution was assessed in both a 20 mM sodium phosphate buffer (pH 7.48) (1 mM concentration of fragment) at 1 h and 24 h and in DMSO (2 mM concentration of fragment) at 24 h and 6 weeks using ^{1}H NMR spectroscopy.

2.2. Commercial Fragment Library Details

The following commercially available fragment libraries were analysed:

Maybridge RO3 Diversity Fragment Library: Core Set (1000 compounds, https://www.maybridge.com/portal/alias__Rainbow/lang__en/tabID__230/DesktopDefault.aspx; last accessed 18/10/2017)

Chembridge Fragment Library (9067 compounds, https://www.chembridge.com/screening_libraries/fragment_library/; last accessed 23/04/2018)

Enamine RO3 Fragment Library (69583 compounds, https://enamine.net/index.php?option=com_content&task=view&id=208; last accessed 19/03/2018)

Life Chemicals 3D Fragment Library (1377 compounds, https://lifechemicals.com/screening-libraries/fragment-libraries; last accessed 21/8/2018)

ChemDiv 3D FL Fragments Library (4460 compounds, http://www.chemdiv.com/3d-fl-fragments-library/; last accessed 10/01/2019)

Enamine 3D Shape Diverse Fragment Library (1200 compounds, https://enamine.net/index.php?option=com_content&view=article&id=640&Itemid=716; last accessed 10/01/2019)

2.3. Relationship between Fsp^3 and ΣNPR for Commecial Libaries

The ΣNPR and Fsp^3 were calculated for ground state conformers of a random 1000 compounds from each of the six commercially available libraries detailed in section 2.2. R-squared values (Fig S1 – S6) indicate little correlation between Fsp^3 and three-dimentionality as measured by ΣNPR.

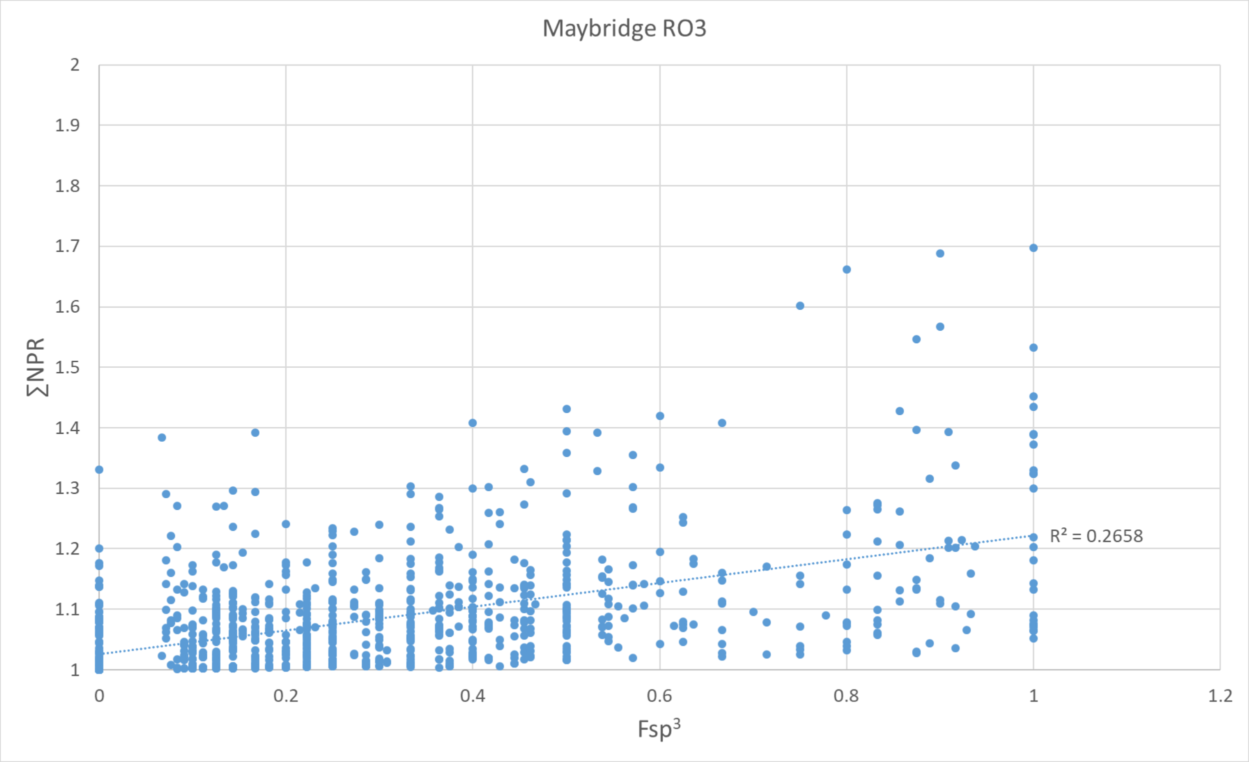

Fig S1. Plot of ΣNPR vs. Fsp^3 for the Maybride RO3 Diversity Fragment Library

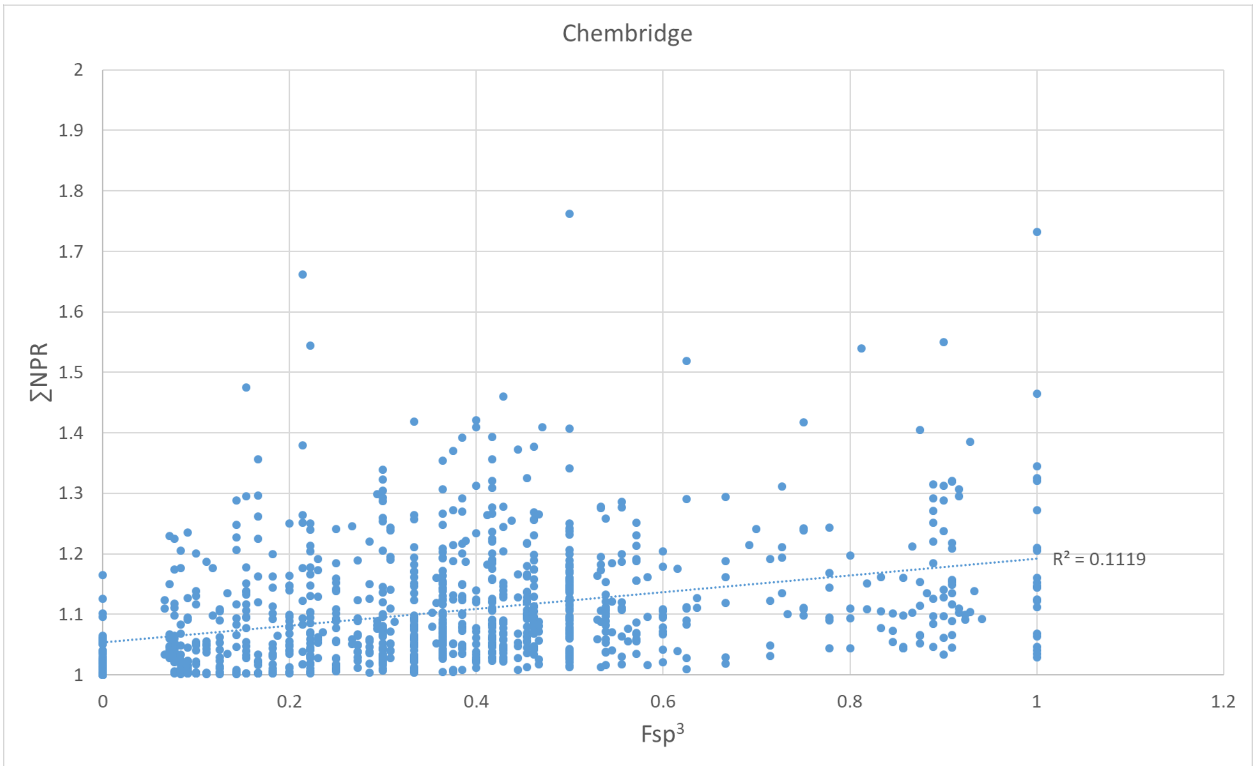

Fig S2. Plot of ΣNPR vs. Fsp^3 for the Chembridge Fragment Library

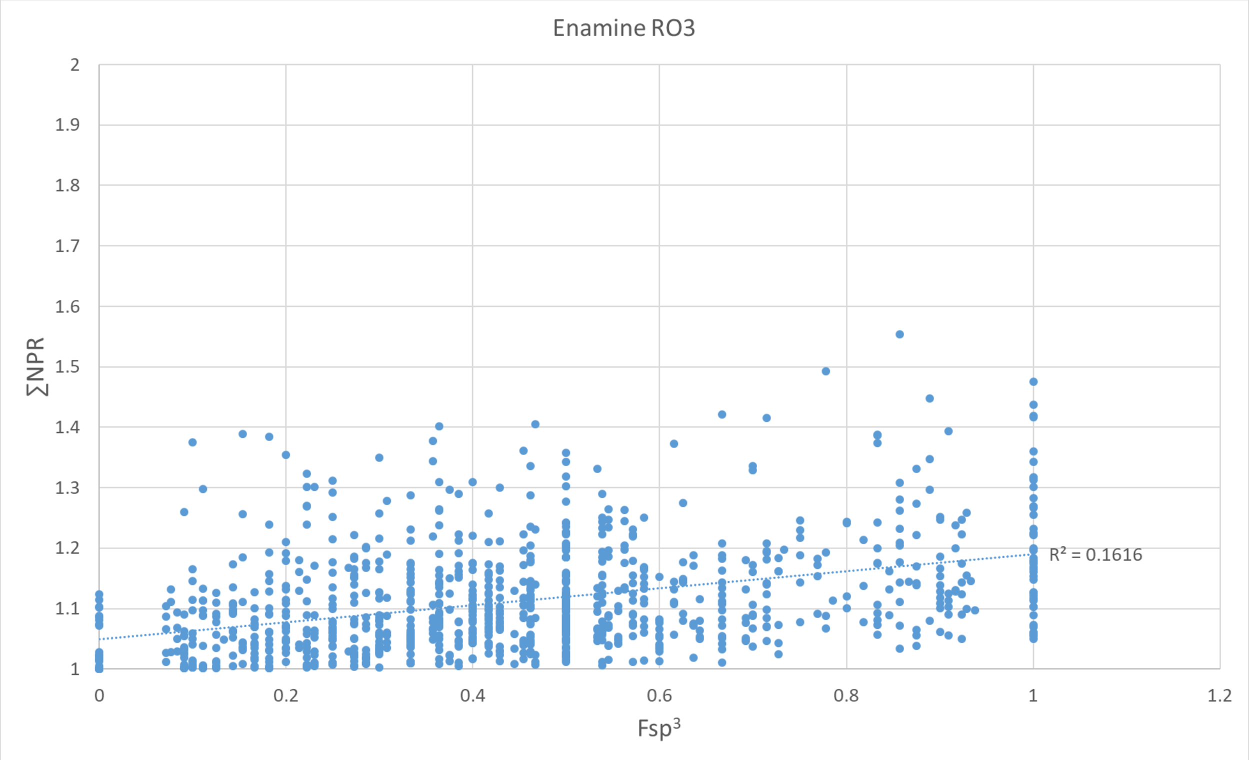

Fig S3. Plot of ΣNPR vs. Fsp^3 for the Enamine RO3 Fragment Library

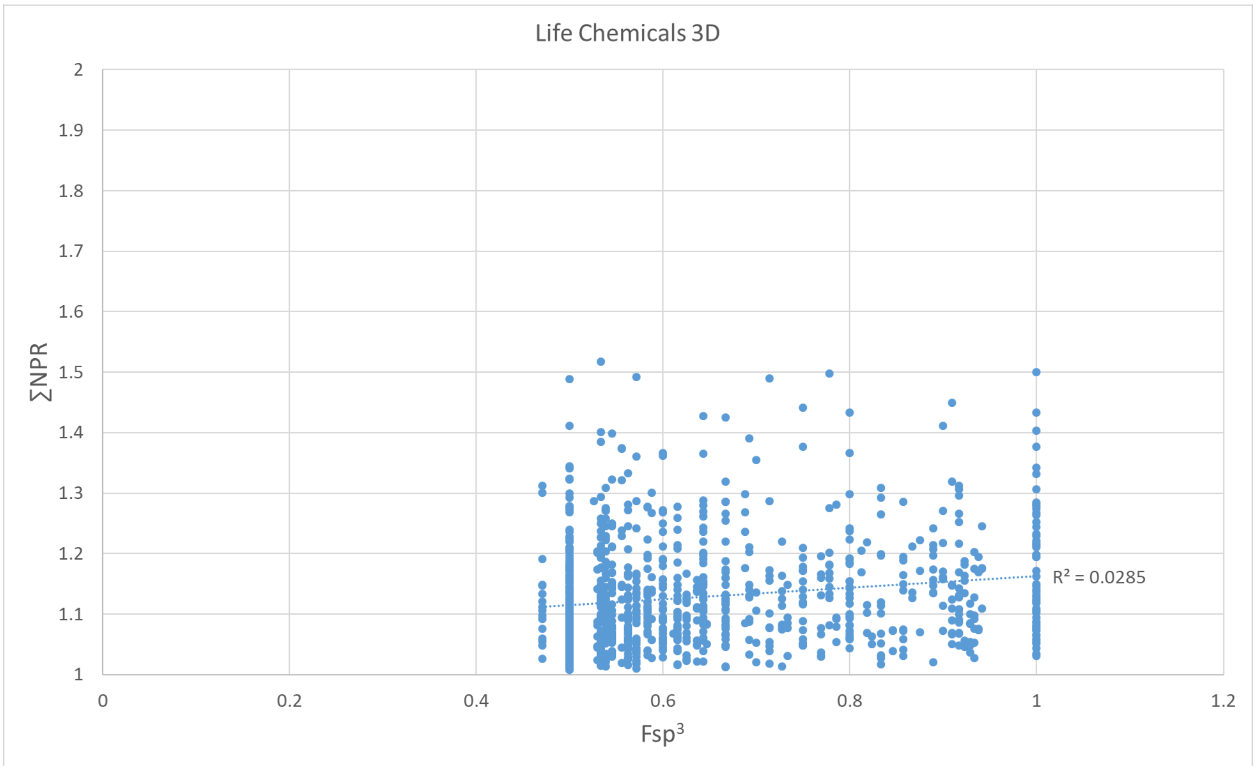

Fig S4. Plot of ΣNPR vs. Fsp^3 for the Life Chemicals 3D Fragment Library

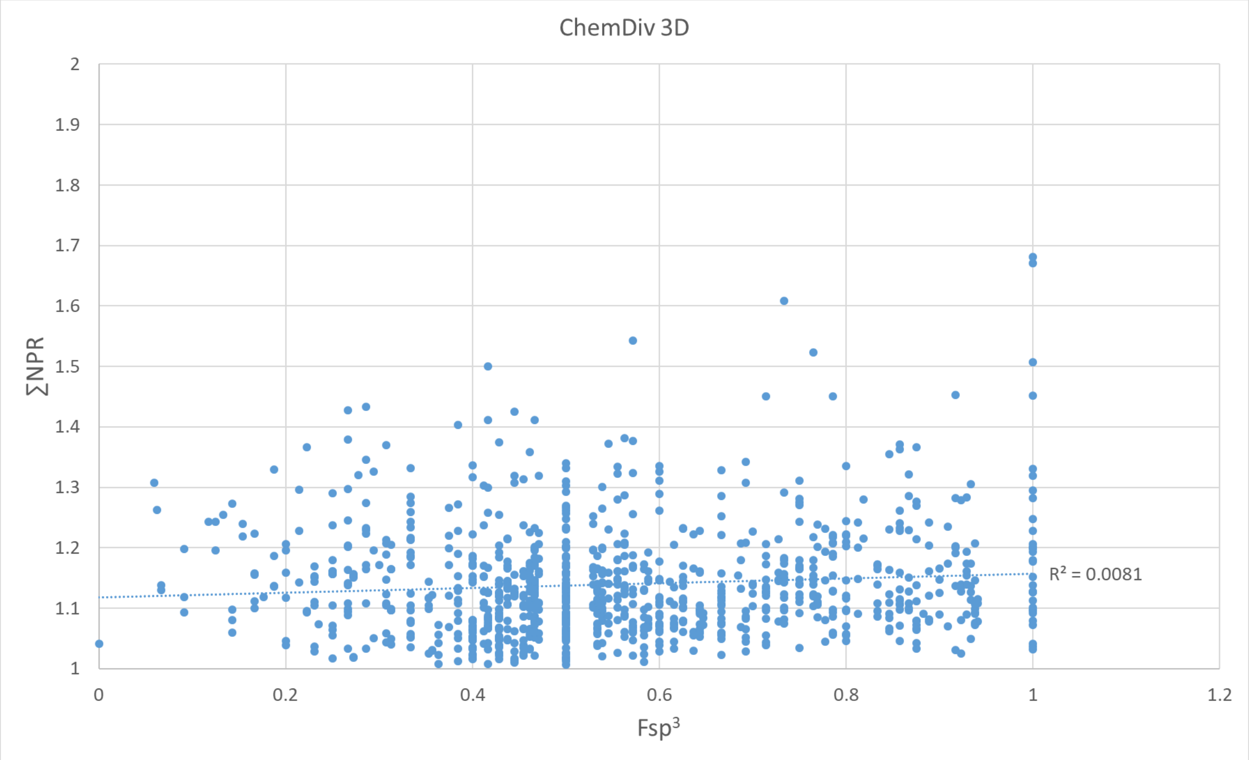

Fig S5. Plot of ΣNPR vs. Fsp^3 for the ChemDiv 3D FL Fragments Library

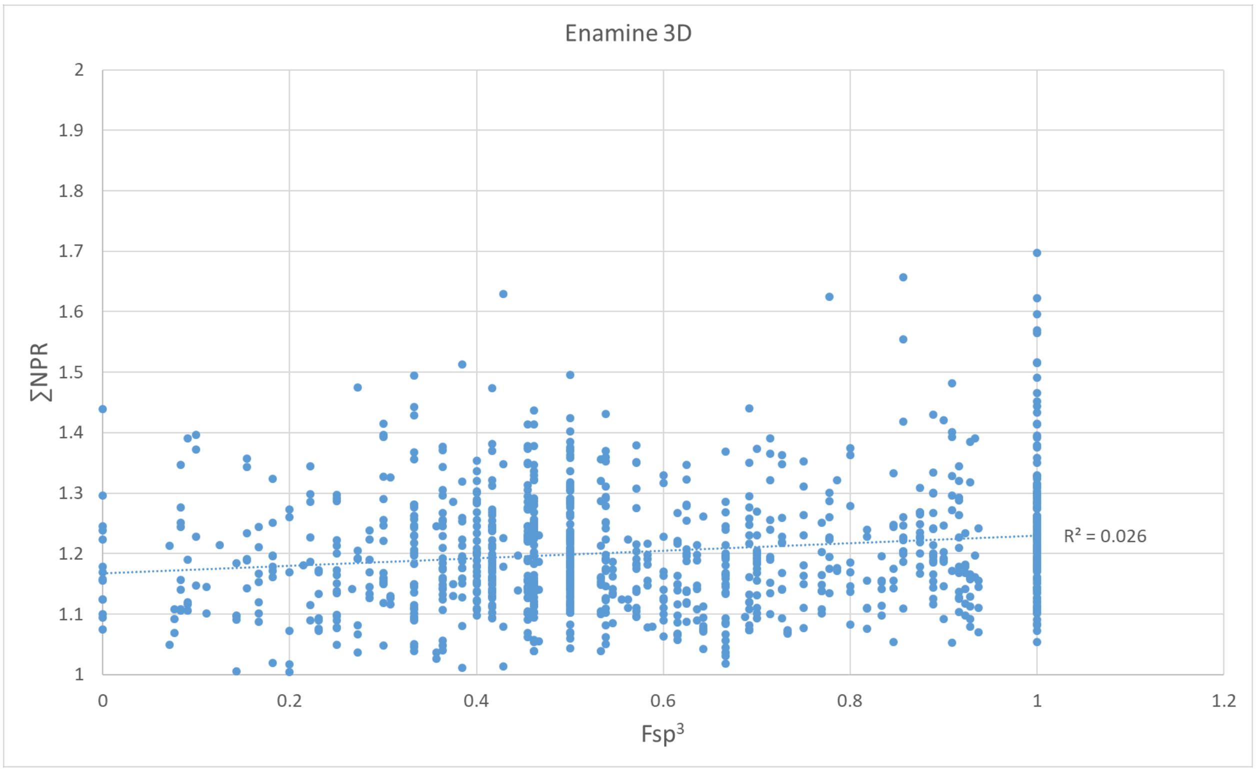

Fig S6. Plot of ΣNPR vs. Fsp^3 for the Enamine 3D Shape Diverse Fragment Library

2.4 Cumulative PMI analysis of commercially available libraries

Cumulative PMI analysis of the six commercial fragment libraries (section 2.2) was performed. Only ground state conformers were analysed. The ΣNPR values used are the same as those calculated for analysis in section 2.3.

Two ‘3D’ fragment libaraies were selected based upon Fsp^3 cut-offs – Life Chemicals 3D ($Fsp^3 > 0.47$) and ChemDiv3D ($Fsp^3 > 0.4$) are only marginally more 3D tham libraries with no Fsp^3 restrictions (Maybridge RO3, Chembridge RO3 and Enamine RO3). The Enamine 3D library is designed around PMI restrictions ($NPR1 \geq 0.15$; $NPR2 \geq (1.15 - NPR1)$) and is significantly more 3D (Fig S7).

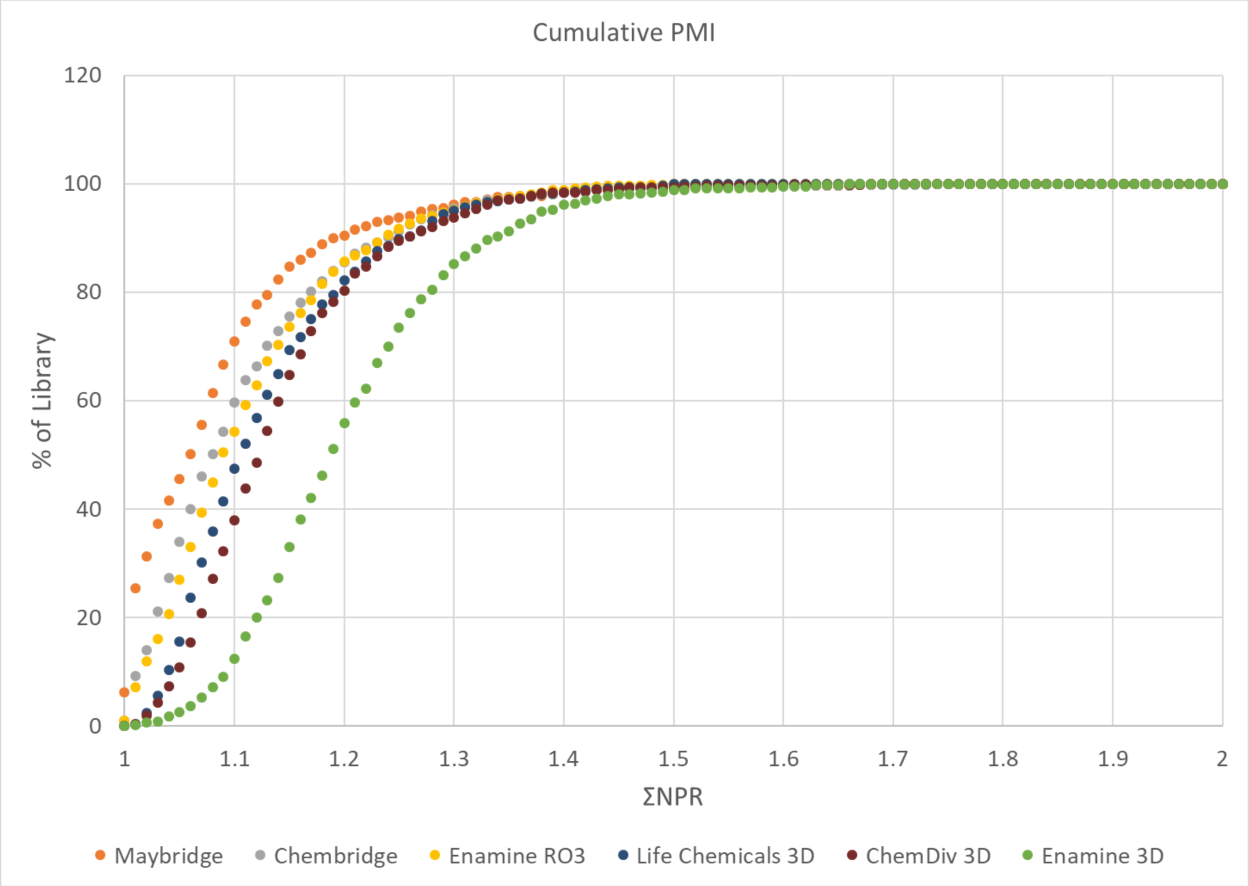

Fig S7. Plot of ΣNPR vs. Fsp^3 for the Enamine 3D Shape Diverse Fragment Library

2.5 Selected Fragments

Figure S8 shows the 33 selected pyrrolidine and piperidine fragments. Pyrrolidines **S51** and **S52** were not synthesised.

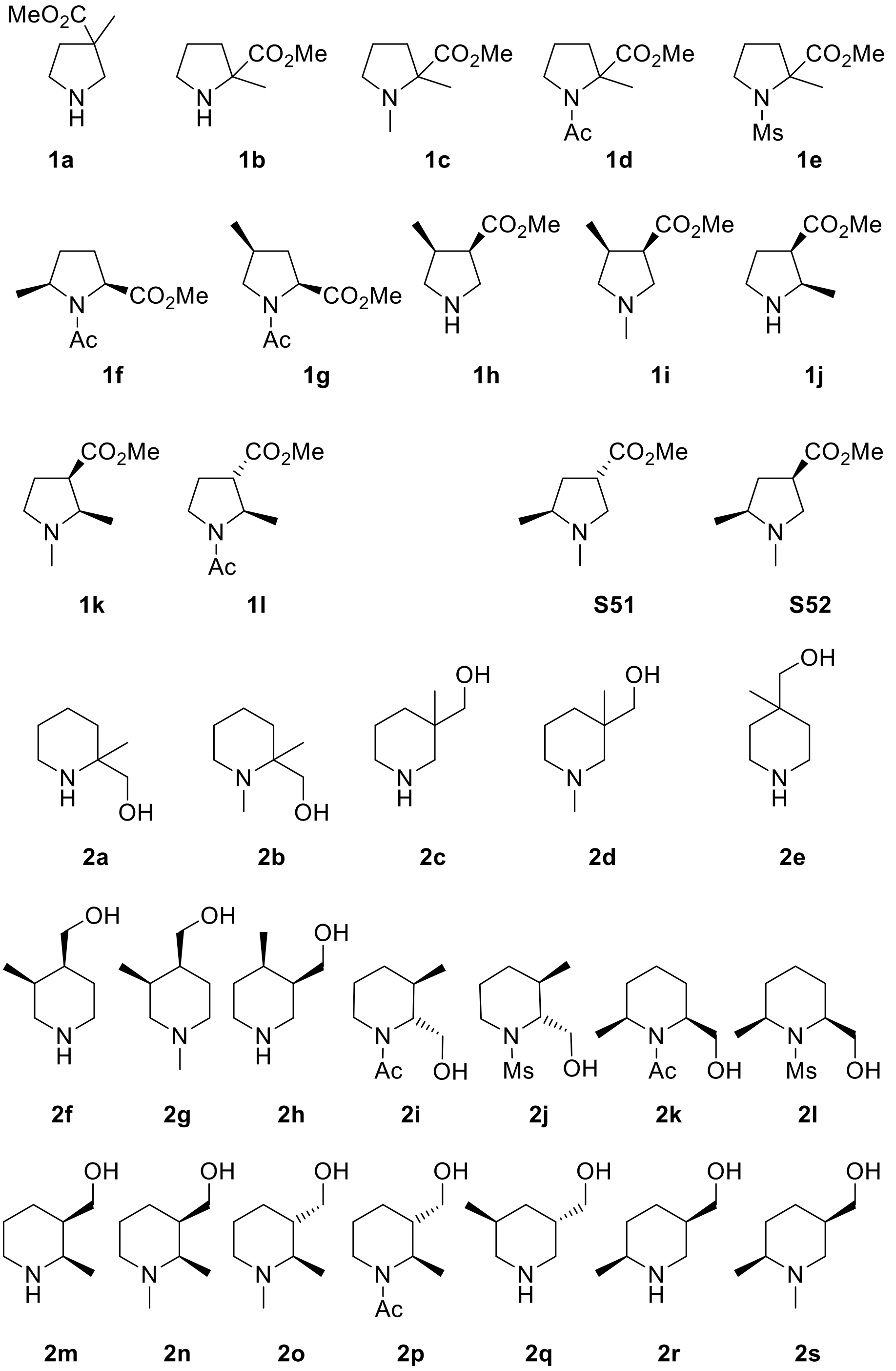

Figure S8. Structrues of the 33 selected fragments

2.6 Synthesised Fragments

Figure S9 shows the 56 synthesised pyrrolidine and piperidine fragments.

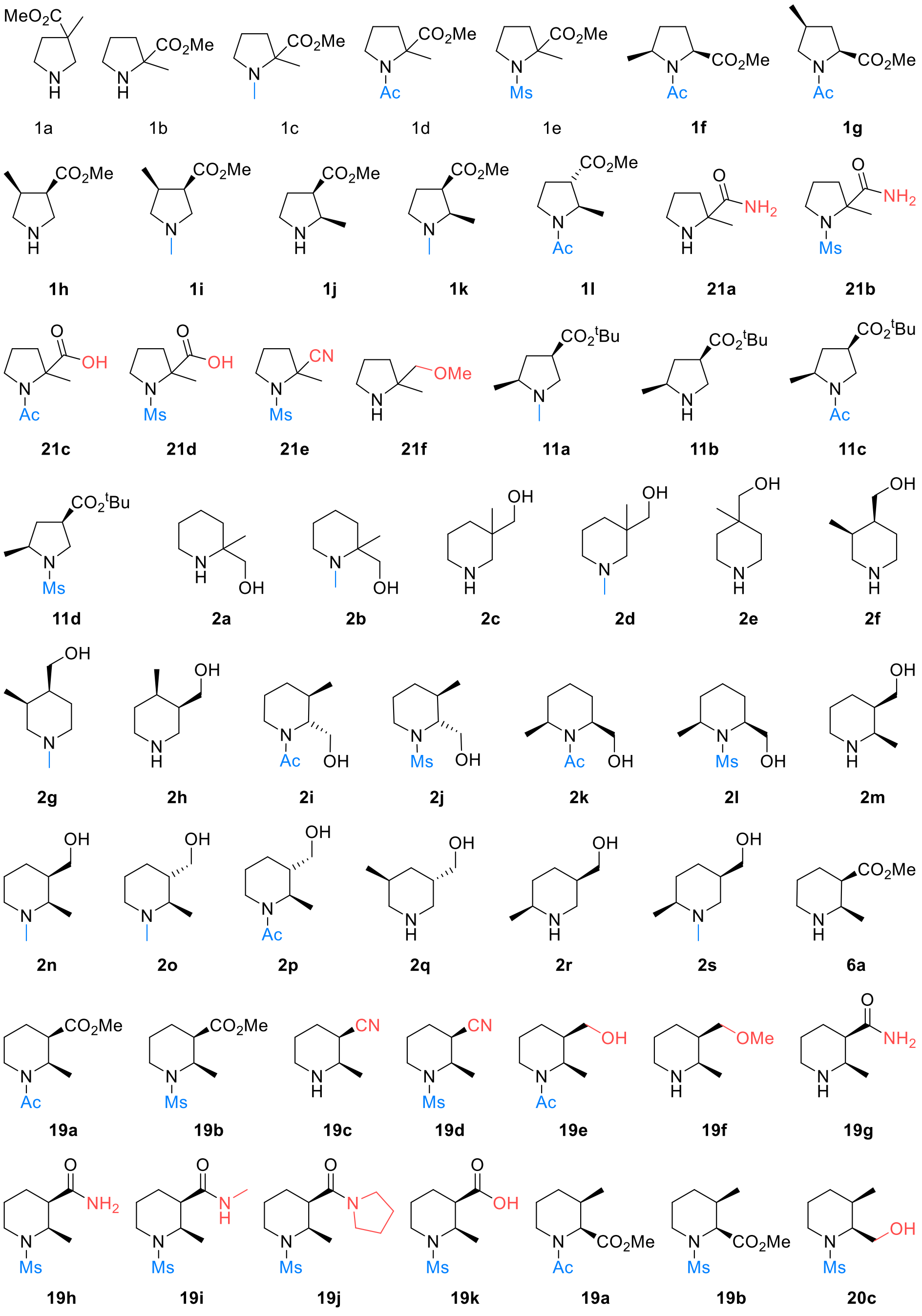

Figure S9. Structrues of the 56 synthesised fragments

2.7 Molecular Properties of Synthesised Fragments and Commercial Libraries

Molecular properties were calculated for all compounds in the libraries (Fig S10-S17). ClogP data was available for only the Maybridge RO3 and Chembridge RO3 libraries, hence SlogP was calculated for all libraries detailed in section 2.2.

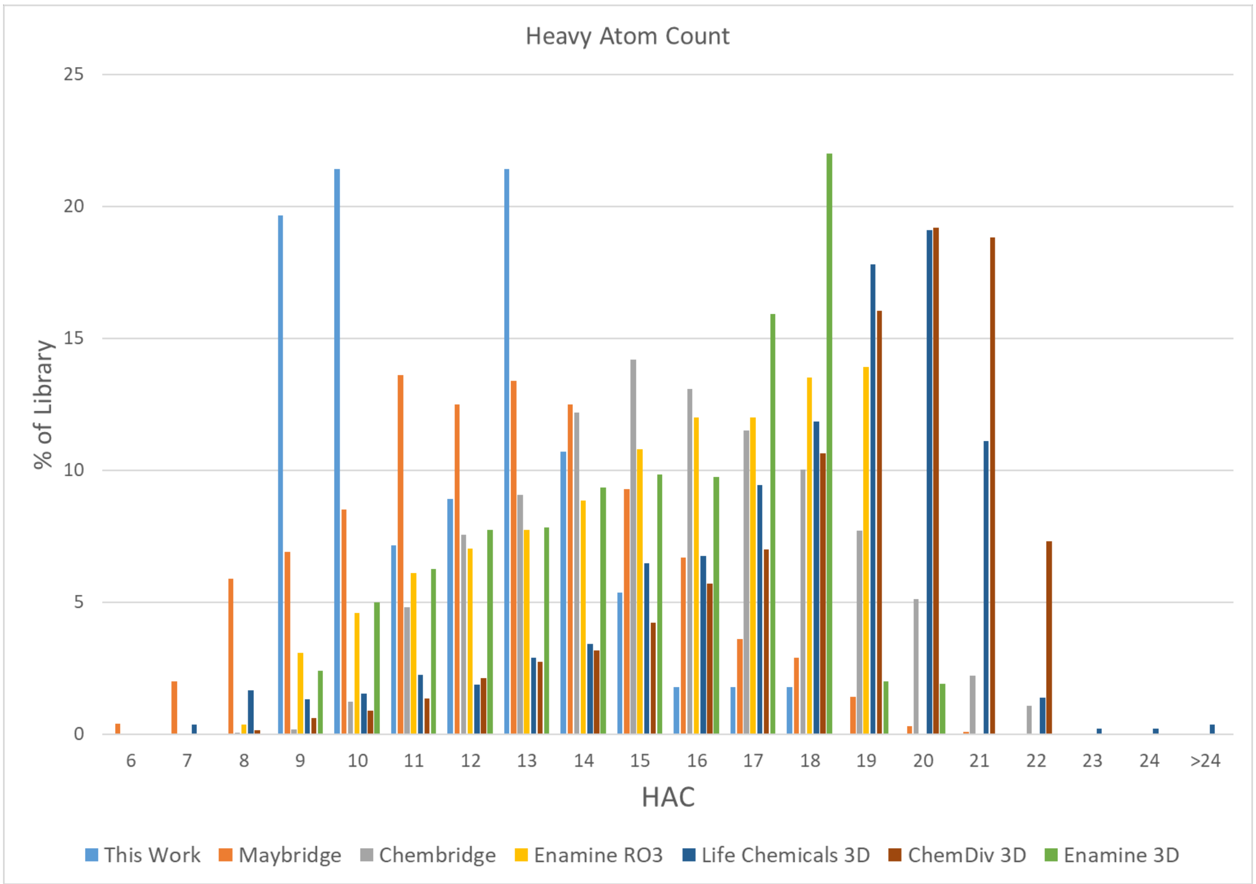

Fig S10. Heavy atom count distribution of this work and commercial libraries

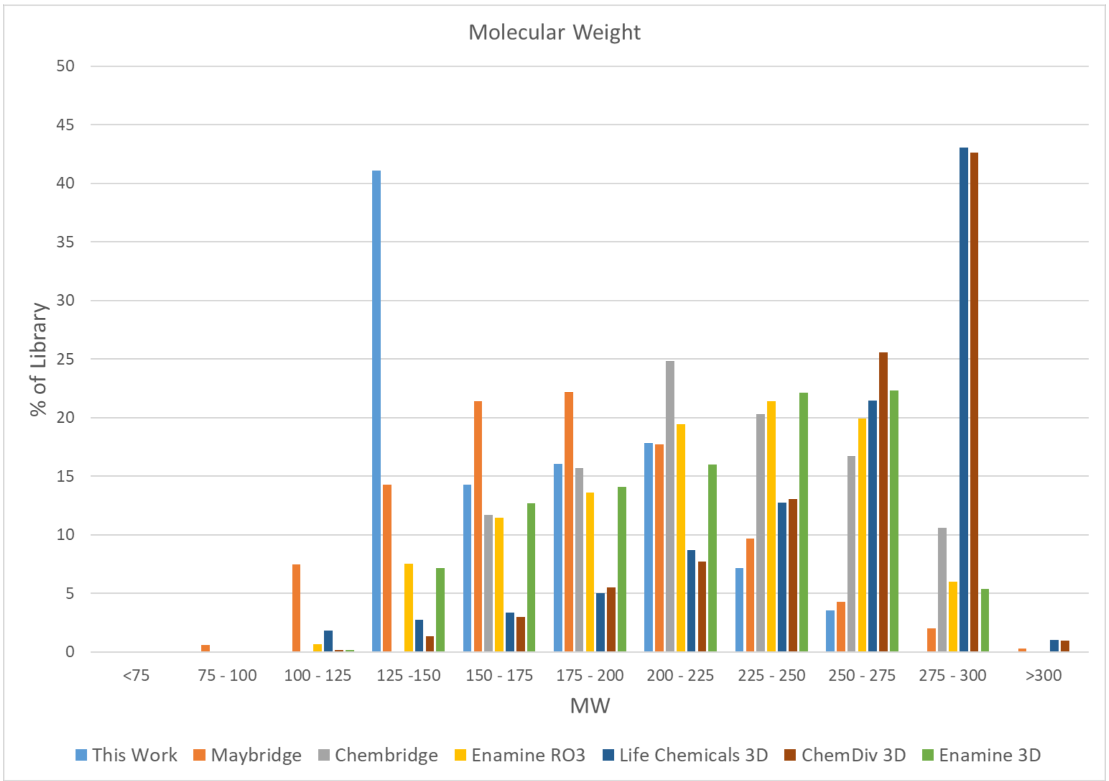

Fig S11. Molecular weight distribution of this work and commercial libraries

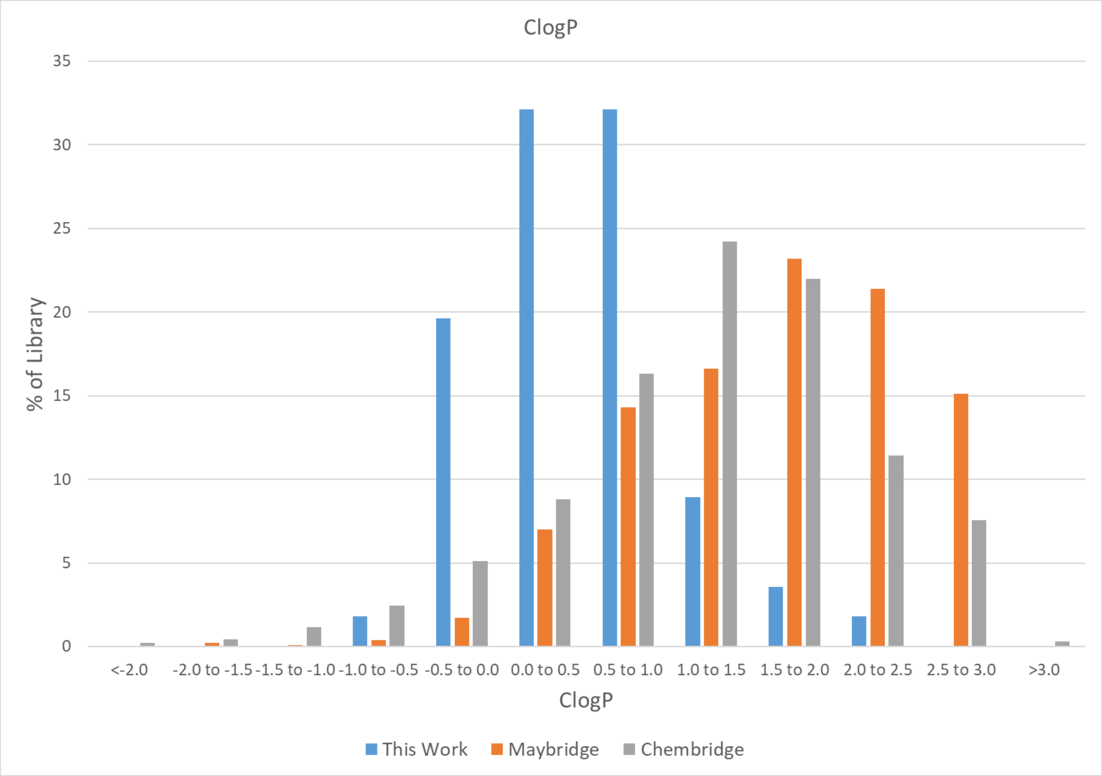

Fig S12. ClogP distribution of this work and commercial libraries

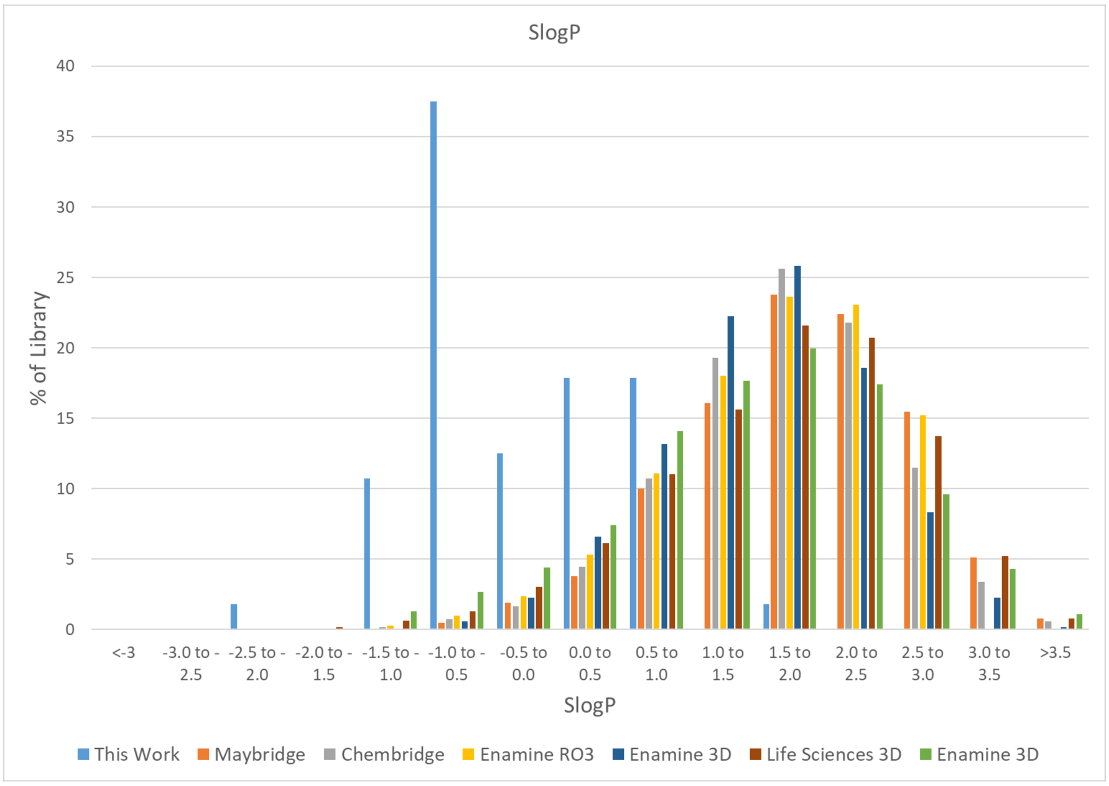

Fig S13. SlogP distribution of this work and commercial libraries

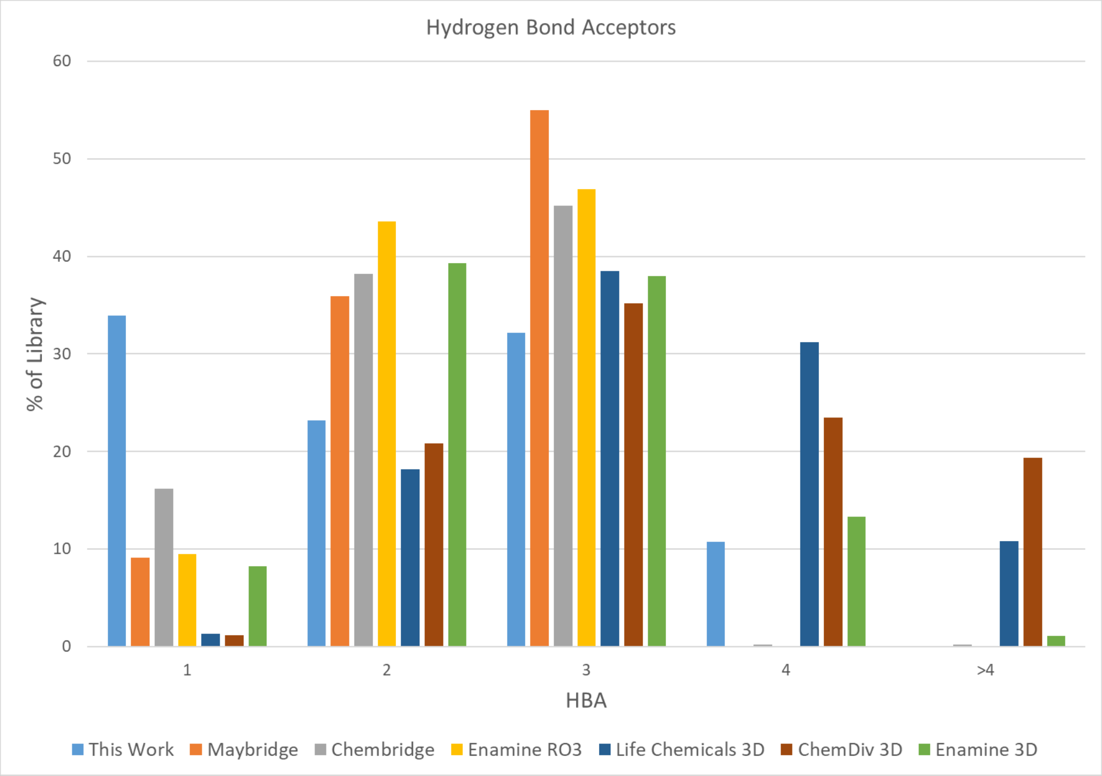

Fig S14. Hydrogen bond acceptor distribution of this work and commercial libraries

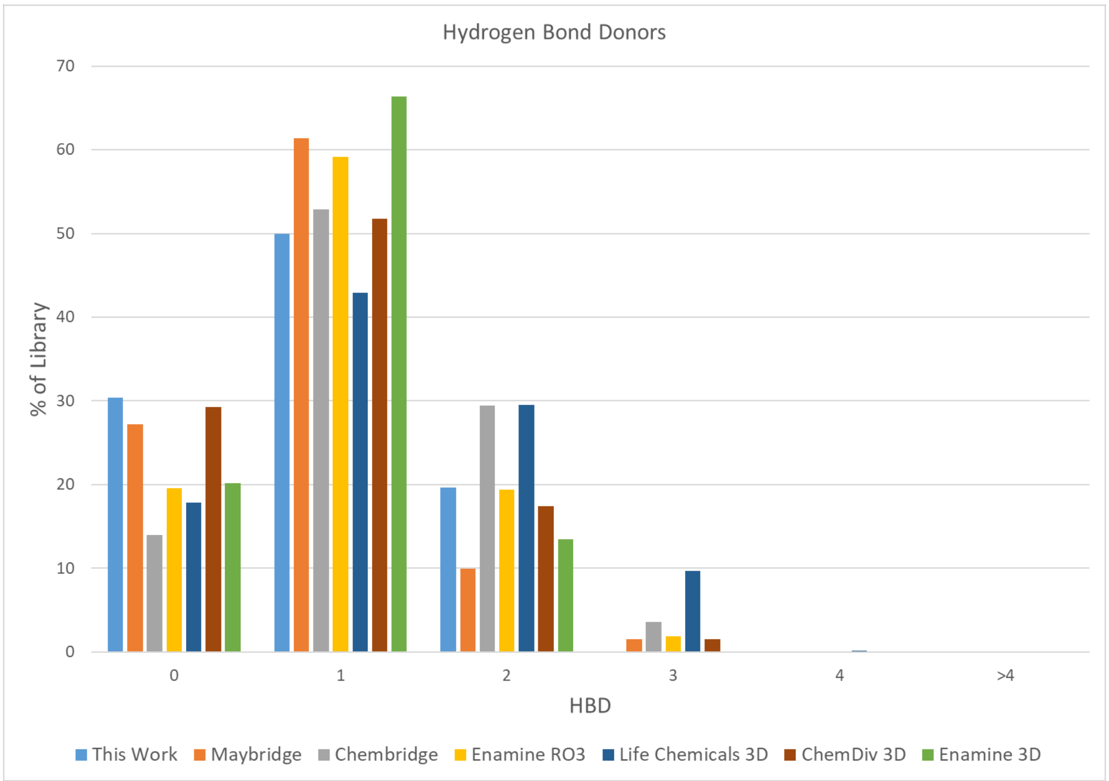

Fig S15. Hydrogen bond donor distribution of this work and commercial libraries

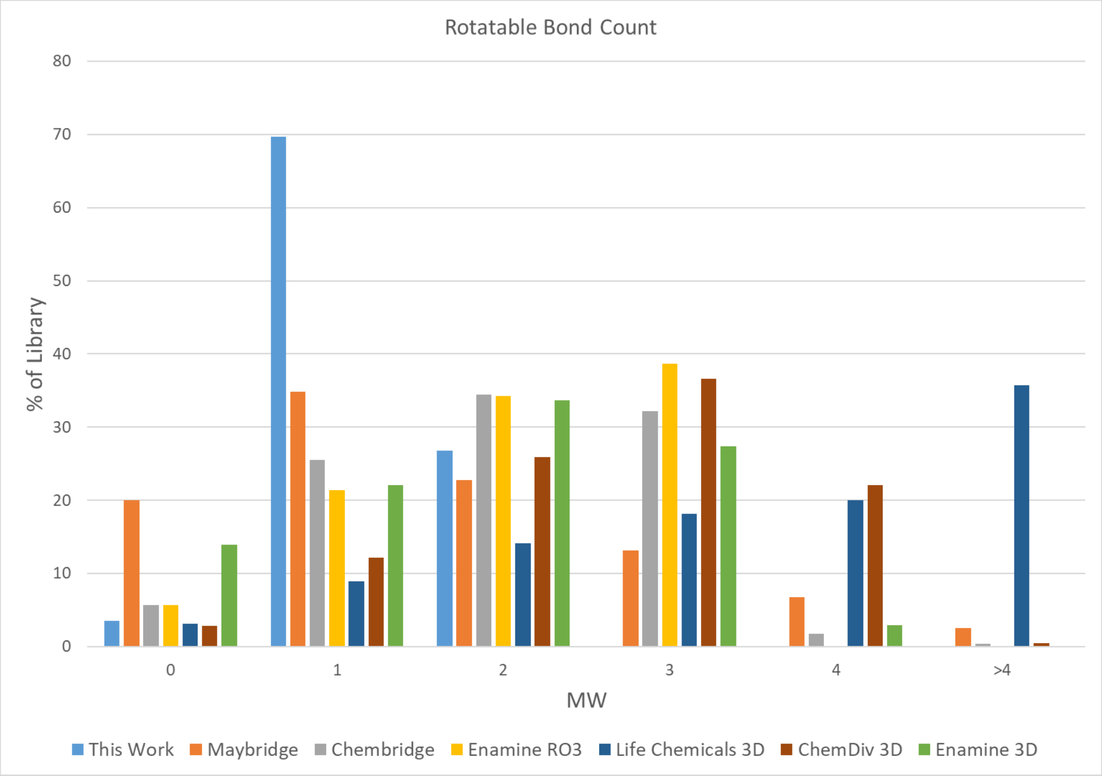

Fig S16. Rotatable bond count distribution of this work and commercial libraries

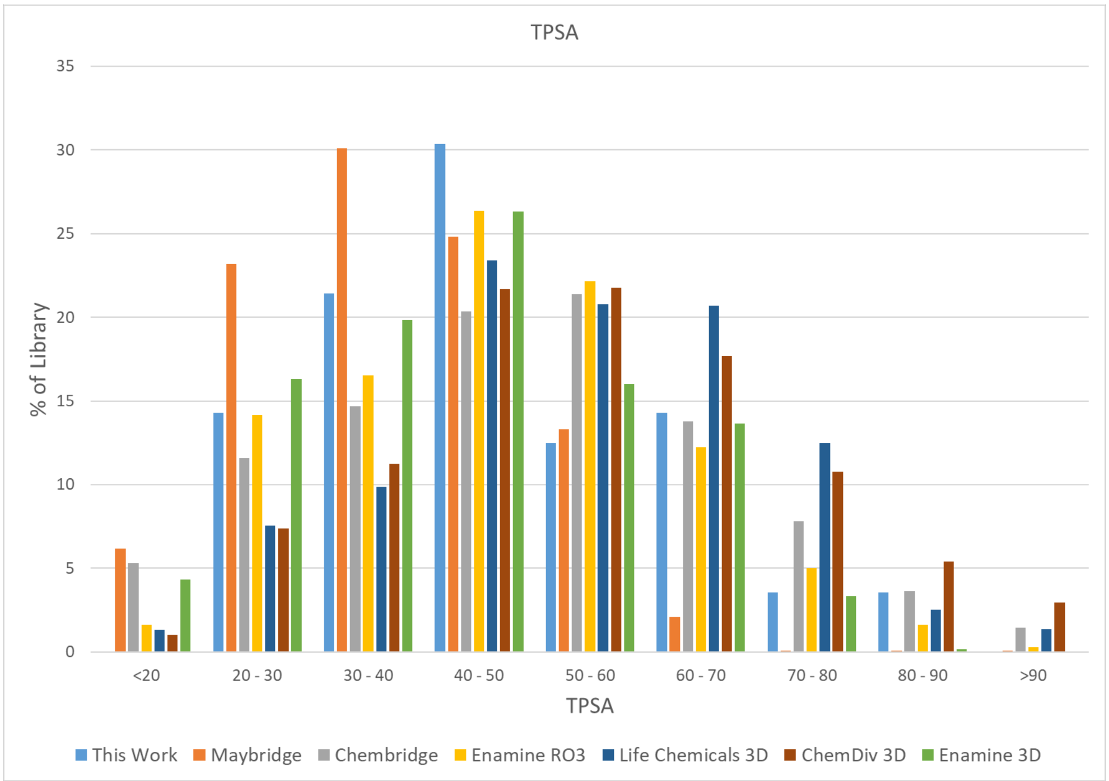

Fig S17. TPSA distribution of this work and commercial libraries

2.8 Calculated Boltzmann Populations of Molecular Mechanics-Computed Conformers

Figure S18 shows the PMI plots from Figures 1 and 3 with the points coloured by their calculated Boltzmann populations.

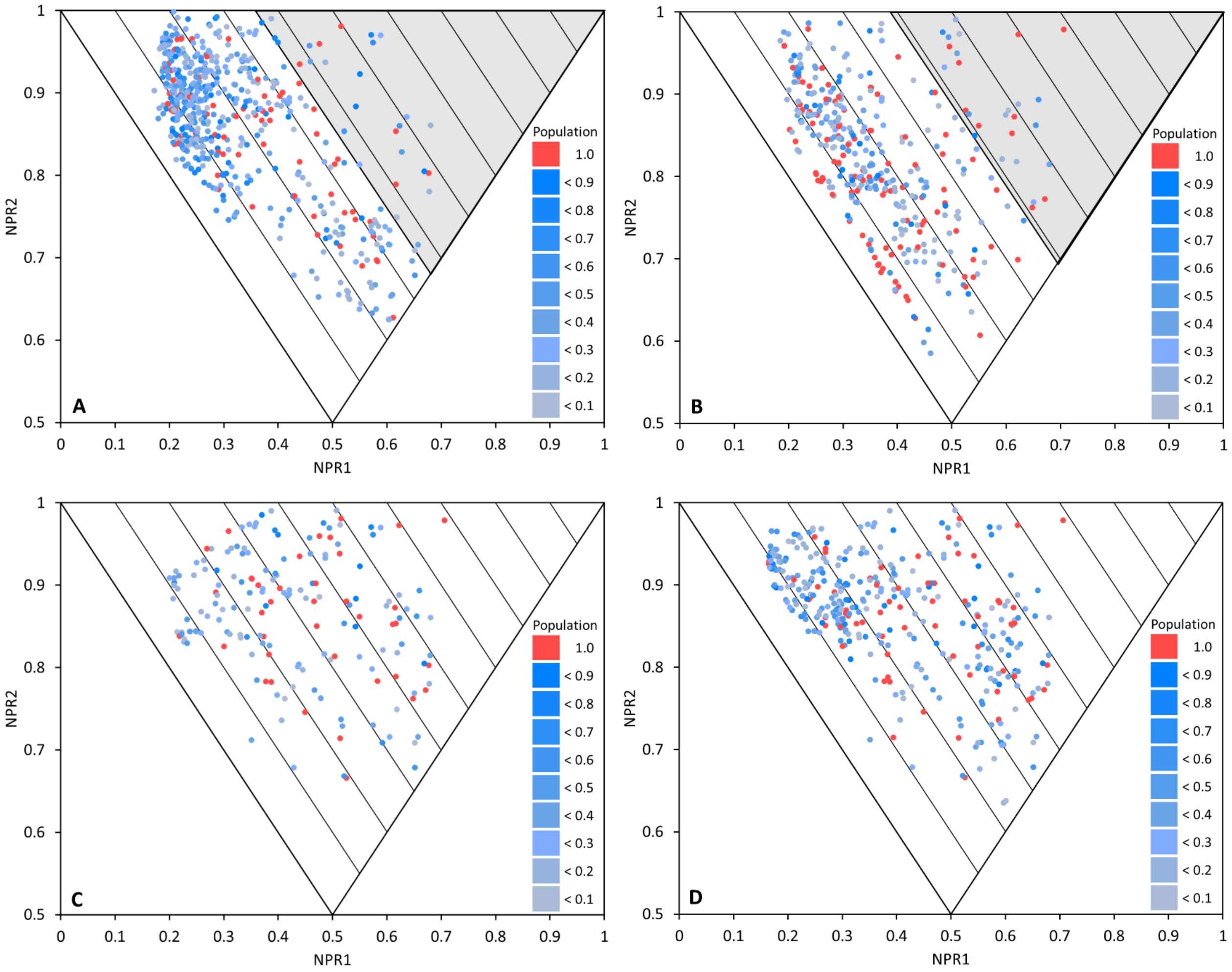

Fig S18. PMI plots couloured by Boltzmann populations. A: Conformers of pyrrolidine scaffold **1** – see Fig 1A. B: Conformers of piperidine scaffold **2** – see Fig 1B. C: Conformers of 33 selected fragments – see Fig 1C. D: PMI plot of the final fragment collection – see Fig 3A.

3. ^{1}H and ^{13}C NMR Spectra

400 MHz ^{1}H NMR spectrum; 100.6 MHz ^{13}C NMR spectrum; $CDCl_3$

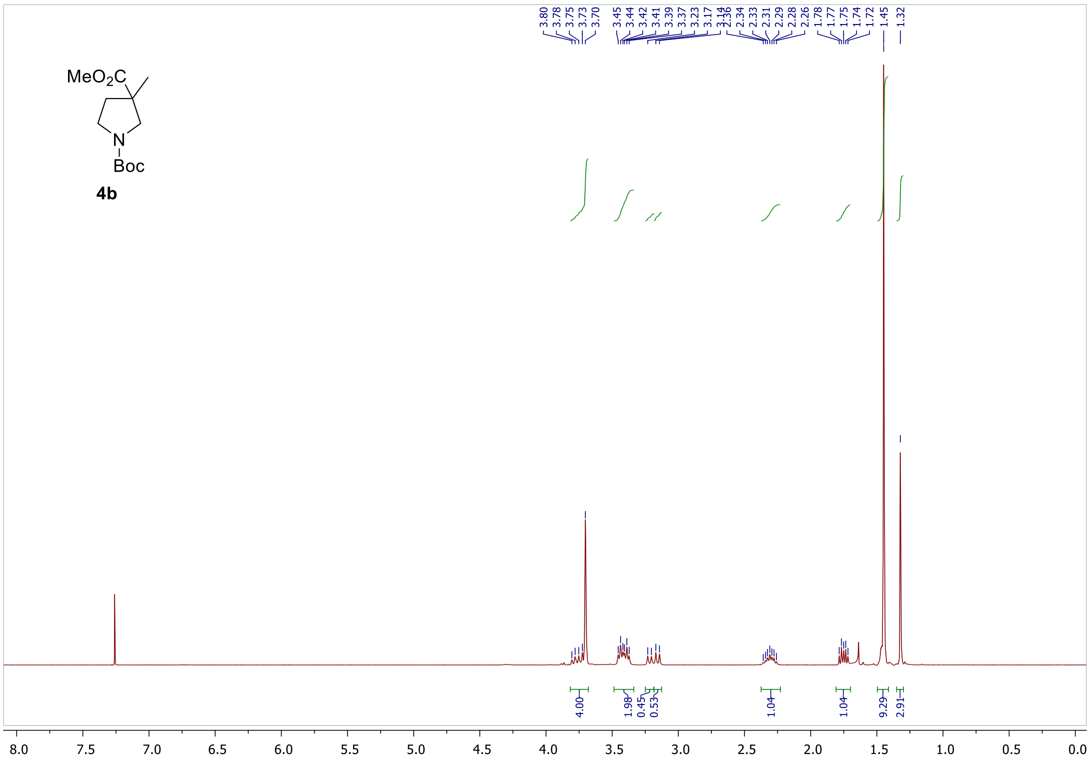

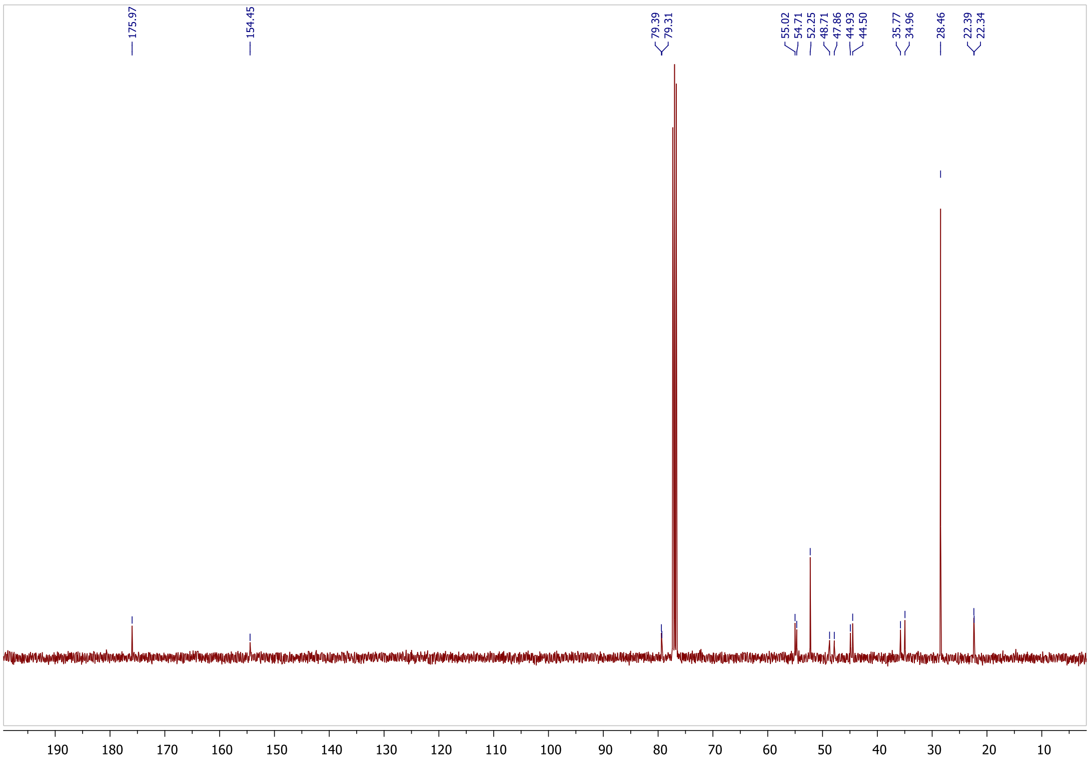

400 MHz ^{1}H NMR spectrum; 100.6 MHz ^{13}C NMR spectrum; $CDCl_3$

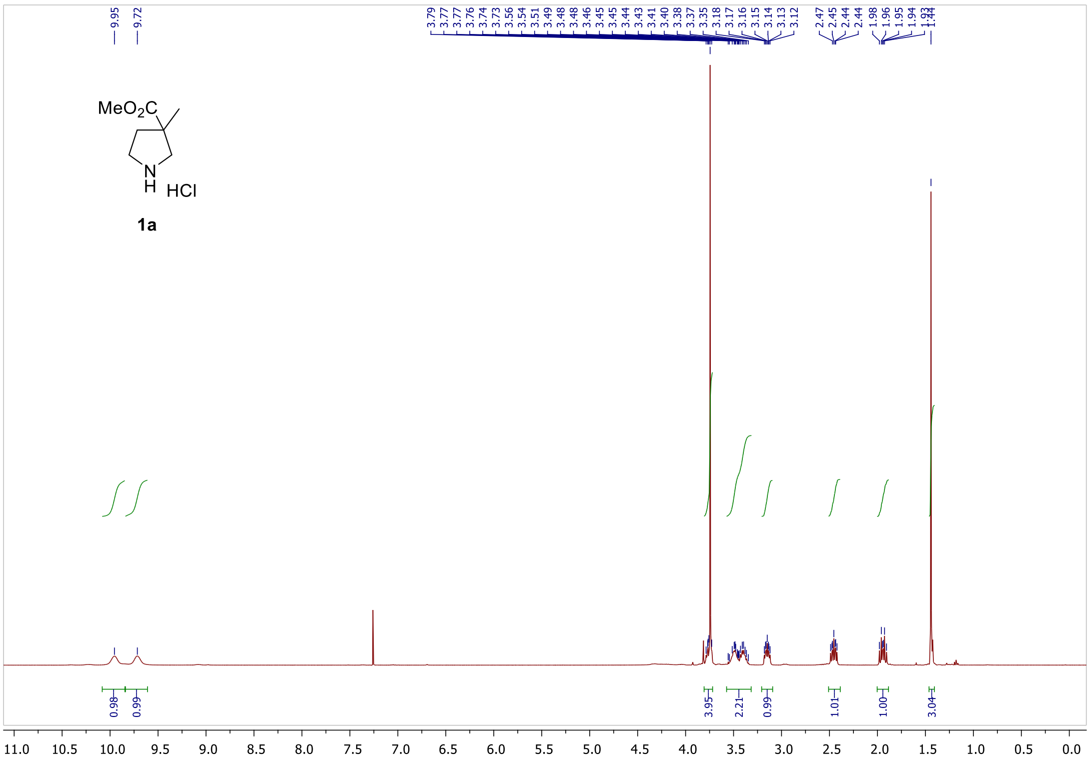

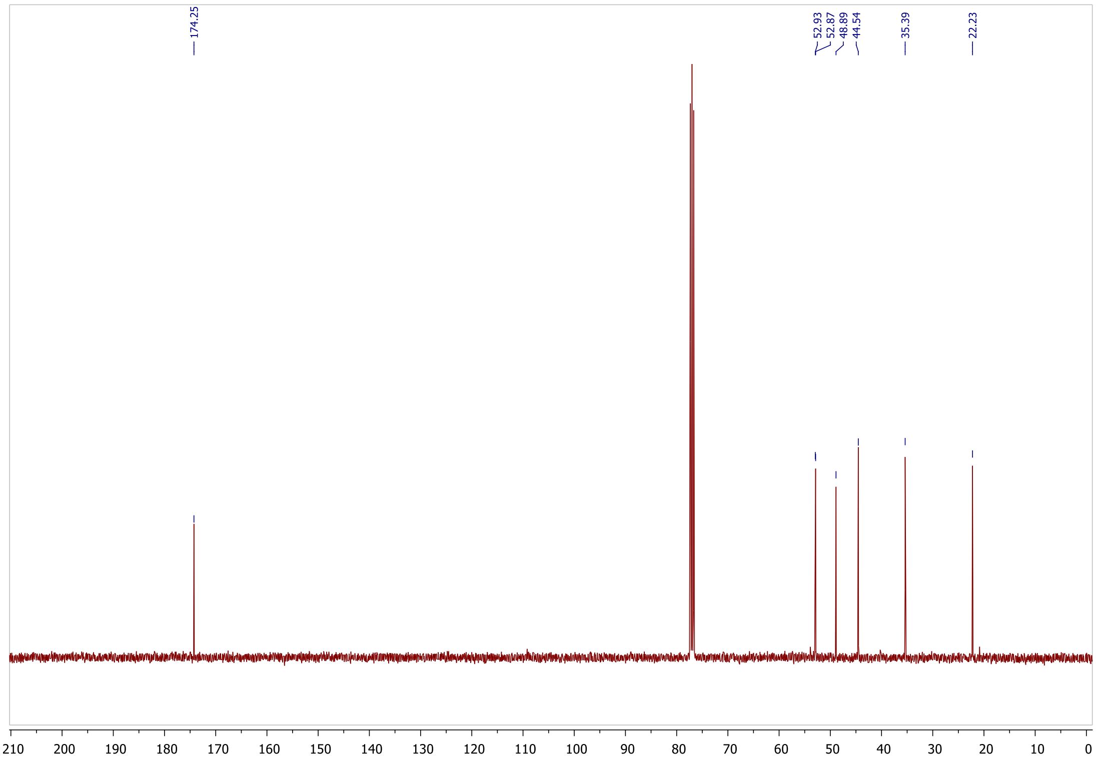

400 MHz ^{1}H NMR spectrum; 100.6 MHz ^{13}C NMR spectrum; $CDCl_3$

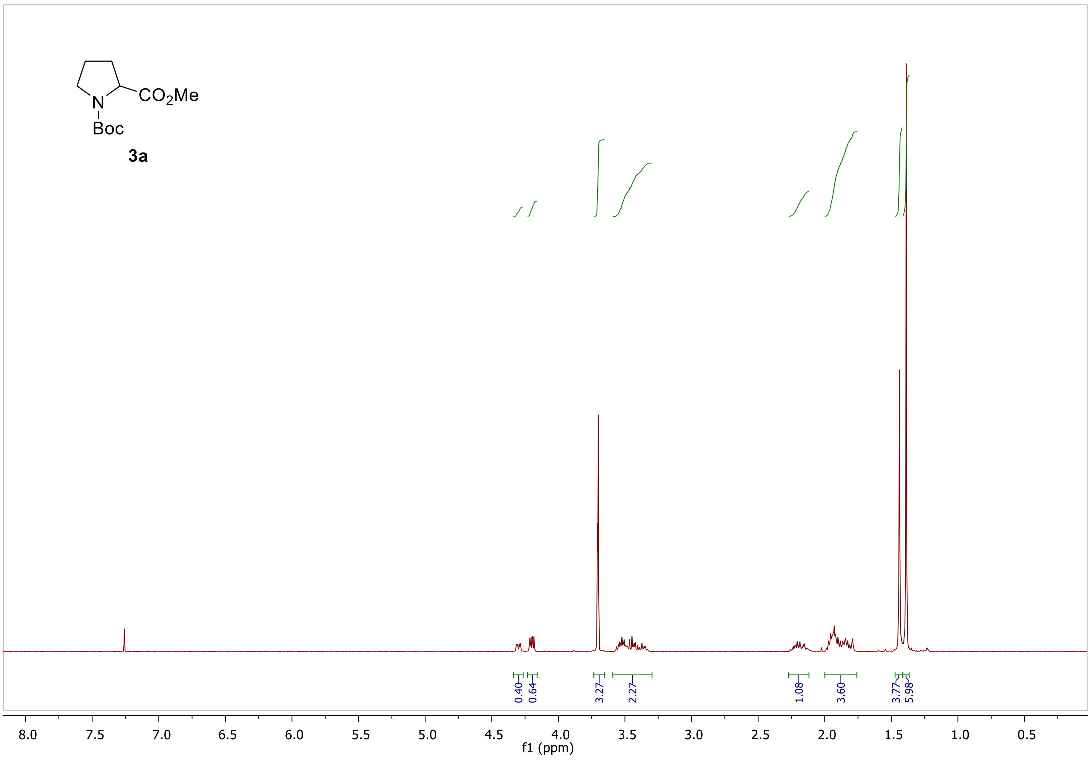

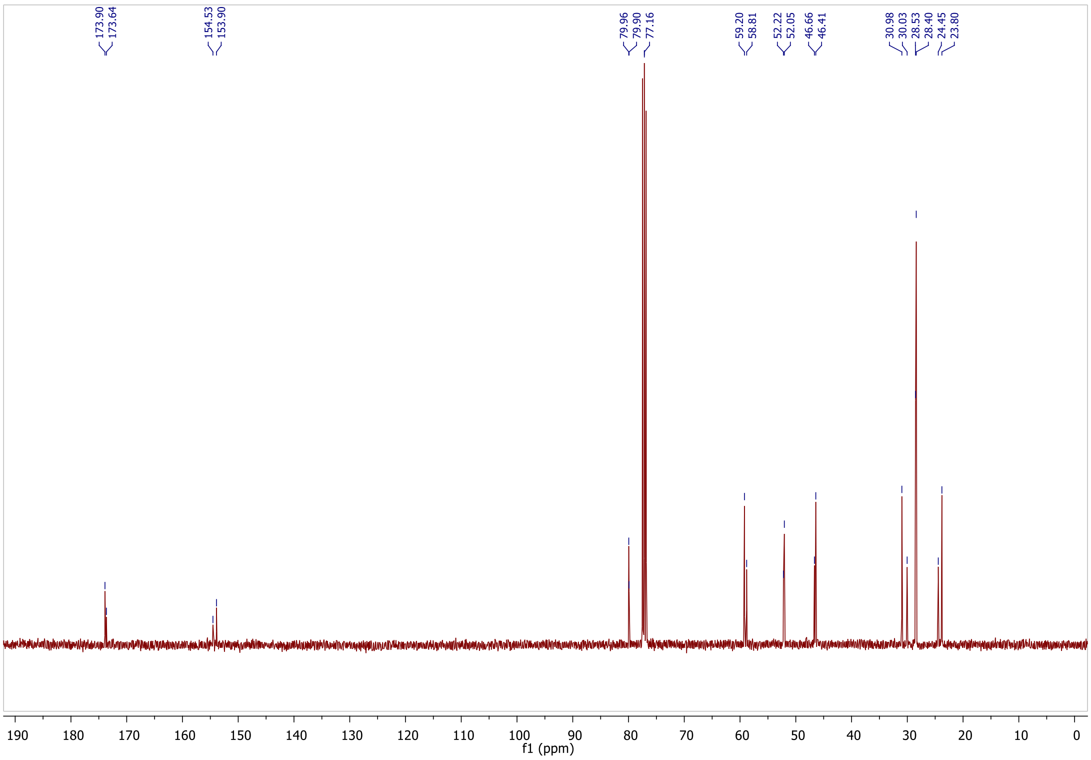

400 MHz ^{1}H NMR spectrum; 100.6 MHz ^{13}C NMR spectrum; $CDCl_3$

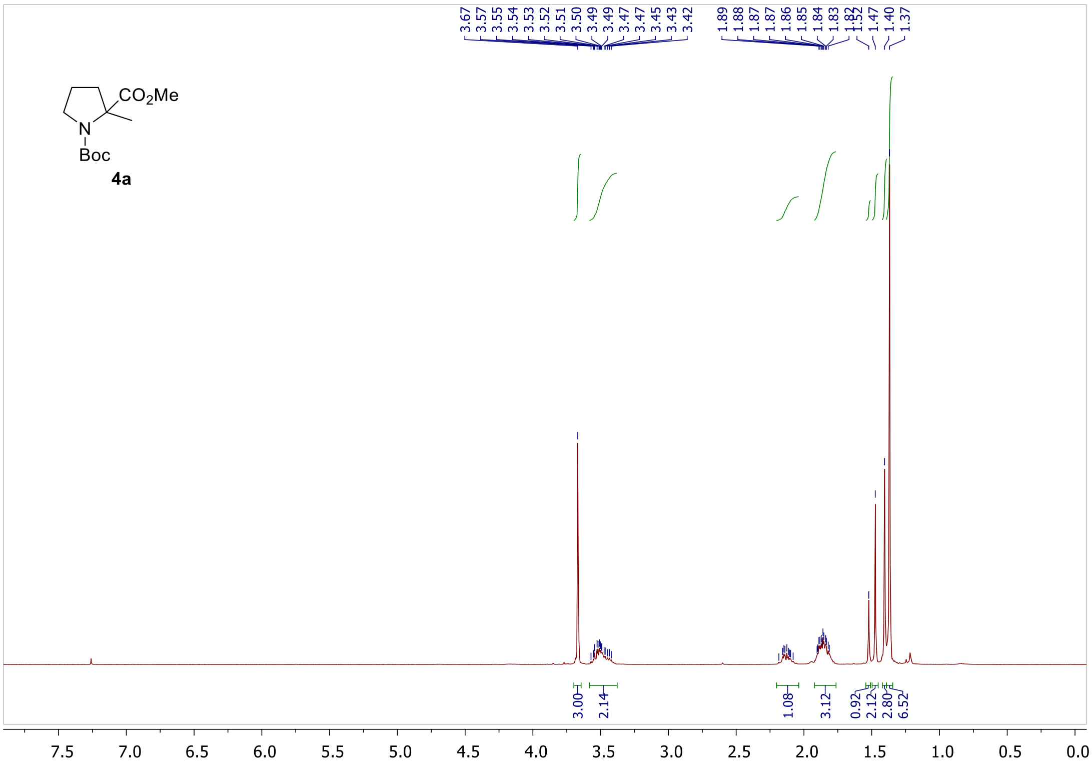

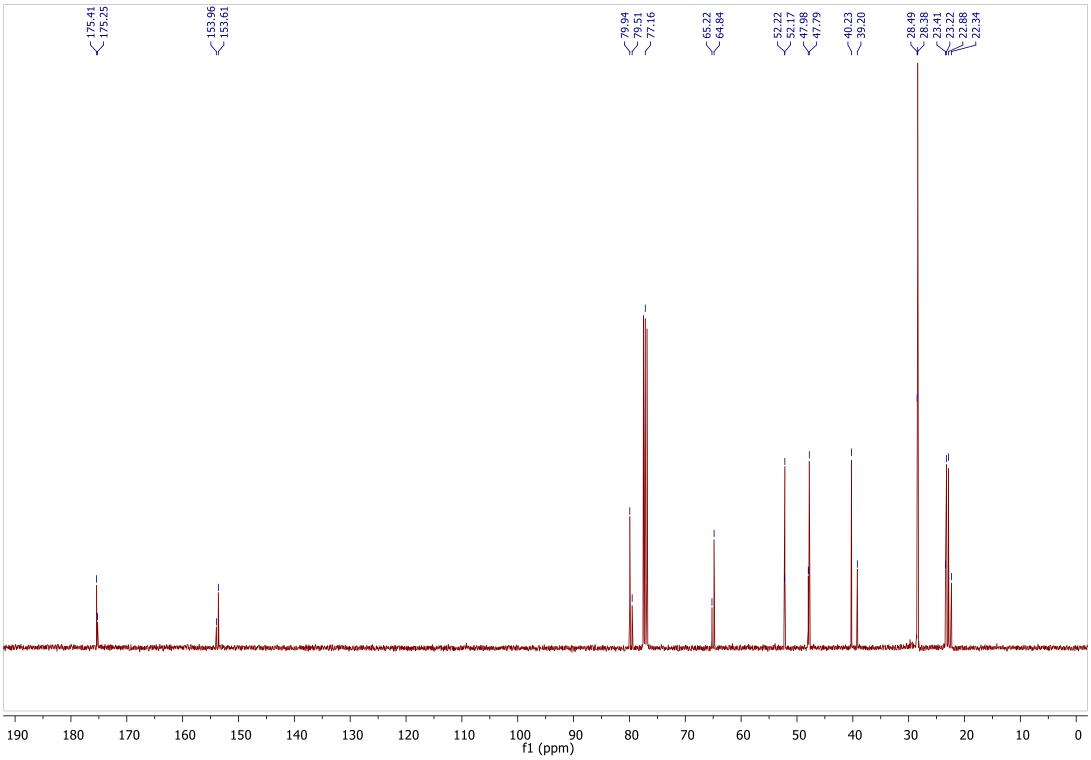

400 MHz ^{1}H NMR spectrum; 100.6 MHz ^{13}C NMR spectrum; $CDCl_3$

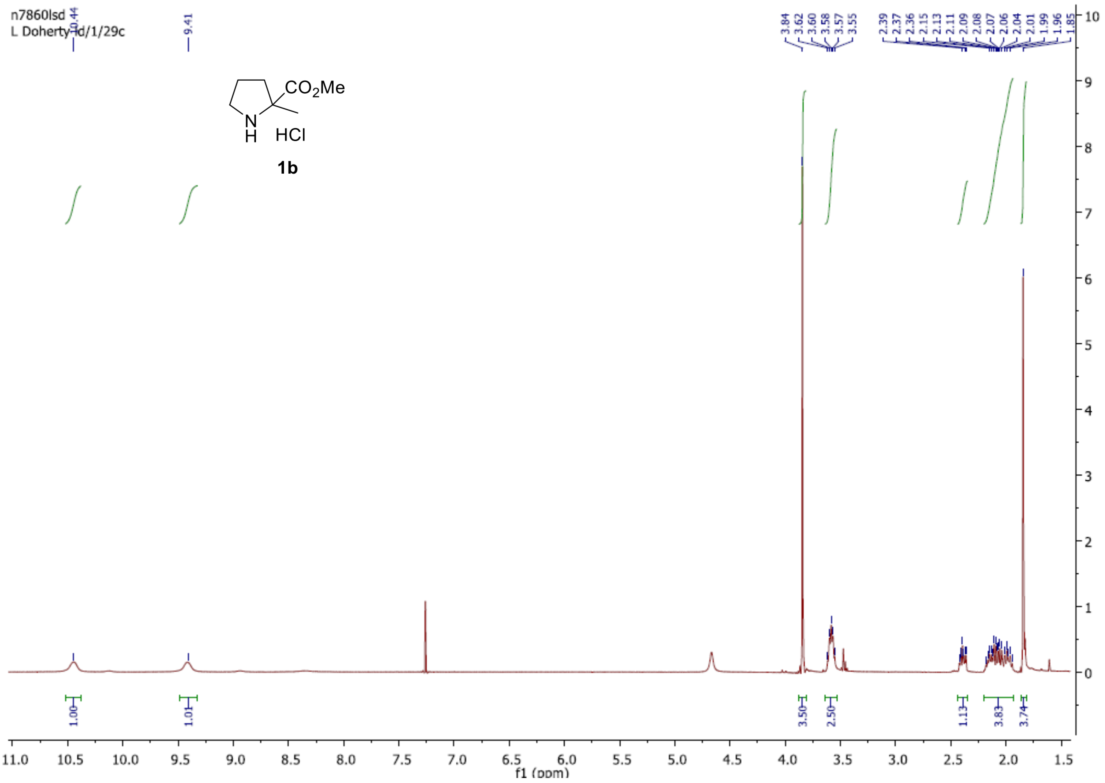

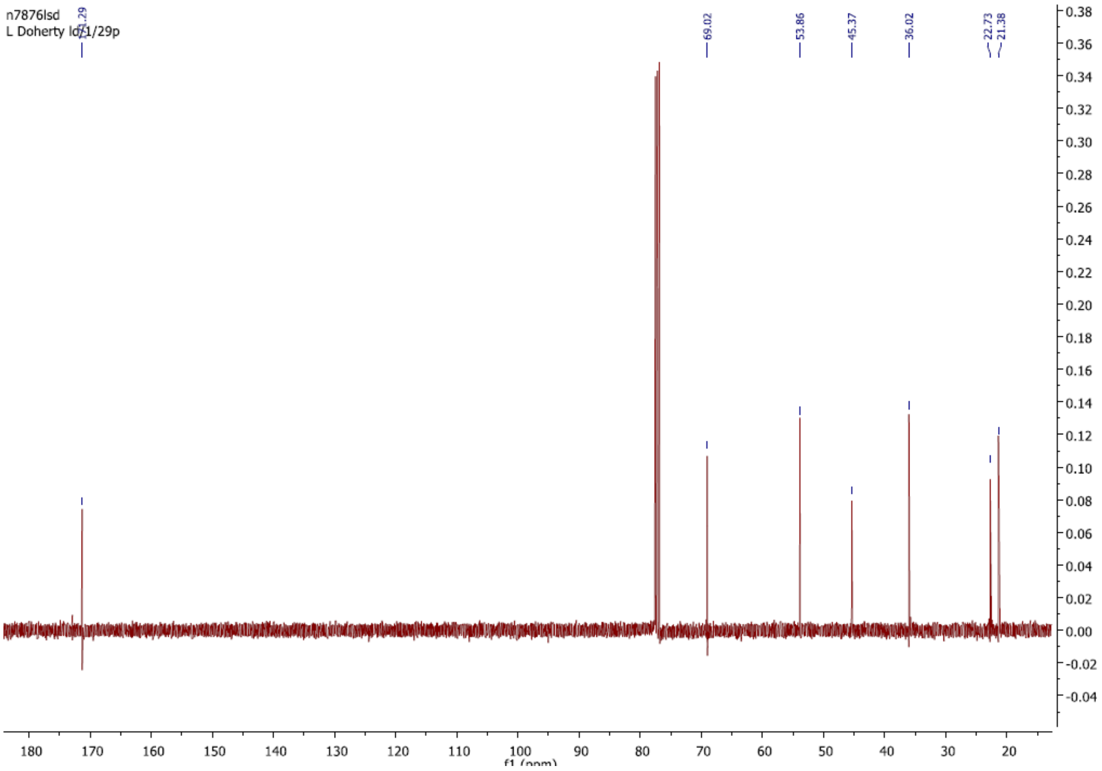

400 MHz ^{1}H NMR spectrum; 100.6 MHz ^{13}C NMR spectrum; MeOH-*d*4

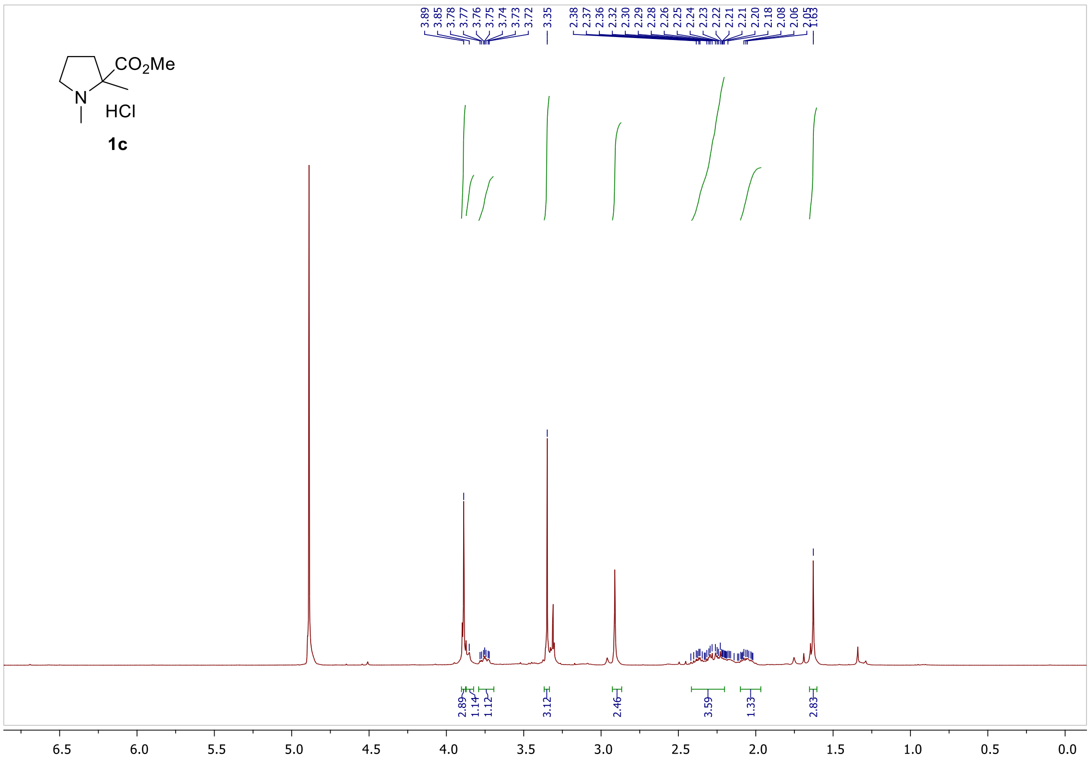

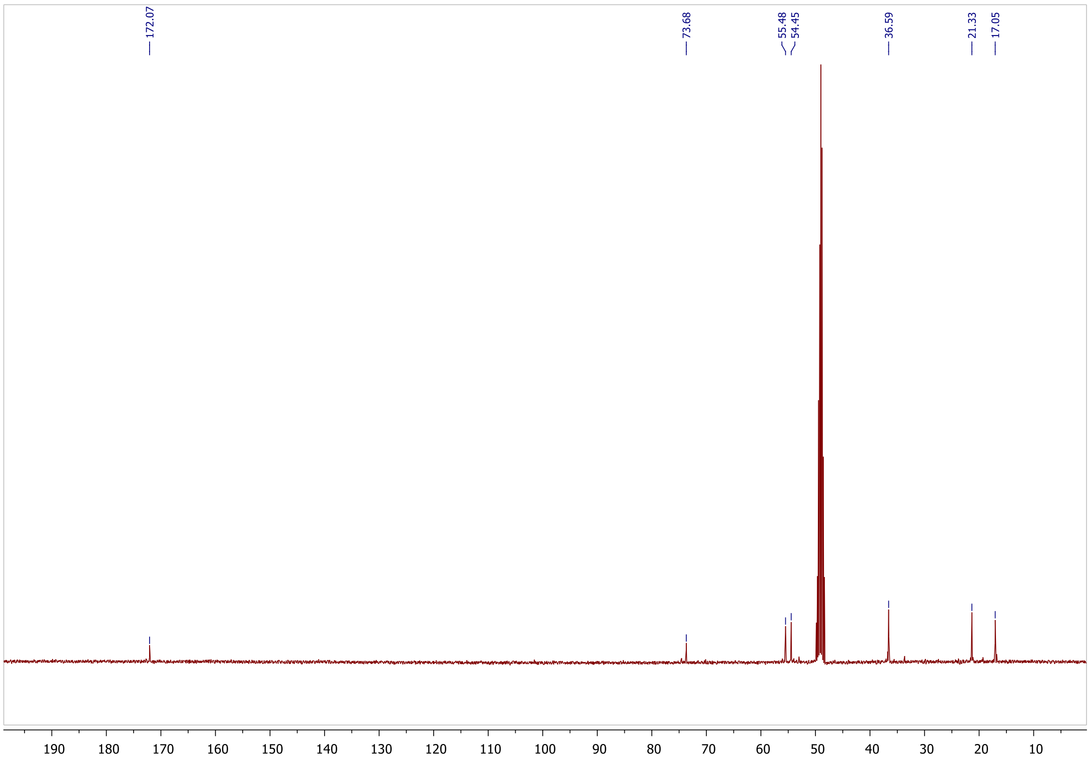

400 MHz ^{1}H NMR spectrum; 100.6 MHz ^{13}C NMR spectrum; $CDCl_3$

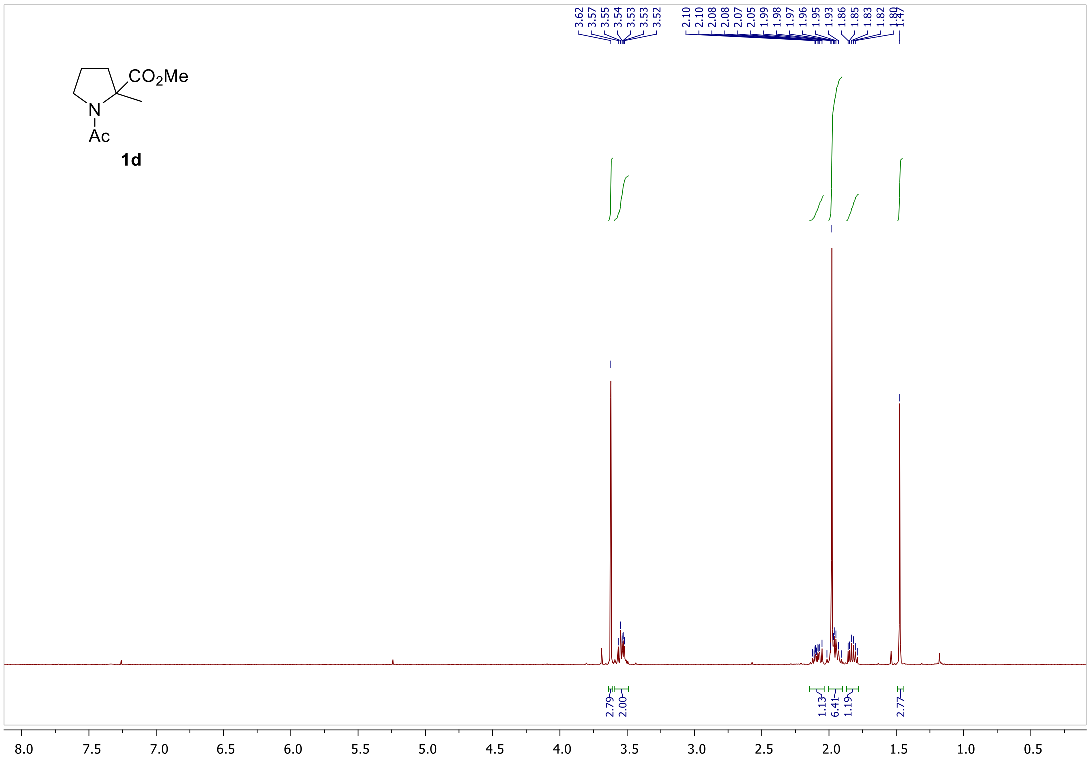

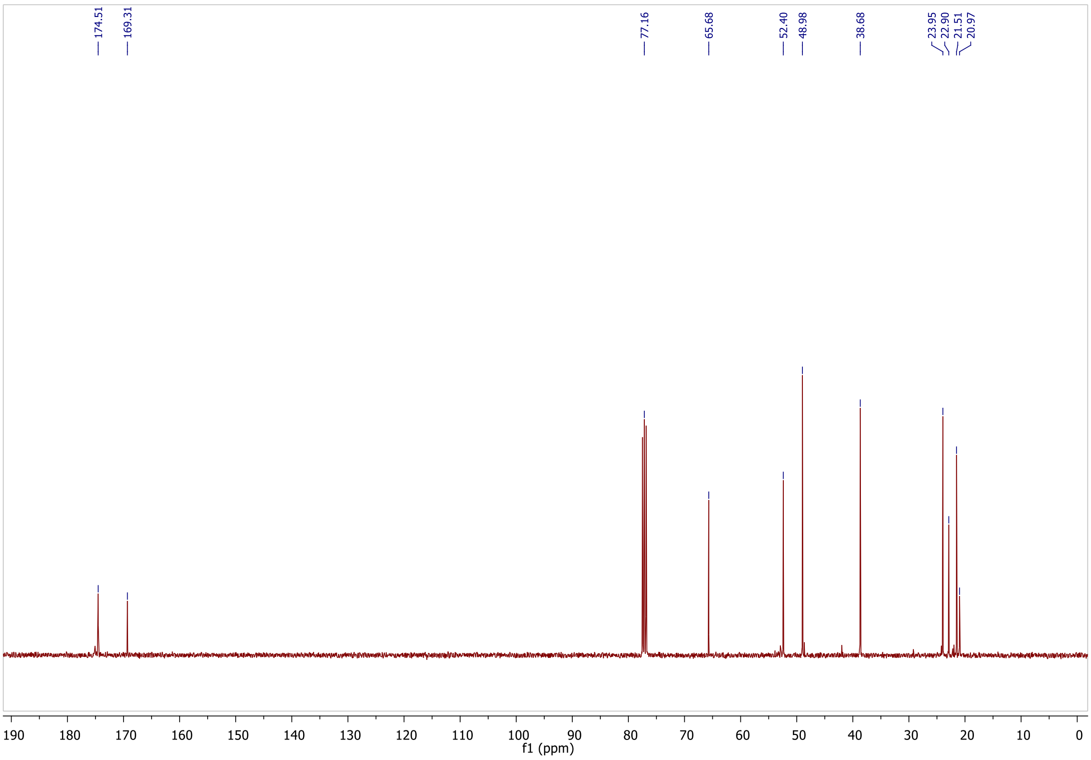

400 MHz ^{1}H NMR spectrum; 100.6 MHz ^{13}C NMR spectrum; $CDCl_3$

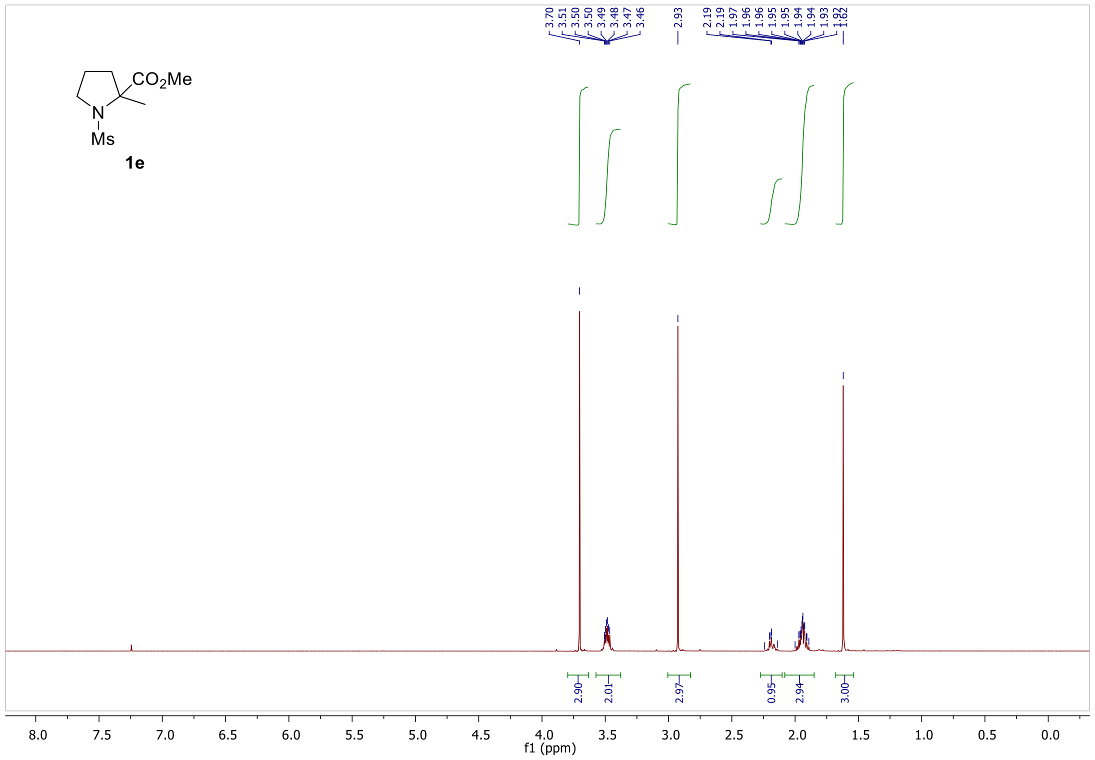

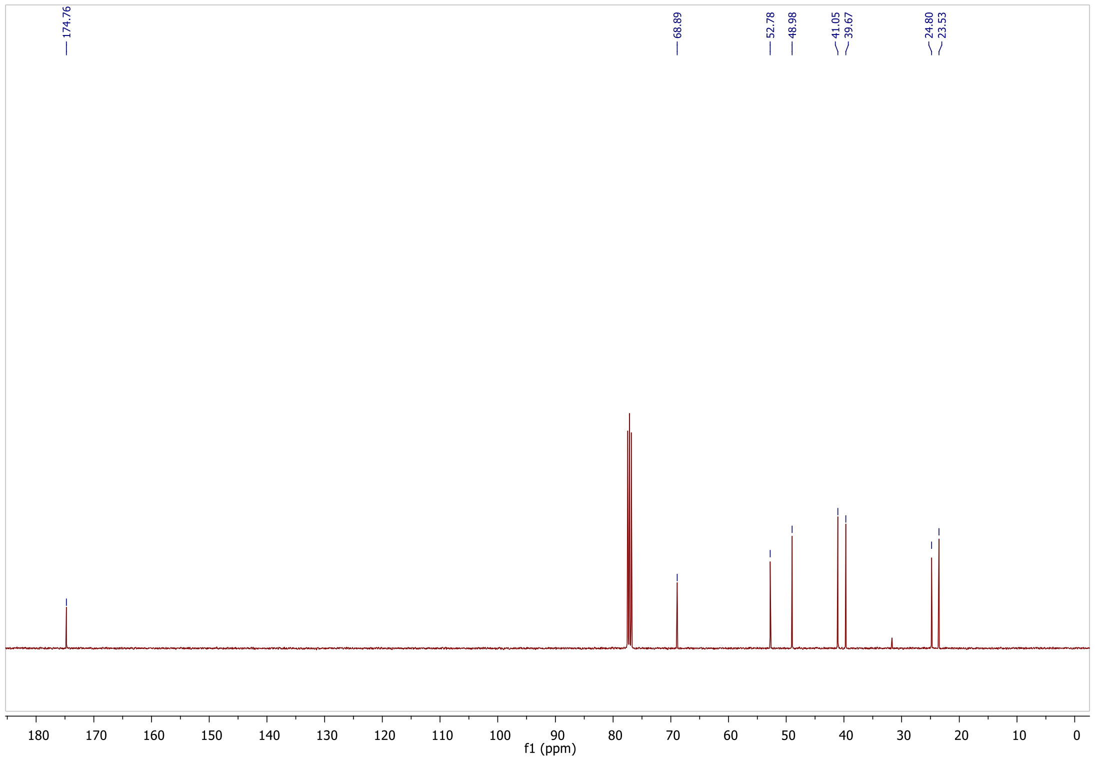

400 MHz ^{1}H NMR spectrum; 100.6 MHz ^{13}C NMR spectrum; $CDCl_3$

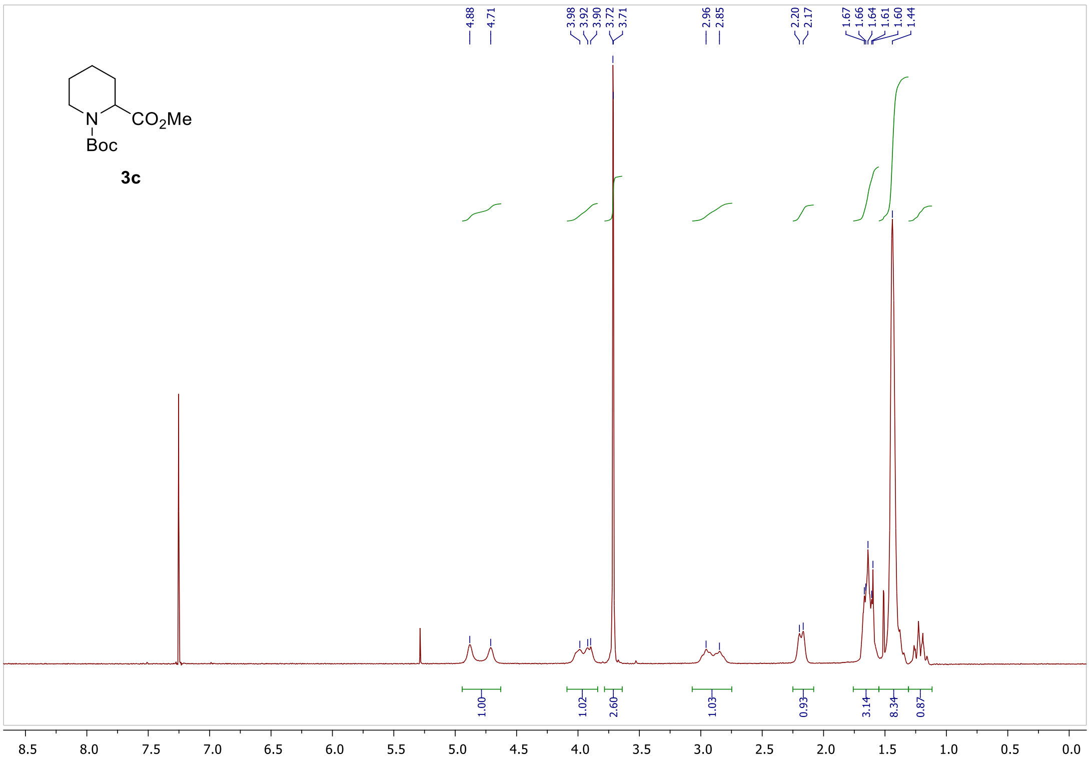

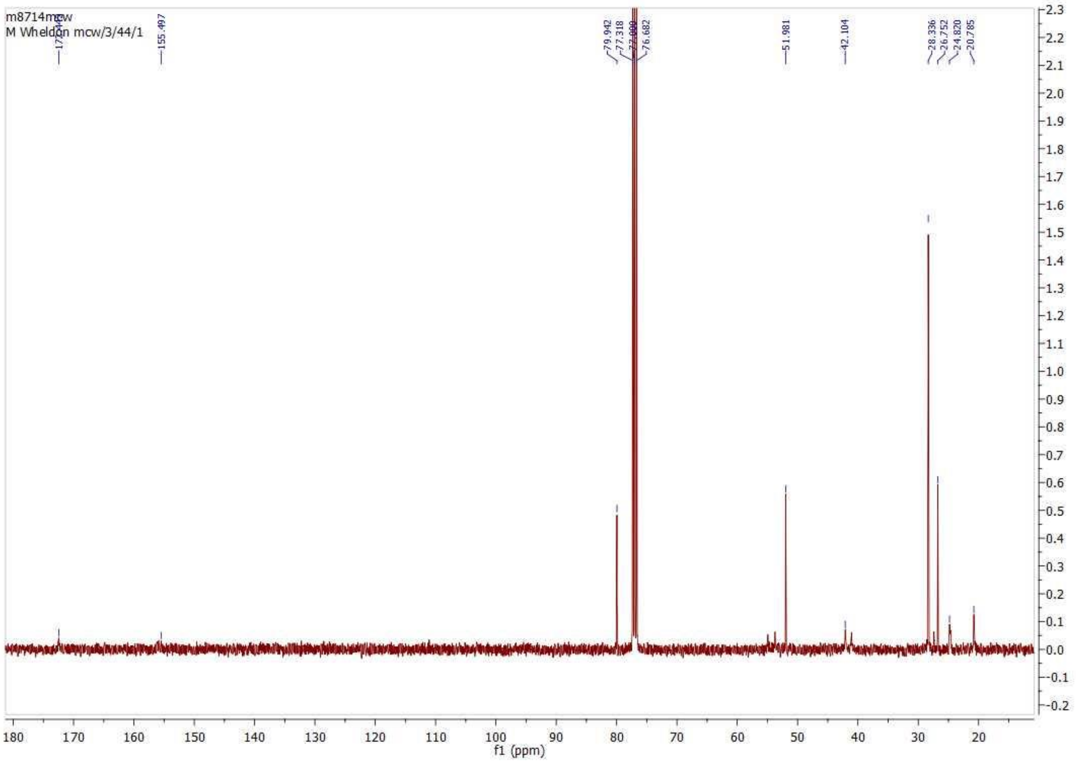

400 MHz ^{1}H NMR spectrum; 100.6 MHz ^{13}C NMR spectrum; $CDCl_3$

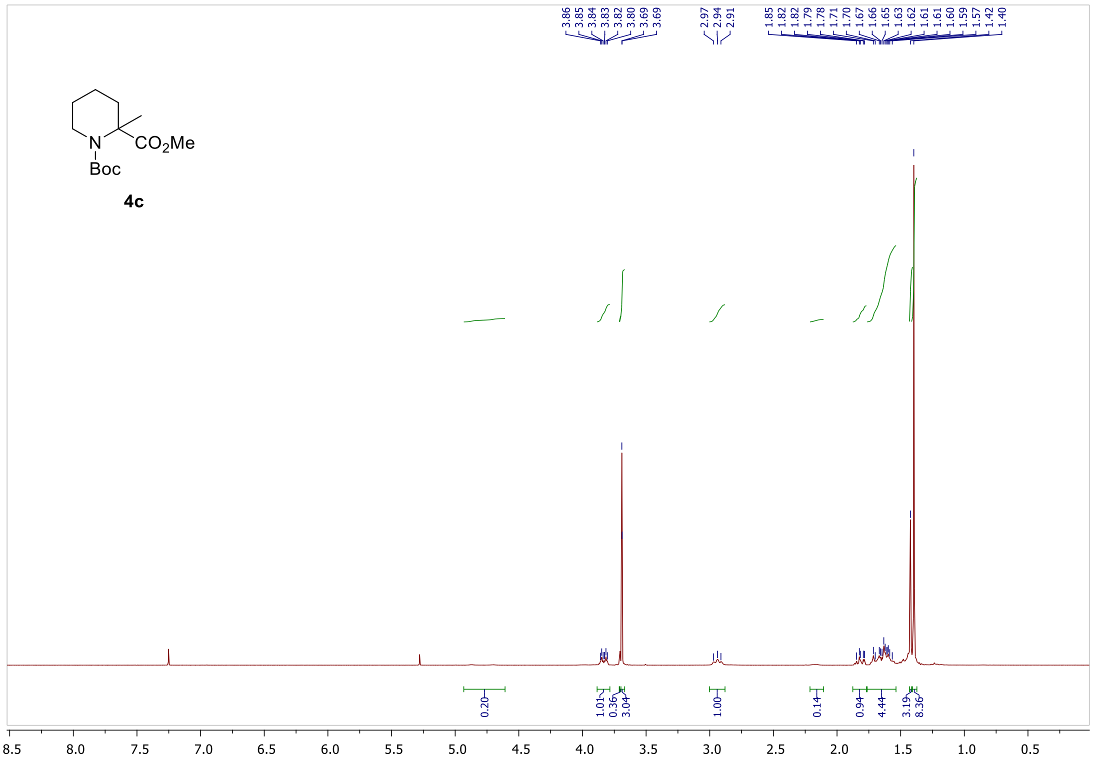

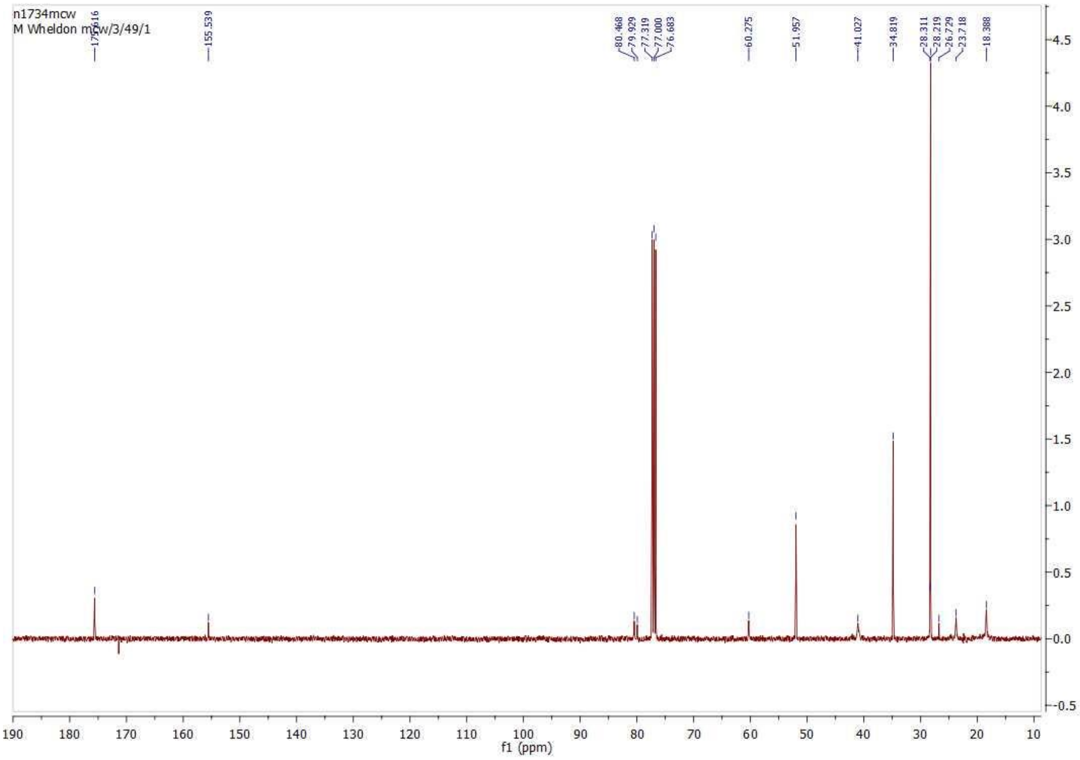

400 MHz ^{1}H NMR spectrum; 100.6 MHz ^{13}C NMR spectrum; $CDCl_3$

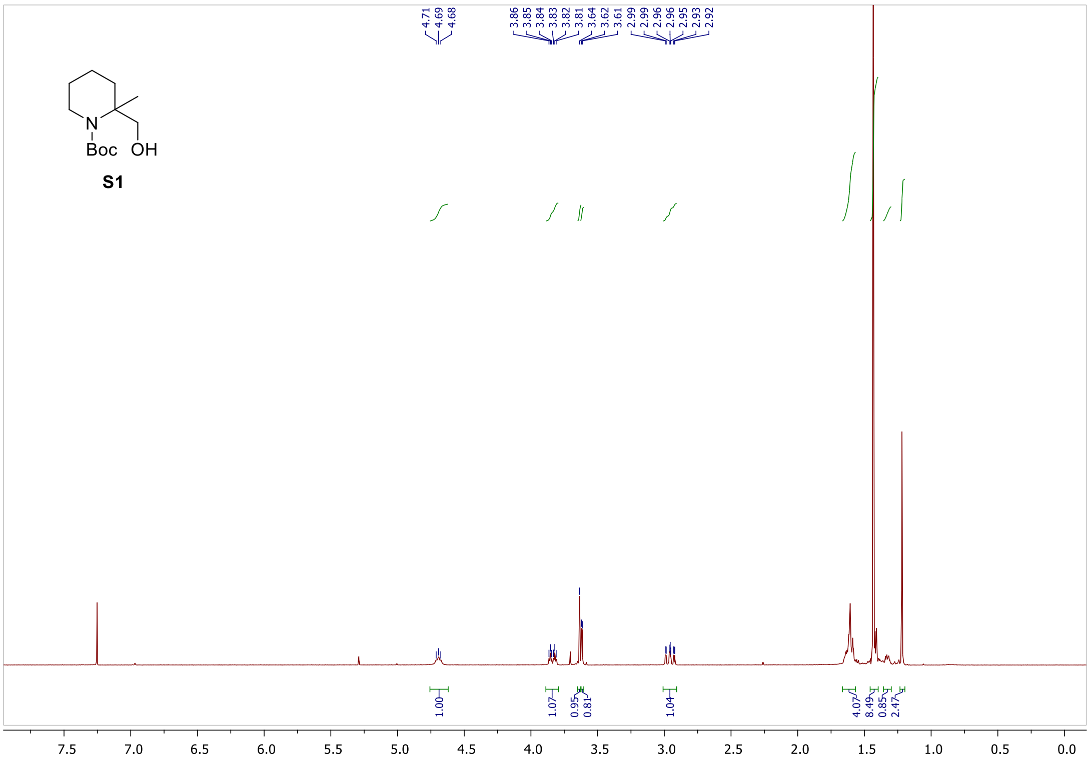

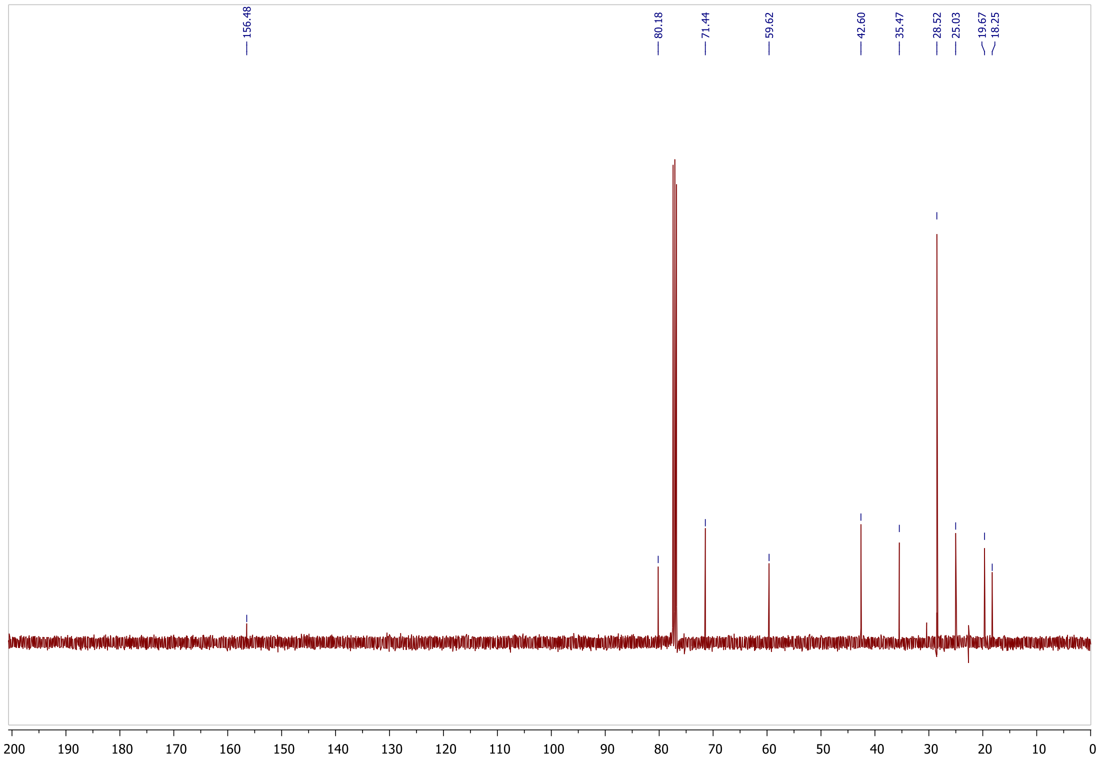

400 MHz ^{1}H NMR spectrum; 100.6 MHz ^{13}C NMR spectrum; DMSO-d_6

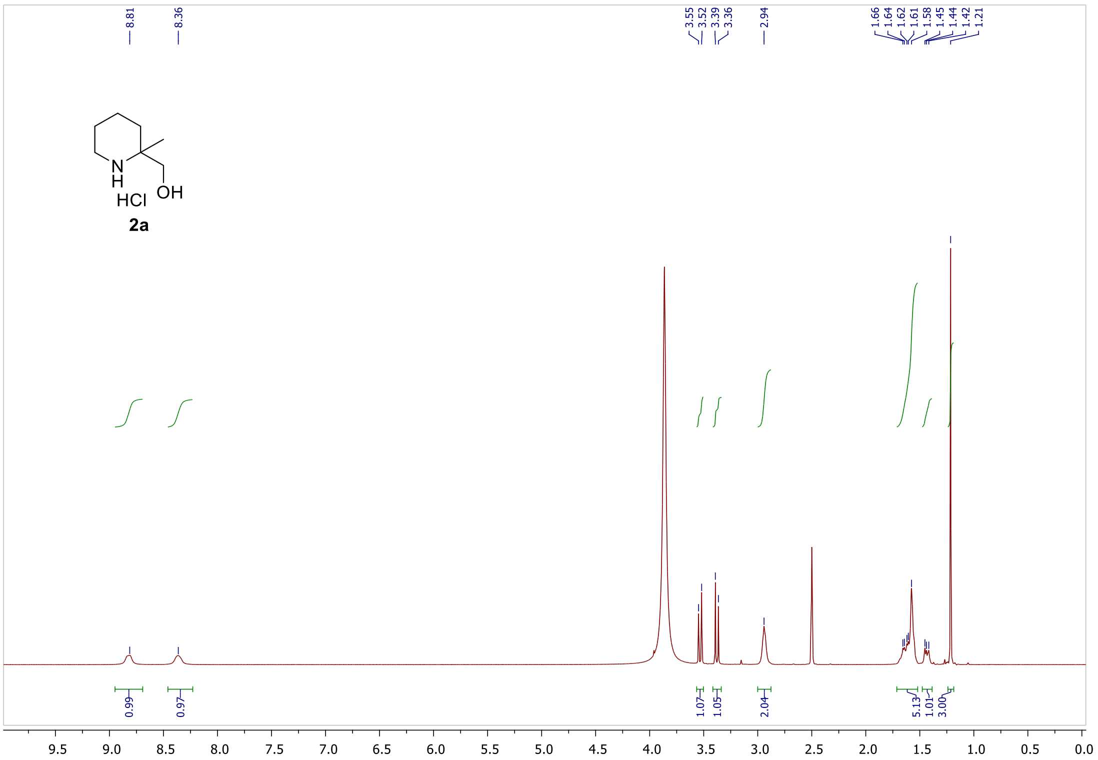

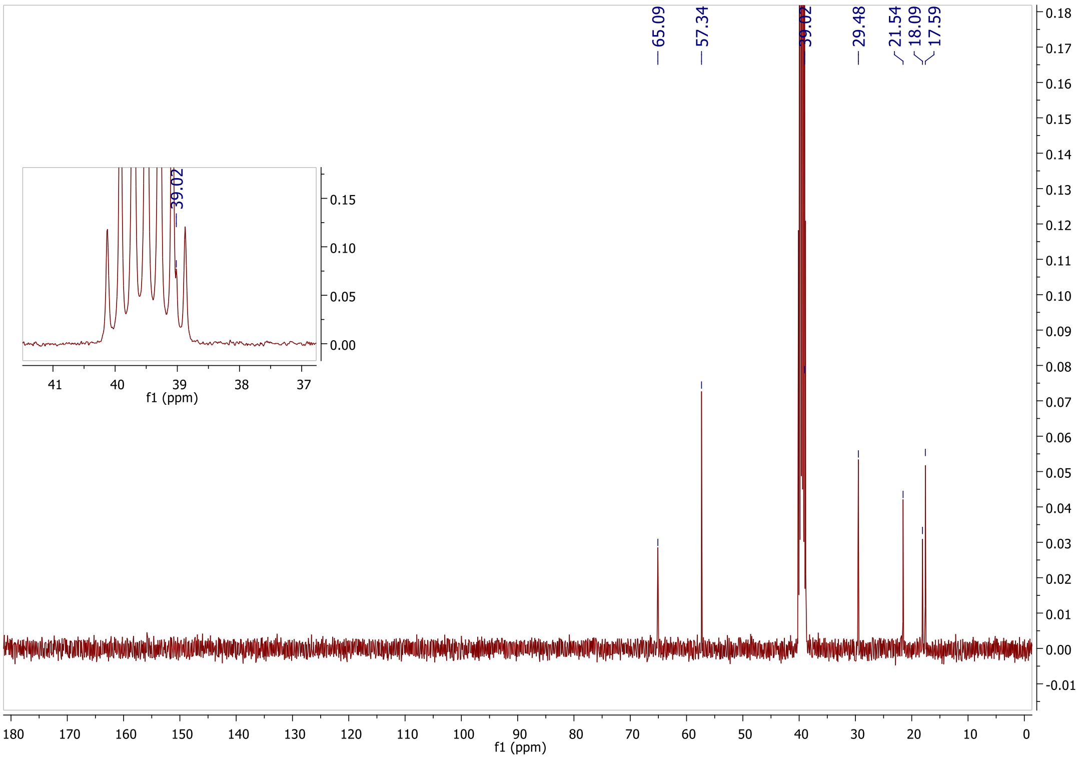

400 MHz ^{1}H NMR spectrum; 100.6 MHz ^{13}C NMR spectrum; $CDCl_3$

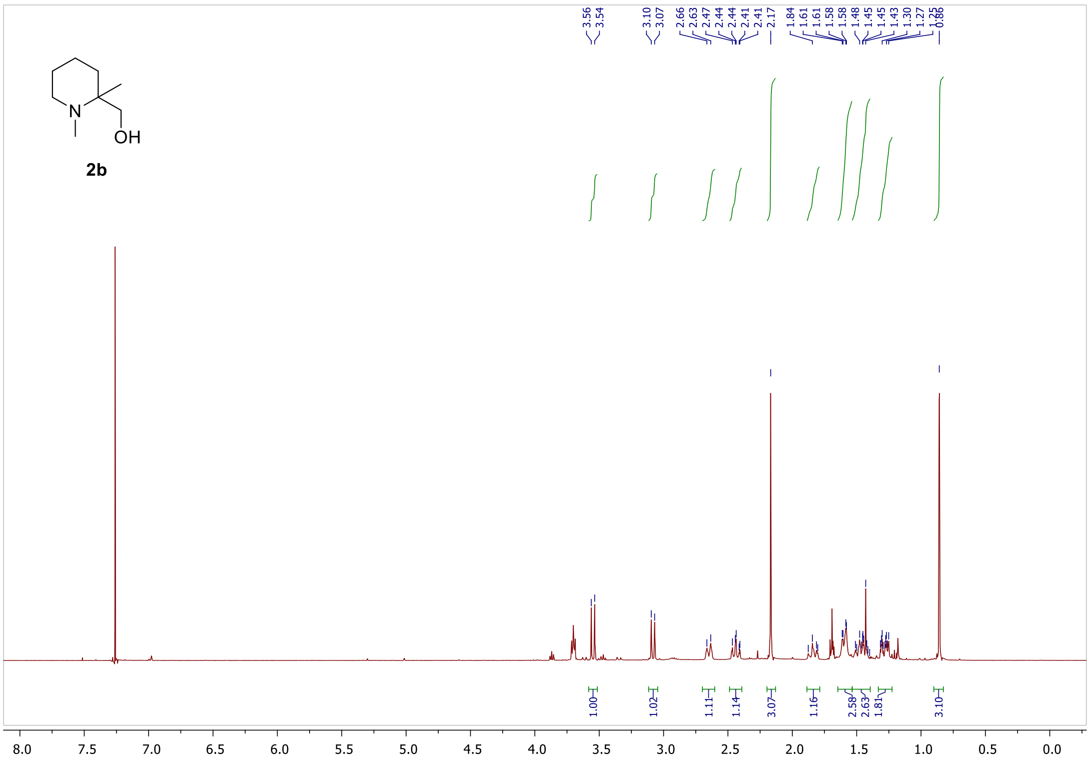

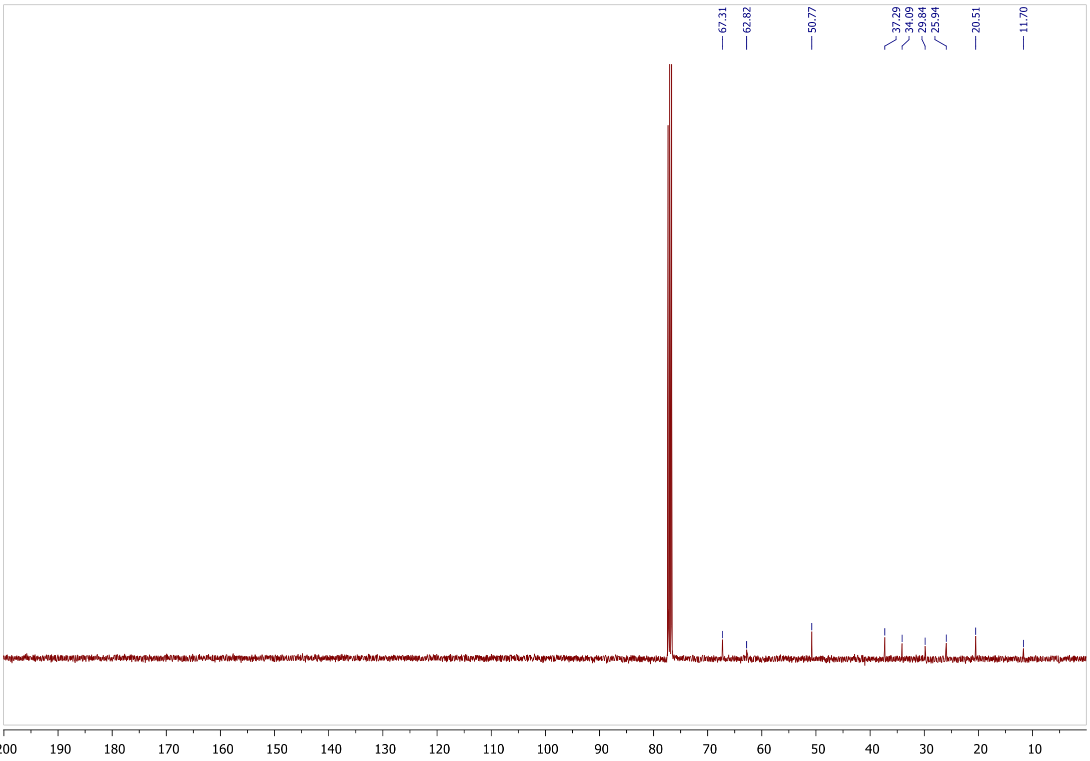

400 MHz ^{1}H NMR spectrum; 100.6 MHz ^{13}C NMR spectrum; $CDCl_3$

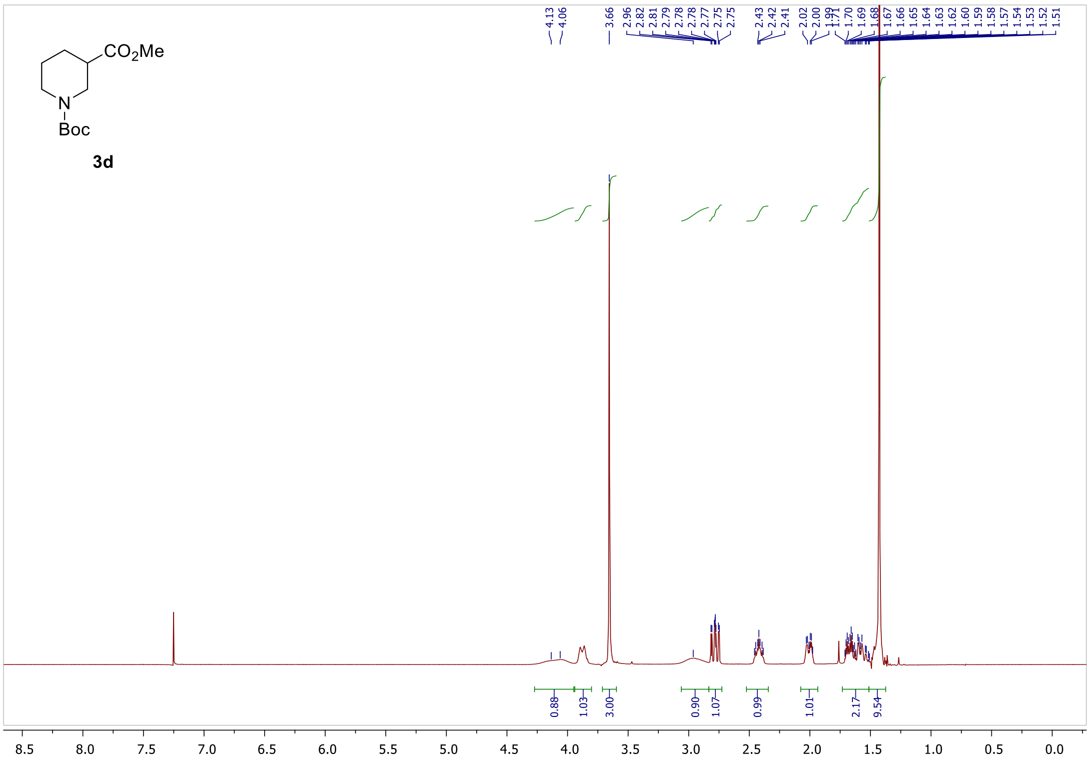

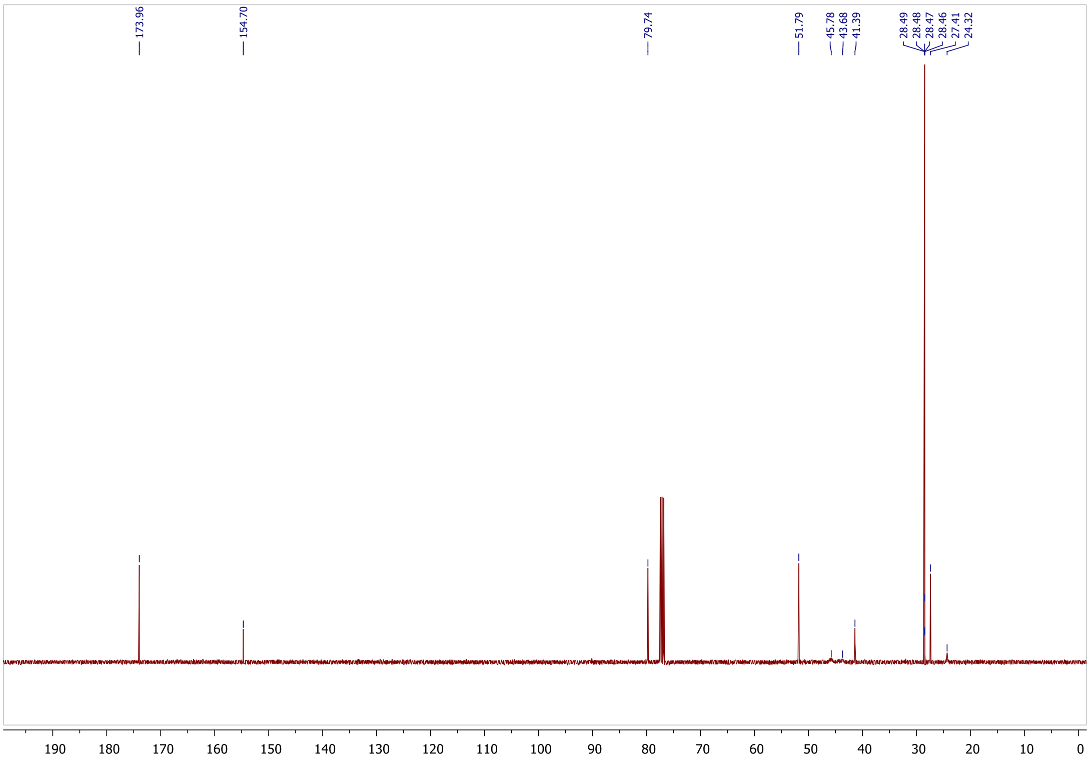

400 MHz ^{1}H NMR spectrum; 100.6 MHz ^{13}C NMR spectrum; $CDCl_3$

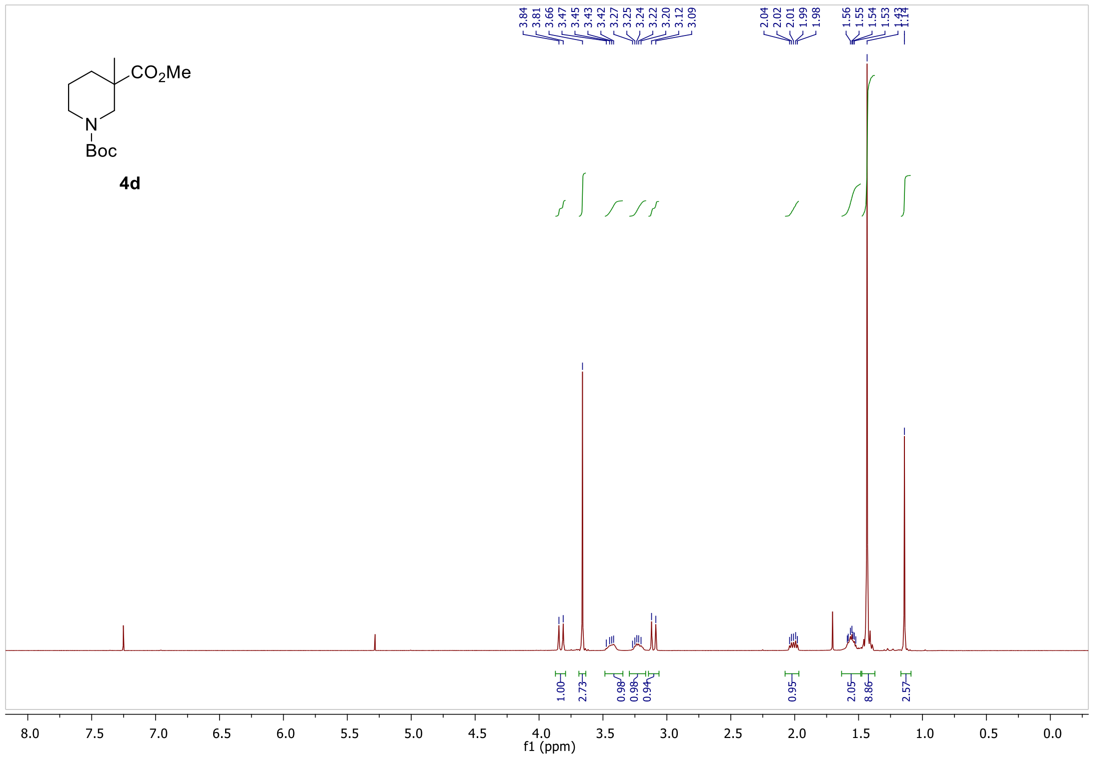

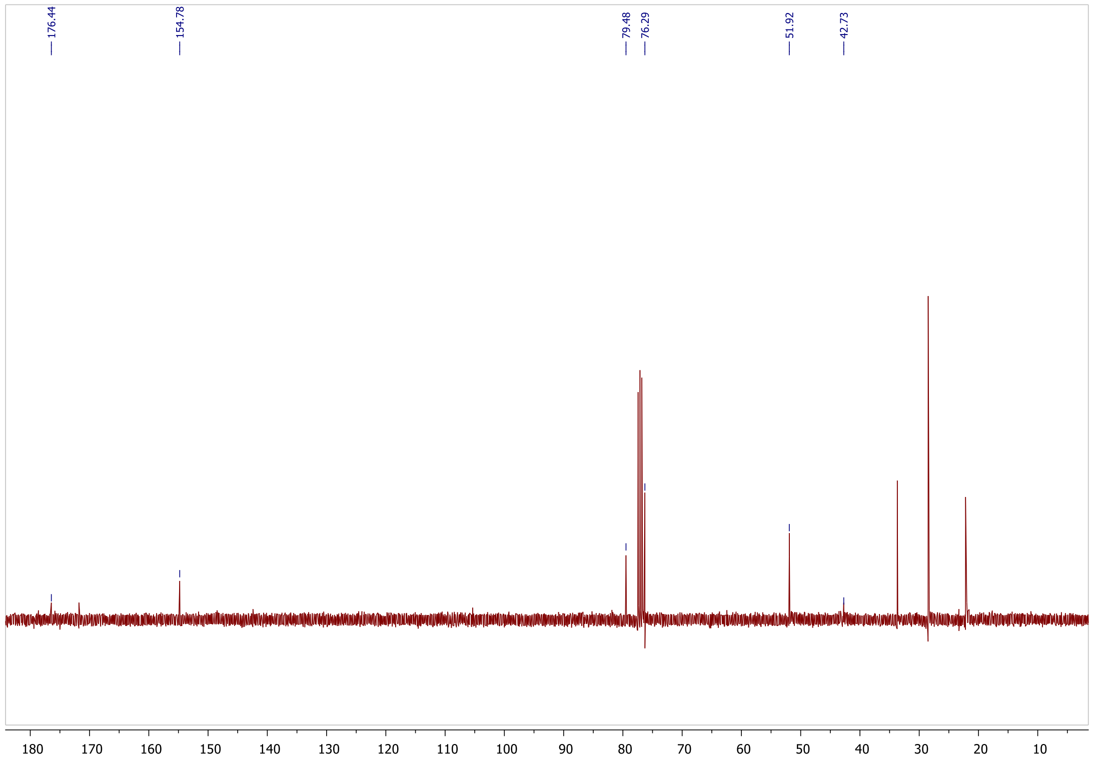

400 MHz ^{1}H NMR spectrum; 100.6 MHz ^{13}C NMR spectrum; $CDCl_3$

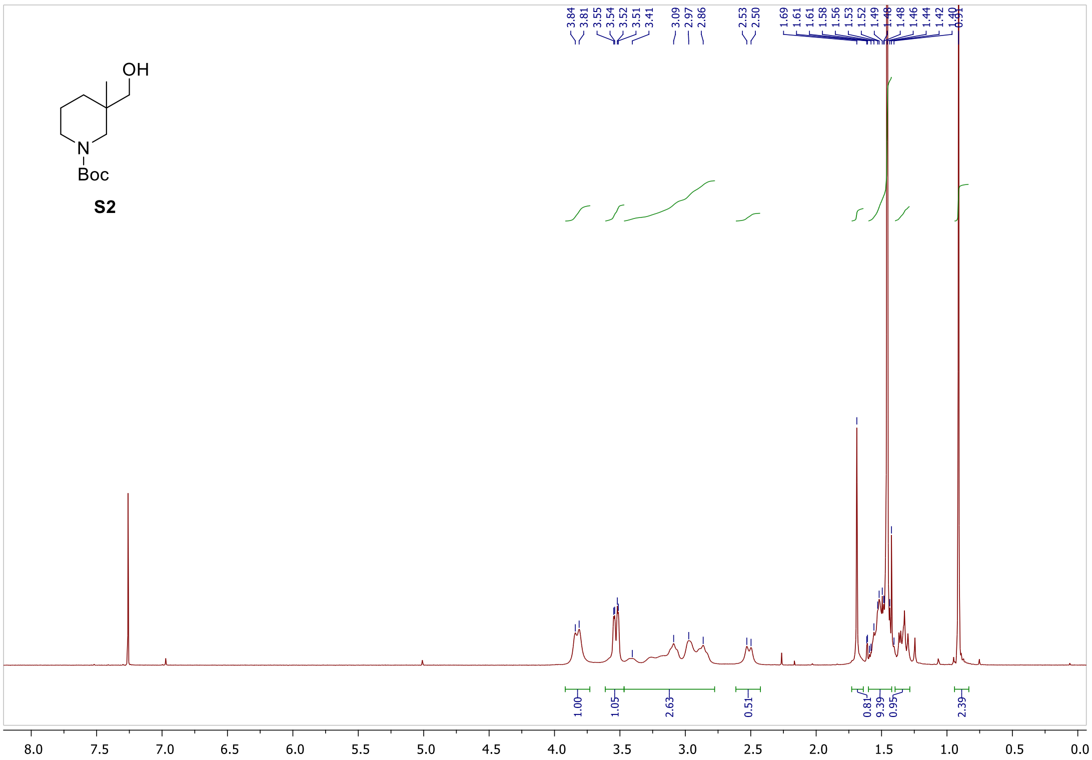

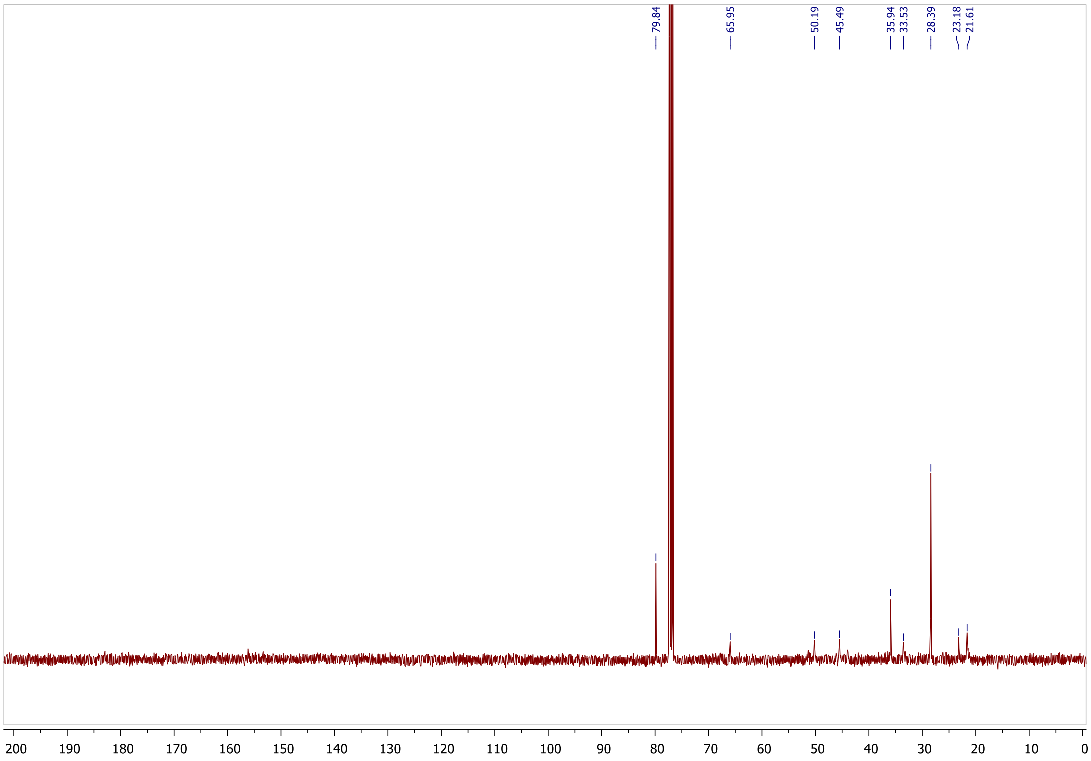

400 MHz ^{1}H NMR spectrum; 100.6 MHz ^{13}C NMR spectrum; DMSO-d_6

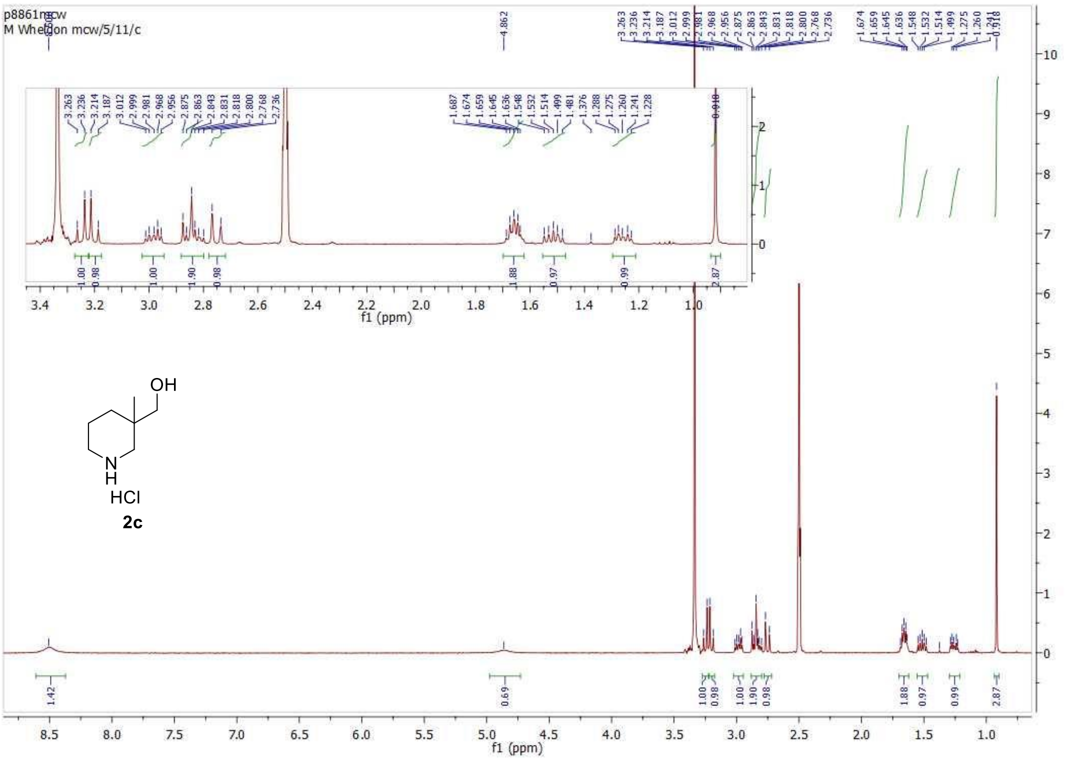

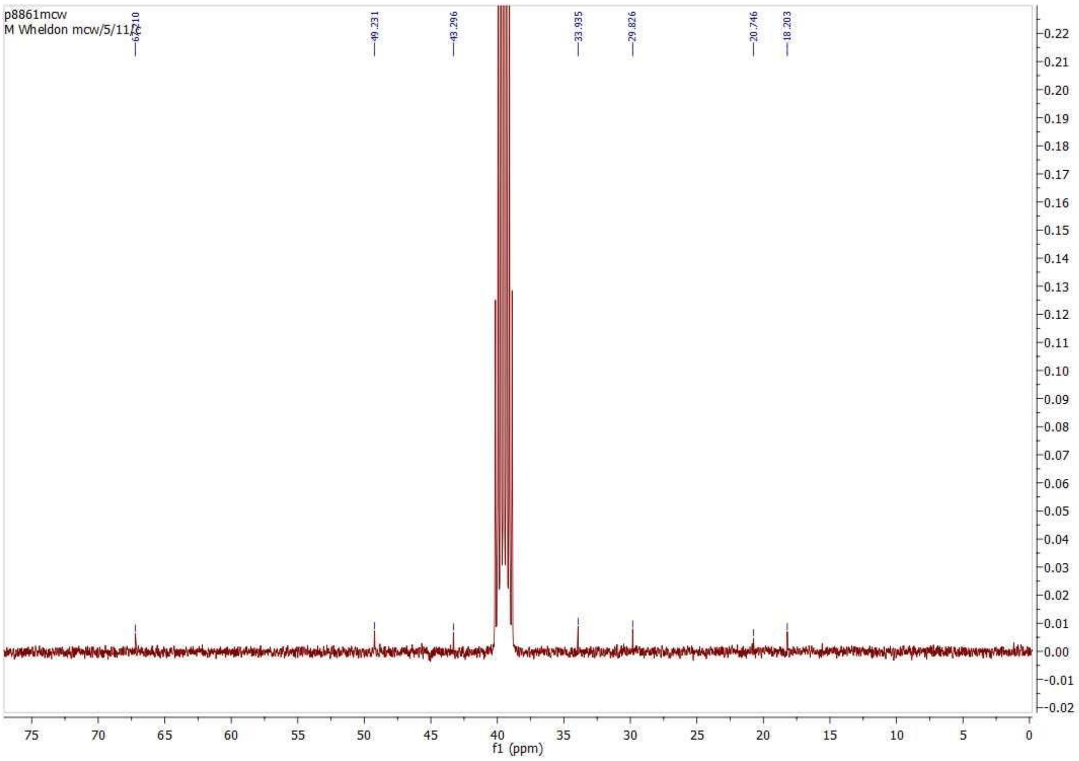

400 MHz ^{1}H NMR spectrum; 100.6 MHz ^{13}C NMR spectrum; $CDCl_3$

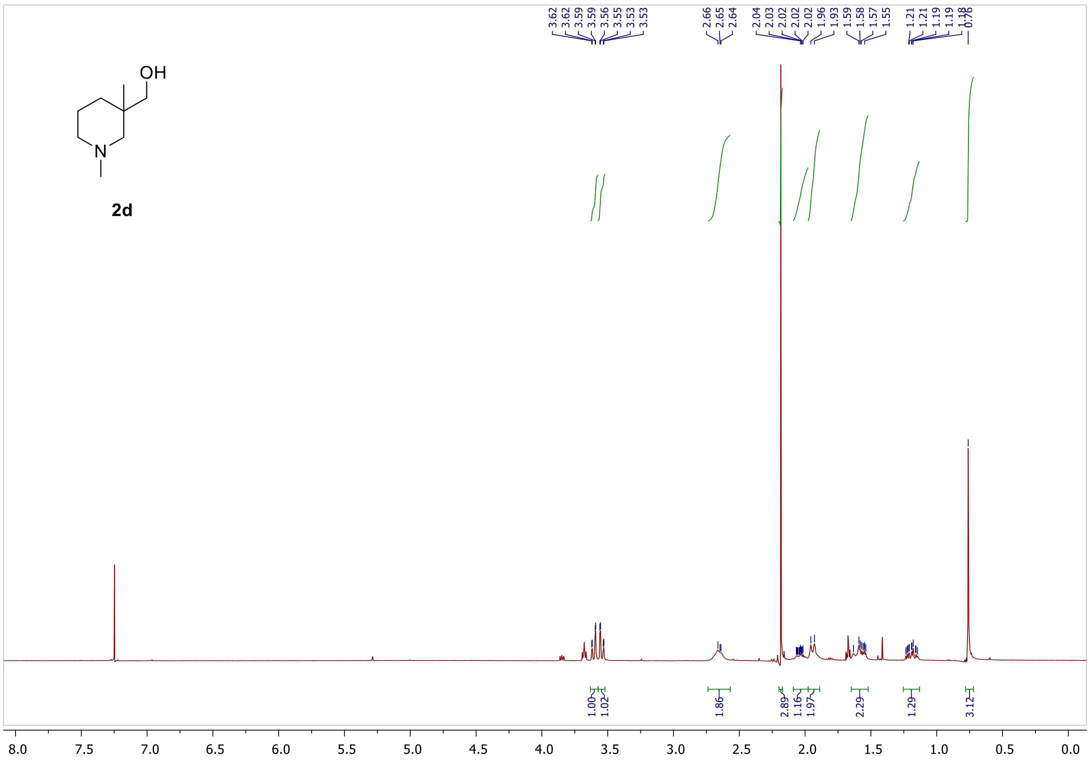

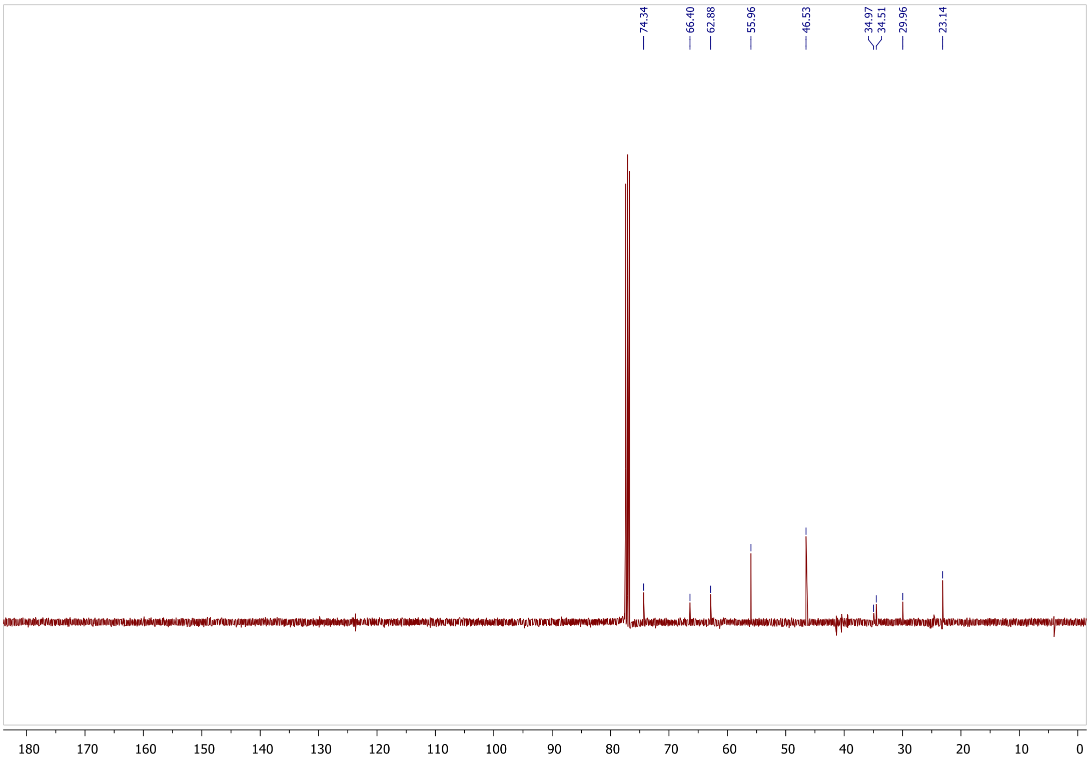

400 MHz ^{1}H NMR spectrum; 100.6 MHz ^{13}C NMR spectrum; $CDCl_3$

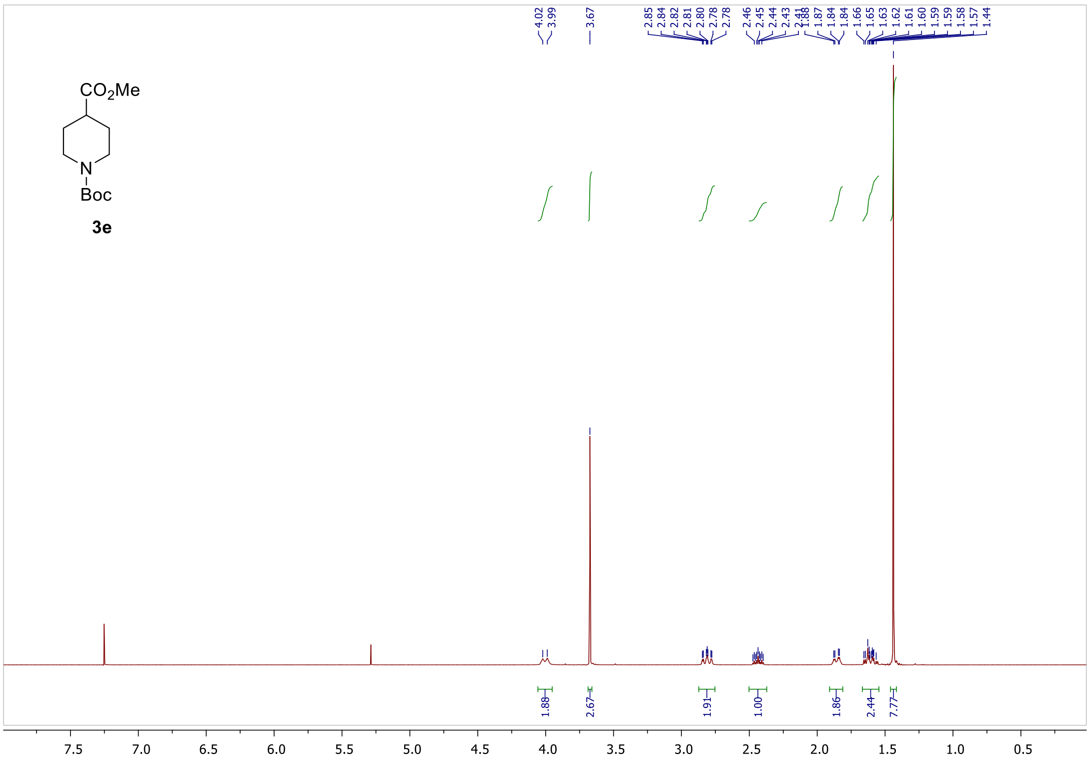

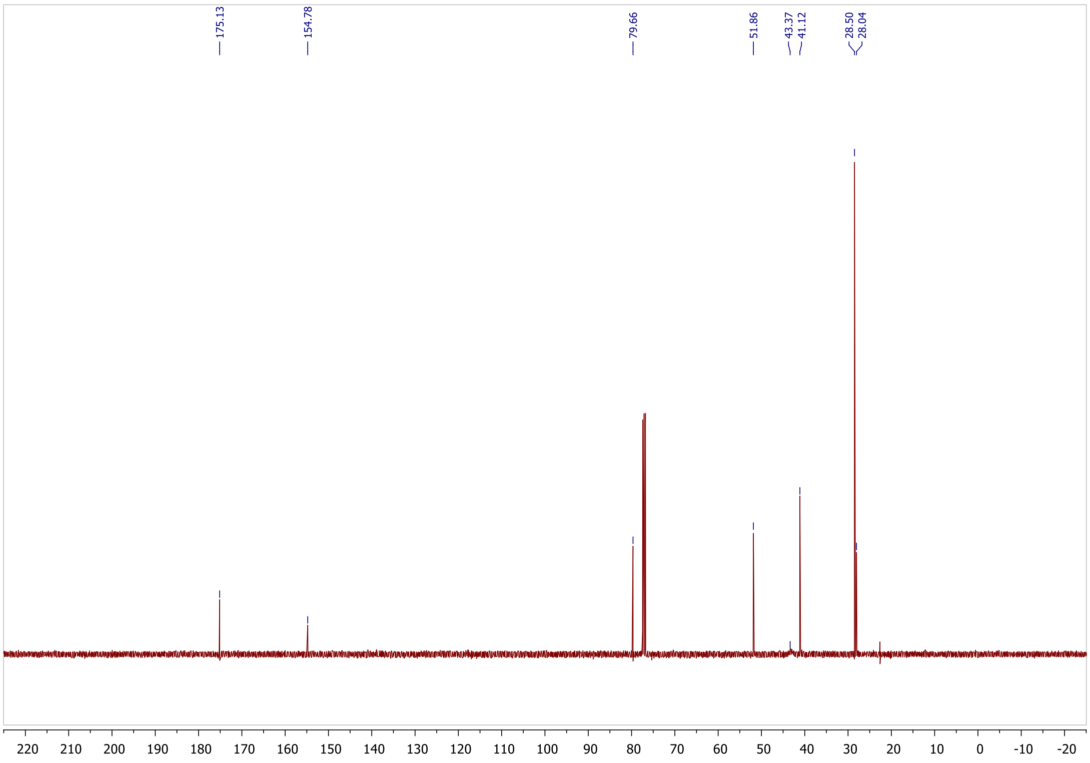

400 MHz ^{1}H NMR spectrum; 100.6 MHz ^{13}C NMR spectrum; $CDCl_3$

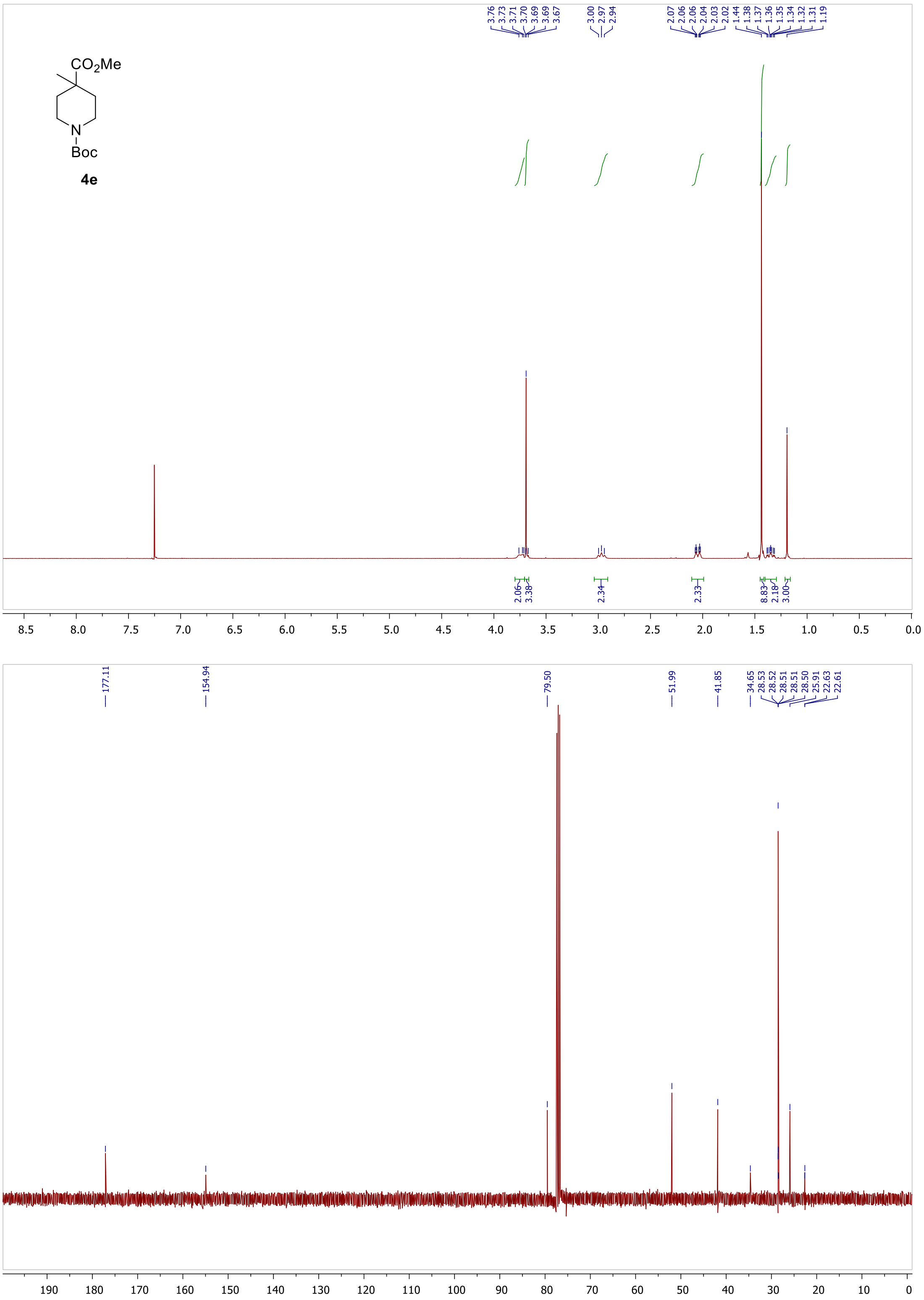

400 MHz ^{1}H NMR spectrum; 100.6 MHz ^{13}C NMR spectrum; $CDCl_3$

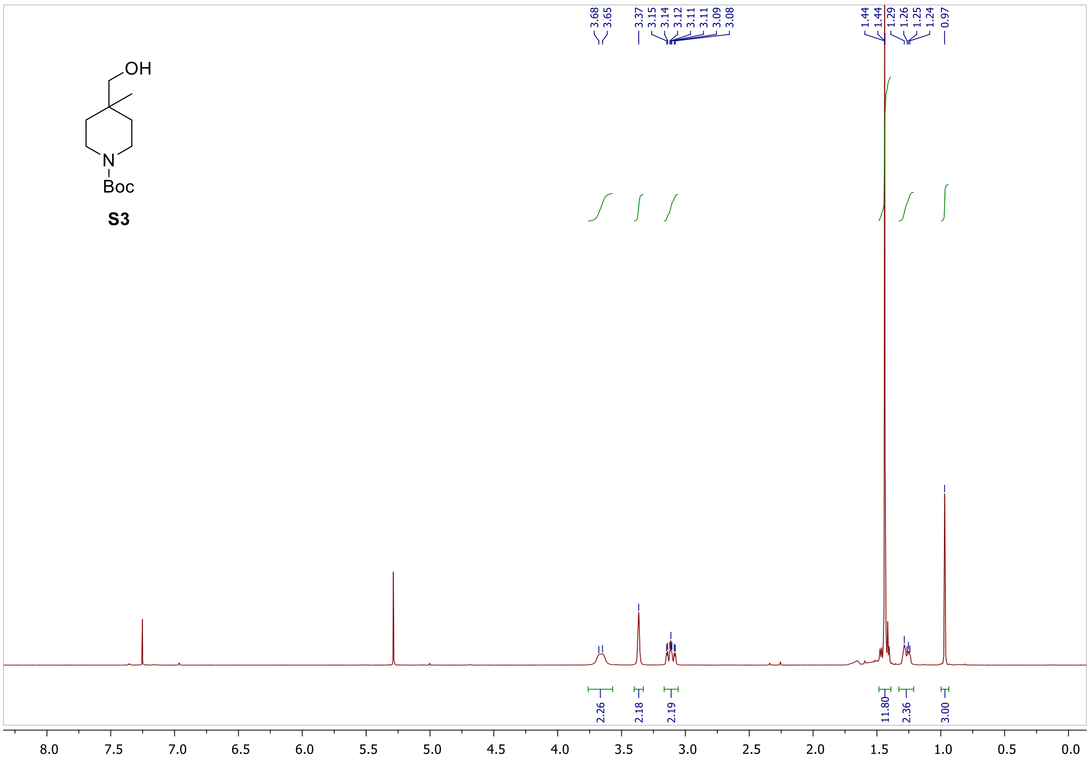

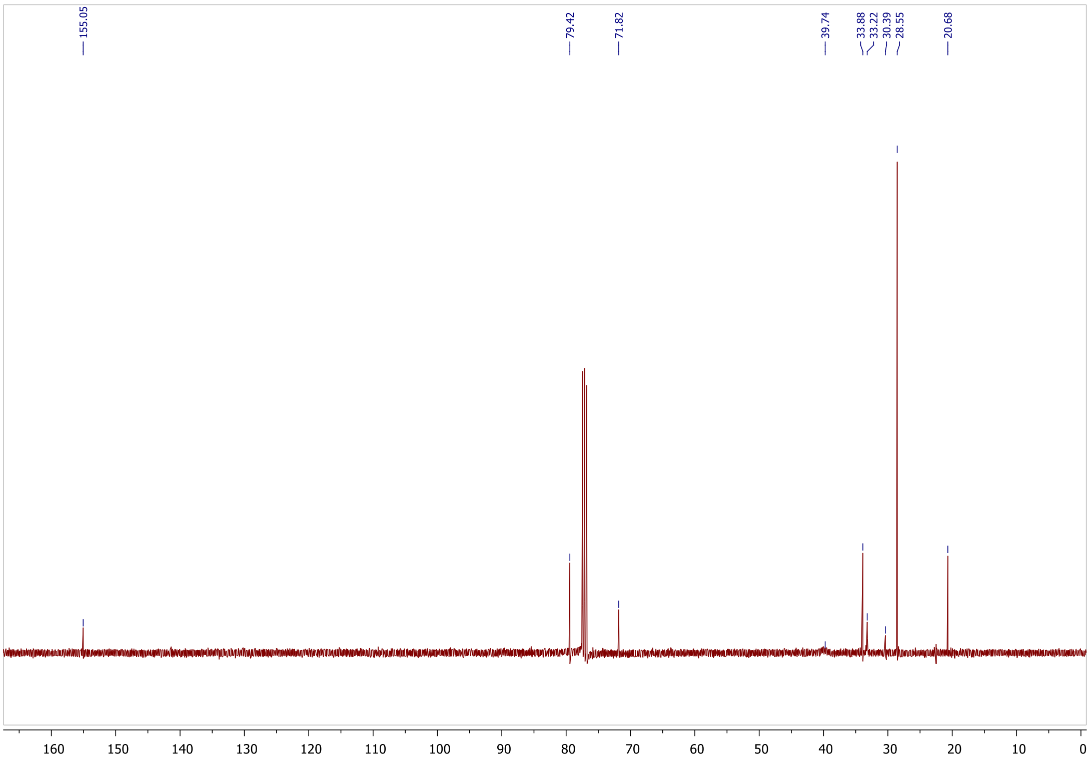

400 MHz ^{1}H NMR spectrum; 100.6 MHz ^{13}C NMR spectrum; DMSO-d_6

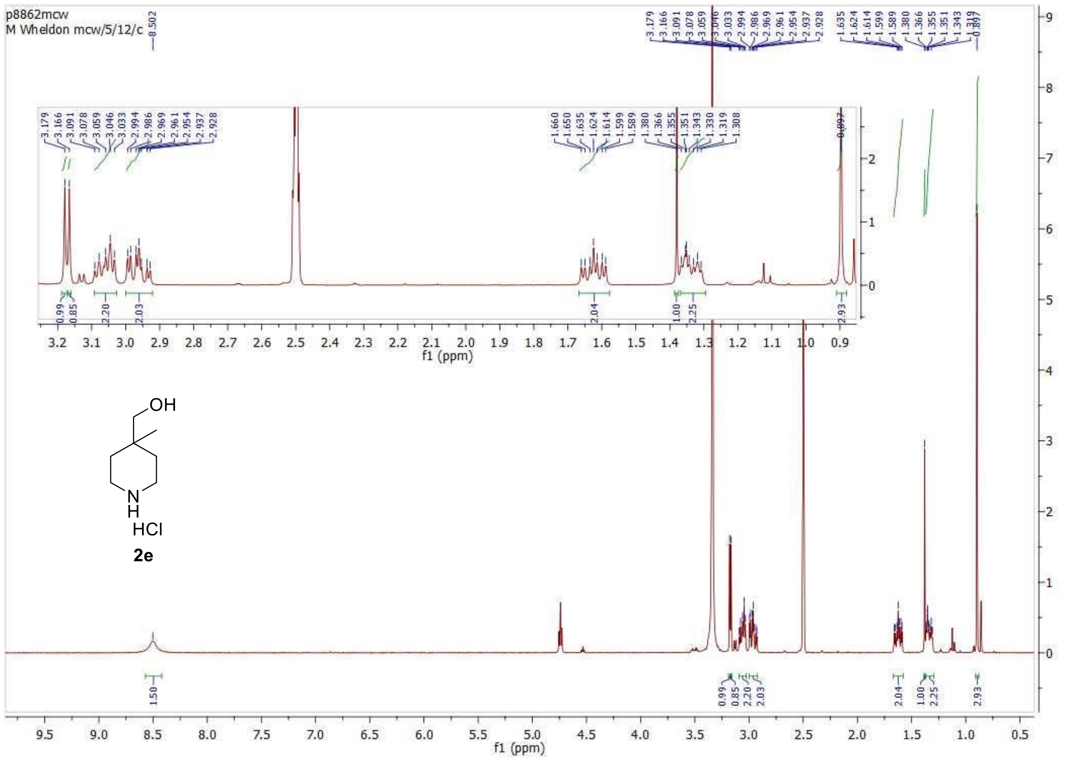

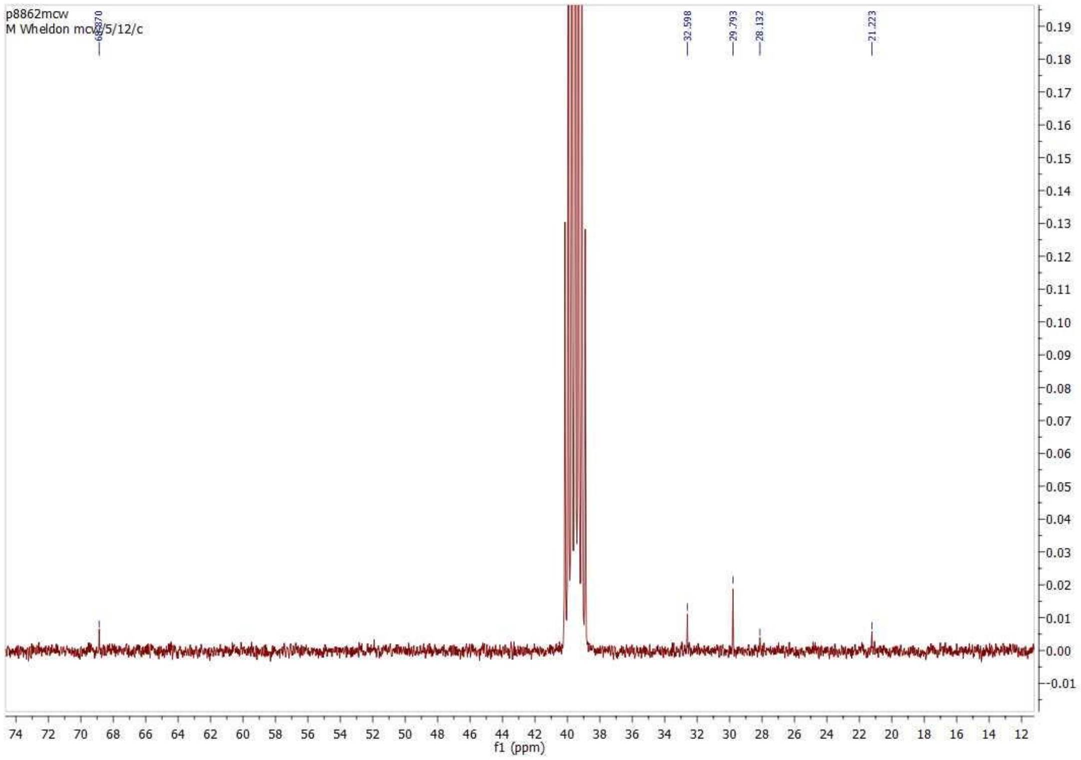

400 MHz ^{1}H NMR spectrum; 100.6 MHz ^{13}C NMR spectrum; $CDCl_3$

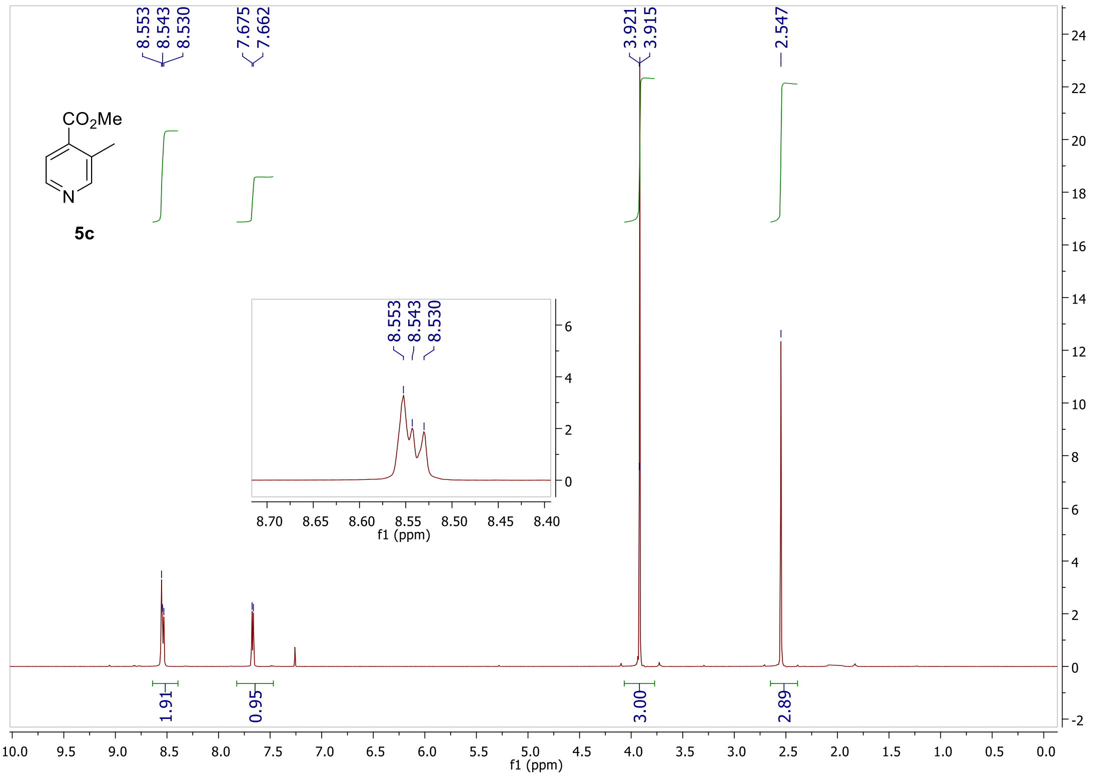

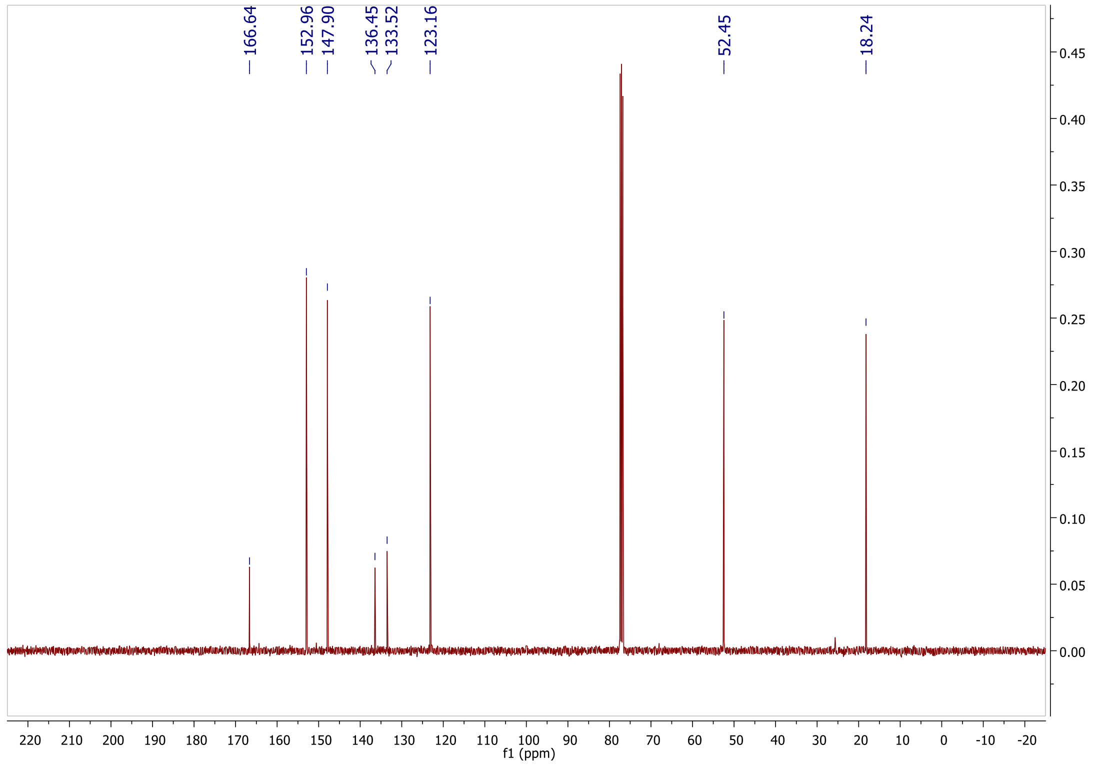

400 MHz ^{1}H NMR spectrum; 100.6 MHz ^{13}C NMR spectrum; $CDCl_3$

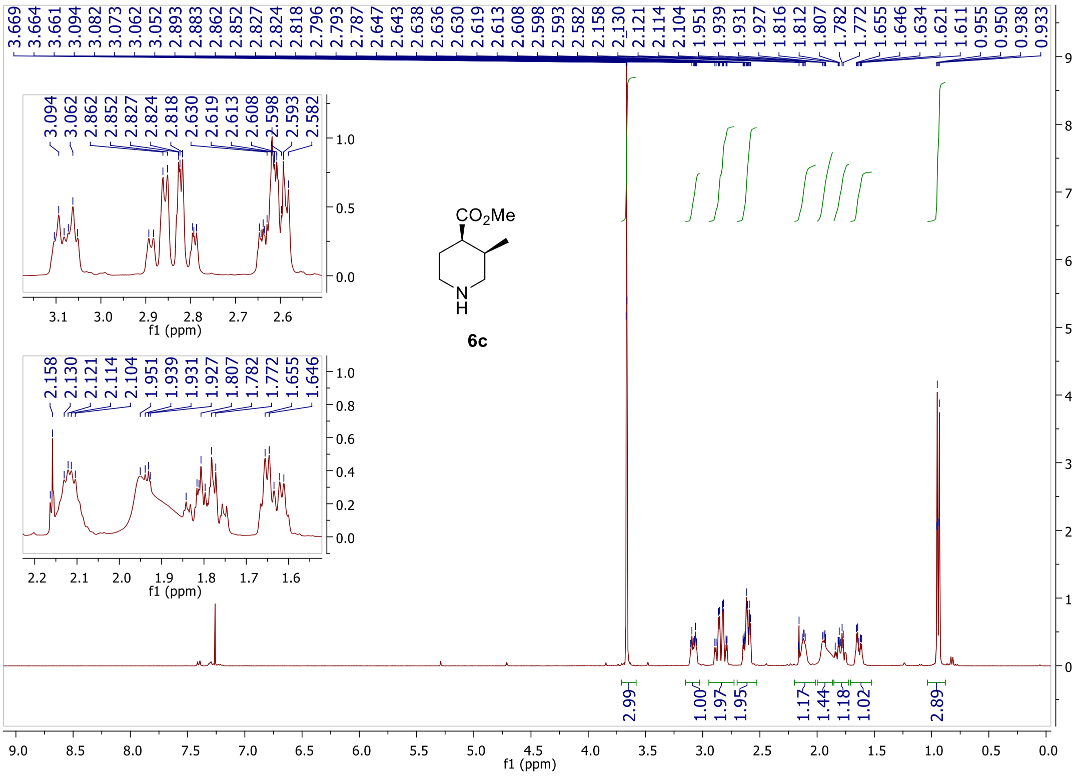

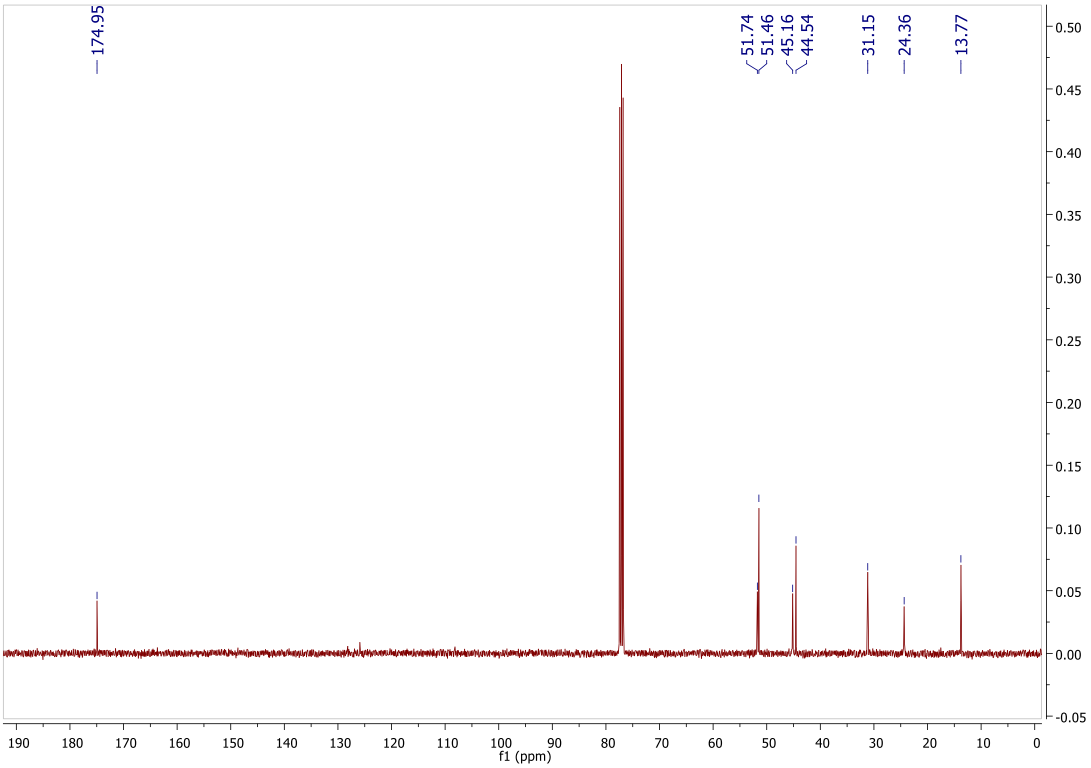

400 MHz ^{1}H NMR spectrum; 100.6 MHz ^{13}C NMR spectrum; MeOH-*d*4

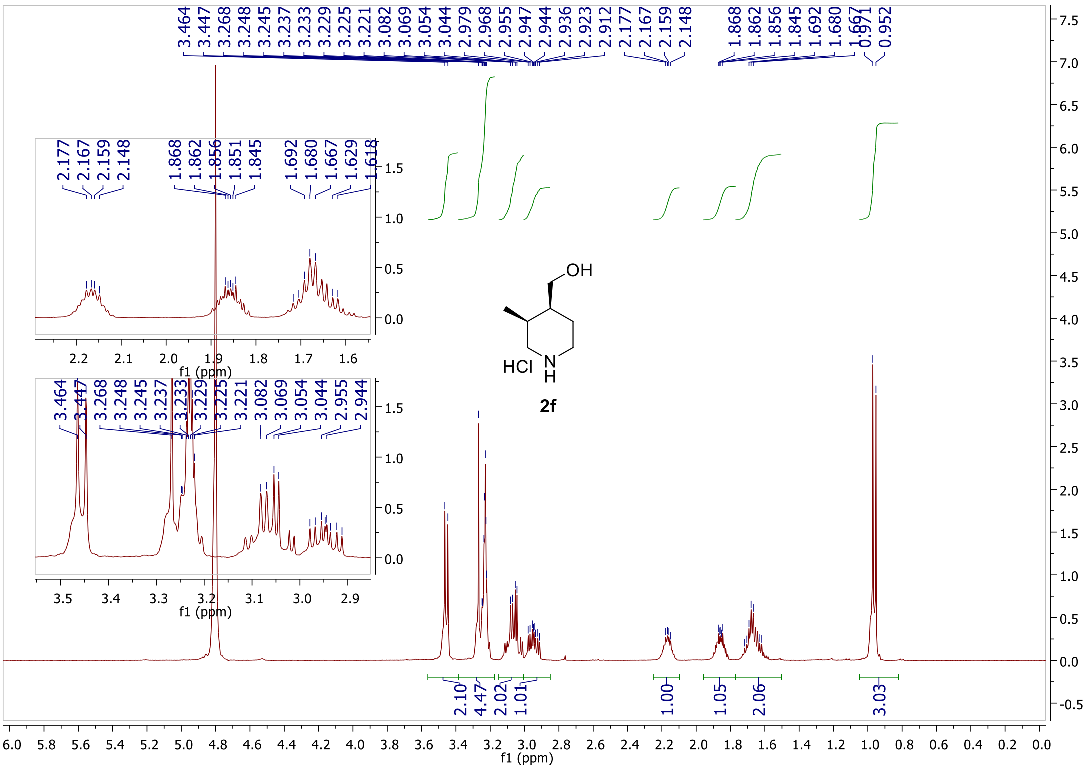

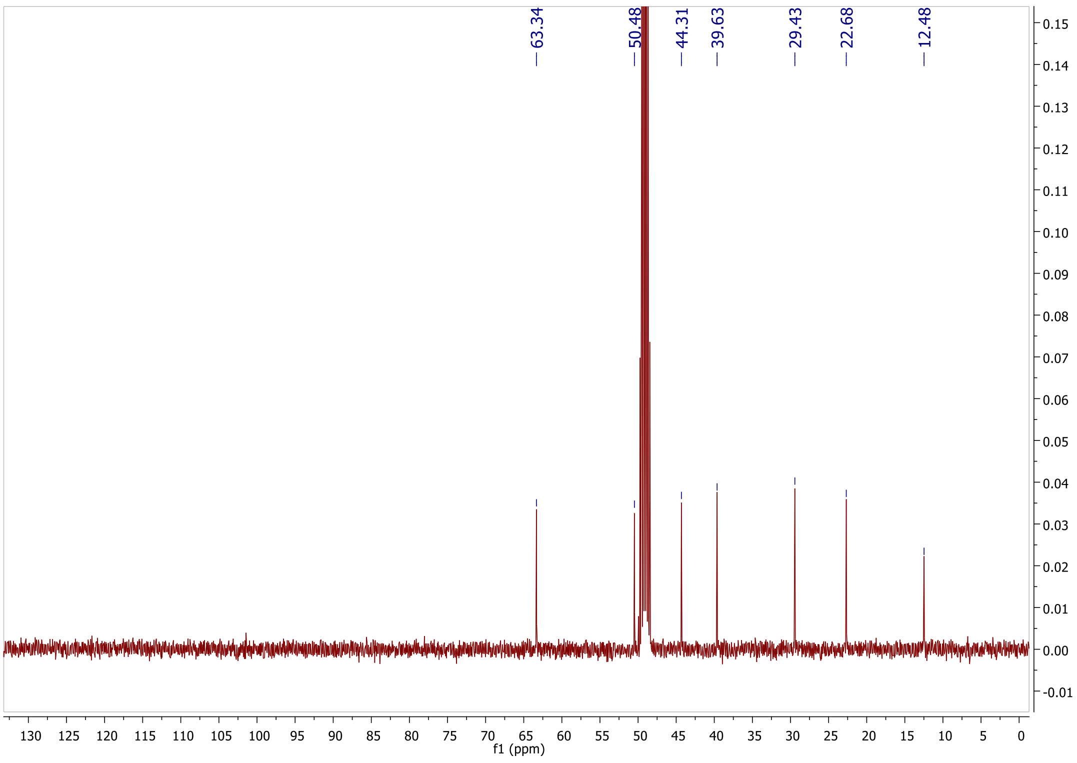

400 MHz ^{1}H NMR spectrum; 100.6 MHz ^{13}C NMR spectrum; $CDCl_3$

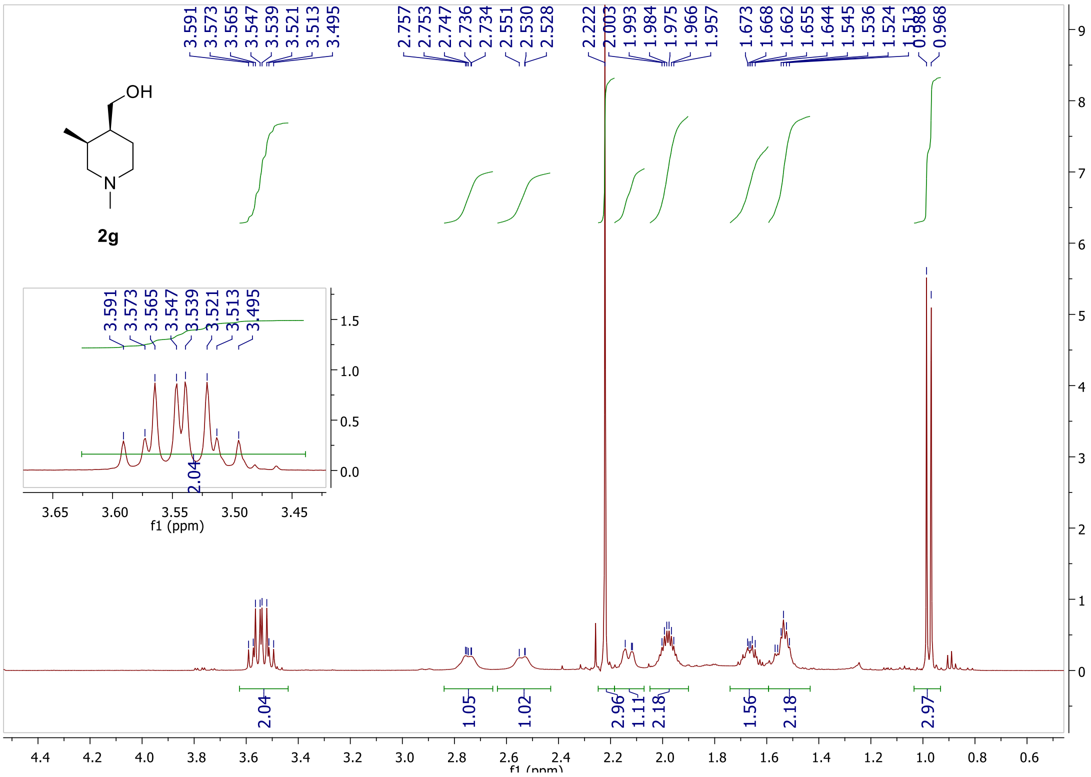

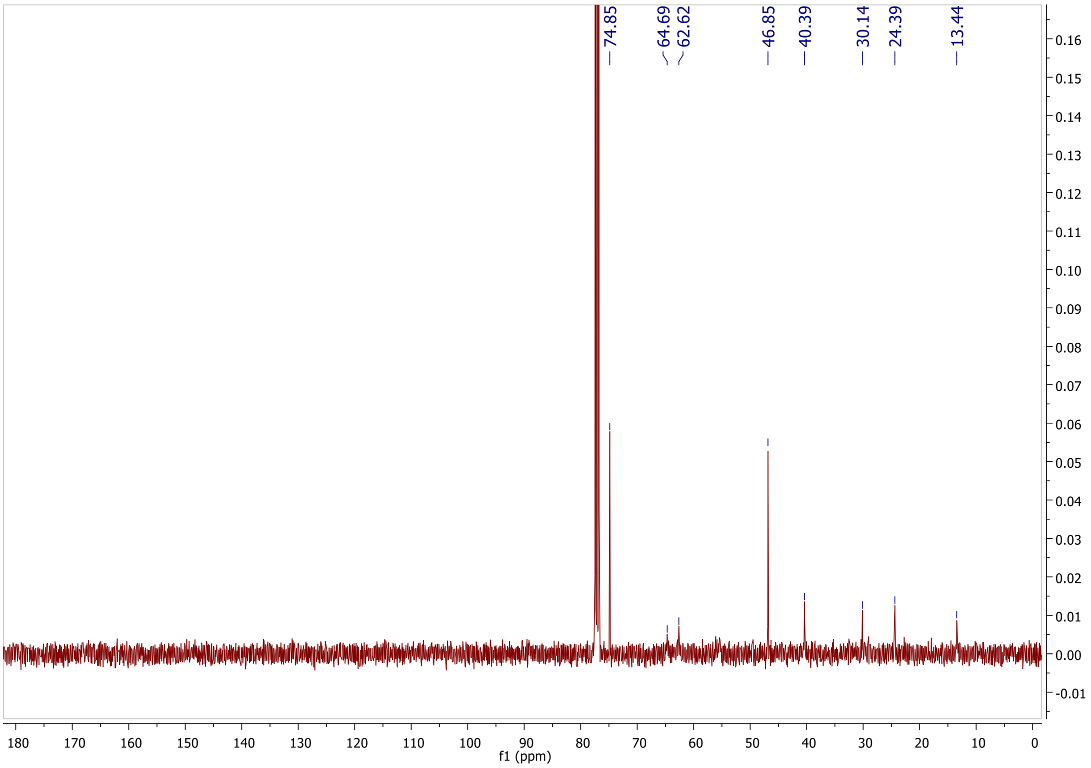

400 MHz ^{1}H NMR spectrum; 100.6 MHz ^{13}C NMR spectrum; $CDCl_3$

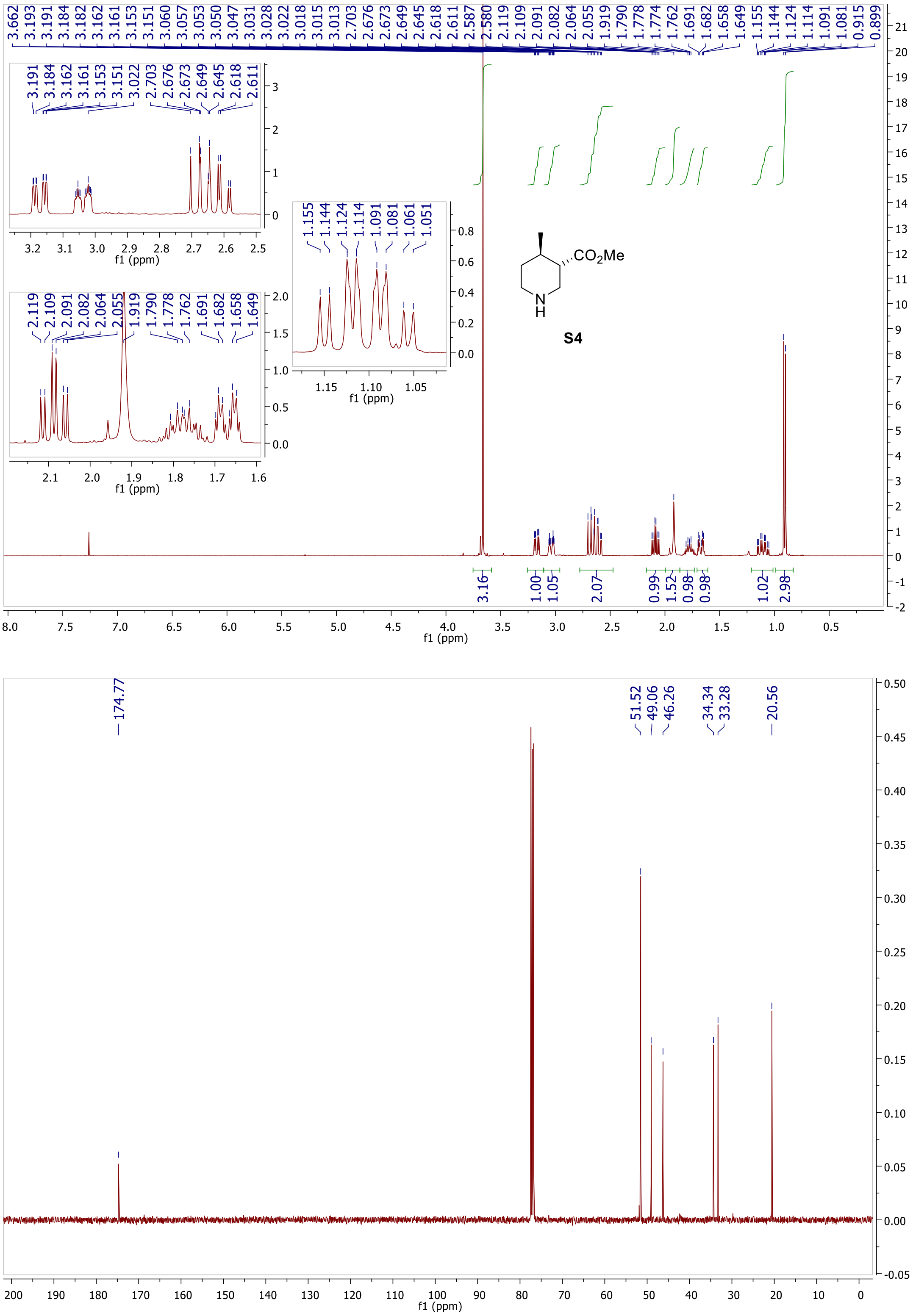

400 MHz ^{1}H NMR spectrum; 100.6 MHz ^{13}C NMR spectrum; $CDCl_3$

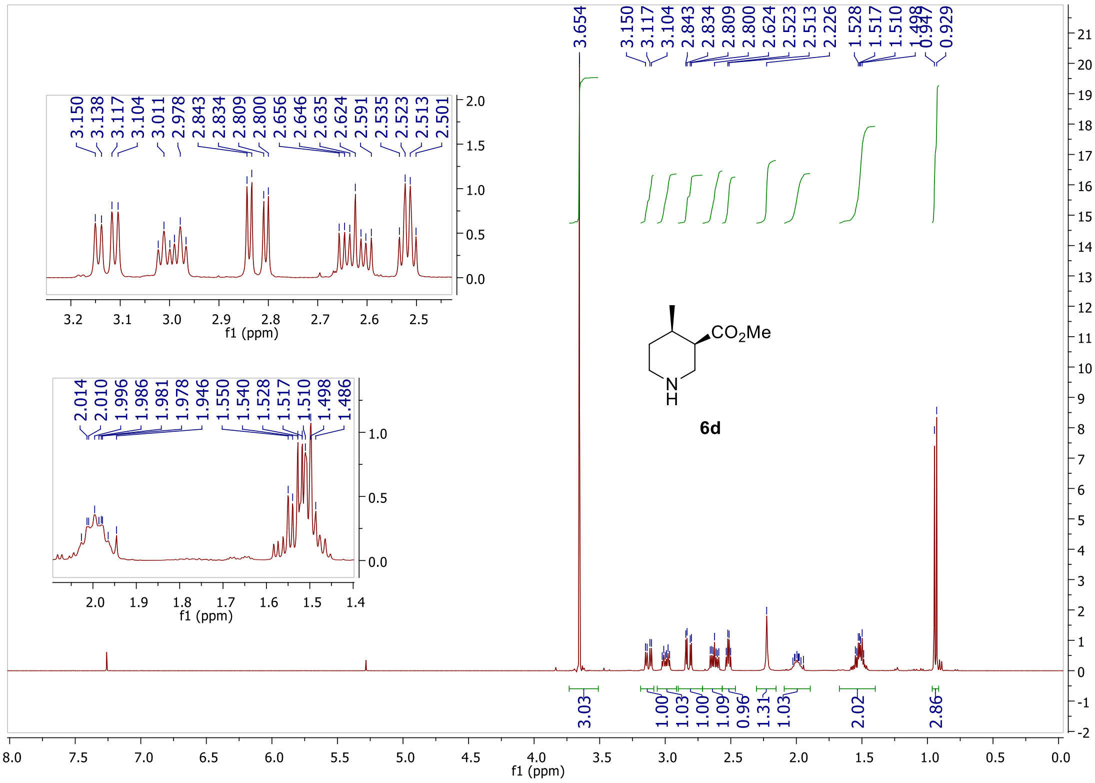

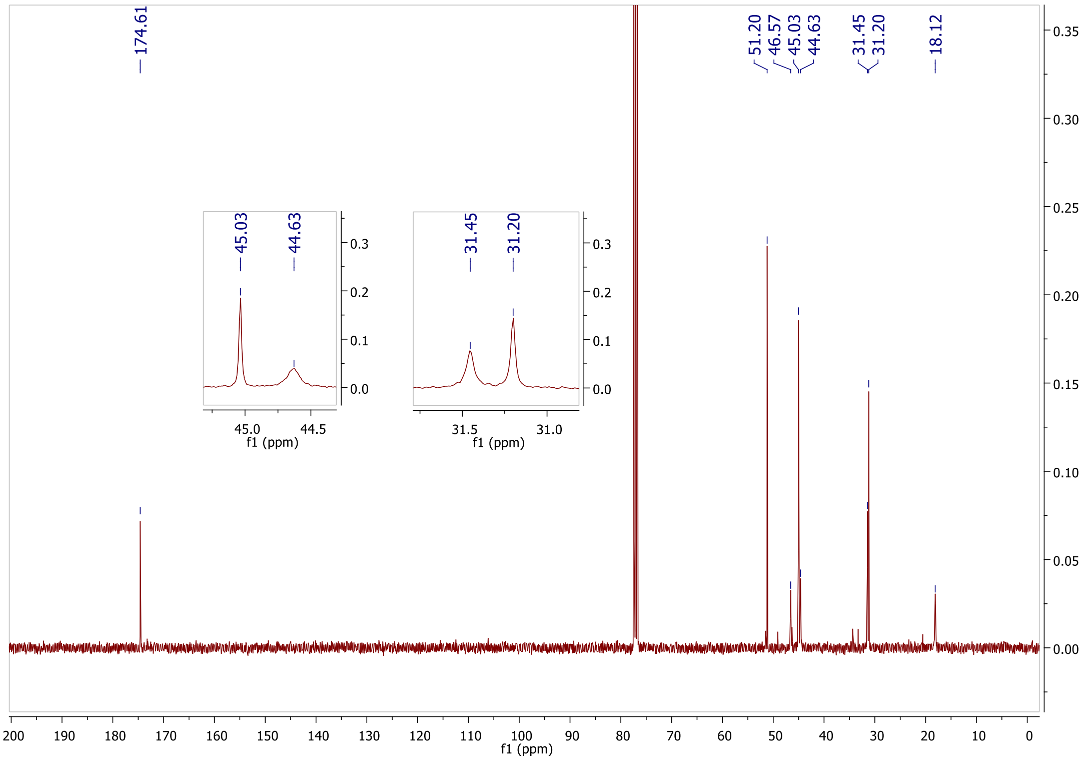

400 MHz ^{1}H NMR spectrum; 100.6 MHz ^{13}C NMR spectrum; MeOH-*d*4

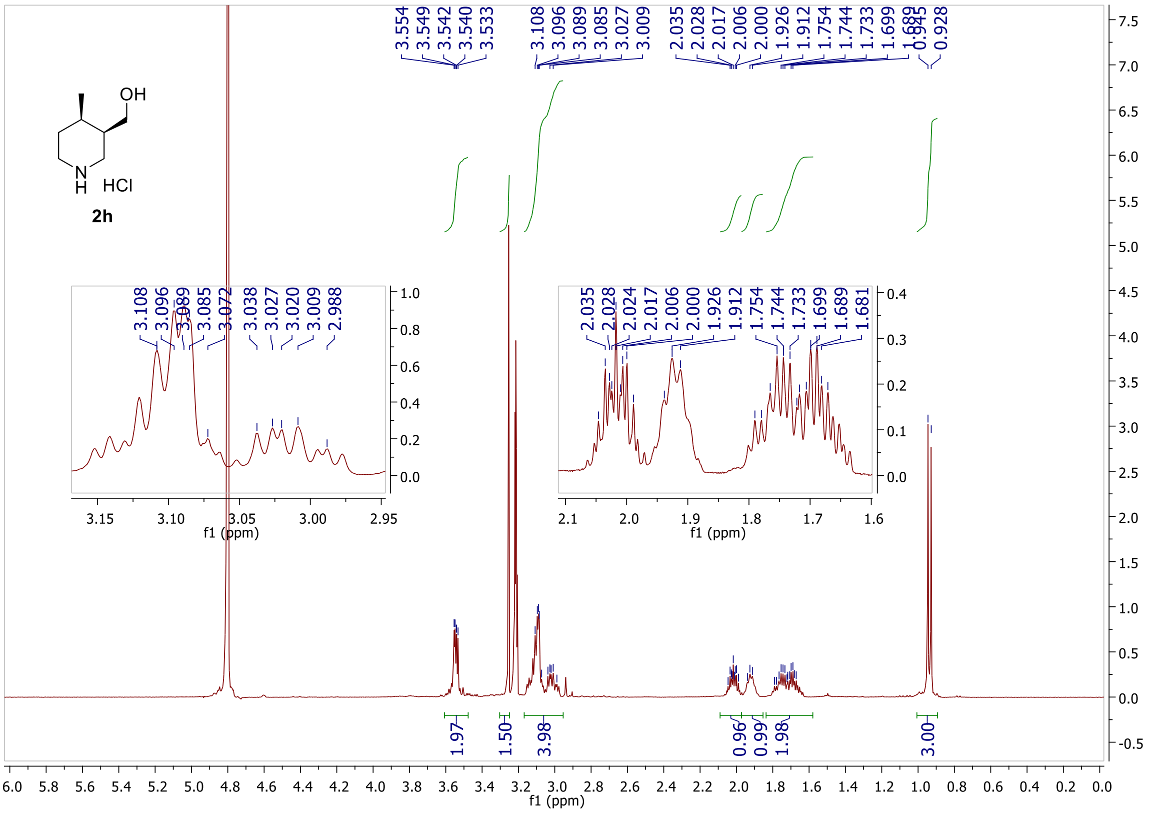

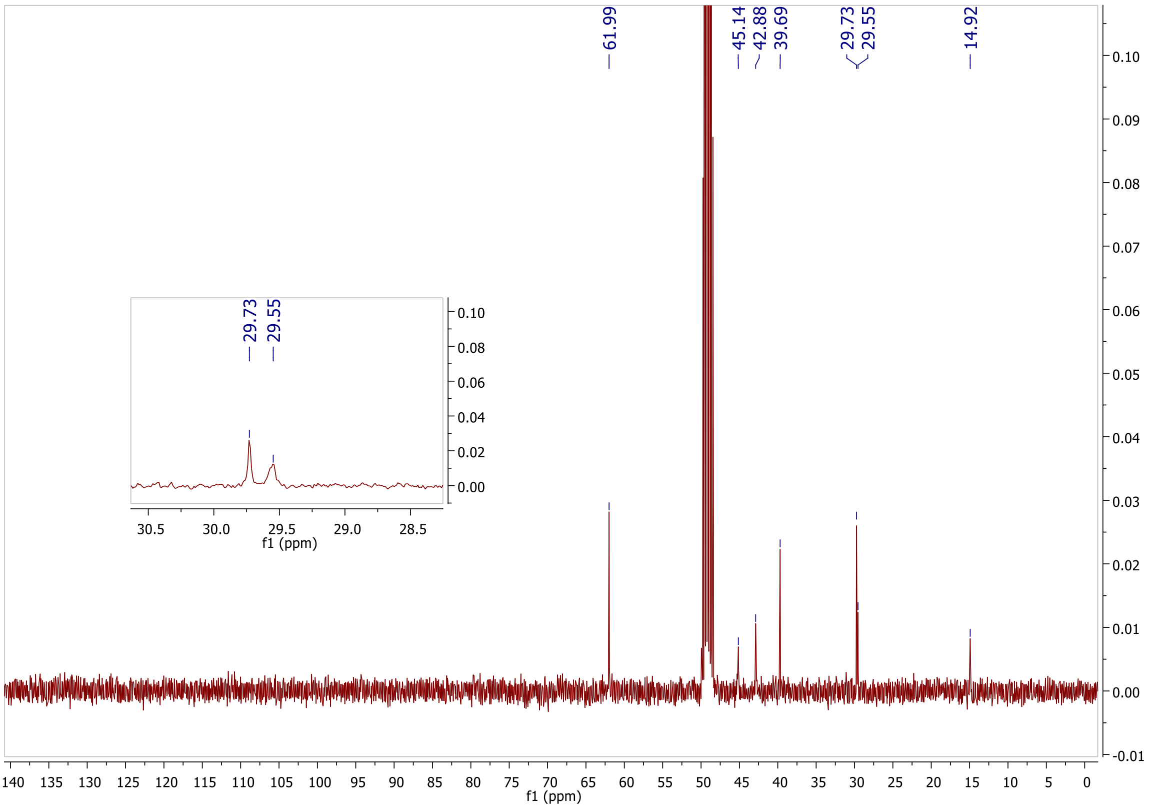

400 MHz ^{1}H NMR spectrum; 100.6 MHz ^{13}C NMR spectrum; $CDCl_3$

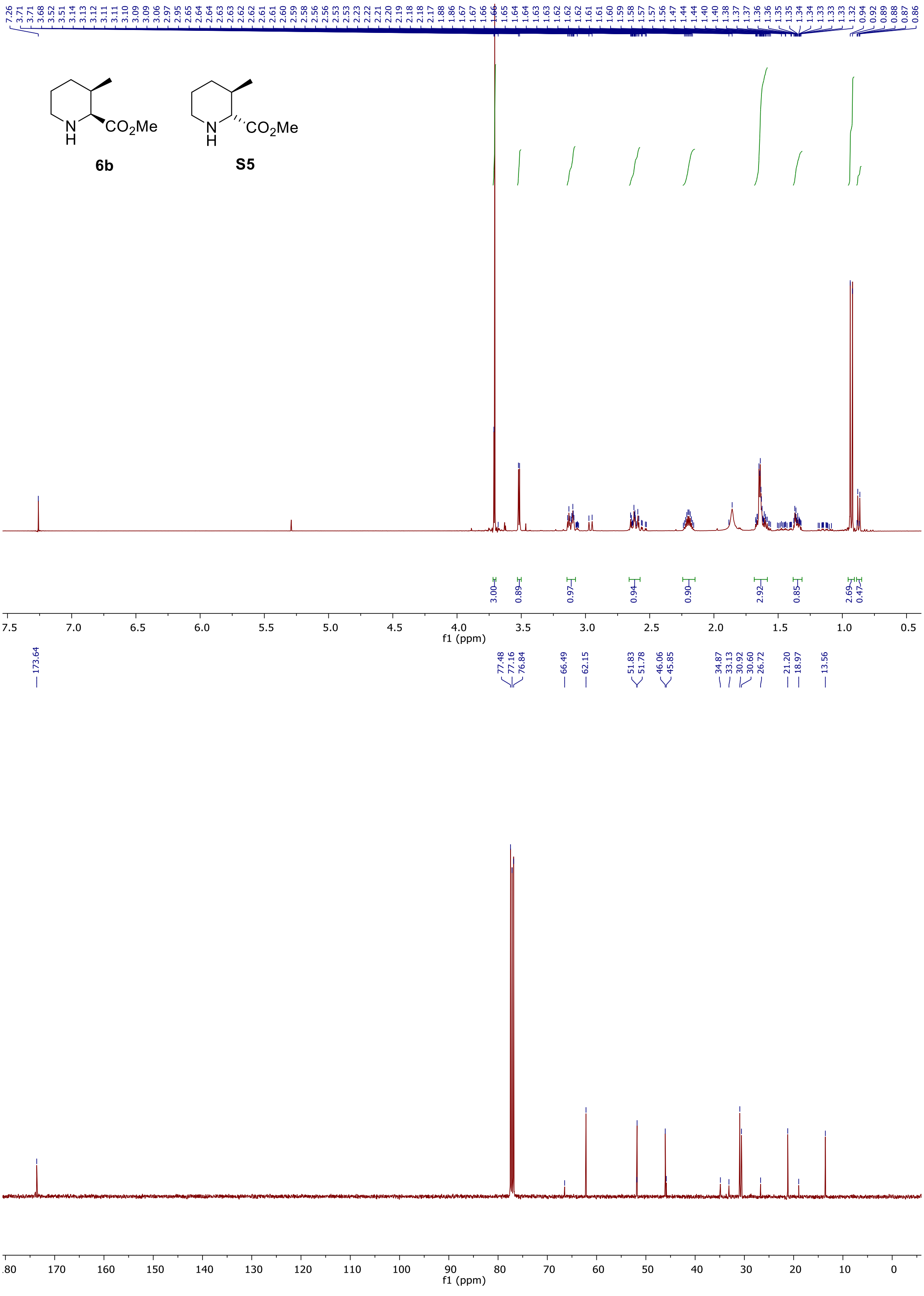

400 MHz ^{1}H NMR spectrum; 100.6 MHz ^{13}C NMR spectrum; $CDCl_3$

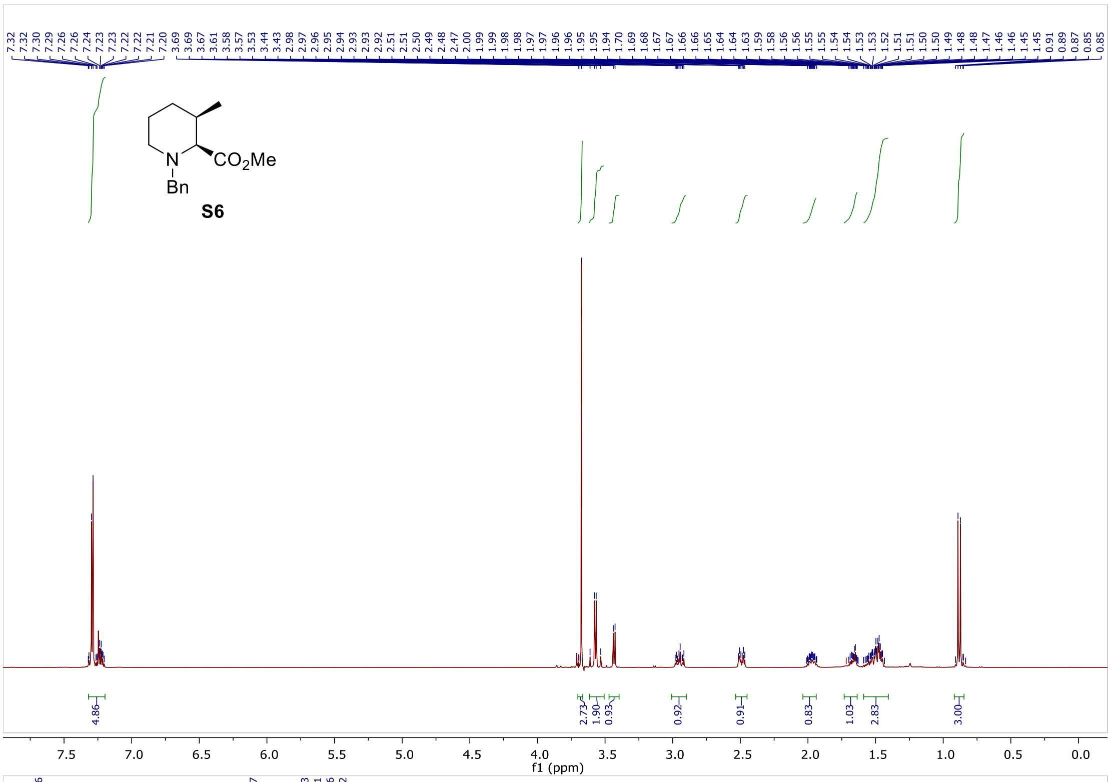

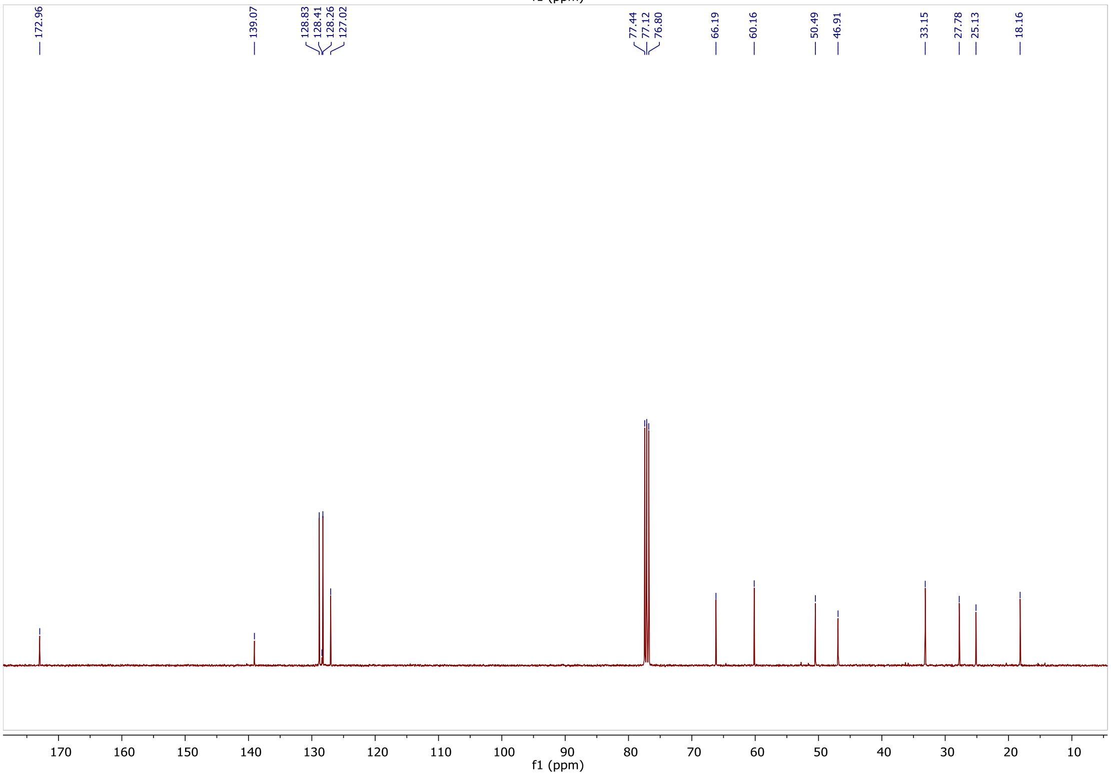

400 MHz ^{1}H NMR spectrum; 100.6 MHz ^{13}C NMR spectrum; $CDCl_3$

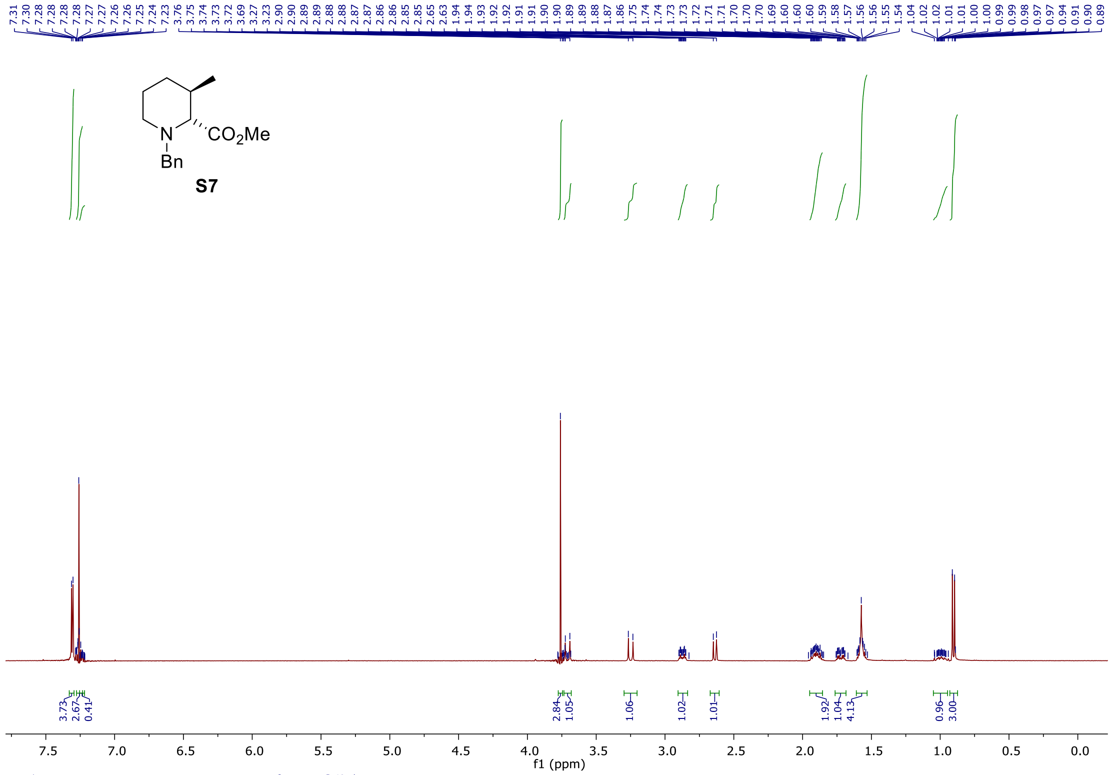

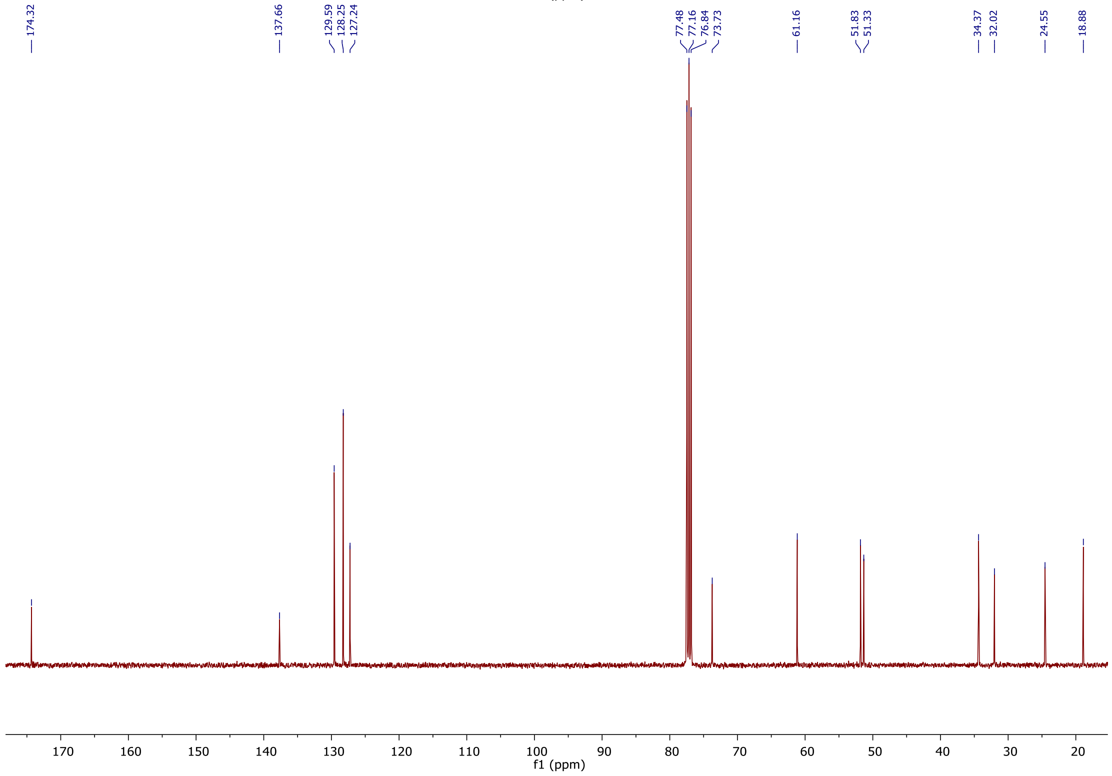

400 MHz ^{1}H NMR spectrum; 100.6 MHz ^{13}C NMR spectrum; $CDCl_3$

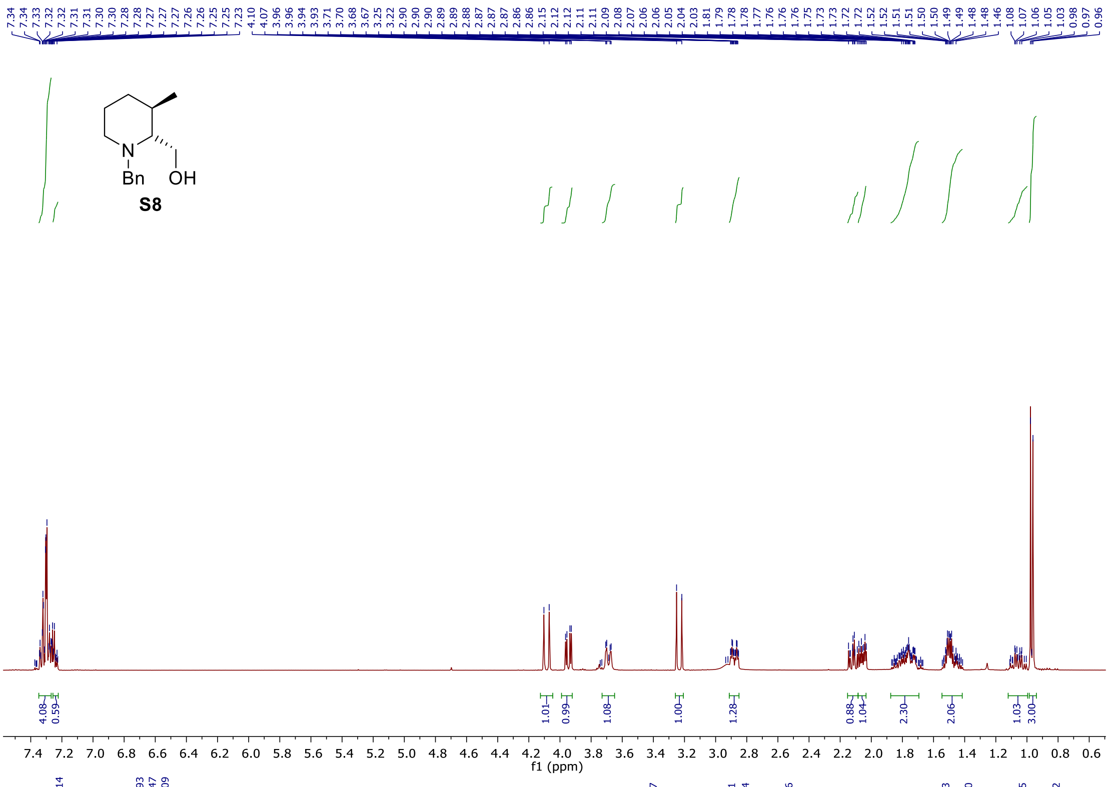

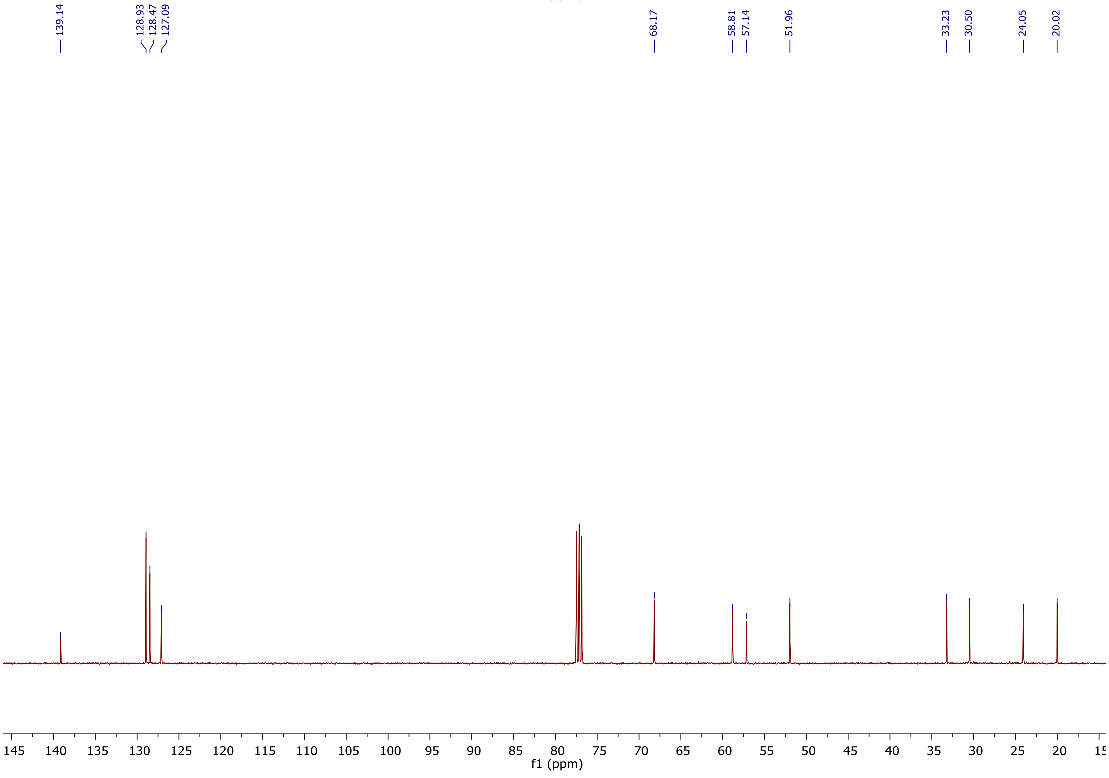

400 MHz ^{1}H NMR spectrum; 100.6 MHz ^{13}C NMR spectrum; $CDCl_3$

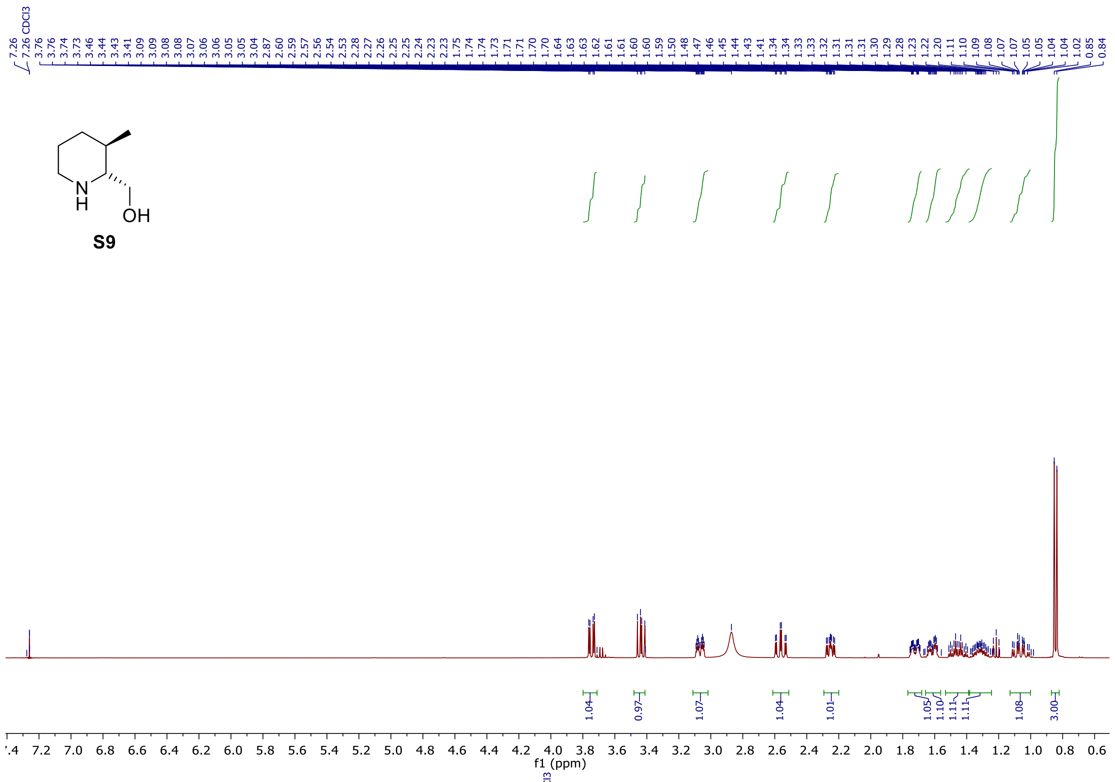

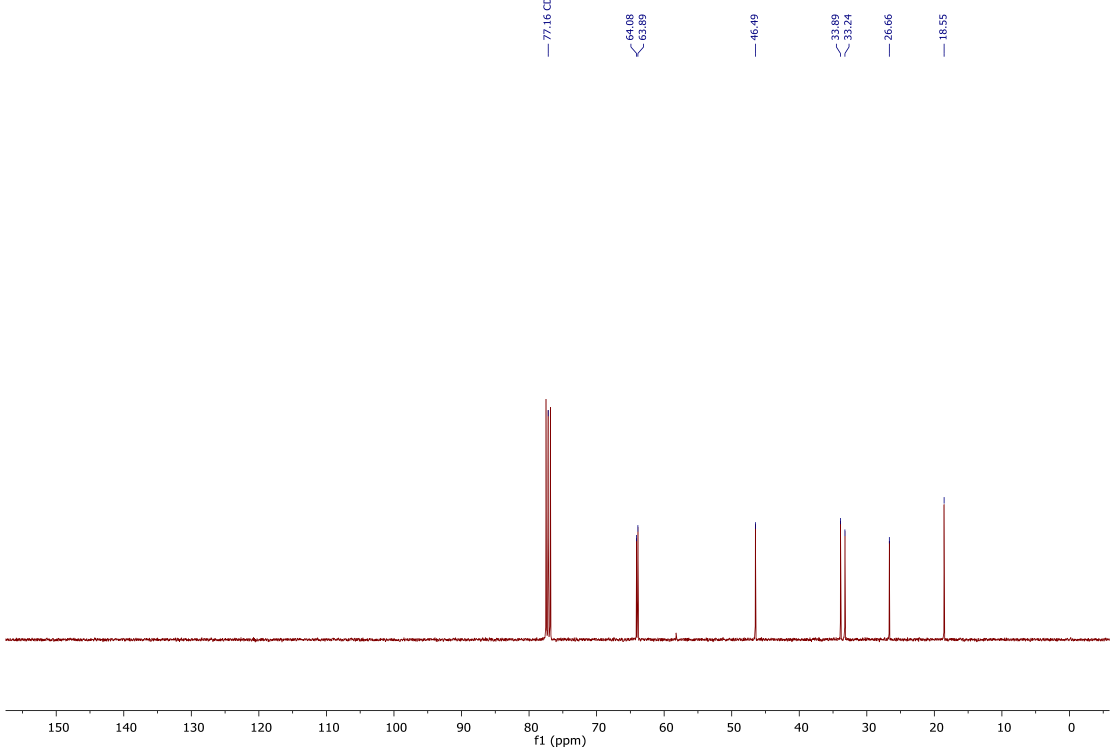

400 MHz ^{1}H NMR spectrum; 100.6 MHz ^{13}C NMR spectrum; $CDCl_3$

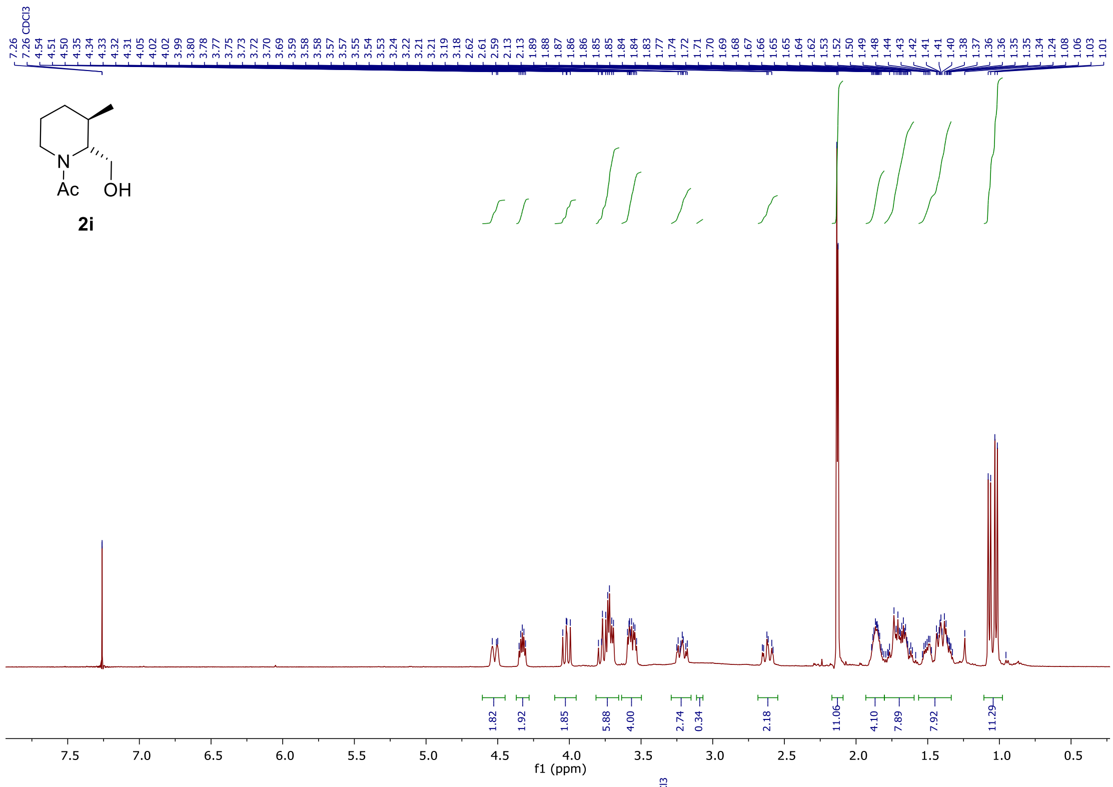

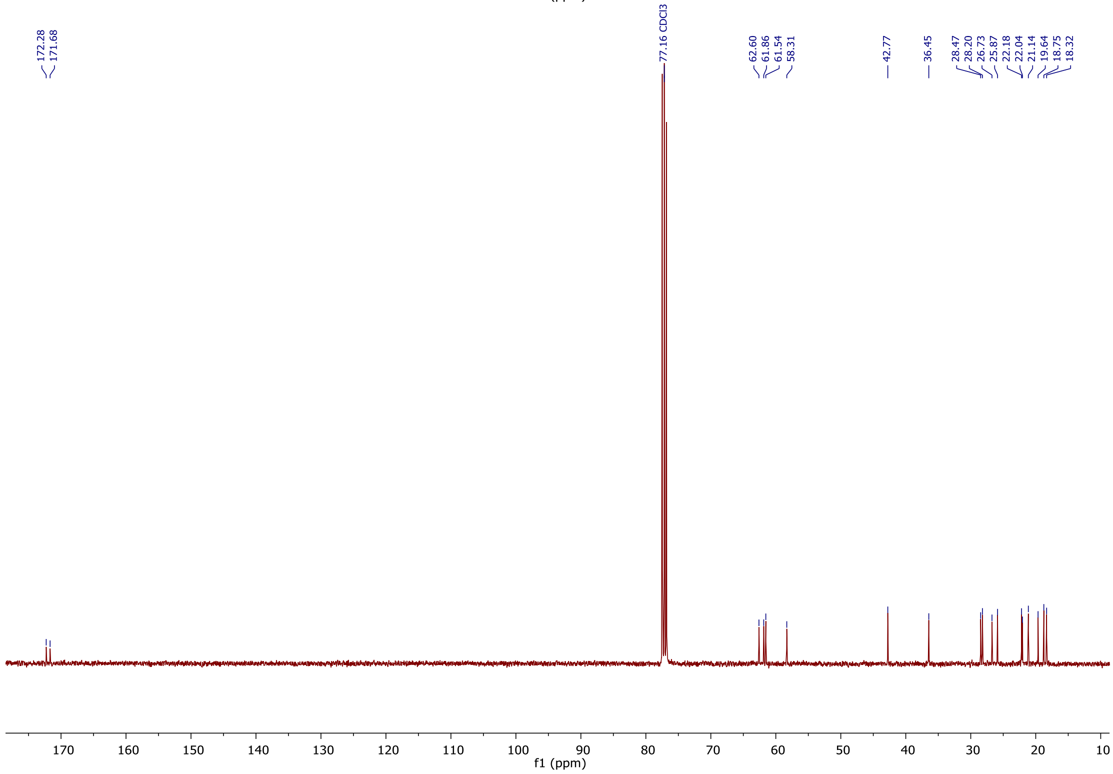

400 MHz ^{1}H NMR spectrum; 100.6 MHz ^{13}C NMR spectrum; $CDCl_3$

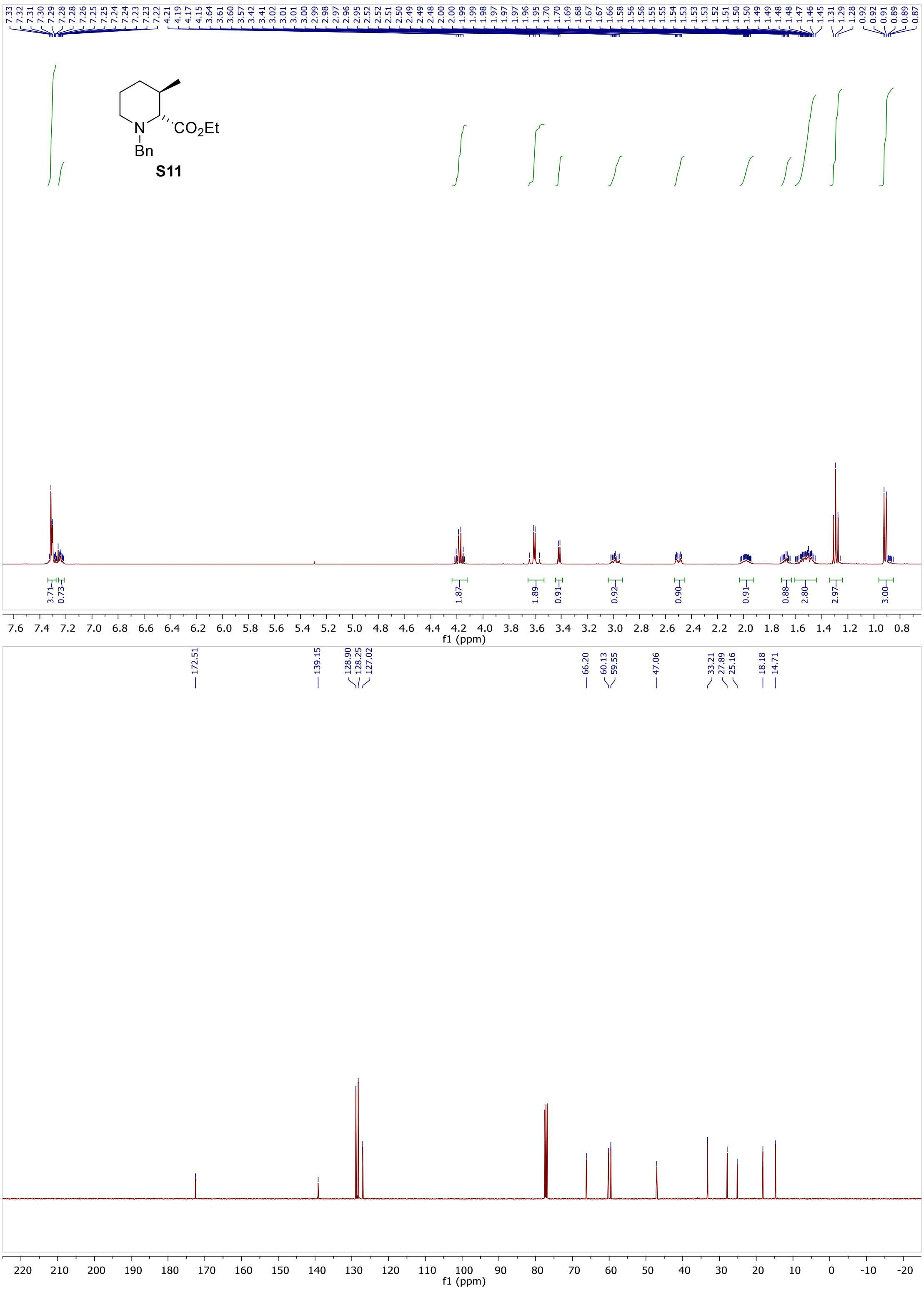

400 MHz ^{1}H NMR spectrum; 100.6 MHz ^{13}C NMR spectrum; $CDCl_3$

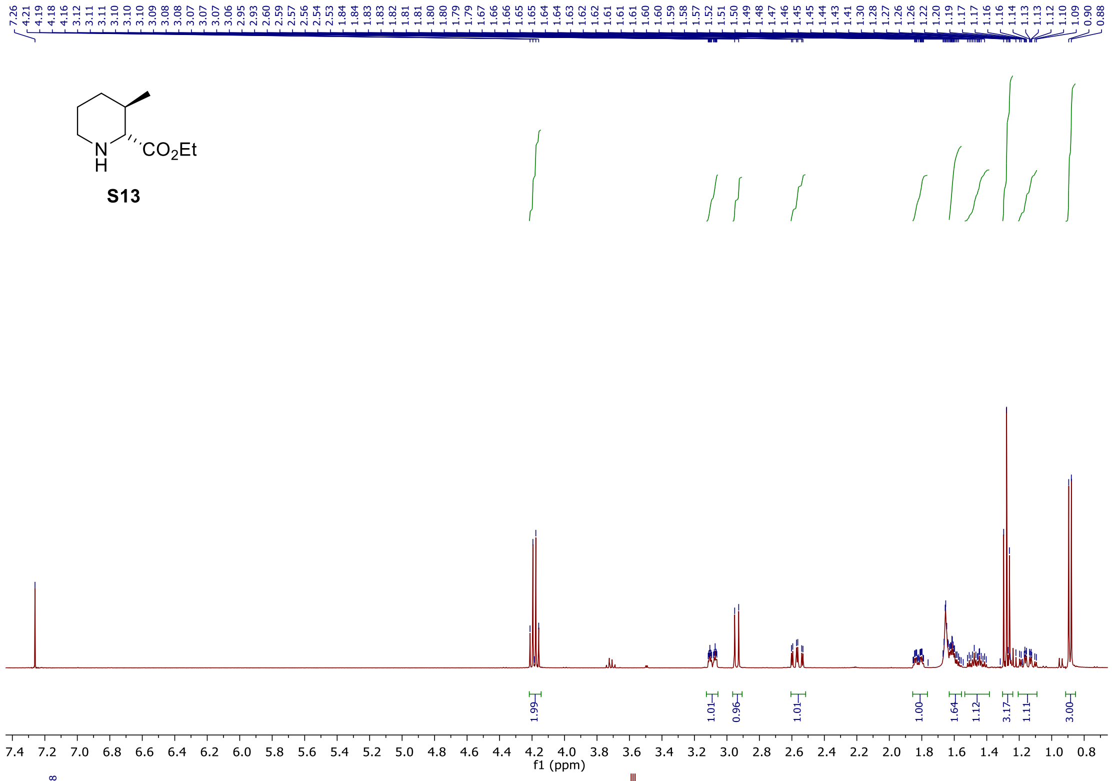

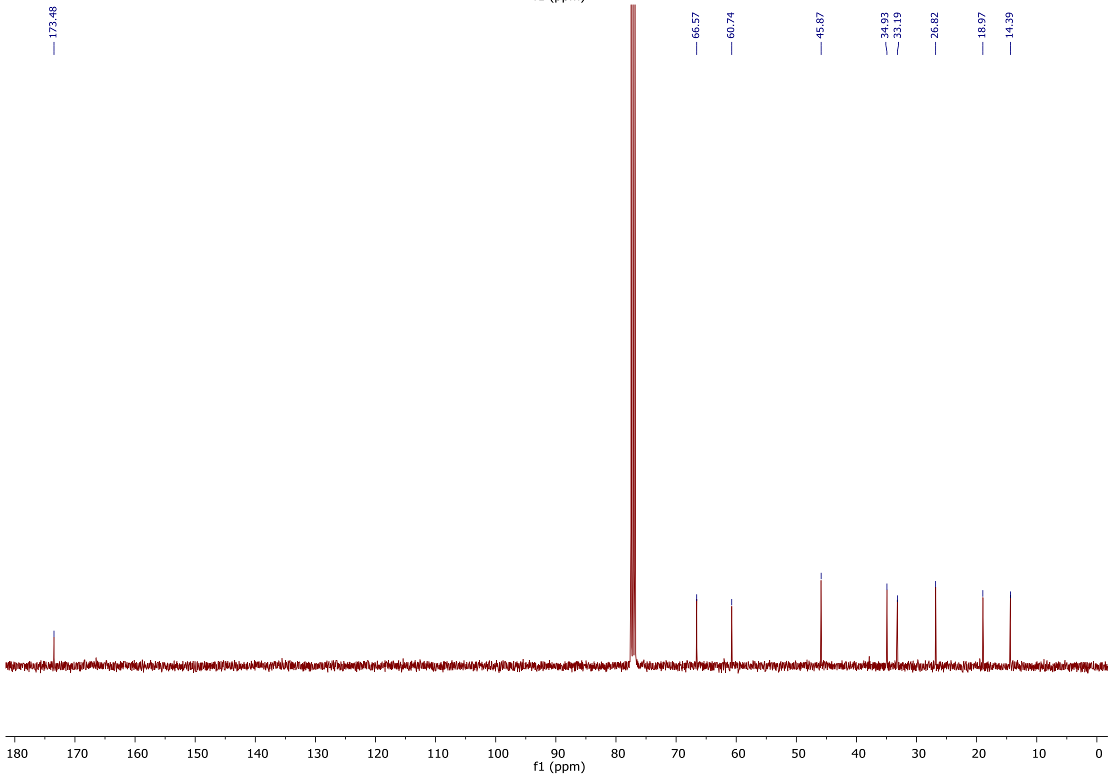

400 MHz ^{1}H NMR spectrum; 100.6 MHz ^{13}C NMR spectrum; $CDCl_3$

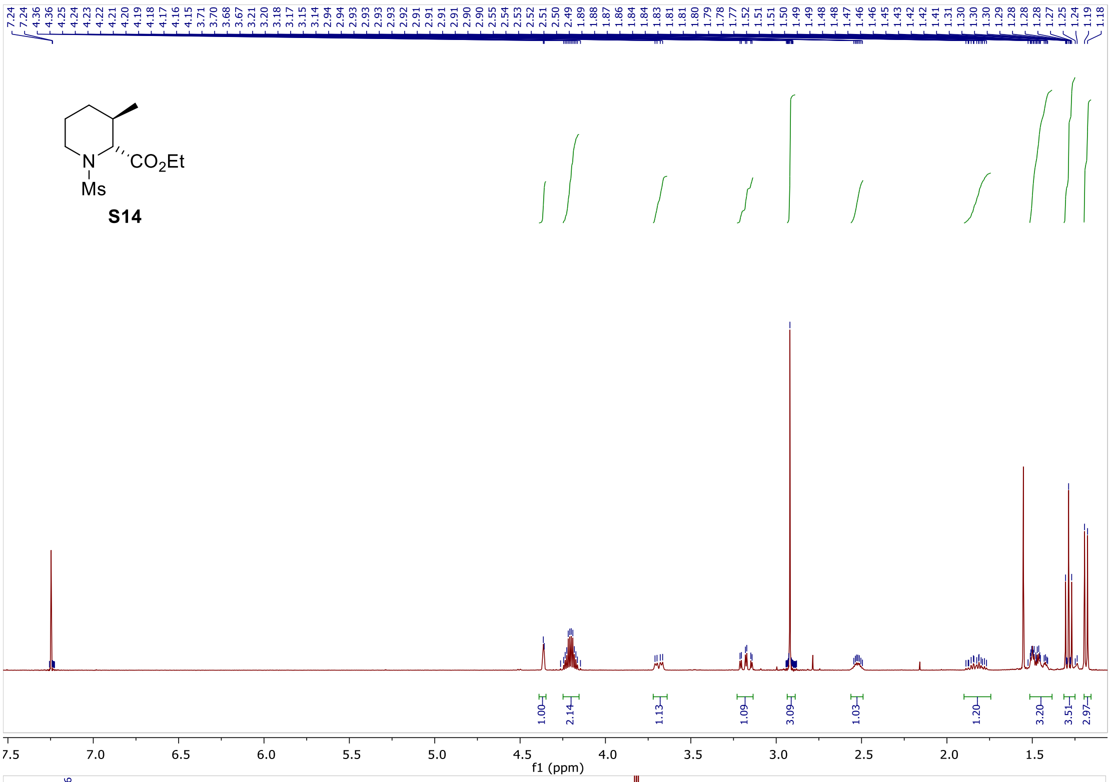

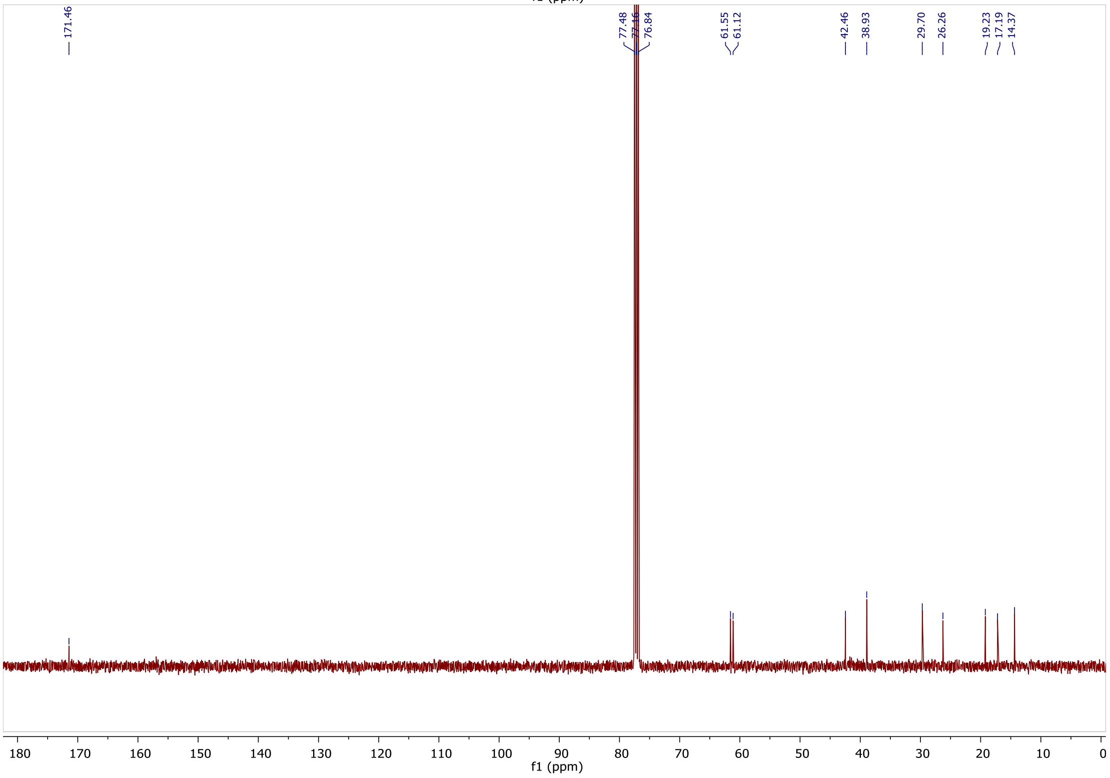

400 MHz ^{1}H NMR spectrum; 100.6 MHz ^{13}C NMR spectrum; $CDCl_3$

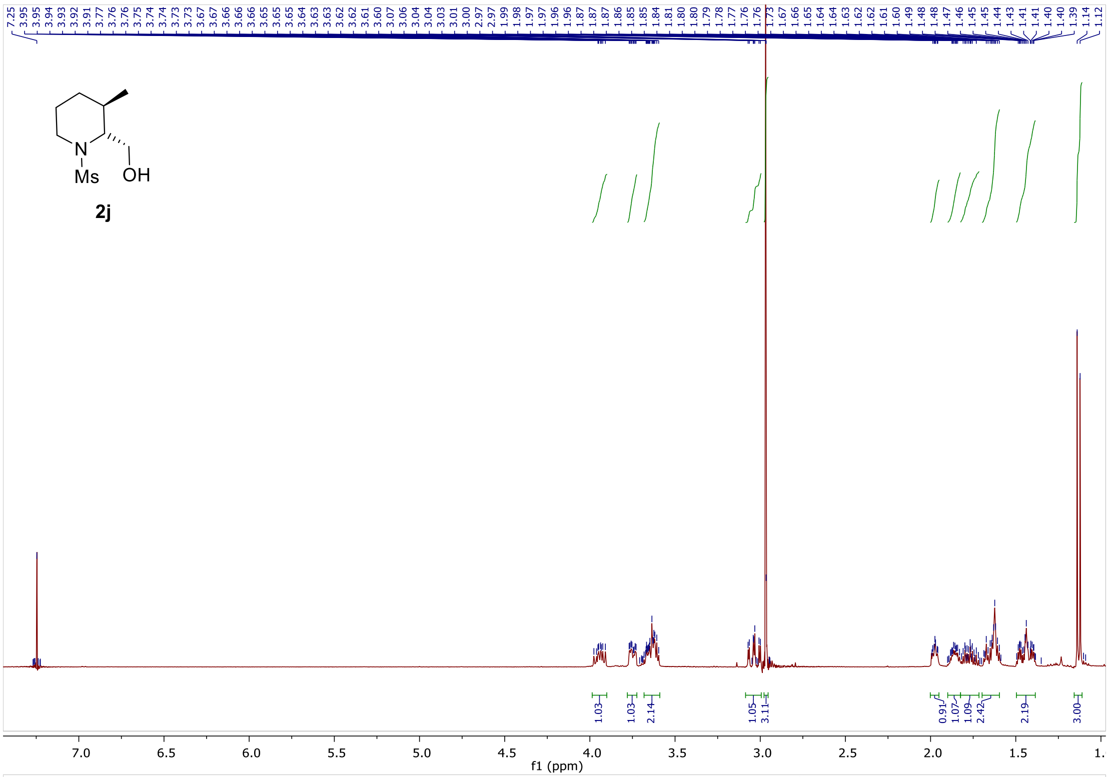

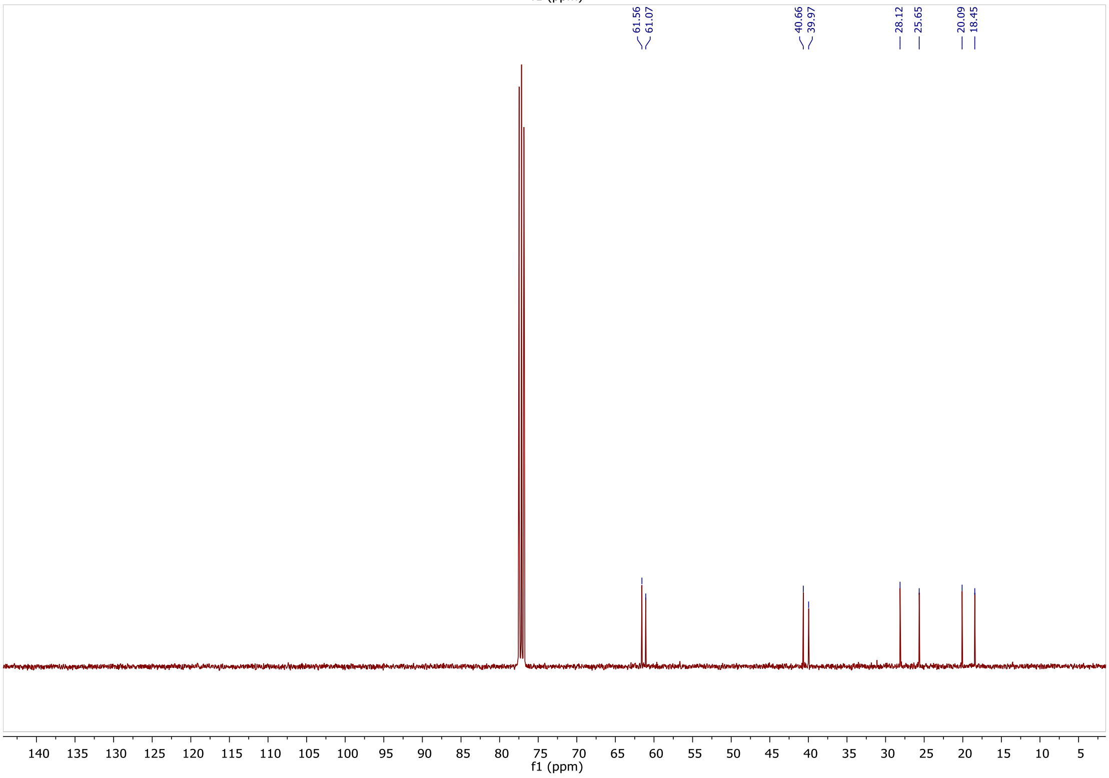

400 MHz ^{1}H NMR spectrum; 100.6 MHz ^{13}C NMR spectrum; $CDCl_3$

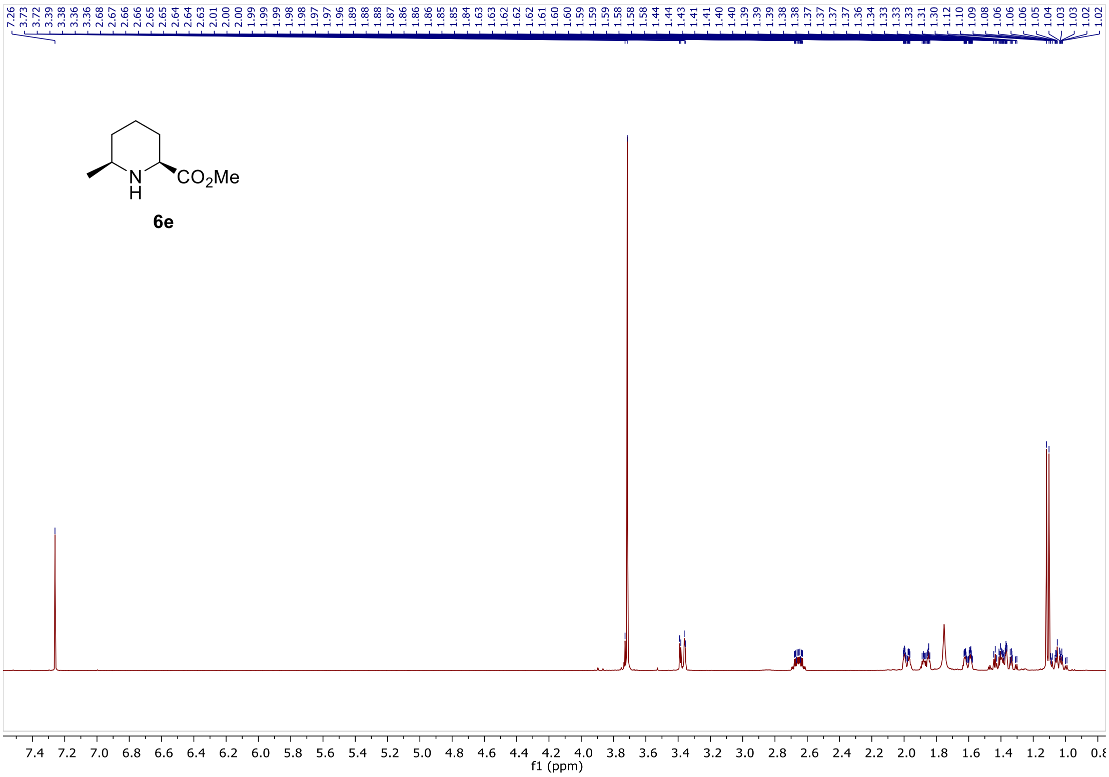

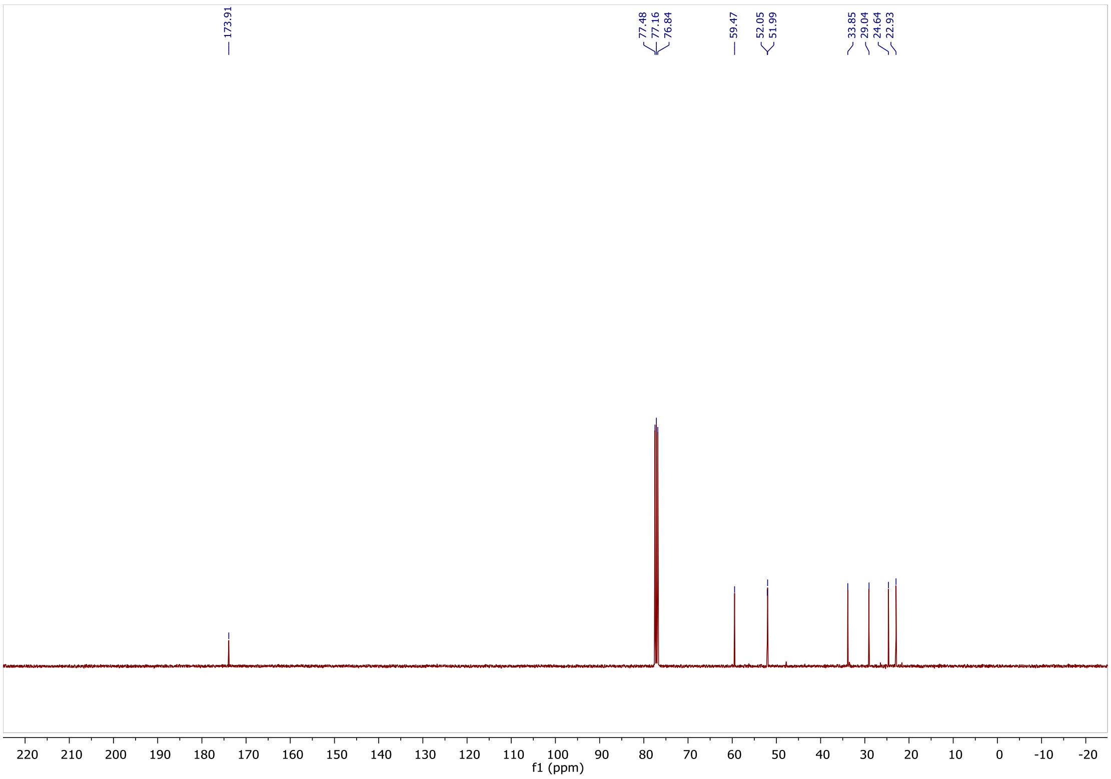

400 MHz ^{1}H NMR spectrum; 100.6 MHz ^{13}C NMR spectrum; $CDCl_3$

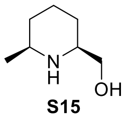

S15

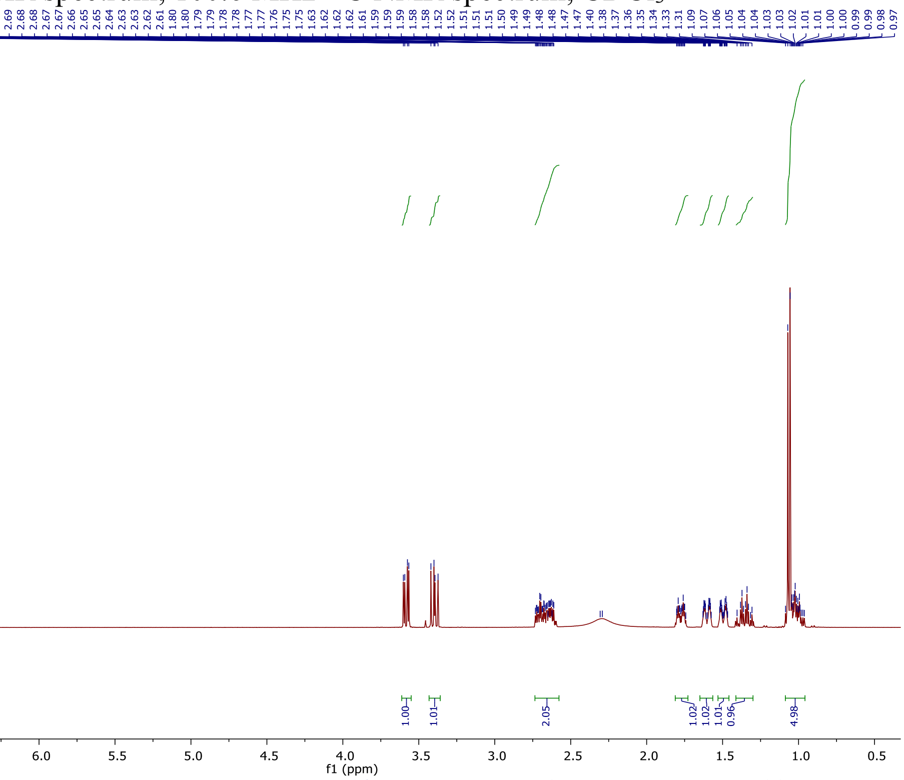

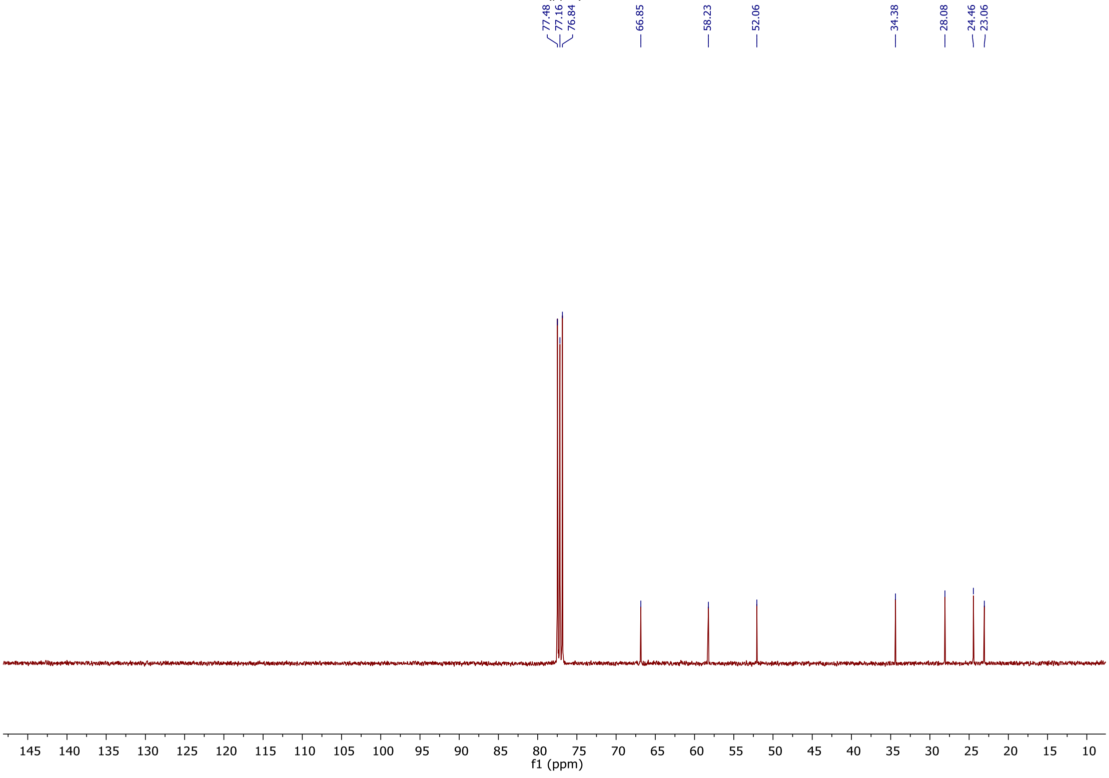

400 MHz ^{1}H NMR spectrum; 100.6 MHz ^{13}C NMR spectrum; $CDCl_3$

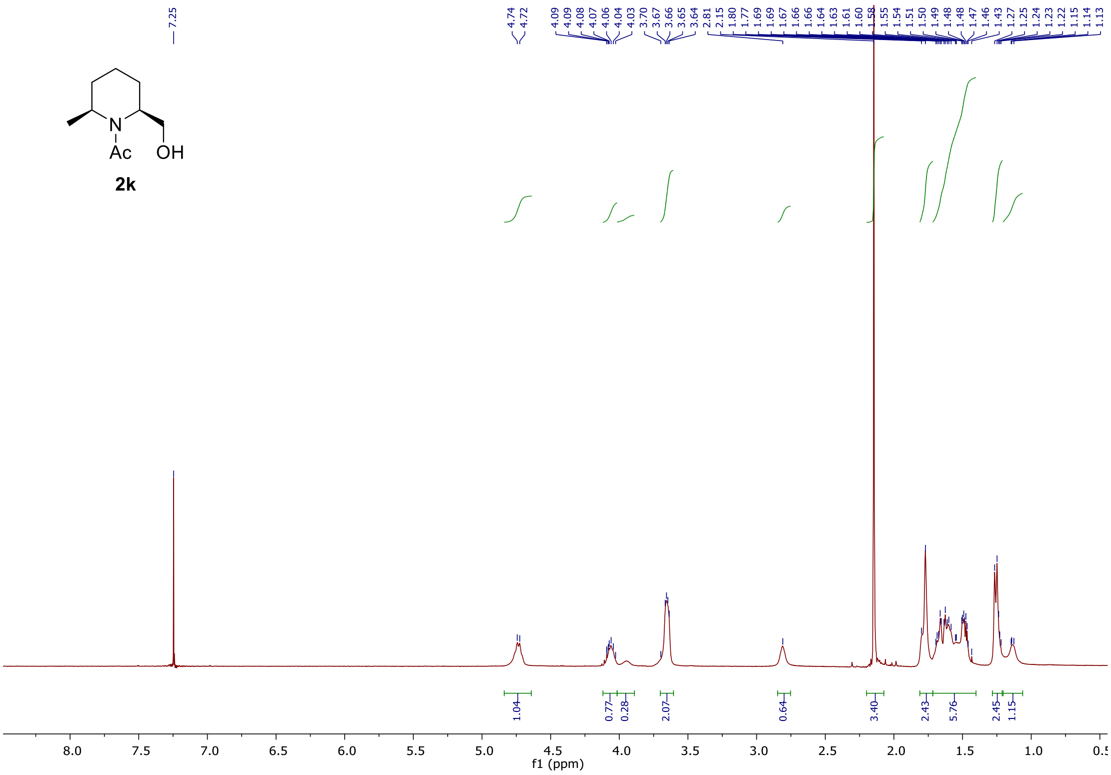

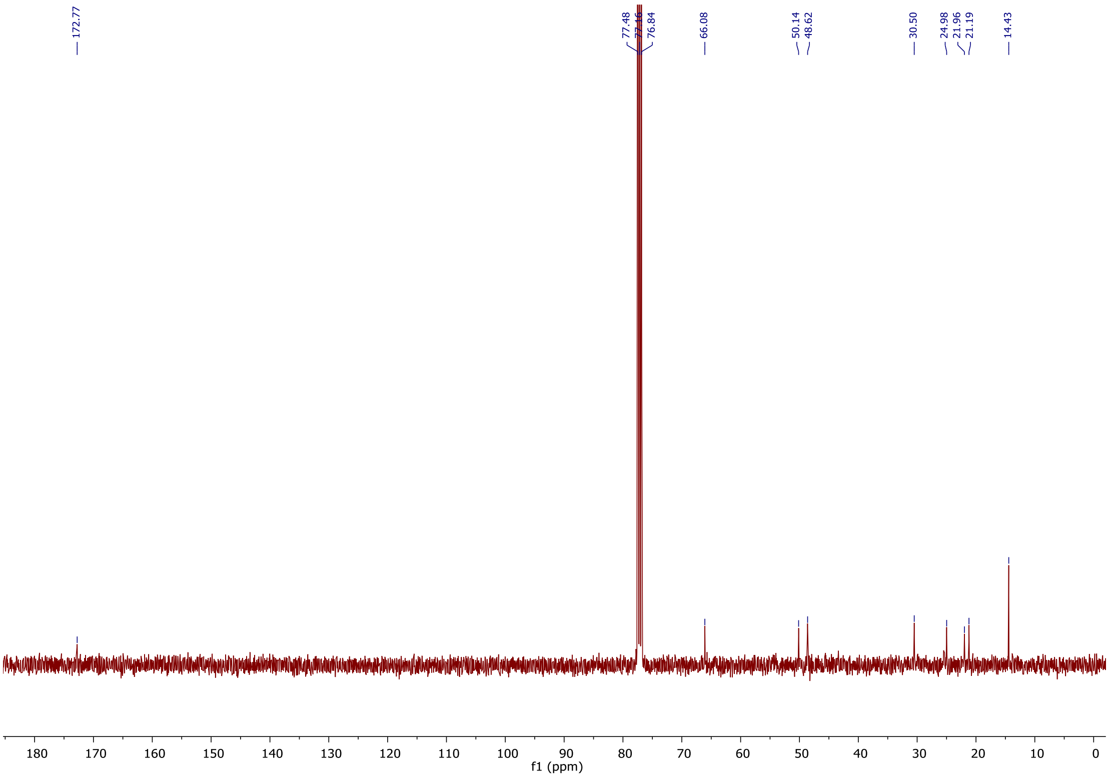

400 MHz ^{1}H NMR spectrum; 100.6 MHz ^{13}C NMR spectrum; $CDCl_3$

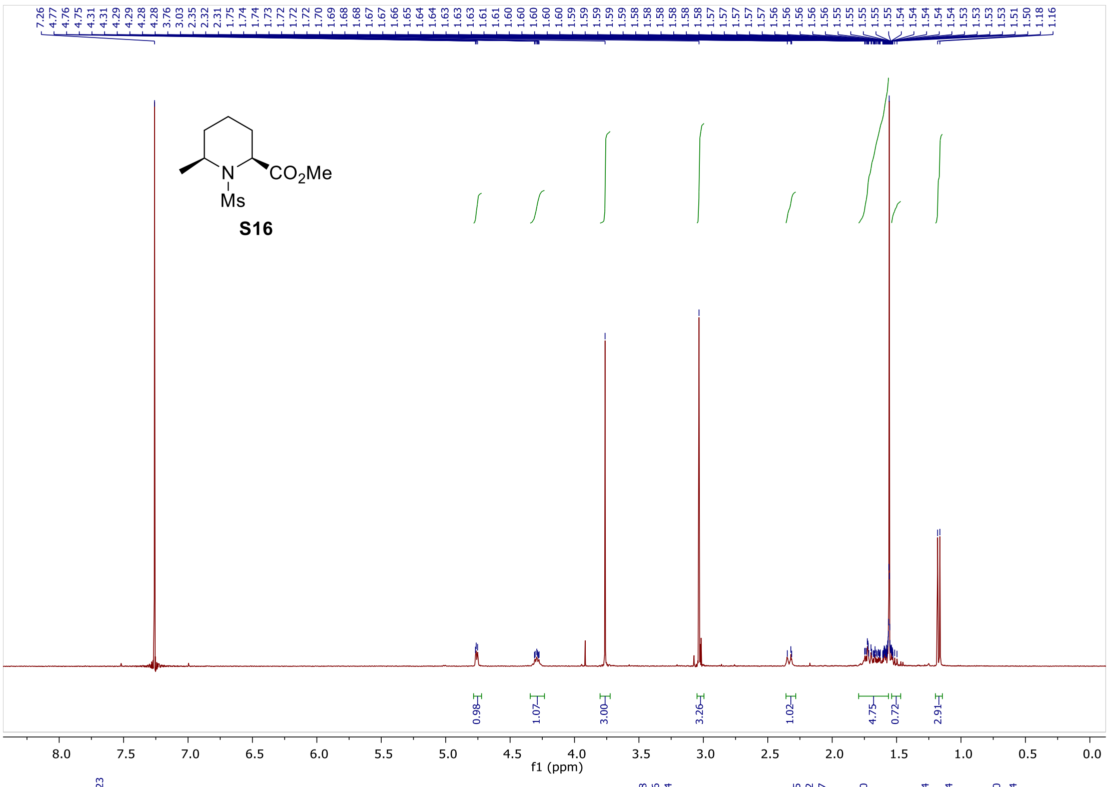

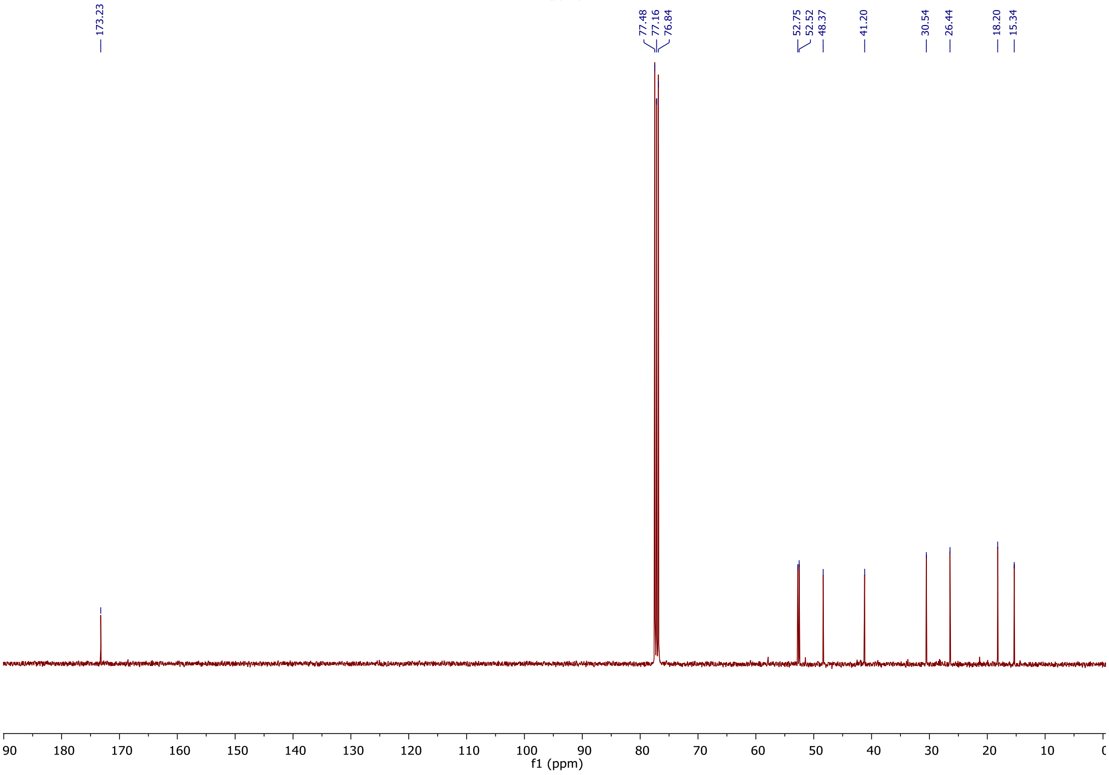

400 MHz ^{1}H NMR spectrum; 100.6 MHz ^{13}C NMR spectrum; $CDCl_3$

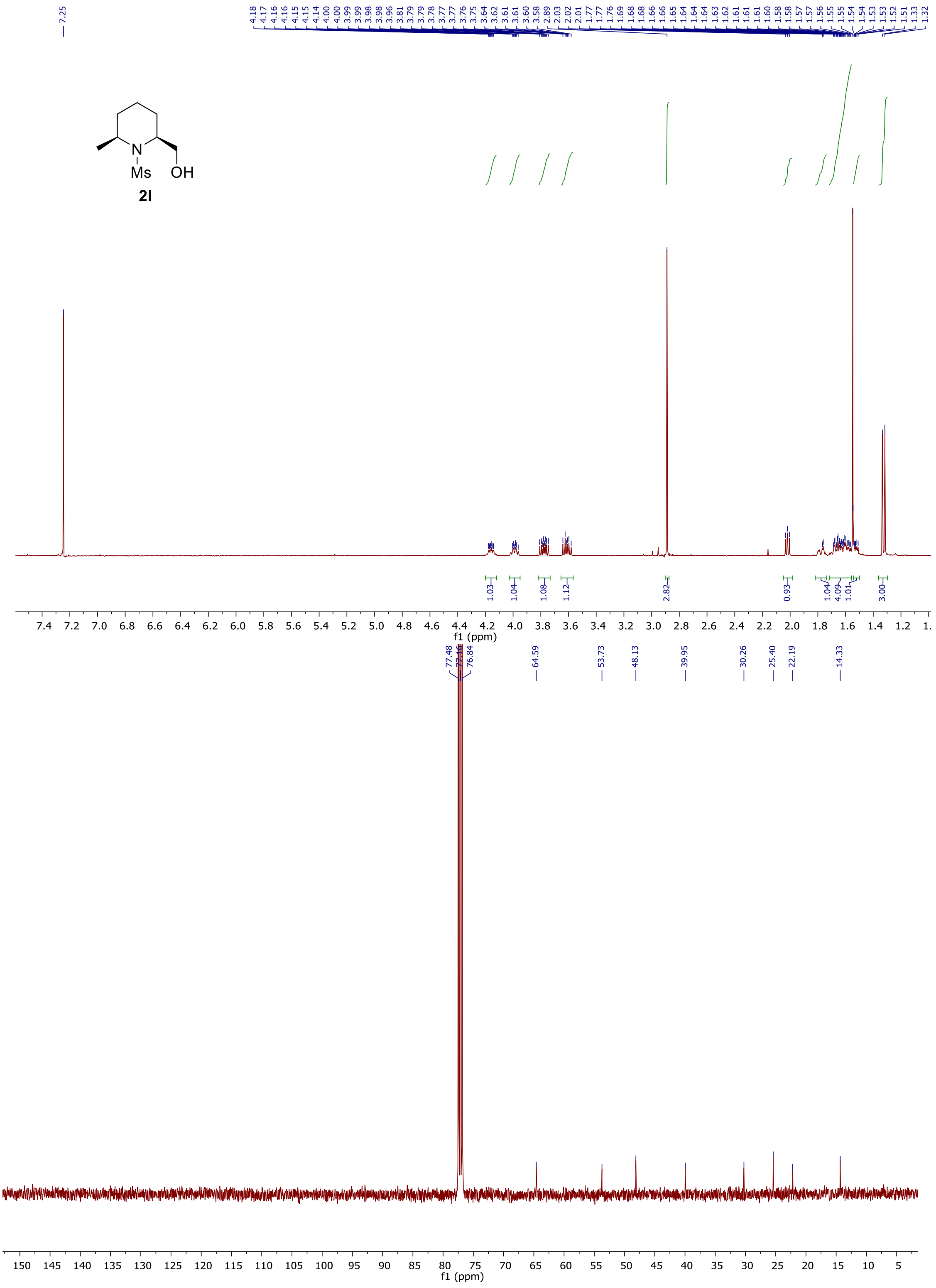

400 MHz ^{1}H NMR spectrum; 100.6 MHz ^{13}C NMR spectrum; $CDCl_3$

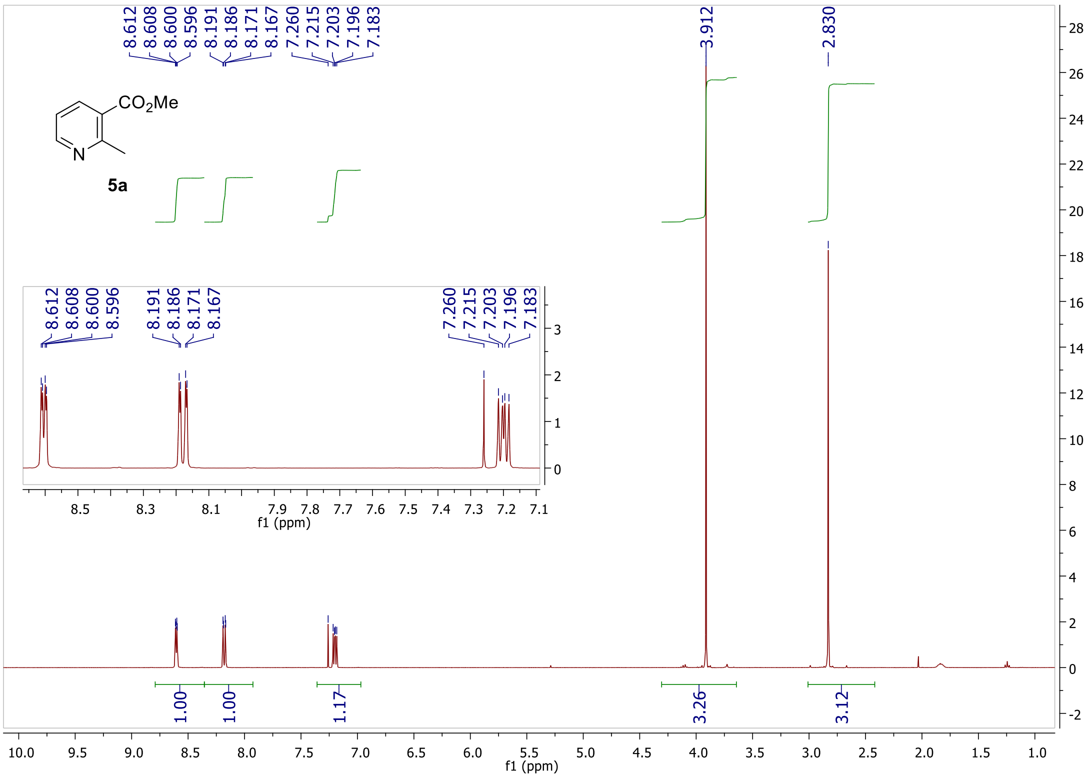

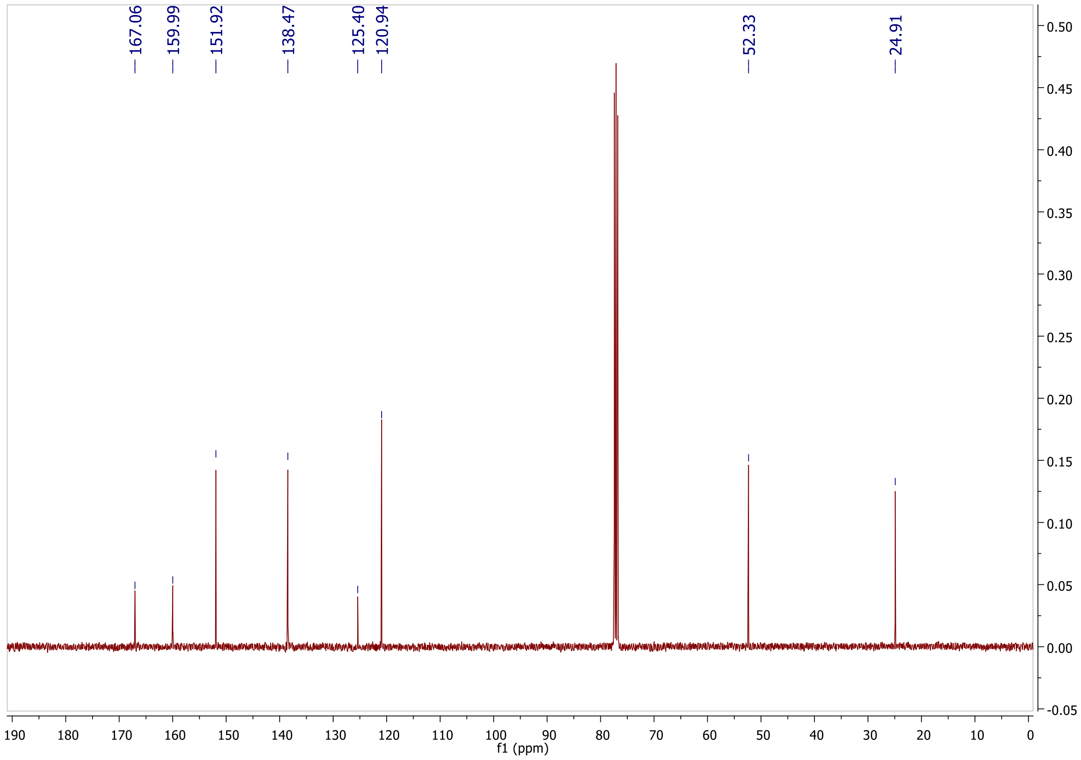

400 MHz ^{1}H NMR spectrum; 100.6 MHz ^{13}C NMR spectrum; $CDCl_3$

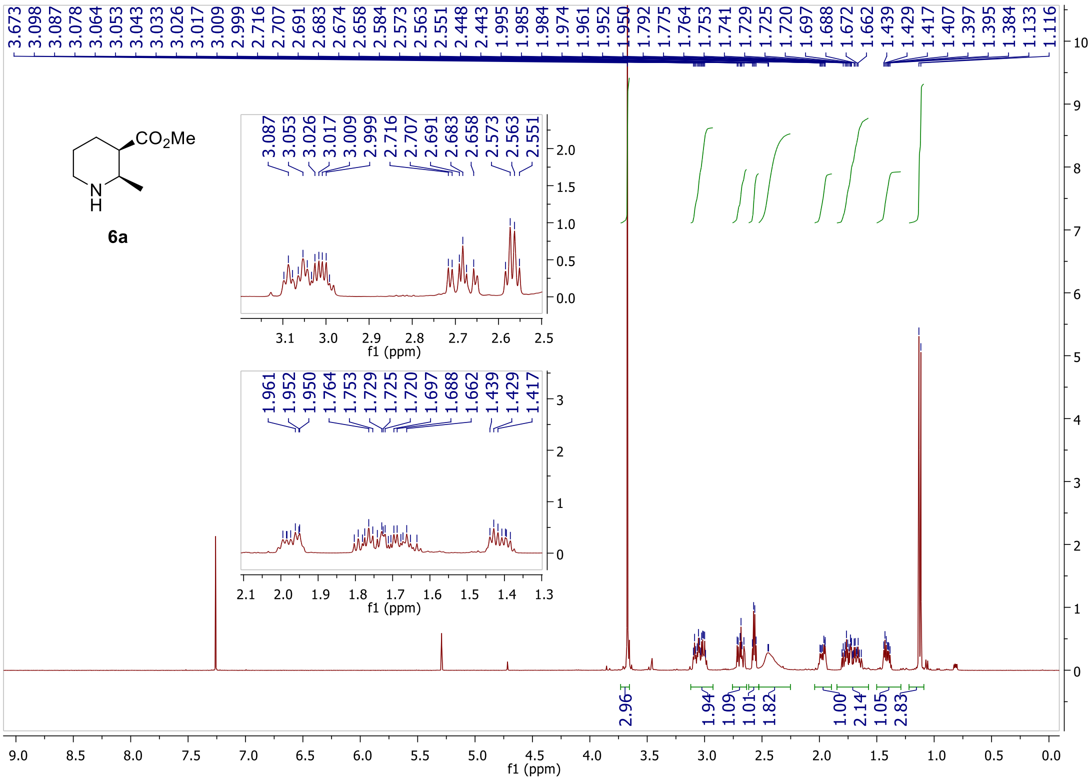

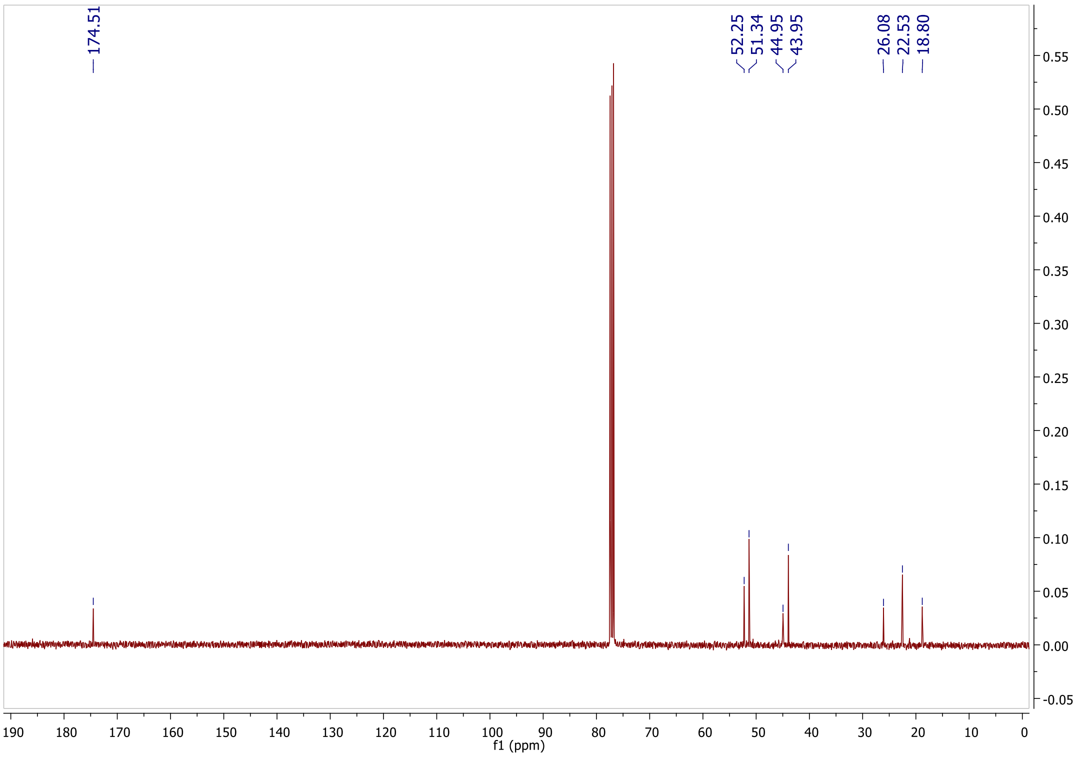

400 MHz ^1H NMR spectrum; 100.6 MHz ^{13}C NMR spectrum; MeOH-*d*4

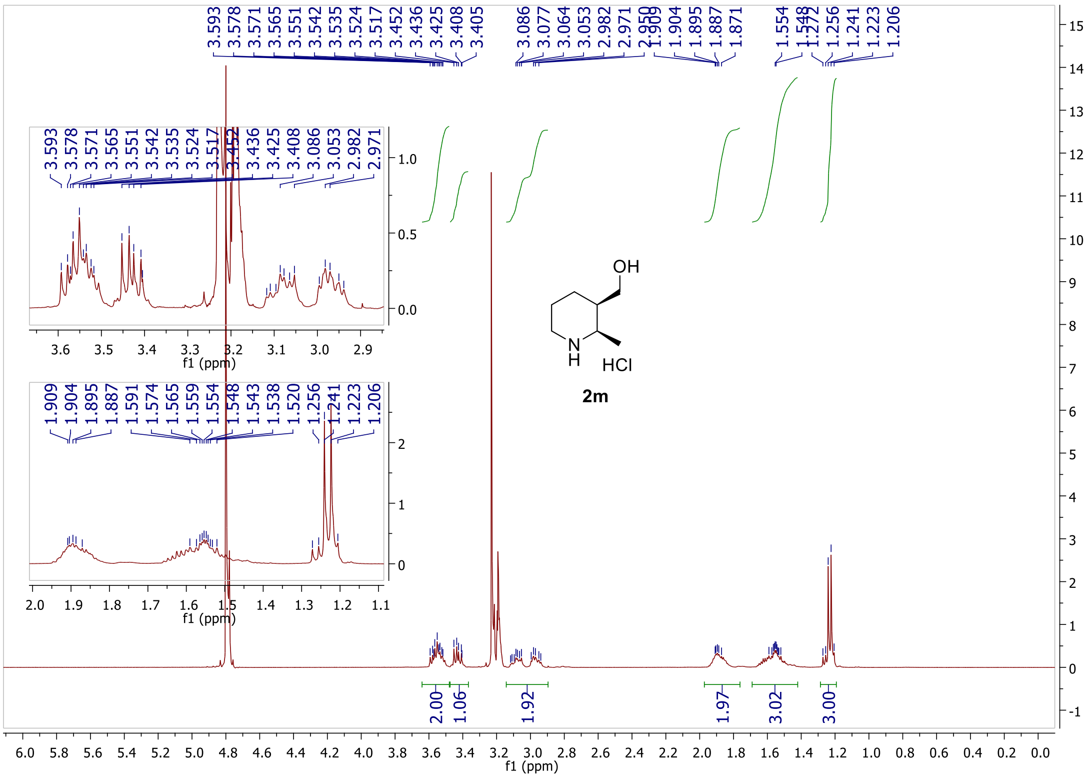

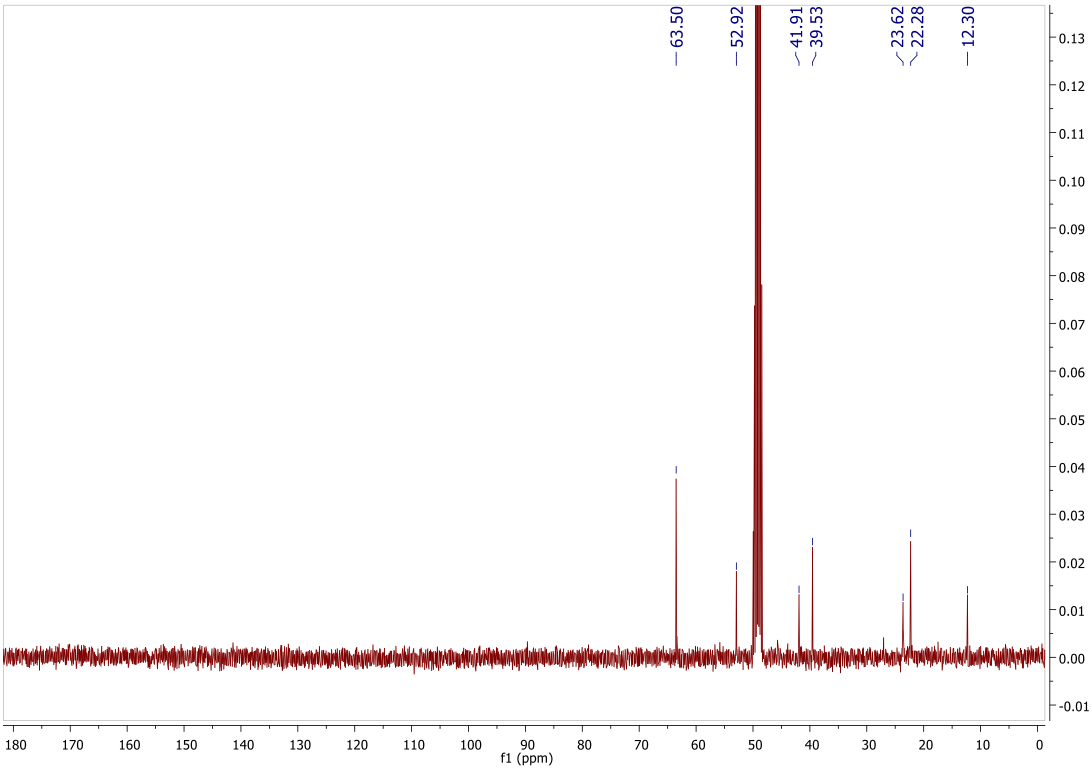

400 MHz ^{1}H NMR spectrum; 100.6 MHz ^{13}C NMR spectrum; $CDCl_3$

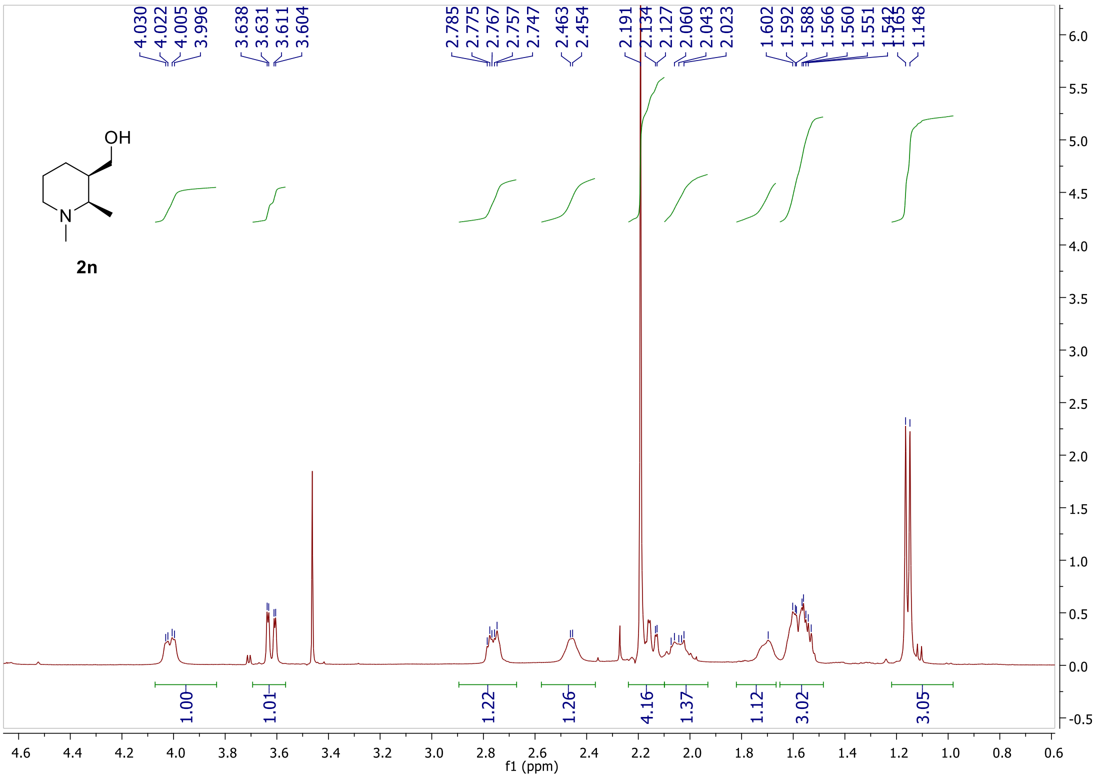

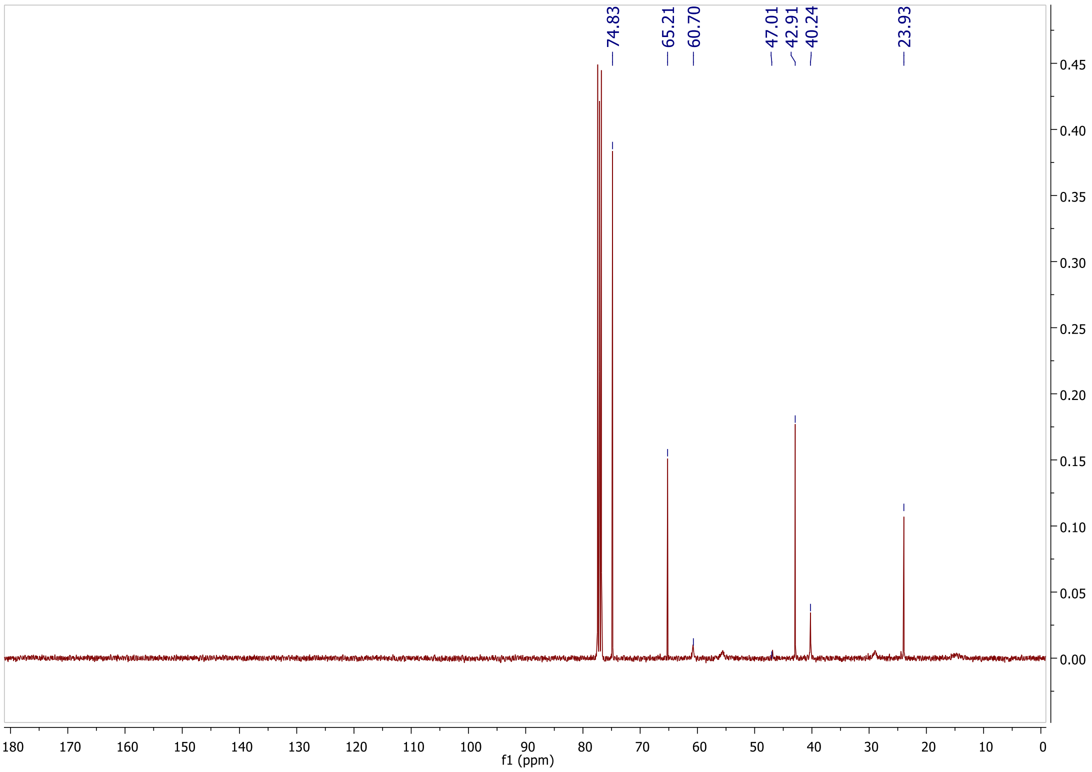

400 MHz ^{1}H NMR spectrum; 100.6 MHz ^{13}C NMR spectrum; $CDCl_3$

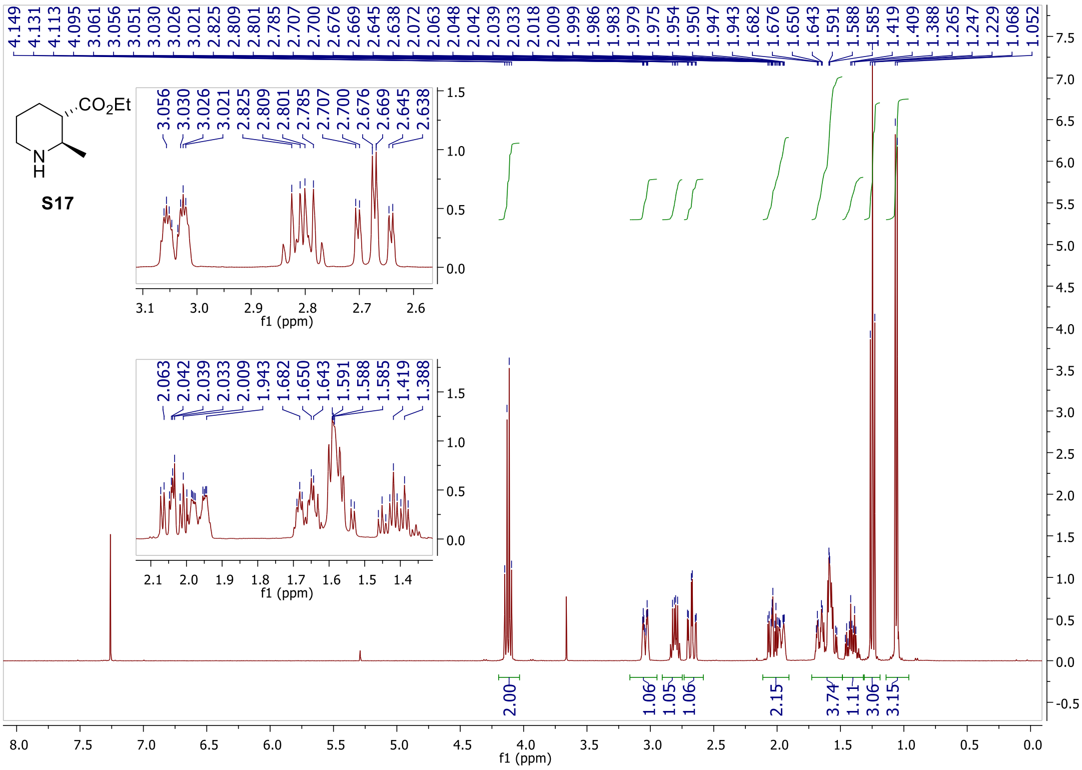

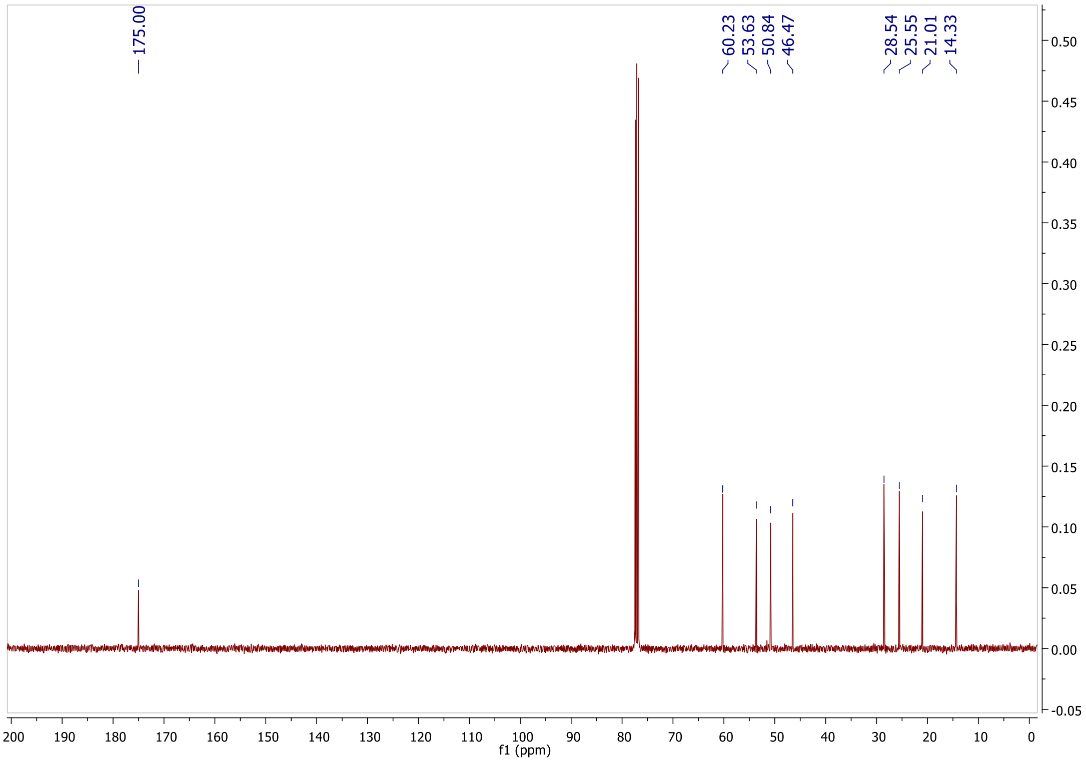

400 MHz ^{1}H NMR spectrum; 100.6 MHz ^{13}C NMR spectrum; $CDCl_3$

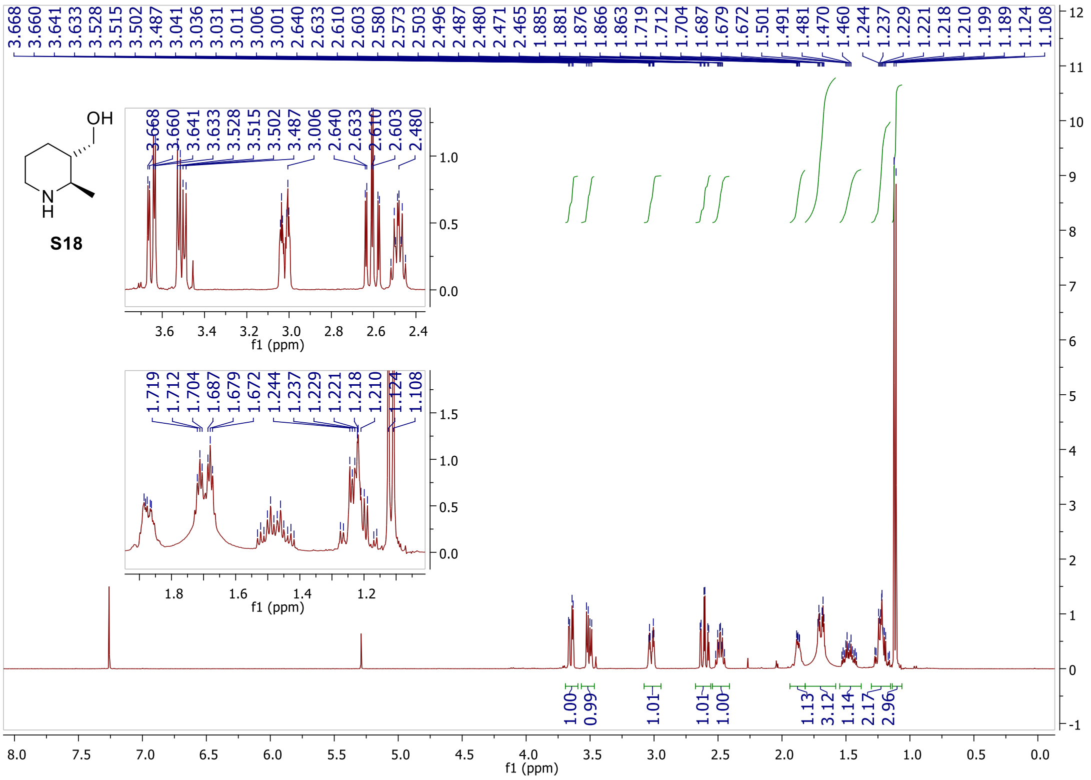

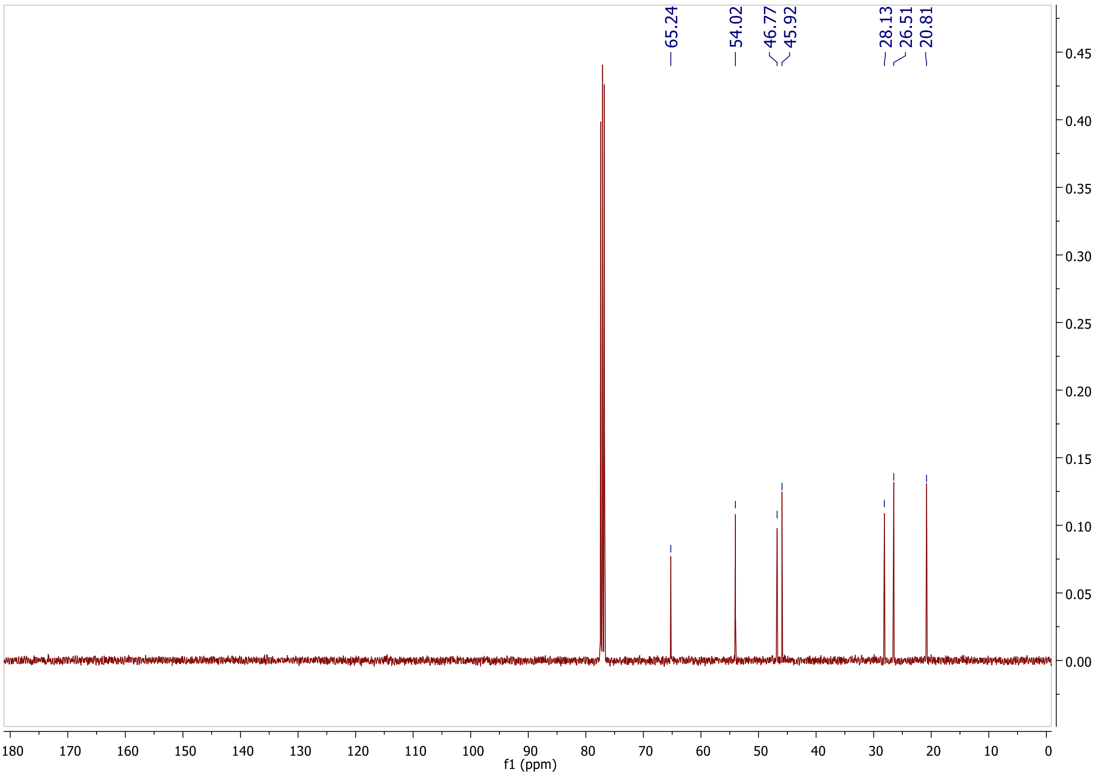

400 MHz ^{1}H NMR spectrum; 100.6 MHz ^{13}C NMR spectrum; $CDCl_3$

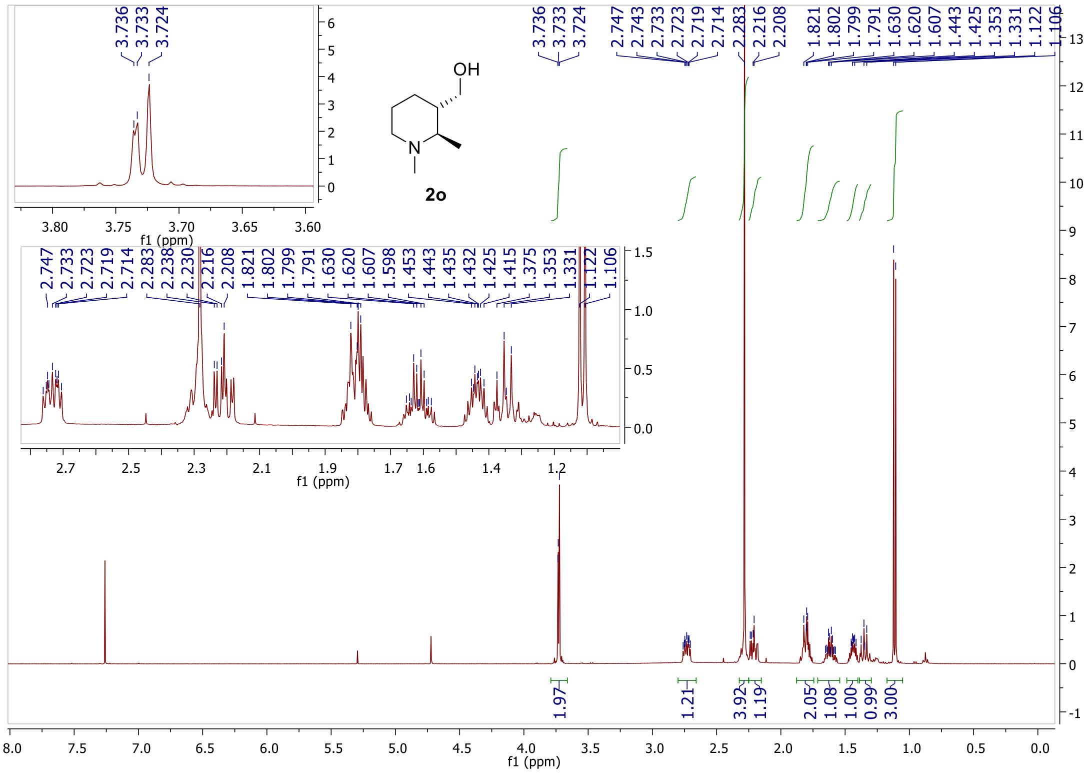

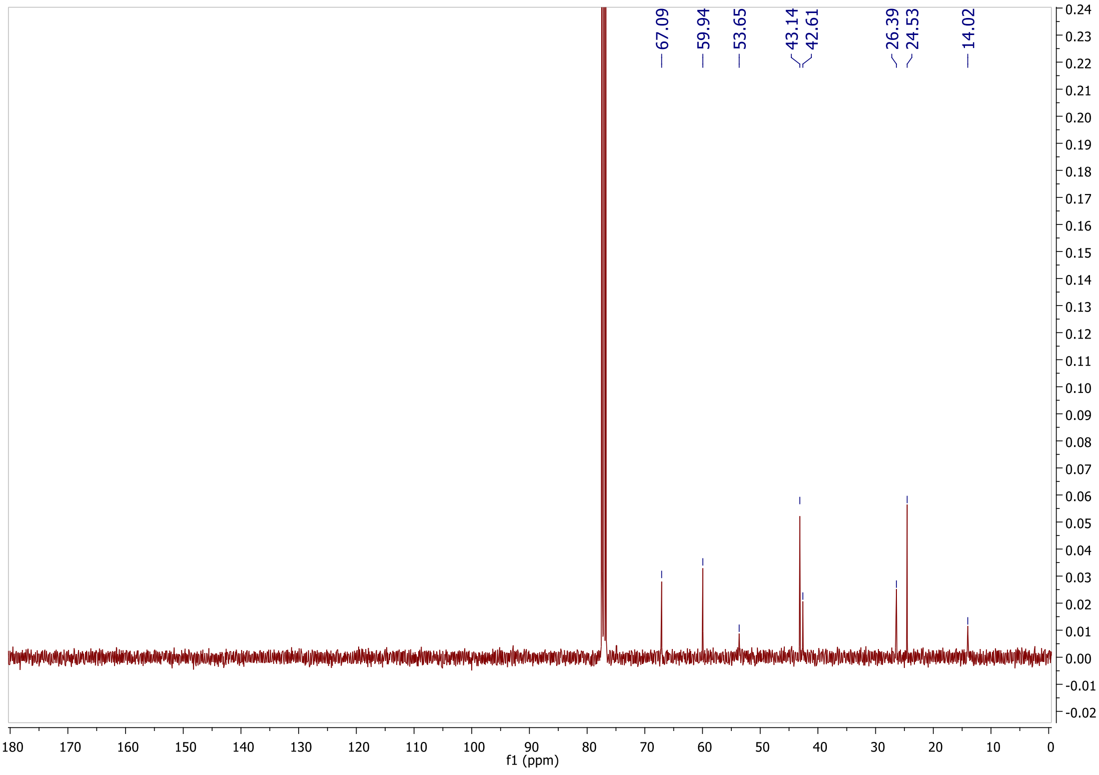

400 MHz ^{1}H NMR spectrum; 100.6 MHz ^{13}C NMR spectrum; $CDCl_3$

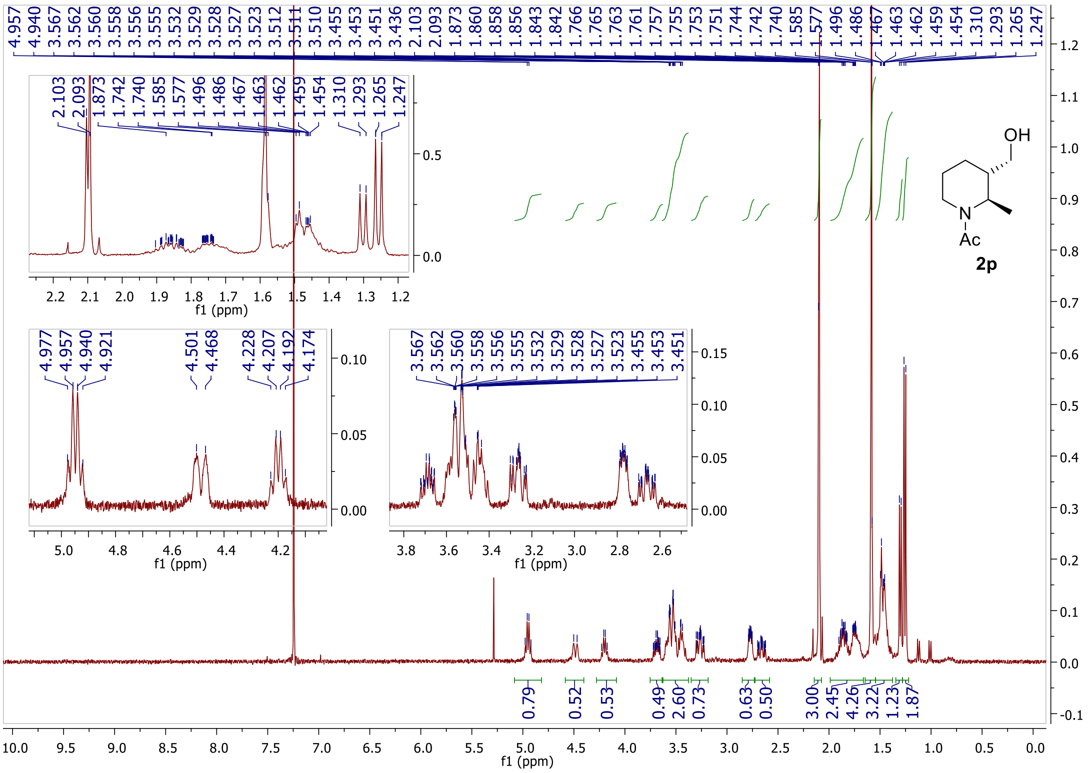

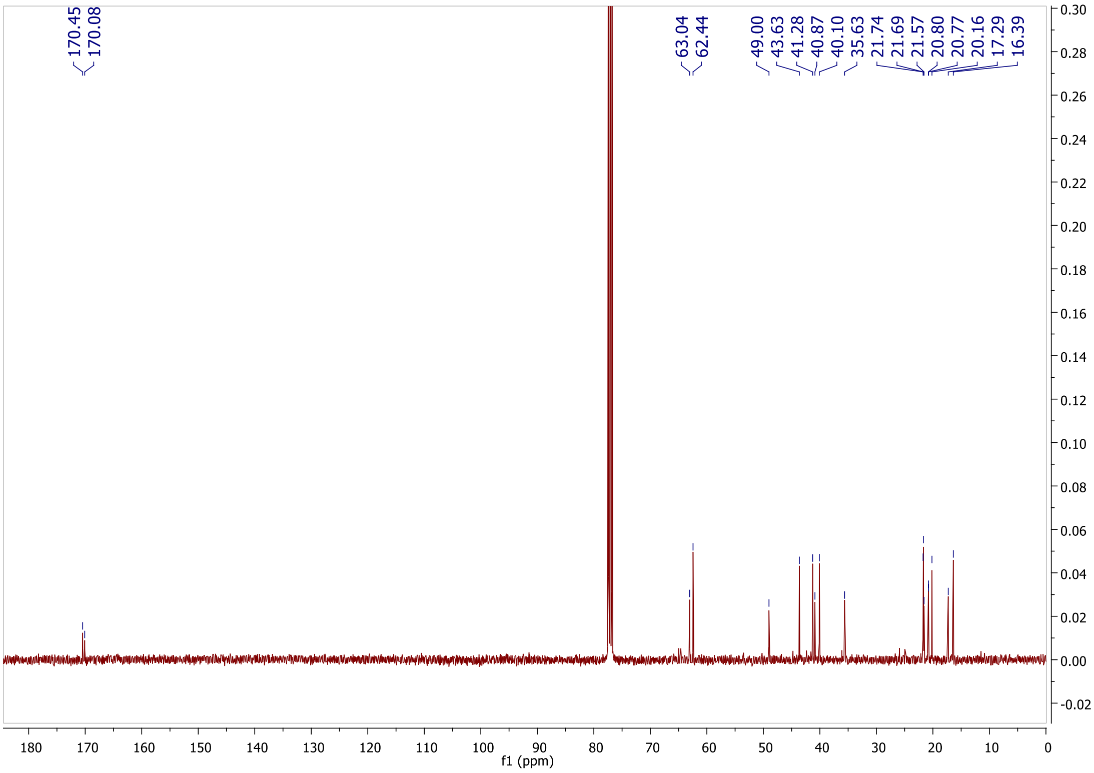

400 MHz ^{1}H NMR spectrum; 100.6 MHz ^{13}C NMR spectrum; $CDCl_3$

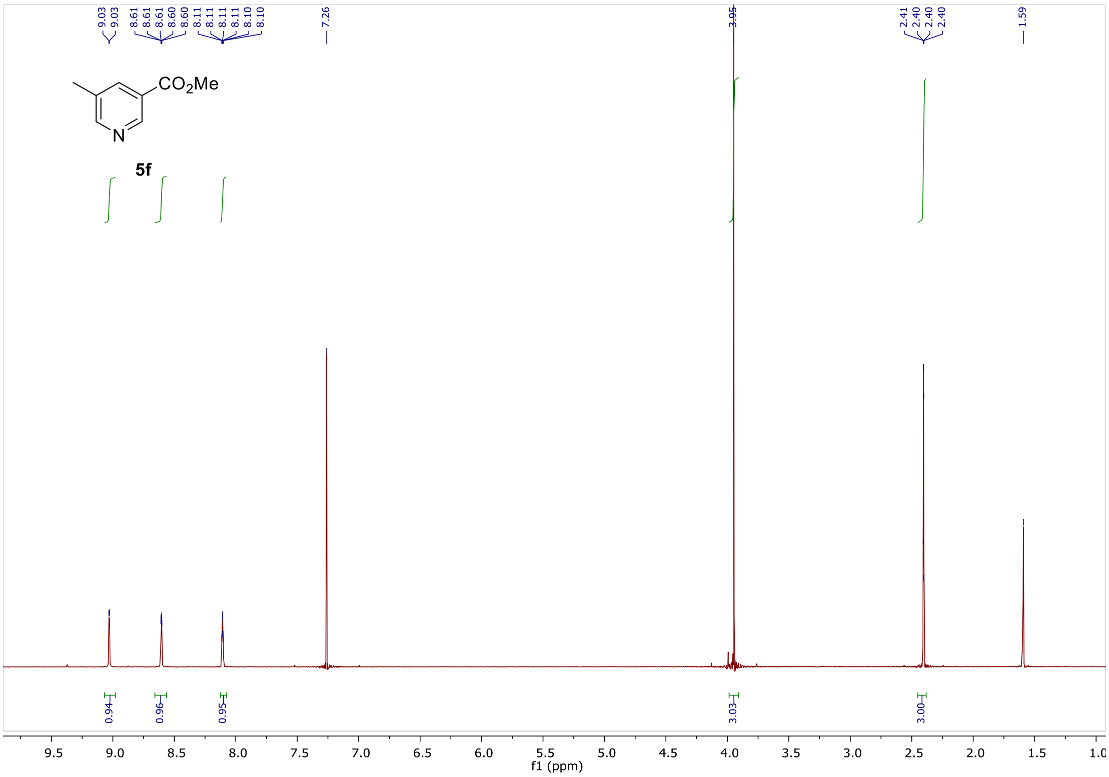

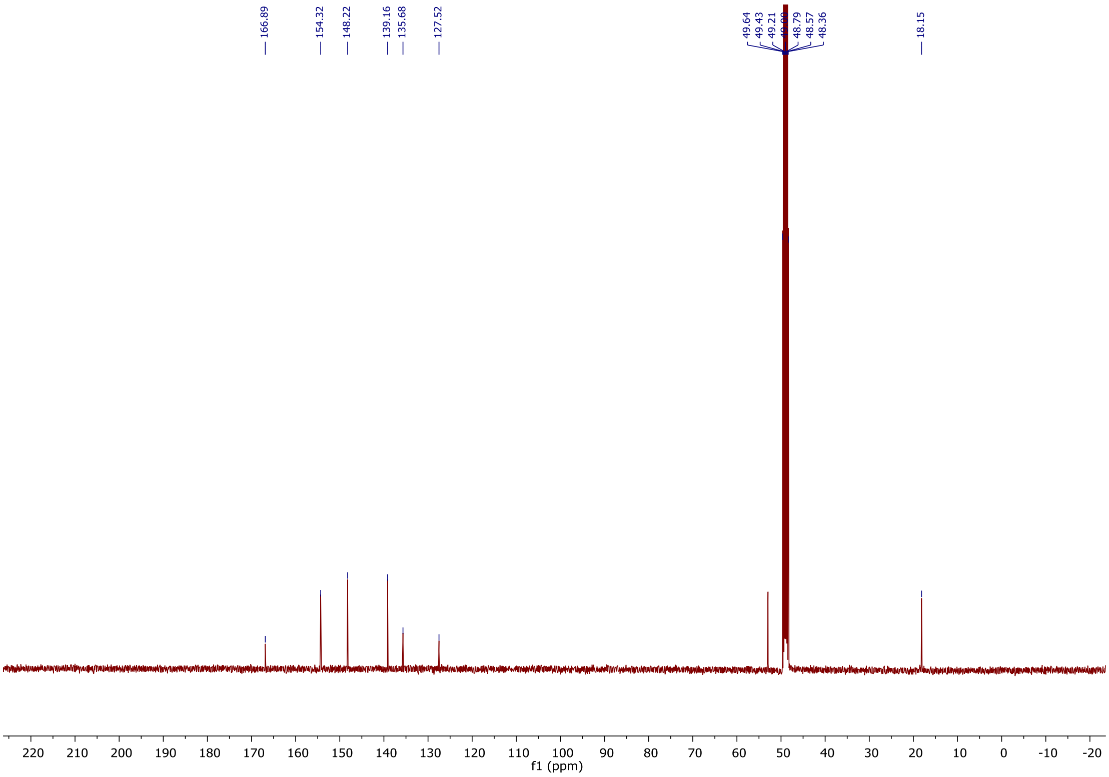

400 MHz ^{1}H NMR spectrum; 100.6 MHz ^{13}C NMR spectrum; $CDCl_3$

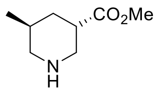

S19

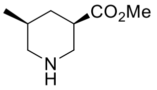

6f

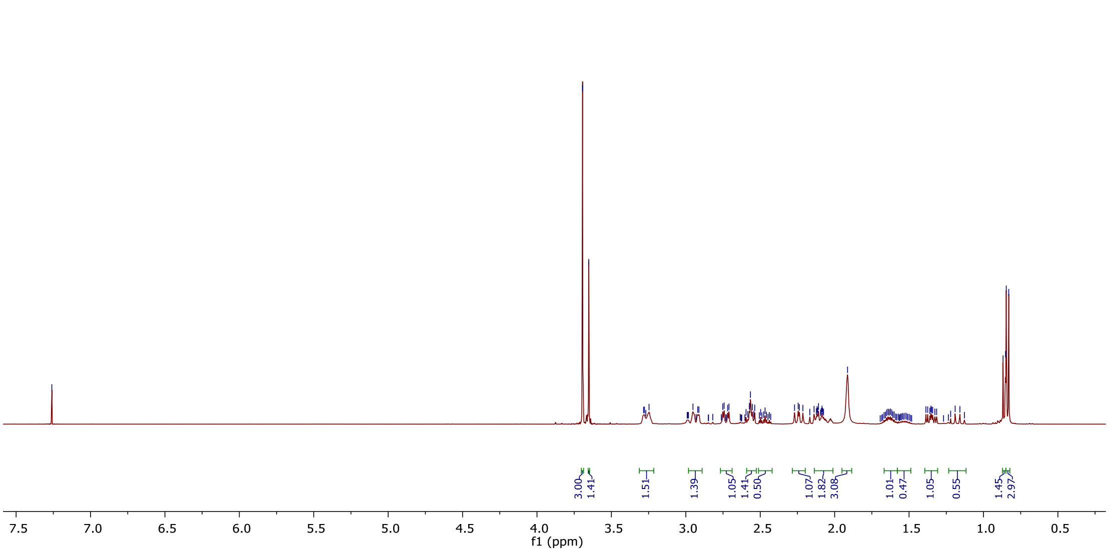

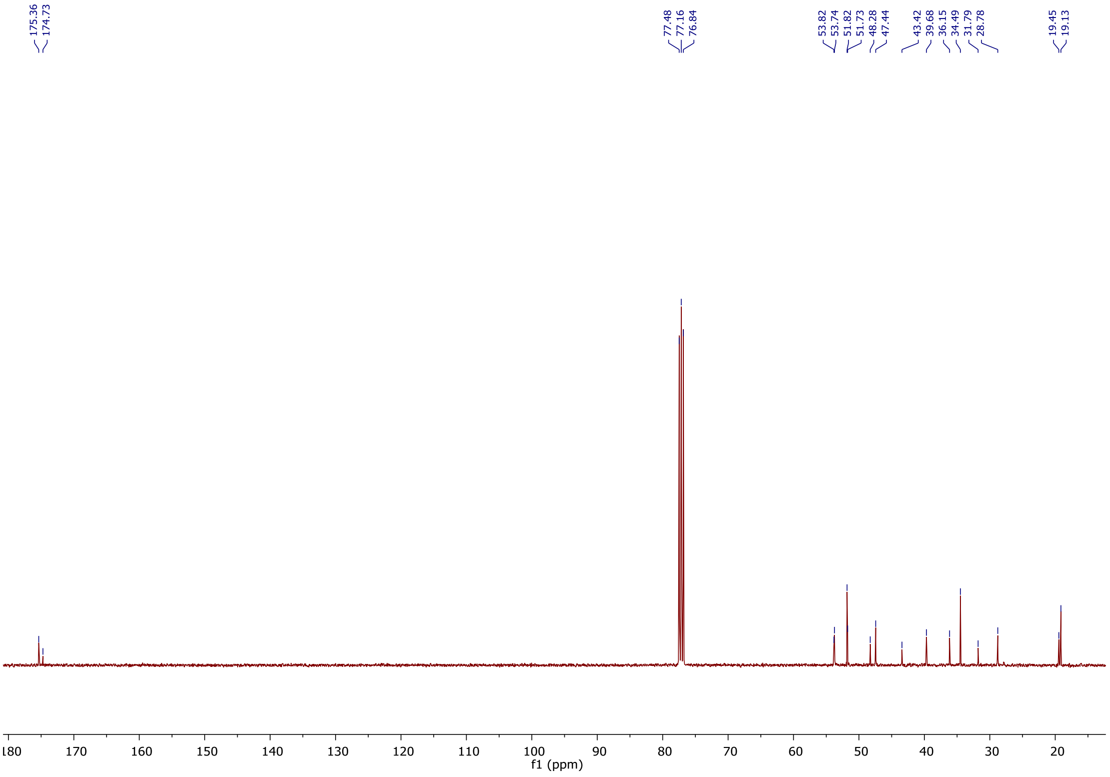

400 MHz ^{1}H NMR spectrum; 100.6 MHz ^{13}C NMR spectrum; $CDCl_3$

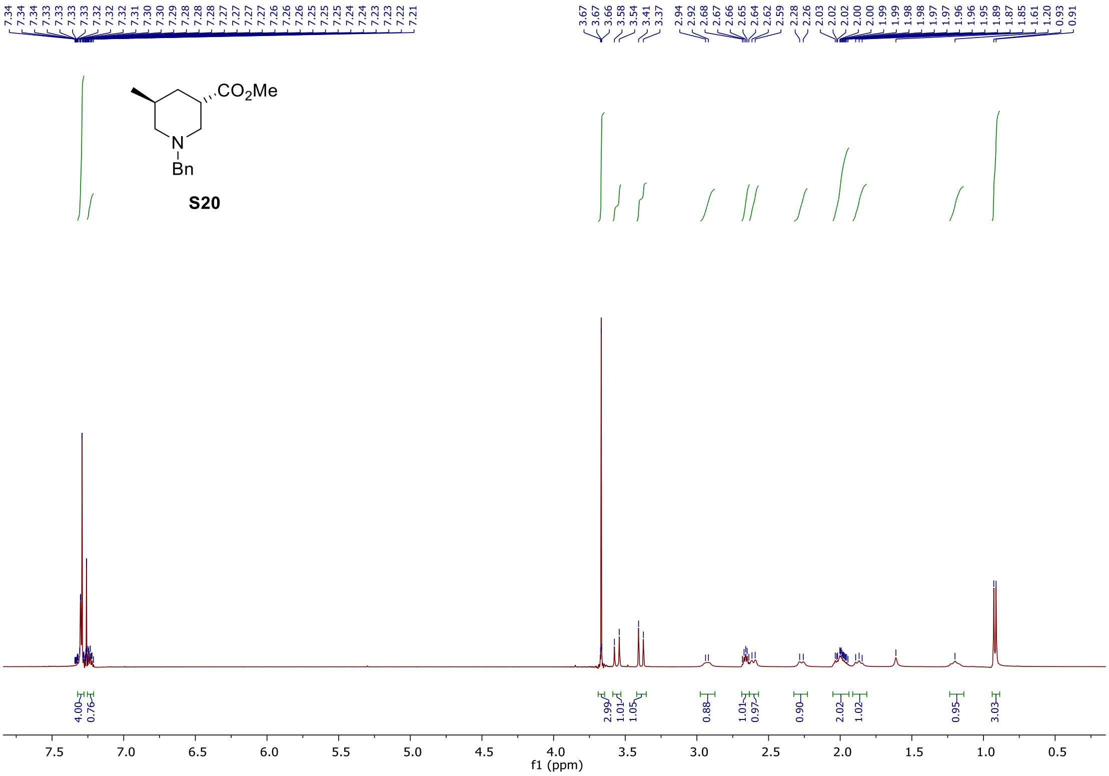

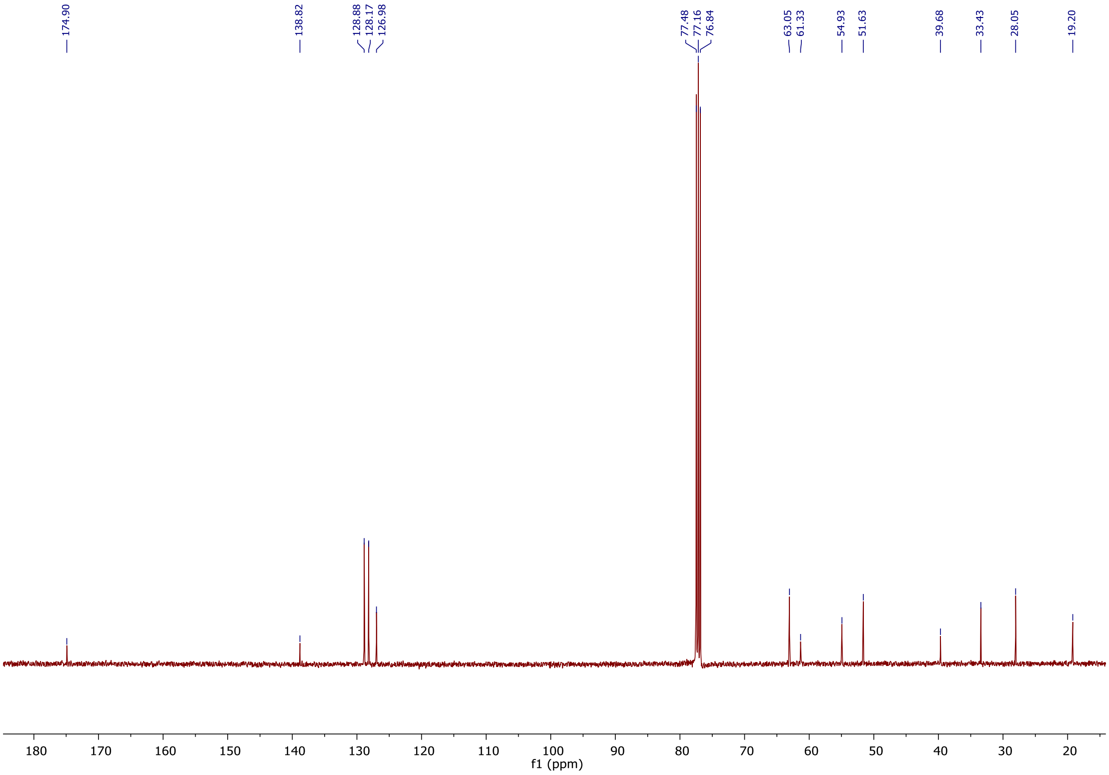

400 MHz ^{1}H NMR spectrum; 100.6 MHz ^{13}C NMR spectrum; $CDCl_3$

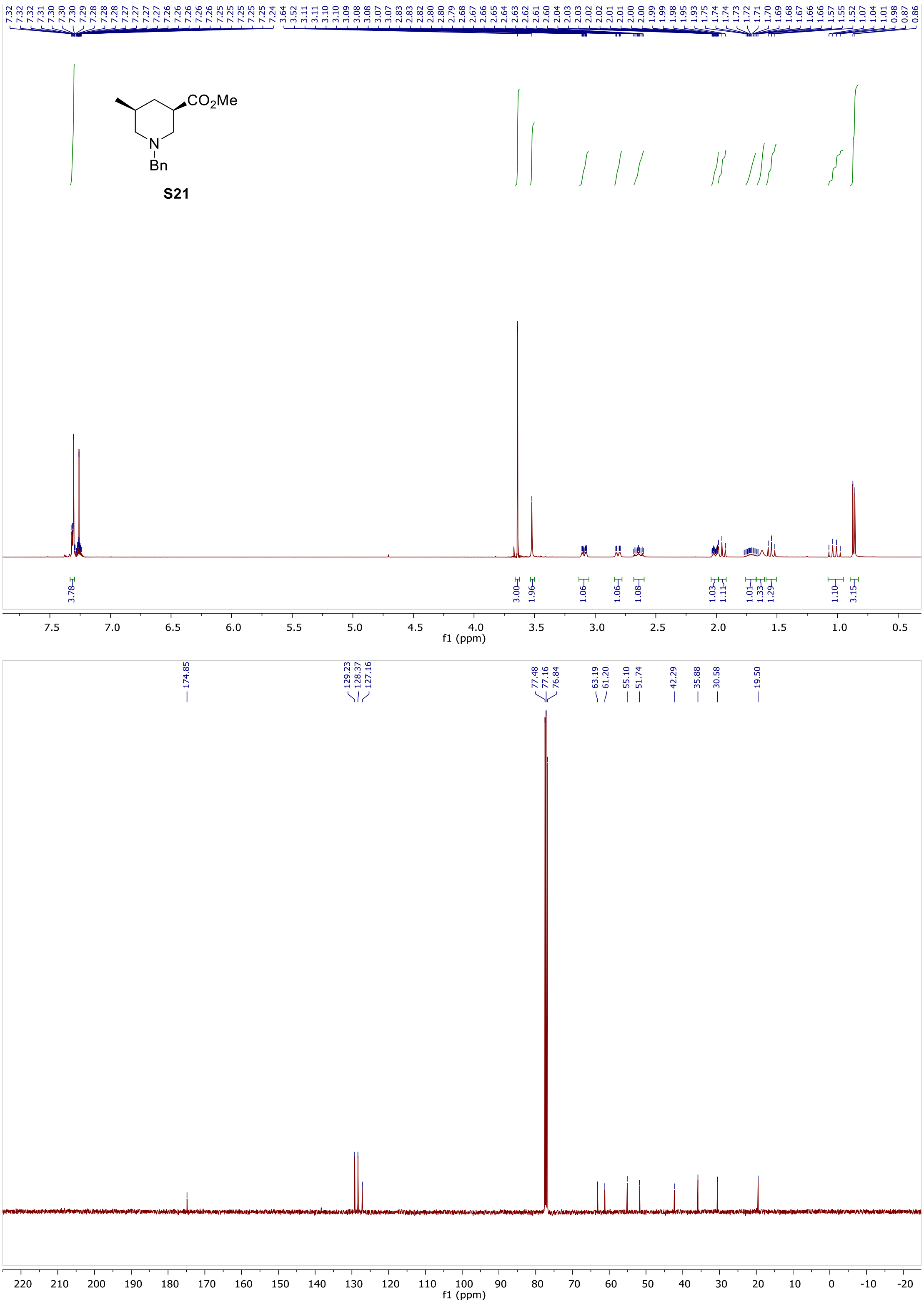

400 MHz ^{1}H NMR spectrum; 100.6 MHz ^{13}C NMR spectrum; $CDCl_3$

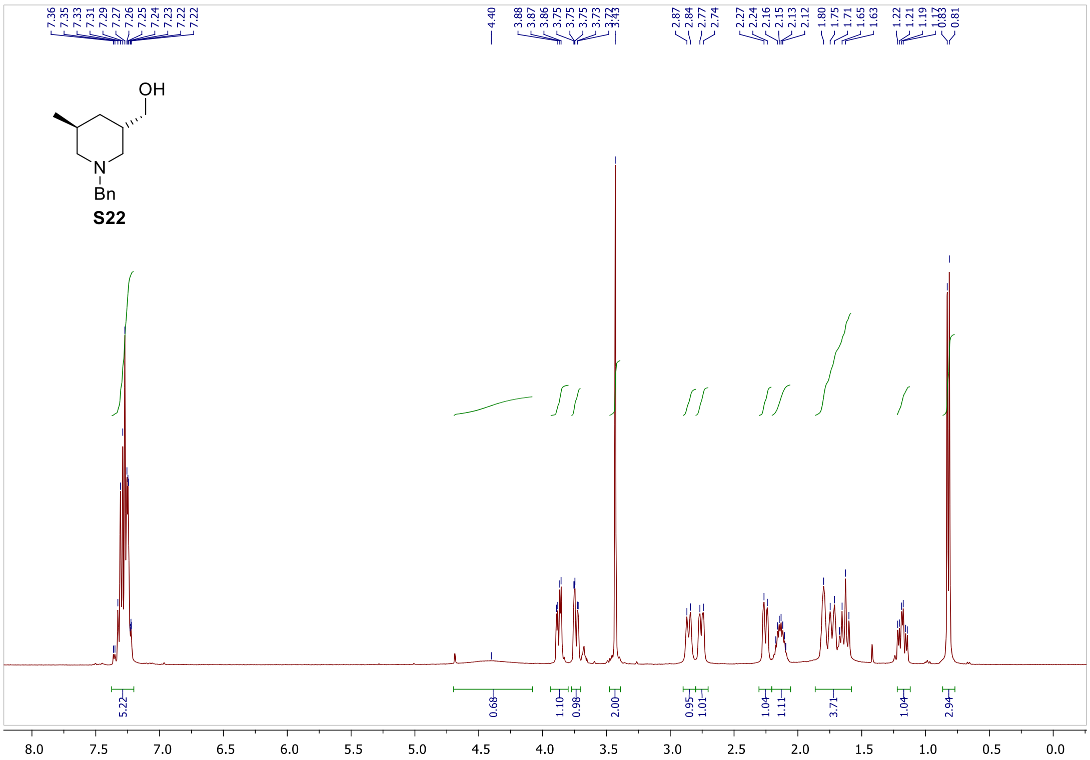

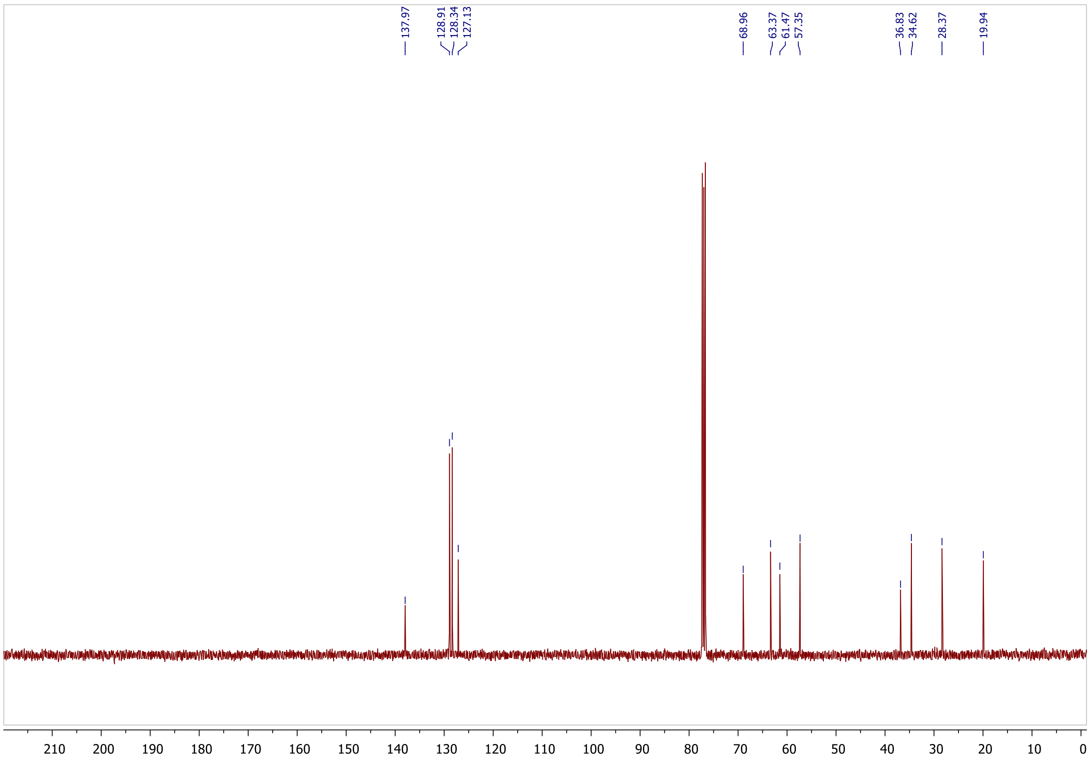

400 MHz ^{1}H NMR spectrum; 100.6 MHz ^{13}C NMR spectrum; $CDCl_3$

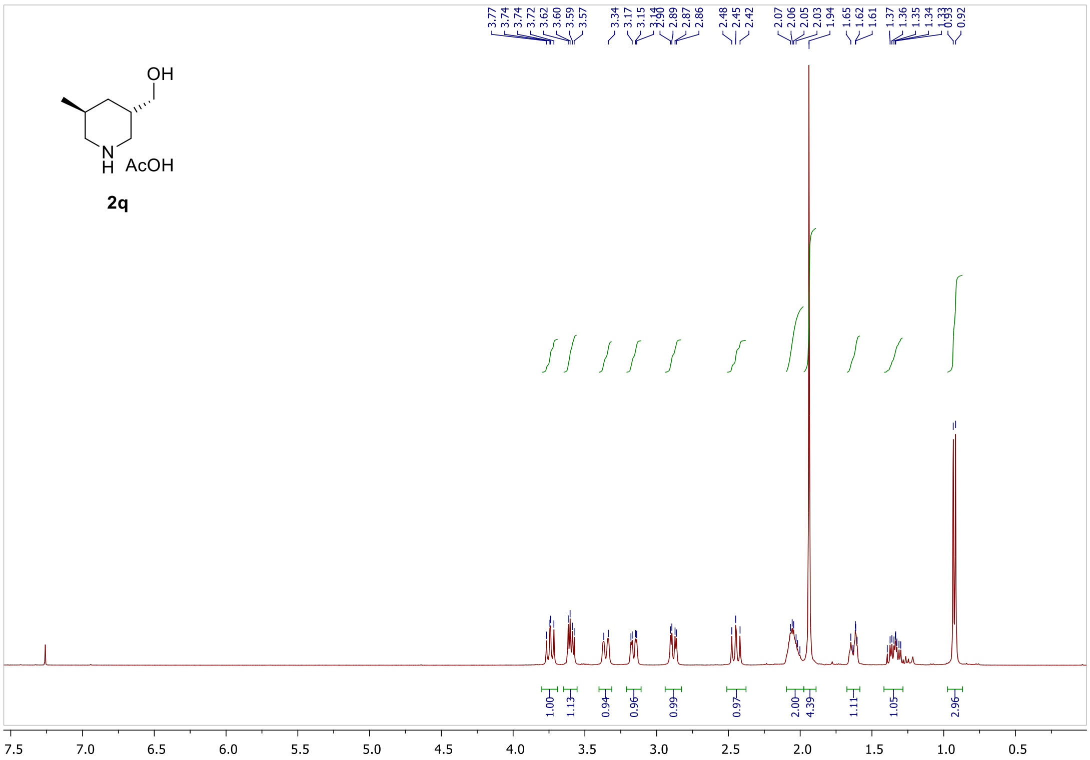

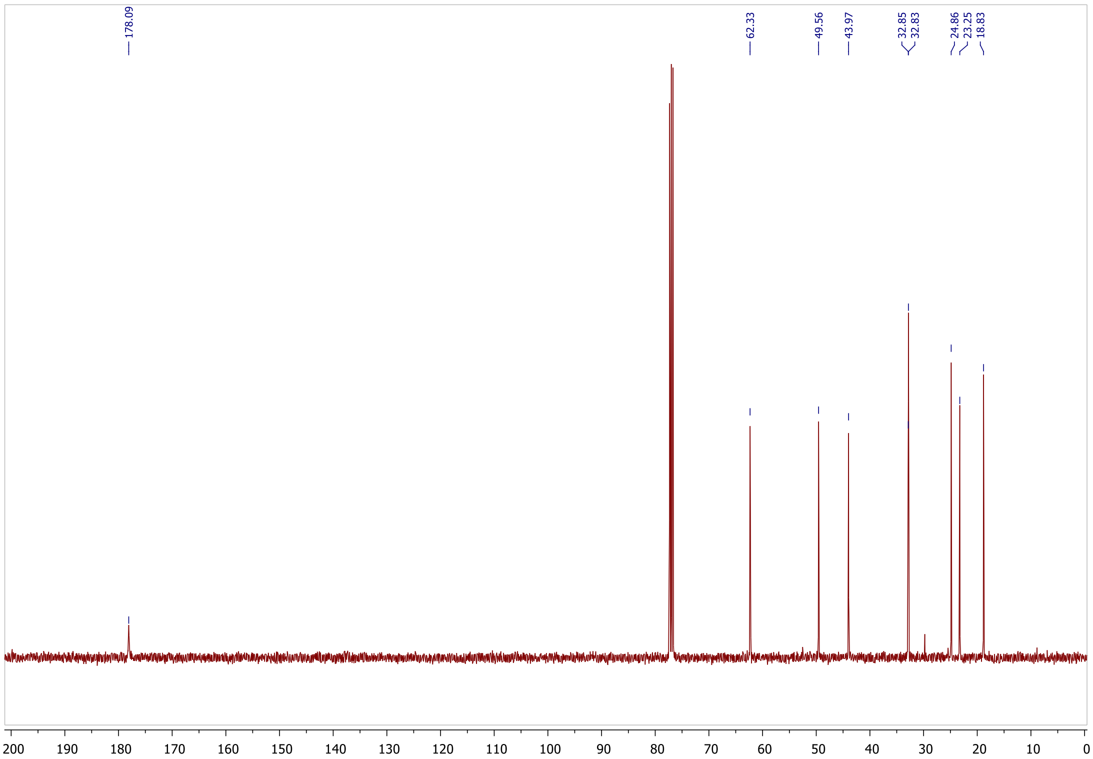

400 MHz ^1H NMR spectrum; $CDCl_3$

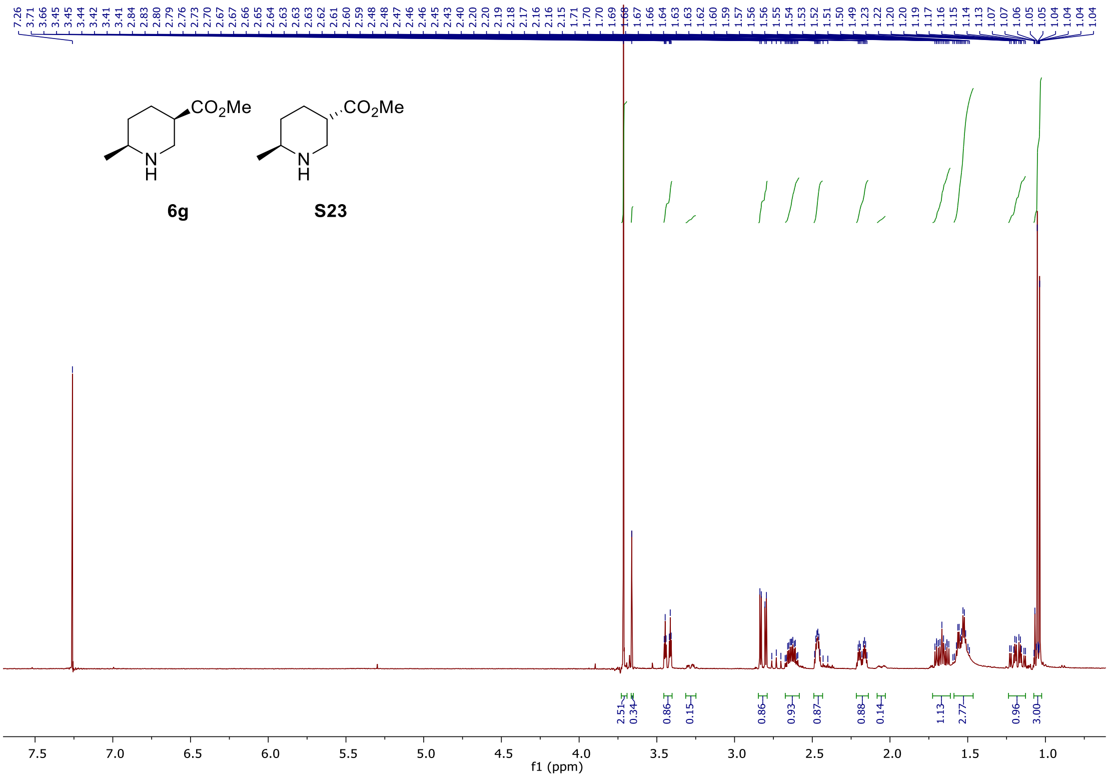

400 MHz ^1H NMR spectrum; 100.6 MHz ^{13}C NMR spectrum; $CDCl_3$

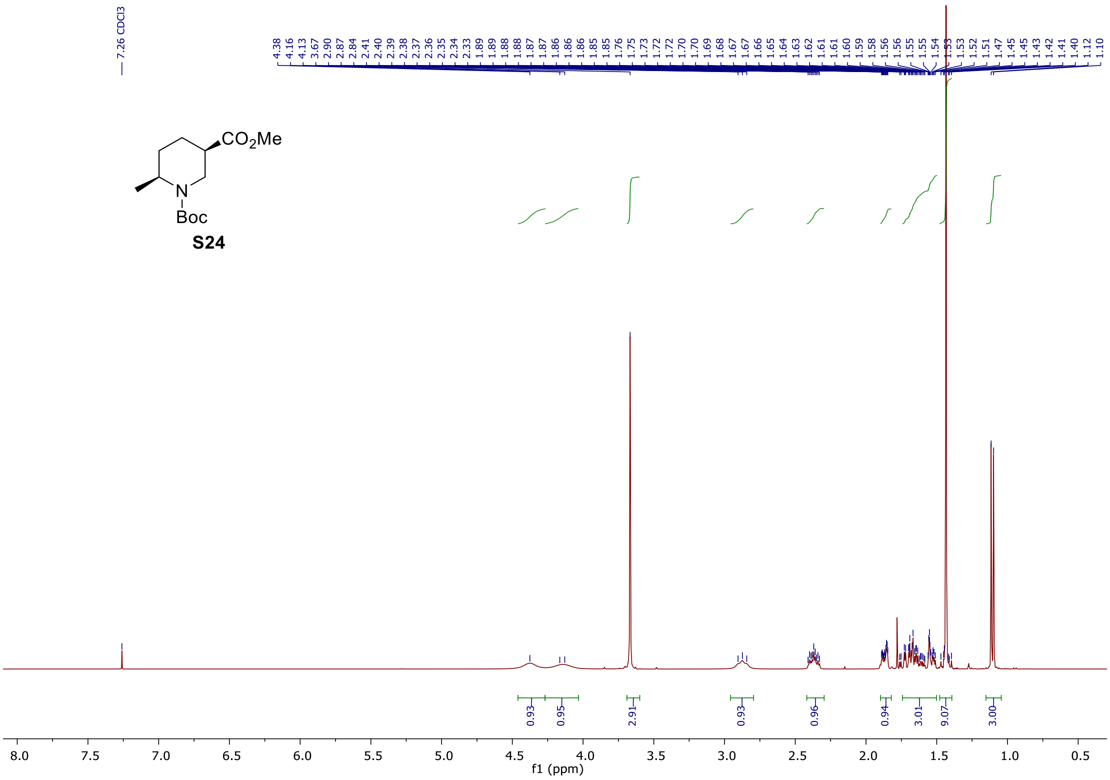

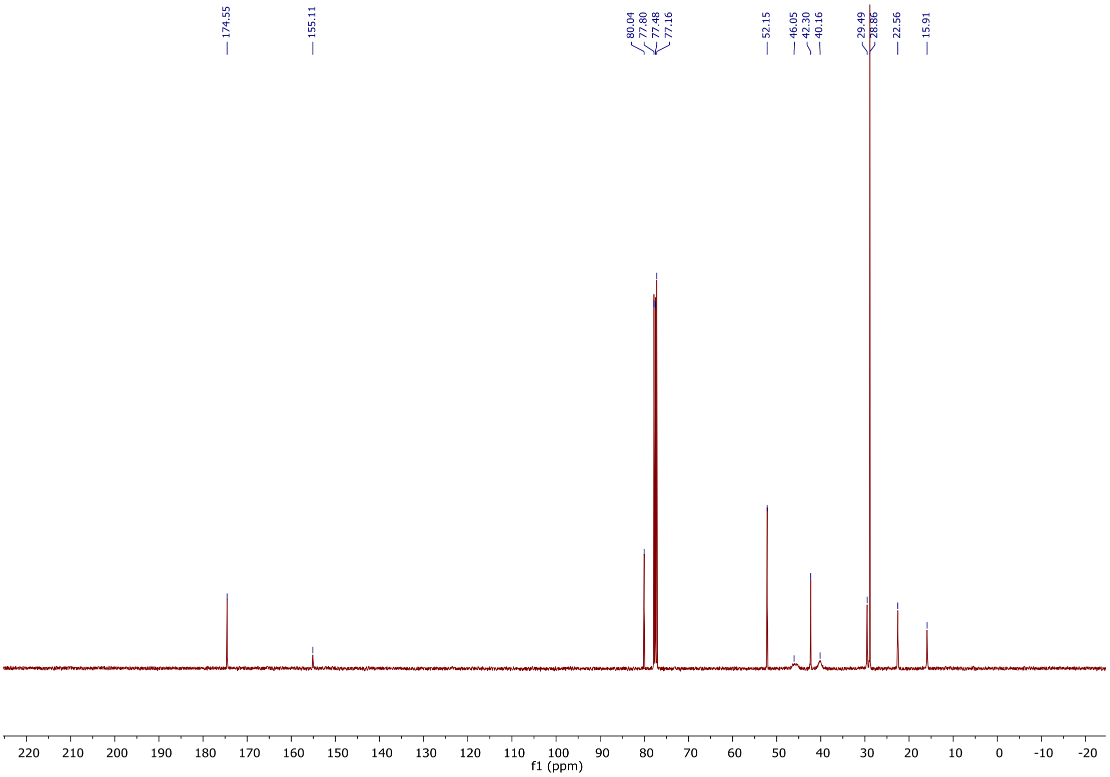

400 MHz ^{1}H NMR spectrum; 100.6 MHz ^{13}C NMR spectrum; $CDCl_3$

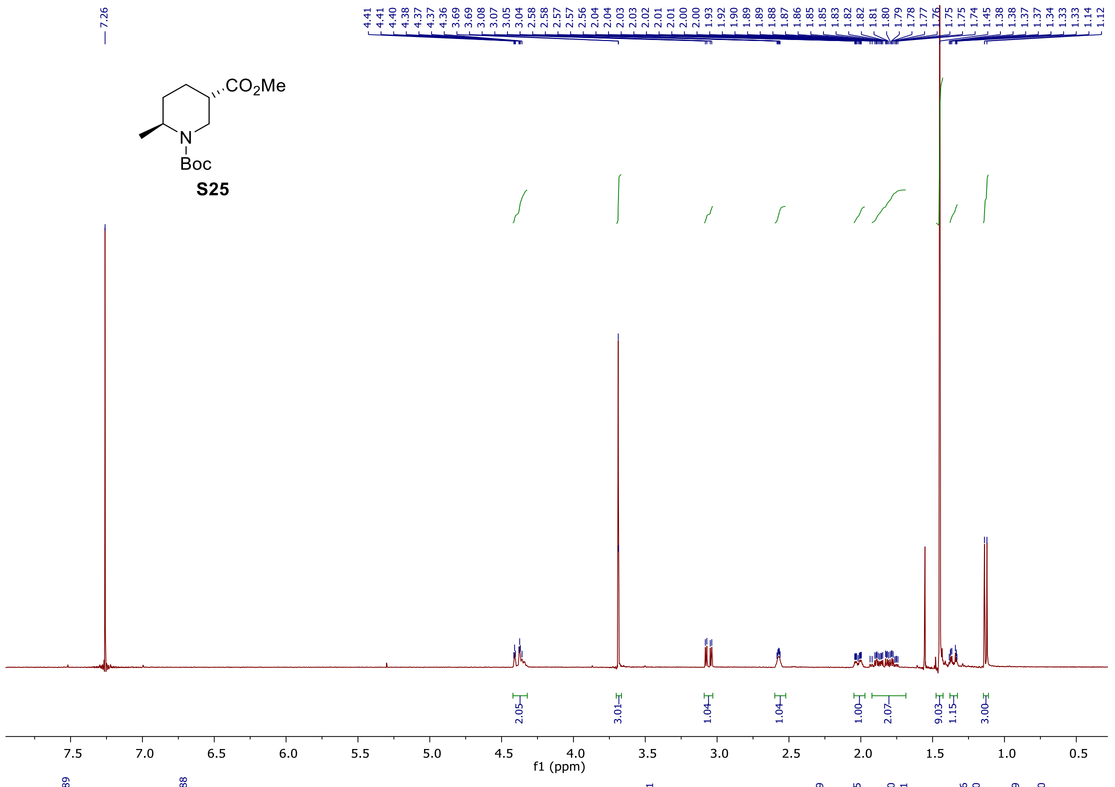

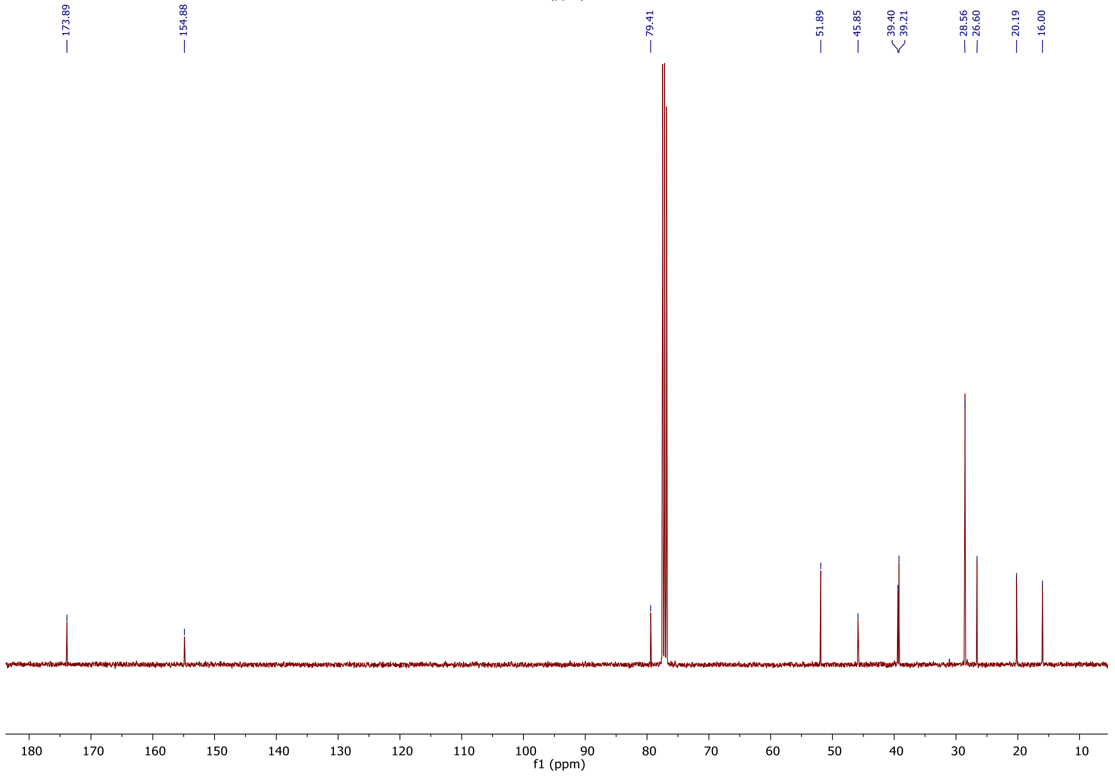

400 MHz ^{1}H NMR spectrum; 100.6 MHz ^{13}C NMR spectrum; $CDCl_3$

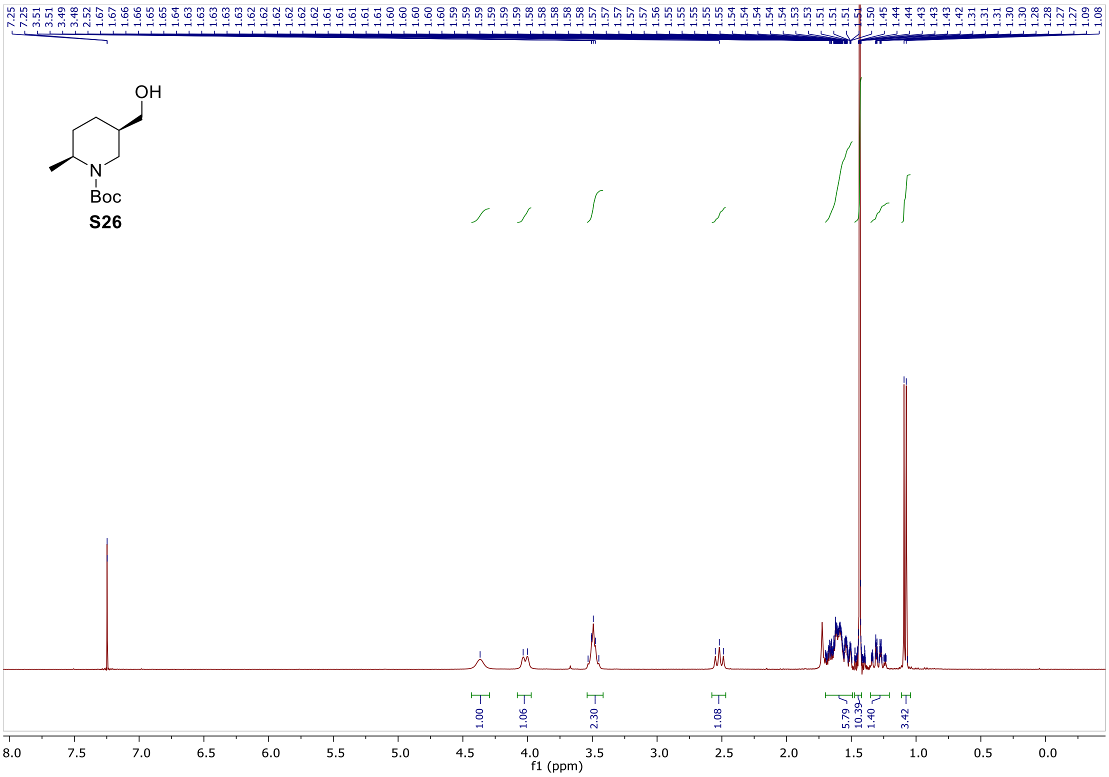

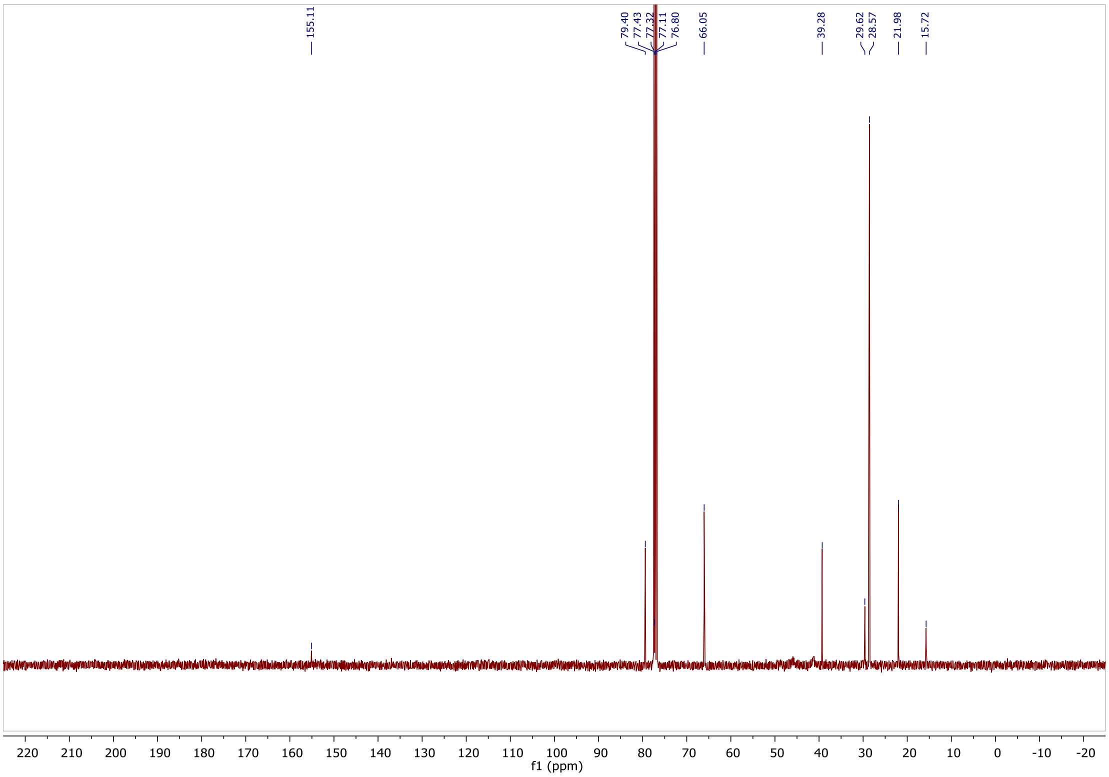

400 MHz ^{1}H NMR spectrum; 100.6 MHz ^{13}C NMR spectrum; MeOH-d_4

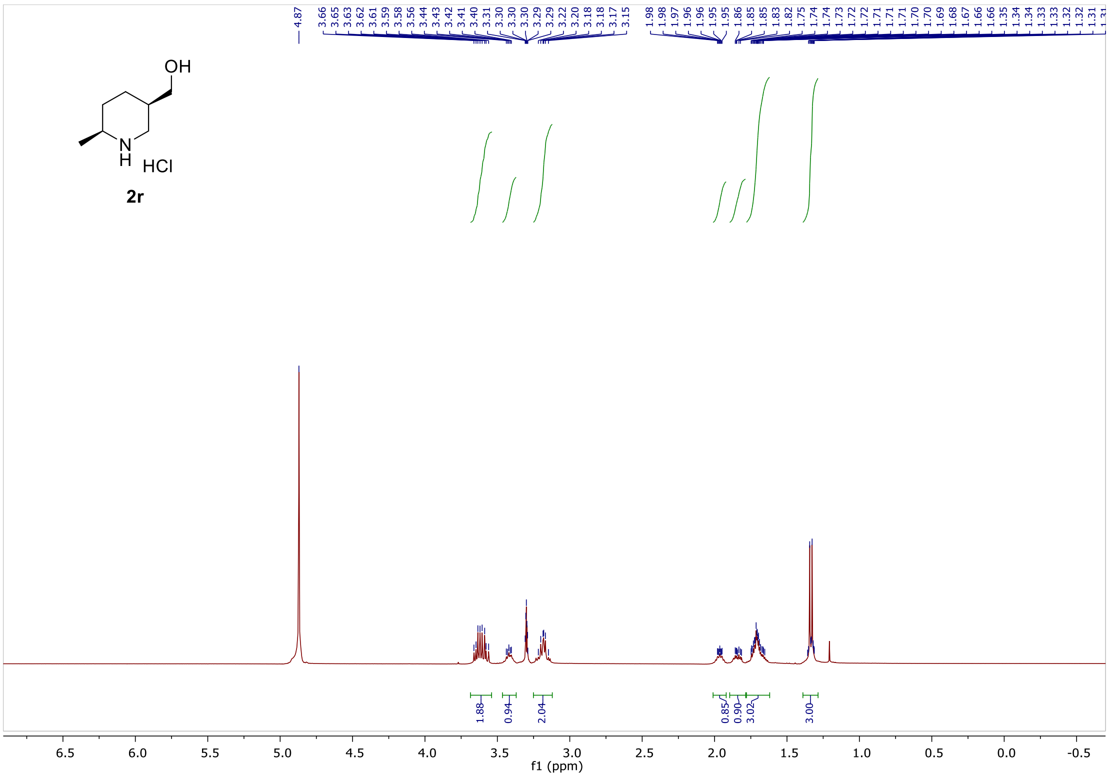

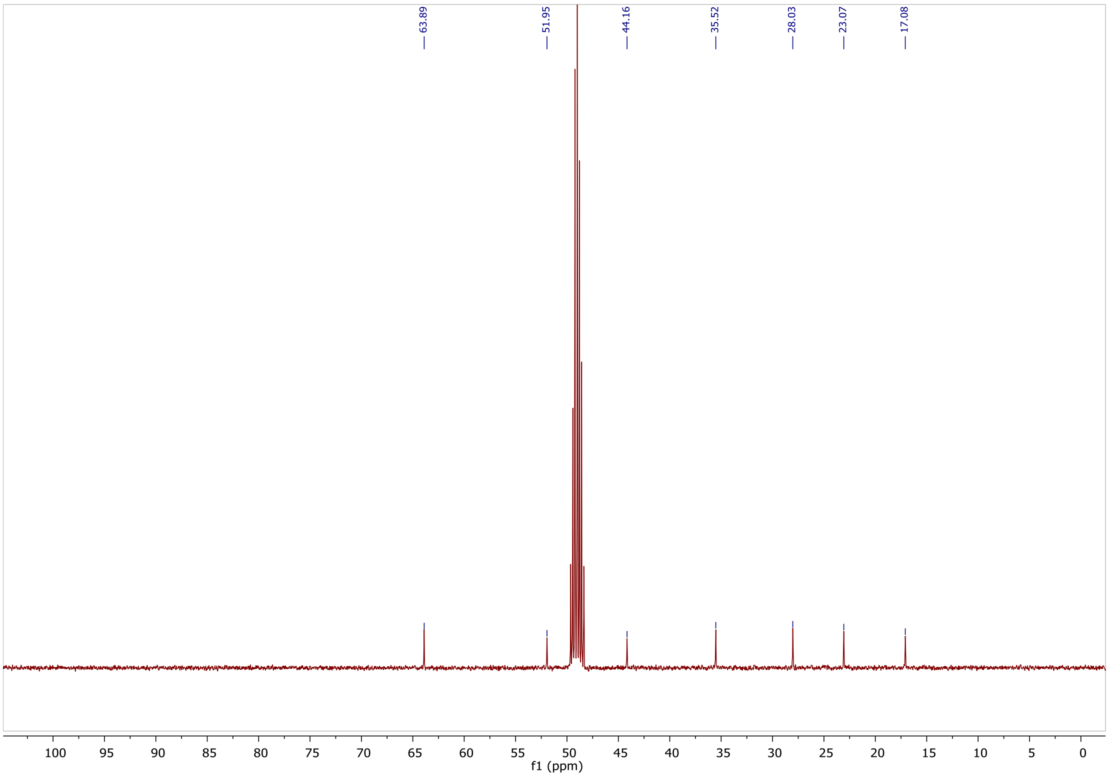

400 MHz ^{1}H NMR spectrum; 100.6 MHz ^{13}C NMR spectrum; $CDCl_3$

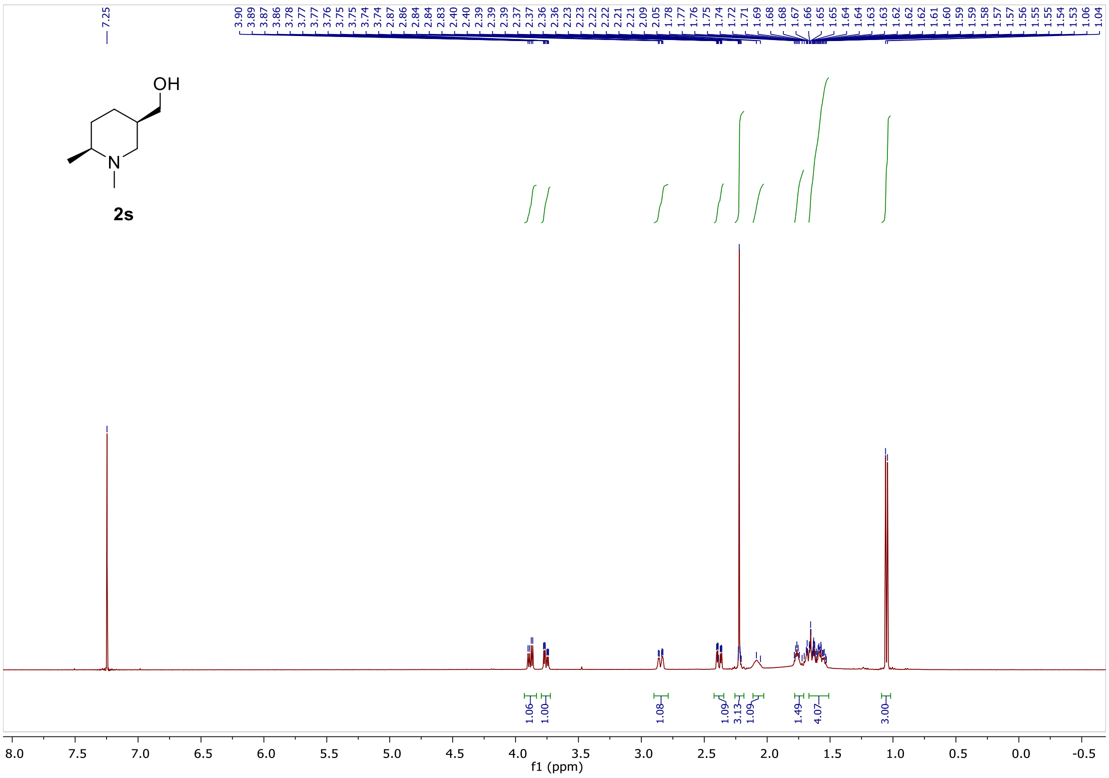

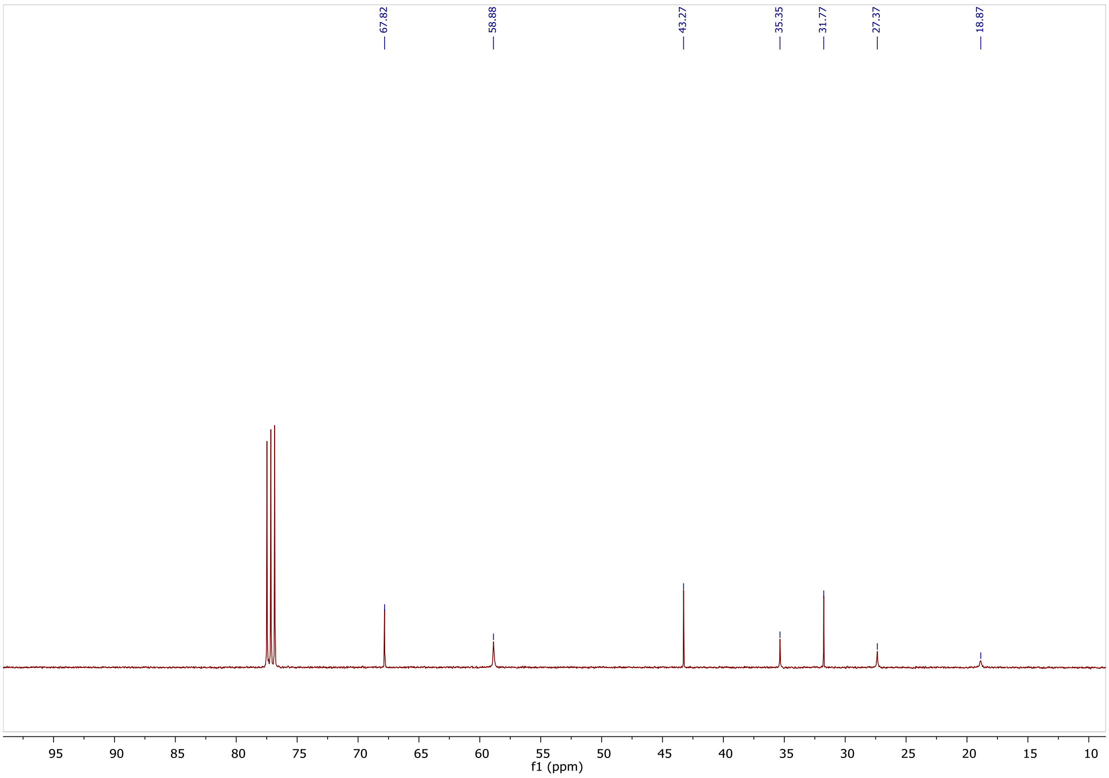

400 MHz ^{1}H NMR spectrum; 100.6 MHz ^{13}C NMR spectrum; $CDCl_3$

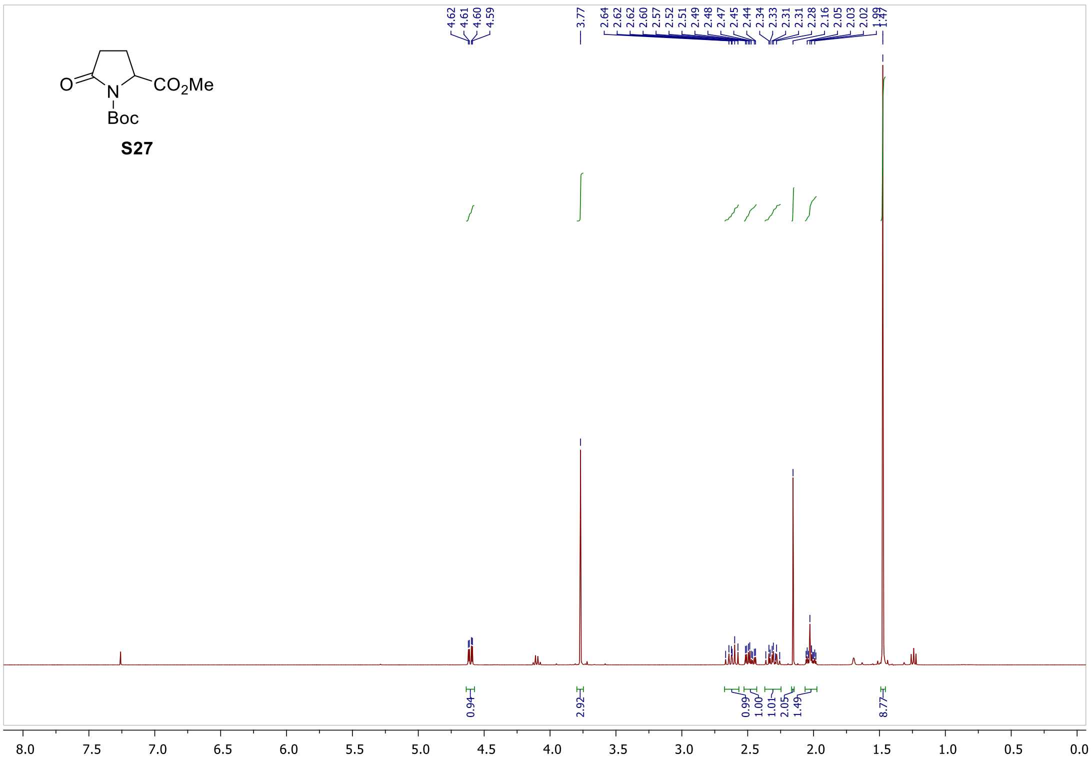

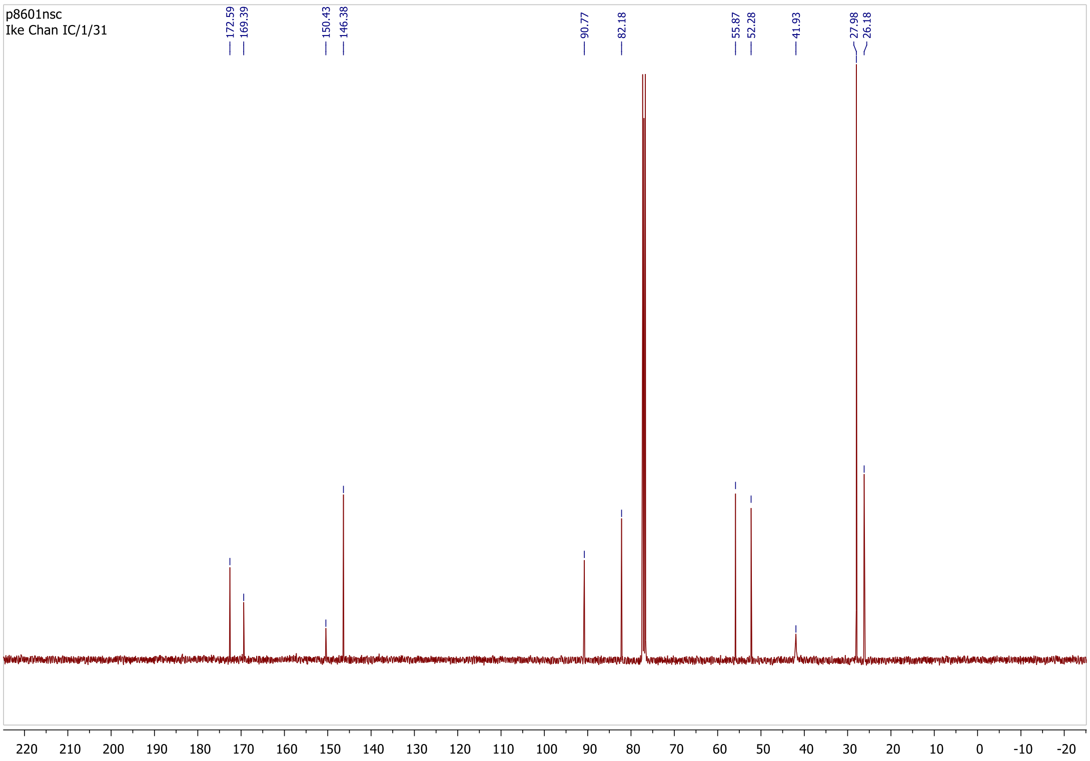

400 MHz ^{1}H NMR spectrum; 100.6 MHz ^{13}C NMR spectrum; $CDCl_3$

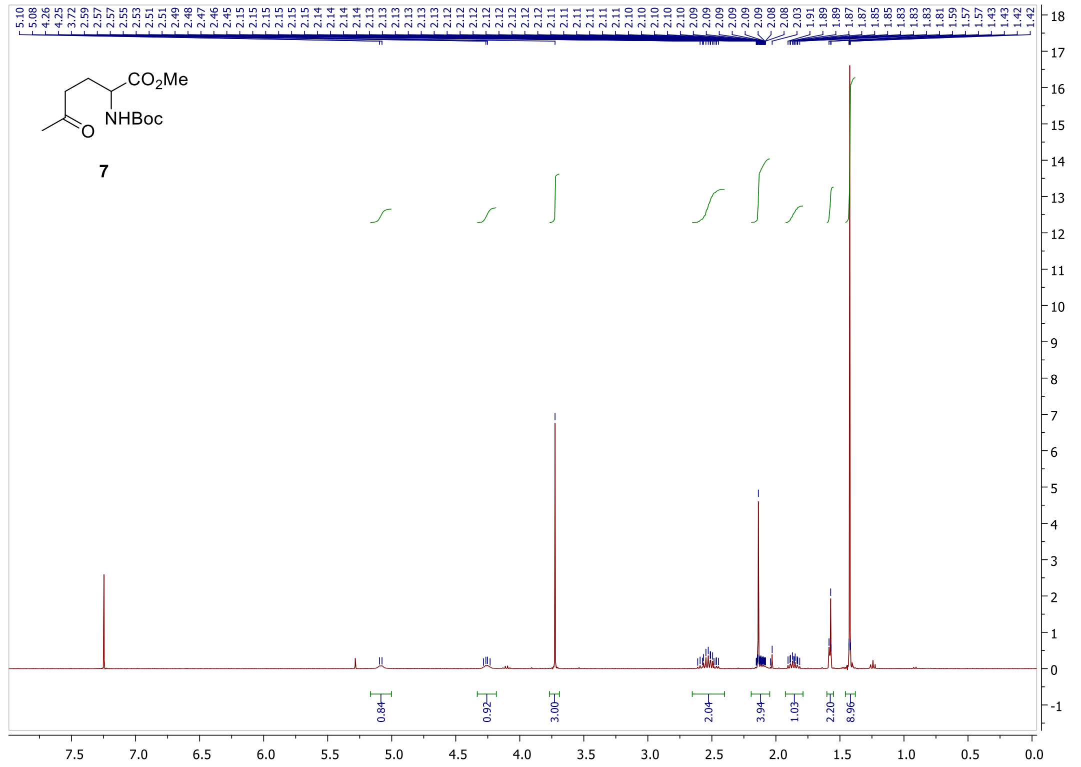

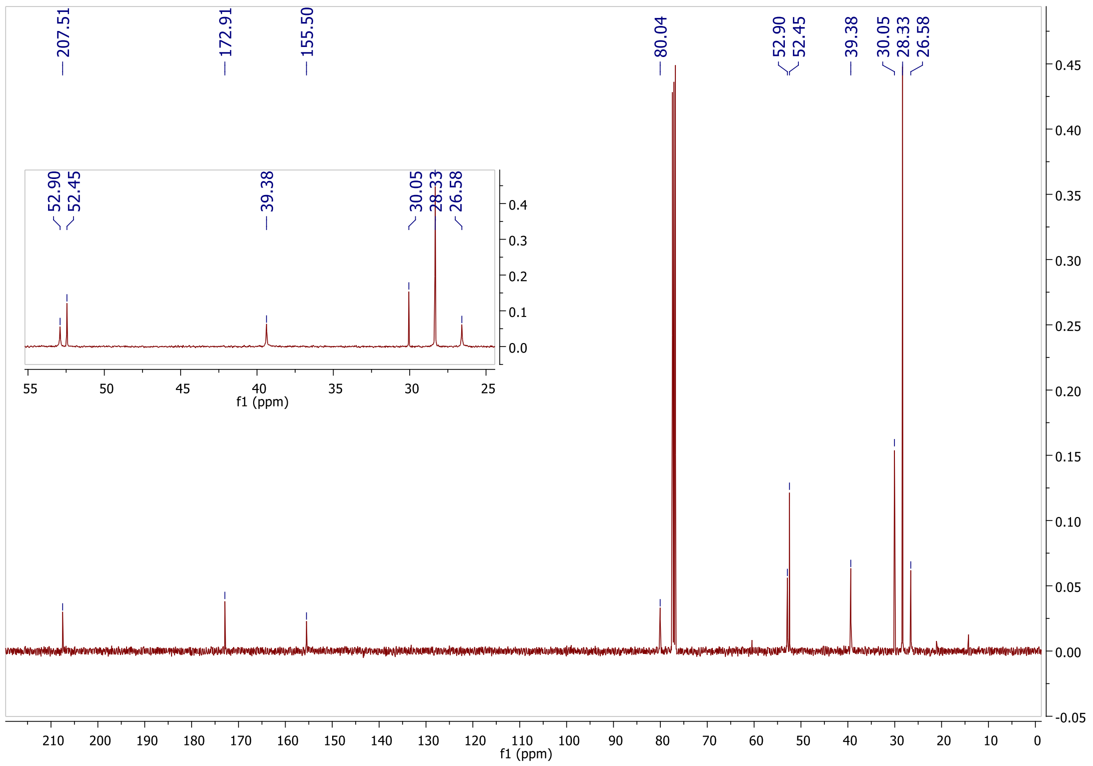

400 MHz ^{1}H NMR spectrum; 100.6 MHz ^{13}C NMR spectrum; $CDCl_3$

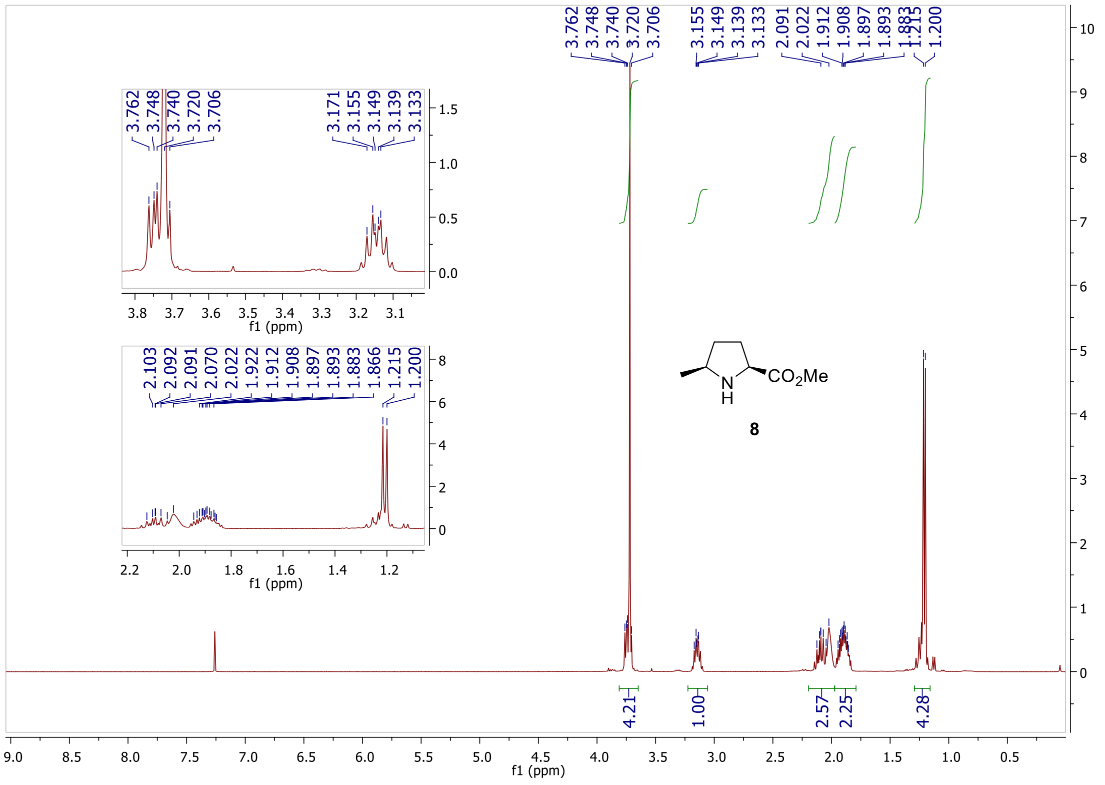

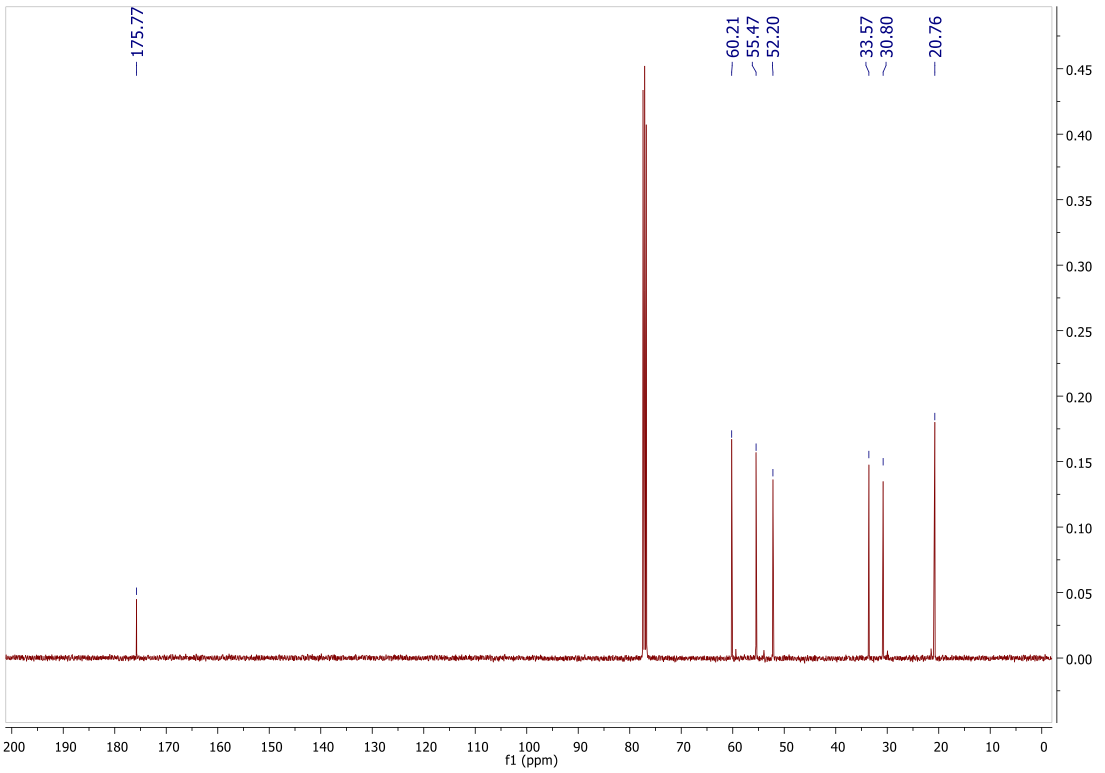

400 MHz ^{1}H NMR spectrum; 100.6 MHz ^{13}C NMR spectrum; $CDCl_3$

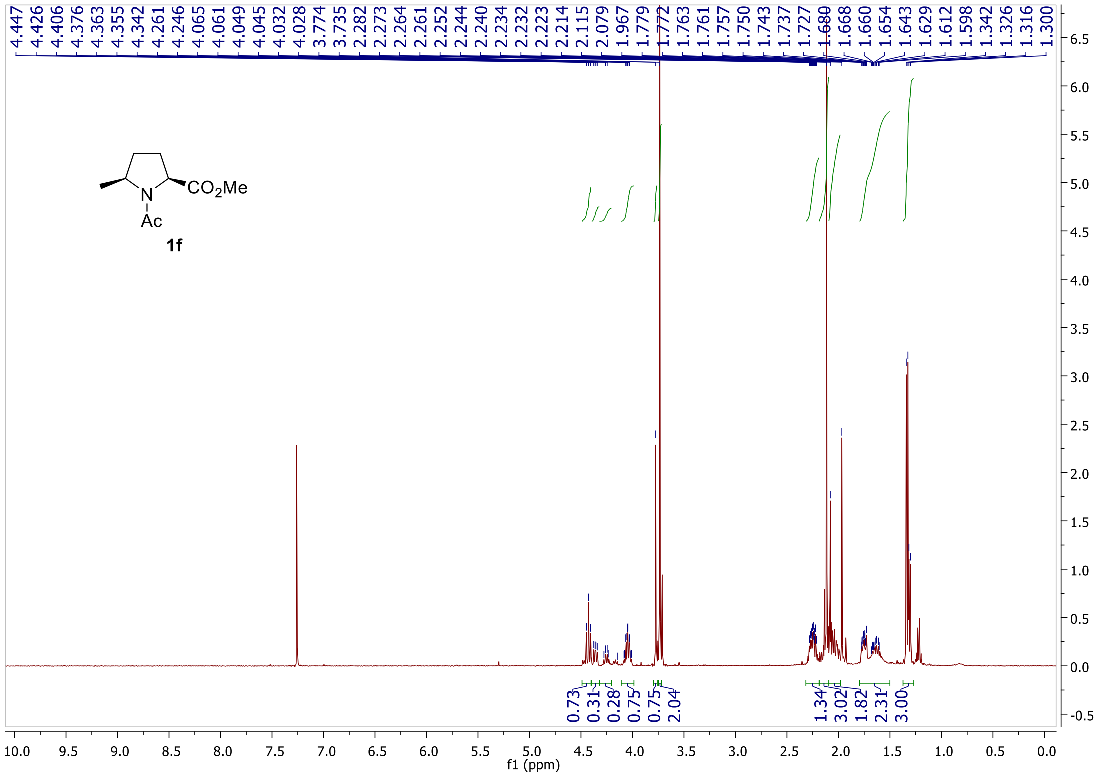

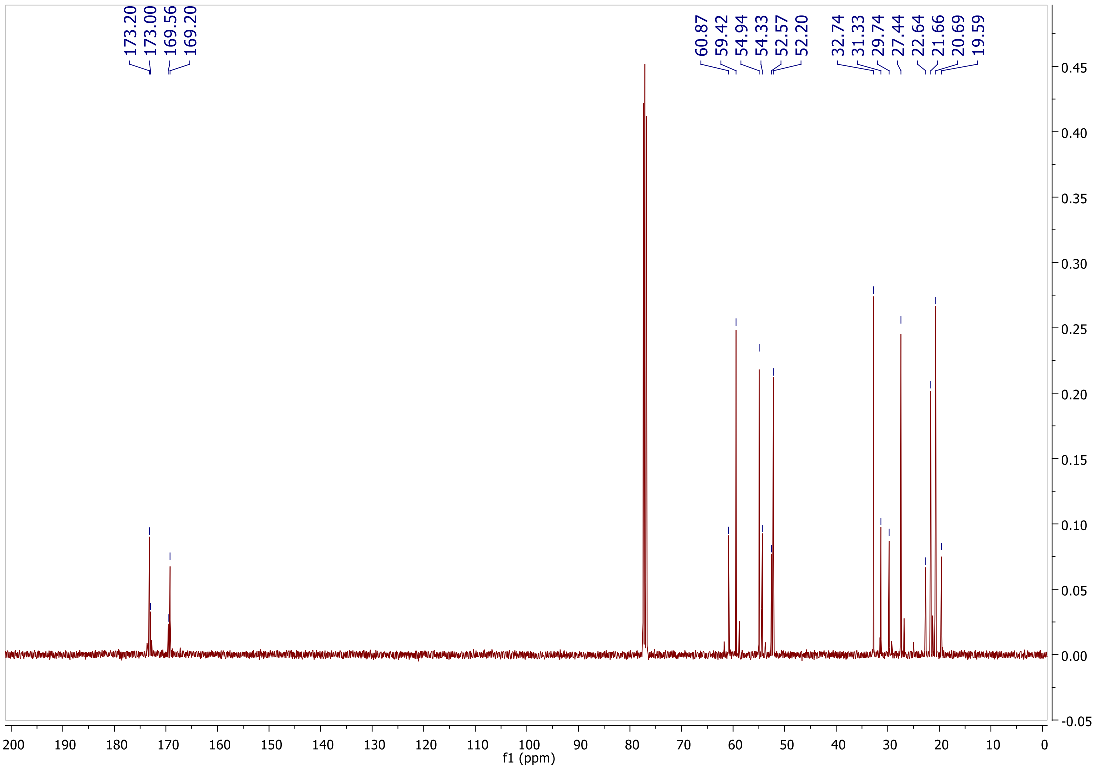

400 MHz ^{1}H NMR spectrum; 100.6 MHz ^{13}C NMR spectrum; $CDCl_3$

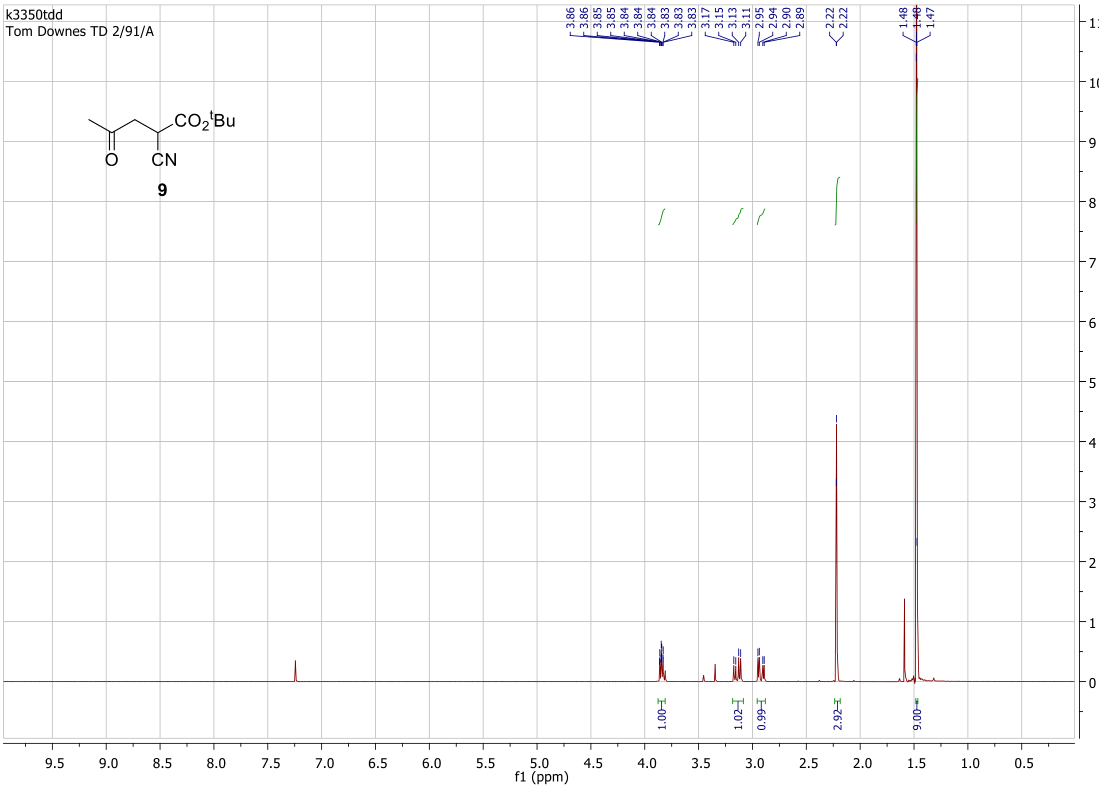

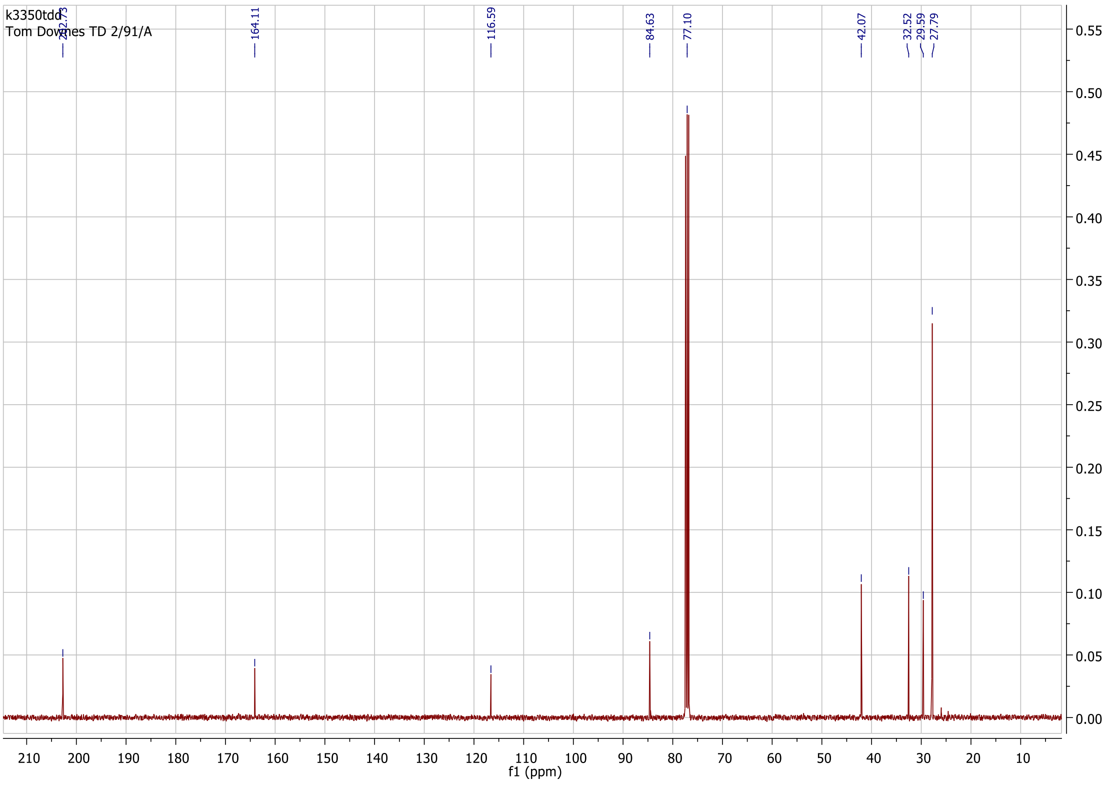

400 MHz ^{1}H NMR spectrum; 100.6 MHz ^{13}C NMR spectrum; $CDCl_3$

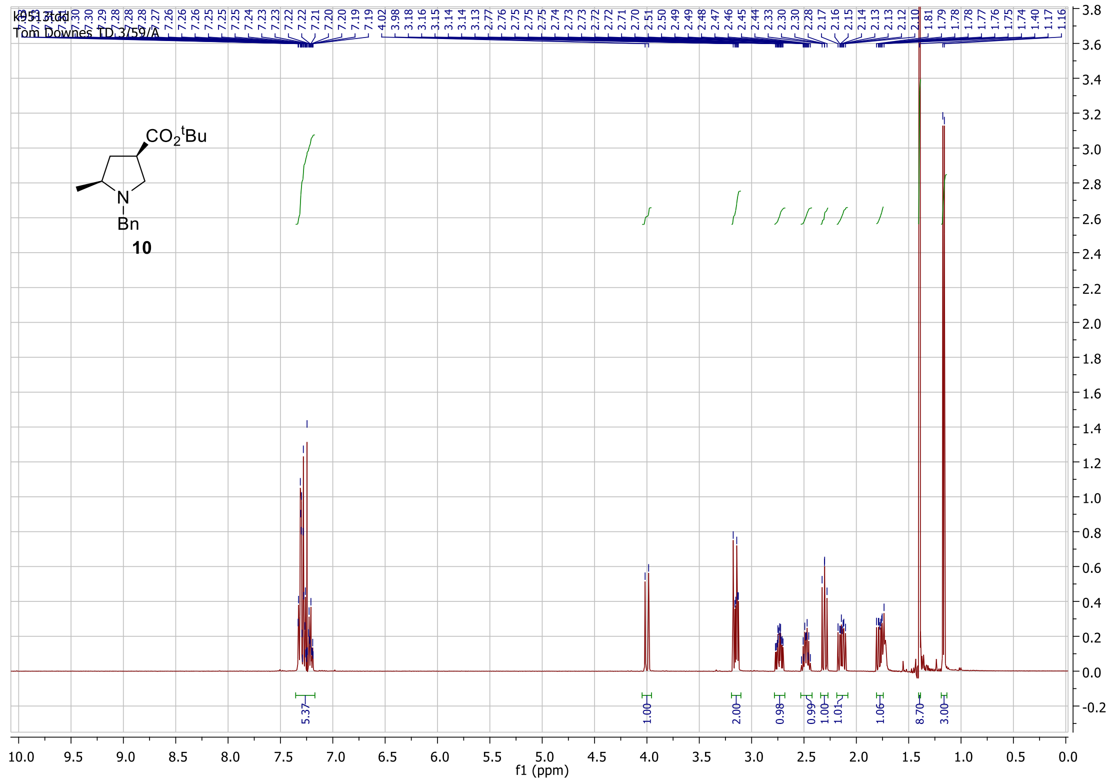

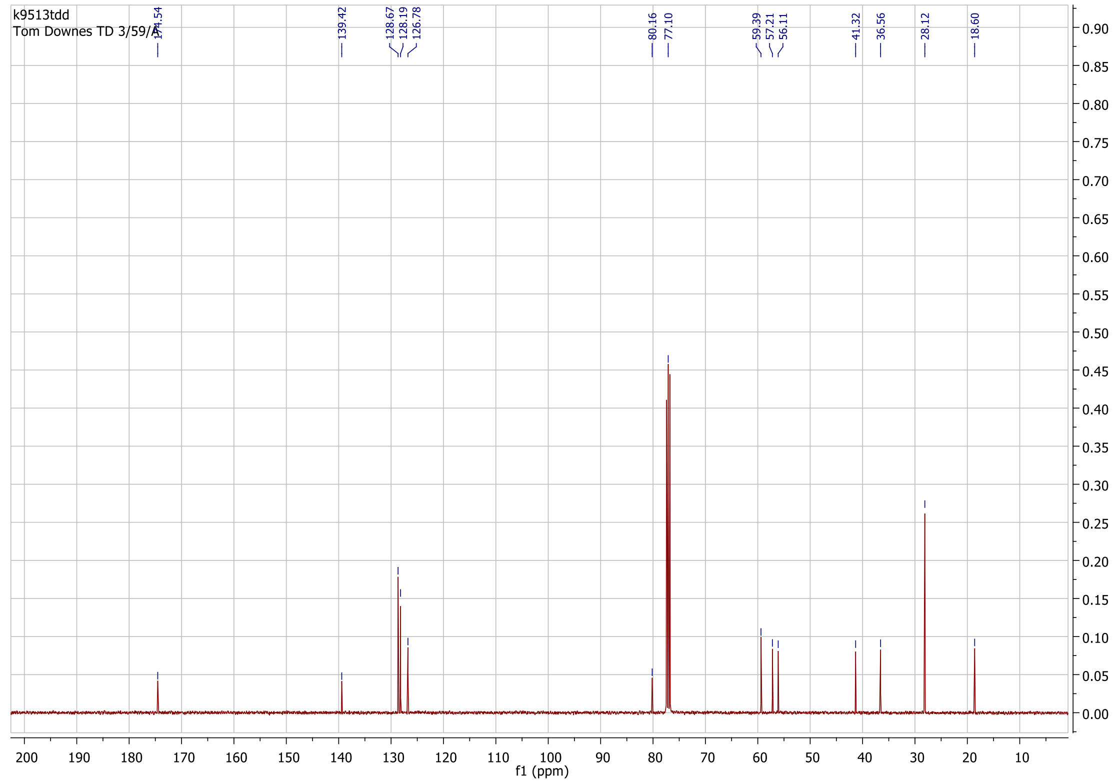

400 MHz ^{1}H NMR spectrum; 100.6 MHz ^{13}C NMR spectrum; $CDCl_3$

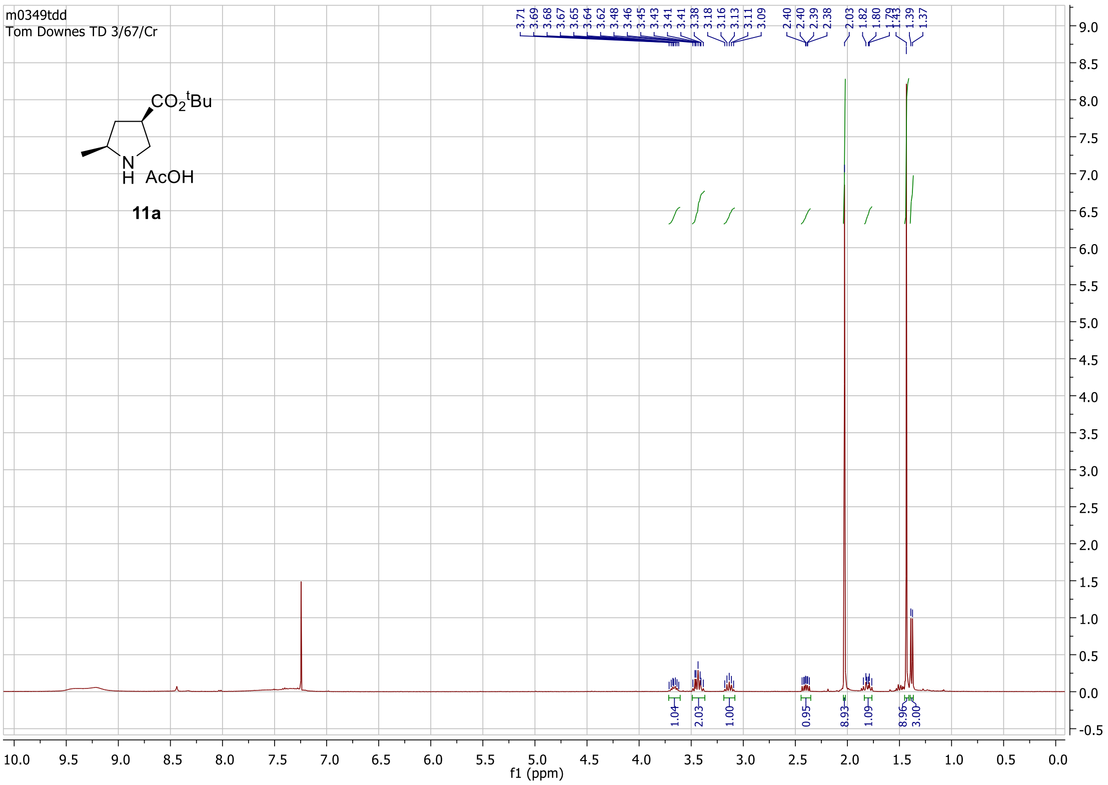

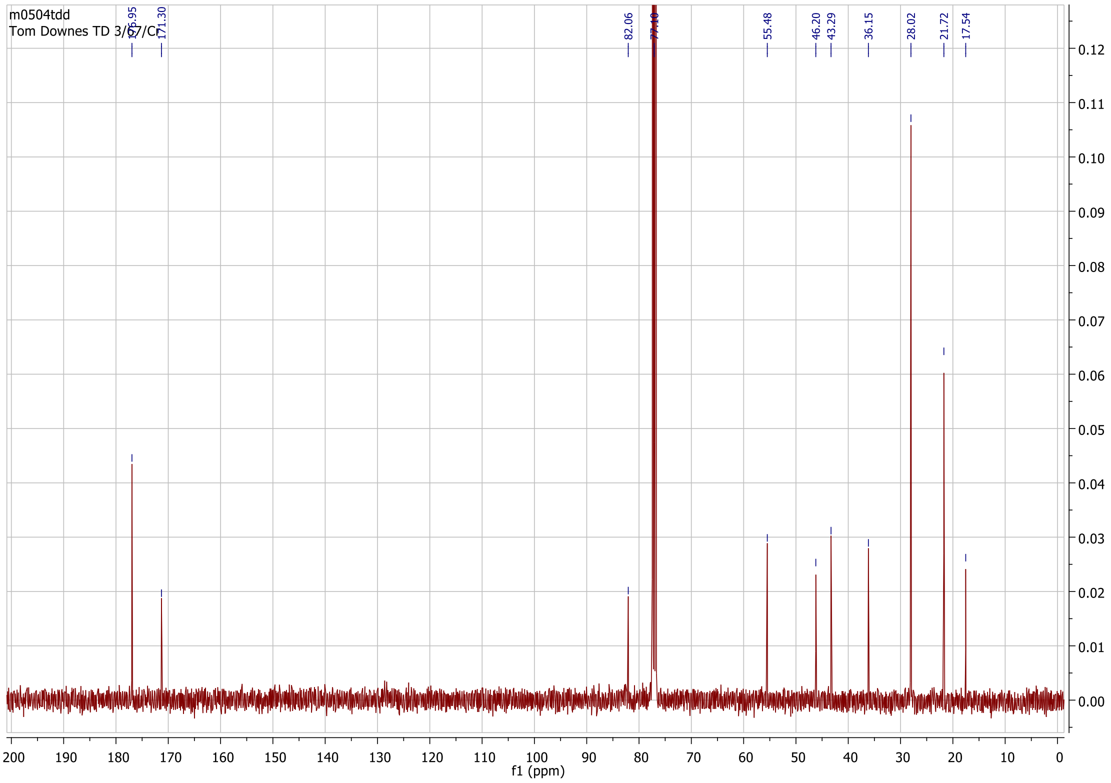

400 MHz ^{1}H NMR spectrum; 100.6 MHz ^{13}C NMR spectrum; $CDCl_3$

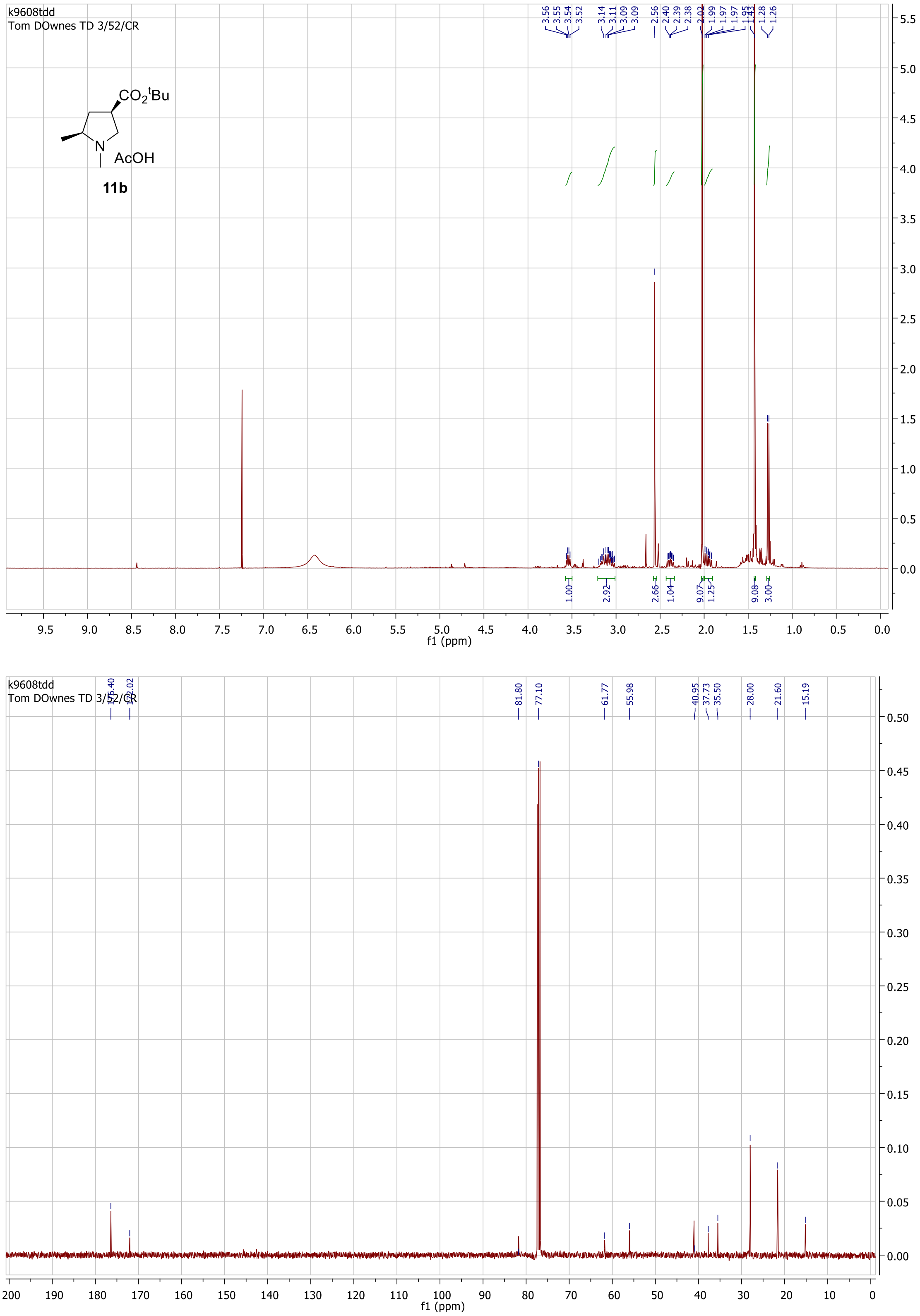

400 MHz ^{1}H NMR spectrum; 100.6 MHz ^{13}C NMR spectrum; $CDCl_3$

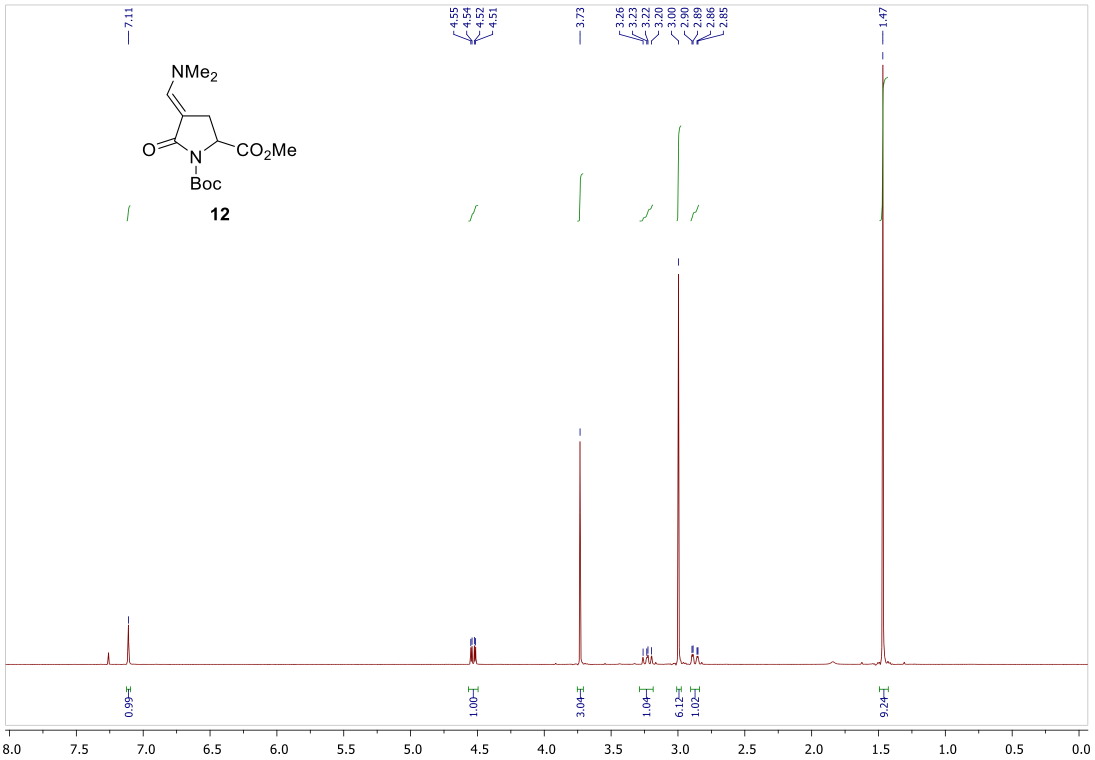

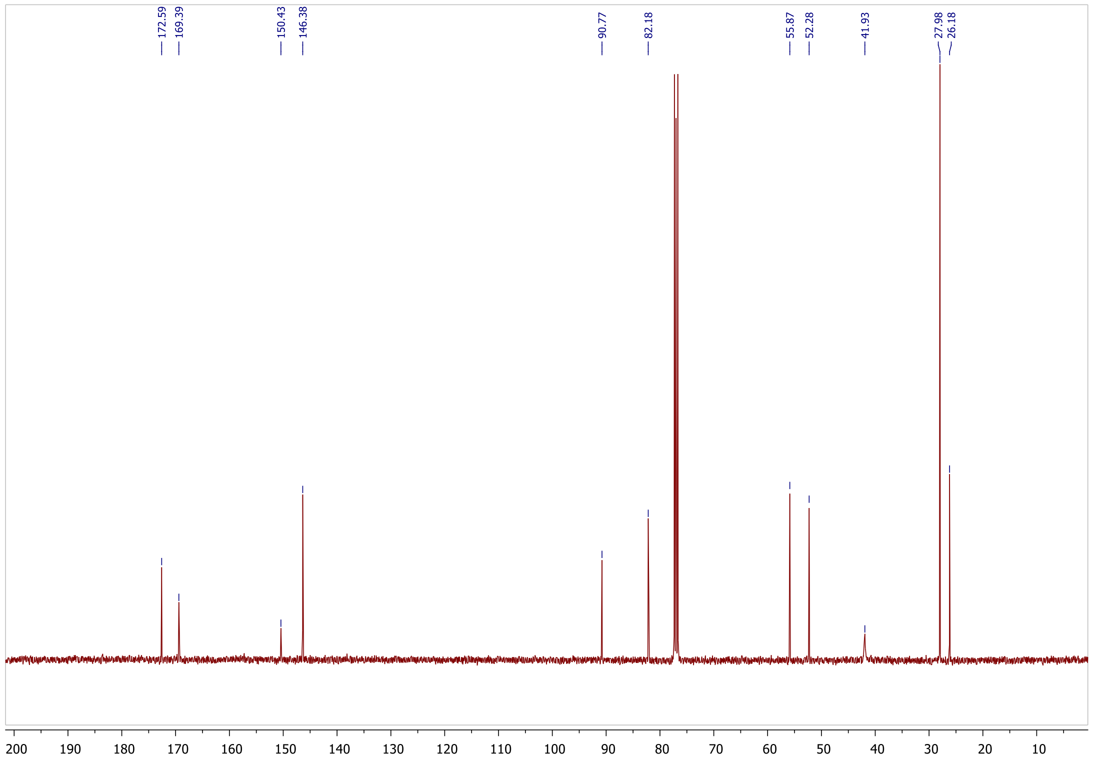

400 MHz ^{1}H NMR spectrum; 100.6 MHz ^{13}C NMR spectrum; $CDCl_3$

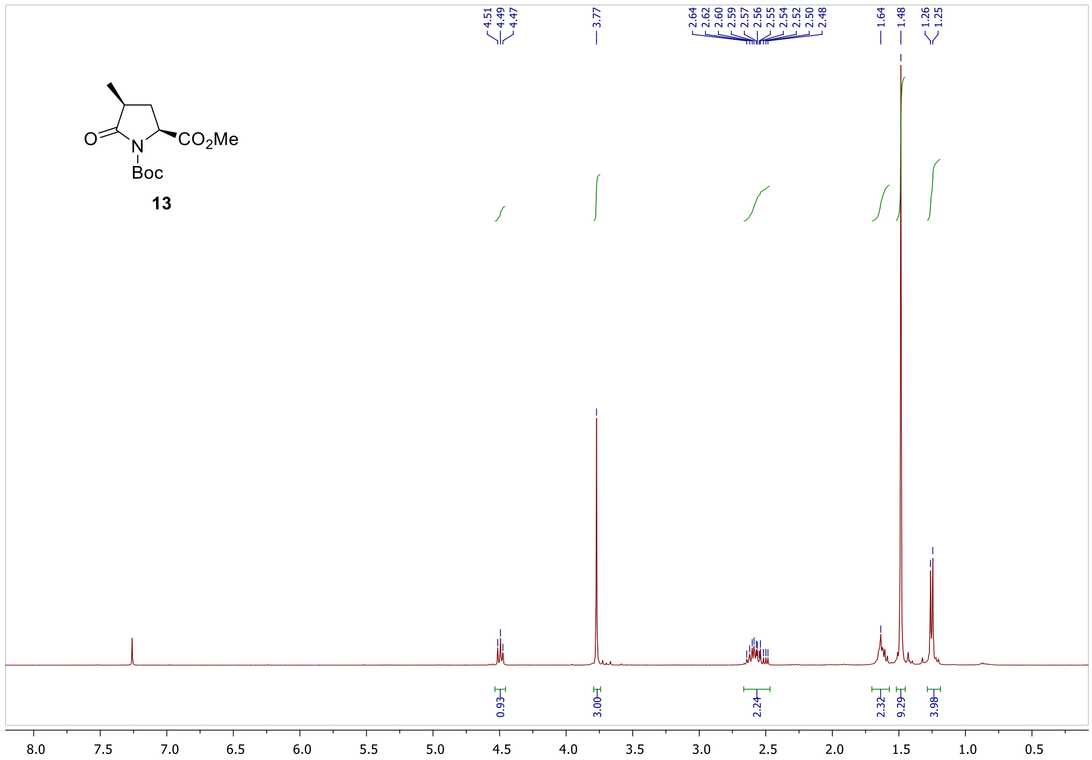

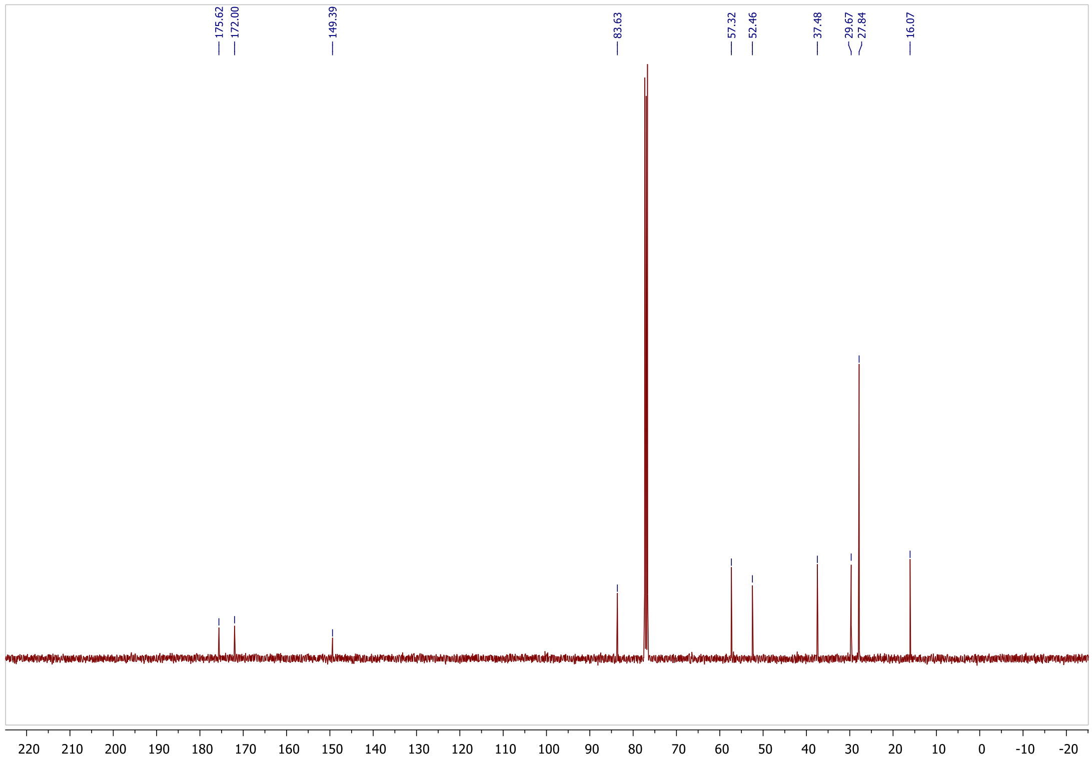

400 MHz ^{1}H NMR spectrum; 100.6 MHz ^{13}C NMR spectrum; $CDCl_3$

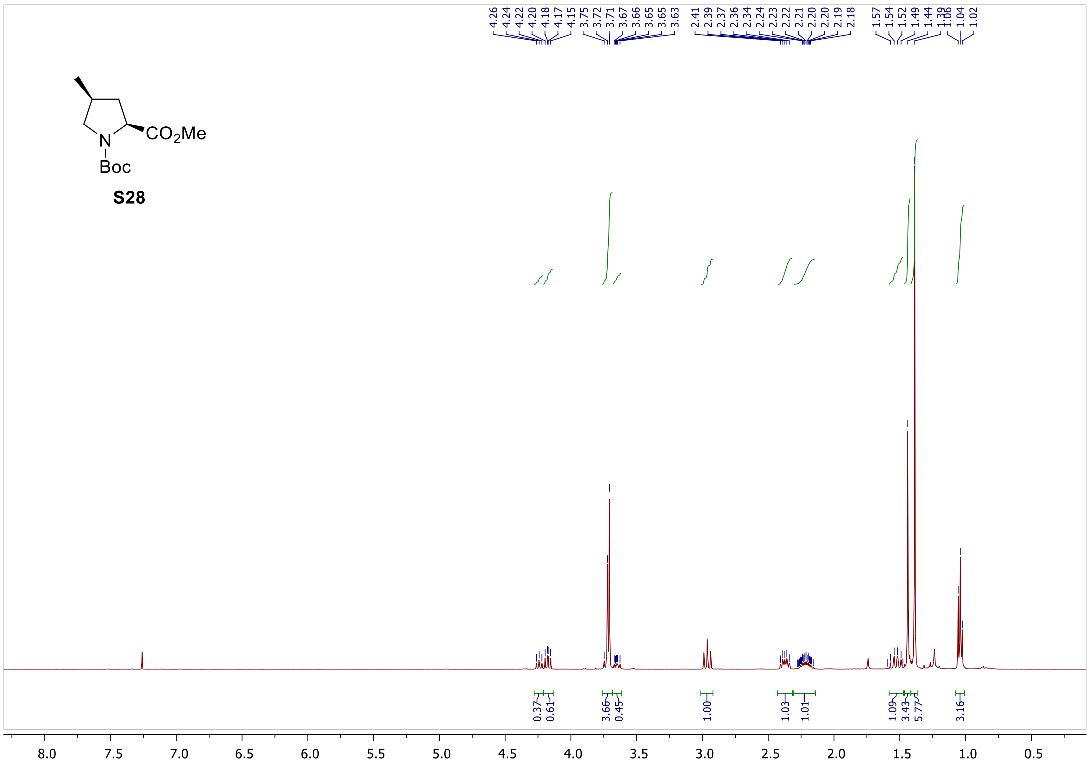

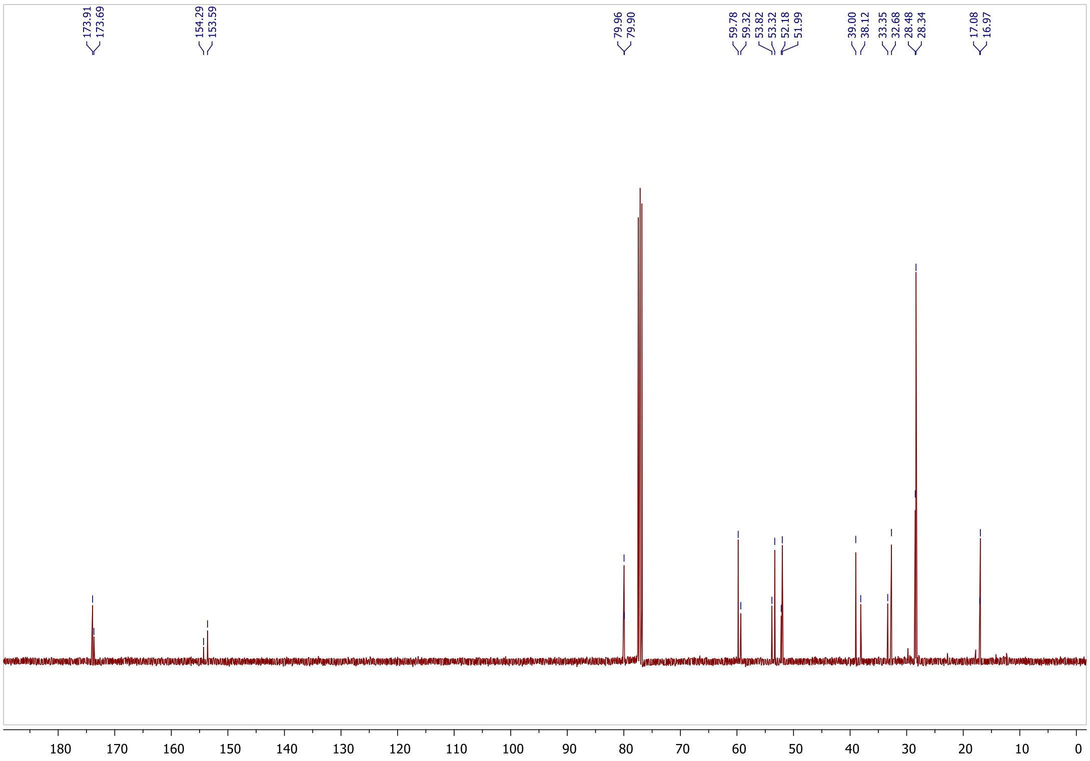

400 MHz ^{1}H NMR spectrum; 100.6 MHz ^{13}C NMR spectrum; MeOH-d_4

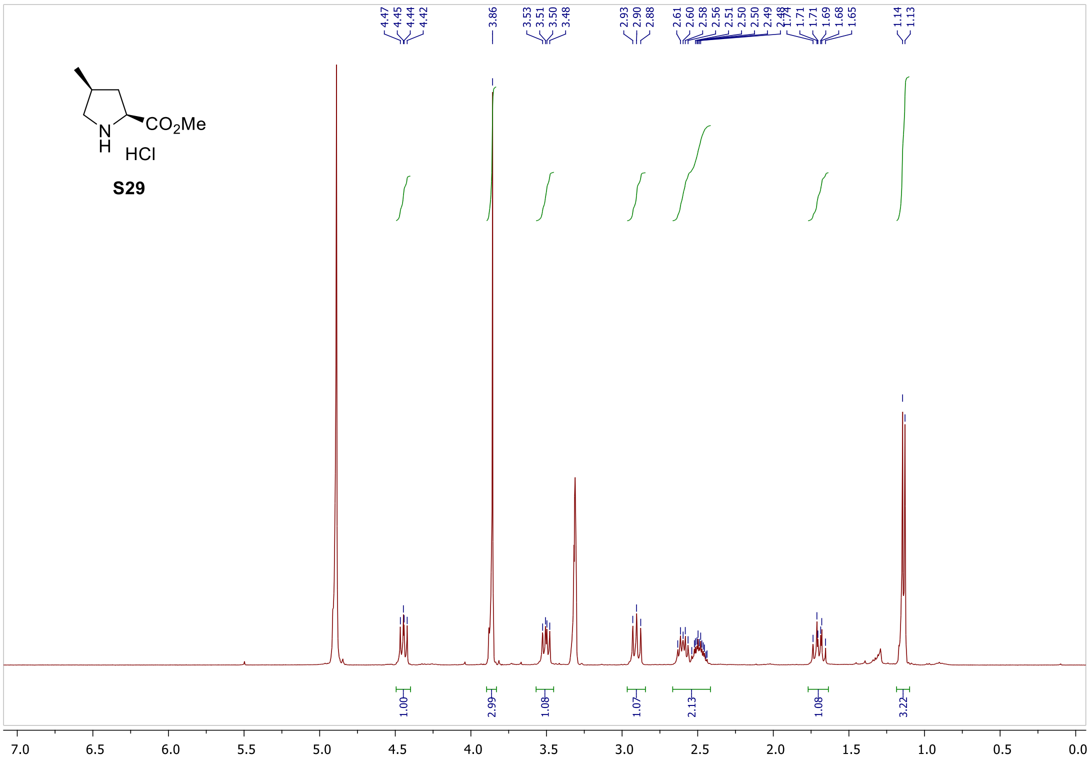

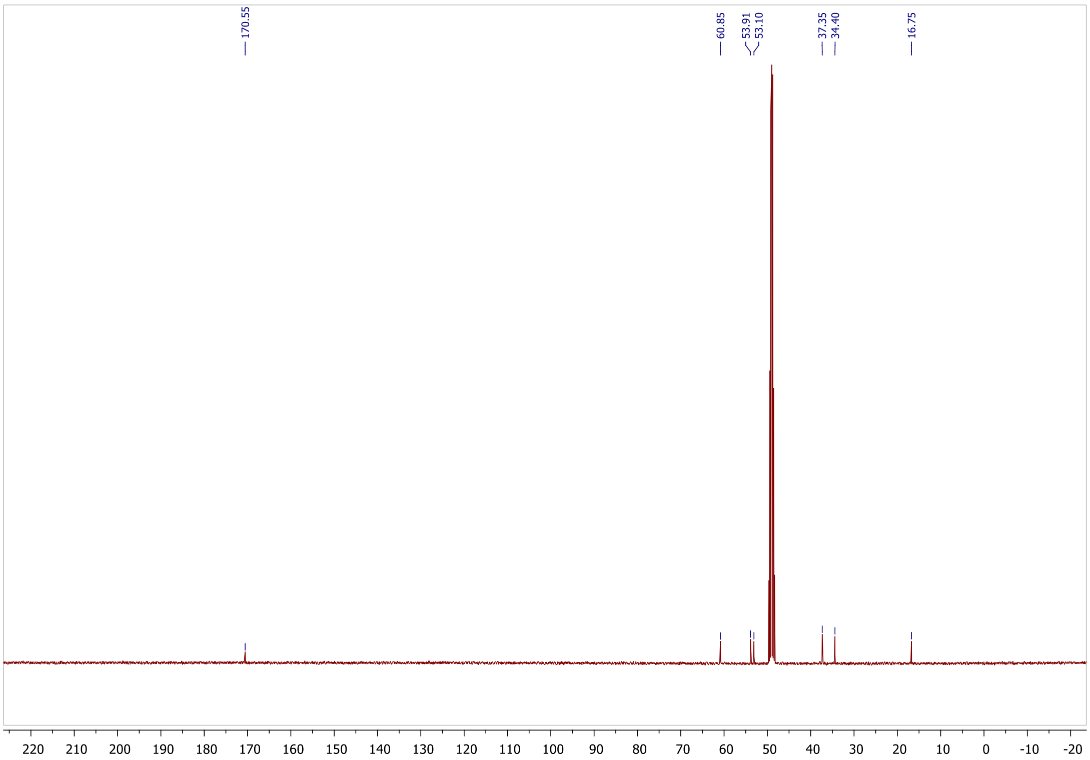

400 MHz ^{1}H NMR spectrum; 100.6 MHz ^{13}C NMR spectrum; $CDCl_3$

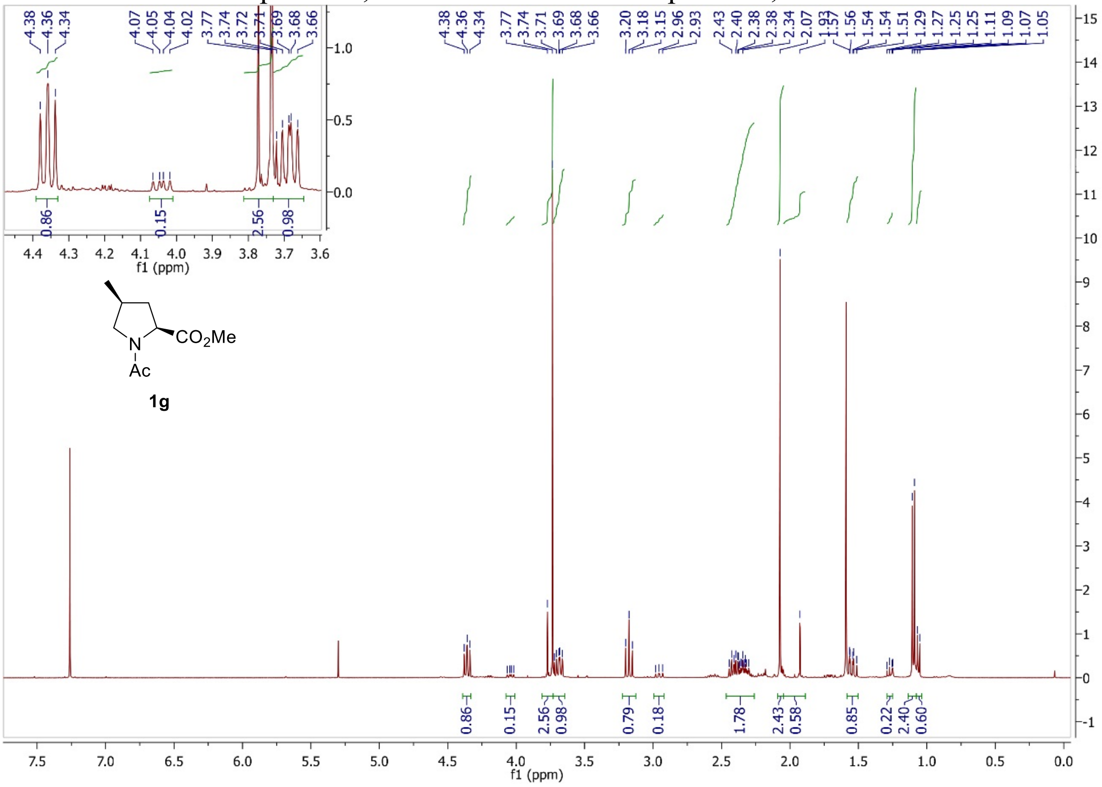

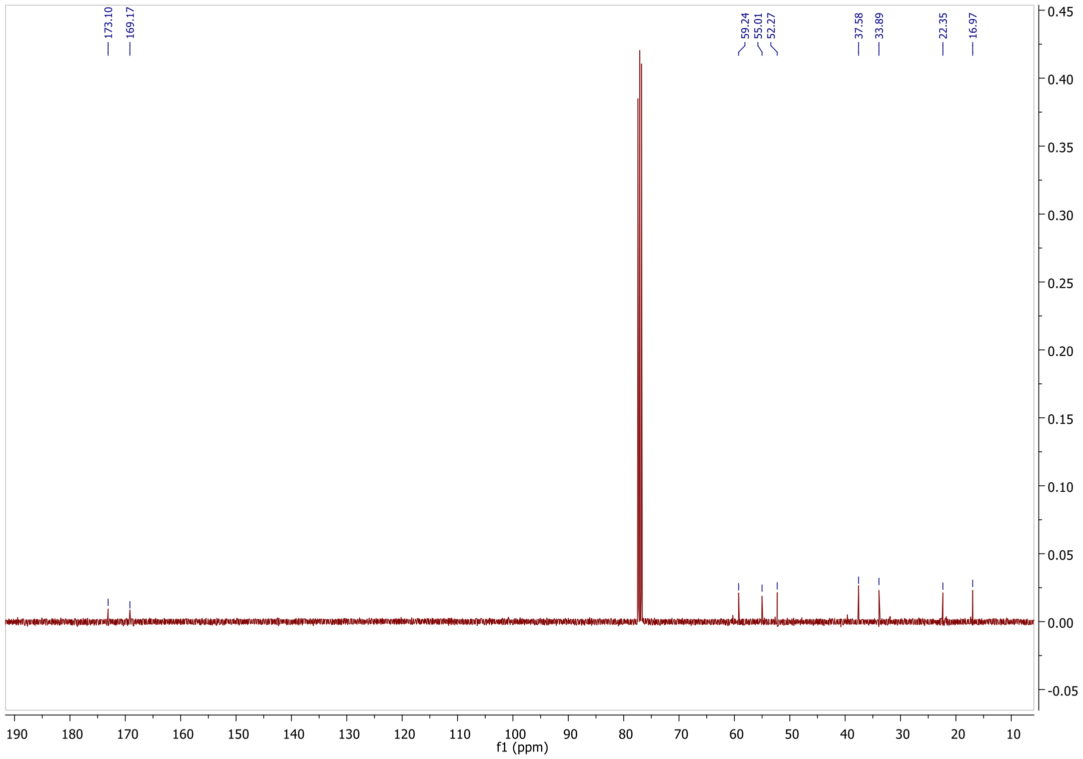

400 MHz ^{1}H NMR spectrum; 100.6 MHz ^{13}C NMR spectrum; $CDCl_3$

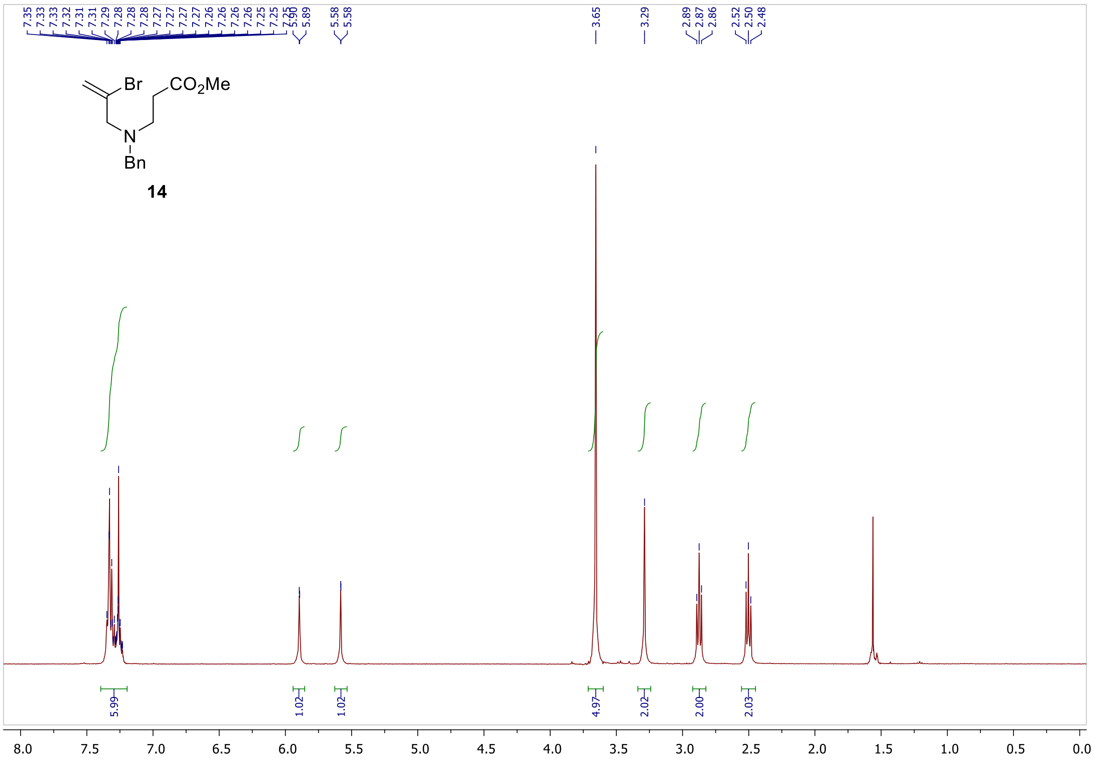

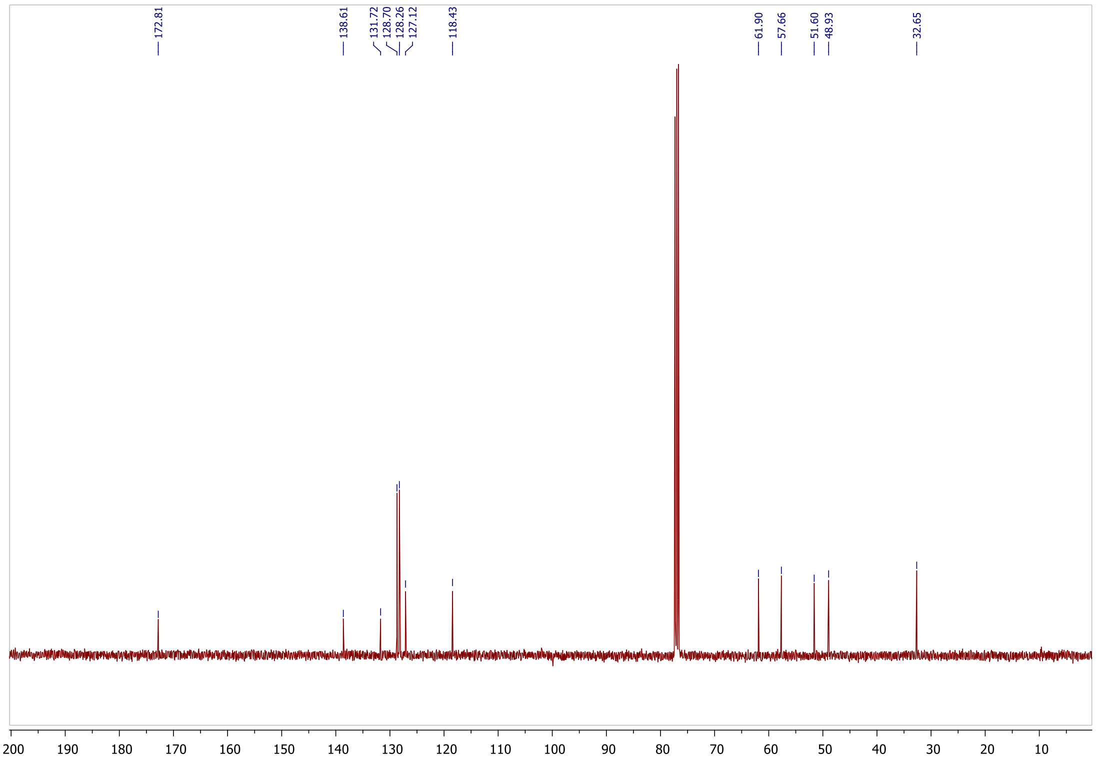

400 MHz ^{1}H NMR spectrum; 100.6 MHz ^{13}C NMR spectrum; $CDCl_3$

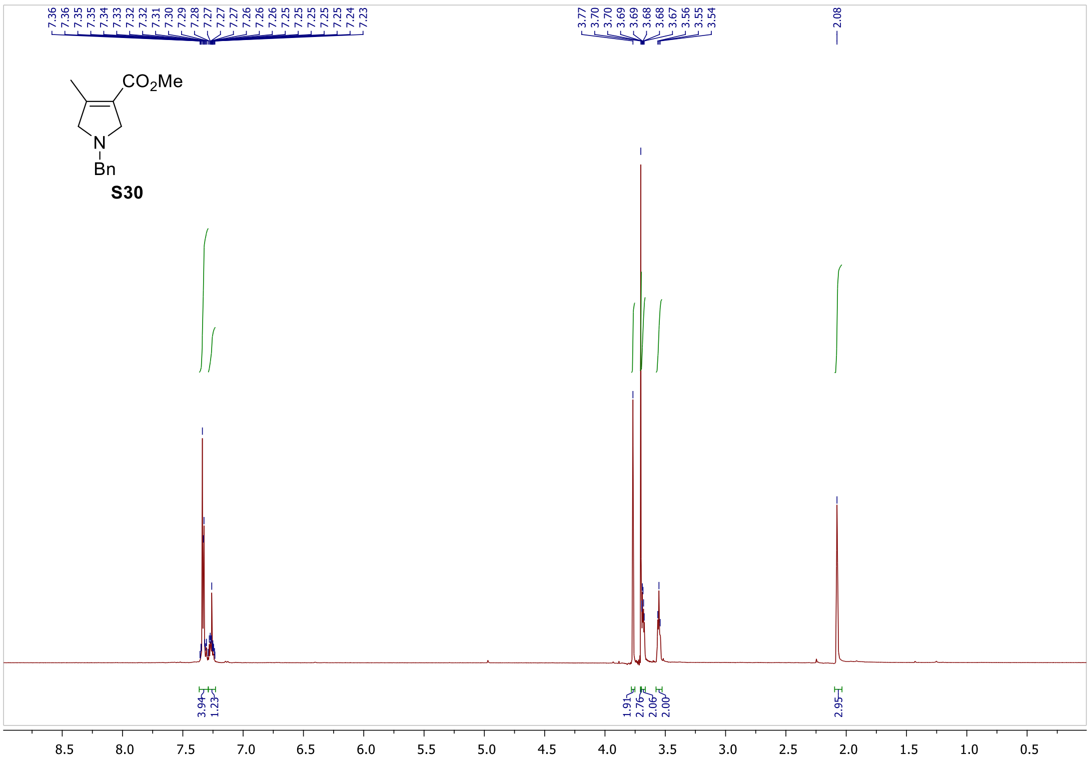

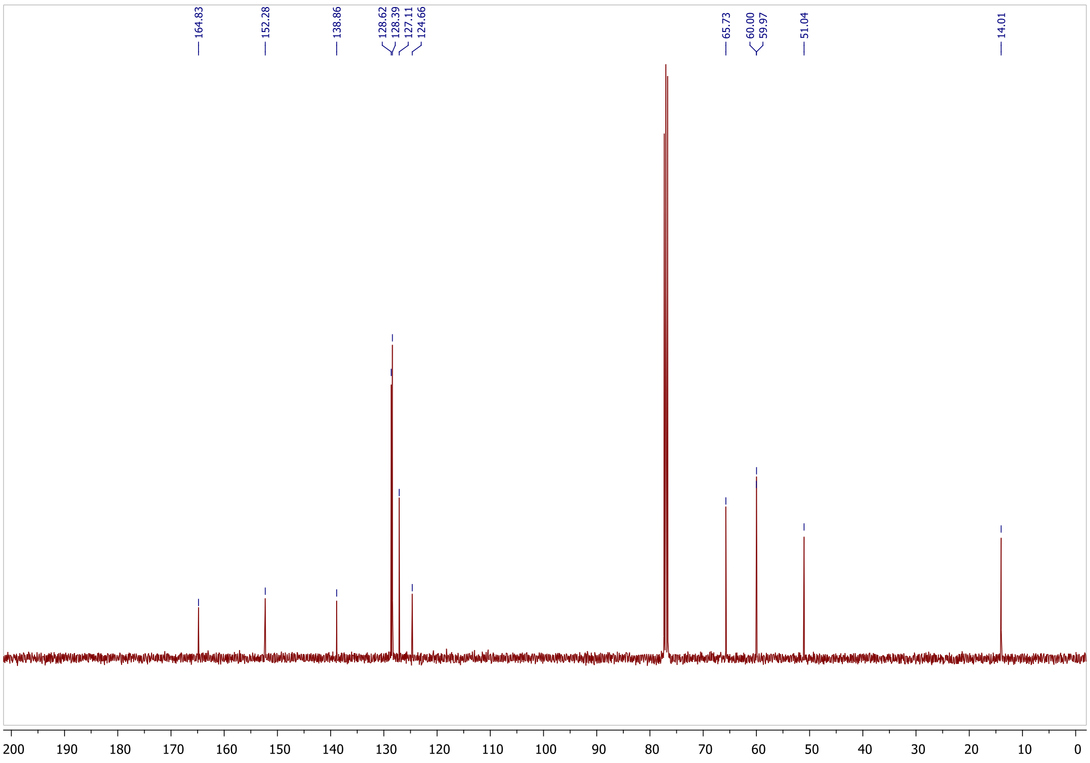

400 MHz ^{1}H NMR spectrum; 100.6 MHz ^{13}C NMR spectrum; $CDCl_3$

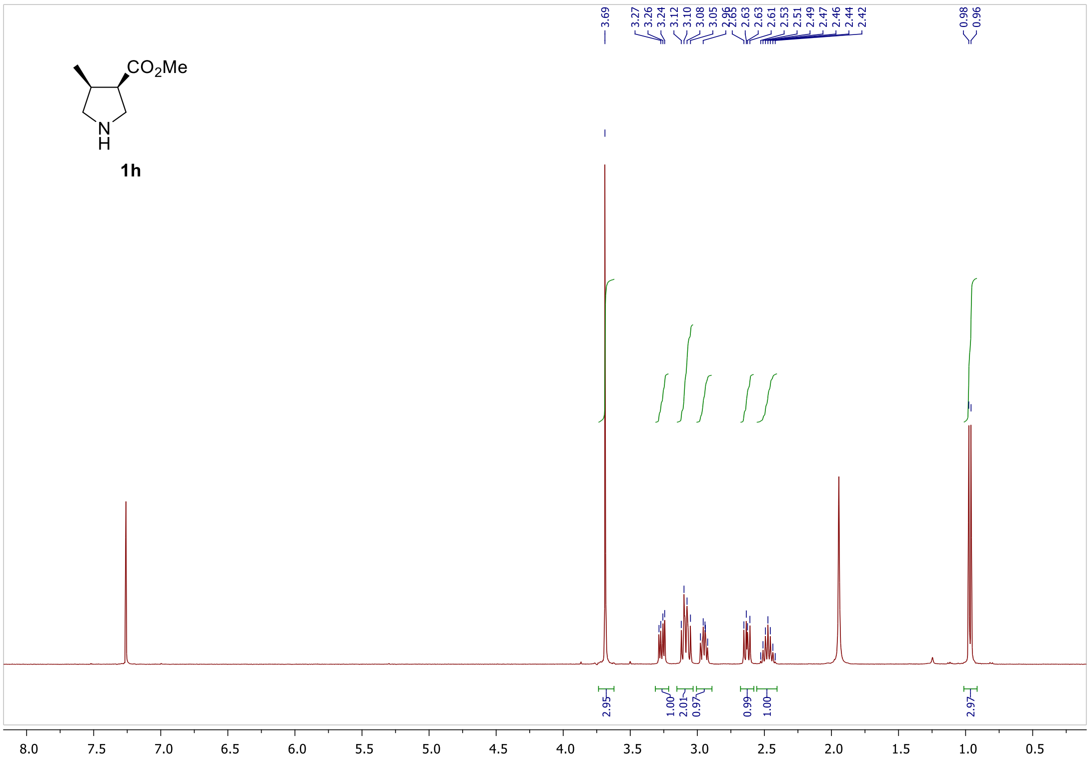

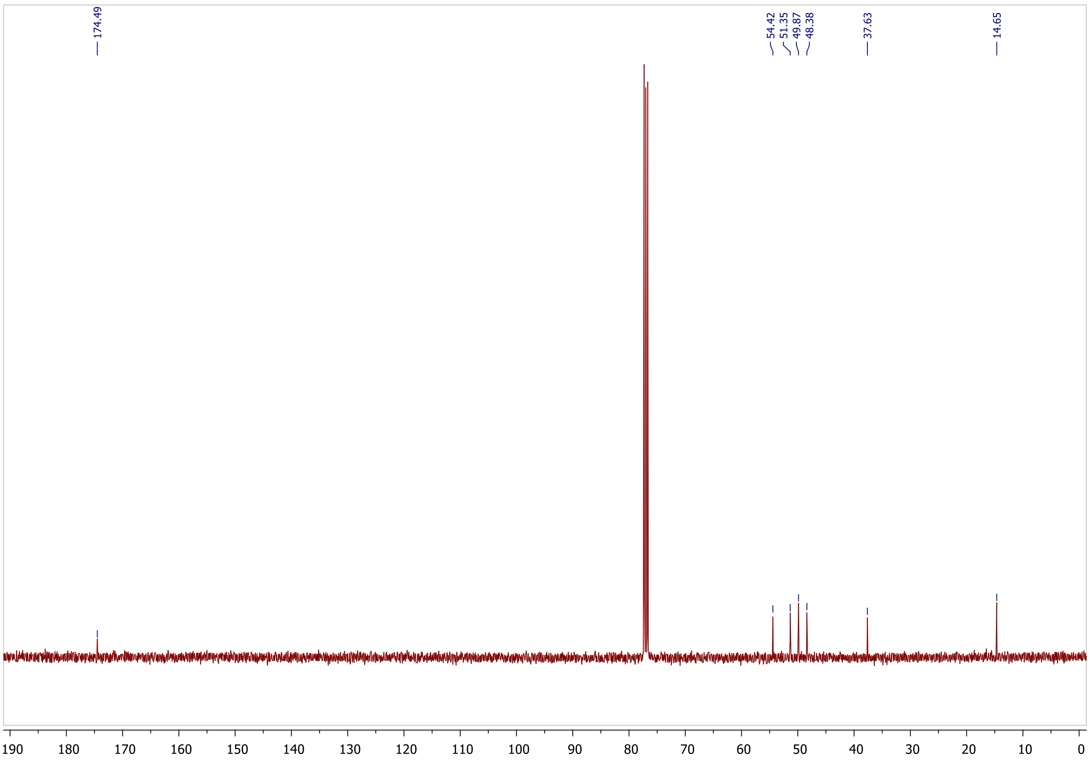

400 MHz ^{1}H NMR spectrum; 100.6 MHz ^{13}C NMR spectrum; $CDCl_3$

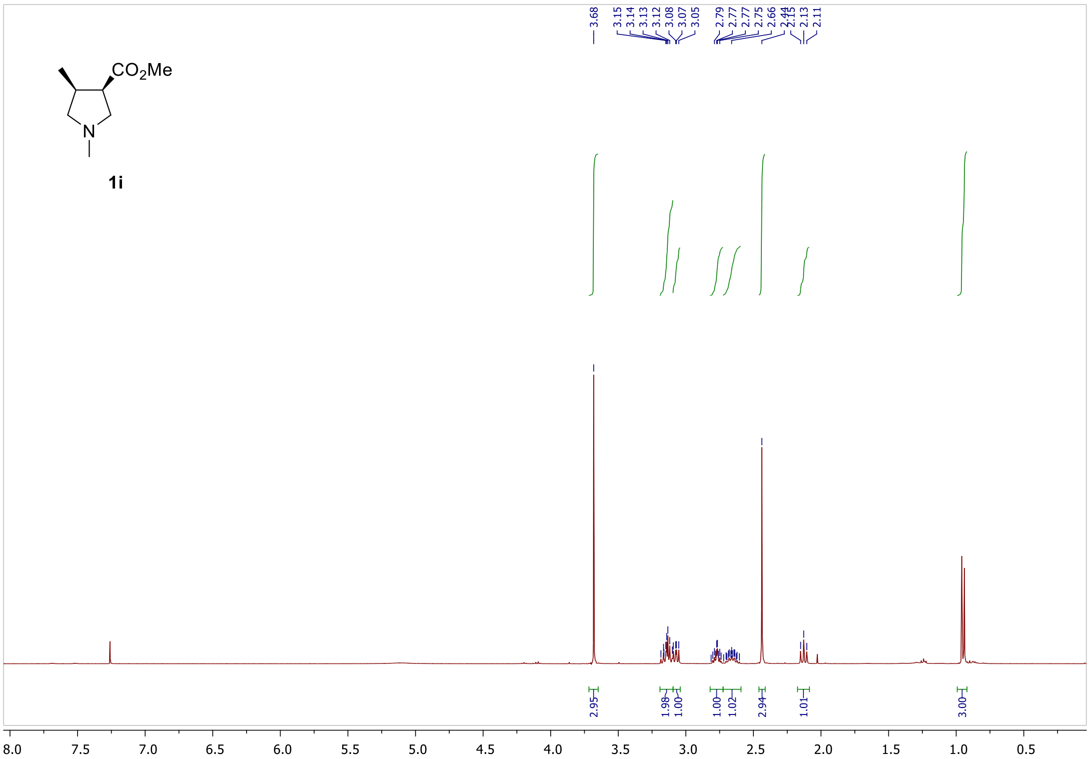

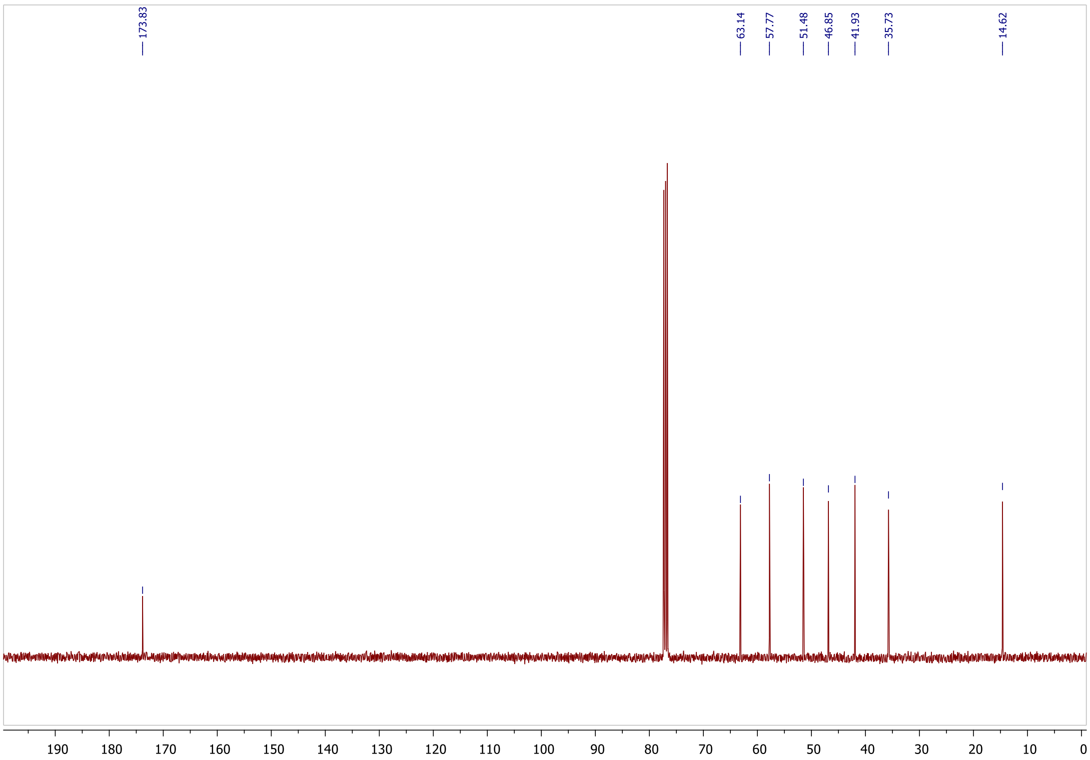

400 MHz ^{1}H NMR spectrum; 100.6 MHz ^{13}C NMR spectrum; $CDCl_3$

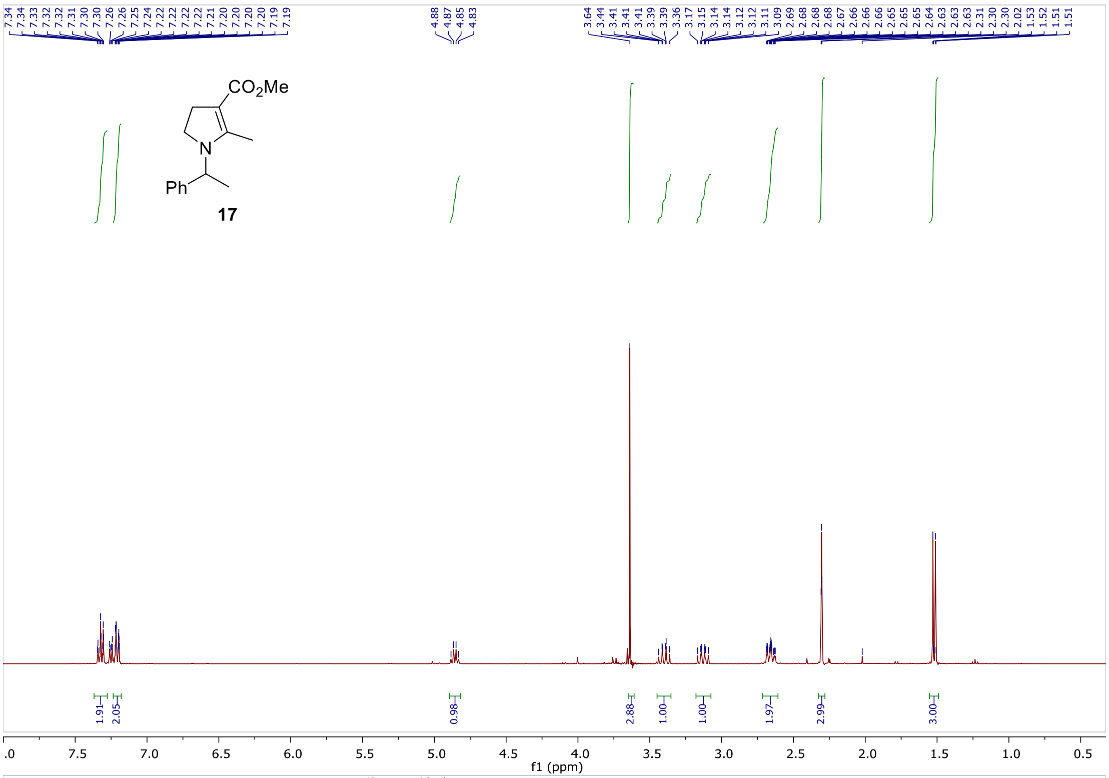

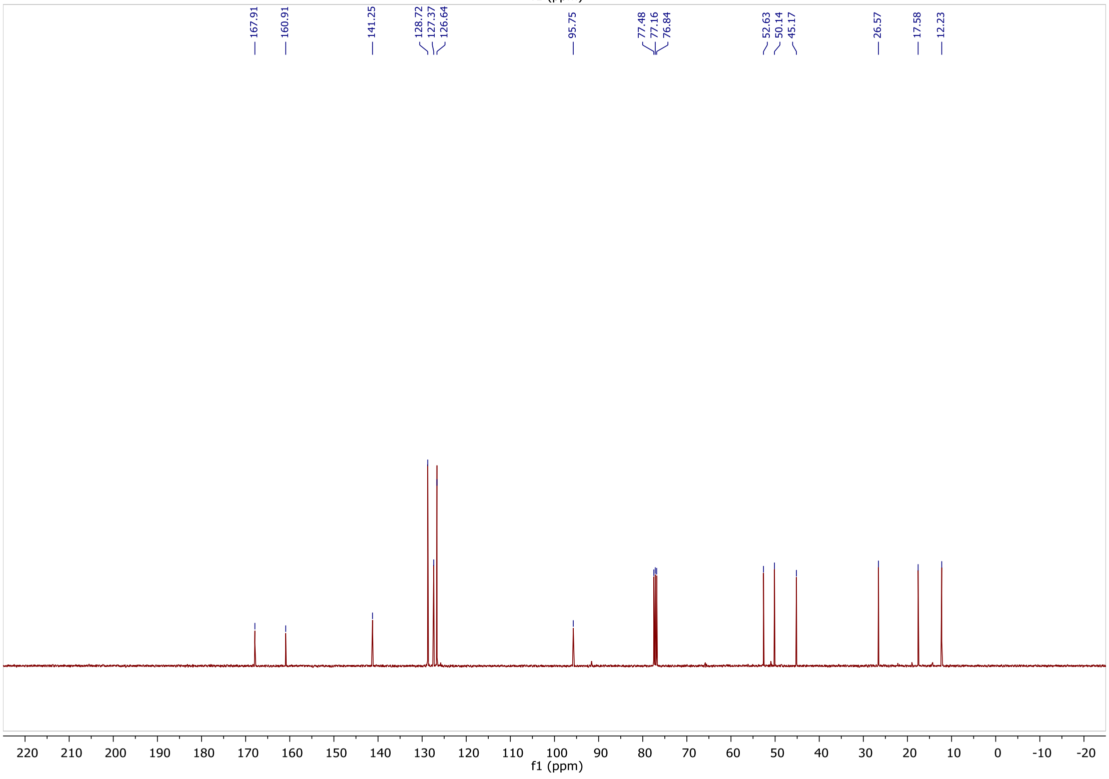

400 MHz ^{1}H NMR spectrum; 100.6 MHz ^{13}C NMR spectrum; $CDCl_3$

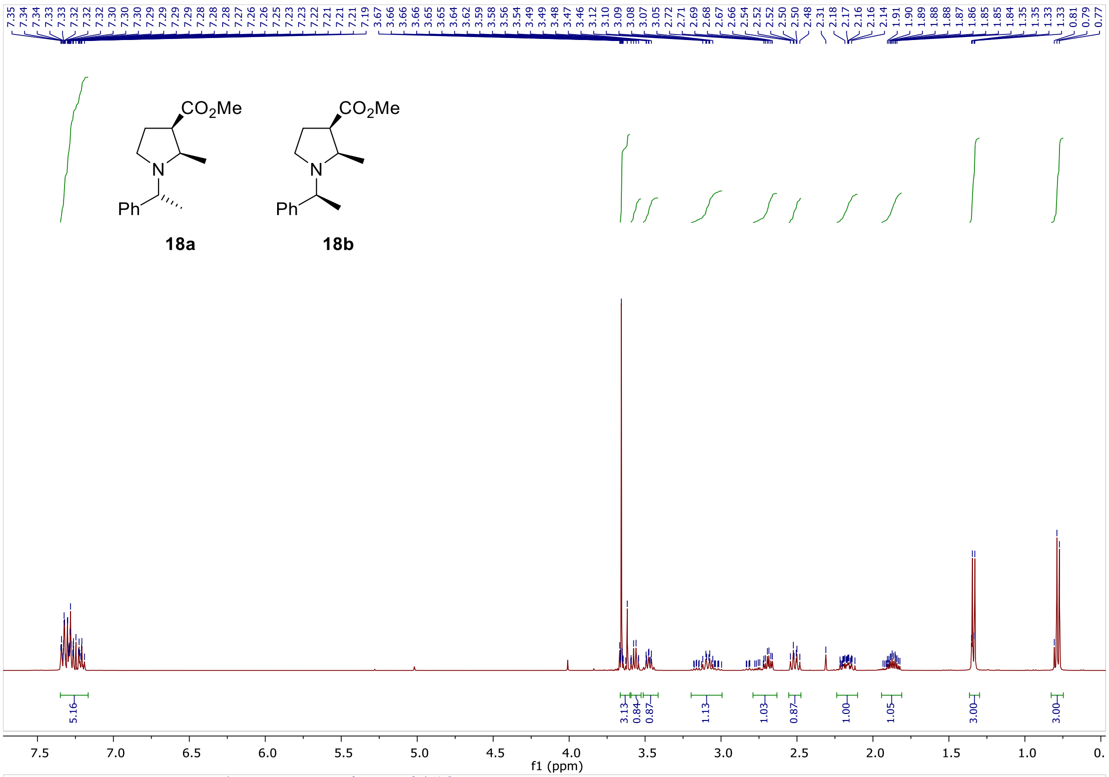

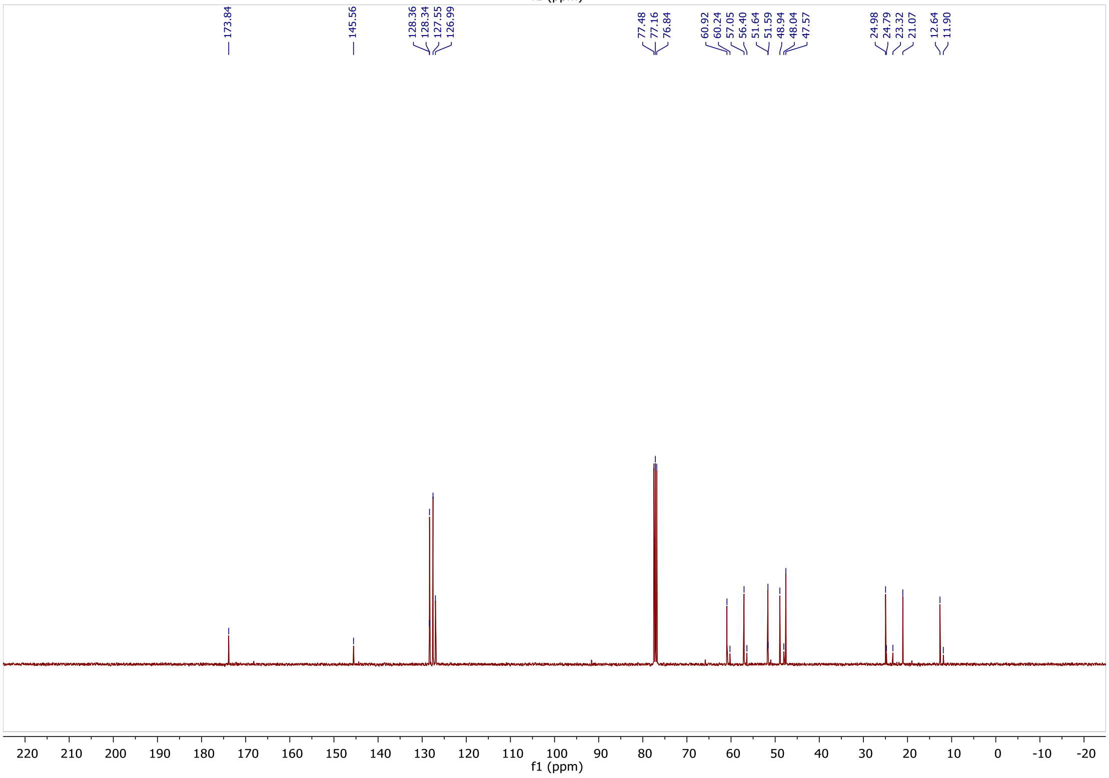

400 MHz ^{1}H NMR spectrum; 100.6 MHz ^{13}C NMR spectrum; $CDCl_3$

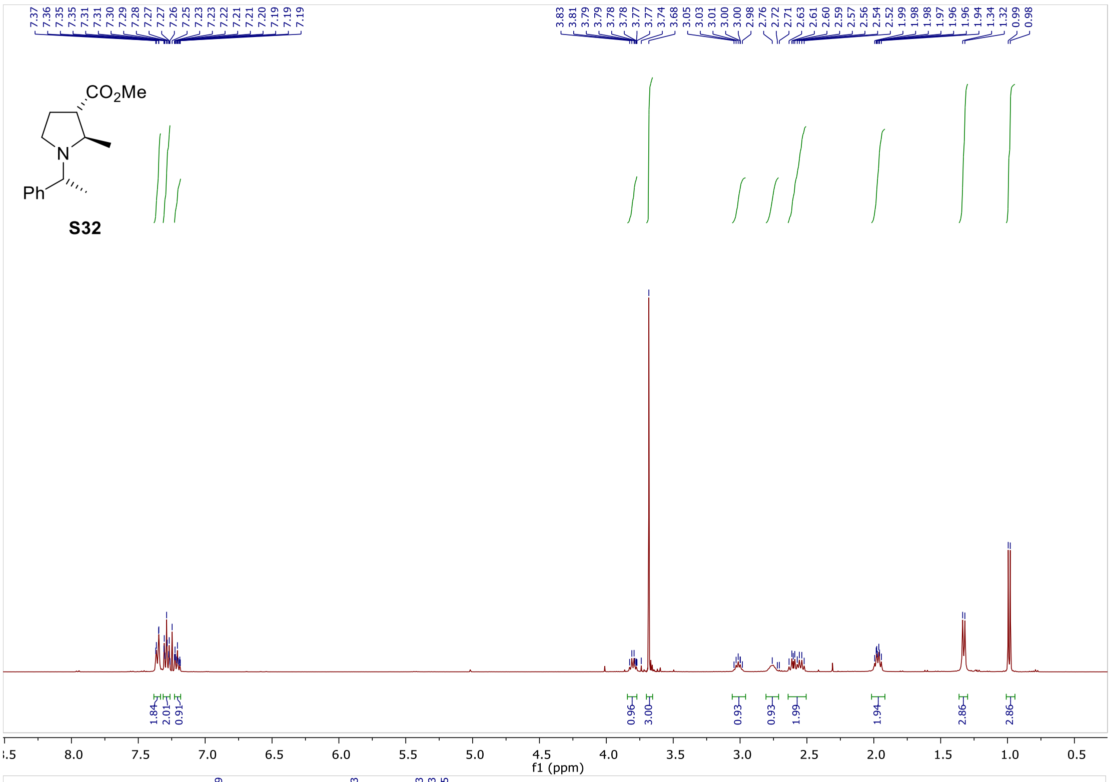

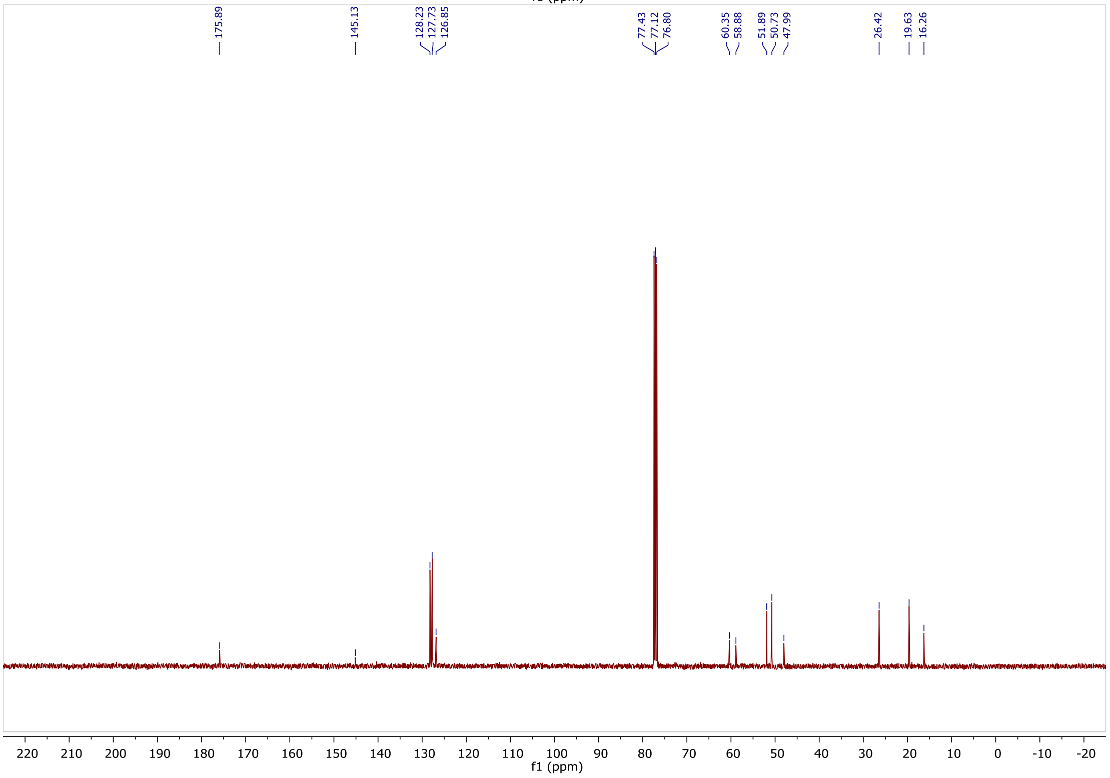

400 MHz ^{1}H NMR spectrum; 100.6 MHz ^{13}C NMR spectrum; d_4-MeOH

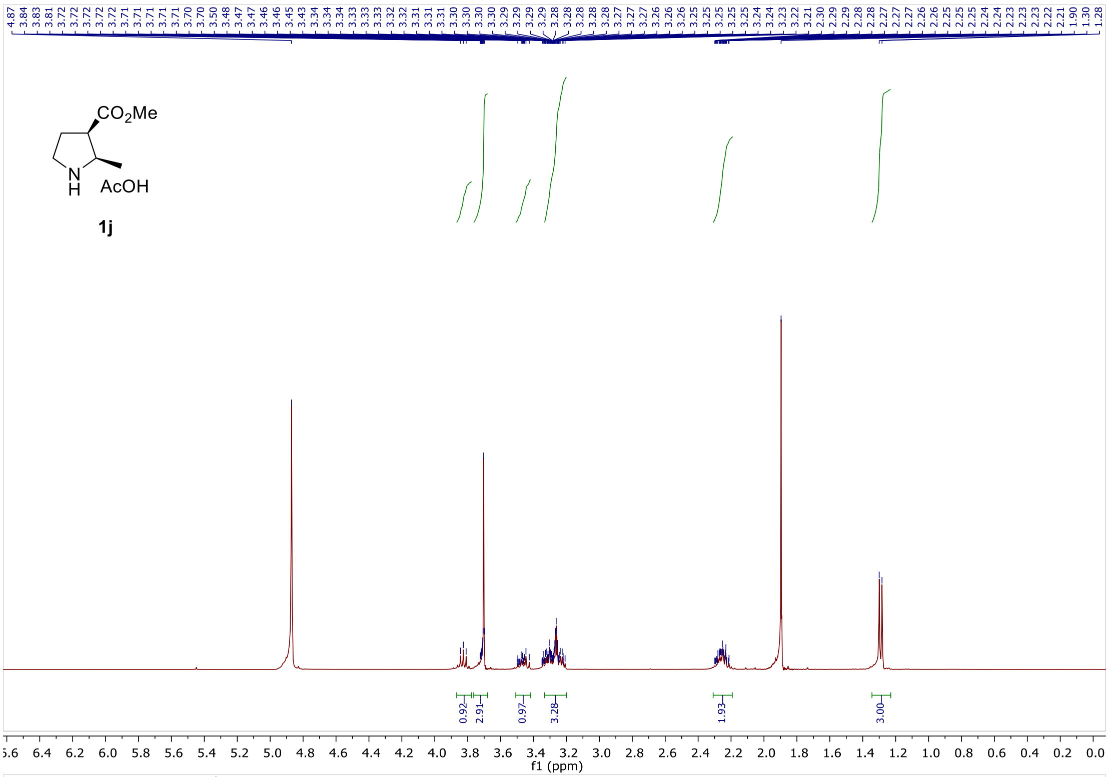

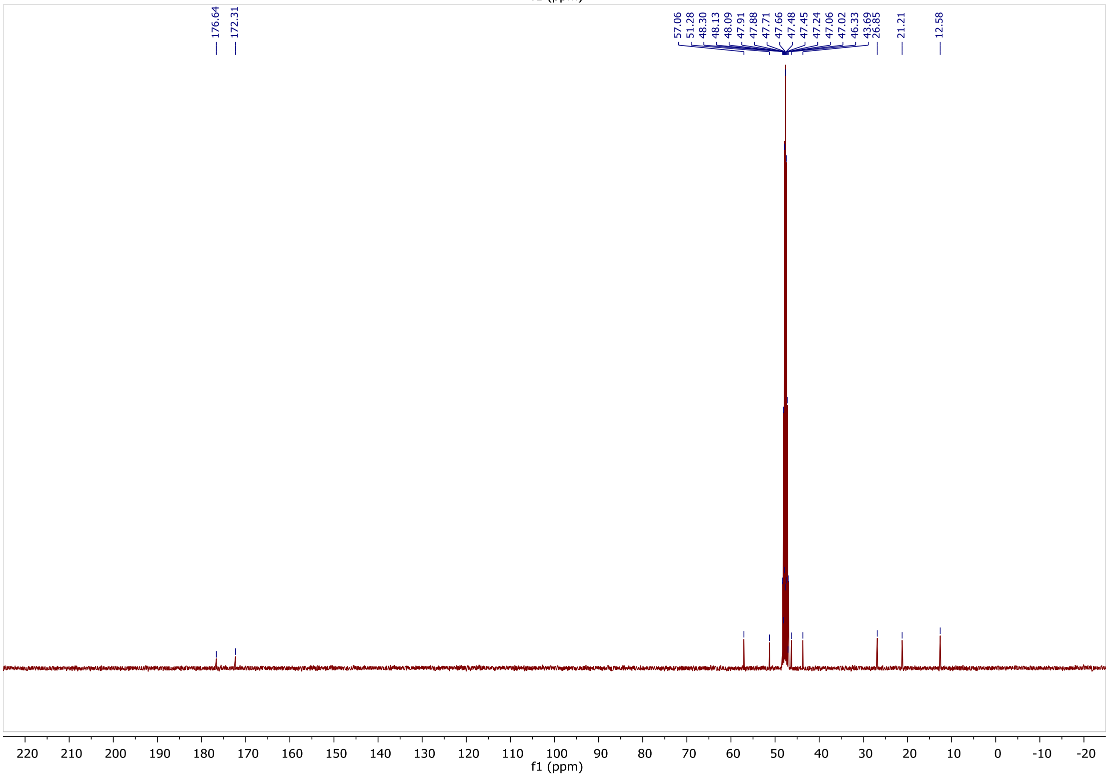

400 MHz ^{1}H NMR spectrum; 100.6 MHz ^{13}C NMR spectrum; d_4-MeOH

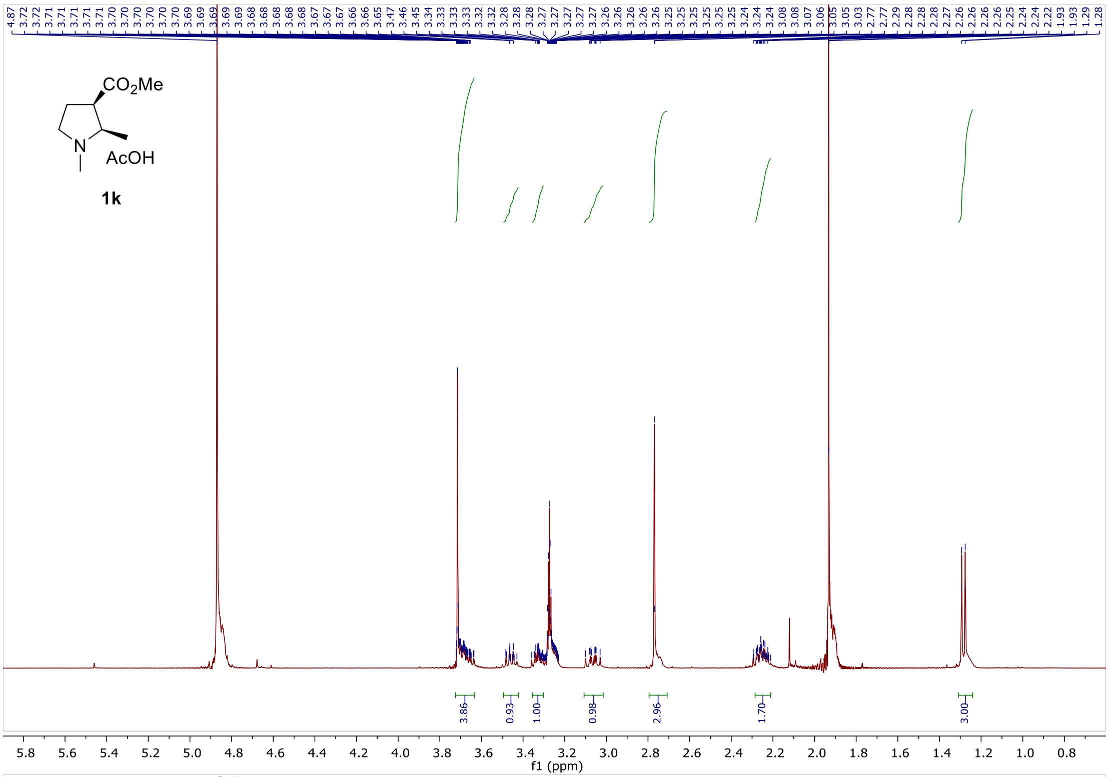

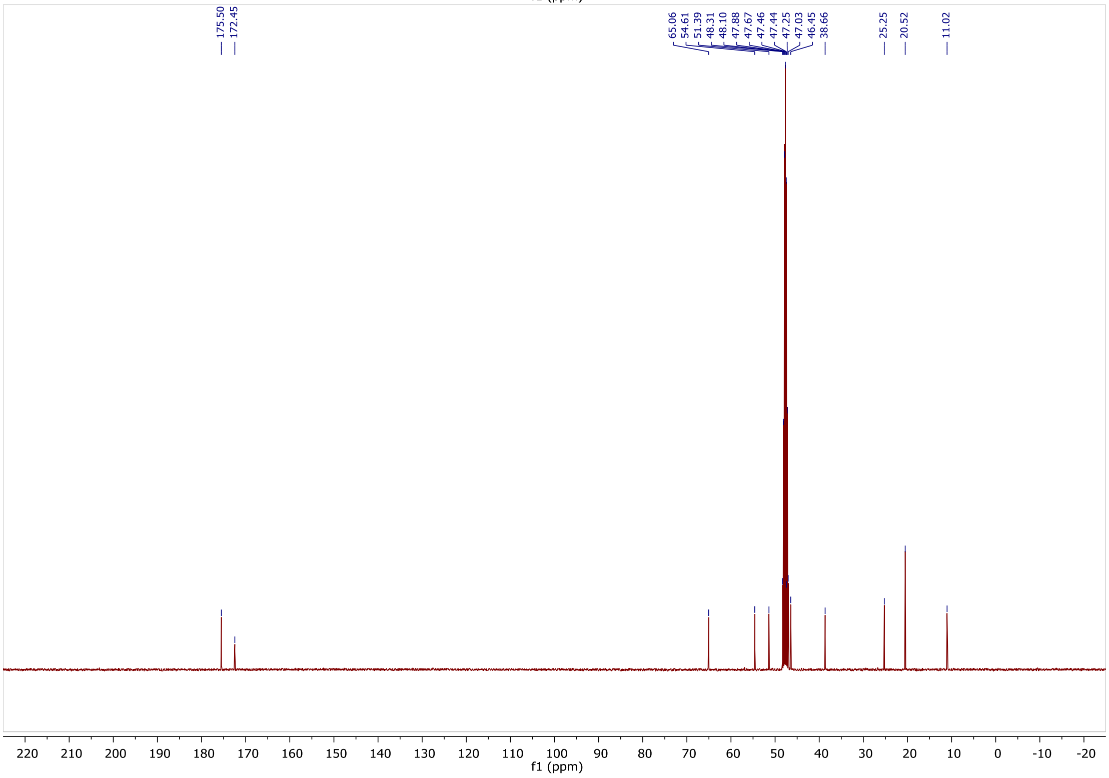

400 MHz ^{1}H NMR spectrum; 100.6 MHz ^{13}C NMR spectrum; $CDCl_3$

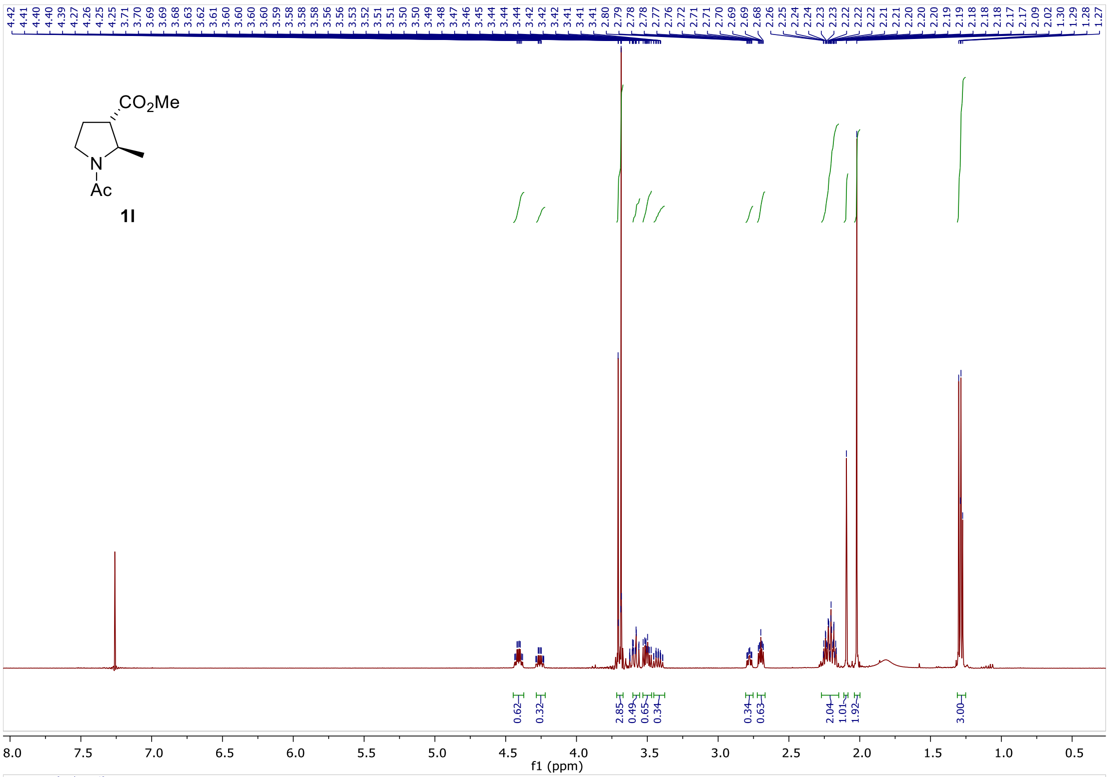

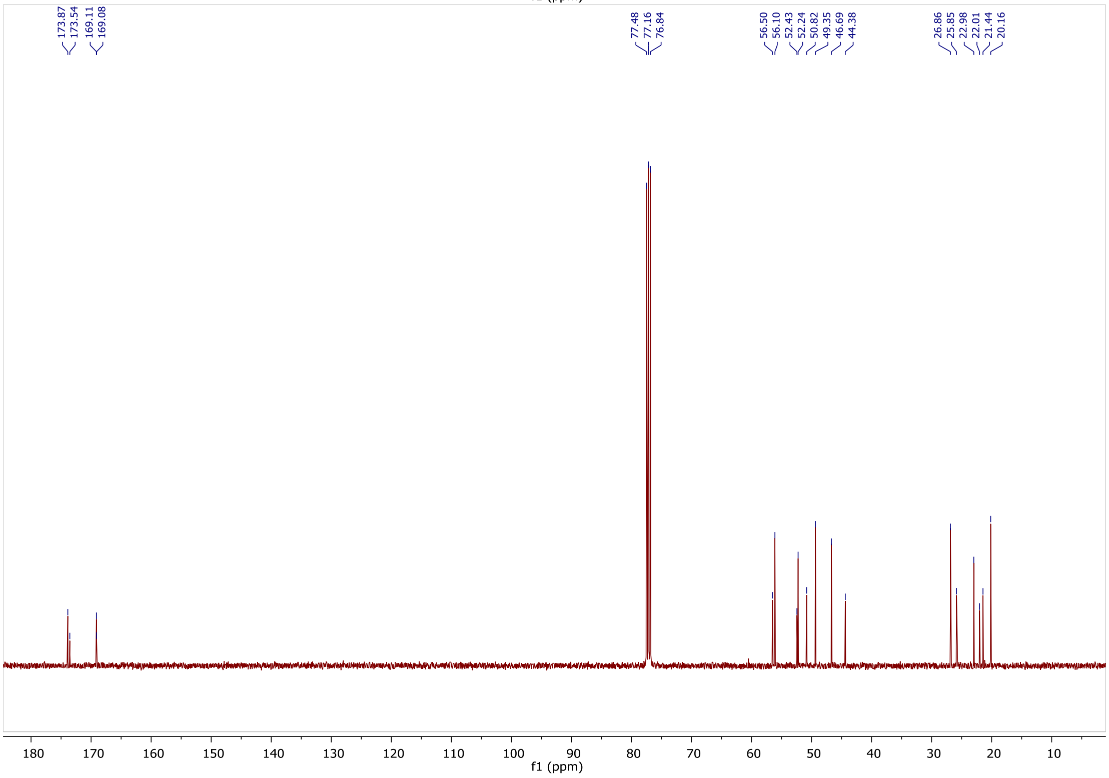

400 MHz ^{1}H NMR spectrum; 100.6 MHz ^{13}C NMR spectrum; $CDCl_3$

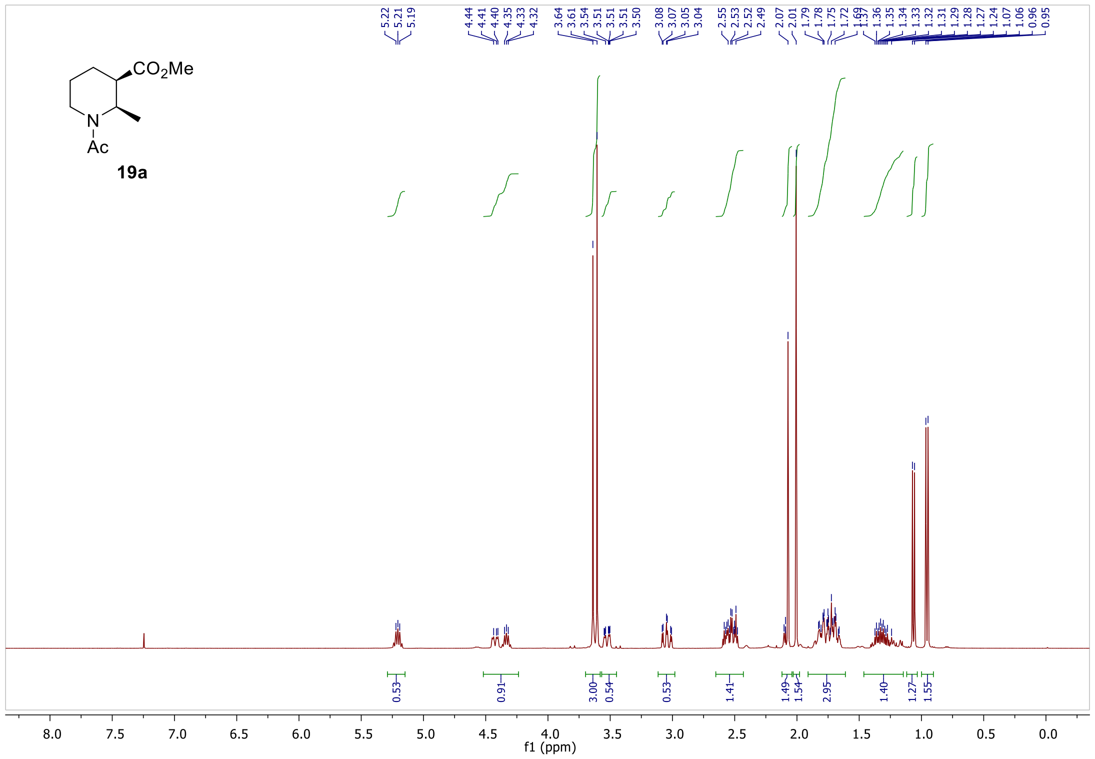

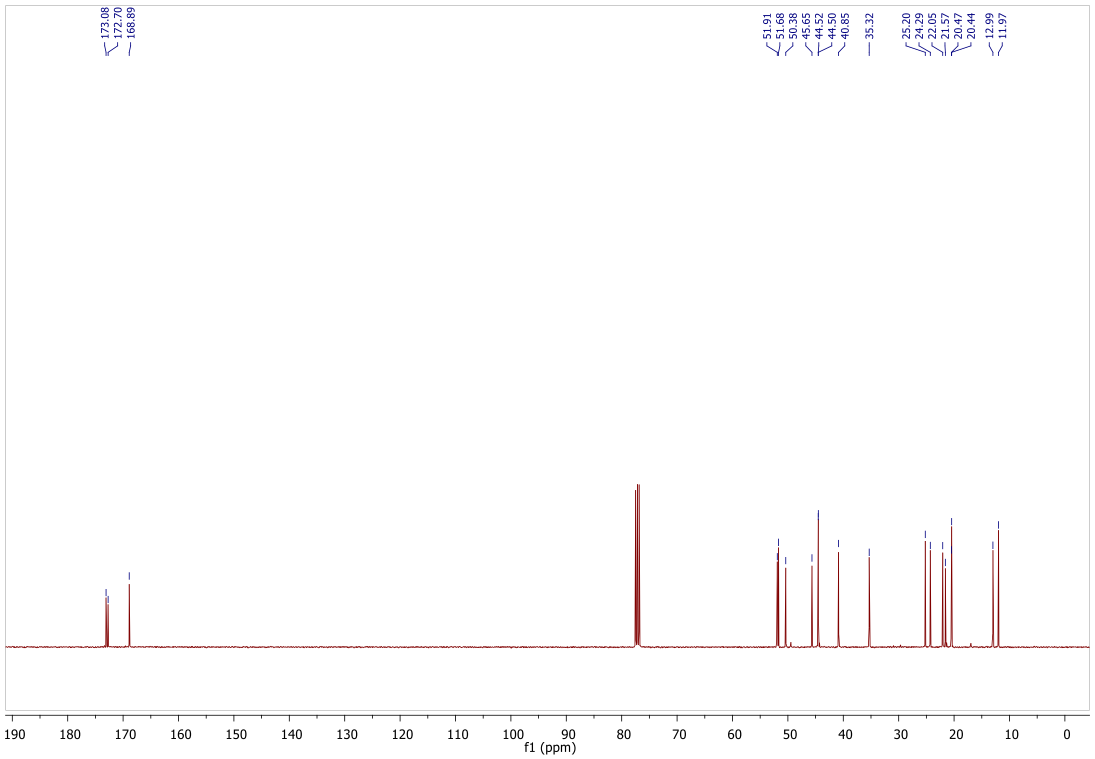

400 MHz ^{1}H NMR spectrum; 100.6 MHz ^{13}C NMR spectrum; $CDCl_3$

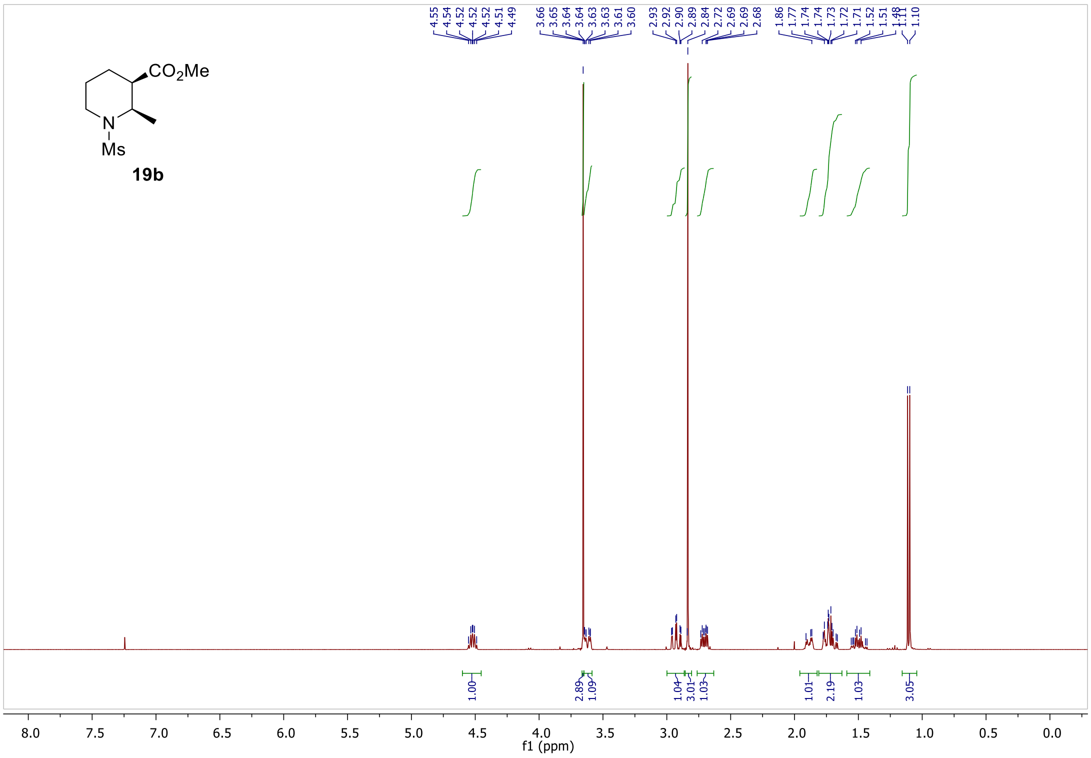

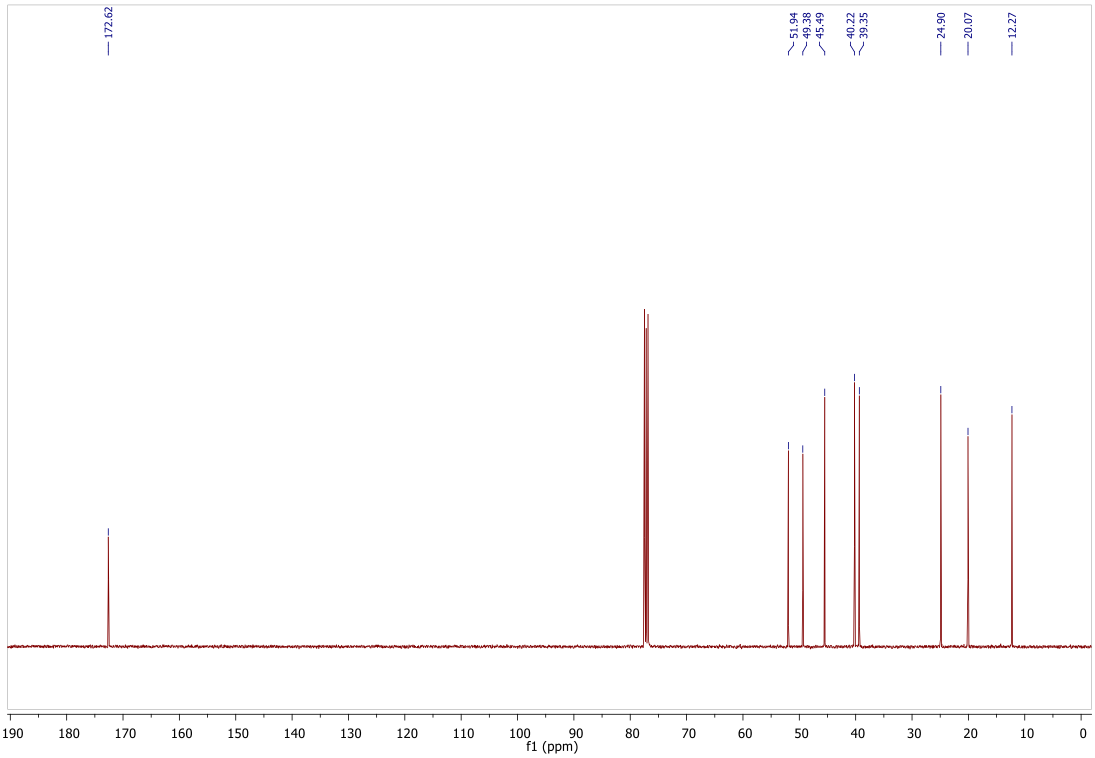

400 MHz ^{1}H NMR spectrum; 100.6 MHz ^{13}C NMR spectrum; $CDCl_3$

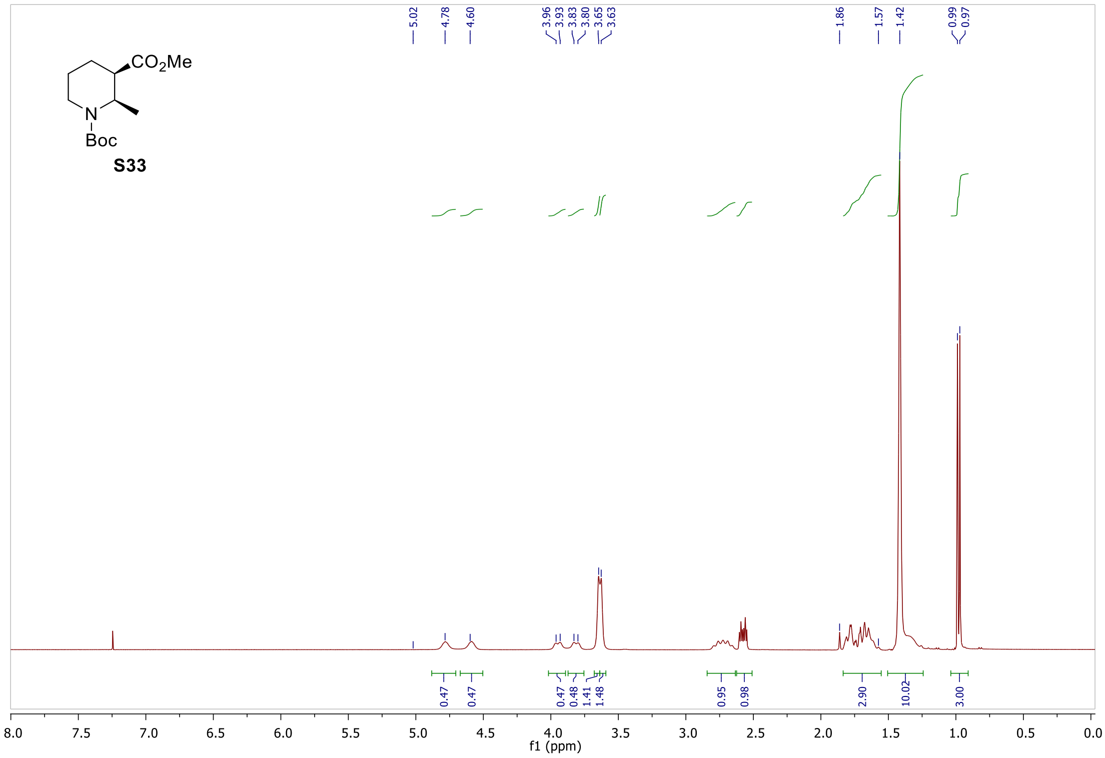

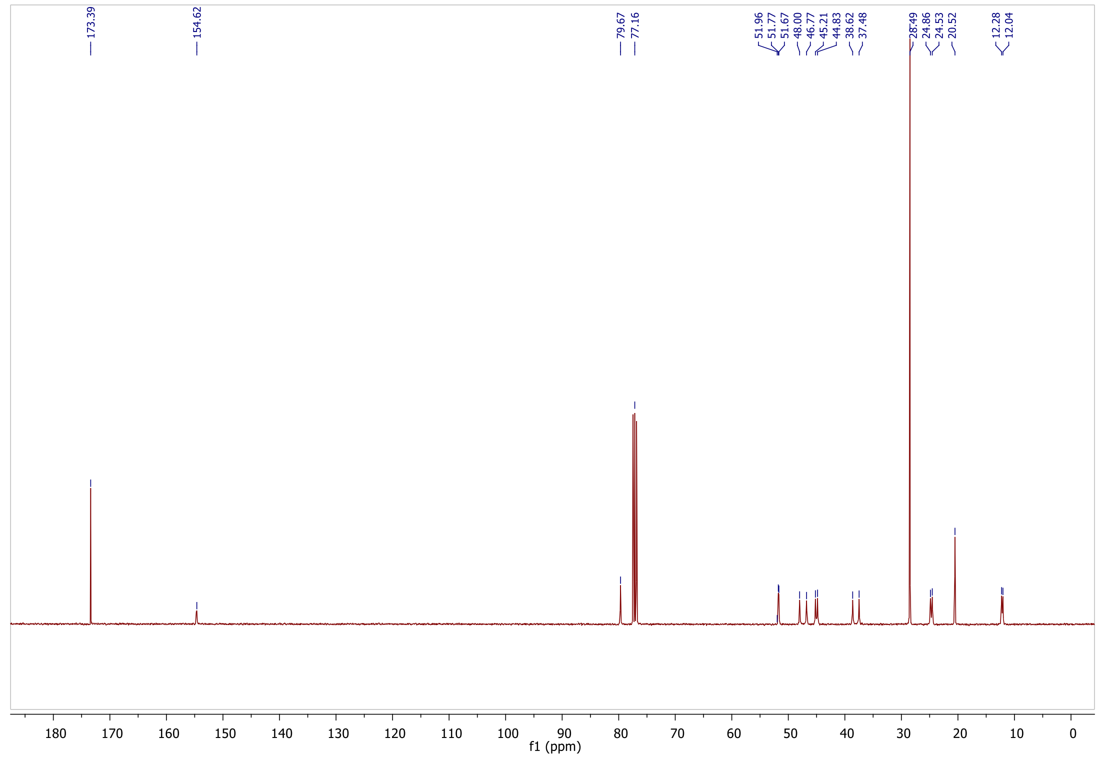

400 MHz ^{1}H NMR spectrum; 100.6 MHz ^{13}C NMR spectrum; $CDCl_3$

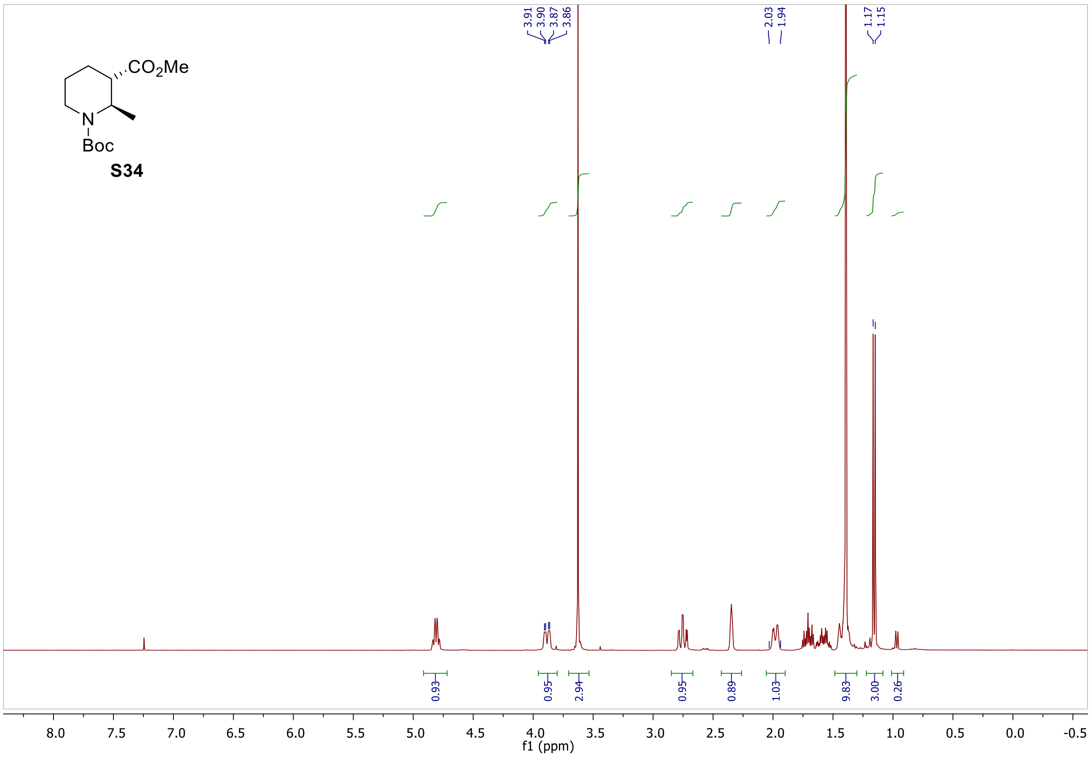

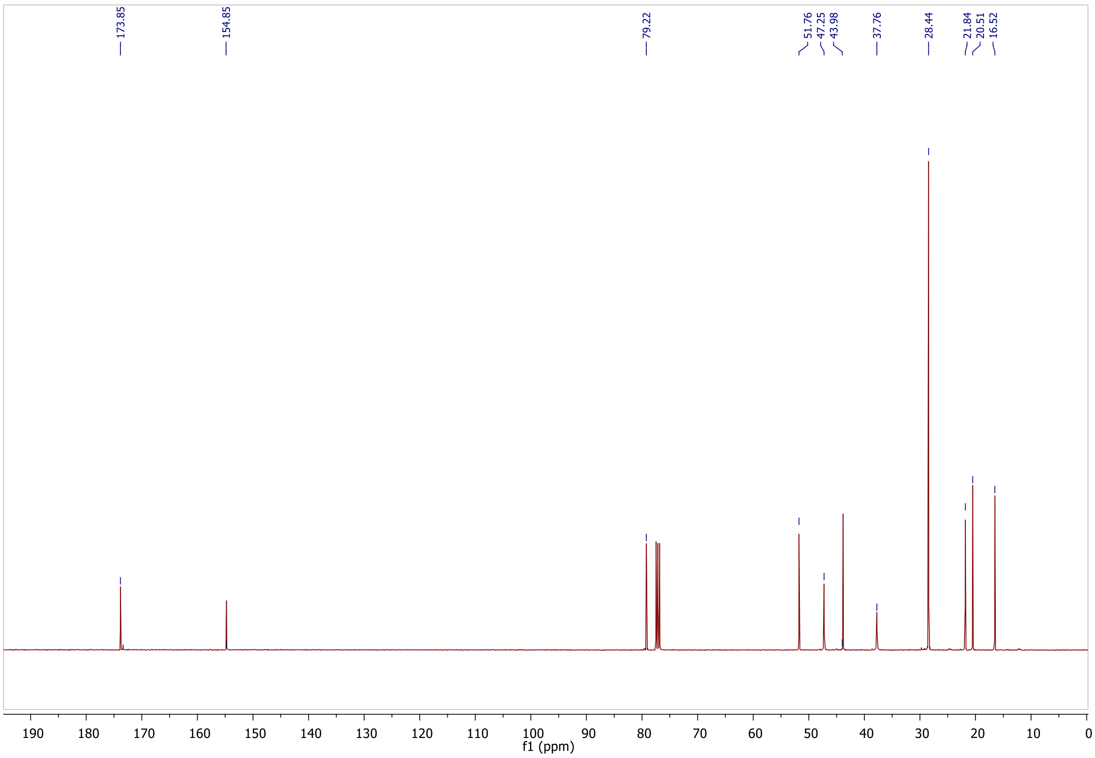

400 MHz ^1H NMR spectrum; 100.6 MHz ^{13}C NMR spectrum; $CDCl_3$

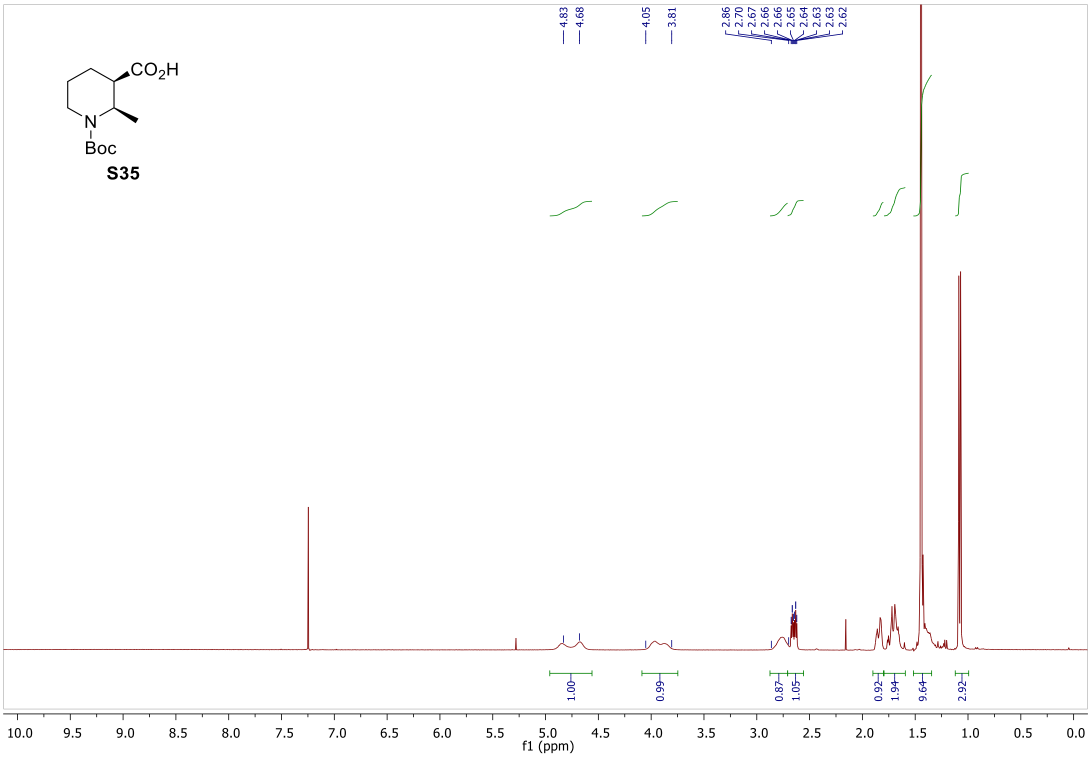

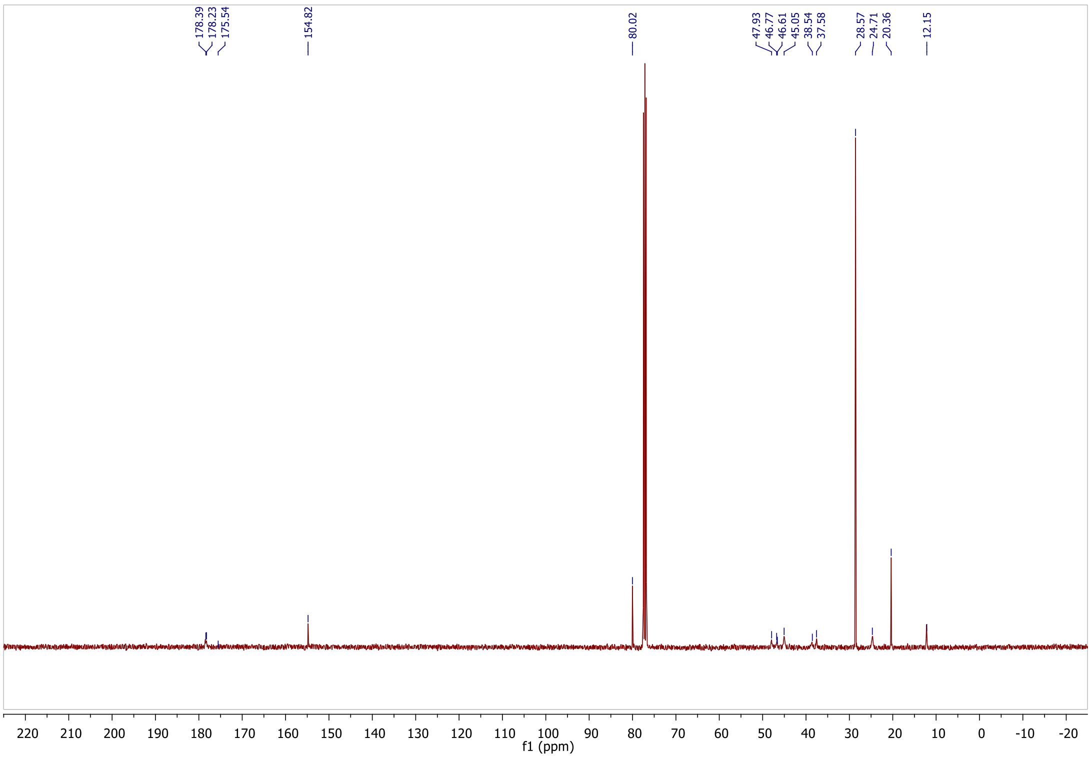

400 MHz ^{1}H NMR spectrum; 100.6 MHz ^{13}C NMR spectrum; $CDCl_3$

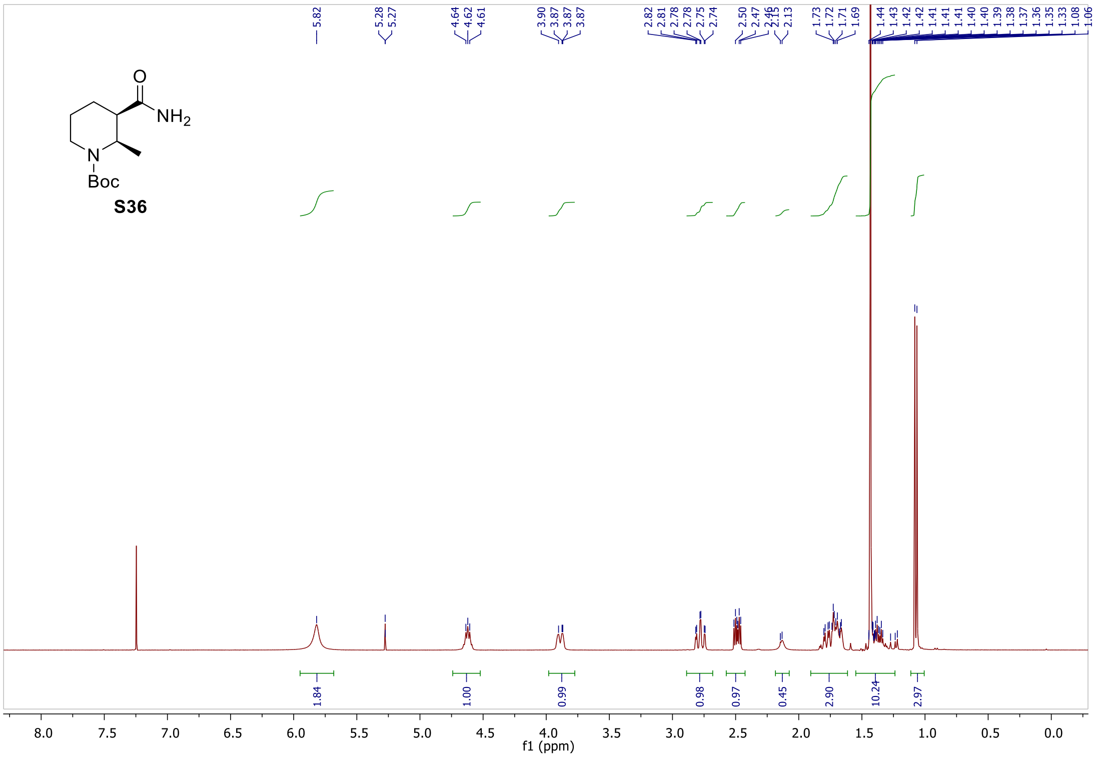

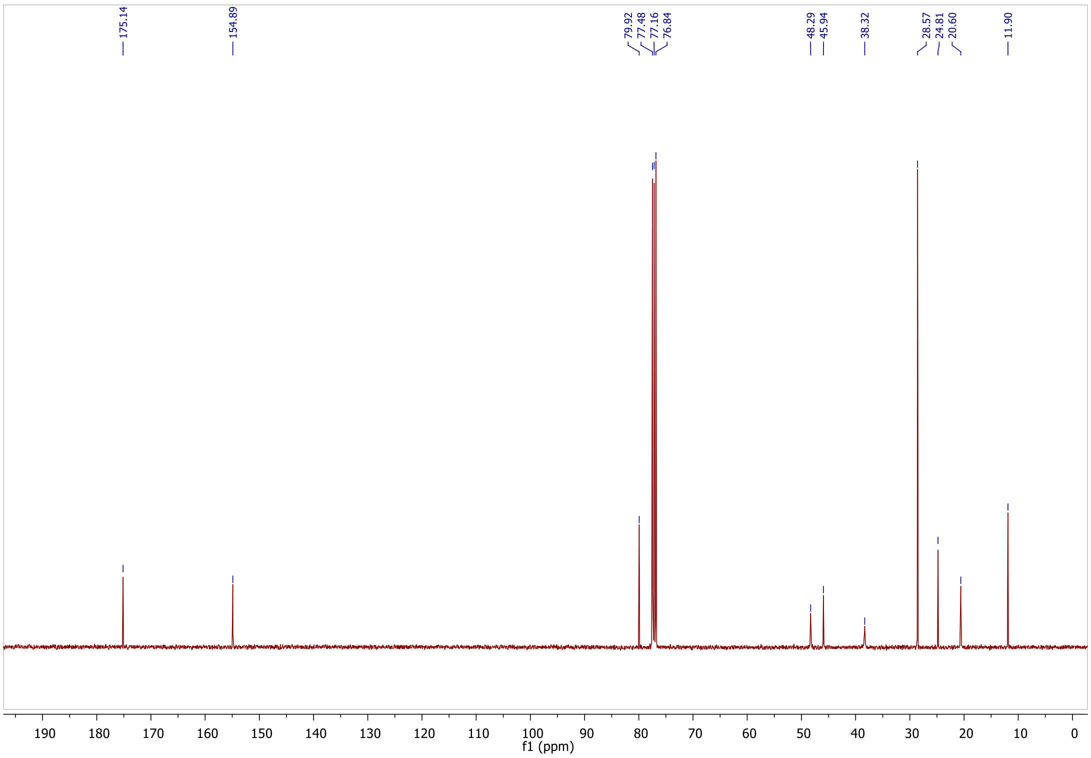

400 MHz ^{1}H NMR spectrum; 100.6 MHz ^{13}C NMR spectrum; $CDCl_3$

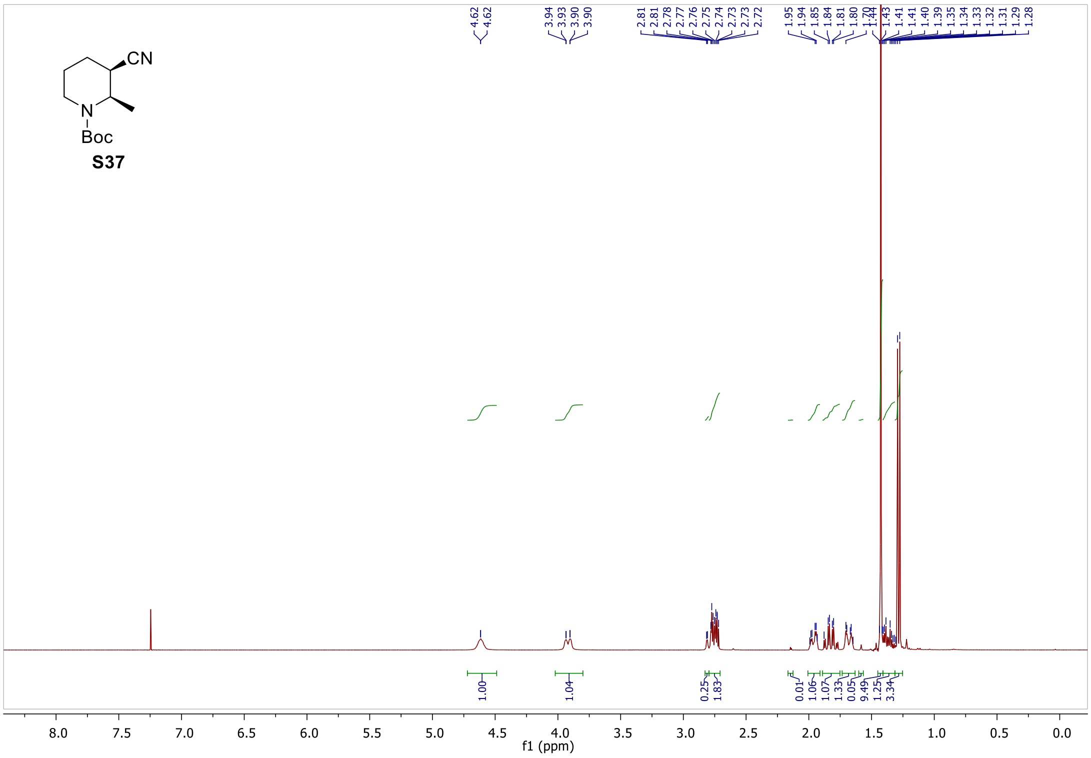

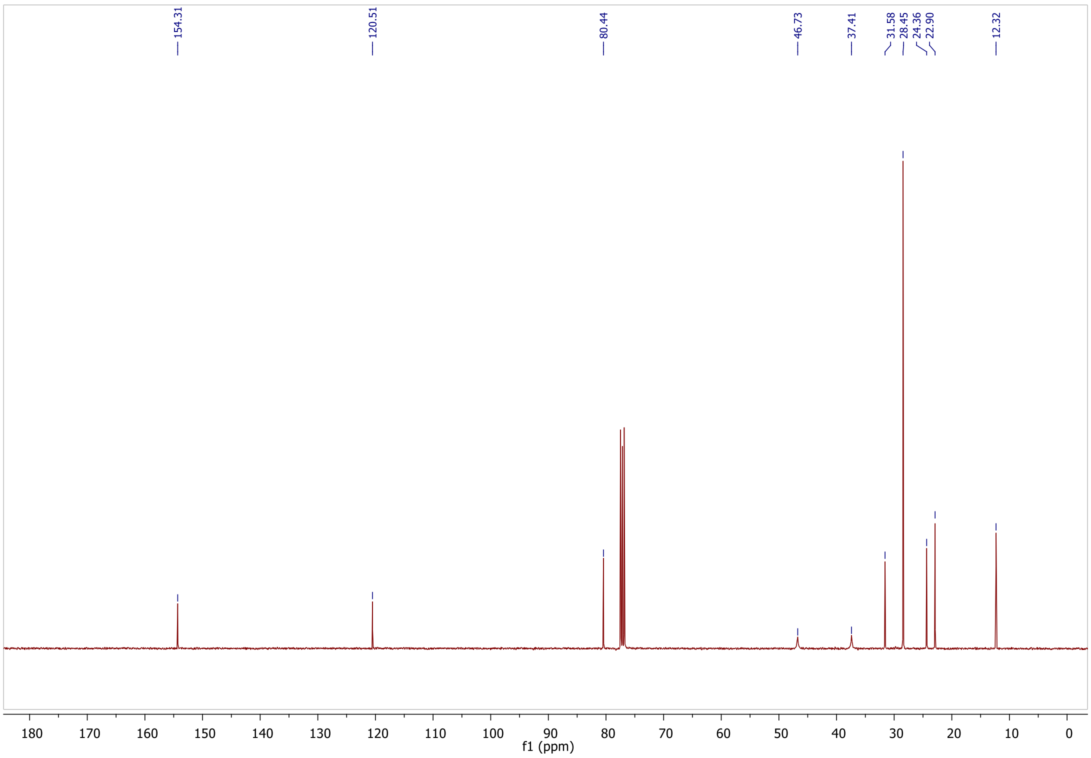

400 MHz ^{1}H NMR spectrum; 100.6 MHz ^{13}C NMR spectrum; MeOH-d_4

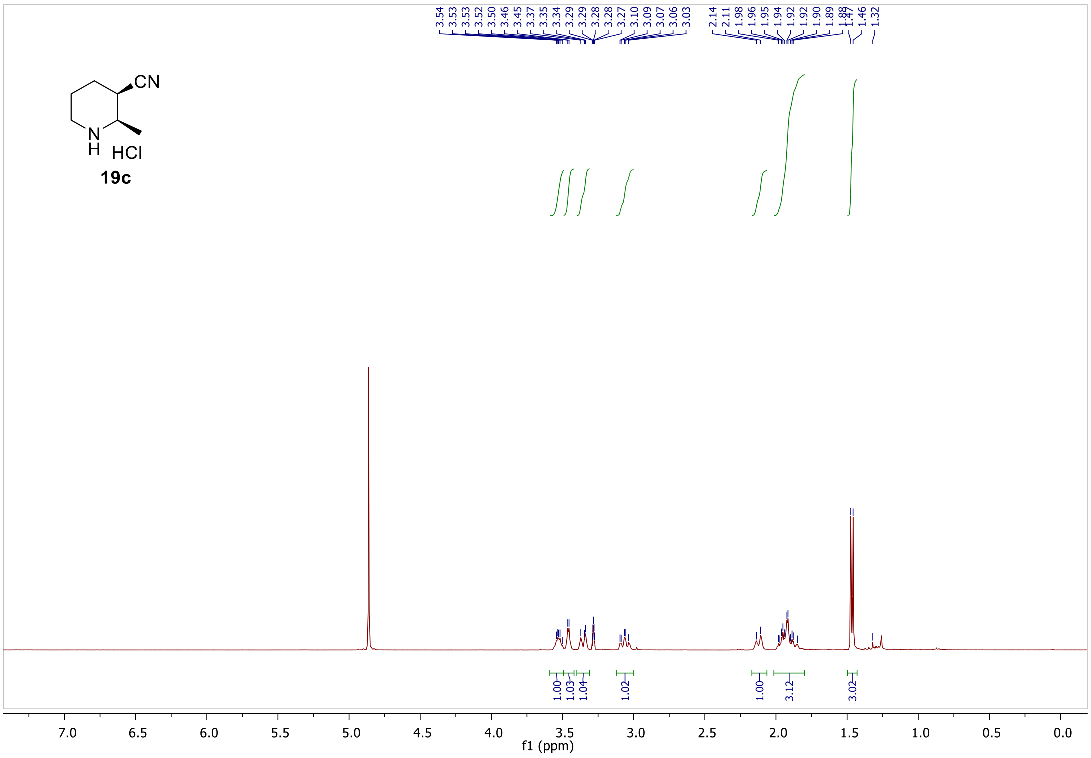

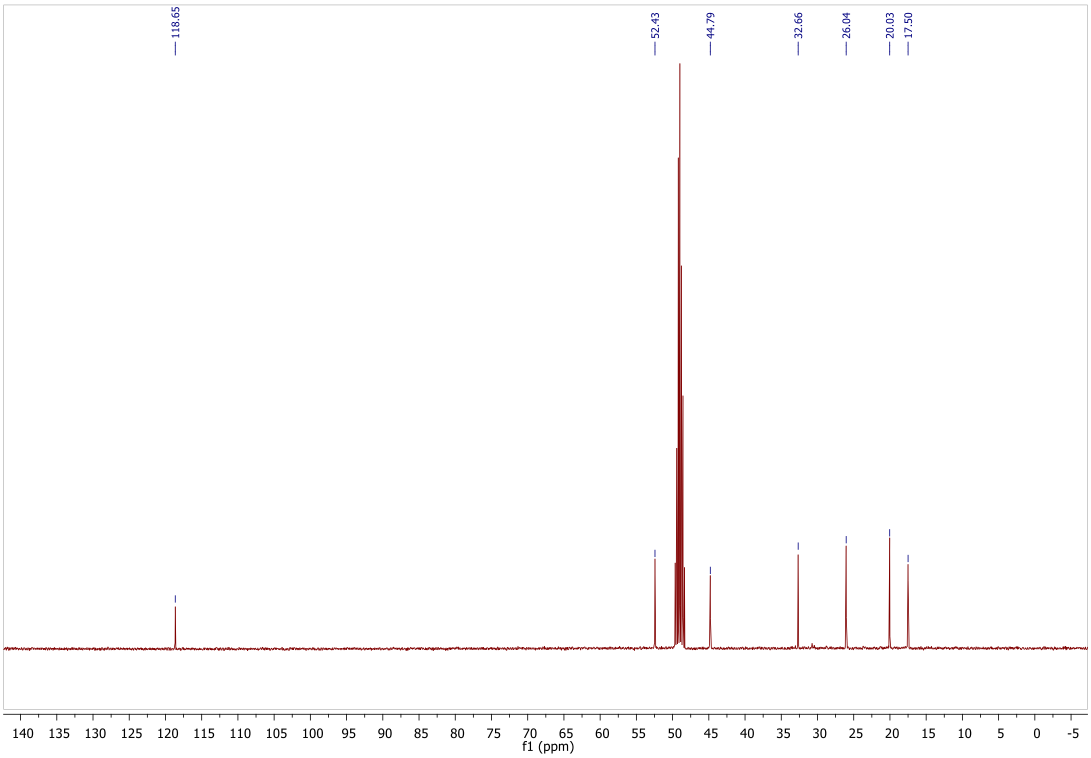

400 MHz ^{1}H NMR spectrum; 100.6 MHz ^{13}C NMR spectrum; $CDCl_3$

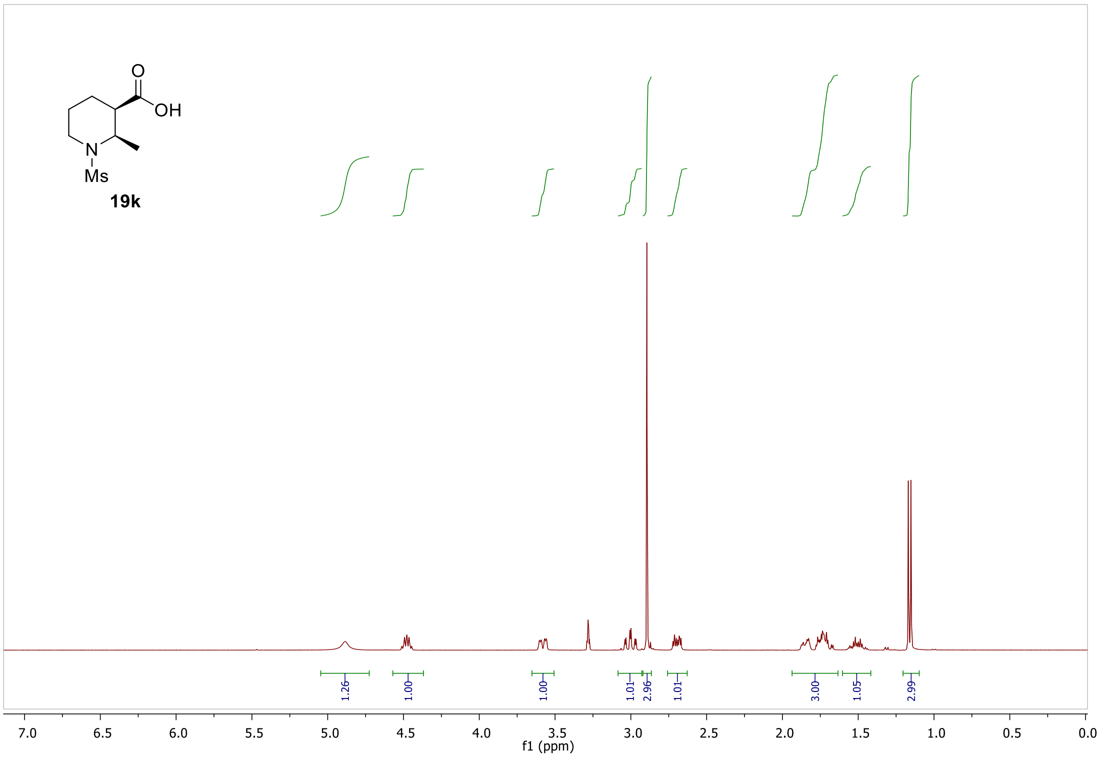

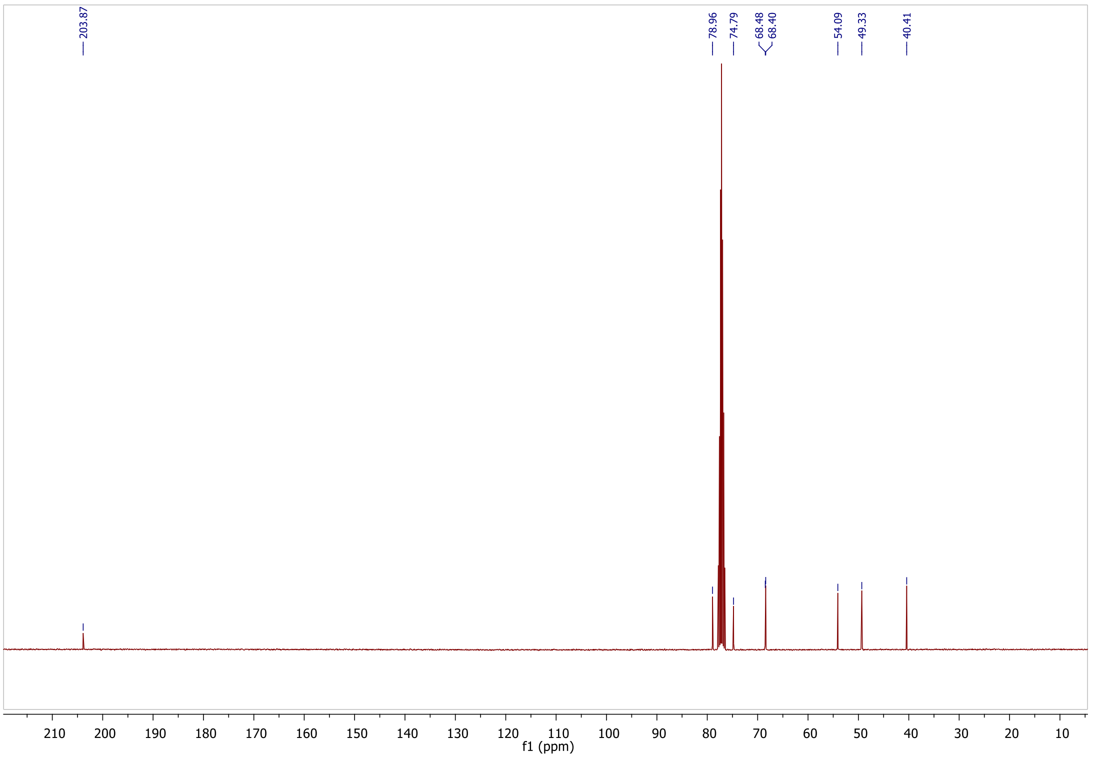

400 MHz ^{1}H NMR spectrum; 100.6 MHz ^{13}C NMR spectrum; MeOH-d_4

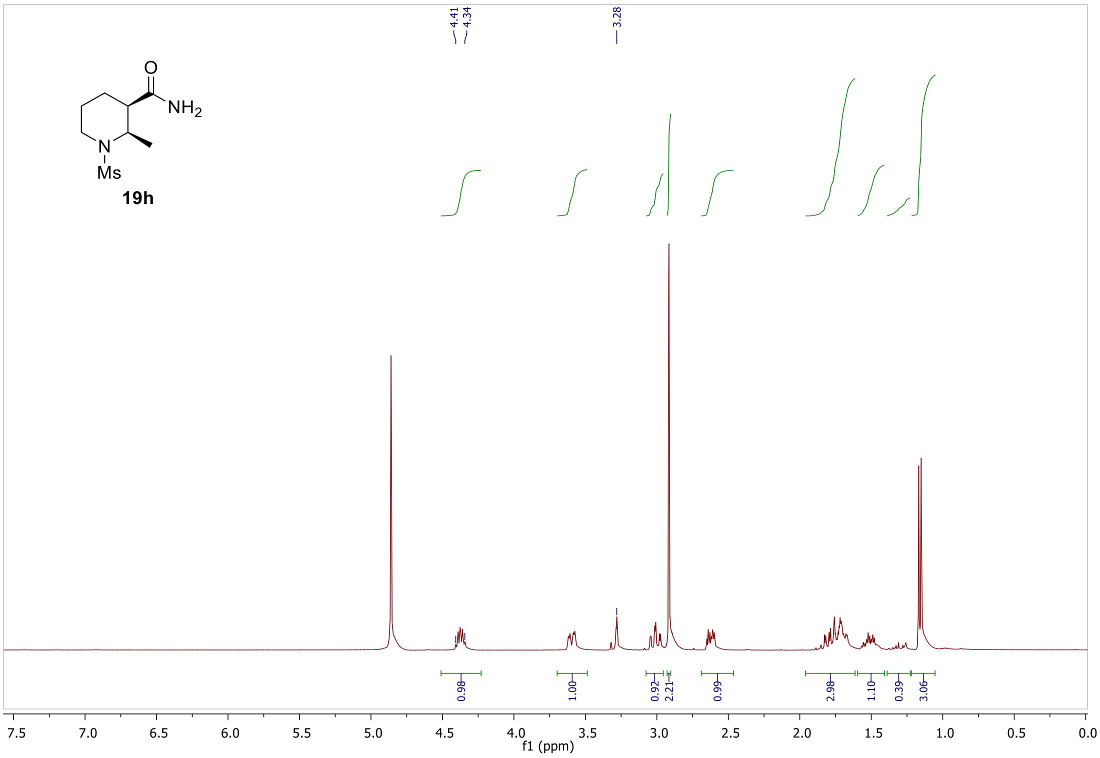

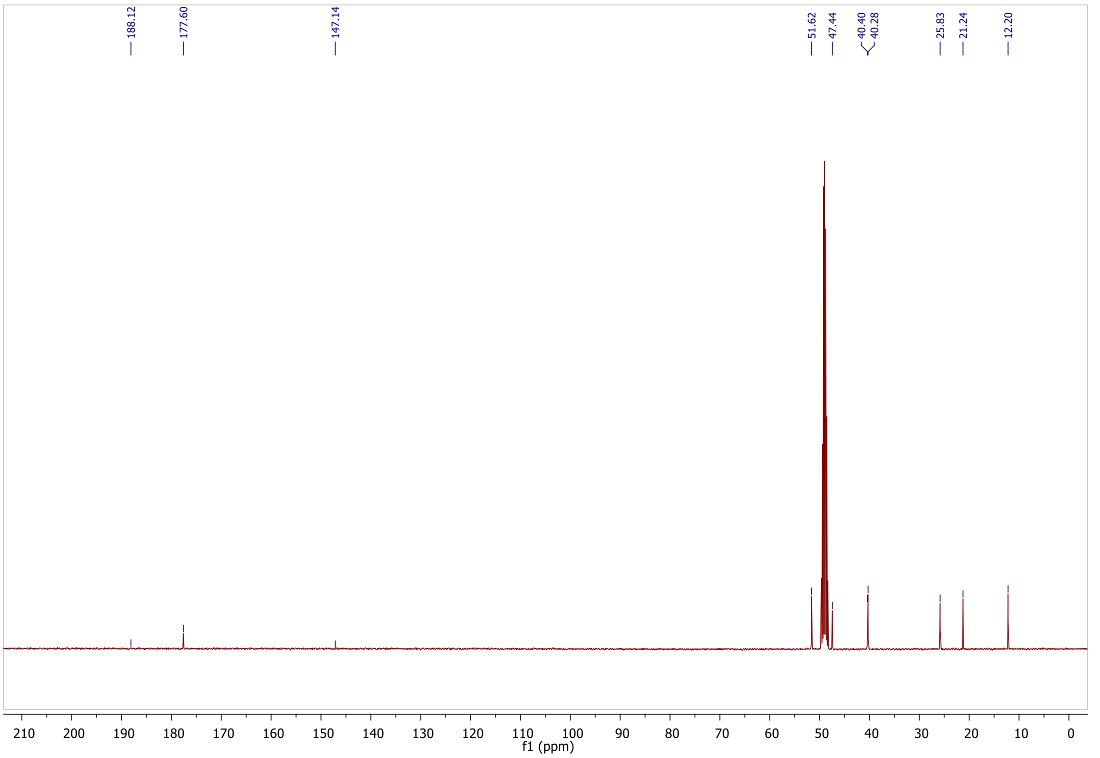

400 MHz ^{1}H NMR spectrum; 100.6 MHz ^{13}C NMR spectrum; $CDCl_3$

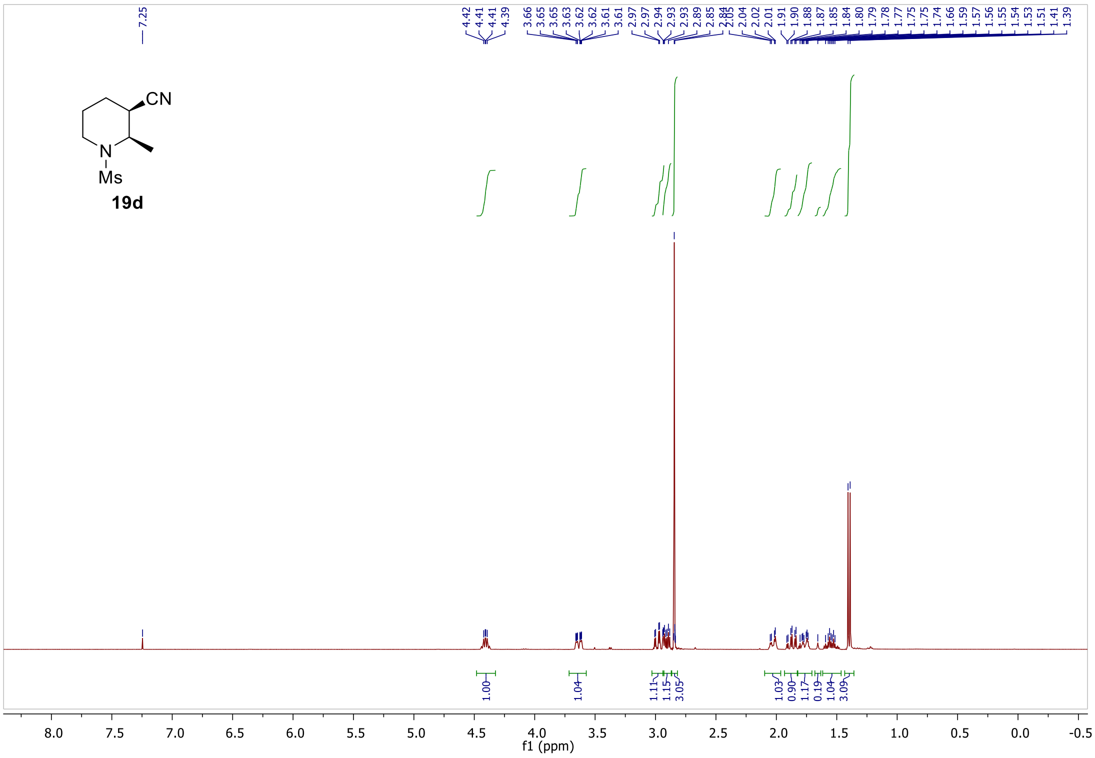

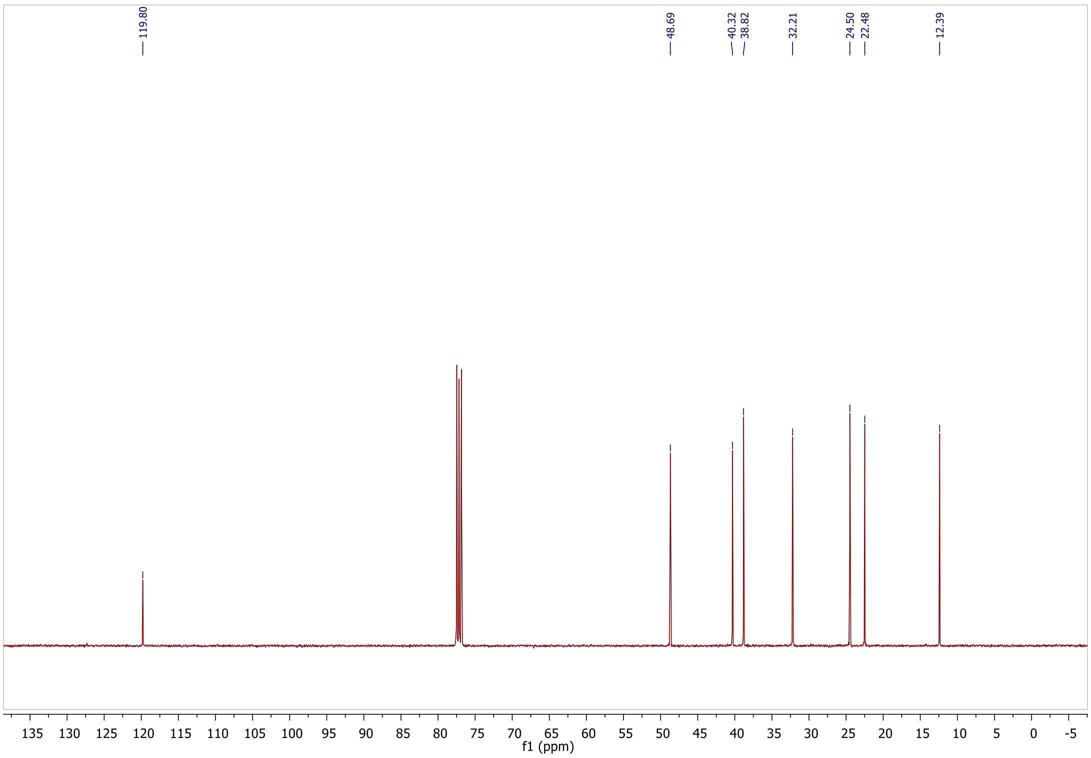

400 MHz ^{1}H NMR spectrum; 100.6 MHz ^{13}C NMR spectrum; $CDCl_3$

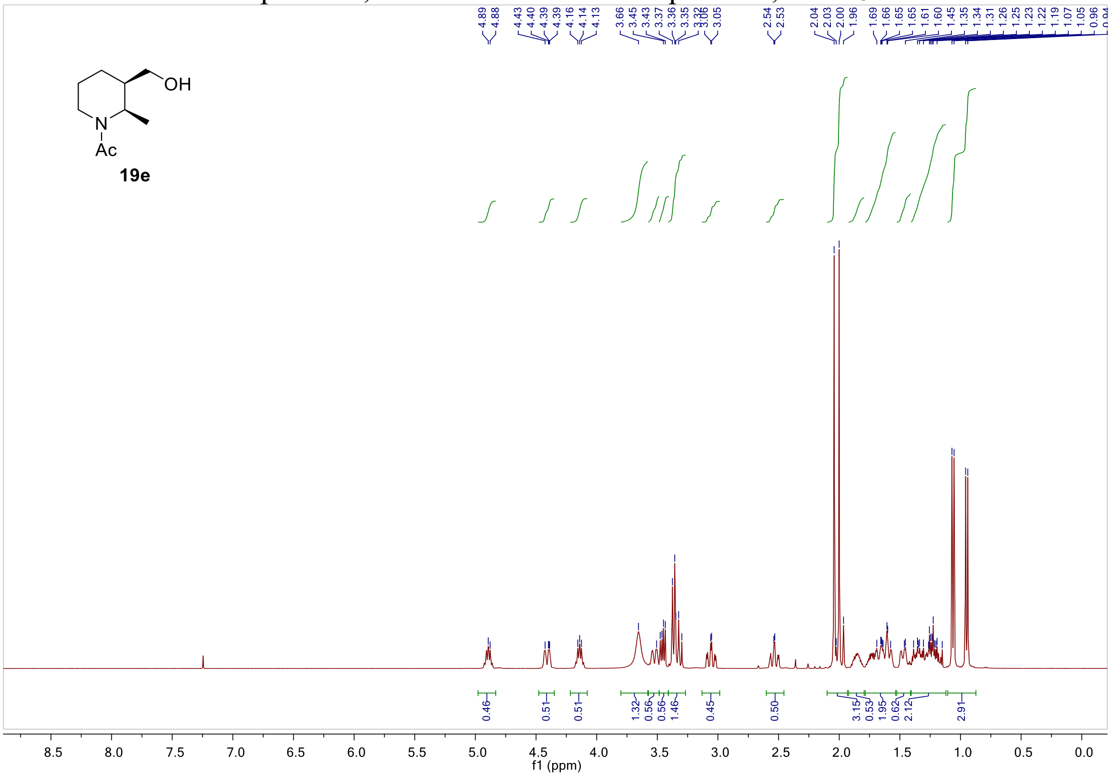

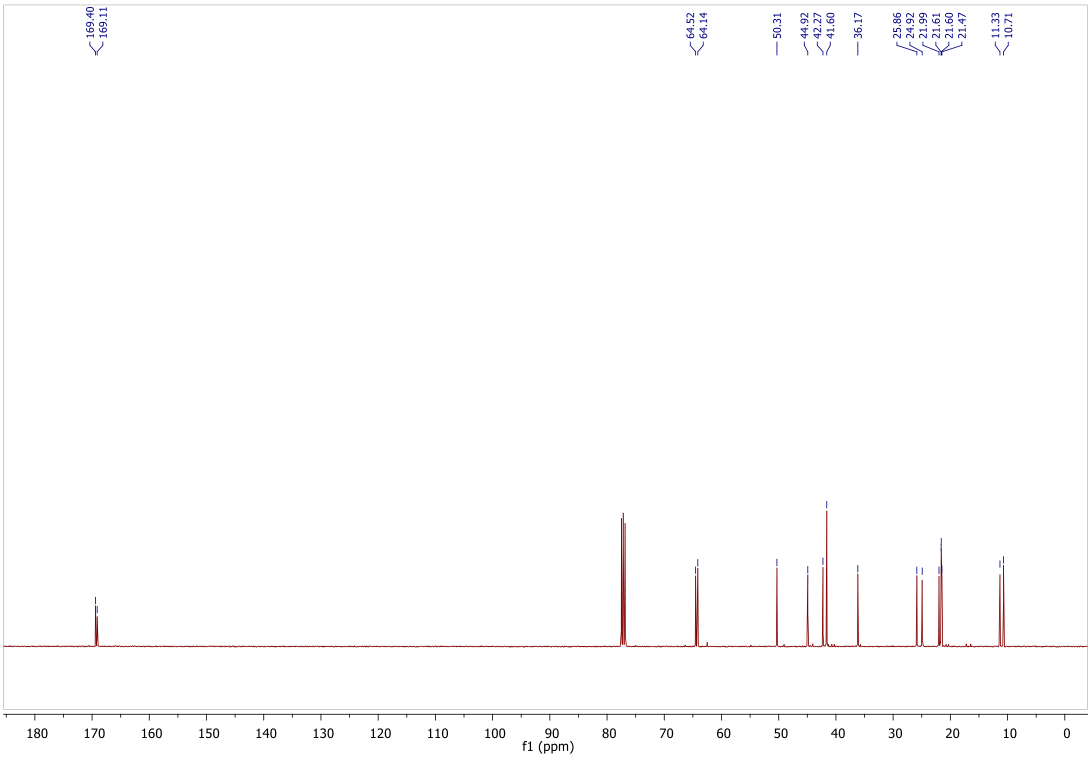

400 MHz ^{1}H NMR spectrum; 100.6 MHz ^{13}C NMR spectrum; $CDCl_3$

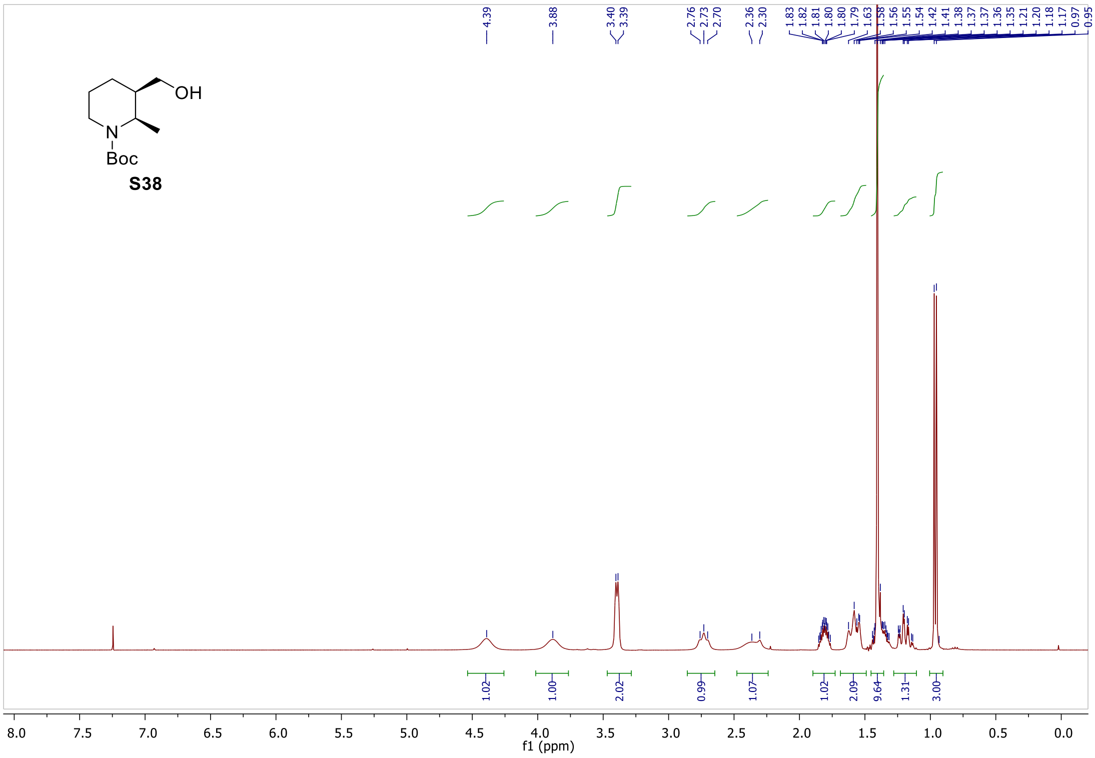

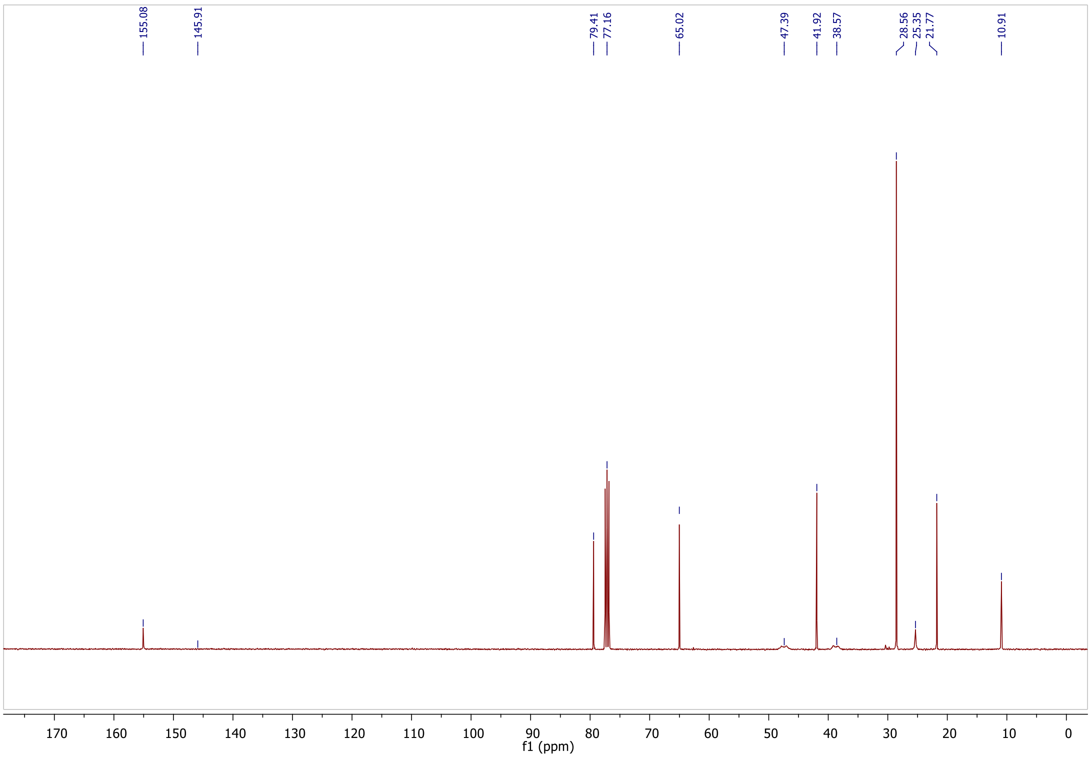

400 MHz ^{1}H NMR spectrum; 100.6 MHz ^{13}C NMR spectrum; $CDCl_3$

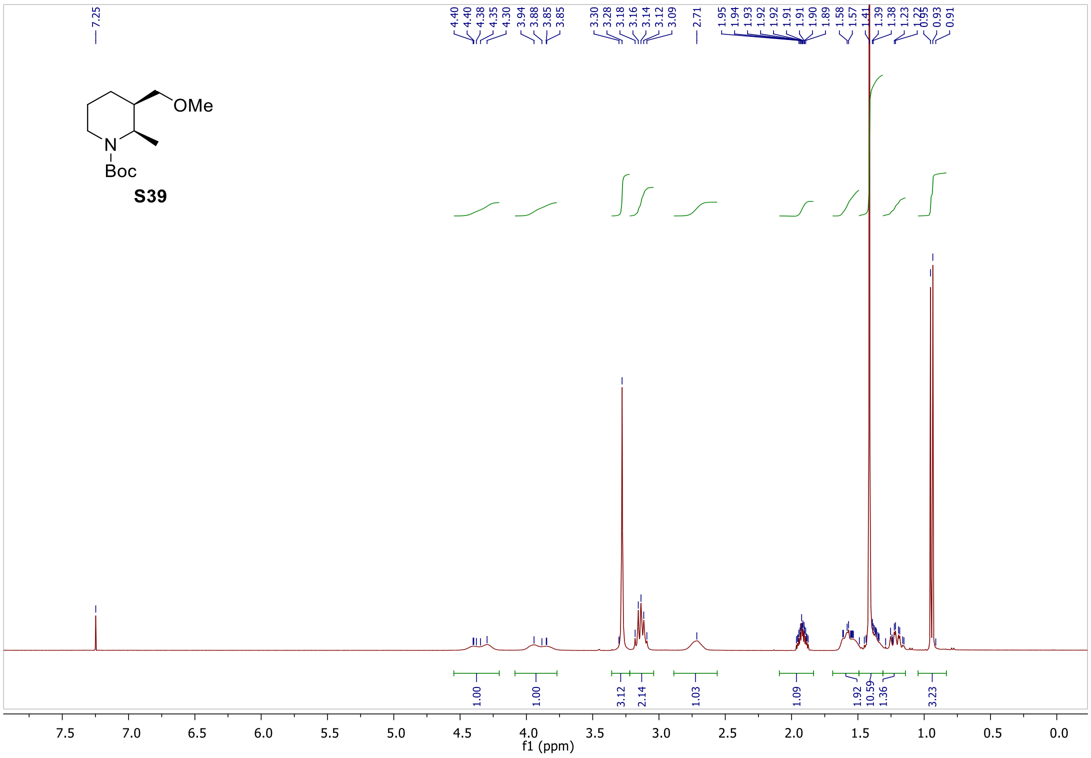

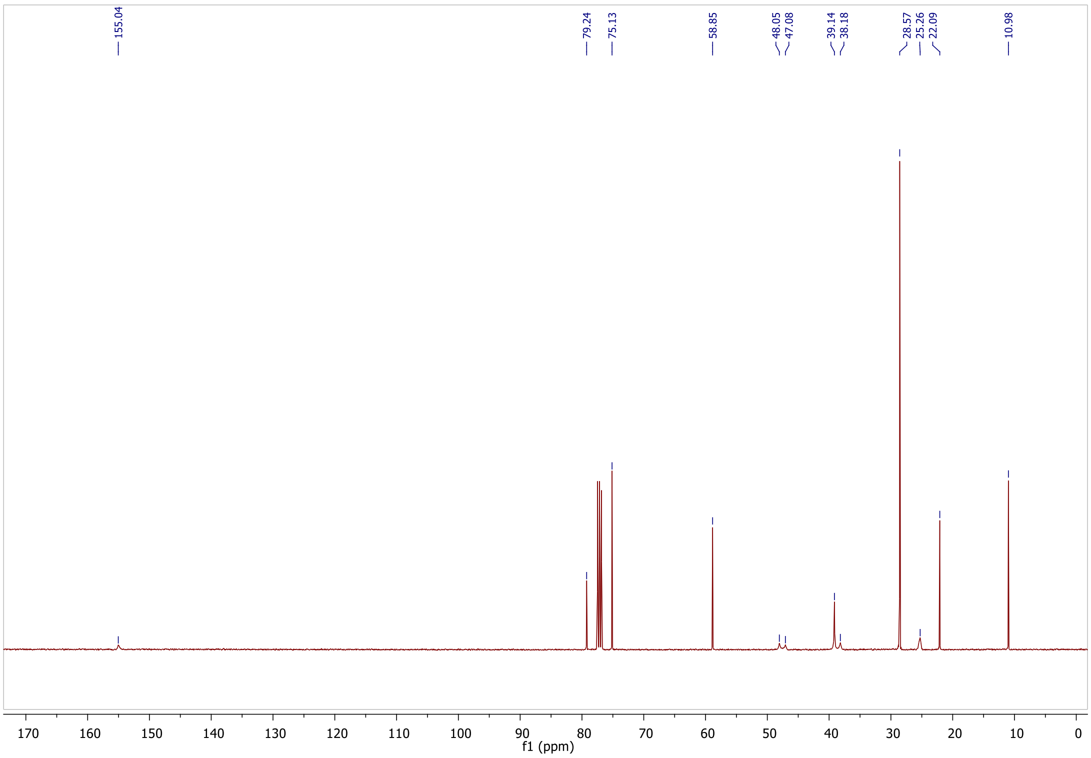

400 MHz ^{1}H NMR spectrum; 100.6 MHz ^{13}C NMR spectrum; $CDCl_3$

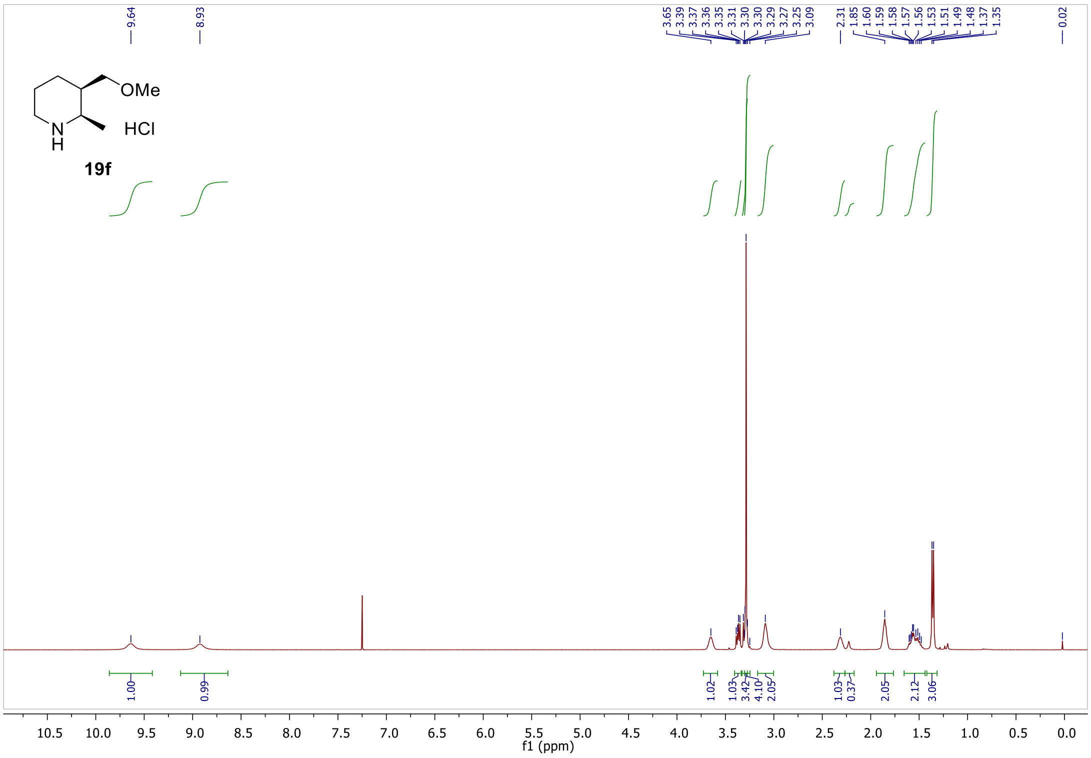

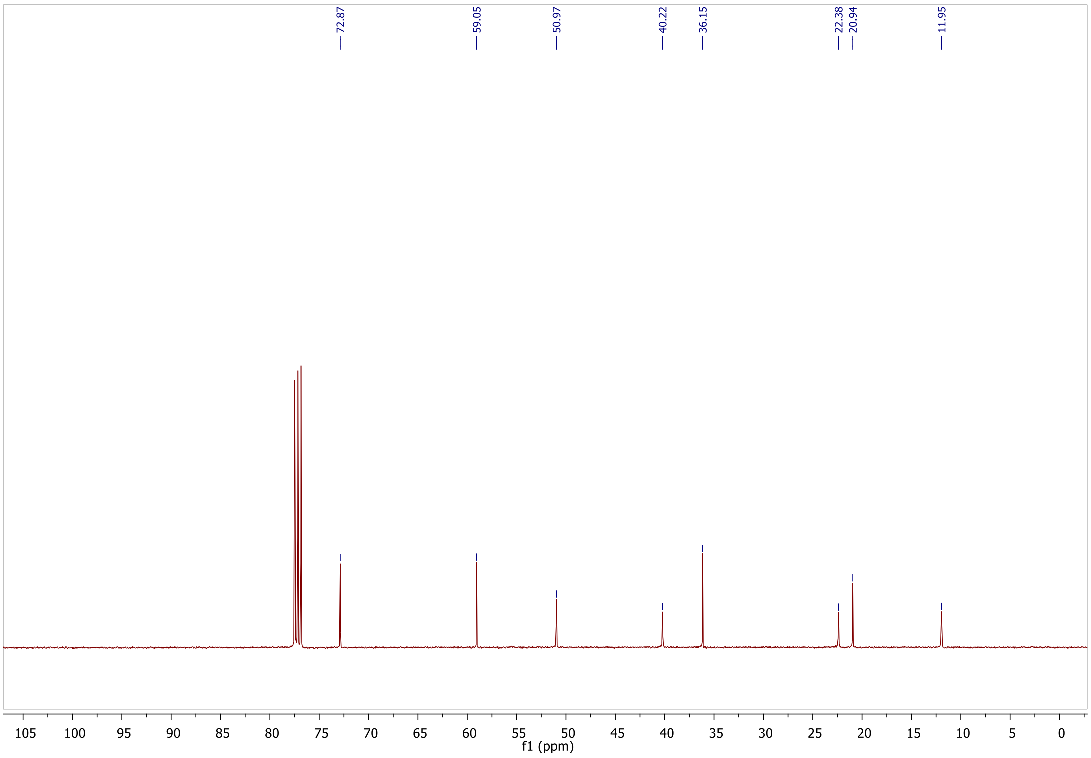

400 MHz ^{1}H NMR spectrum; 100.6 MHz ^{13}C NMR spectrum; D_2O

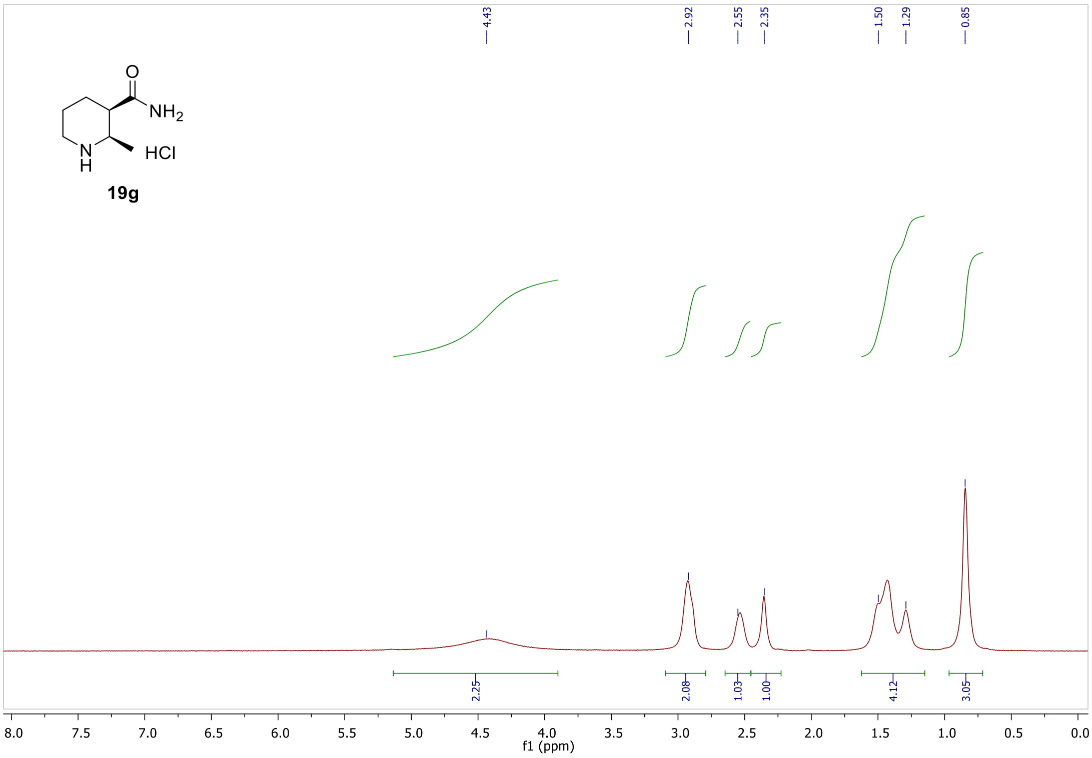

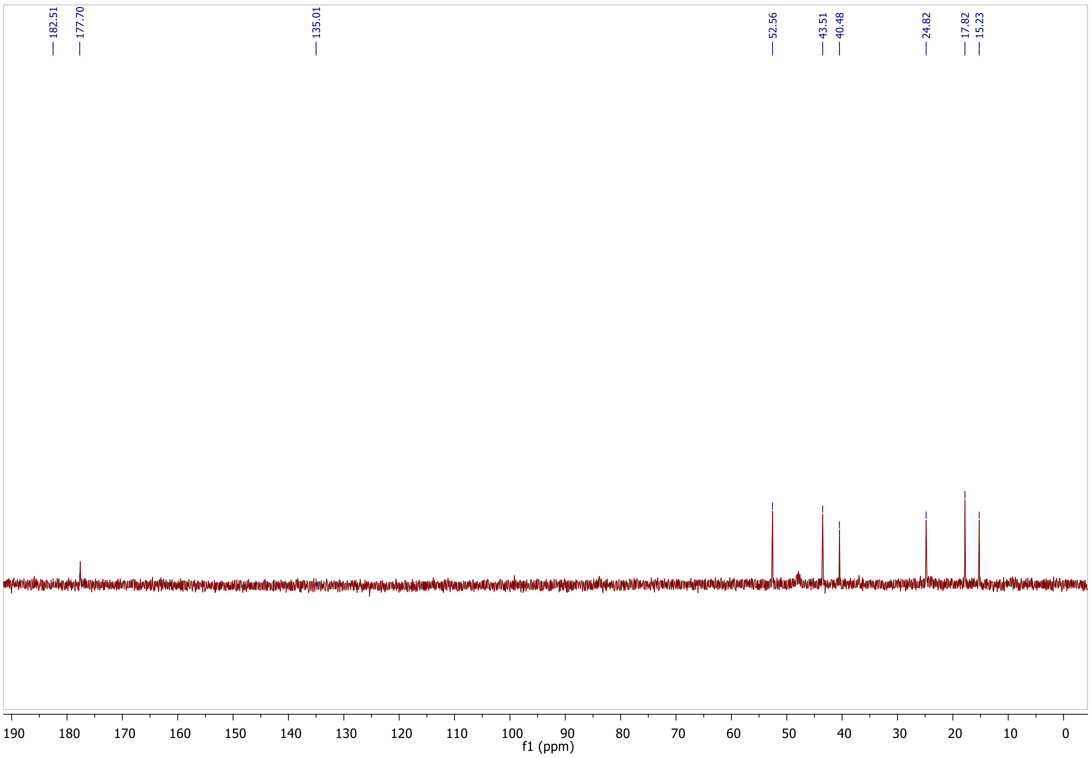

400 MHz ^{1}H NMR spectrum; 100.6 MHz ^{13}C NMR spectrum; $CDCl_3$

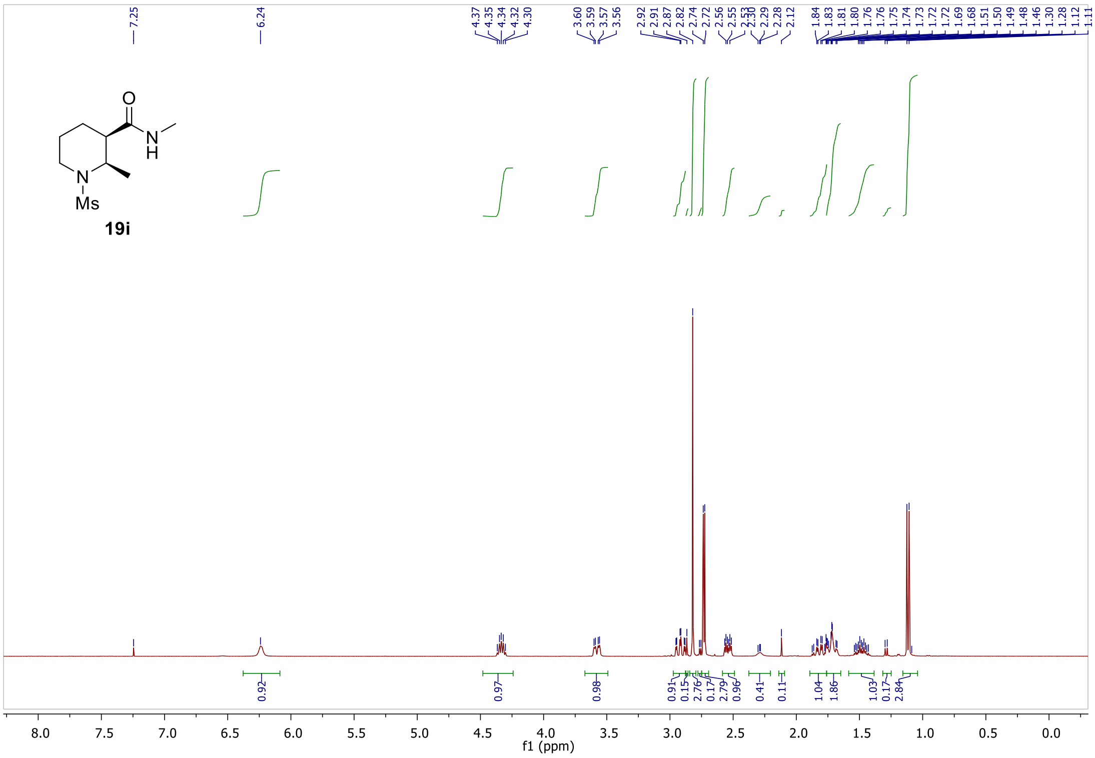

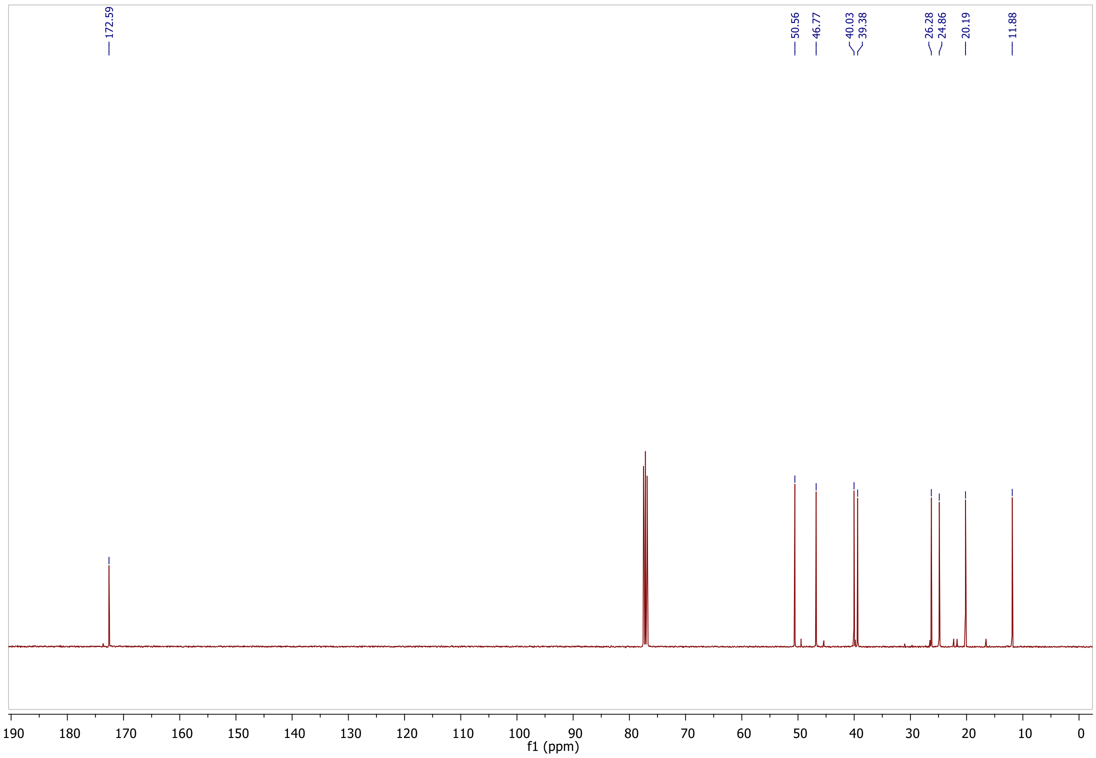

400 MHz ^{1}H NMR spectrum; 100.6 MHz ^{13}C NMR spectrum; $CDCl_3$

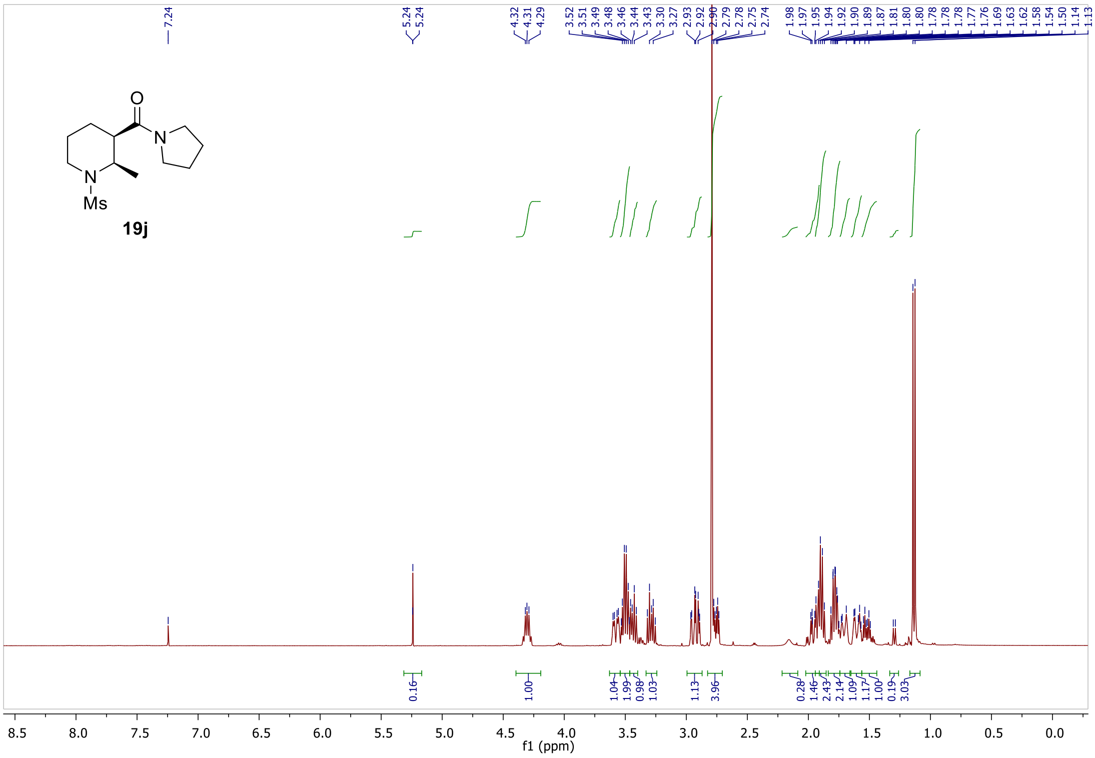

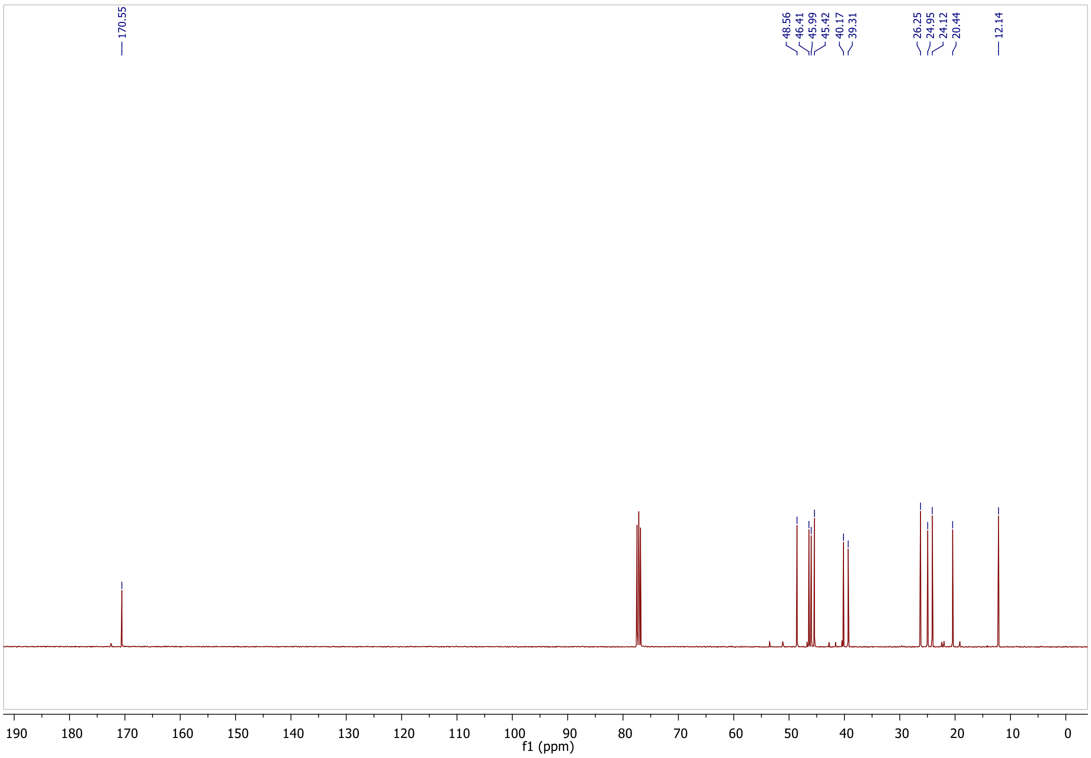

400 MHz ^{1}H NMR spectrum; 100.6 MHz ^{13}C NMR spectrum; $CDCl_3$

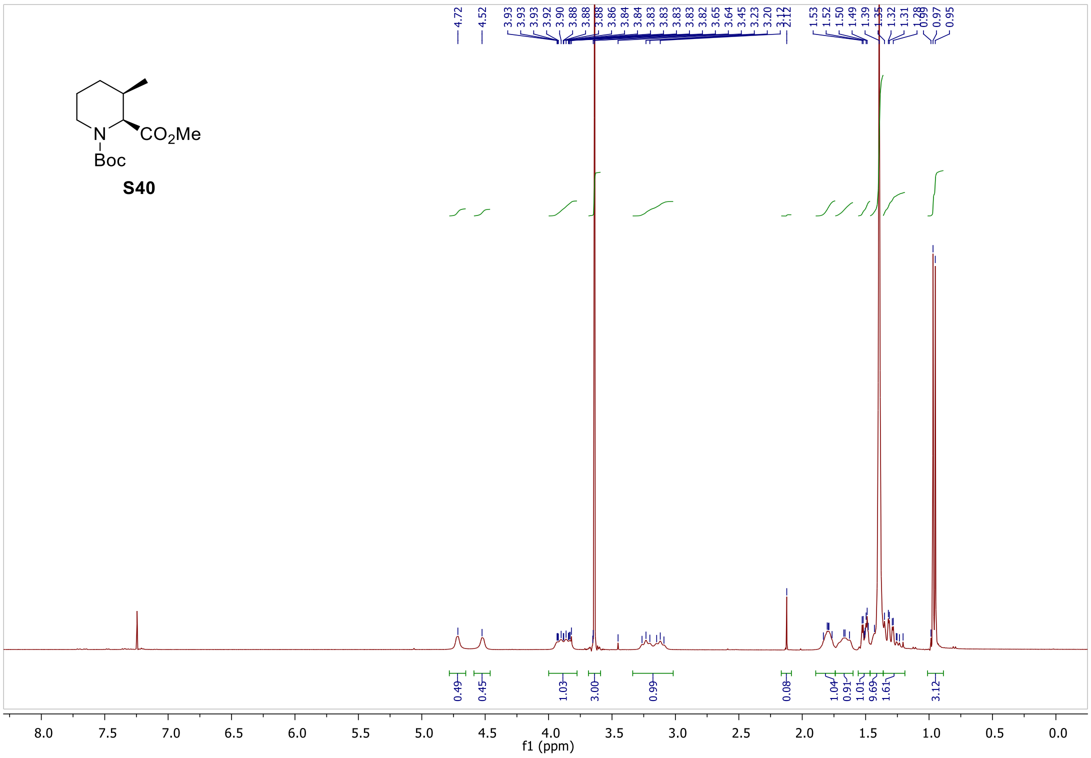

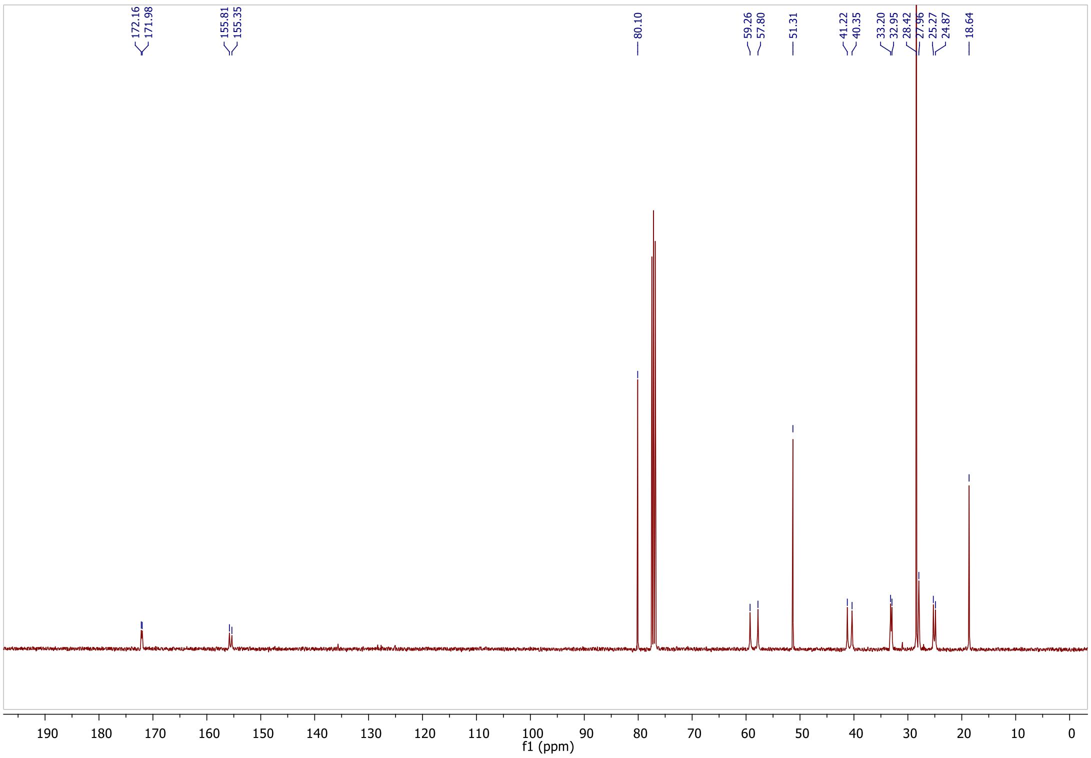

400 MHz ^{1}H NMR spectrum; 100.6 MHz ^{13}C NMR spectrum; $CDCl_3$

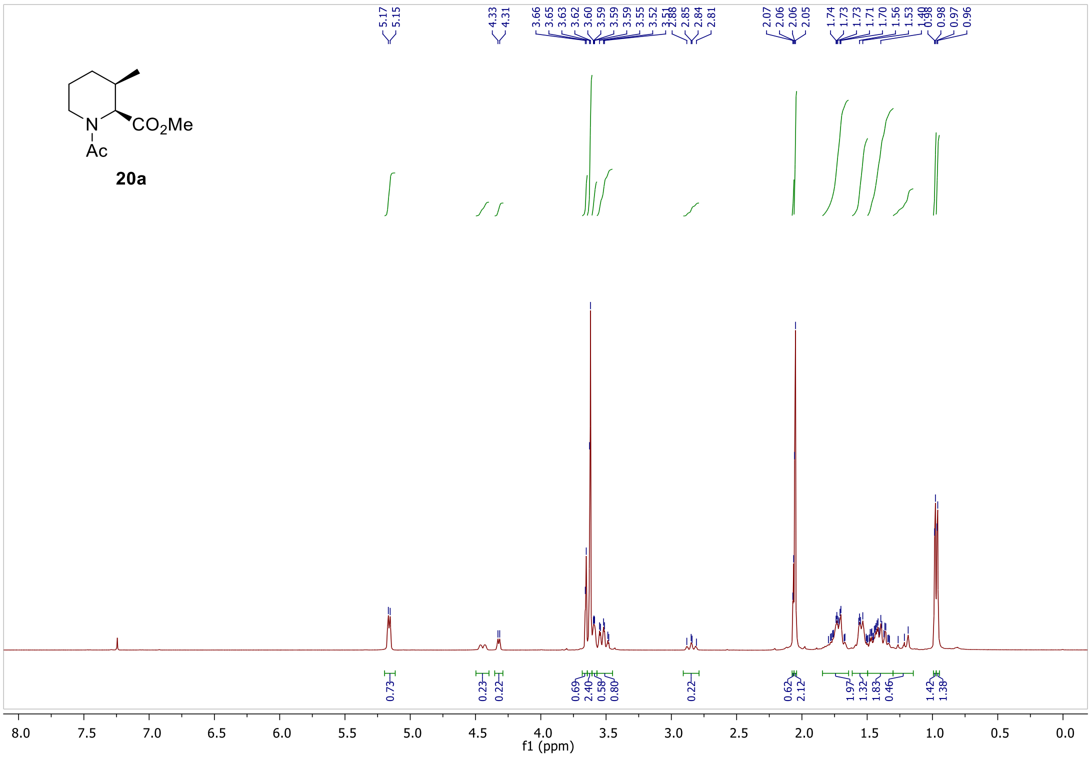

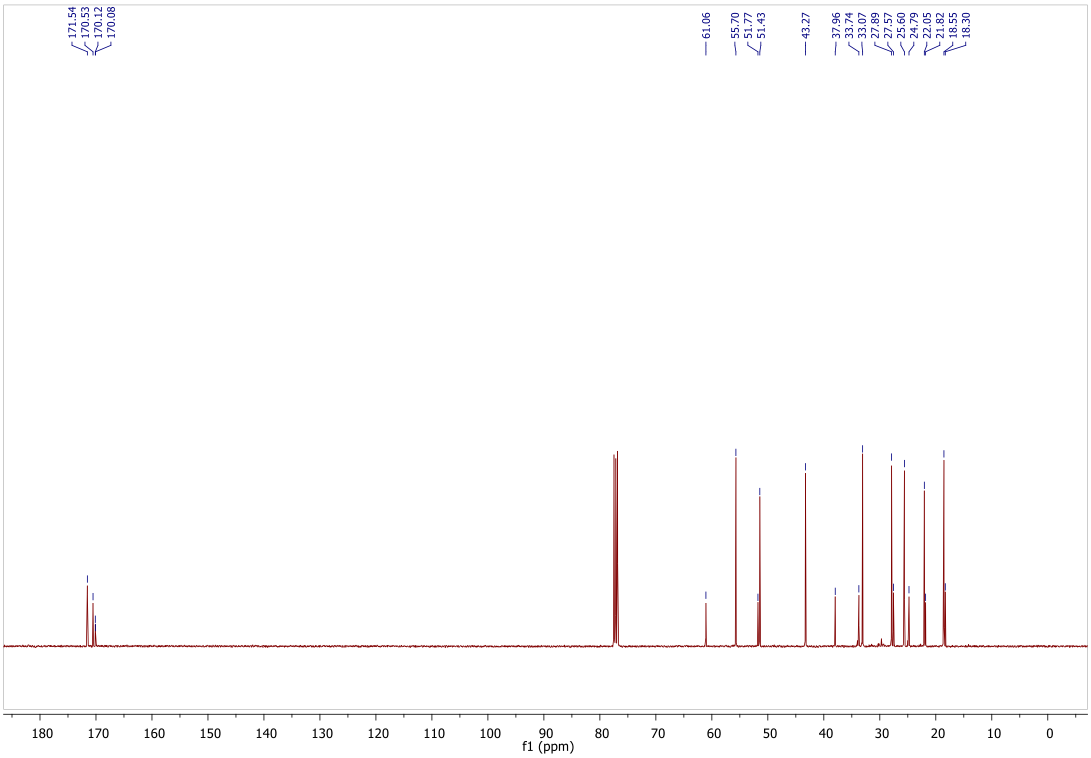

400 MHz ^{1}H NMR spectrum; 100.6 MHz ^{13}C NMR spectrum; $CDCl_3$

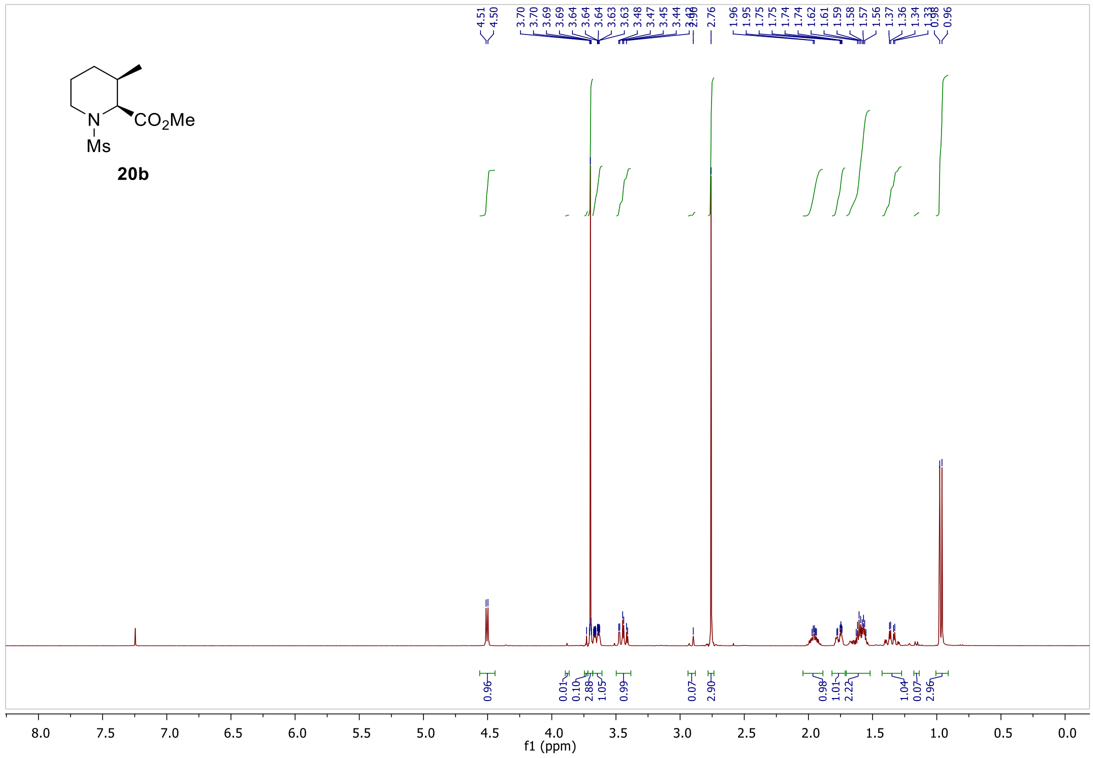

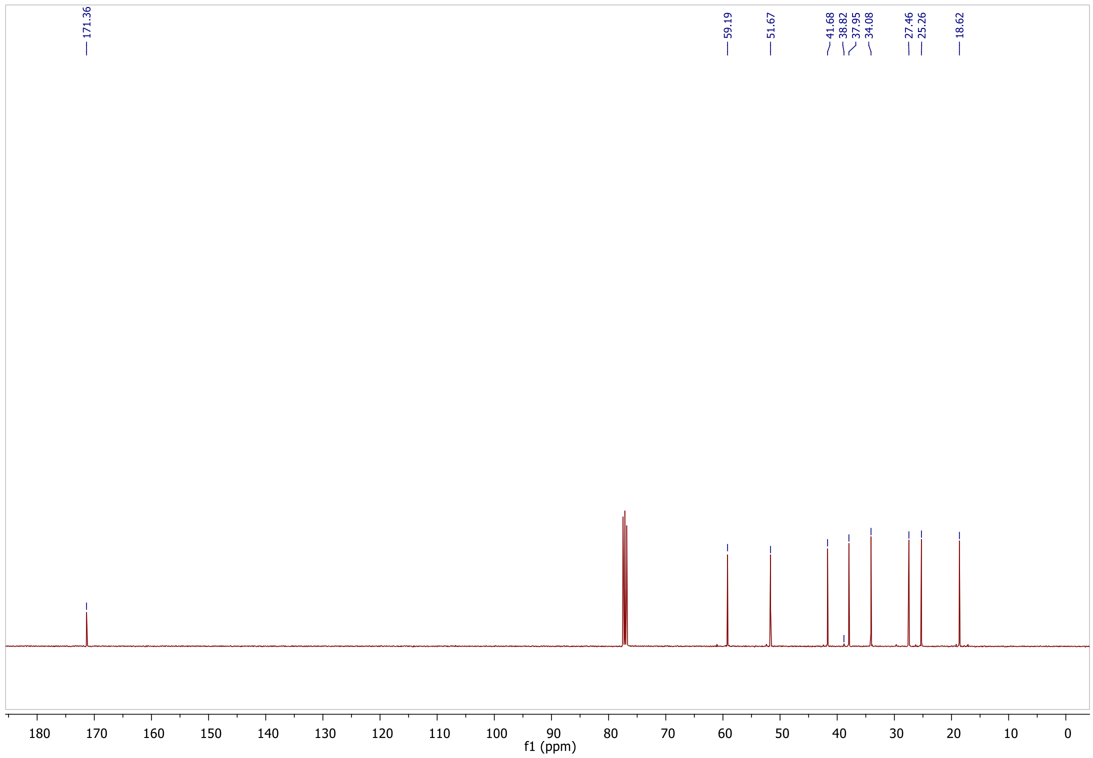

400 MHz ^{1}H NMR spectrum; 100.6 MHz ^{13}C NMR spectrum; $CDCl_3$

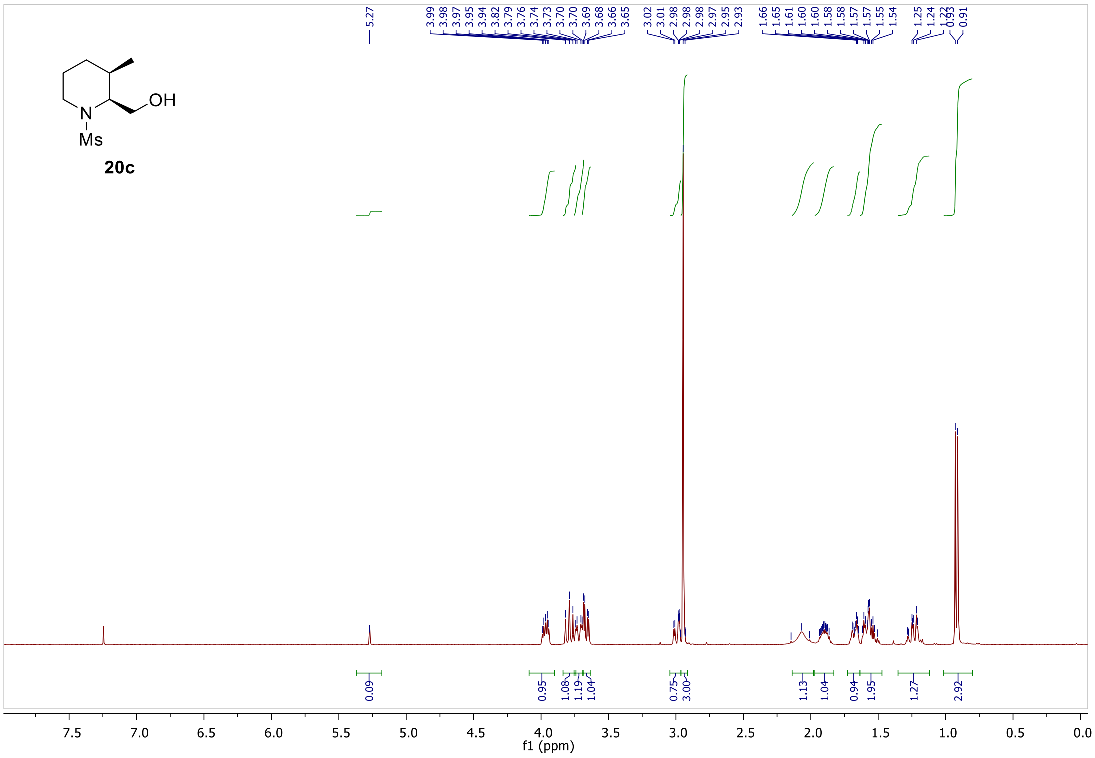

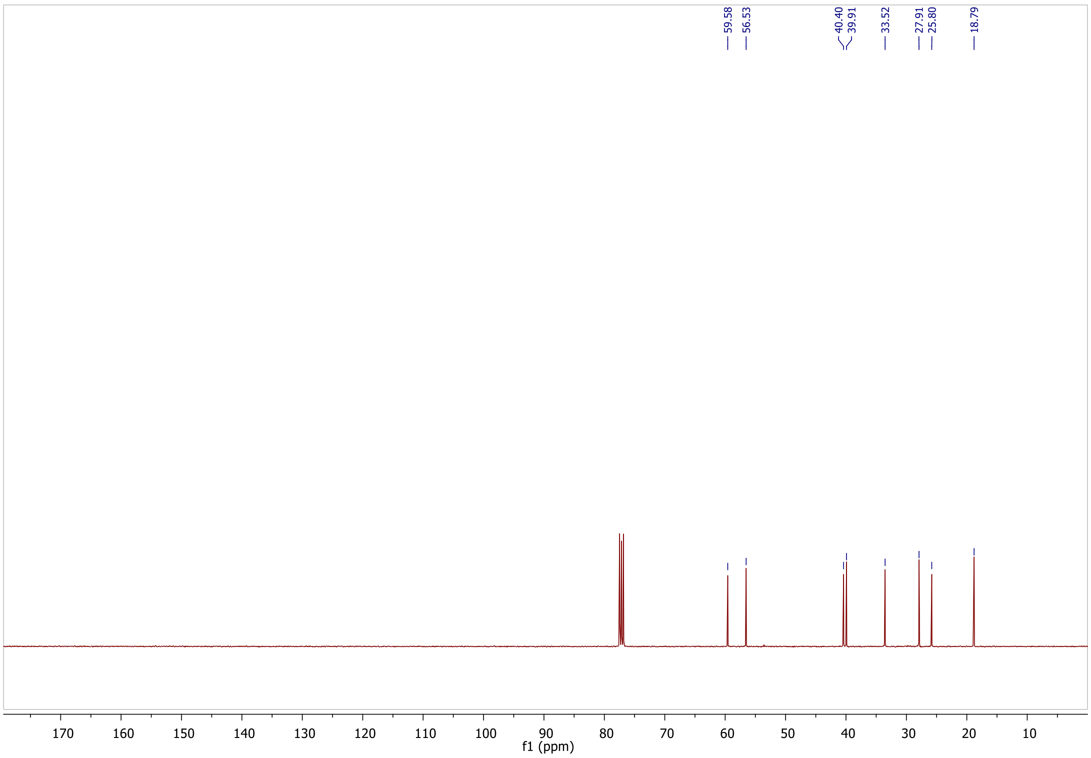

400 MHz ^{1}H NMR spectrum; 100.6 MHz ^{13}C NMR spectrum; $CDCl_3$

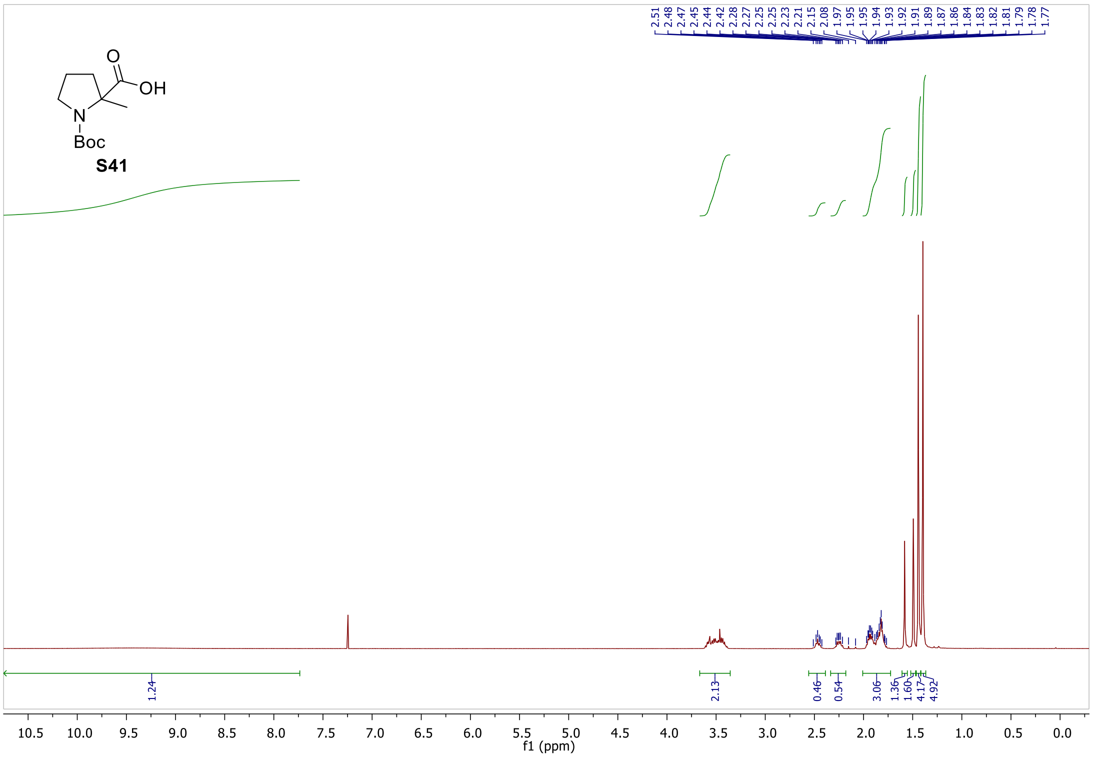

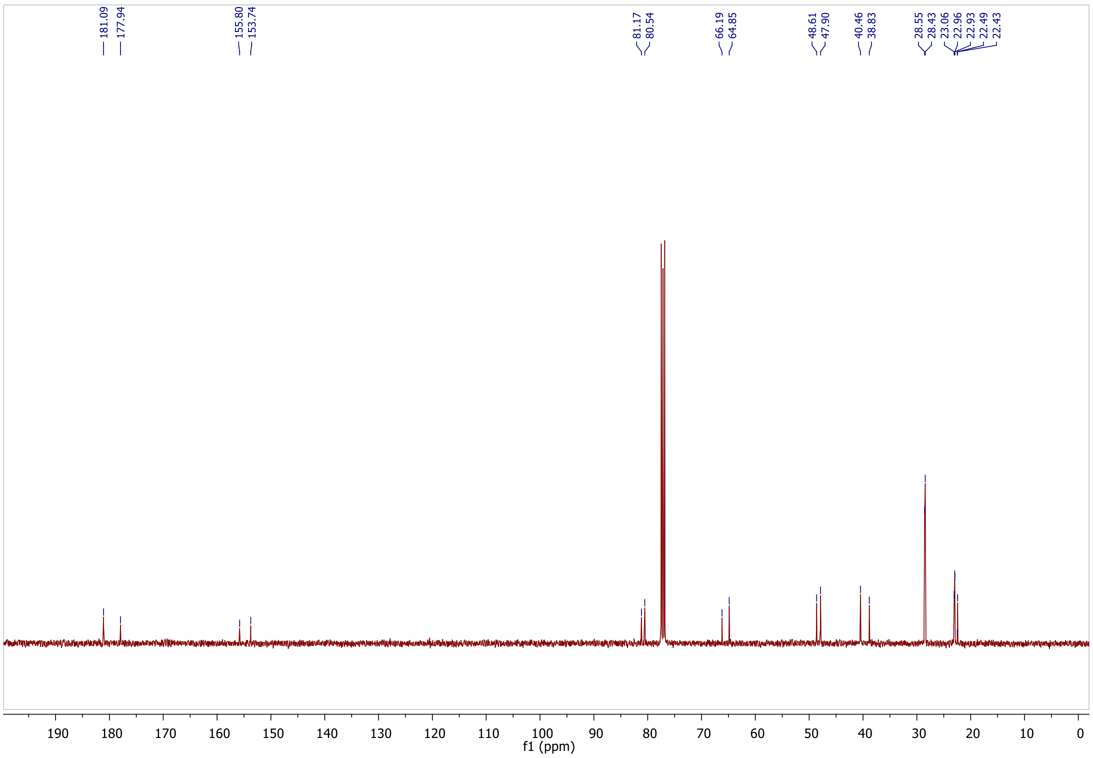

400 MHz ^{1}H NMR spectrum; 100.6 MHz ^{13}C NMR spectrum; $CDCl_3$

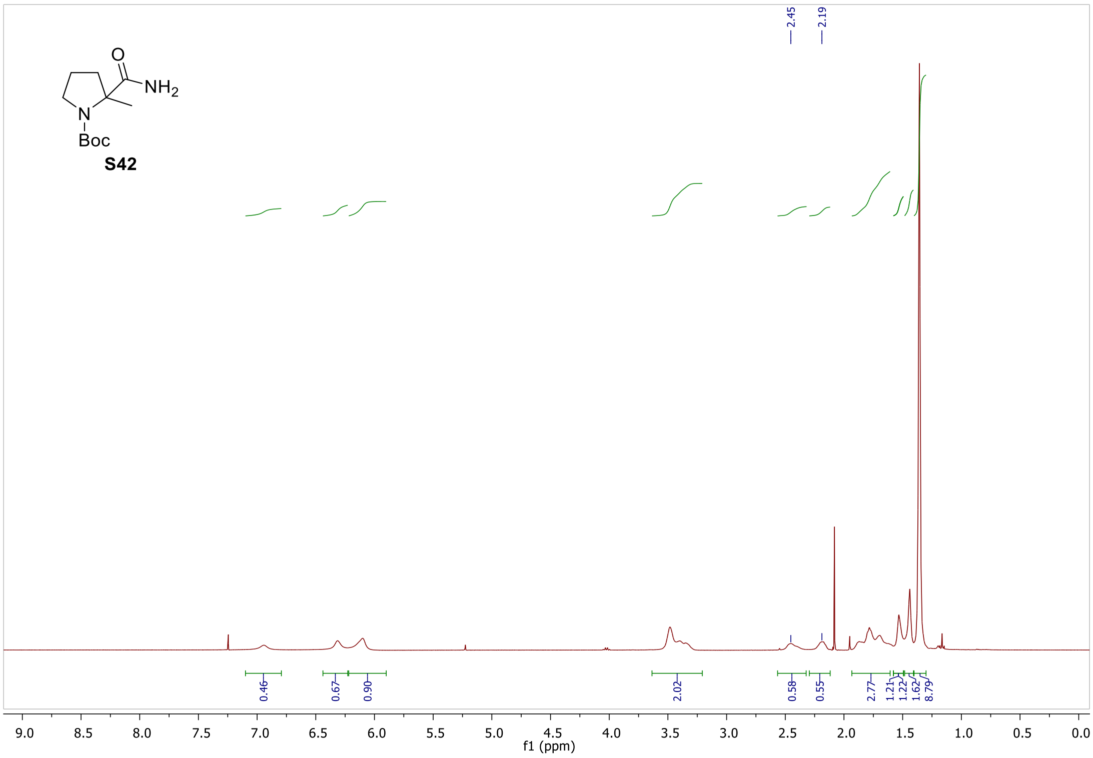

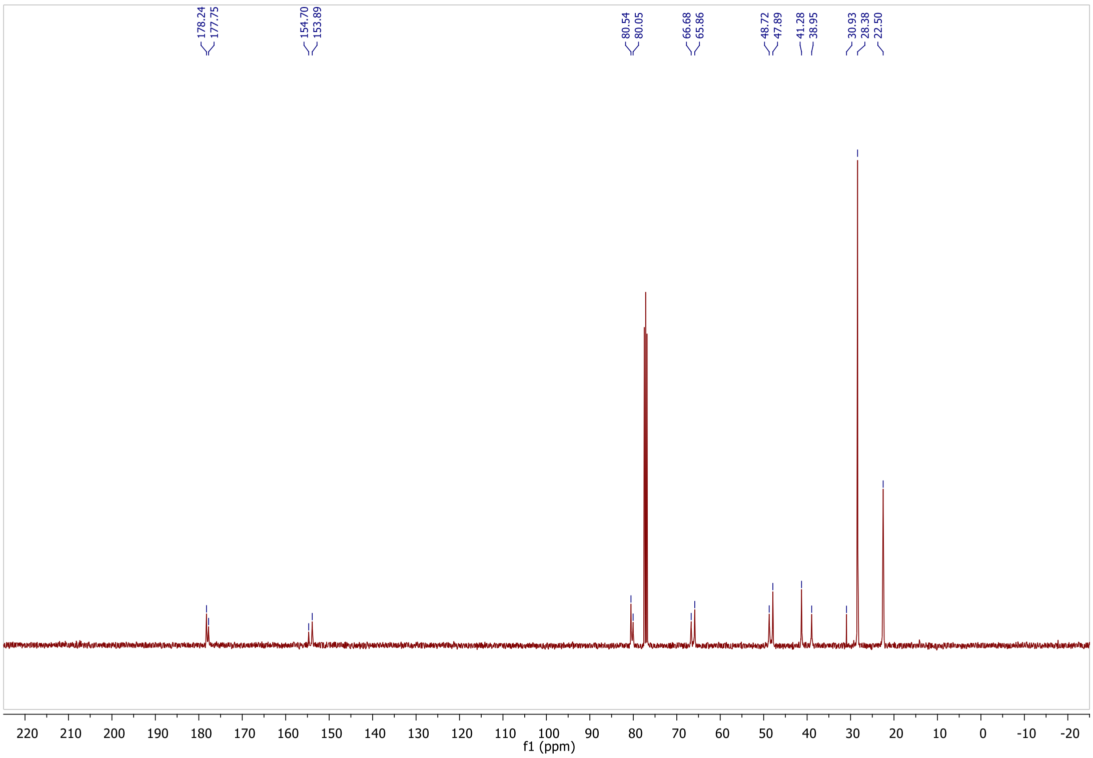

400 MHz ^1H NMR spectrum; 100.6 MHz ^{13}C NMR spectrum; MeOH-d_4

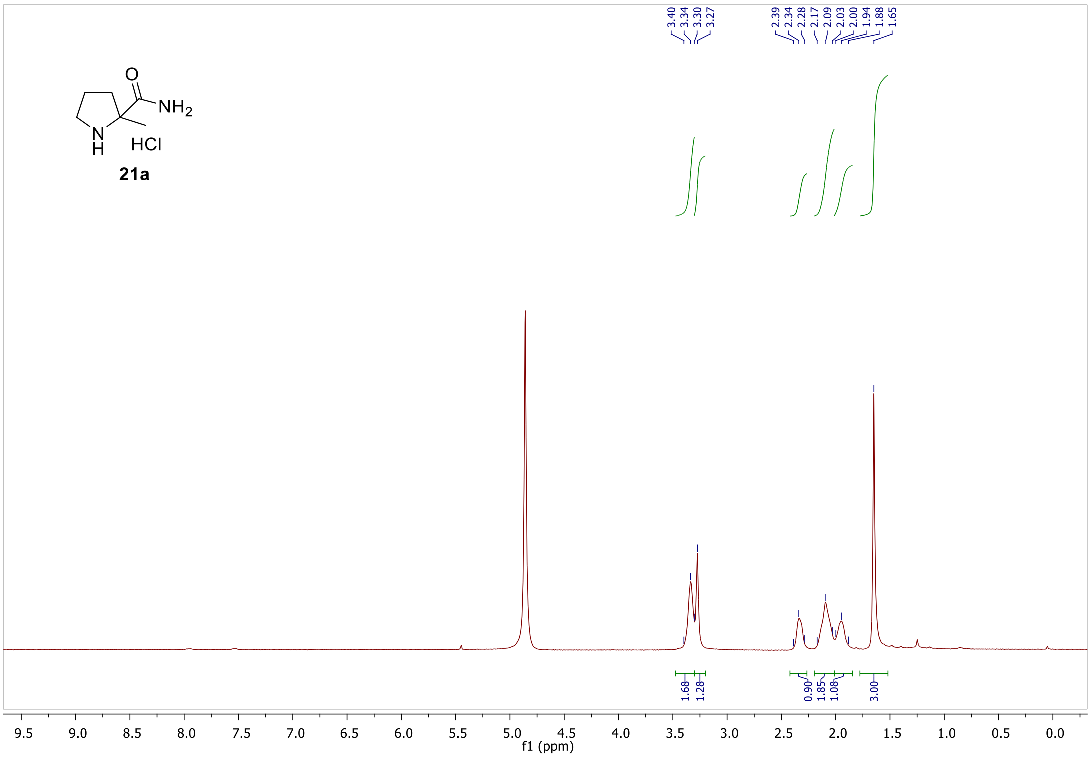

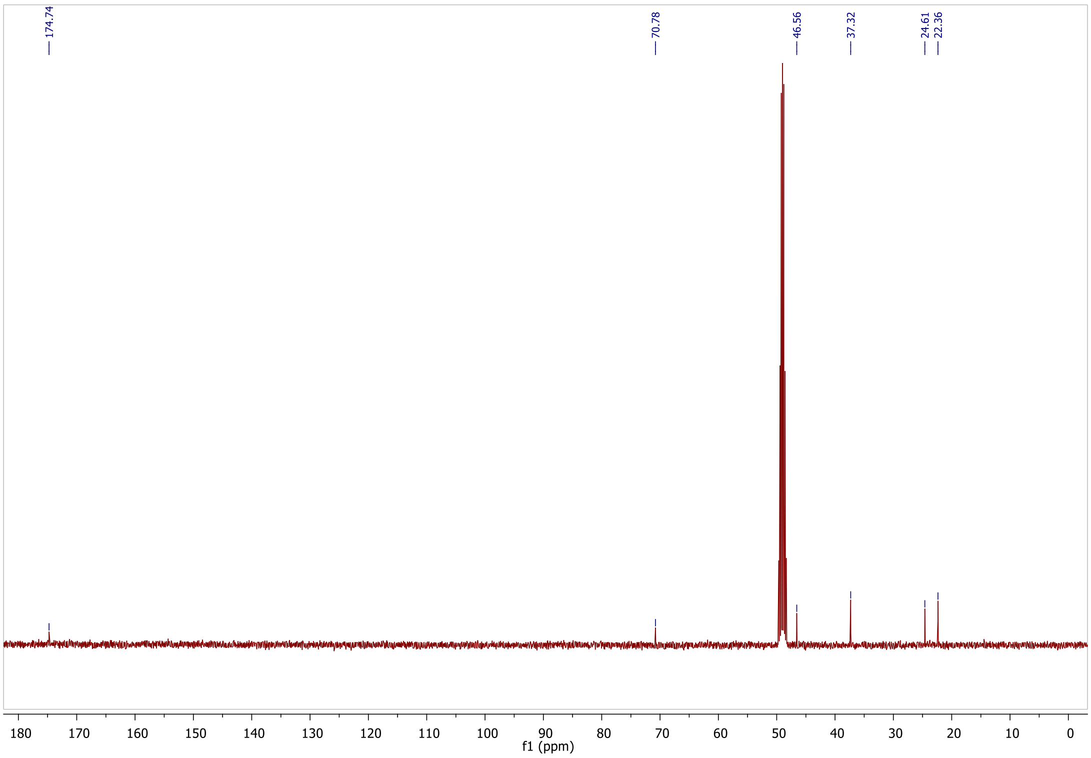

400 MHz ^{1}H NMR spectrum; 100.6 MHz ^{13}C NMR spectrum; MeOH-d_4

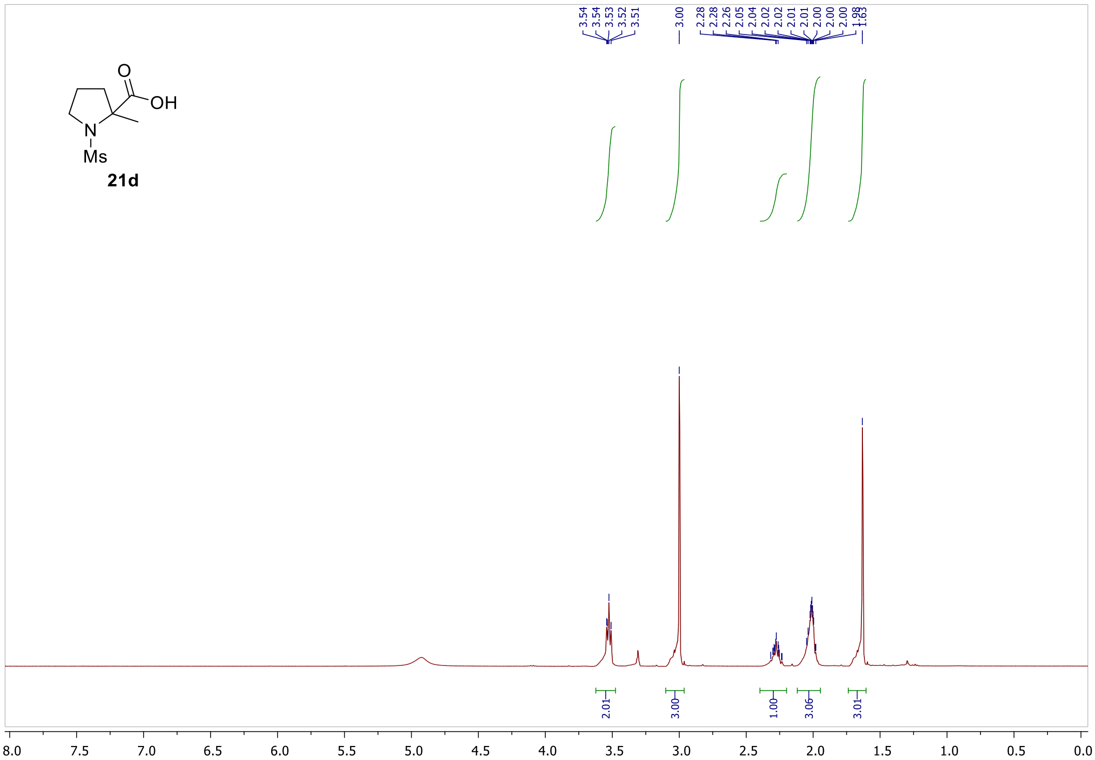

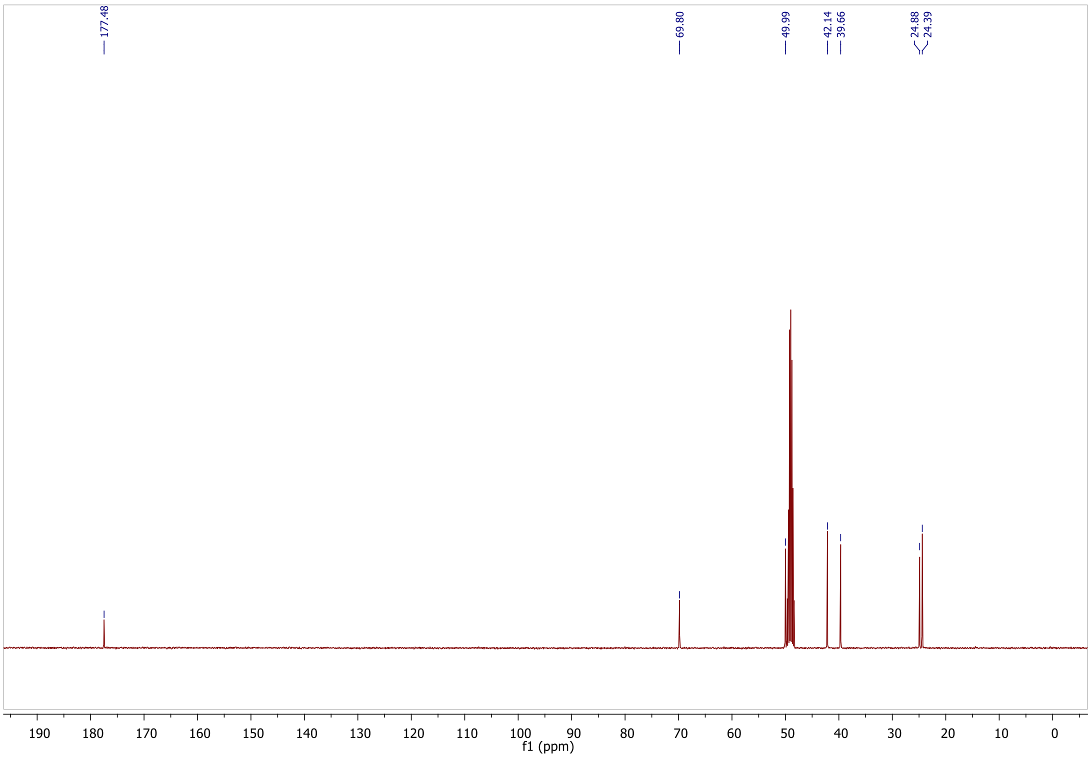

400 MHz ^{1}H NMR spectrum; 100.6 MHz ^{13}C NMR spectrum; $CDCl_3$

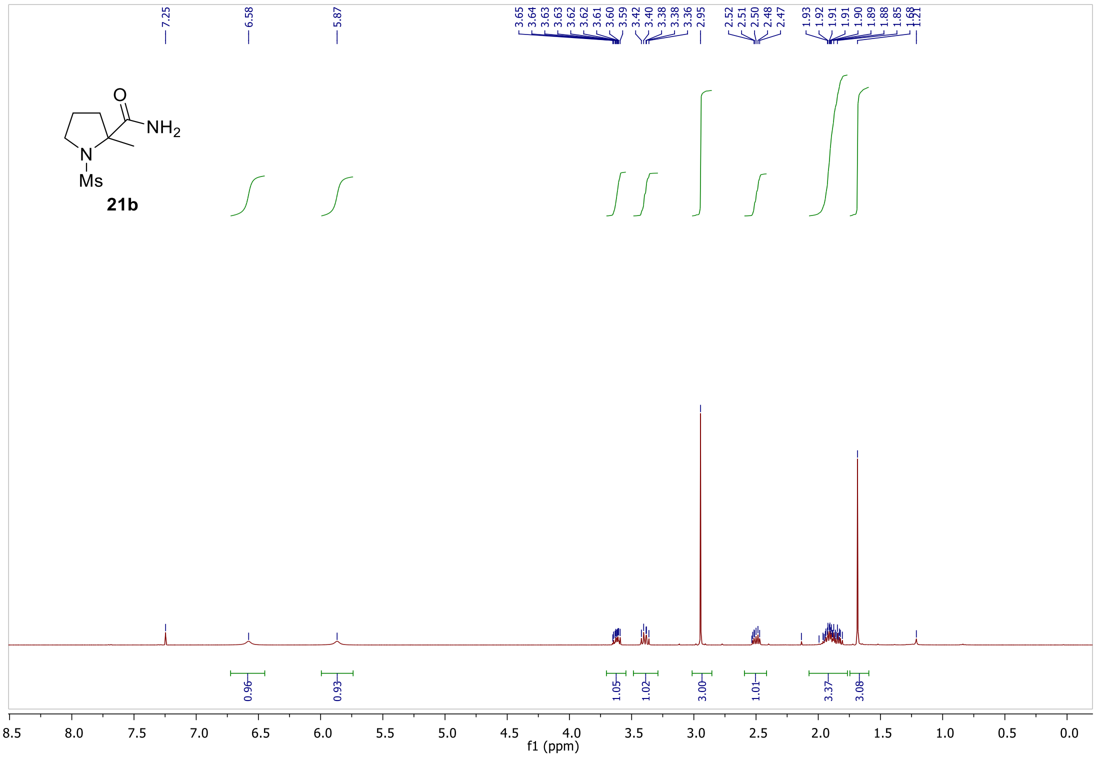

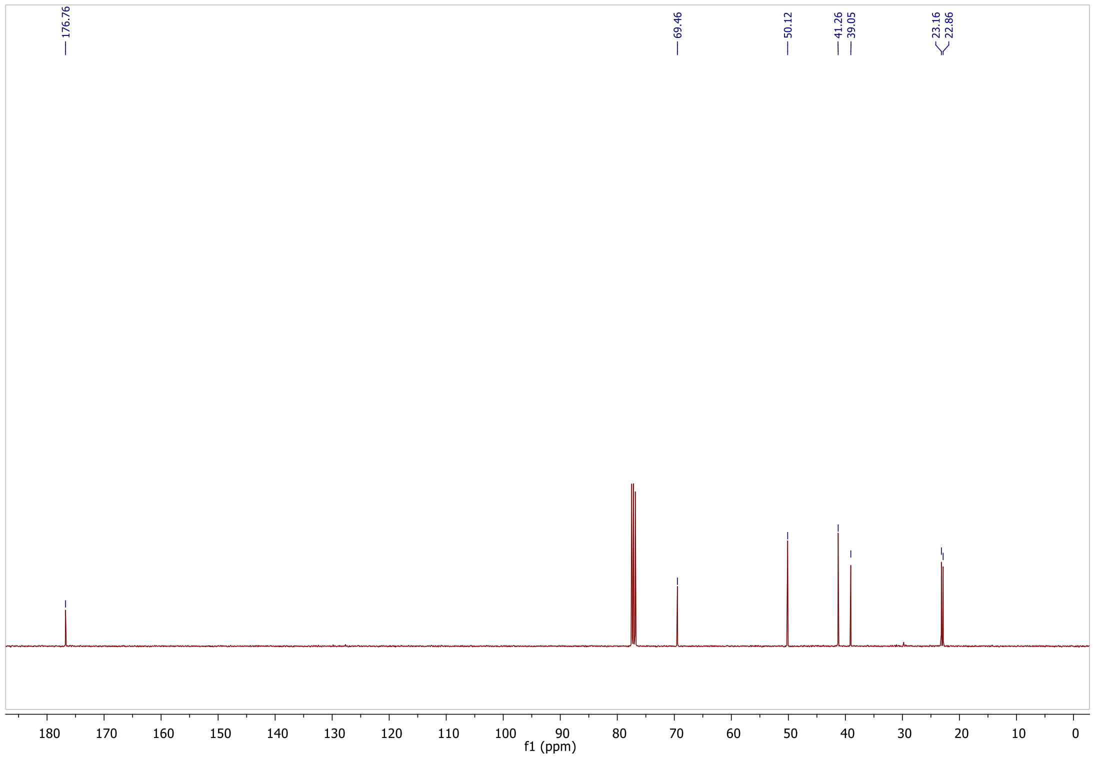

400 MHz ^{1}H NMR spectrum; 100.6 MHz ^{13}C NMR spectrum; $CDCl_3$

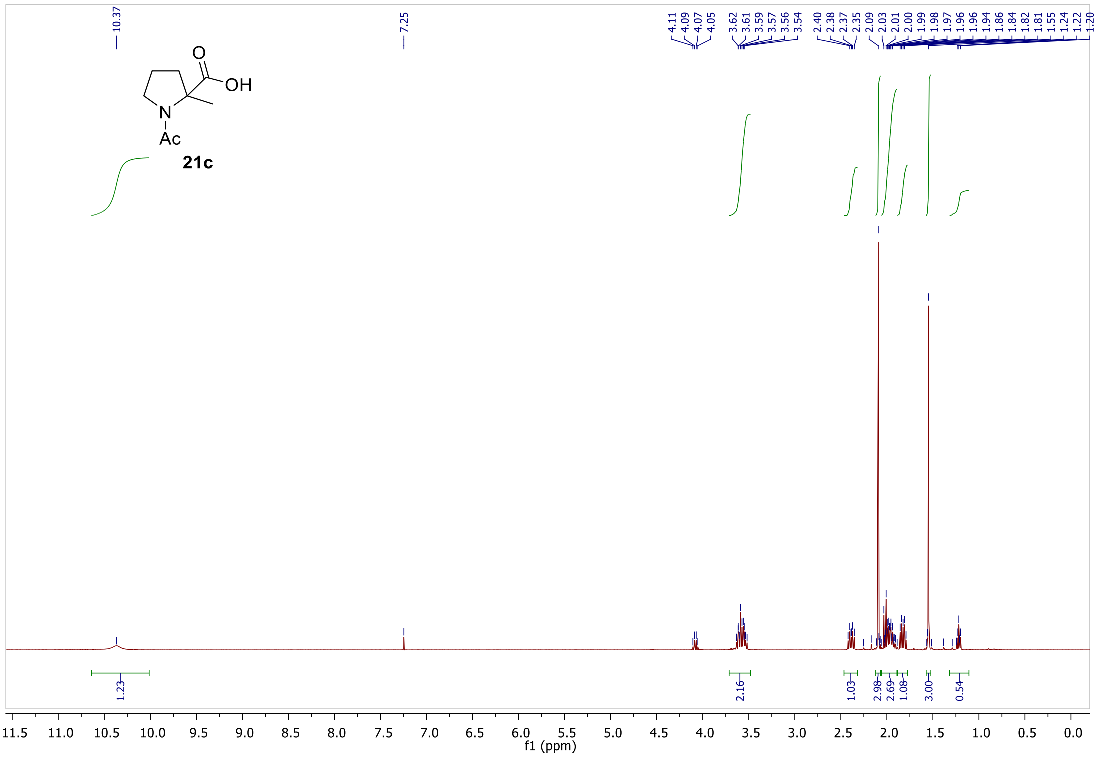

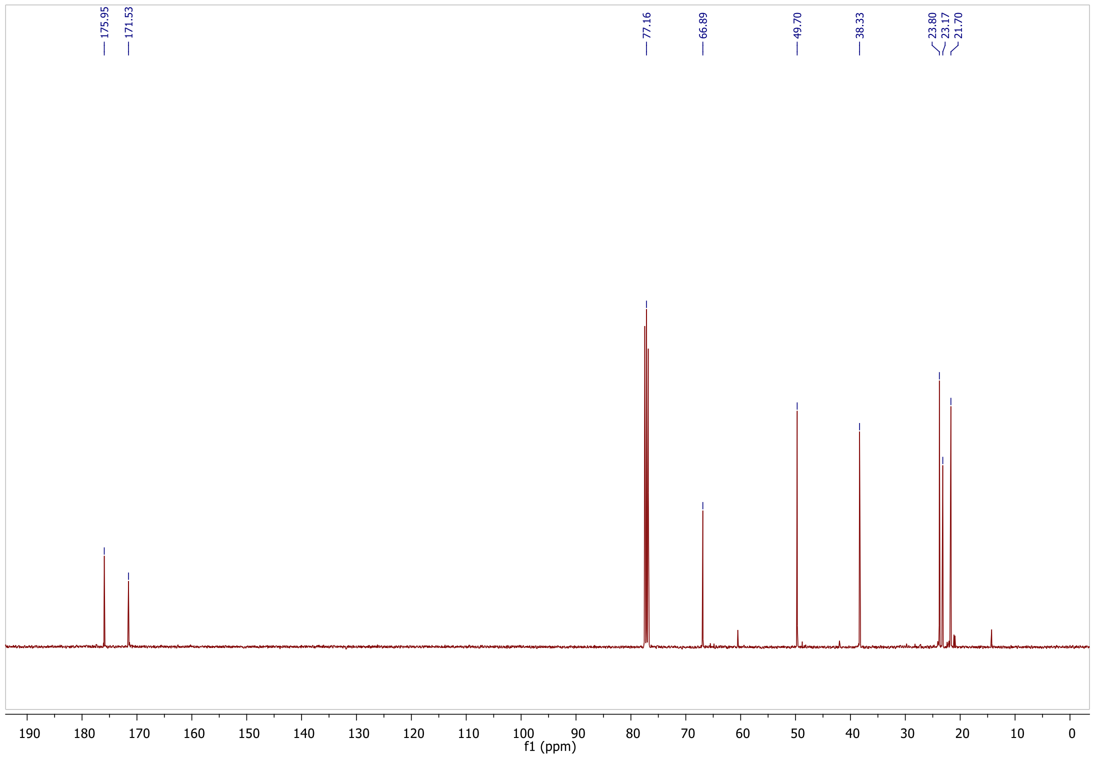

400 MHz ^1H NMR spectrum; 100.6 MHz ^{13}C NMR spectrum; $CDCl_3$

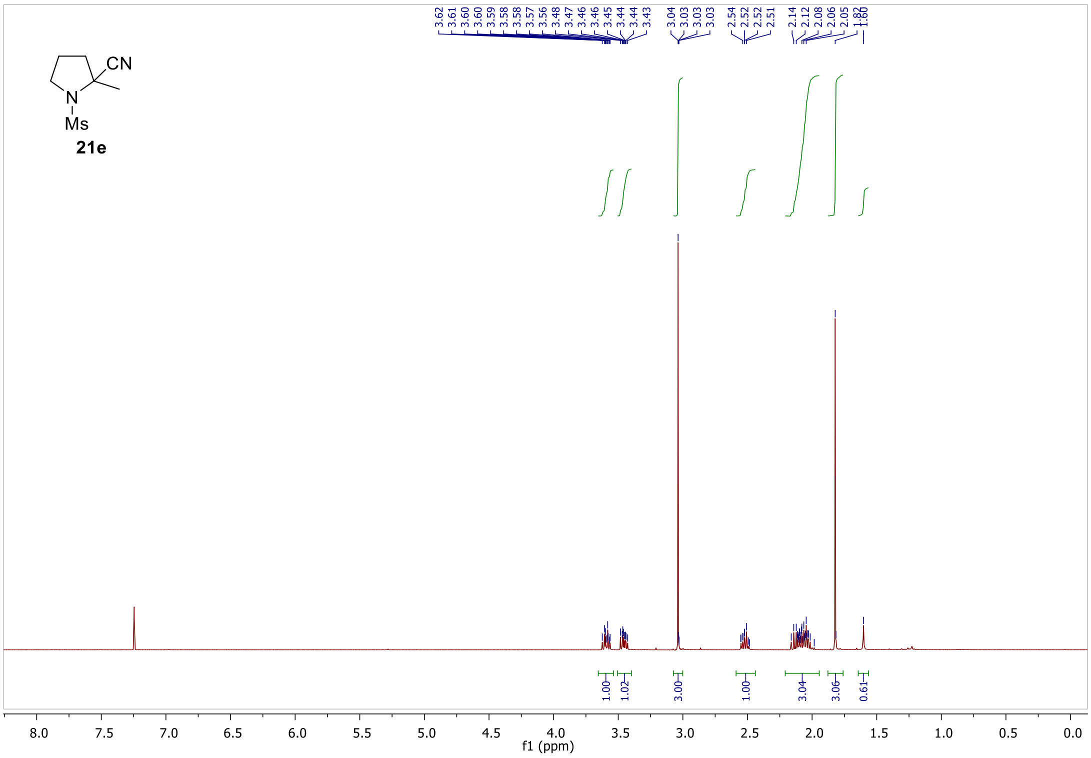

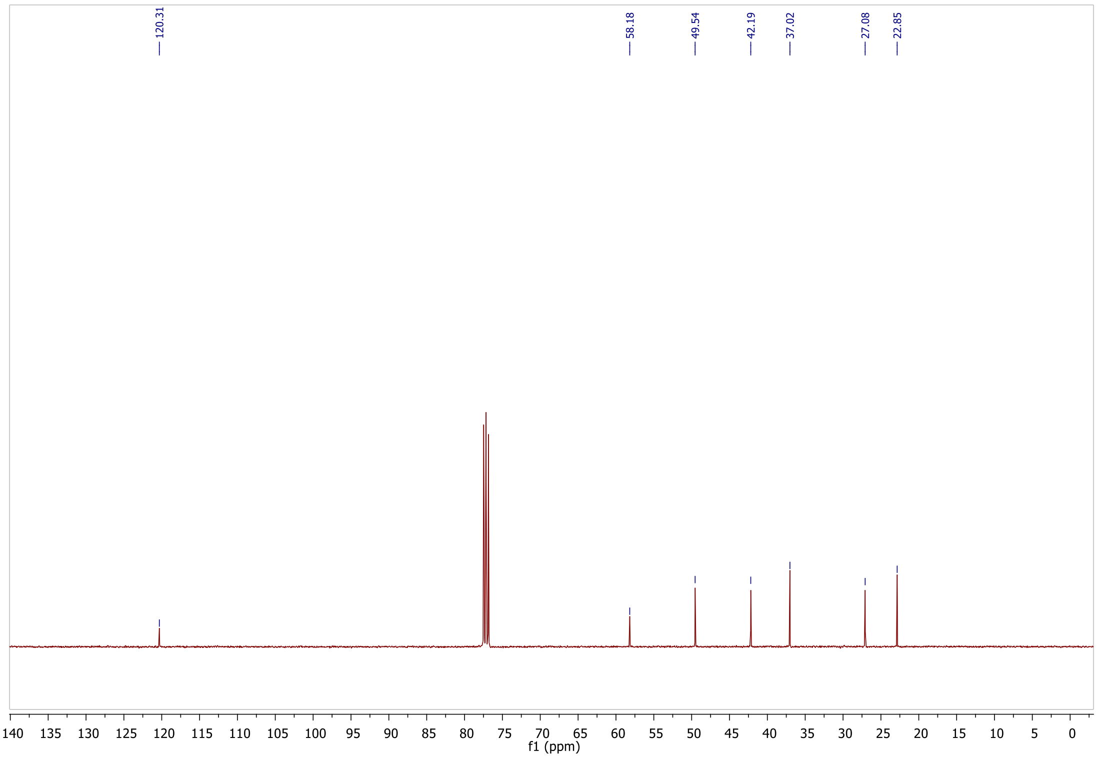

400 MHz ^{1}H NMR spectrum; 100.6 MHz ^{13}C NMR spectrum; $CDCl_3$

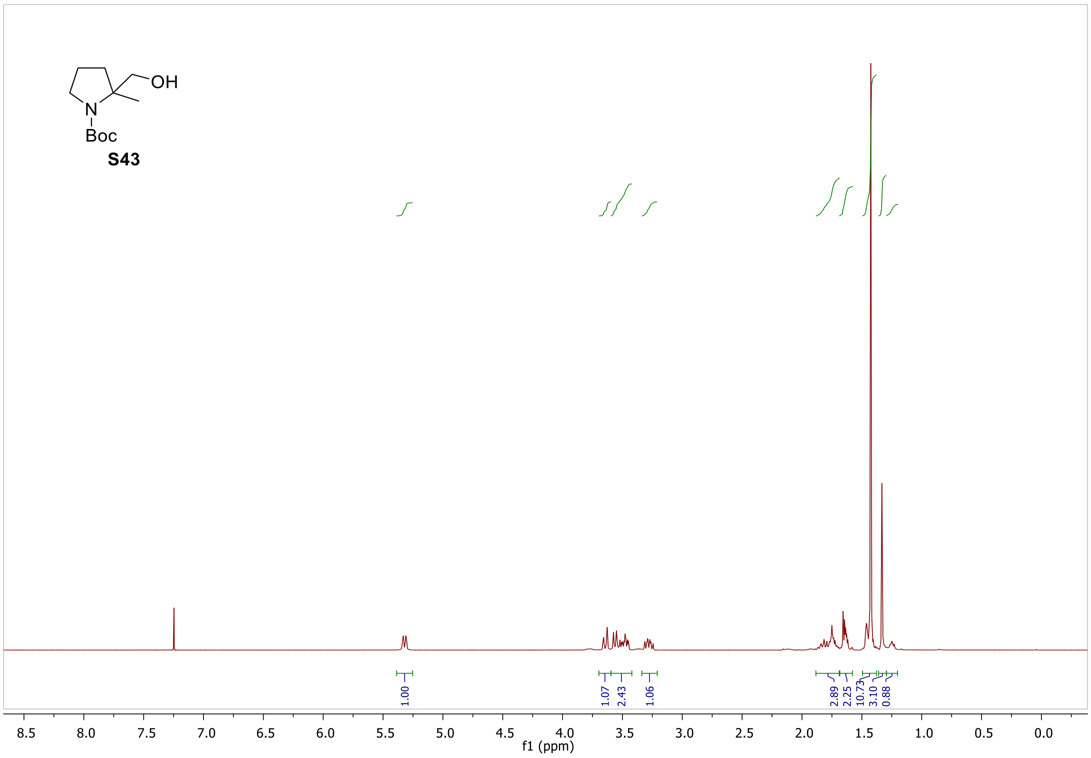

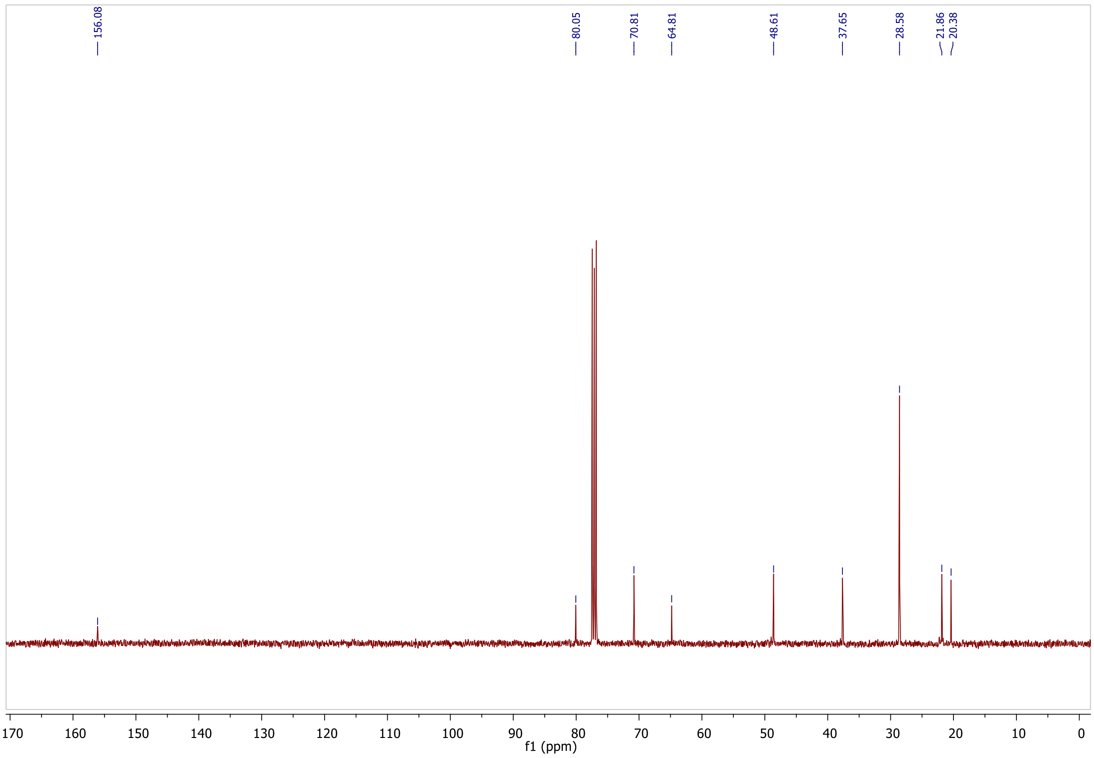

400 MHz ^{1}H NMR spectrum; 100.6 MHz ^{13}C NMR spectrum; $CDCl_3$

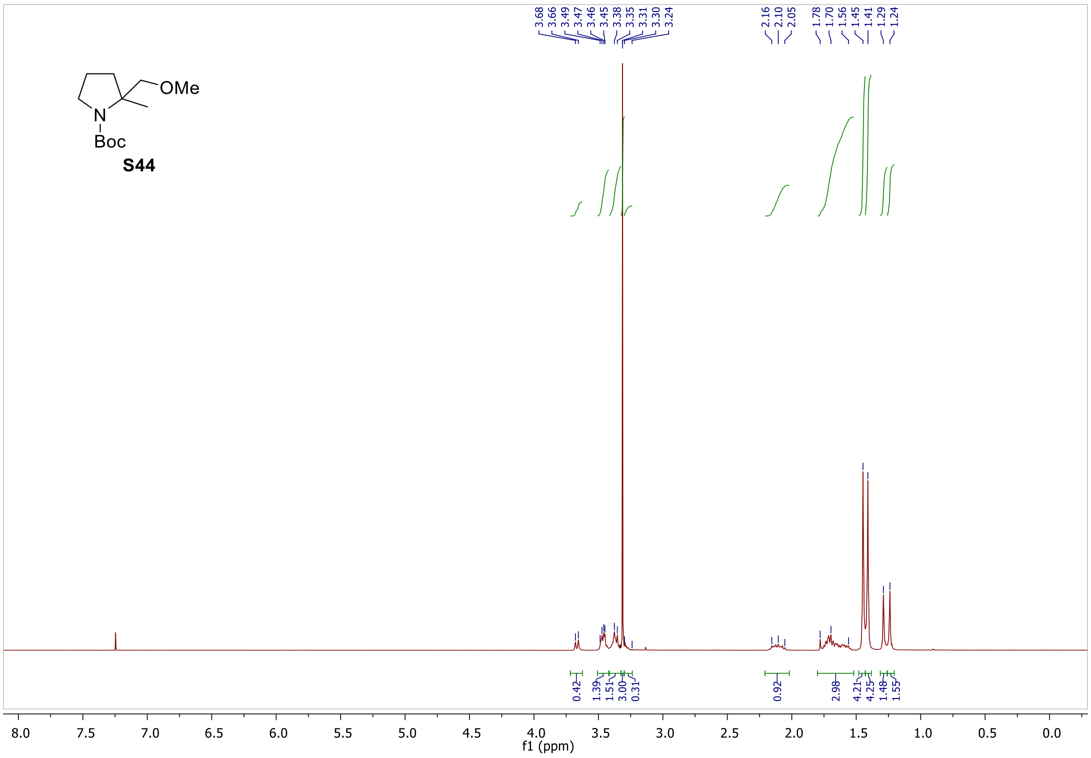

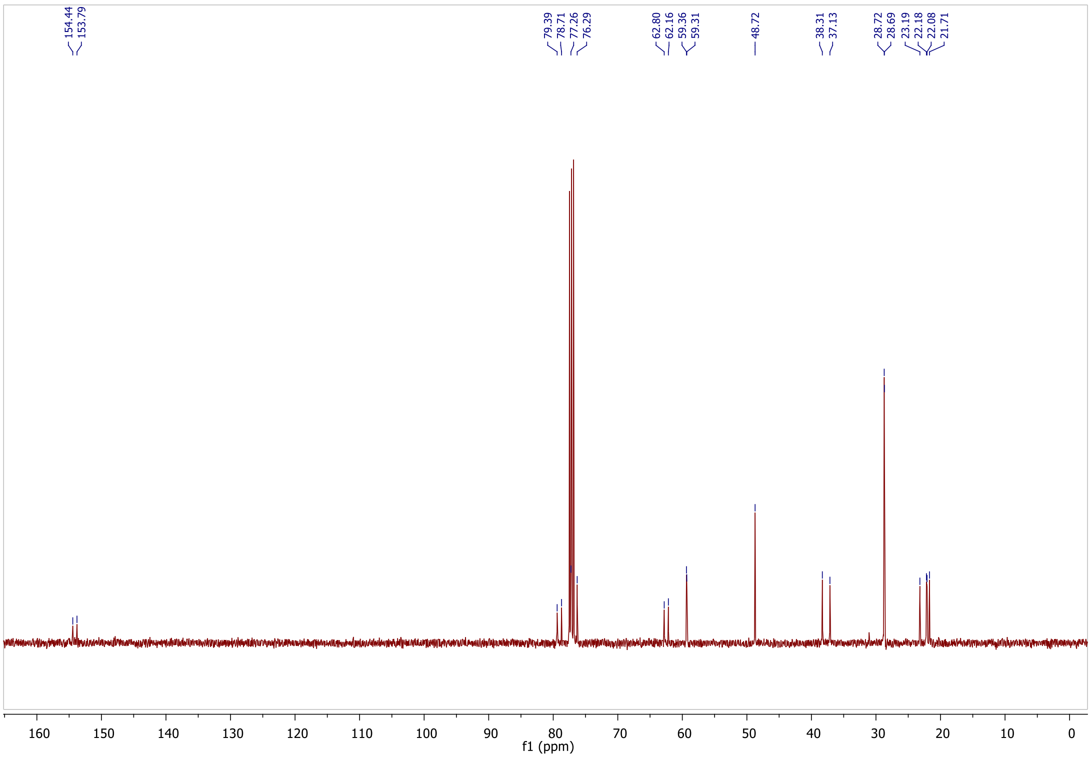

400 MHz ^{1}H NMR spectrum; 100.6 MHz ^{13}C NMR spectrum; MeOH-d_4

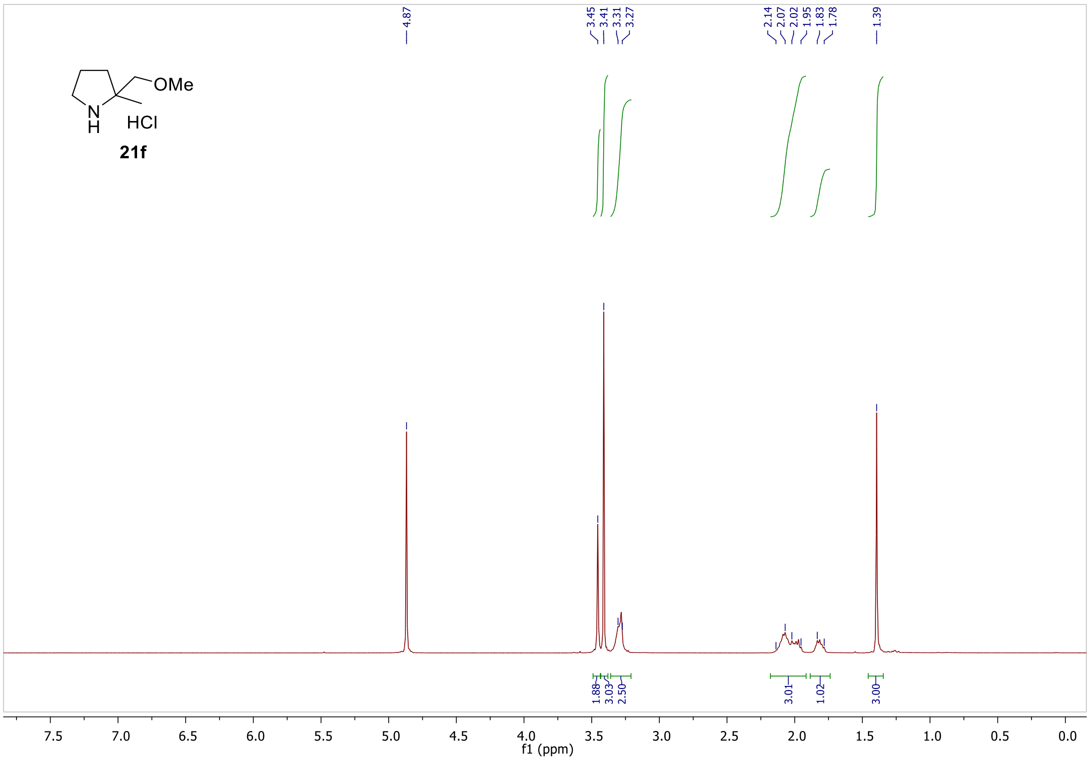

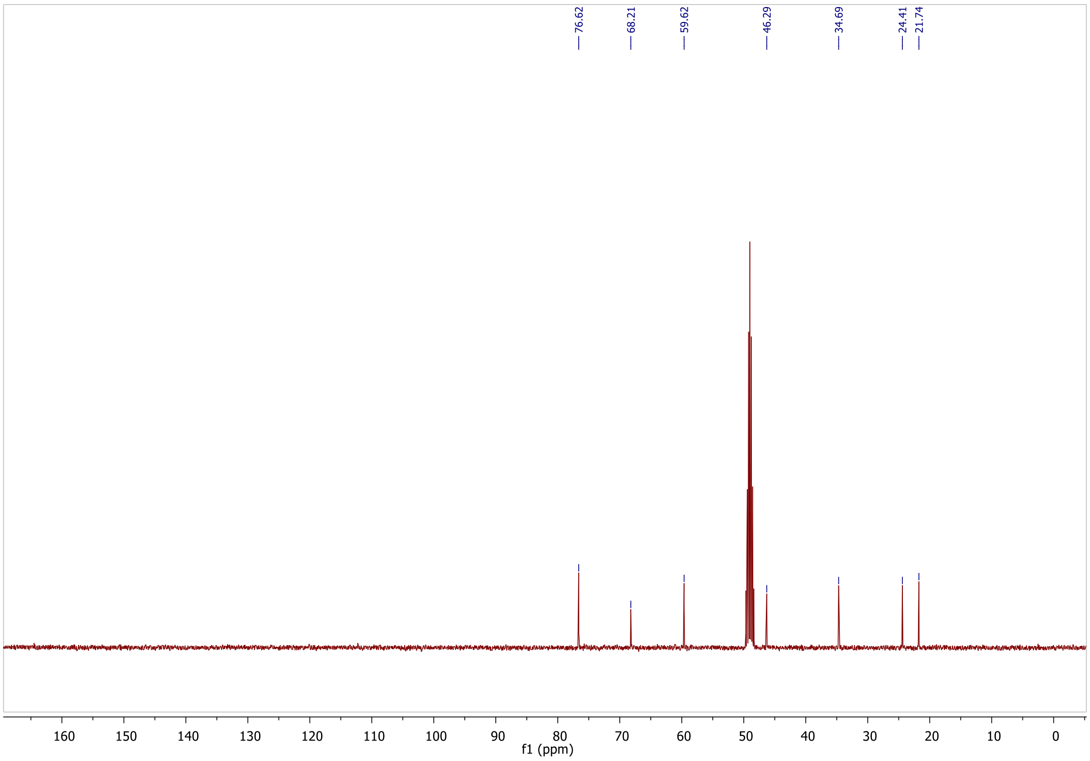

400 MHz ^{1}H NMR spectrum; 100.6 MHz ^{13}C NMR spectrum; $CDCl_3$

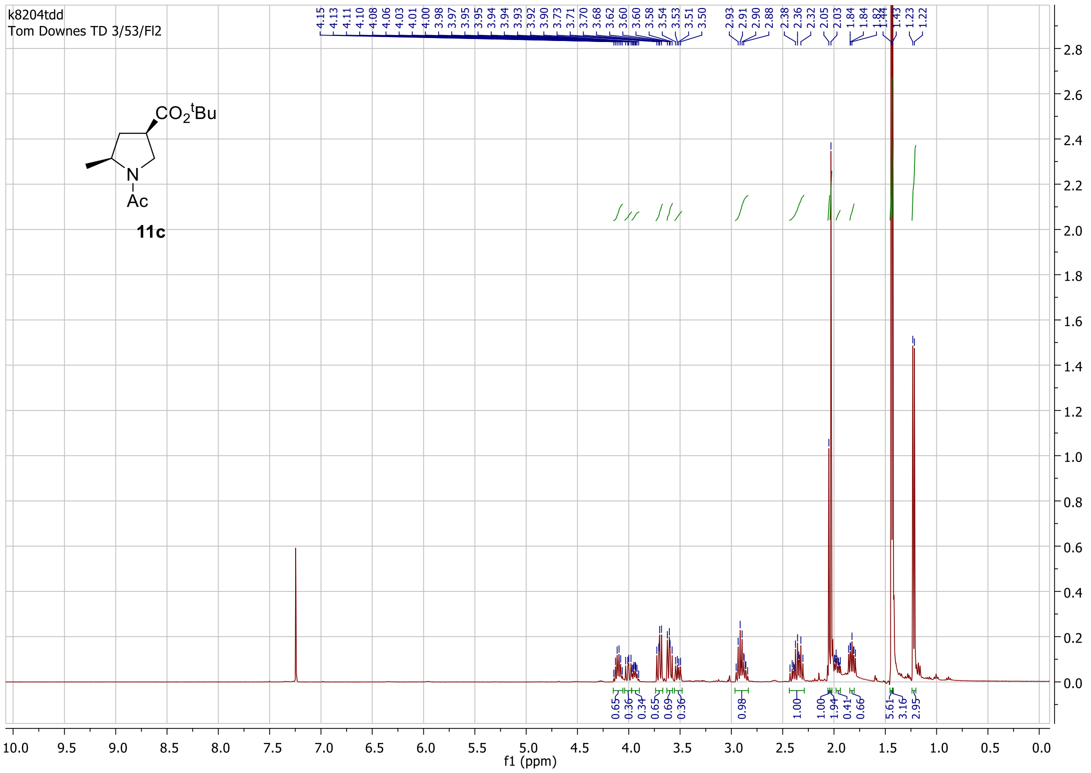

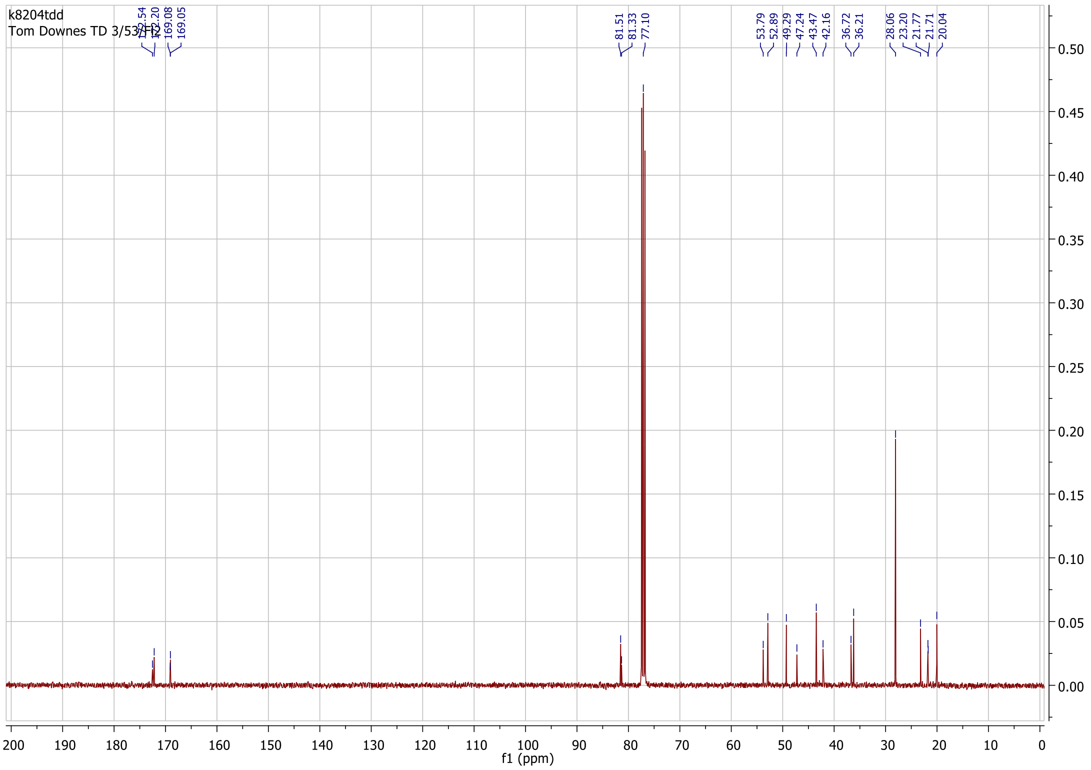

400 MHz ^{1}H NMR spectrum; 100.6 MHz ^{13}C NMR spectrum; $CDCl_3$

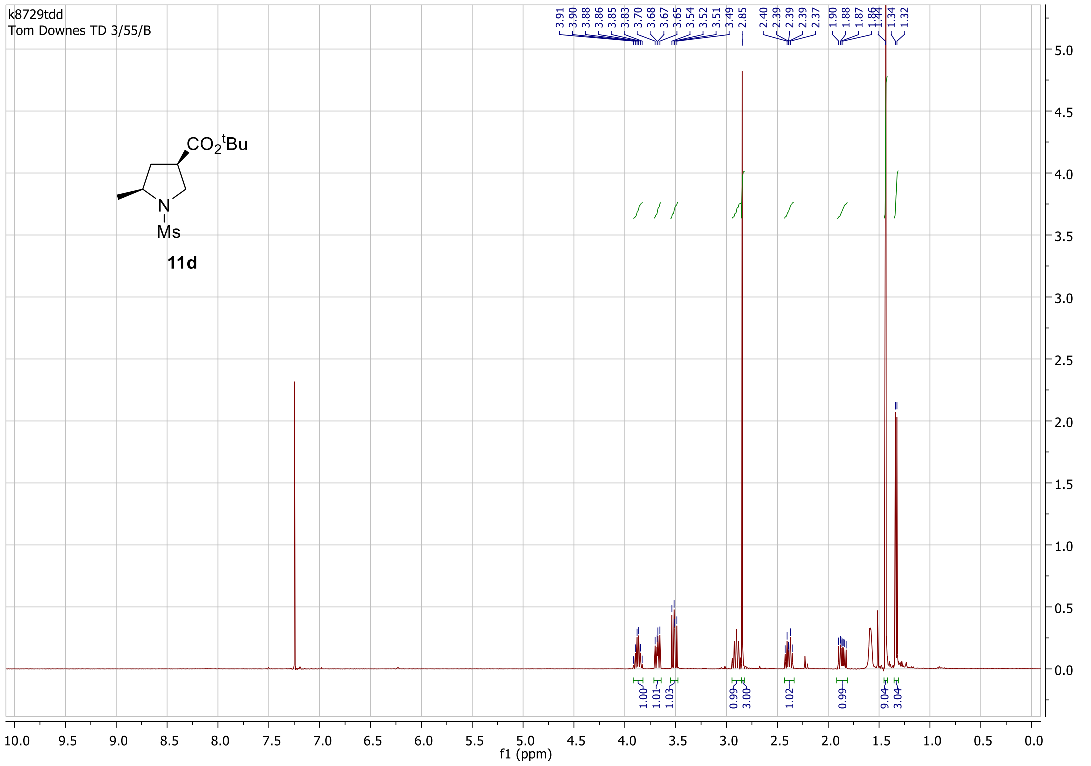

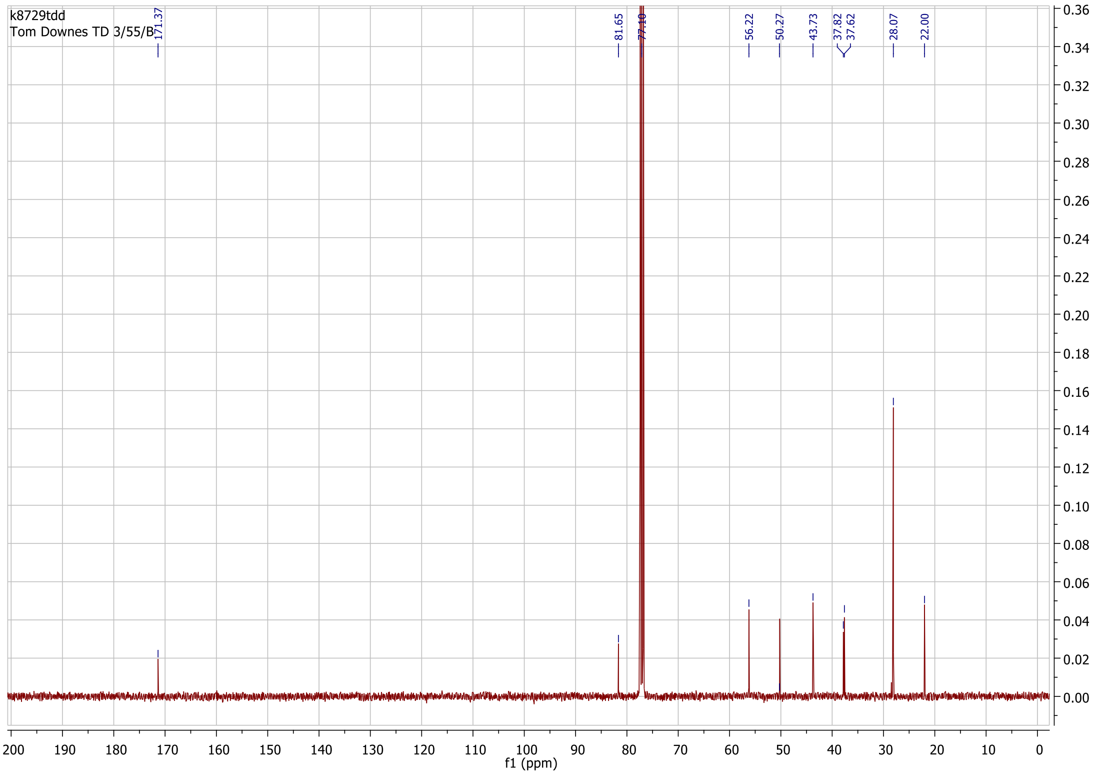

400 MHz ^{1}H NMR spectrum; 100.6 MHz ^{13}C NMR spectrum; $CDCl_3$

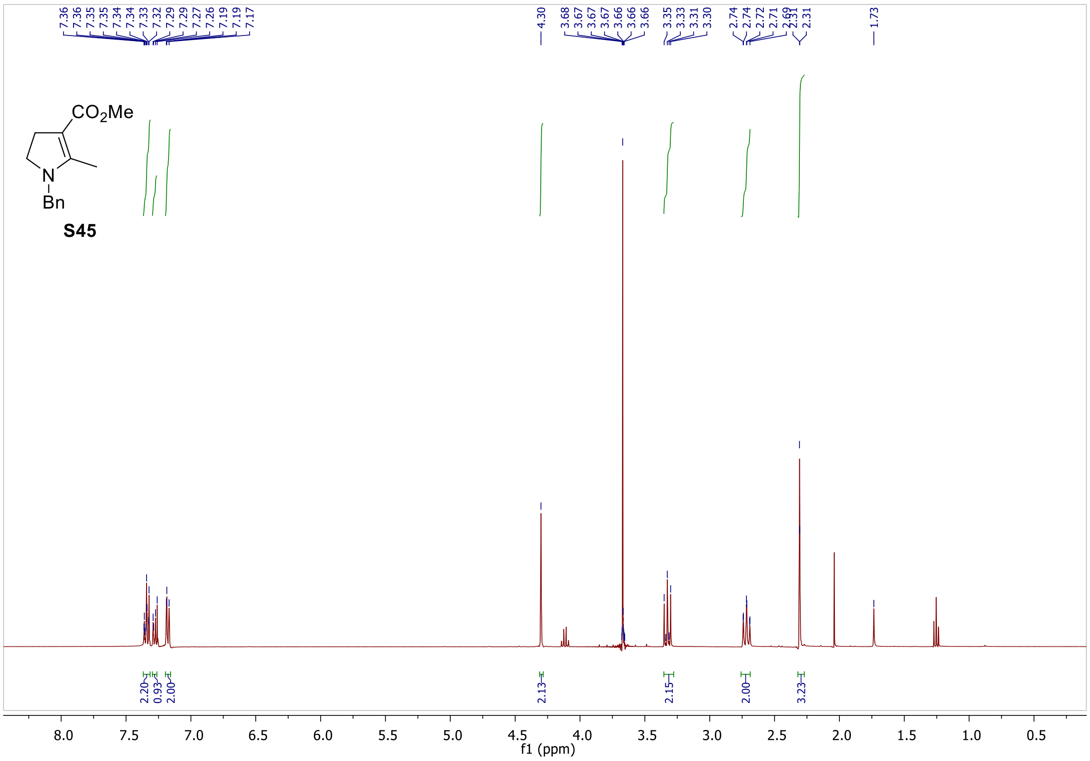

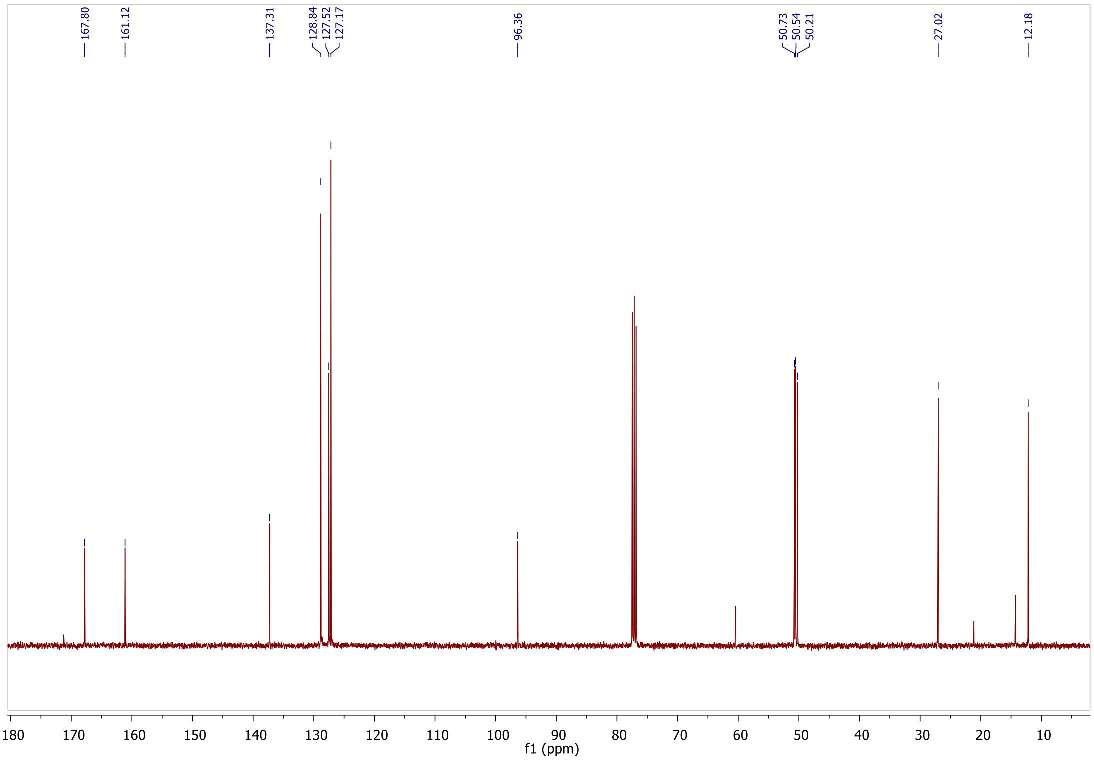

400 MHz ^{1}H NMR spectrum; 100.6 MHz ^{13}C NMR spectrum; $CDCl_3$

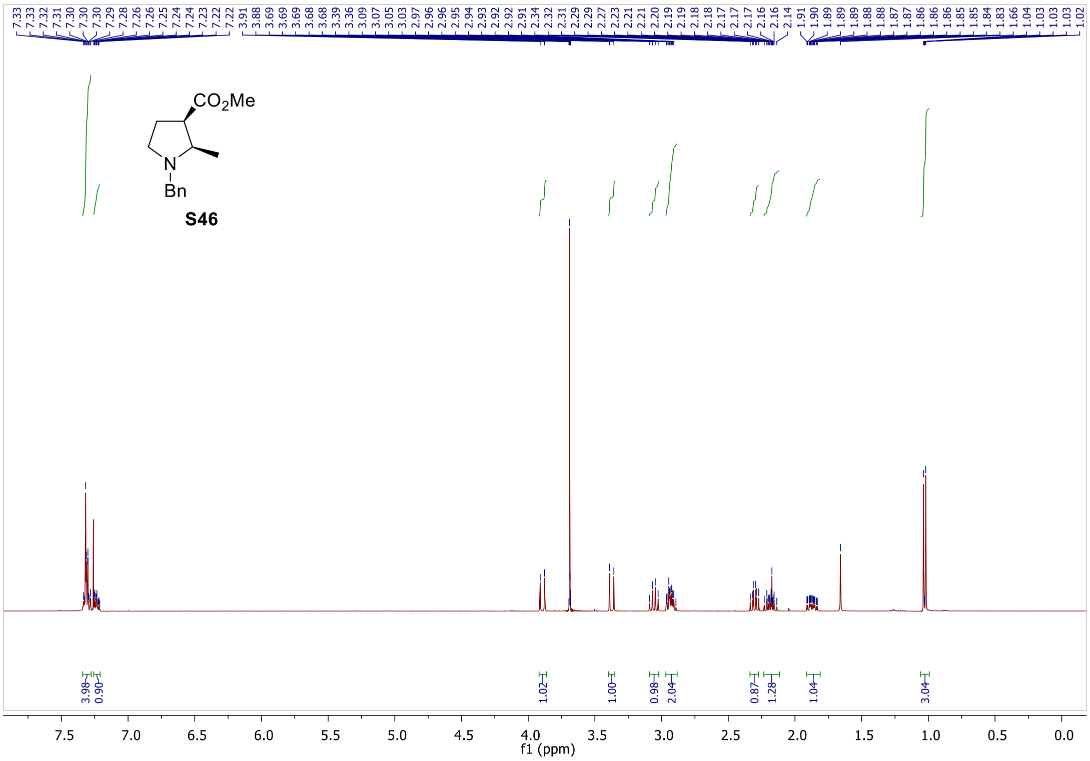

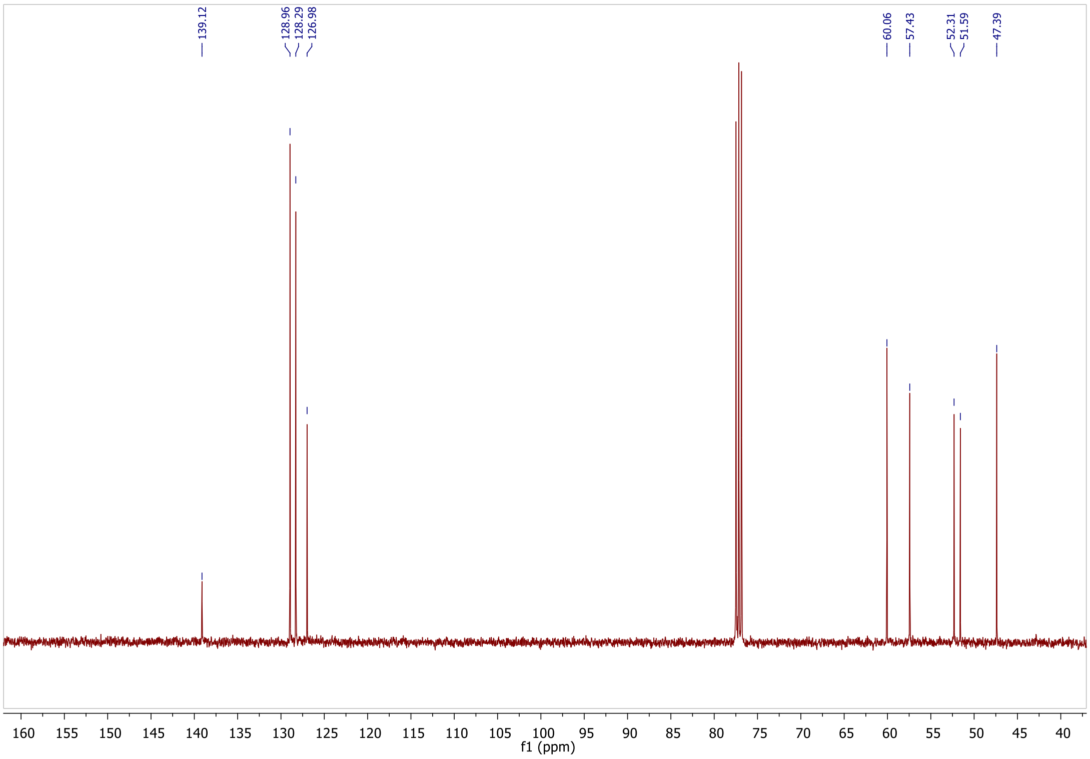

400 MHz ^{1}H NMR spectrum; 100.6 MHz ^{13}C NMR spectrum; $CDCl_3$

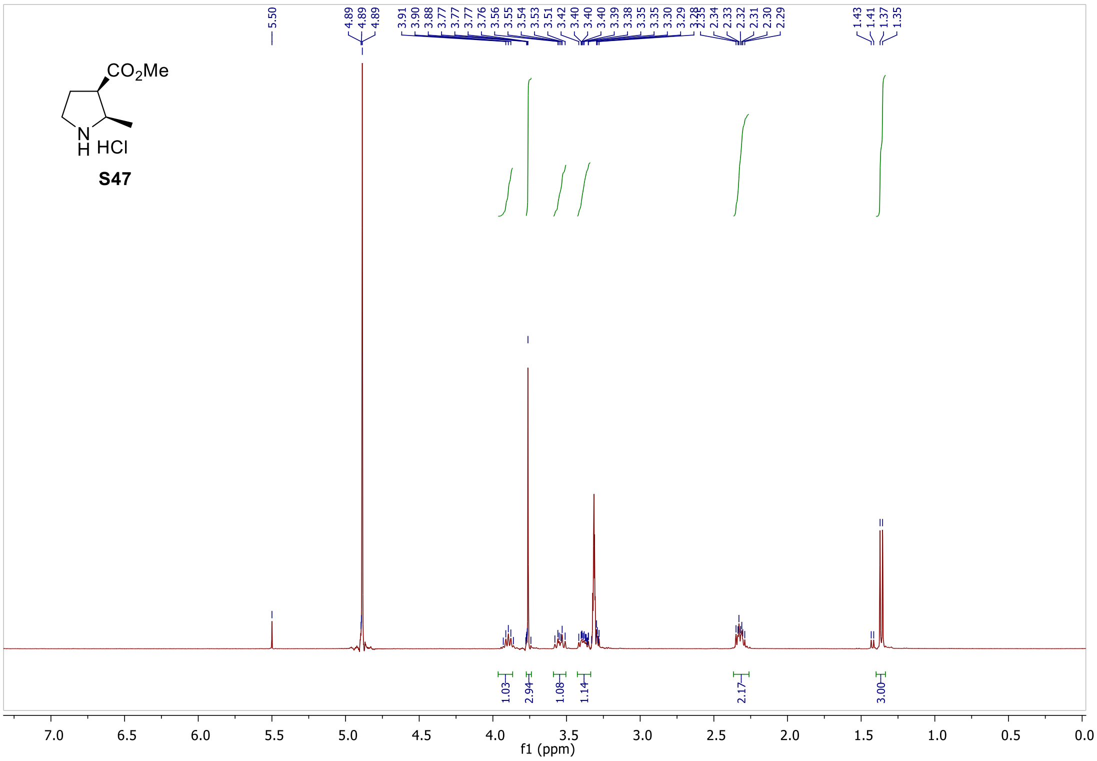

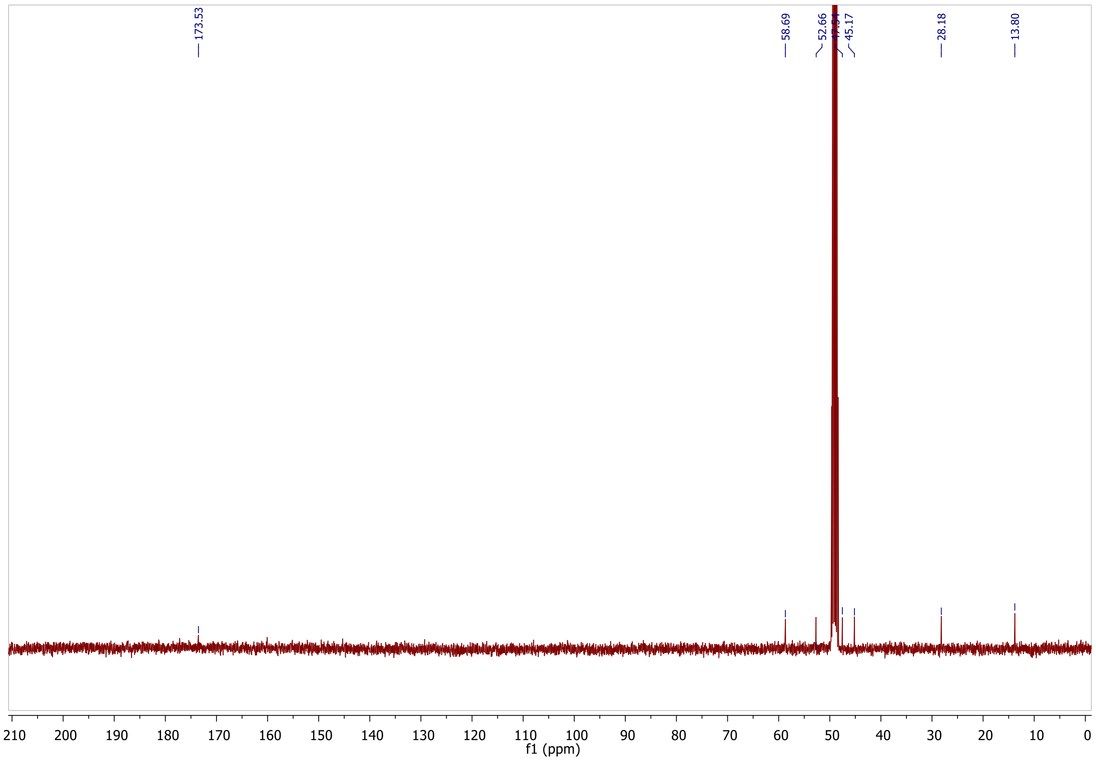

400 MHz ^1H NMR spectrum; 100.6 MHz ^{13}C NMR spectrum; $CDCl_3$

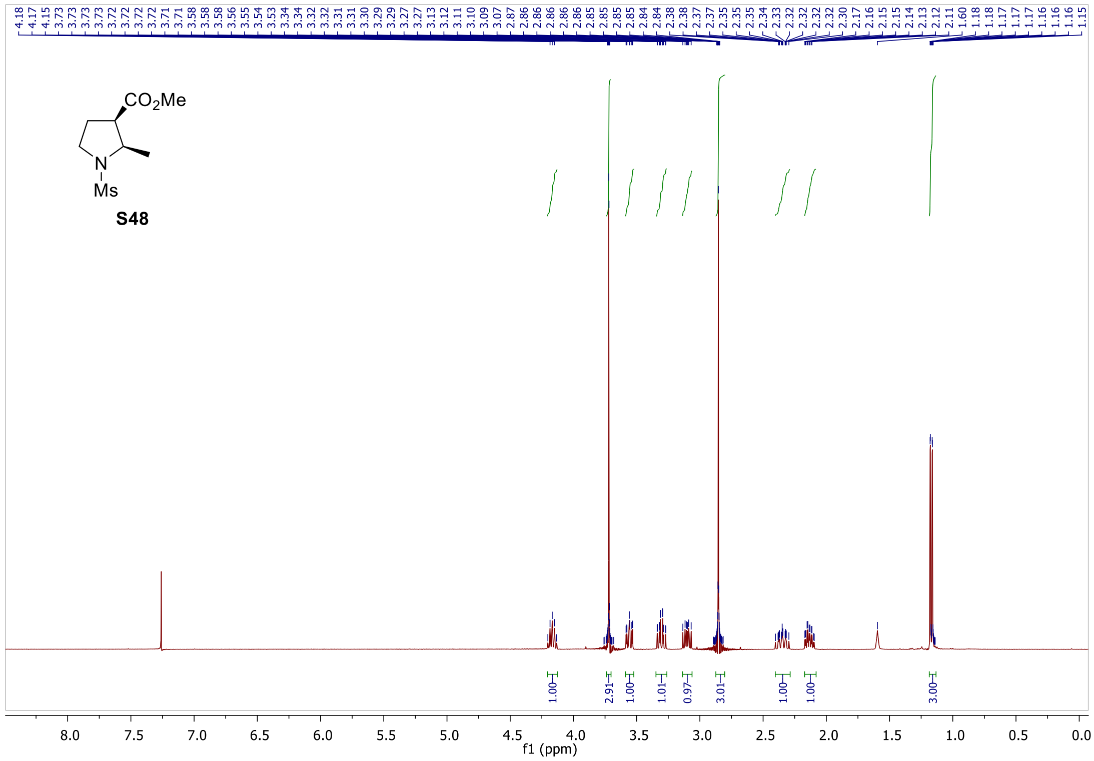

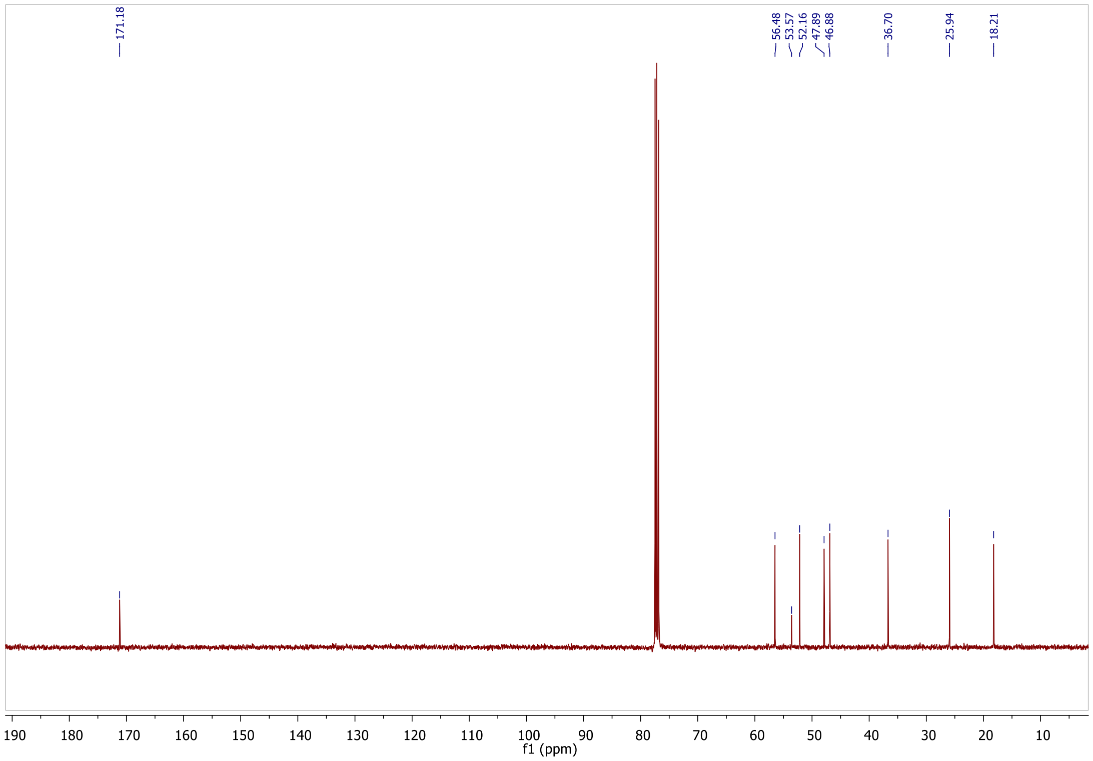

400 MHz ^{1}H NMR spectrum; 100.6 MHz ^{13}C NMR spectrum; $CDCl_3$

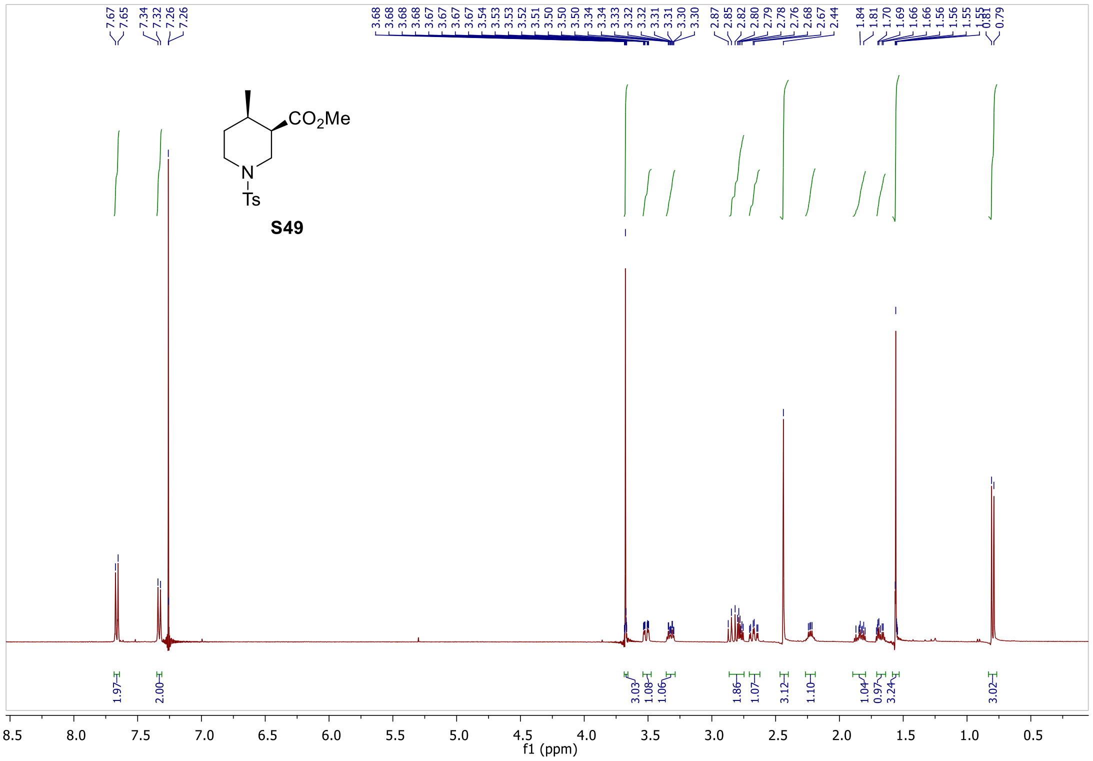

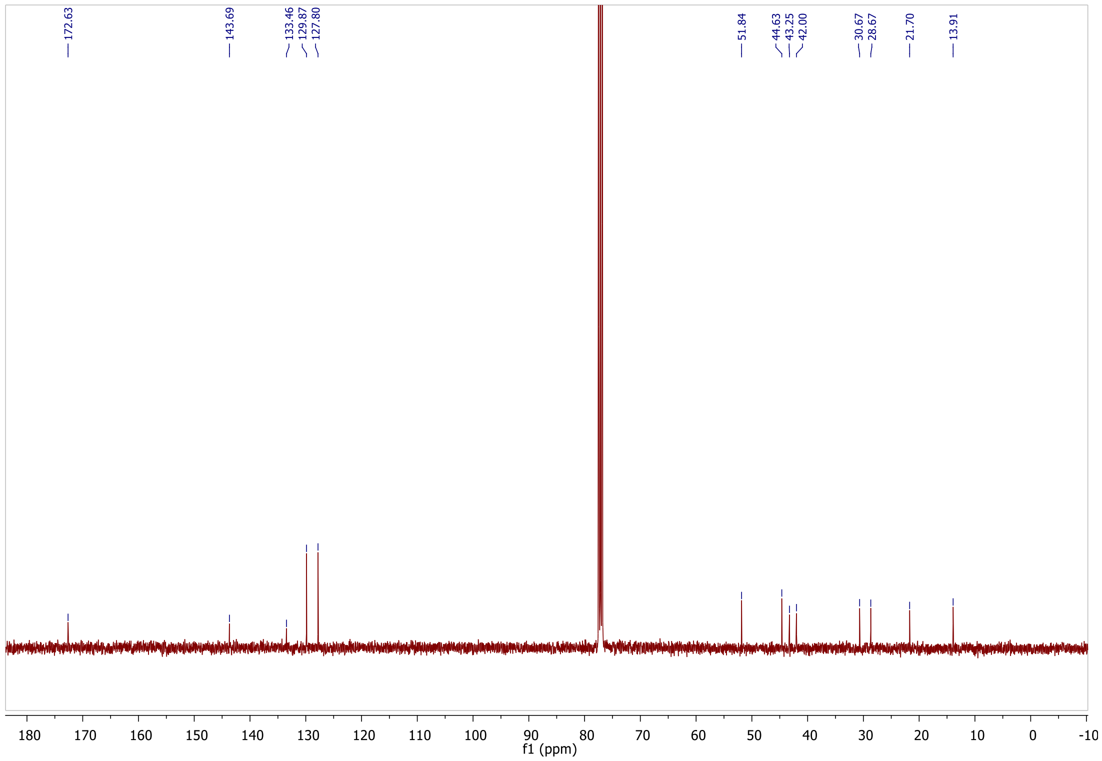

4. References

[1] P. N. Confalone, E. M. Huie, S. S. Ko, G. M. Cole, *J. Org. Chem.* **1988**, *53*, 482–487.

[2] F. Kelleher, S. Kelly, J. Watts, V. McKee, *Tetrahedron* **2010**, *66*, 3525–3536.

[3] A. Lewis, M. D. Ryan, D. Gani, *J. Chem. Soc. Perkin Trans. 1* **1998**, 3767–3776.

[4] B. Su, M. Deng, Q. Wang, *Eur. J. Org. Chem.* **2013**, *1*, 1979–1985.

[5] T. Sato, T. Yamazaki, Y. Nakanishi, J. Uenishi, M. Ikeda, *J. Chem. Soc. Perkin Trans. 1* **2002**, 1438–1443.

[6] A. Liljeblad, H.-M. Kavenius, P. Tähtinen, L. T. Kanerva, *Tetrahedron: Asymmetry* **2007**, *18*, 181–191.

[7] A. D. Dunn, *Org. Prep. Proced. Int.* **1999**, *31*, 120–123.

[8] R. J. Snow, R. Baker, R. H. Herbert, I. J. Hunt, K. J. Merchant, J. Saunders, *J. Chem. Soc. Perkin Trans. 1* **1991**, 409–420.

[9] E. Lorthiois, I. Marek, J. F. Normant, *J. Org. Chem.* **1998**, *63*, 566–574.

[10] R. Chênevert, M. Dickman, *J. Org. Chem.* **1996**, *61*, 3332–3341.

[11] F. Liéby-Muller, C. Allais, T. Constantieux, J. Rodriguez, *Chem. Commun.* **2008**, 4207–4209.

[12] C. Agami, L. Dechoux, C. Ménard, S. Hebbe, *J. Org. Chem.* **2002**, *67*, 7573–7576.

[13] E. P. Kyba, S. T. Liu, K. Chockalingam, B. R. Reddy, *J. Org. Chem.* **1988**, *53*, 3513–3521.

[14] E. Coudert, F. Acher, R. Azerad, *Synthesis* **1997**, 863–865.

[15] P. Dieterich, D. W. Young, *Org. Biomol. Chem.* **2006**, *4*, 1492–1496.

[16] M. D. Shoulders, J. A. Hodges, R. T. Raines, *J. Am. Chem. Soc.* **2006**, *128*, 8112–8113.

[17] D. Solé, X. Urbaneja, J. Bonjoch, *Adv. Synth. Catal.* **2004**, *346*, 1646–1650.

[18] H. Zhang, S. Mitsumori, N. Utsumi, M. Imai, N. Garcia-Delgado, M. Mifsud, K. Albertshofer, P. H.-Y. Cheong, K. N. Houk, F. Tanaka, et al., *J. Am. Chem. Soc.* **2008**, *130*, 875–886.

[19] R. Noël, C. Vanucci-Bacqué, M.-C. Fargeau-Bellassoued, G. Lhommet, *Eur. J. Org. Chem.* **2007**, *2007*, 476–486.

[20] C. M. Pedersen, M. Bols, *Tetrahedron* **2005**, *61*, 115–122.

[21] F. Kelleher, S. Kelly, J. Watts, V. McKee, *Tetrahedron* **2010**, *66*, 3525–3536.

[22] E. Vedejs, F. G. West, *J. Org. Chem.* **1983**, *48*, 4773–4774.

[23] G. Haviari, J. P. Célérier, H. Petit, G. Lhommet, D. Gardette, J. C. Gramain, *Tetrahedron Lett.* **1992**, *33*, 4311–4312.

[24] M. Irfan, E. Petricci, T. N. Glasnov, M. Taddei, C. O. Kappe, *Eur. J. Org. Chem.* **2009**, *2009*, 1327–1334.

[25] J. R. Medina, C. J. Becker, C. W. Blackledge, C. Duquenne, Y. Feng, S. W. Grant, D. Heerding, W. H. Li, W. H. Miller, S. P. Romeril, et al., *J. Med. Chem.* **2011**, *54*, 1871–1895.

[26] Y. Xu, J. Choi, M. Isabel Calaza, S. Turner, H. Rapoport, *J. Org. Chem.* **1999**, *64*, 4069–4078.
